# Correction: Lien et al. Sample Preparation Approach Influences PAM50 Risk of Recurrence Score in Early Breast Cancer. *Cancers* 2021, *13*, 6118

**DOI:** 10.3390/cancers18091337

**Published:** 2026-04-23

**Authors:** Tonje G. Lien, Hege Oma Ohnstad, Ole Christian Lingjærde, Johan Vallon-Christersson, Marit Aaserud, My Anh Tu Sveli, Åke Borg, on behalf of OSBREAC, Øystein Garred, Elin Borgen, Bjørn Naume, Hege Russnes, Therese Sørlie

**Affiliations:** 1 Department of Cancer Genetics, Institute for Cancer Research, Oslo University Hospital, P.O. Box 4953 Nydalen, N-0424 Oslo, Norway; tonje.lien@rr-research.no (T.G.L.); ole@ifi.uio.no (O.C.L.); h.e.g.russnes@ous-research.no (H.R.); 2 Department of Oncology, Division of Cancer Medicine, Oslo University Hospital, P.O. Box 4953 Nydalen, N-0424 Oslo, Norway; hegeo@ous-hf.no (H.O.O.); bjorn.naume@medisin.uio.no (B.N.); 3 Centre for Bioinformatics, Department of Informatics, University of Oslo, Gaustadalléen 23 B, N-0373 Oslo, Norway; 4 Division of Oncology, Department of Clinical Sciences Lund, Lund University, Medicon Village, SE-22381 Lund, Sweden; johan.vallon-christersson@med.lu.se (J.V.-C.); ake.borg@med.lu.se (Å.B.); 5 Department of Pathology, Oslo University Hospital, P.O. Box 4953 Nydalen, N-0424 Oslo, Norway; mhelg@ous-hf.no (M.A.); myahtu@ous-hf.no (M.A.T.S.); uxysga@ous-hf.no (Ø.G.); ebg@ous-hf.no (E.B.); 6 Institute of Clinical Medicine, Faculty of Medicine, University of Oslo, P.O. Box 1171 Blindern, N-0318 Oslo, Norway

## Text Correction

There are two text errors in the original publication [1]. These errors concern the type of data contained within the supplemental tables. The words that were mistakenly included indicate the type of data that should have been deleted before final submission. The words ‘RNA-Seq’ and ‘nCounter’ have now been removed from the respective paragraphs below. The data in the tables remains correctly described.

A correction has been made to Materials and Methods, RNA Extraction and Gene Expression Analysis from FFPE Tissue, Paragraph 1.

From FFPE tissue, RNA purification was performed using the Roche^®^ FFPET RNA Isolation Kit, Roche-025 (NanoString Technologies, Seattle, WA, USA): 1–6 FFPE slides depending on the tumor area, (4–19 mm^2^: 6 slides, 20–99 mm^2^: 3 slides, and ≥100 mm^2^: 1 slide). The recommended RNA input was 250 ng for the hybridizations on the nCounter Analysis System. Two assays were employed: first the Prosigna assay (reporting the final ROR and subtype of each sample, i.e., not the correlation to the subtypes), and second the PAM50 assay (reporting the raw gene expression counts for the 50 breast-cancer-related genes). The raw counts were then normalized using all housekeeping genes and, finally, log2-transformed. The normalized data from the PAM50 genes can be found in Table S2.

A correction has been made to Materials and Methods, RNA Extraction and Gene Expression Analysis from FF Tissue, Paragraph 1.

RNA extraction from FF tissue and subsequent sequencing using Illumina NextSeq500 has previously been described in detail [11]. The raw counts were processed as described in [12], and the resulting FPKM (fragments per kilobase per million) gene expression values were log2-transformed prior to selecting the PAM50 genes for further analysis. Tumor vs. normal cell content in all FF tissue specimens used for RNA extraction was evaluated by a pathologist. The normalized log2-transformed counts for the PAM50 genes can be found in Table S3.

## Error in Data Availability Statement

In the original publication, there was a text error in the Data Availability Statement. The sentences “Normalized log2-transformed nCounter counts for the PAM50 genes can be found in Table S6. Normalized RNA-Seq data from the PAM50 genes can be found in Table S7” have now been deleted from the statement. During the final stage of manuscript preparation, when supplemental Tables S6 and S7 were finalized, this sentence should have been removed.

The authors state that the scientific conclusions are unaffected. This correction has been approved by the Academic Editor. The original publication has also been updated.
