# Supplementary material for: Sample Preparation Approach Influences PAM50 Risk of Recurrence Score in Early Breast Cancer
Source: Cancers (Basel). 2021 Dec 4;13(23):6118. doi: 10.3390/cancers13236118 (PMC8657125; doi:10.3390/cancers13236118)
Supplement: Supplementary file 1 [file cancers-13-06118-s001.zip › TableS4_S5_S6_S7.pdf]

Supplementary Table S4: Comparison of subtype calls using Subtype-Prosigna and Subtype-Macro. Both methods used the counts from nCounter based on analysis of FFPE tissue. Subtype-Macro in rows and Subtypes-Prosigna in columns.

**Macrodissected FFPE**		**Subtype-Prosigna**				
		Basal-like	HER2-enriched	Luminal A	Luminal B	Normal-like (not applicable)
**Subtype-Macro**	Basal-like	12				-
	HER2-enriched	1	5	2	1	-
	Luminal A			46	8	-
	Luminal B		1		17	-
	Normal-like			1		-

Supplementary Table S5: ROR, subtype and correlation to the five gene expression centroids for the 14 discordant cases. The correlation values are from the Subtype-Macro algorithm based on the FFPE tumor tissue.

**SampleID**	**ROR-Prosigna**	**Subtype-Prosigna**	**Subtype-Macro**	**Basal-like**	**HER2-enriched**	**Luminal-A**	**Luminal-B**	**Normal-like**
BC-06	79	HER2-enriched	Luminal B	-0.26	0.27	0.12	0.39	-0.18
BC-14	60	Luminal B	Luminal A	-0.35	-0.28	0.43	0.42	-0.24
BC-19	62	Luminal B	Luminal A	-0.37	-0.1	0.5	0.23	-0.23
BC-27	61	Luminal B	Luminal A	-0.29	-0.26	0.26	0.18	-0.1
BC-28	72	Basal-like	HER2-enriched	0.42	0.55	-0.6	-0.05	-0.31
BC-38	55	Luminal B	Luminal A	-0.3	0.03	0.23	0.19	-0.18
BC-47	60	Luminal B	Luminal A	-0.47	0	0.49	0.47	-0.27
BC-52	19	Luminal A	Normal-like	-0.14	-0.28	0.5	-0.51	0.68
BC-55	47	Luminal B	Luminal A	-0.7	0.09	0.7	0.49	-0.34
BC-58	46	Luminal A	HER2-enriched	-0.39	0.26	0.23	0.06	-0.19
BC-61	68	Luminal B	Luminal A	-0.56	-0.04	0.59	0.5	-0.38
BC-63	42	Luminal A	HER2-enriched	0.18	0.34	-0.3	-0.42	0.27
BC-65	62	Luminal B	HER2-enriched	-0.43	0.36	0.1	0.33	-0.5
BC-88	57	Luminal B	Luminal A	-0.71	0.08	0.63	0.59	-0.41

Supplementary Table S6: Comparison of subtype calls using Subtype-Macro and the genefu package. Both methods used the counts from nCounter based on analysis of FFPE tissue. Subtype-Macro in rows and the genefu subtype in columns.

**Macrodissected FFPE**		**Genefu**				
		Basal-like	HER2-enriched	Luminal A	Luminal B	Normal-like
**Subtype-Macro**	Basal-like	12				
	HER2-enriched		8	1		
	Luminal A			48	6	
	Luminal B			1	17	
	Normal-like			1		

Supplementary Table S7: Comparison of subtype calls in FFPE and FF tissue-derived data using genefu. Gene expression data from macrodissected FFPE tumor tissue in rows and gene expression data from FF bulk tissue in columns.

**Subtype method: Genefu**		**FF bulk**				
		Basal-like	HER2-enriched	Luminal A	Luminal B	Normal-like
**Macro-dissected FFPE**	Basal-like	12				
	HER2-enriched		6	1		1
	Luminal A			39	4	8
	Luminal B			2	19	2
	Normal-like					
