# Supplementary figures and images for: Sample Preparation Approach Influences PAM50 Risk of Recurrence Score in Early Breast Cancer
Source: Cancers (Basel). 2021 Dec 4;13(23):6118. doi: 10.3390/cancers13236118 (PMC8657125; doi:10.3390/cancers13236118)

Supplementary Figure S1

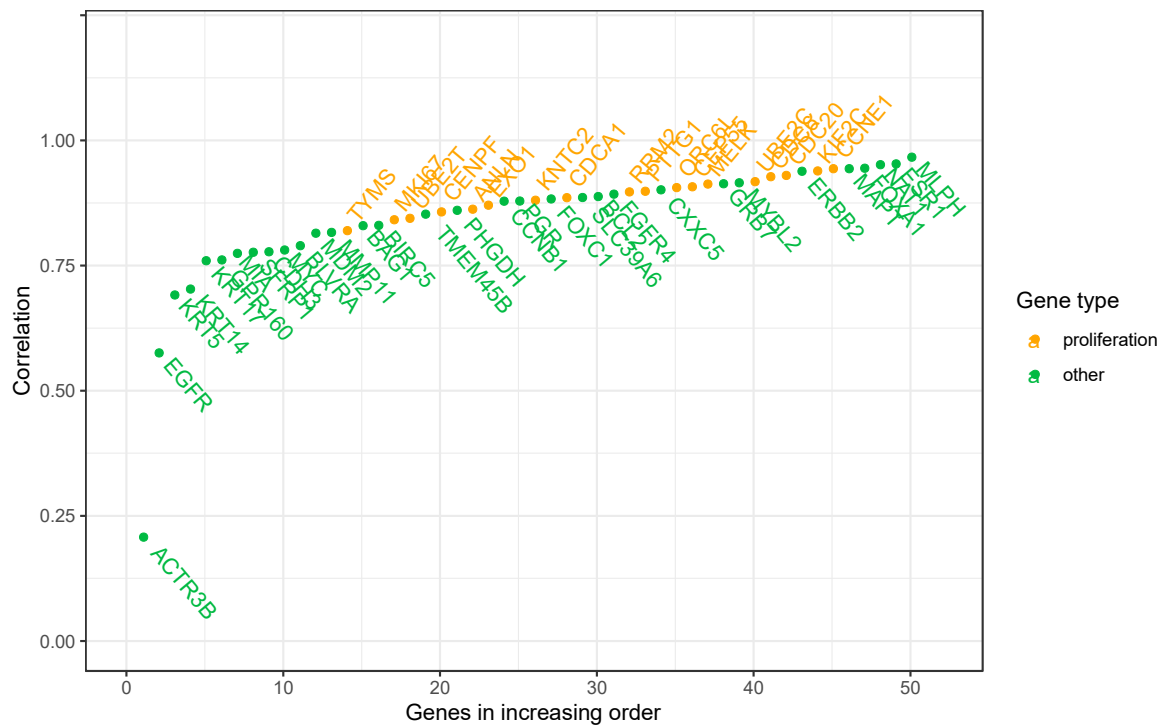

Supplementary Figure S2

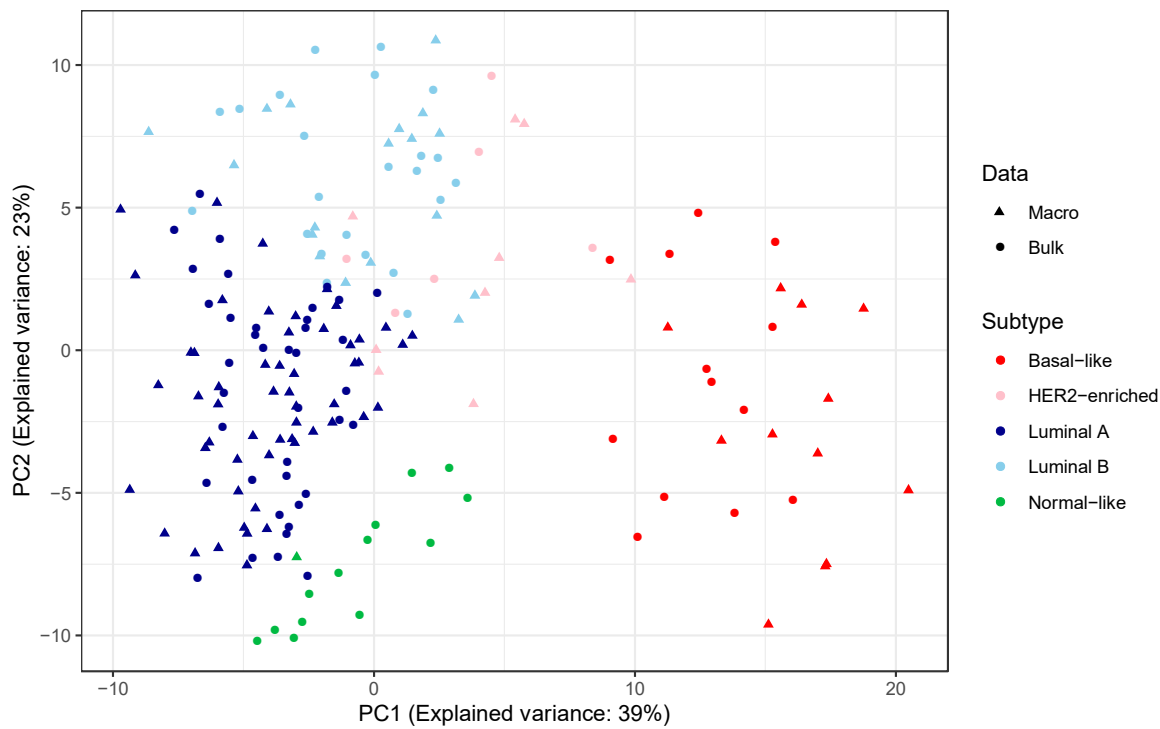

Supplement: Supplementary file 1 [file cancers-13-06118-s001.zip › Figure S1 and S2.pdf]

# ESR1 with ER cut off at 2.36

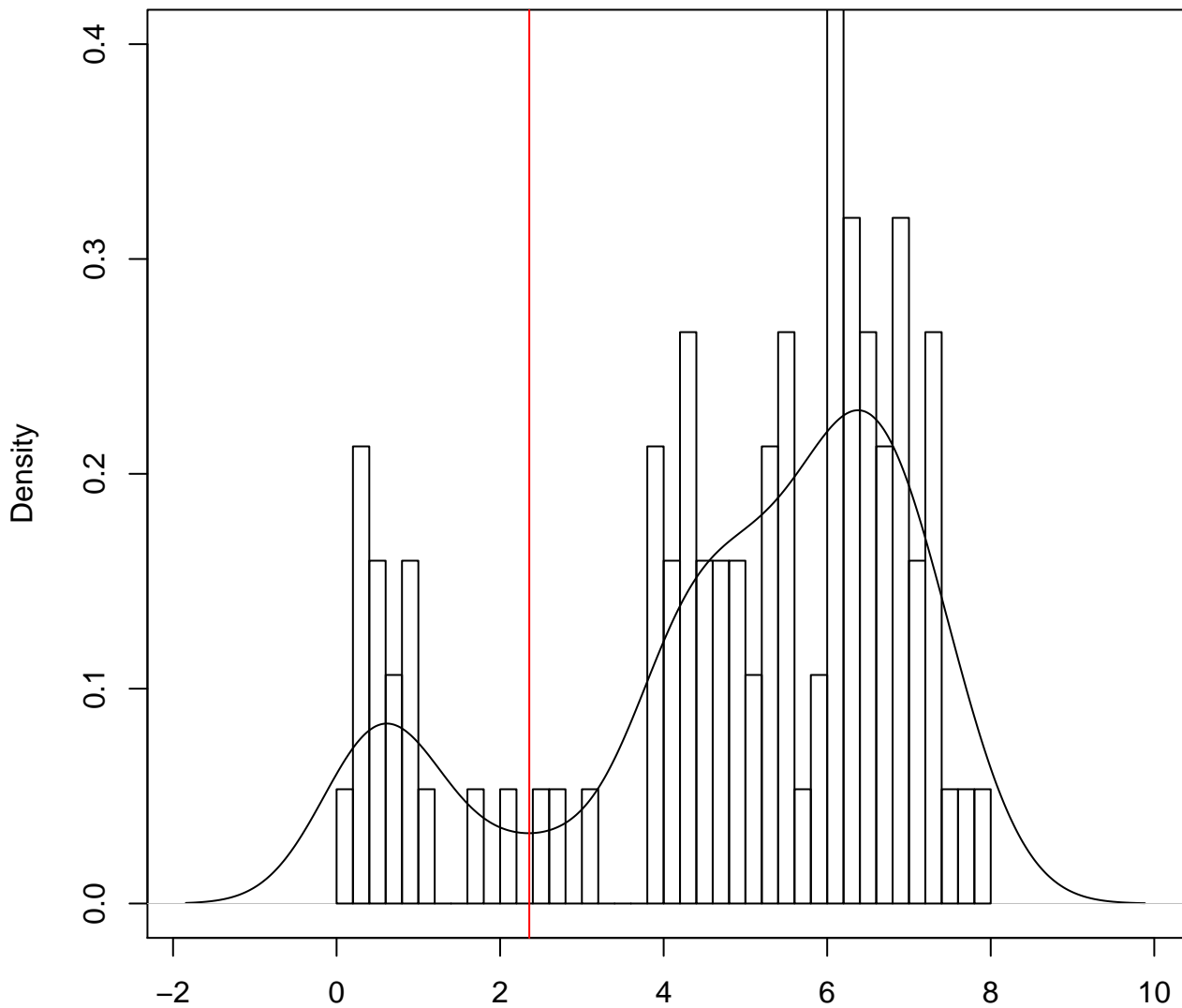

Supplement: Supplementary file 1 [file cancers-13-06118-s001.zip › SupplementaryCode/kodeTilSubmition/dataOutput/bulk/distribution_ESR1.pdf]

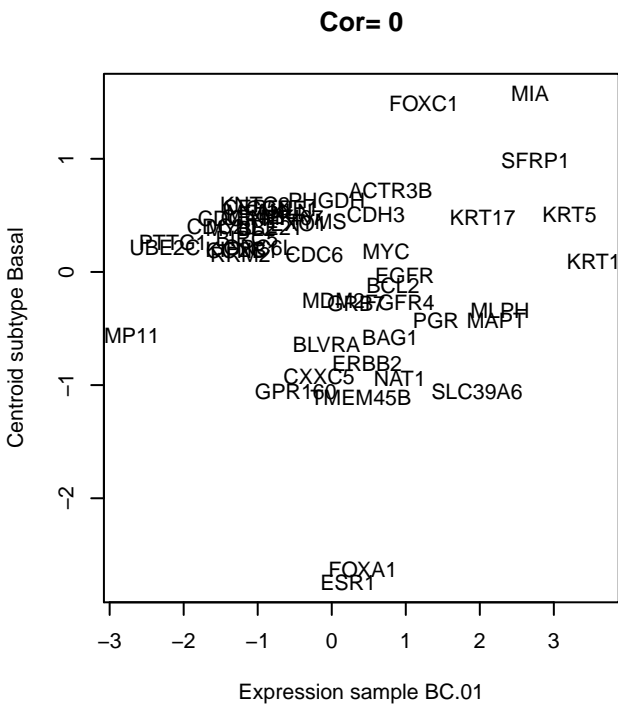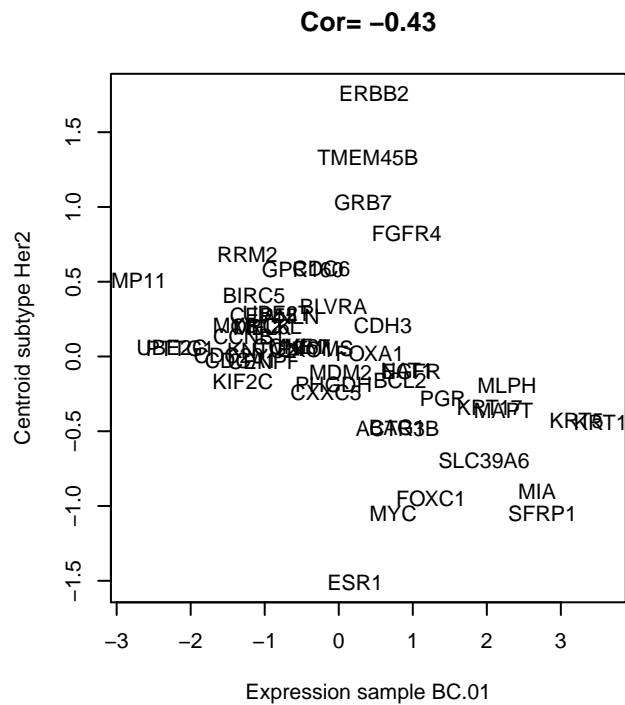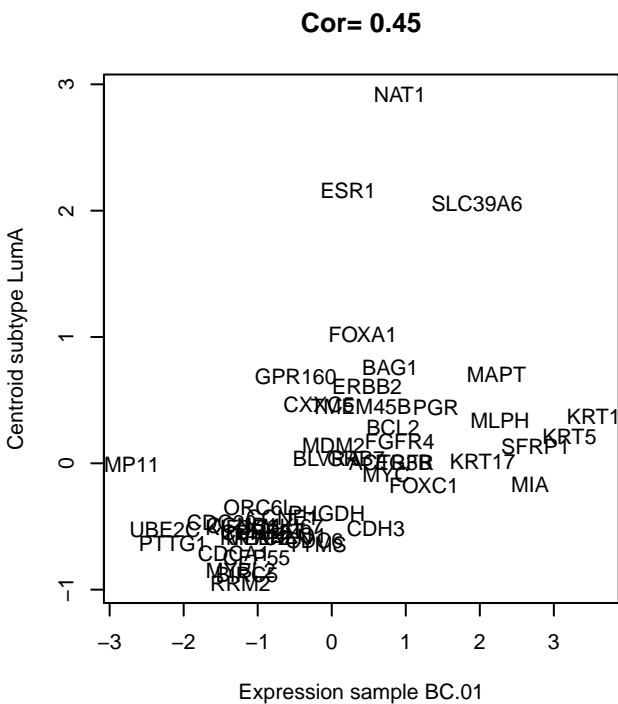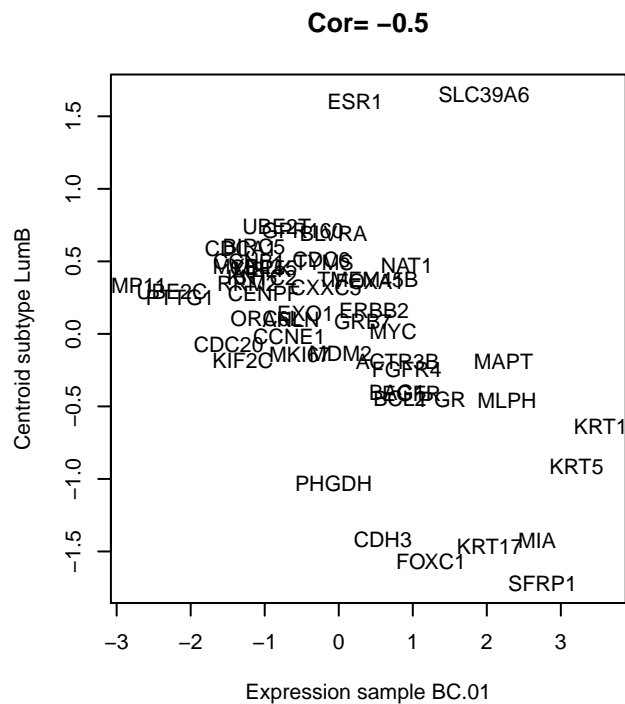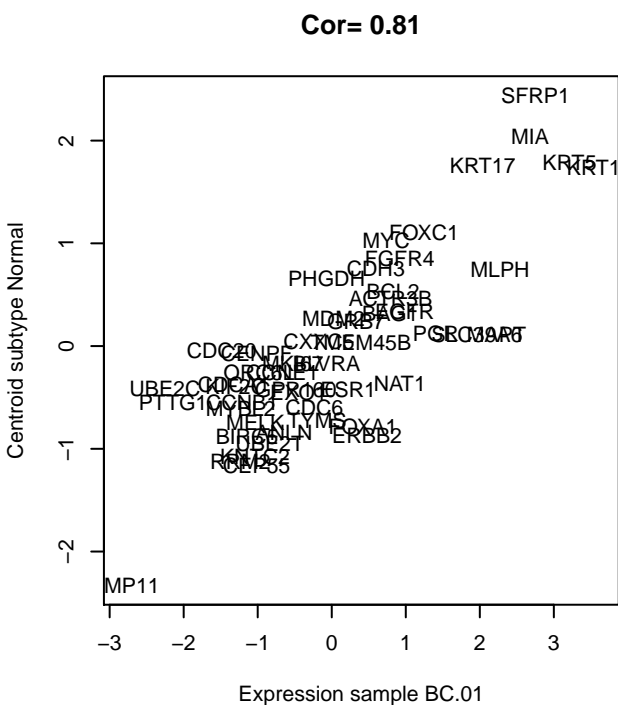



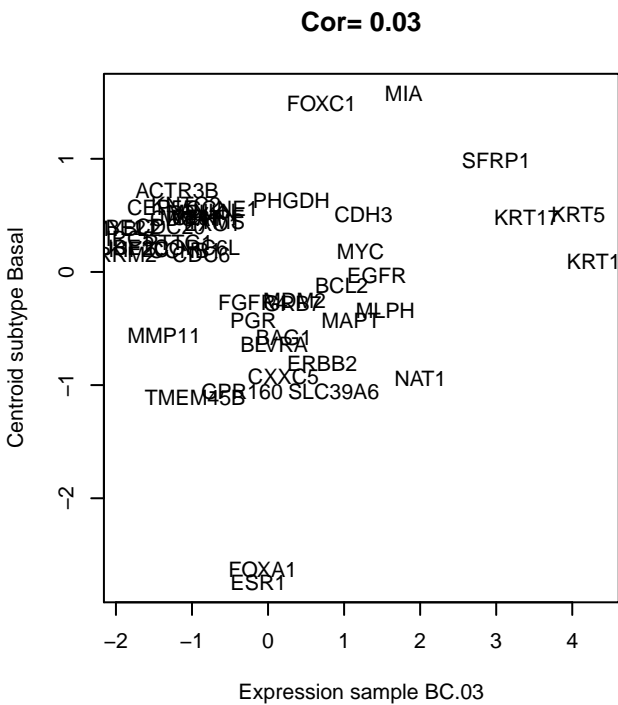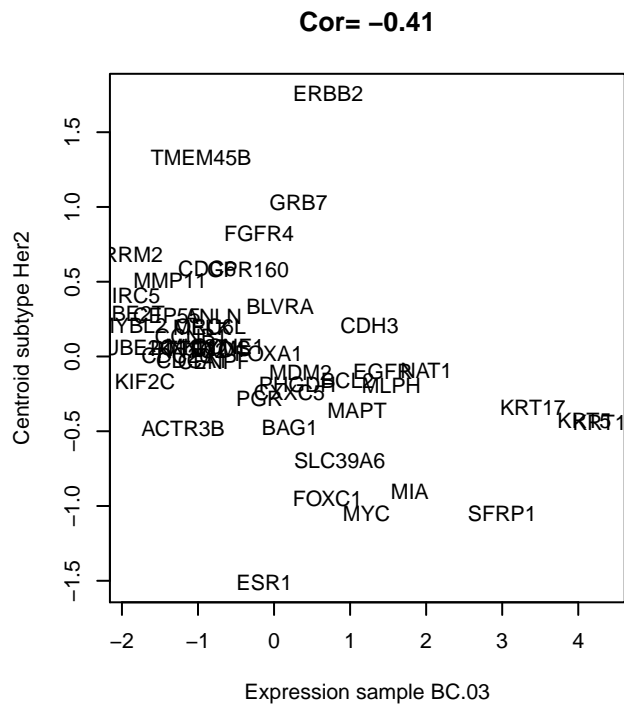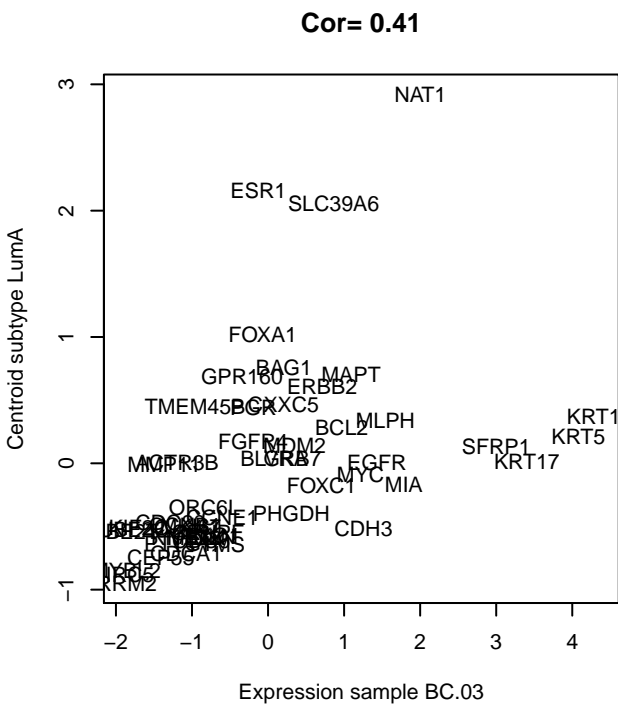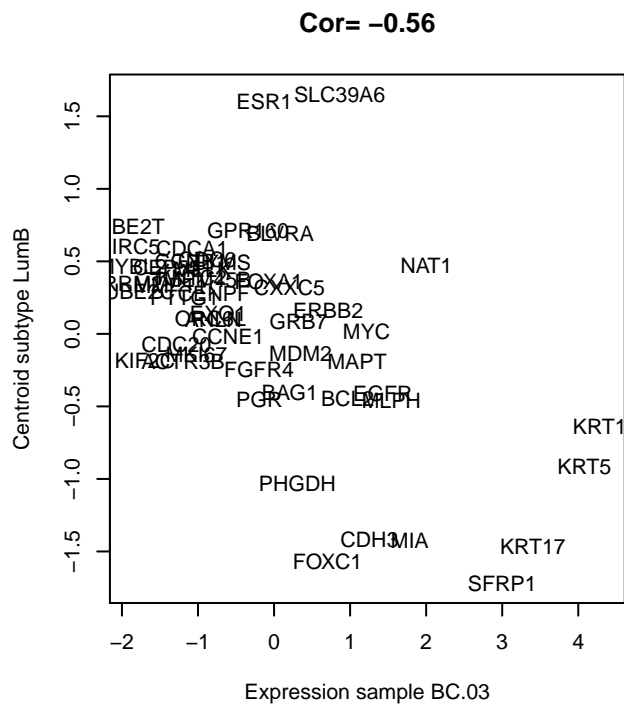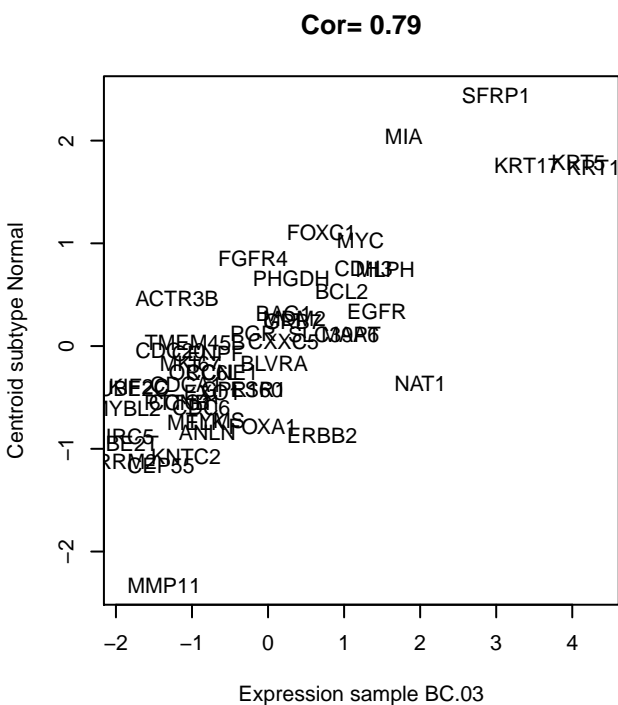



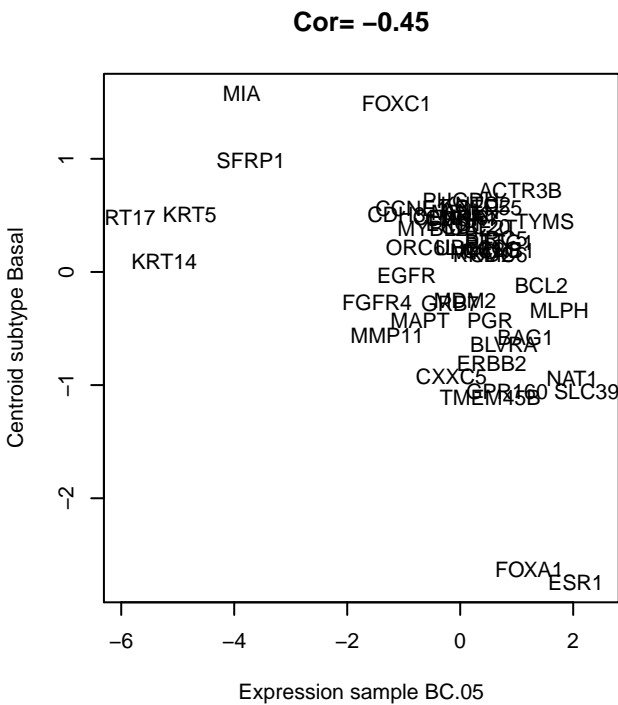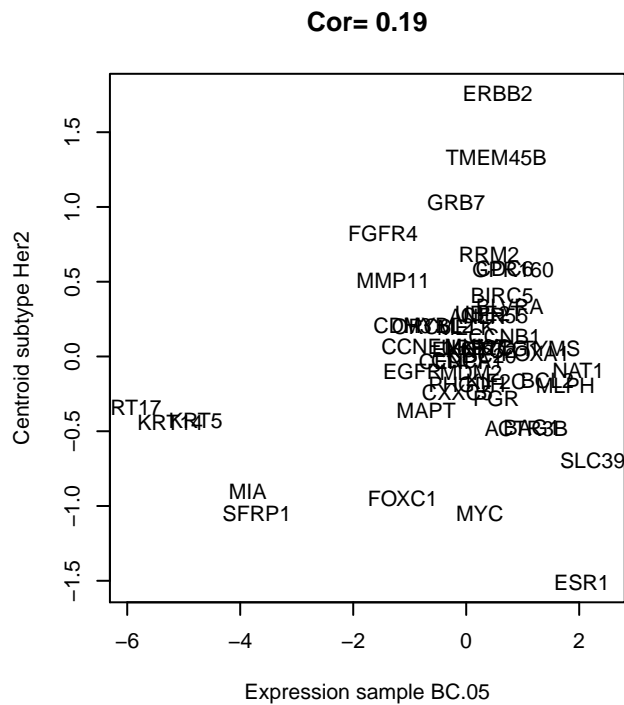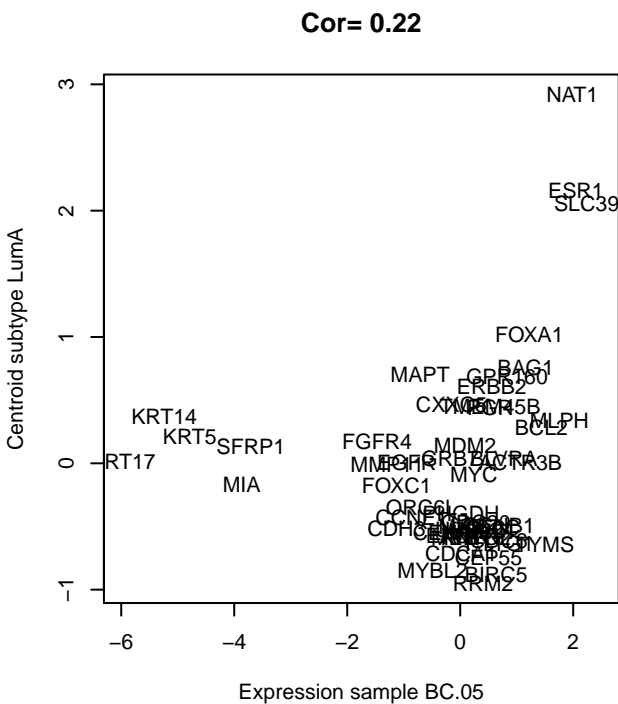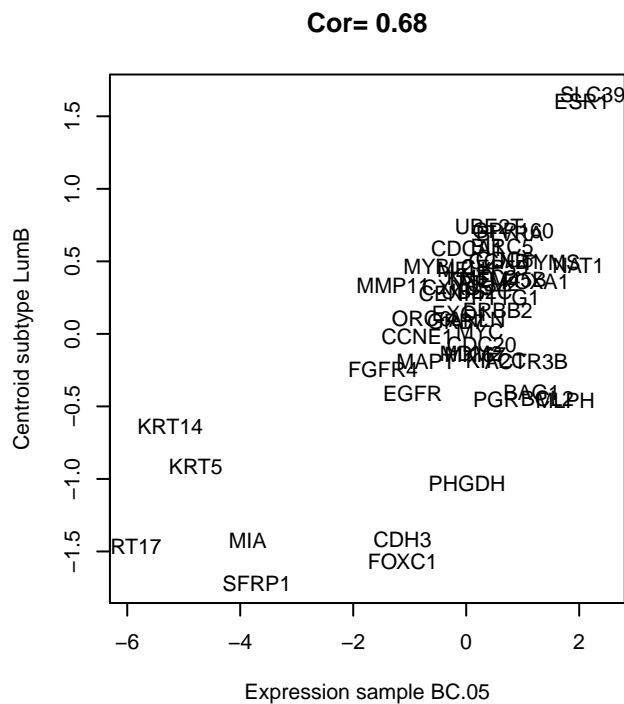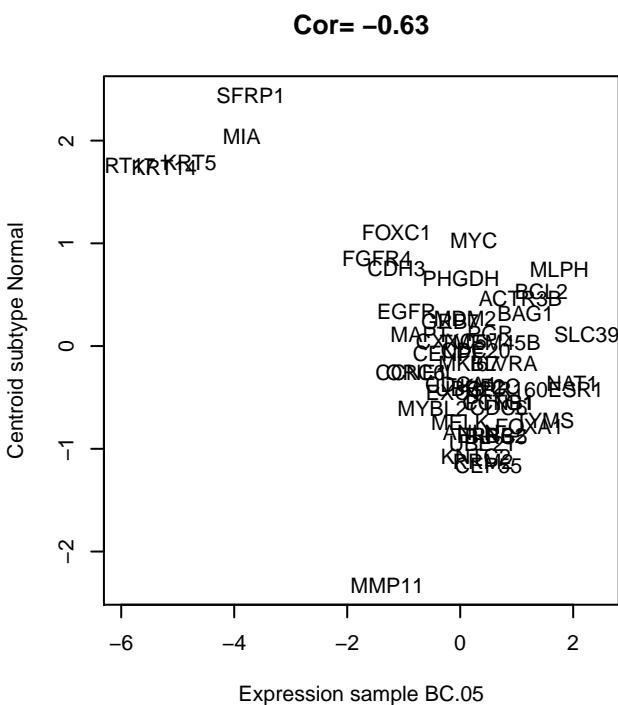

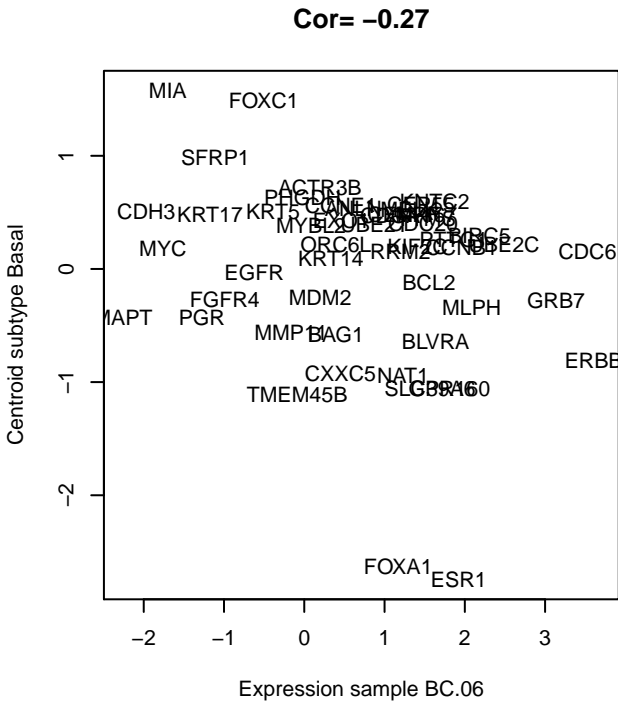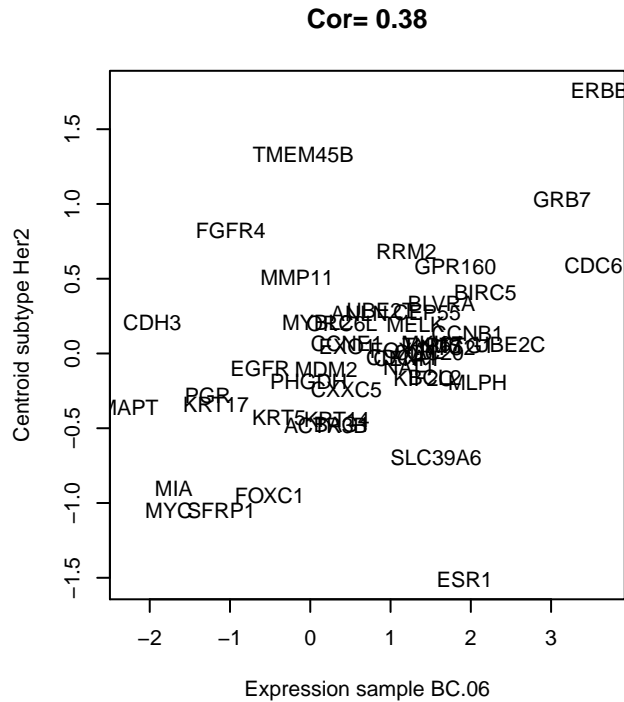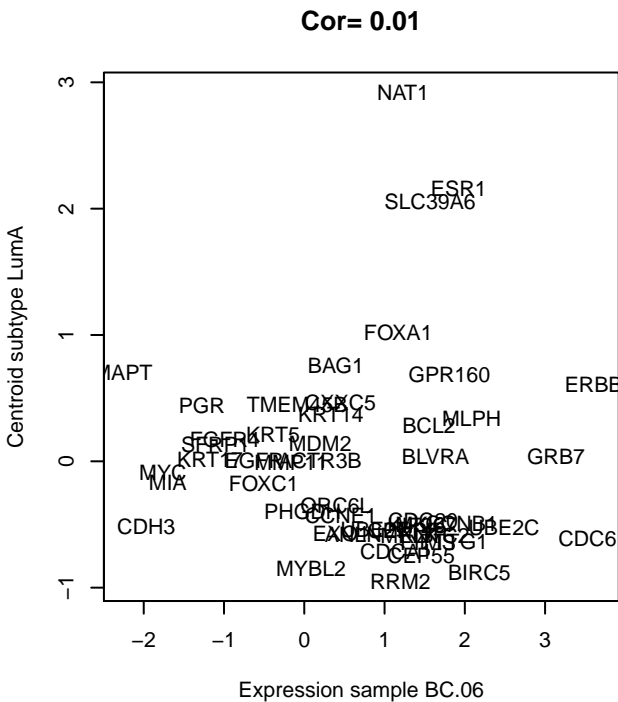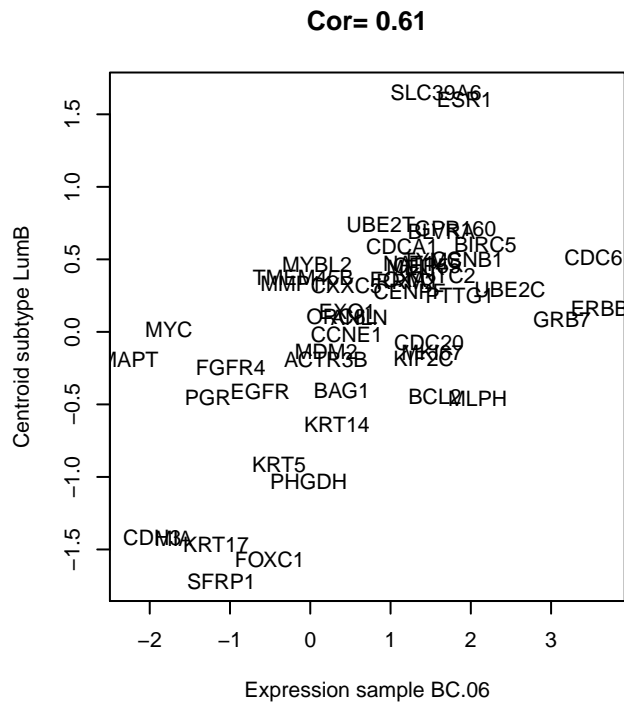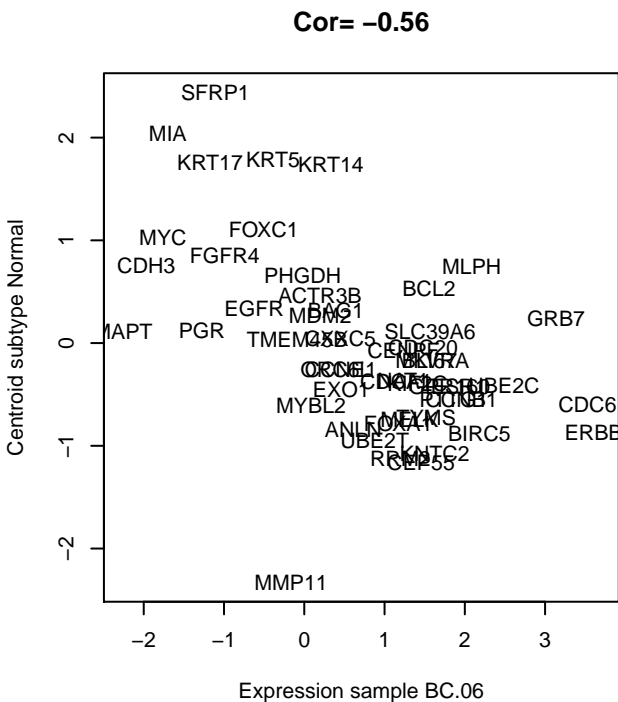

**Cor= -0.23**

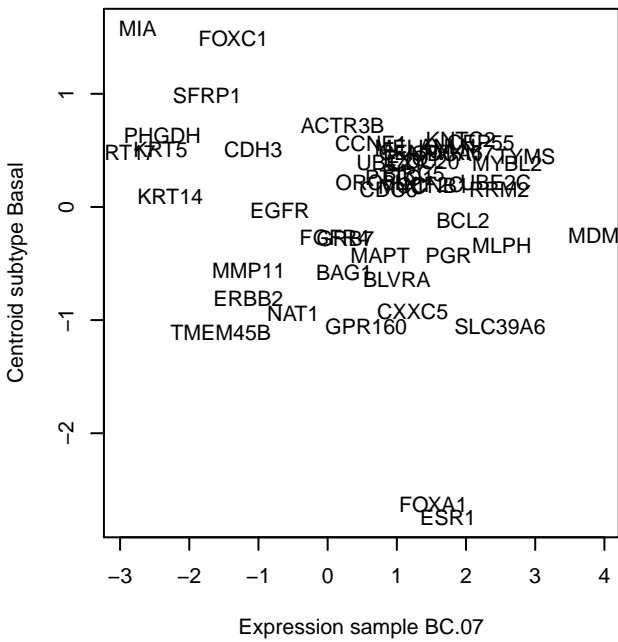

**Cor= 0.05**

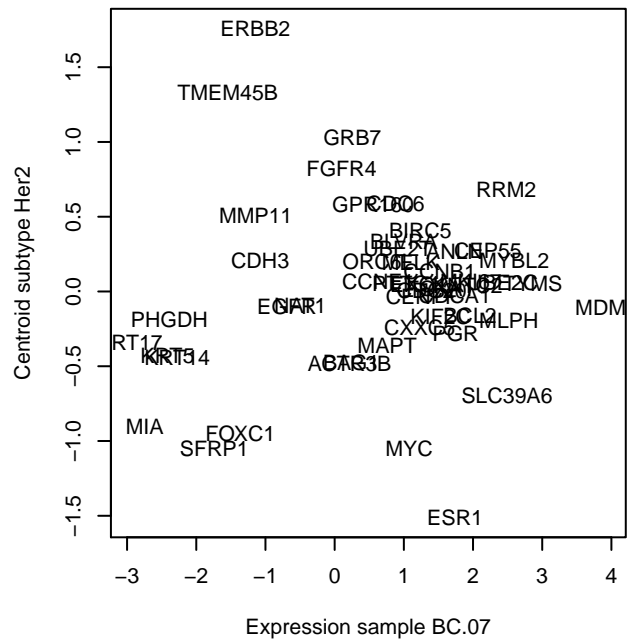

**Cor= -0.1**

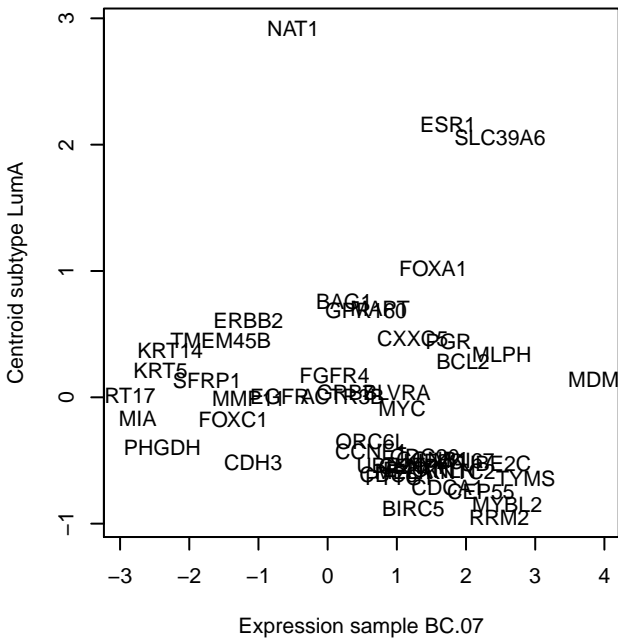

**Cor= 0.62**

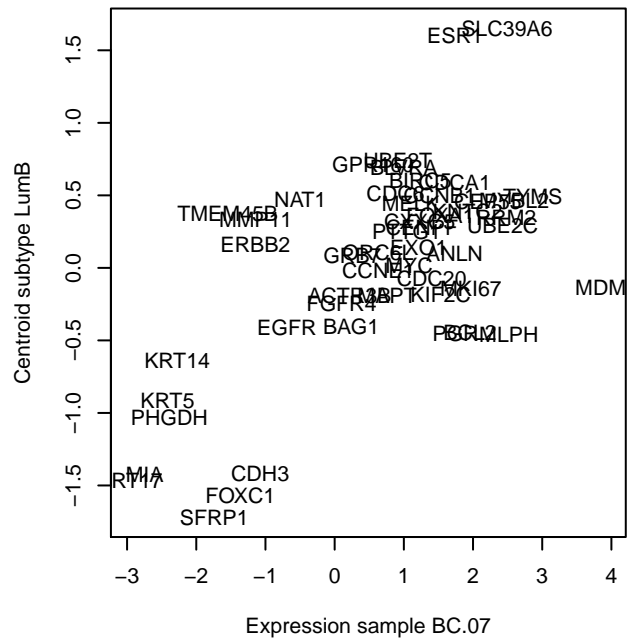

**Cor= -0.54**

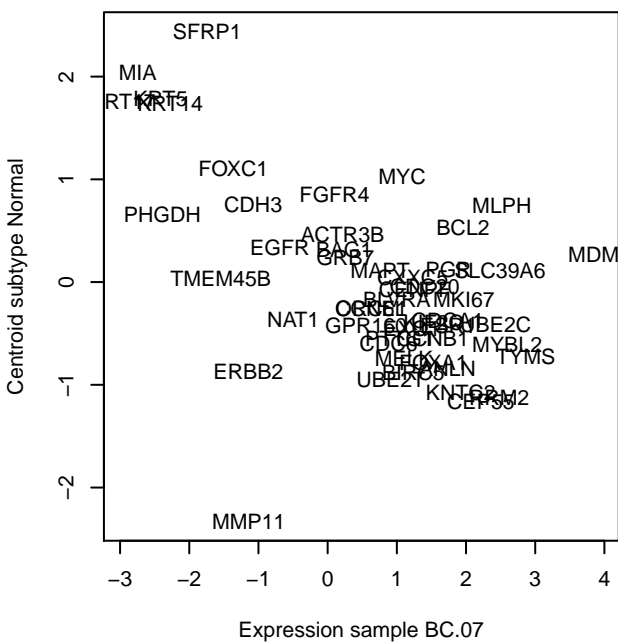



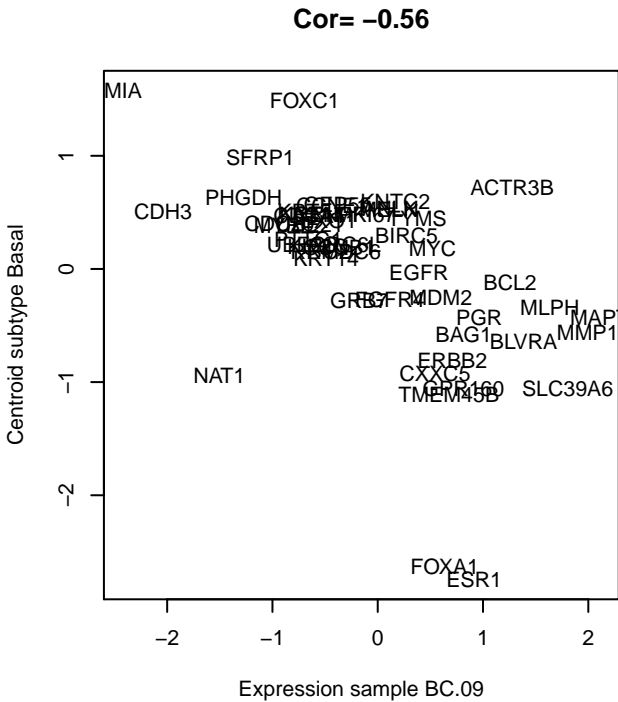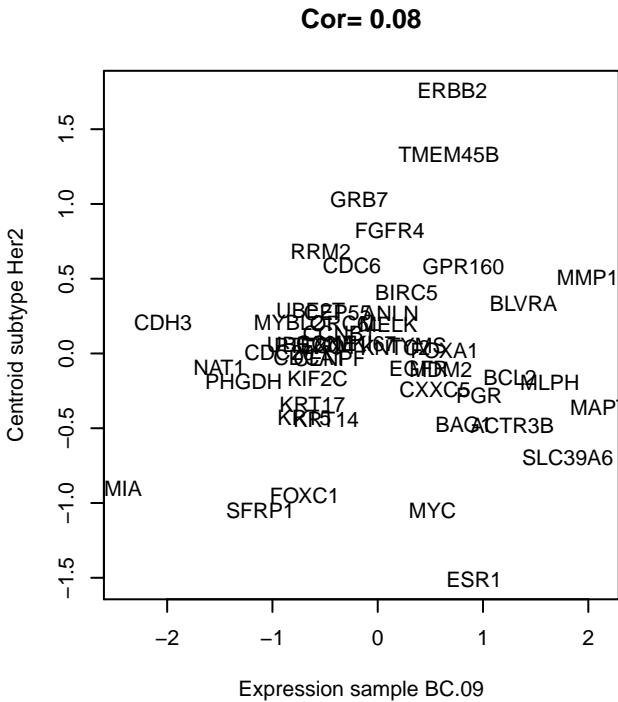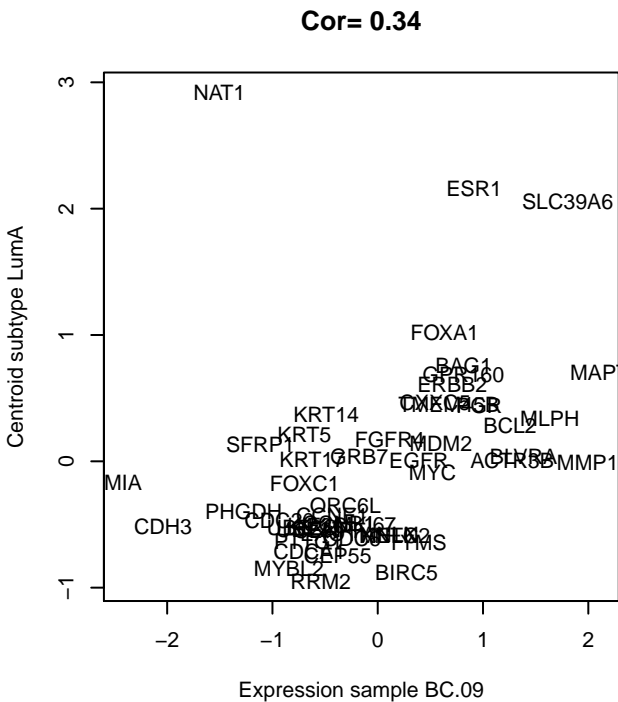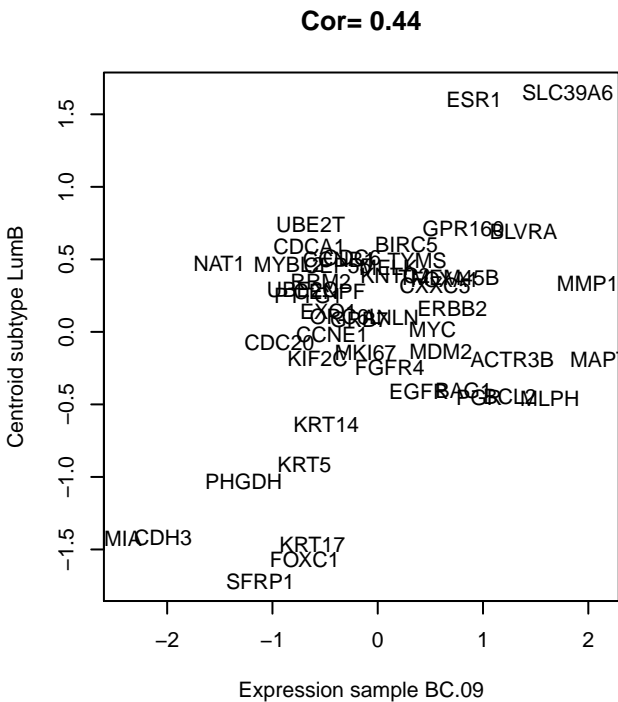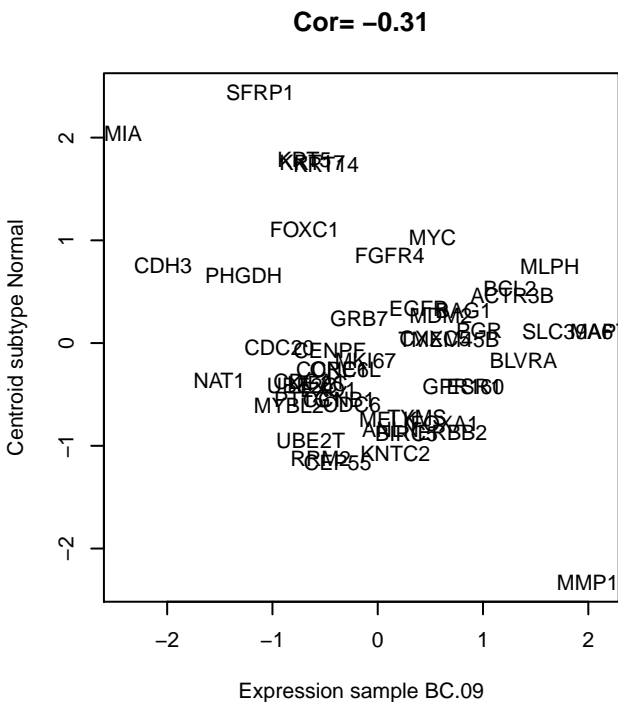

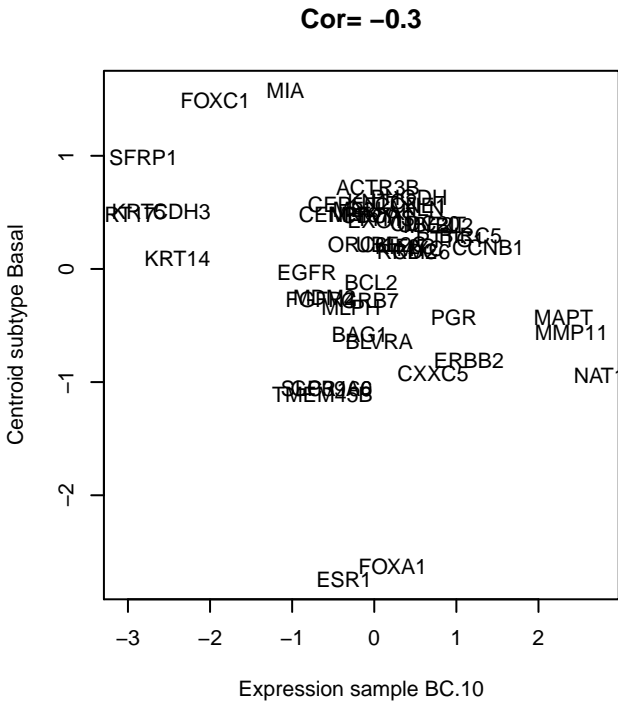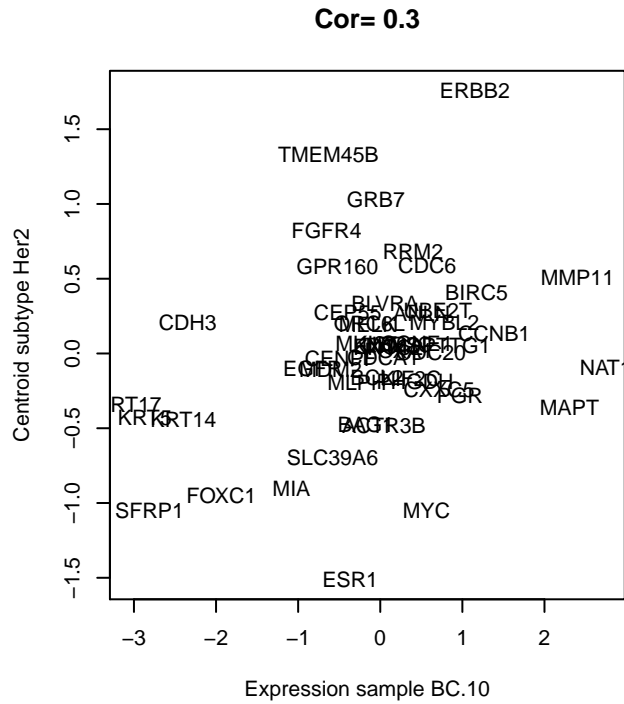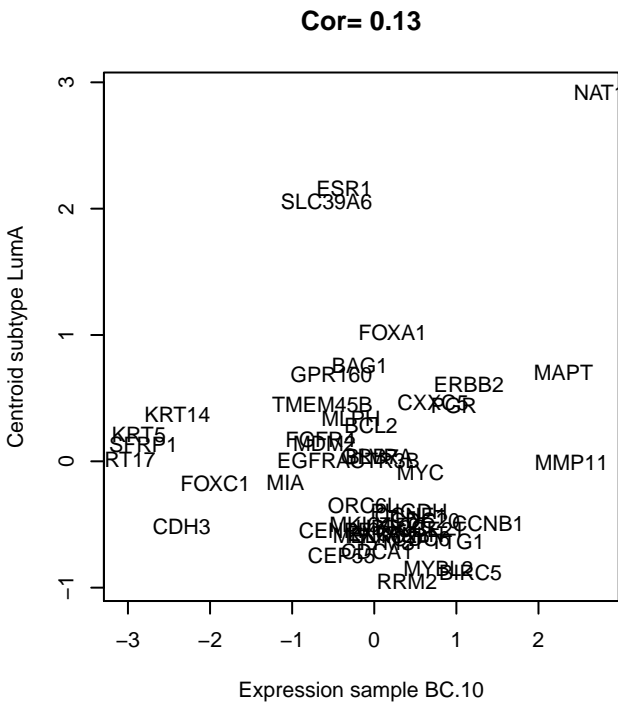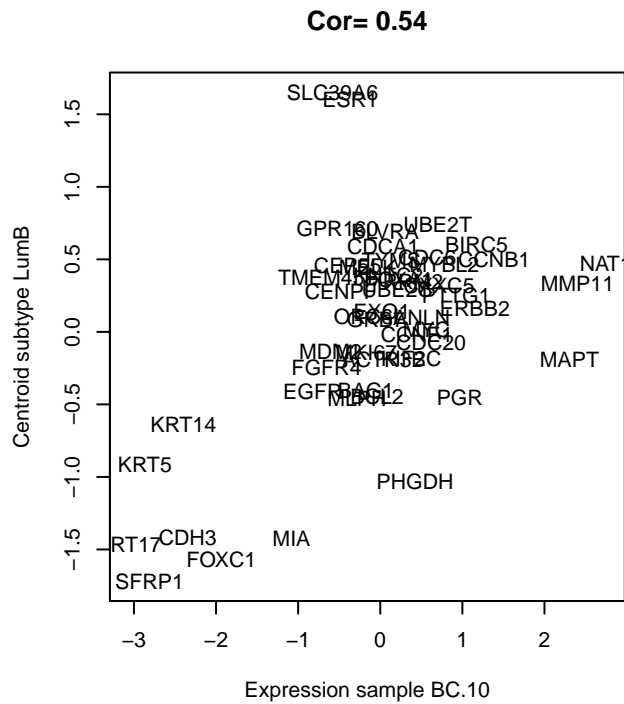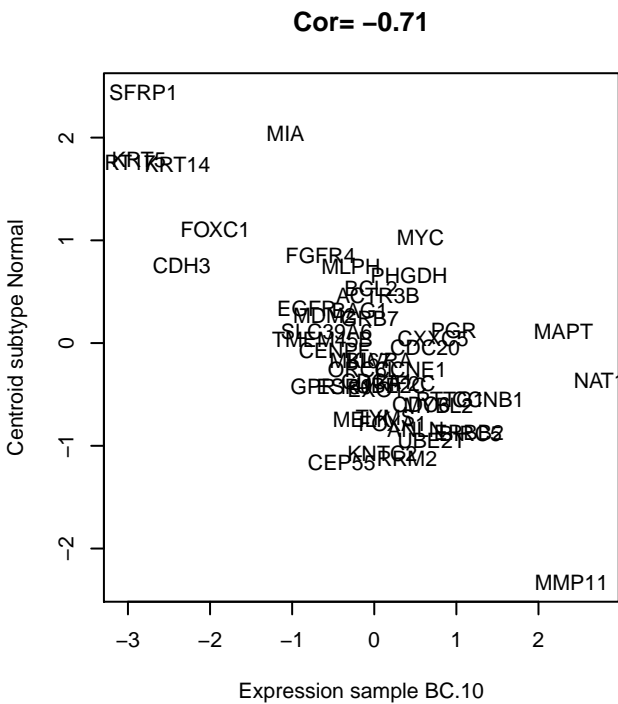

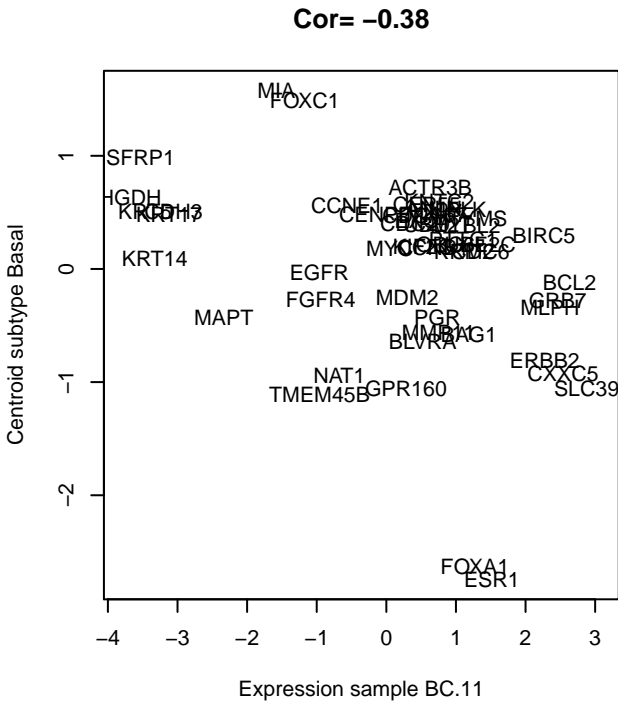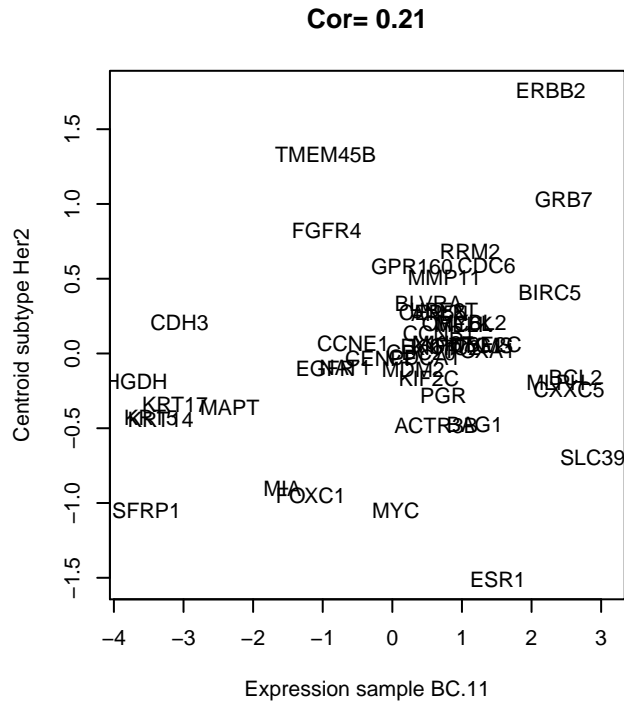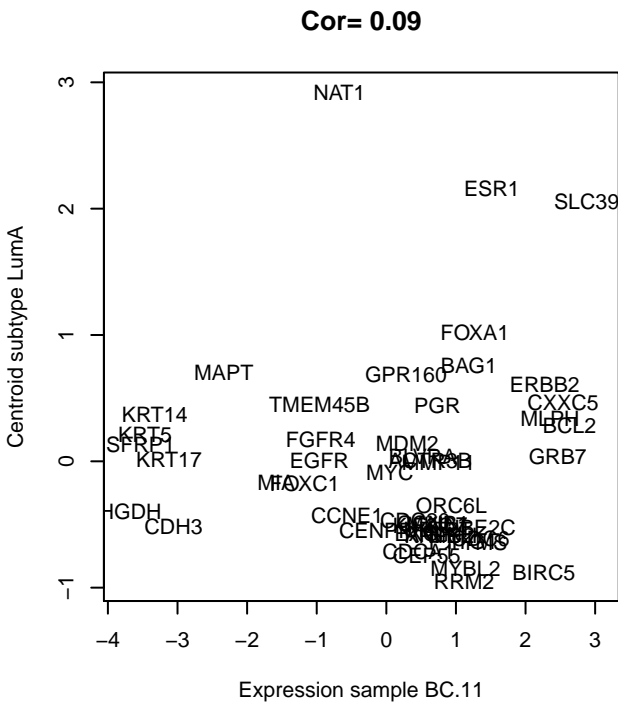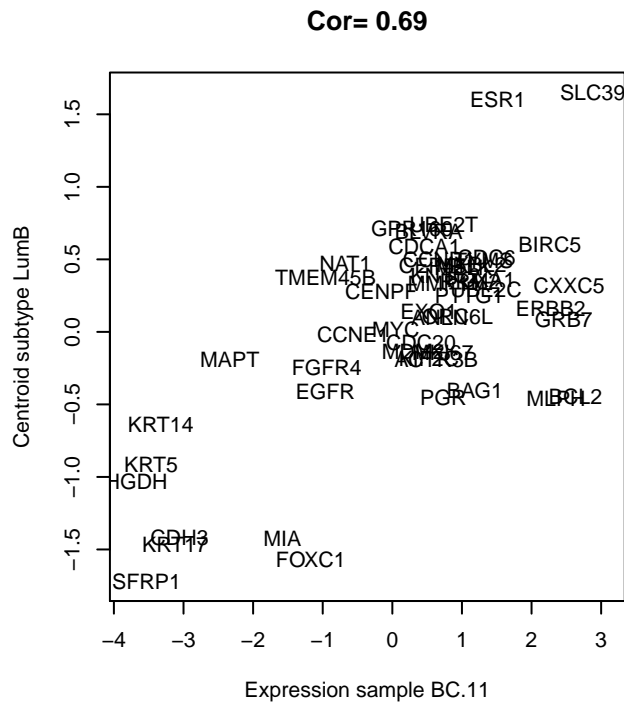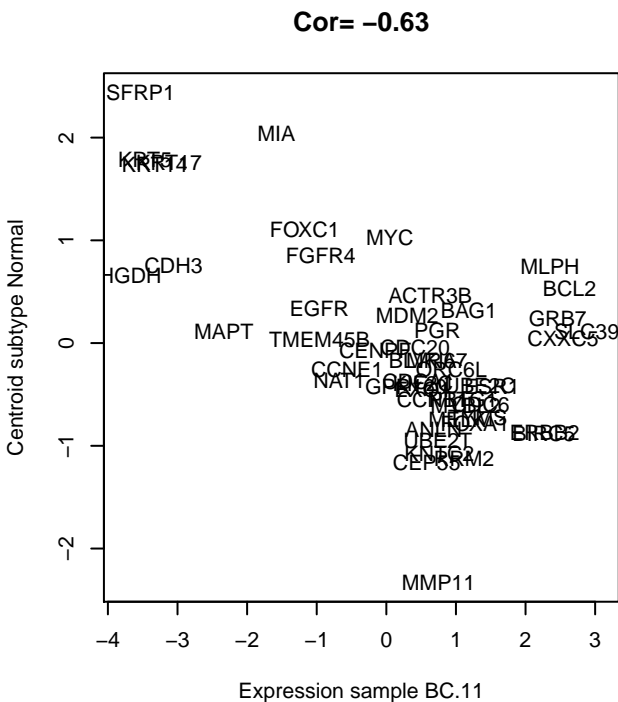

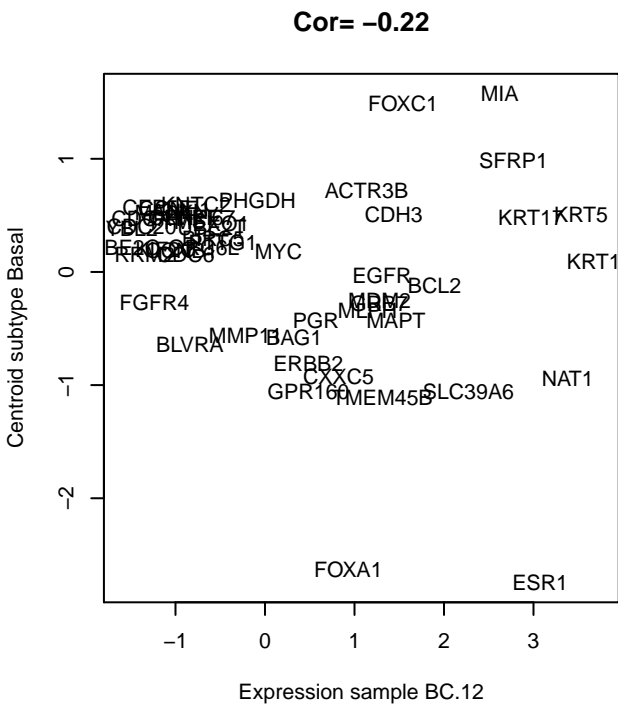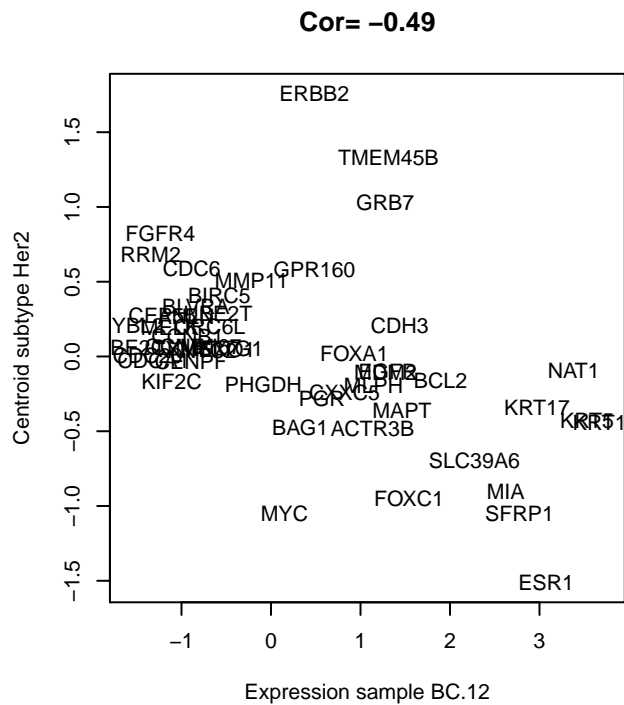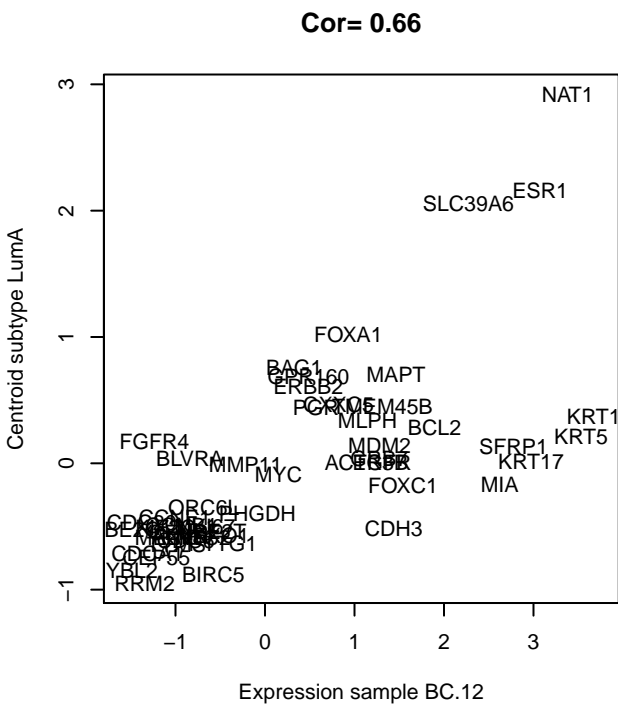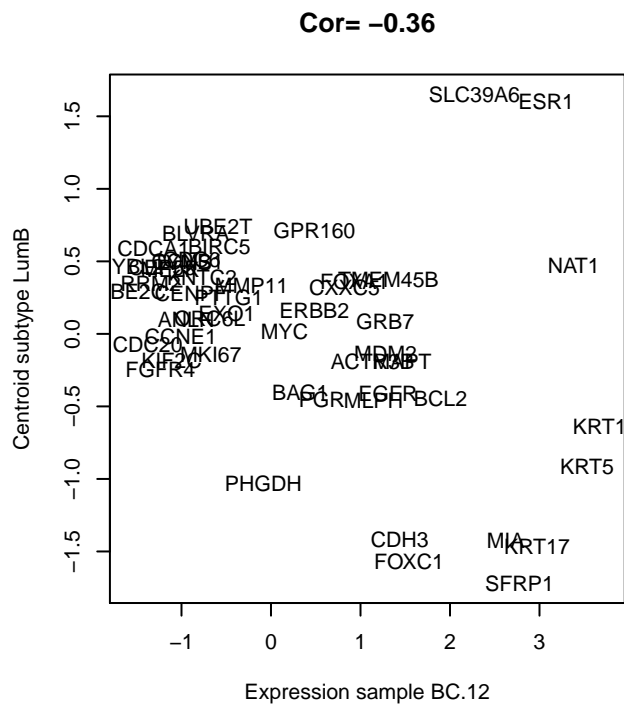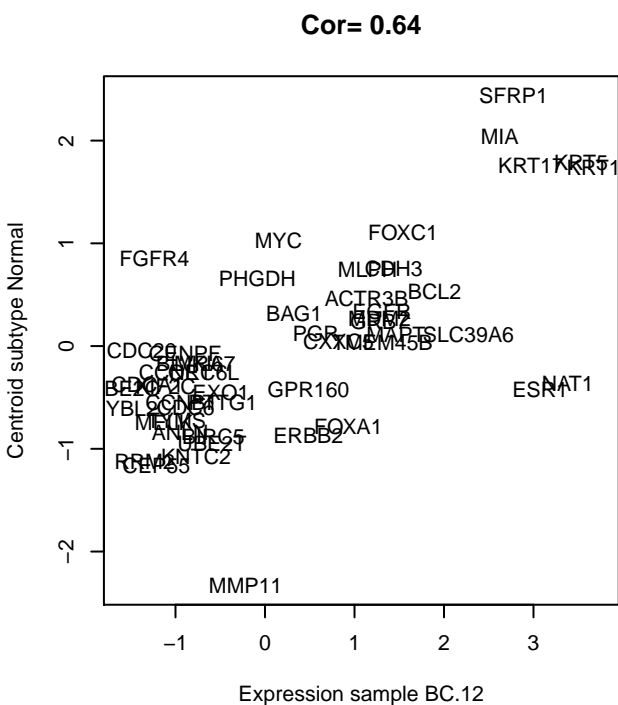

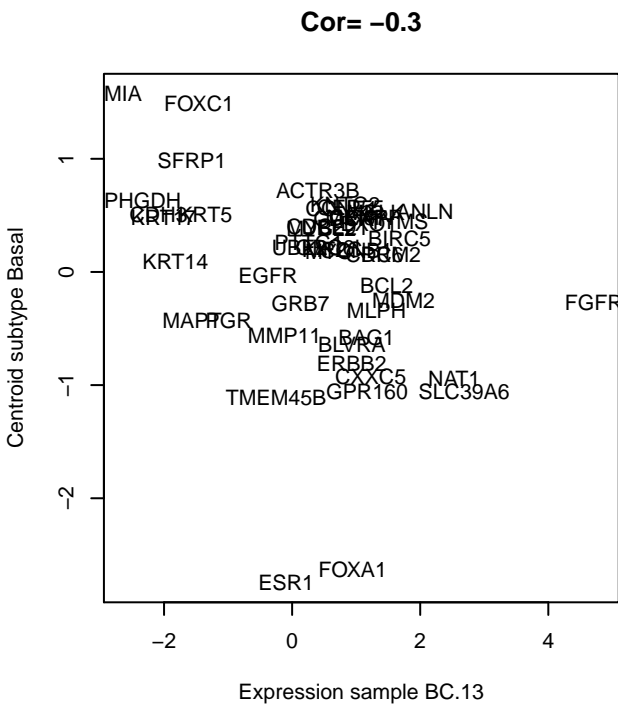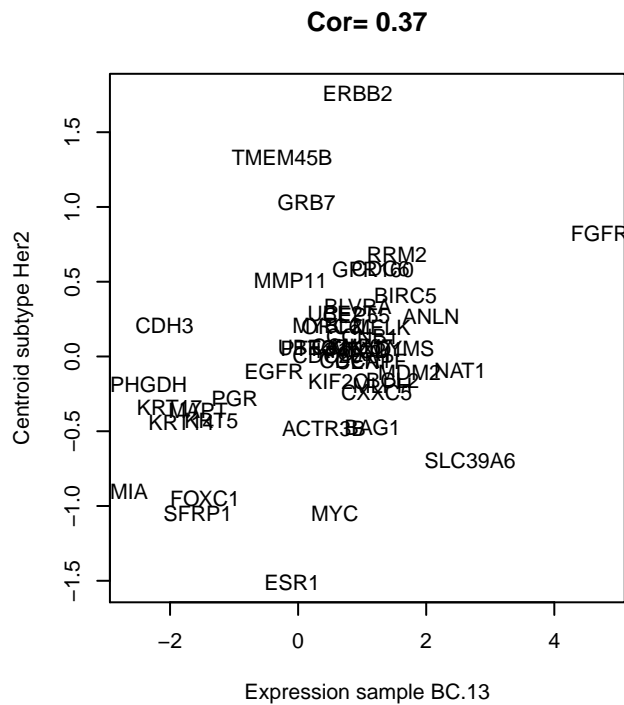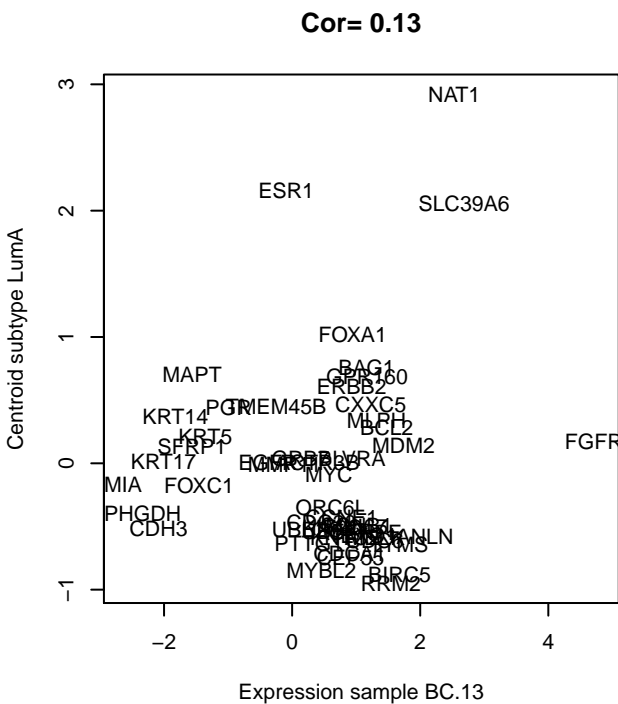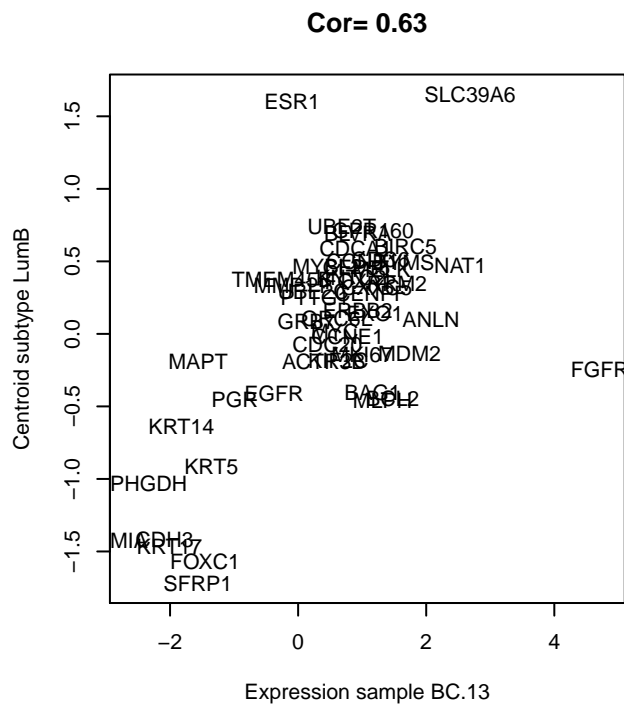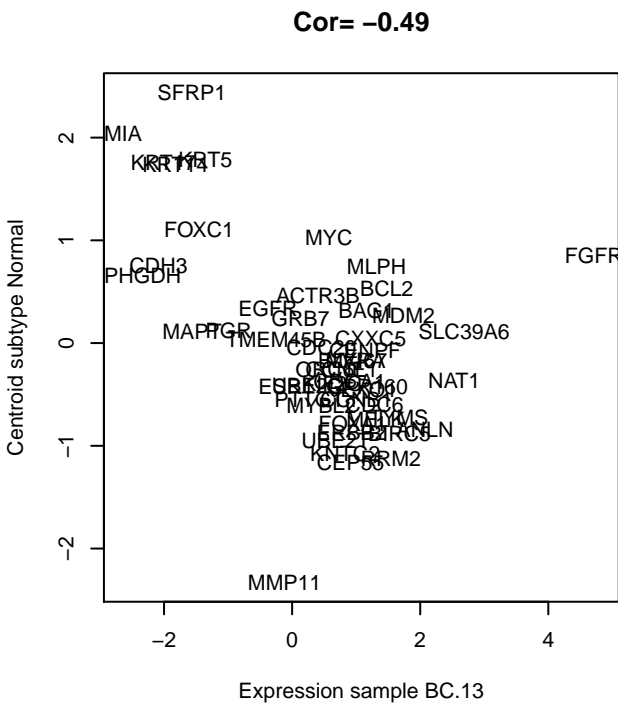

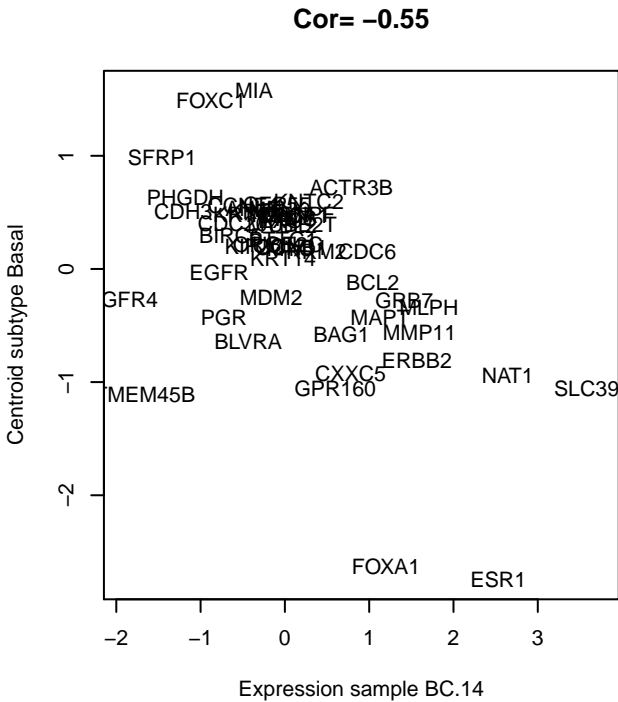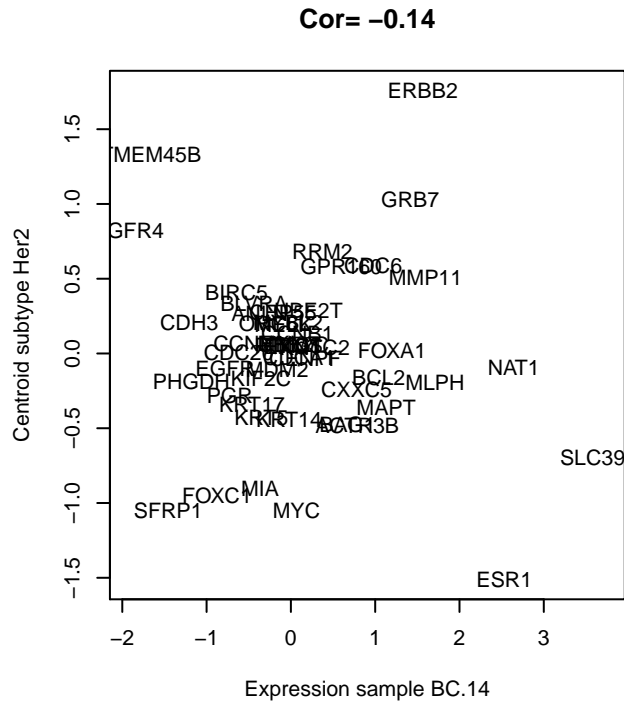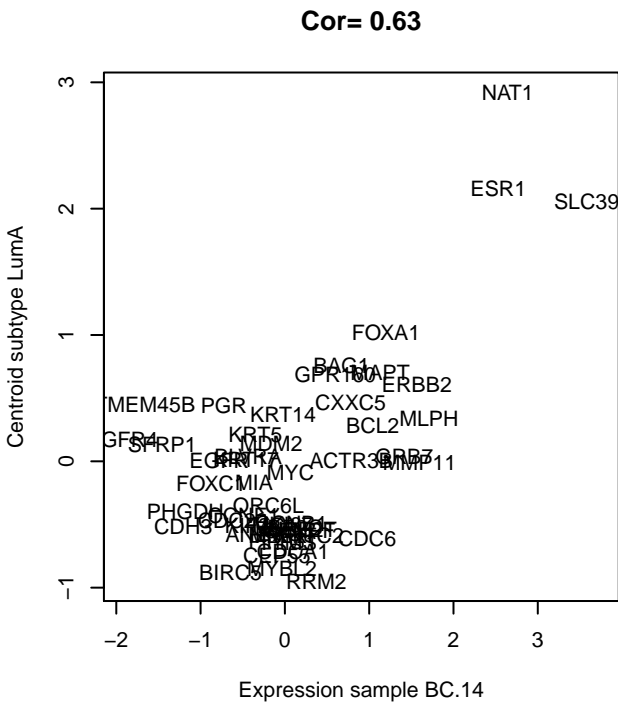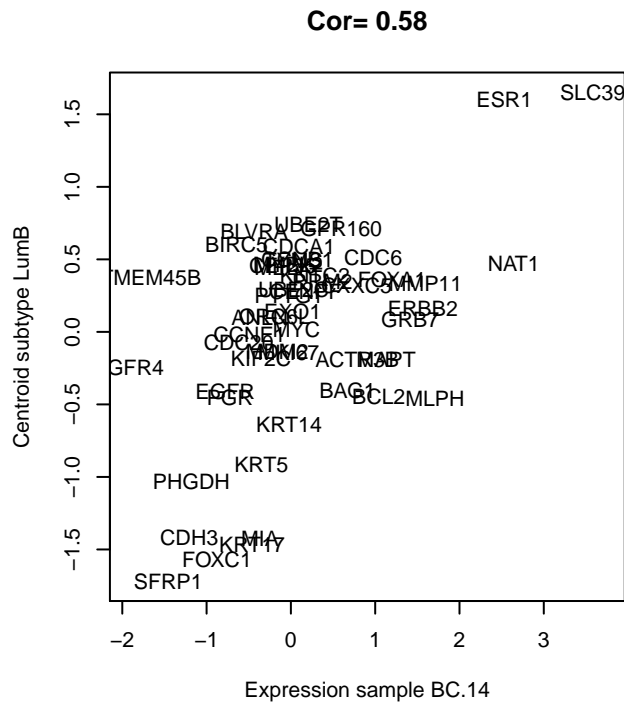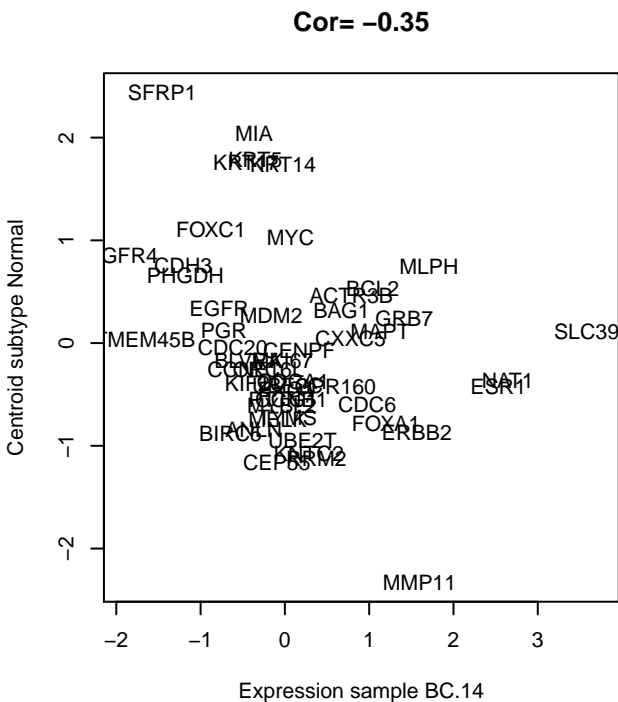



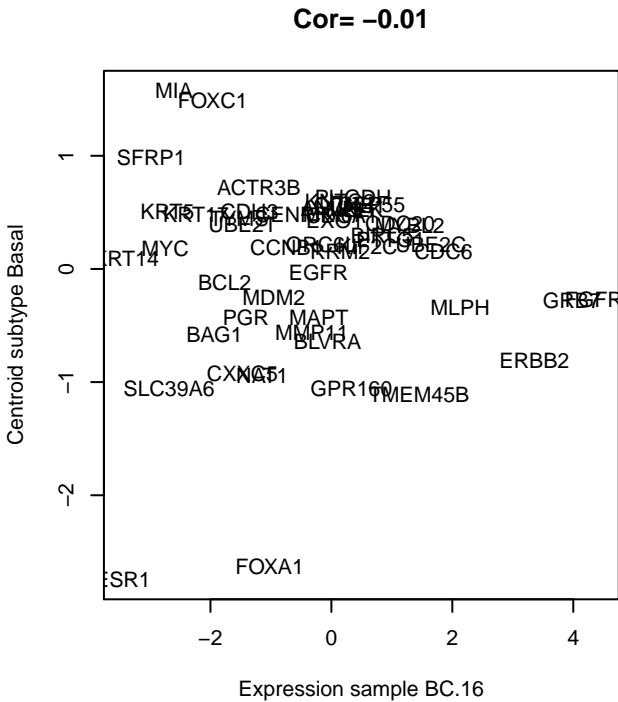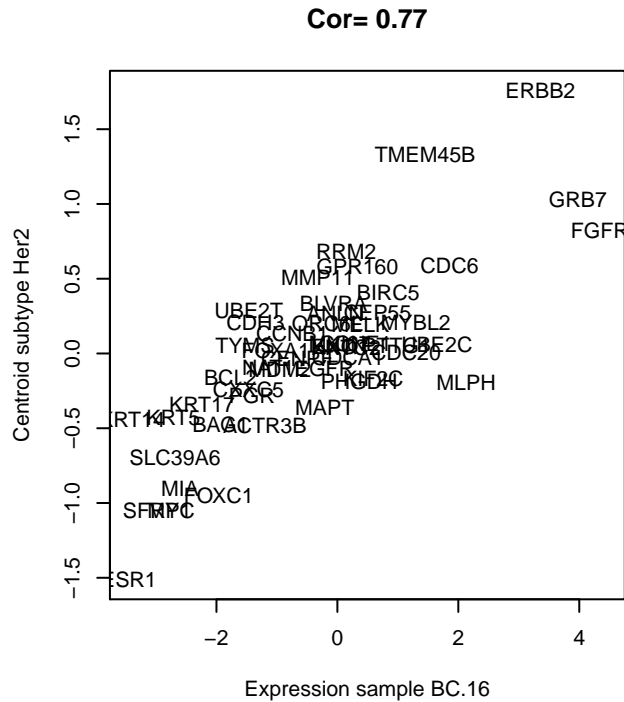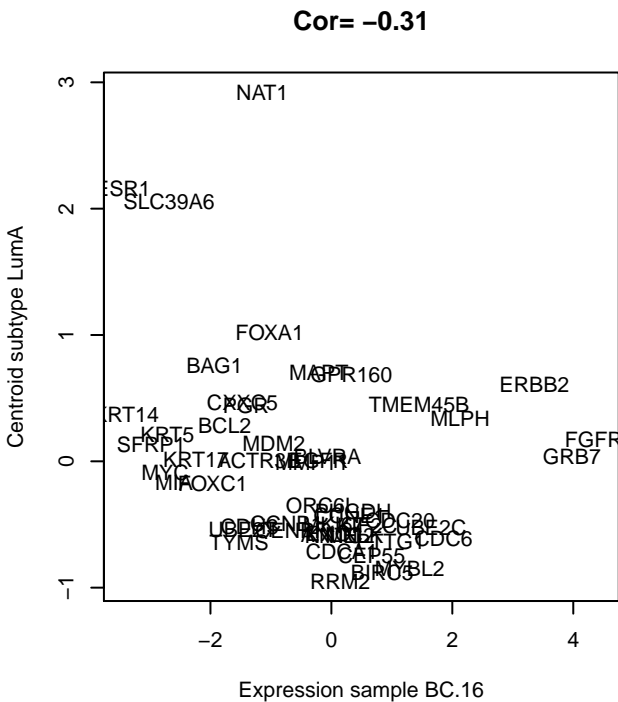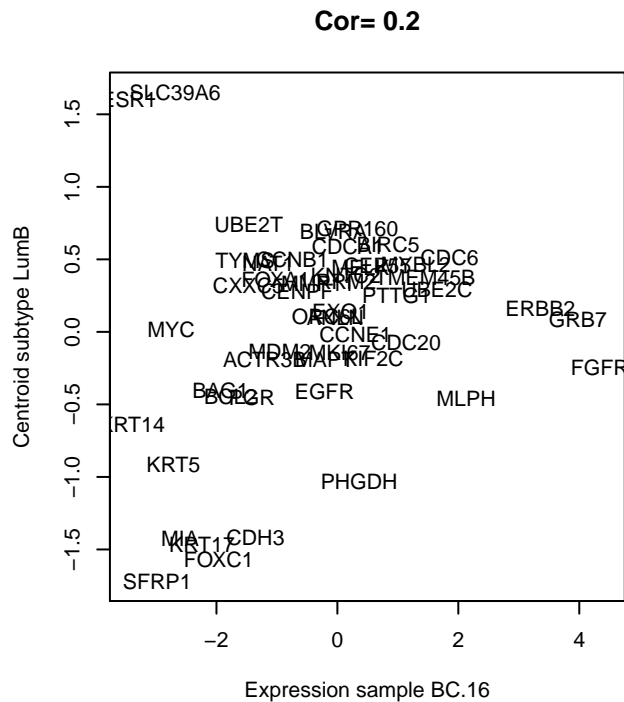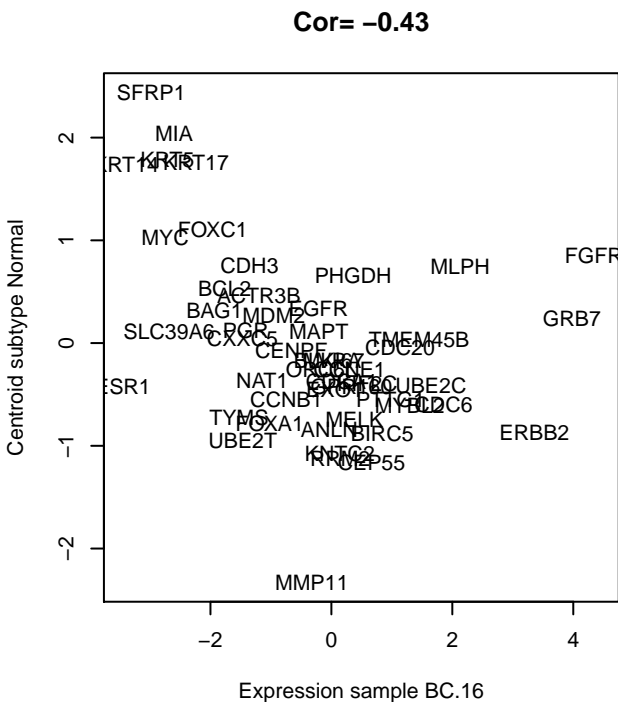







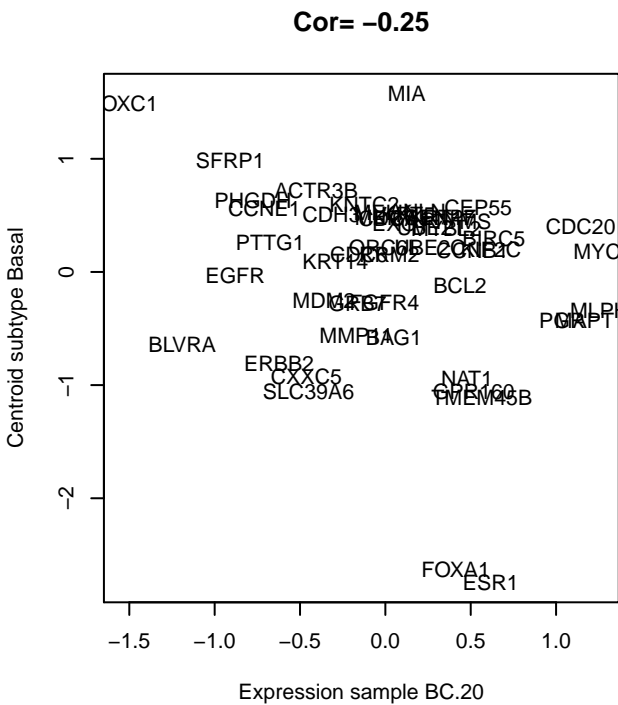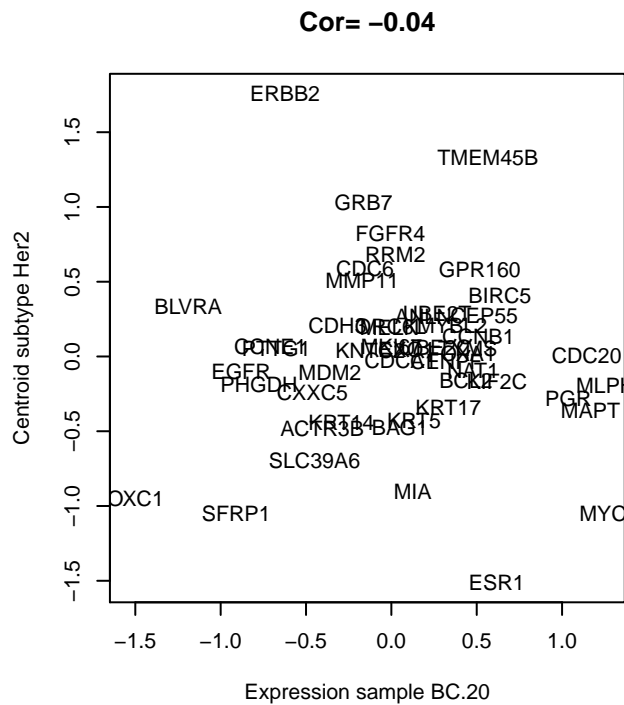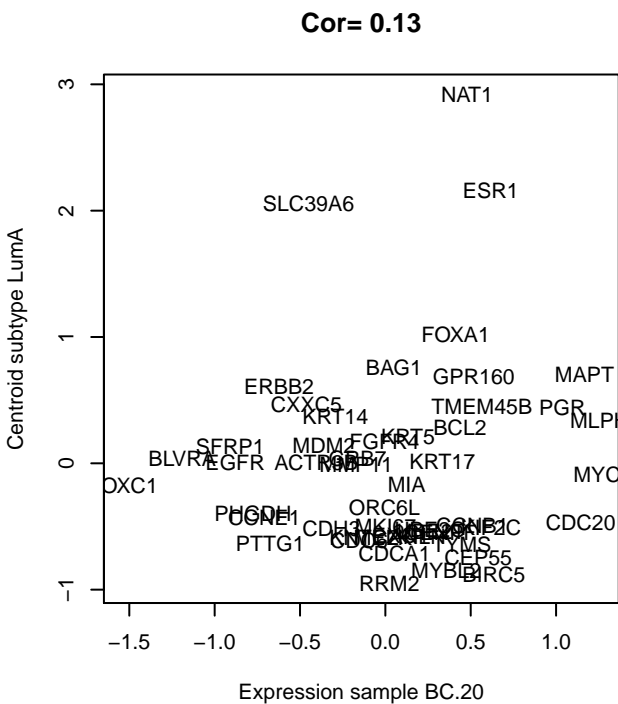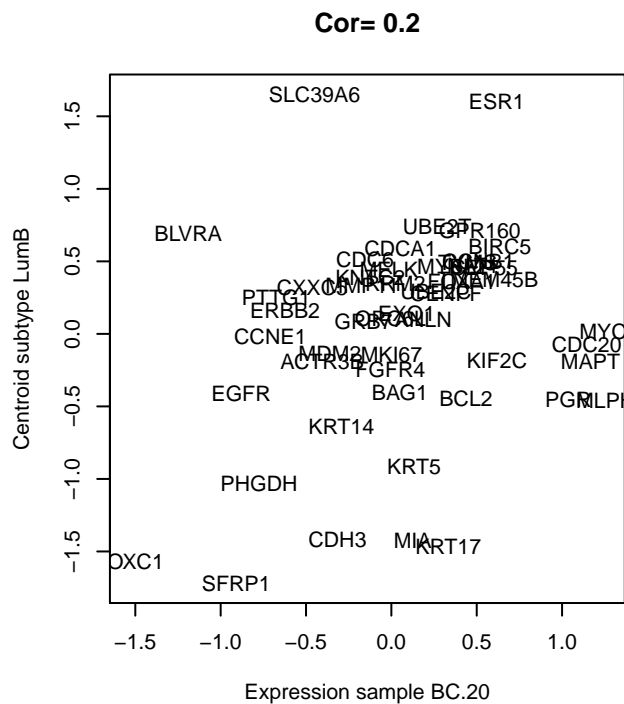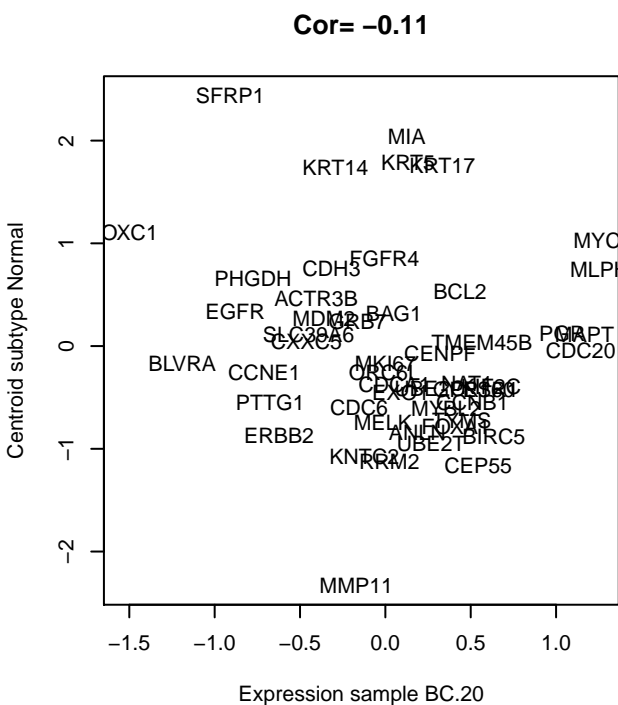



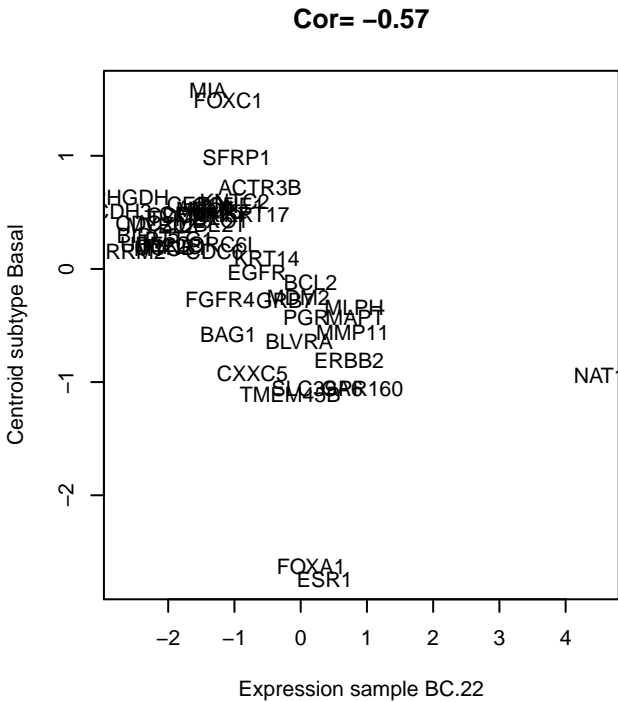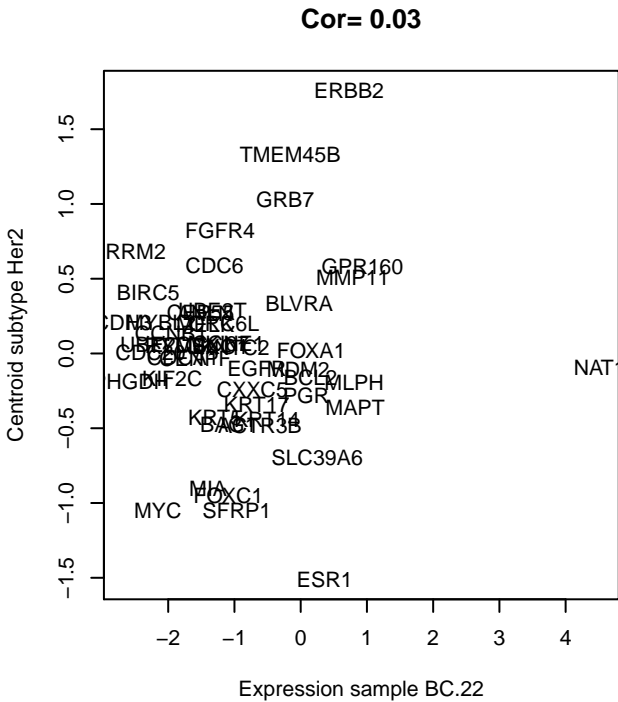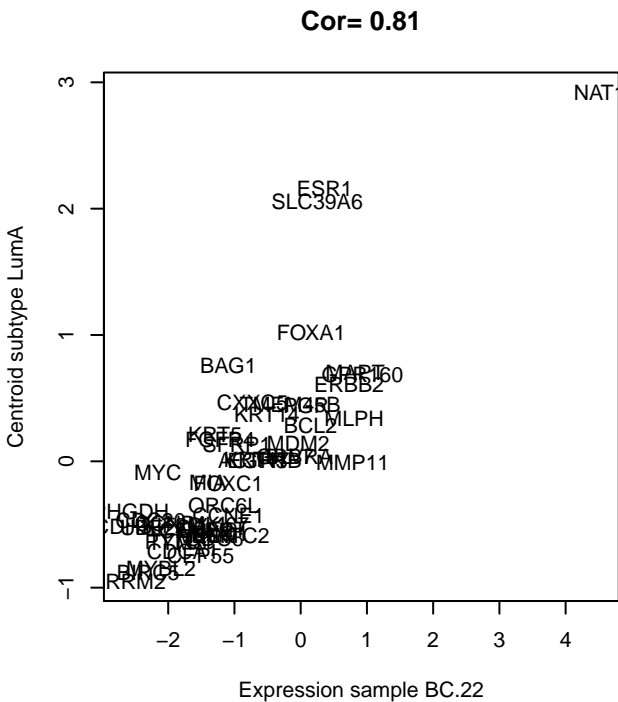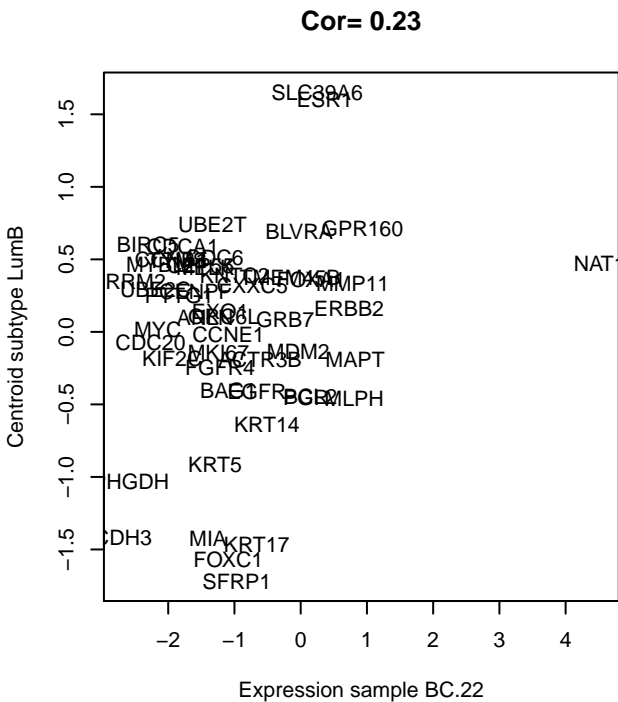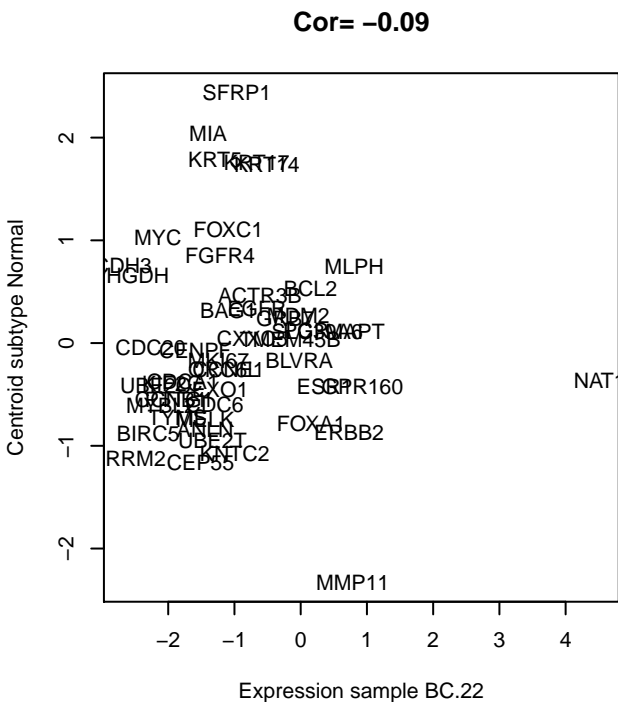

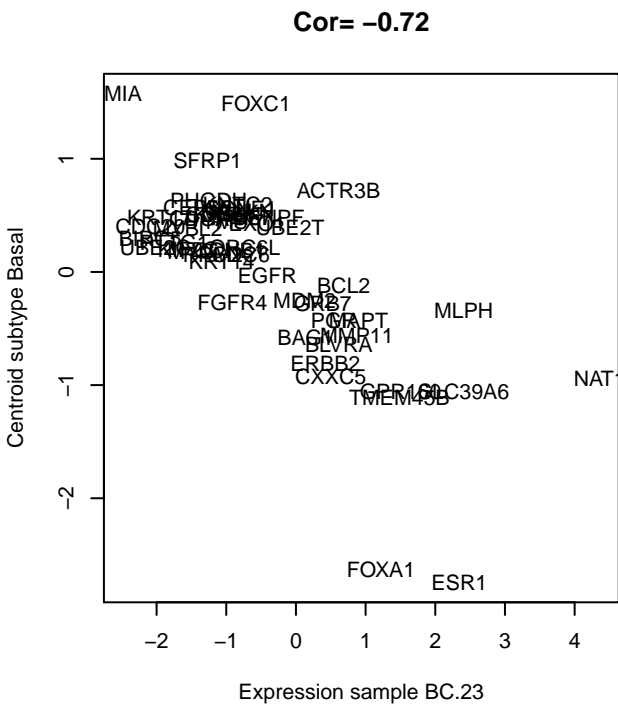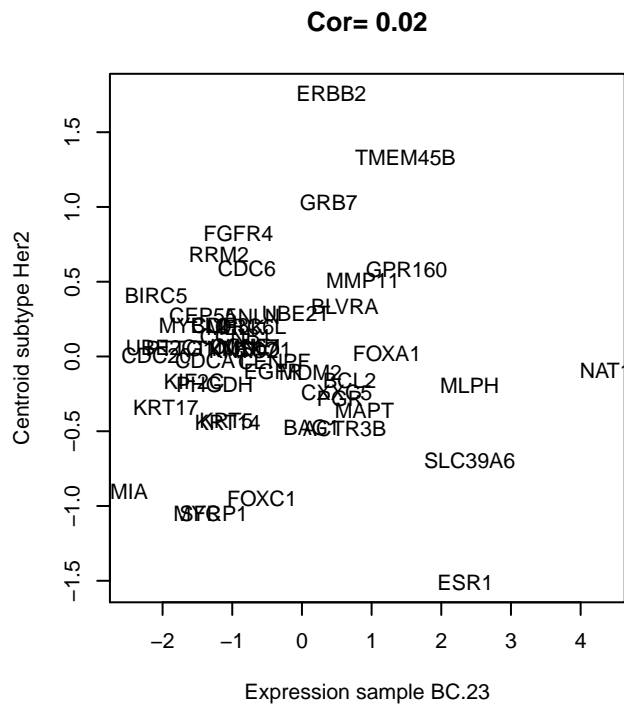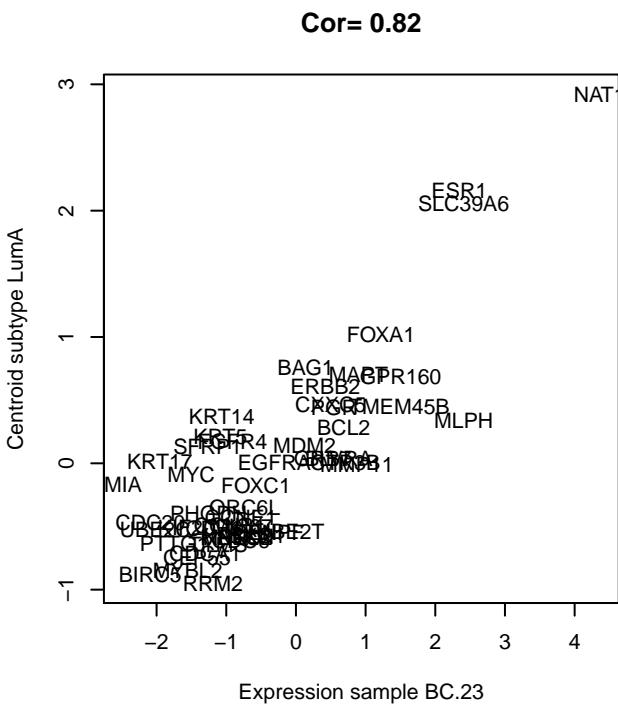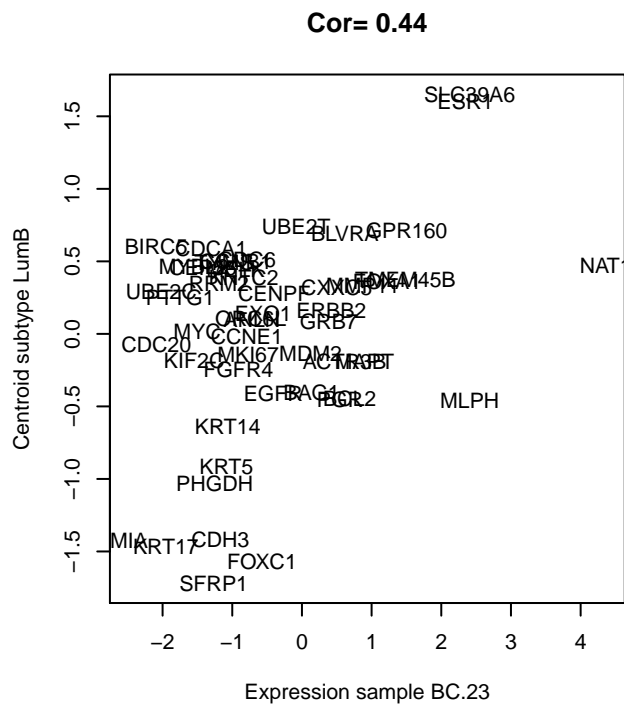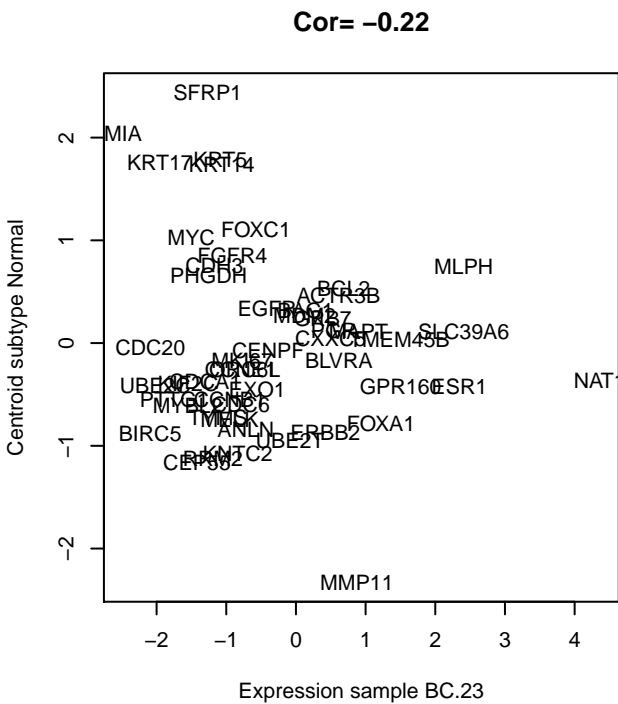

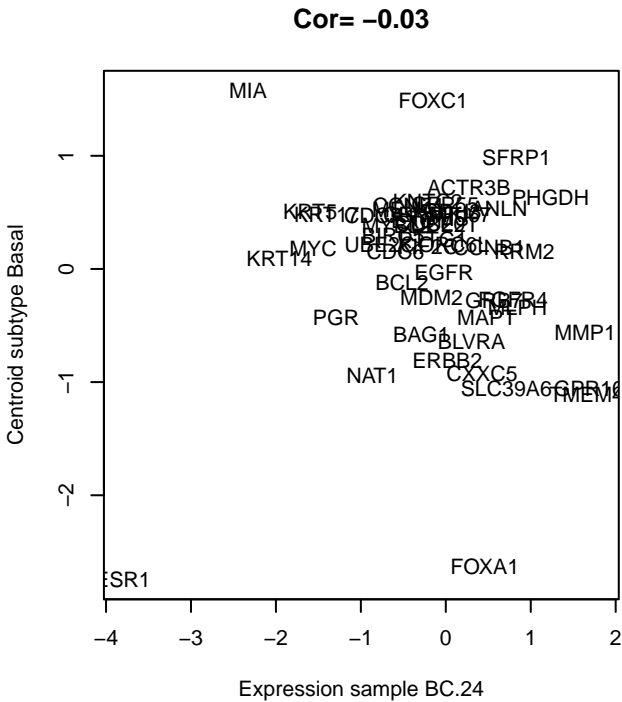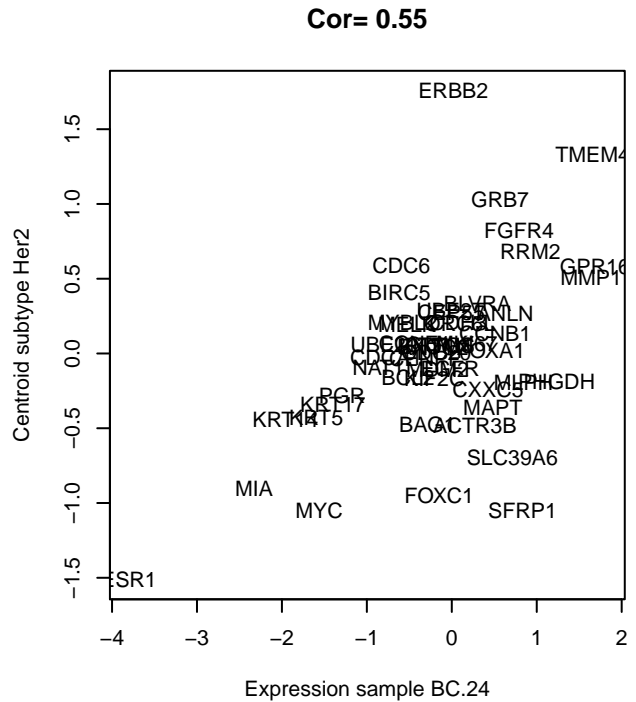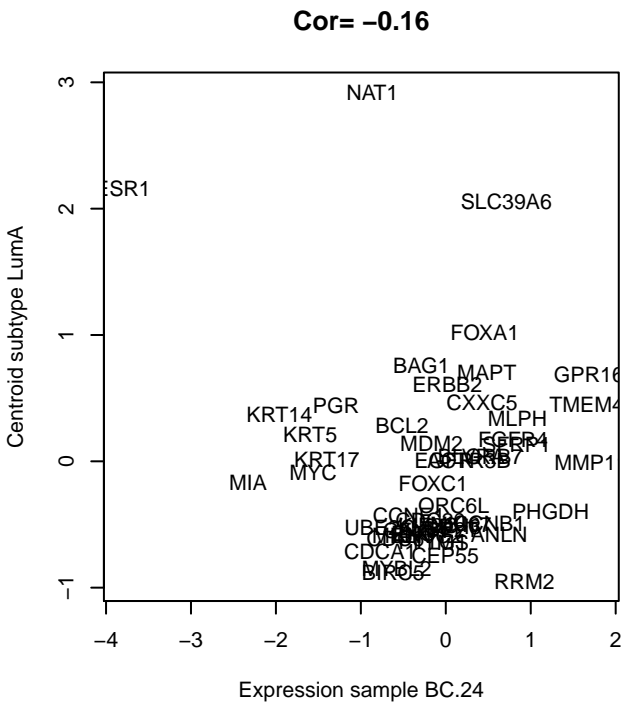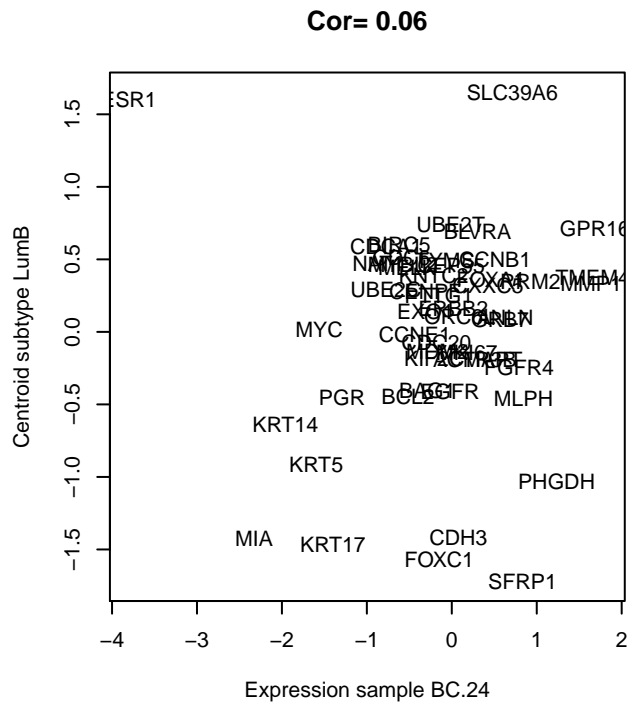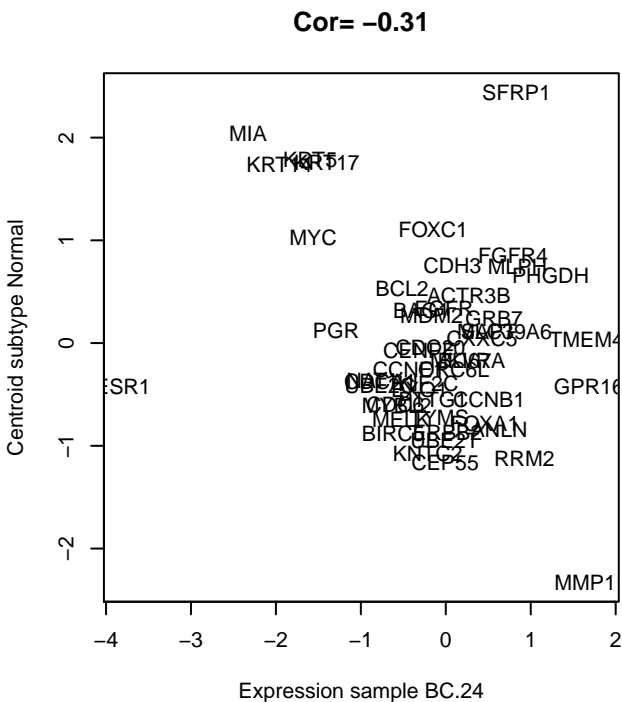

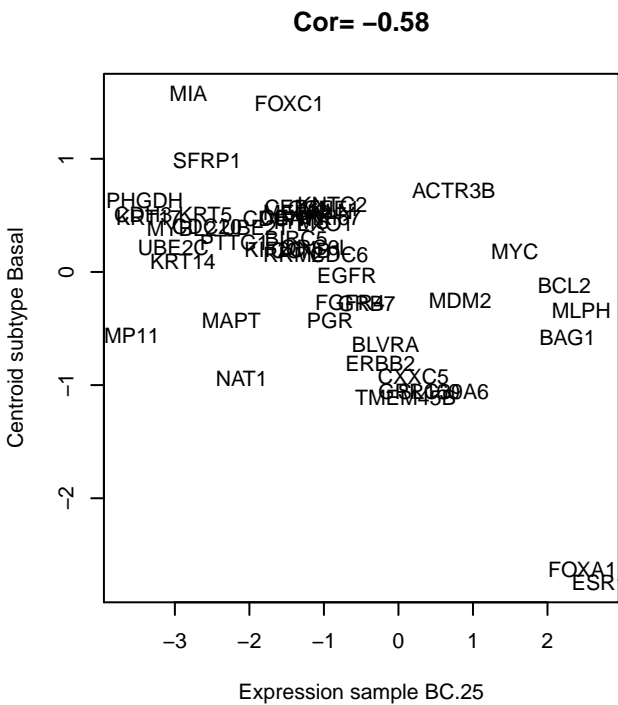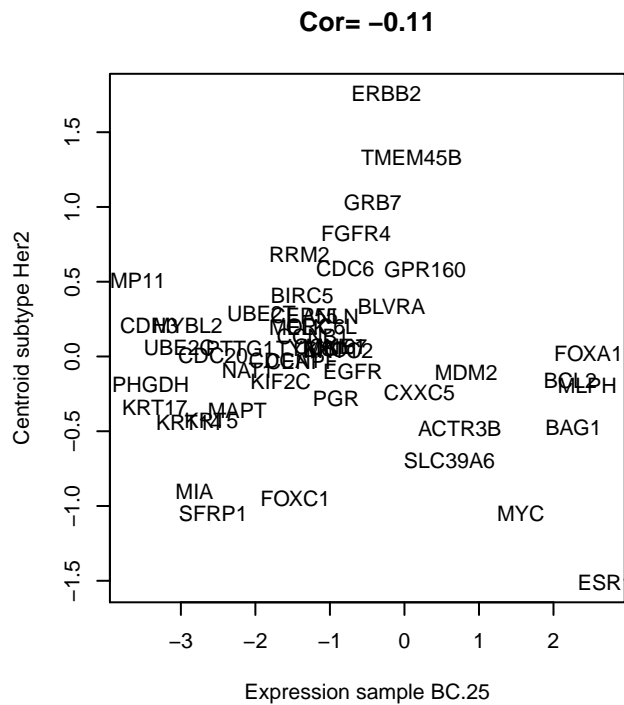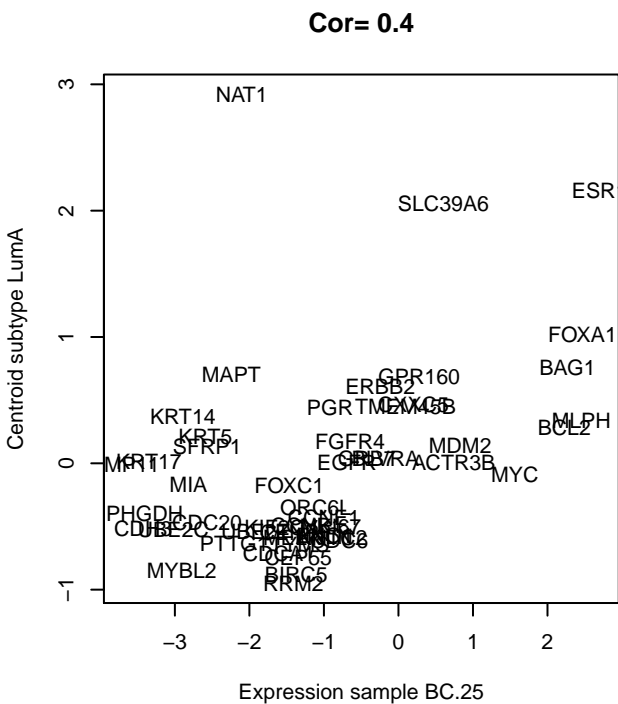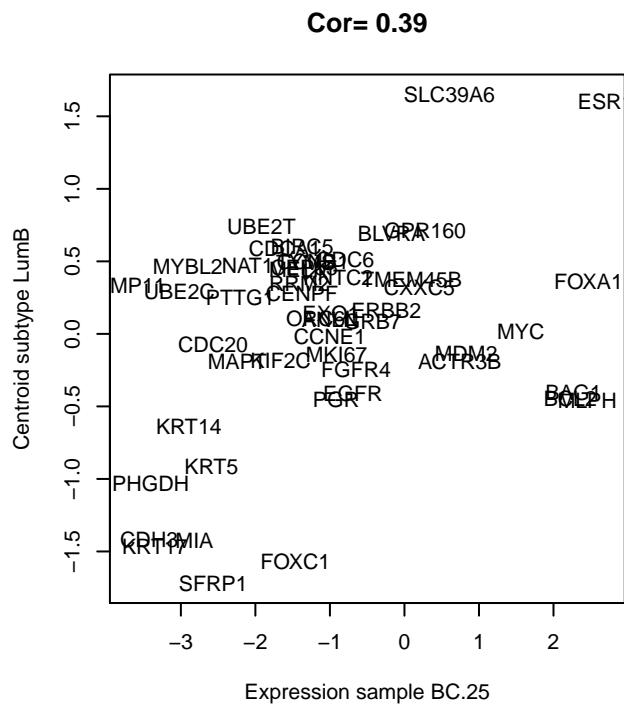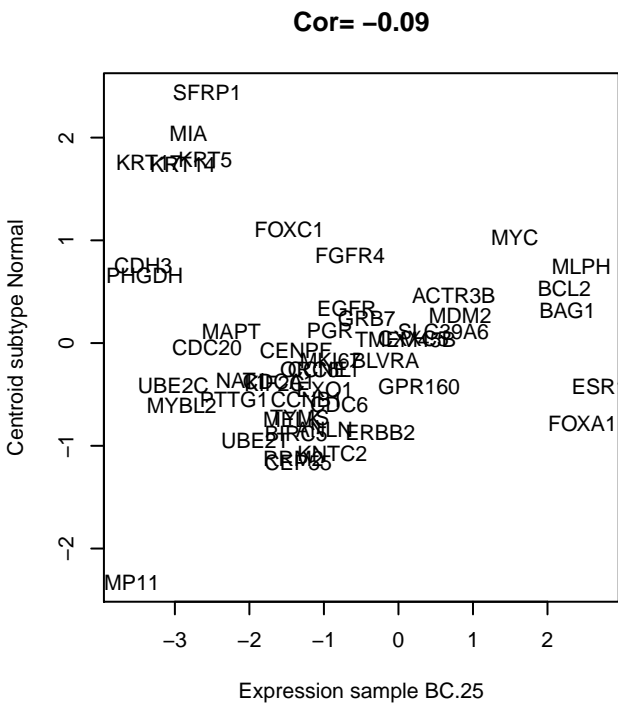

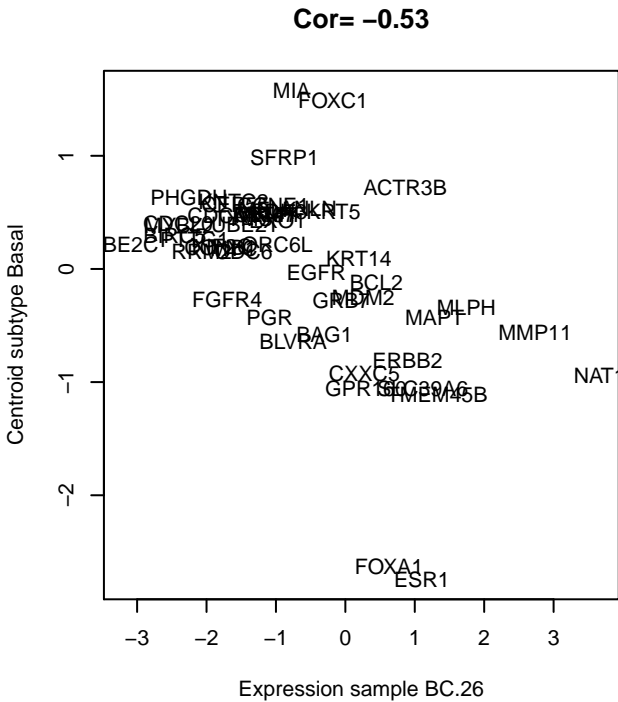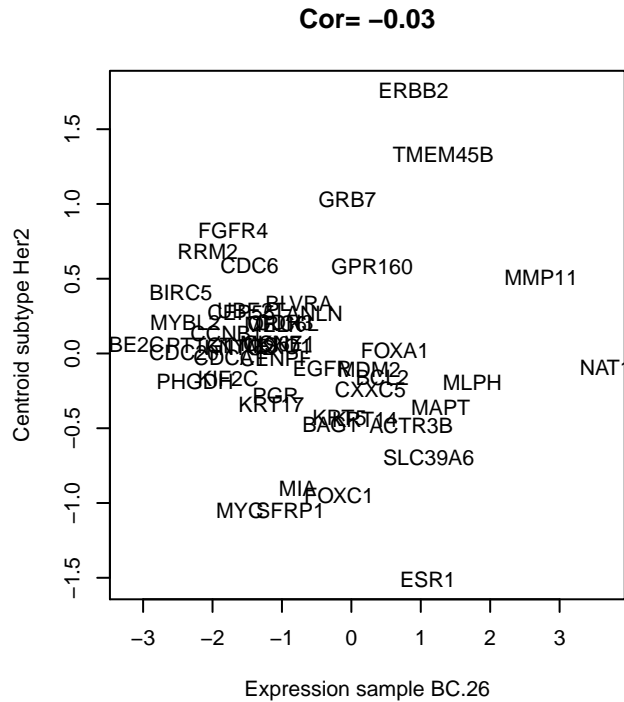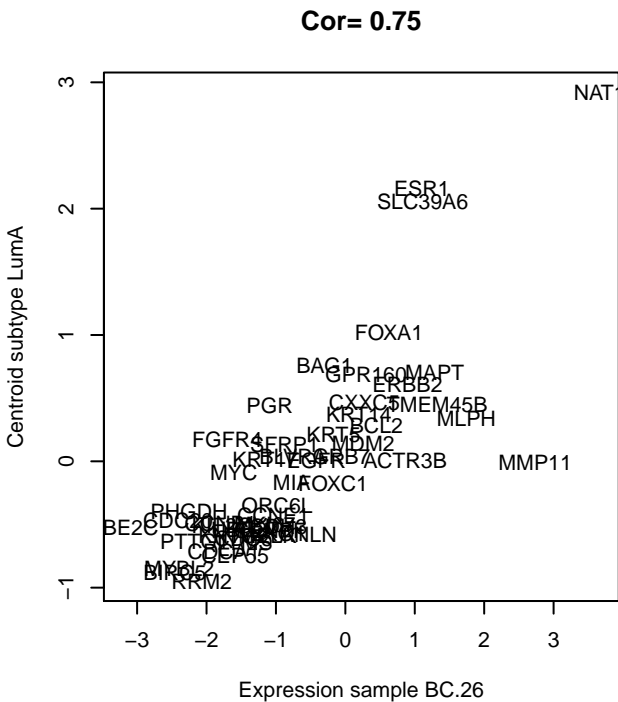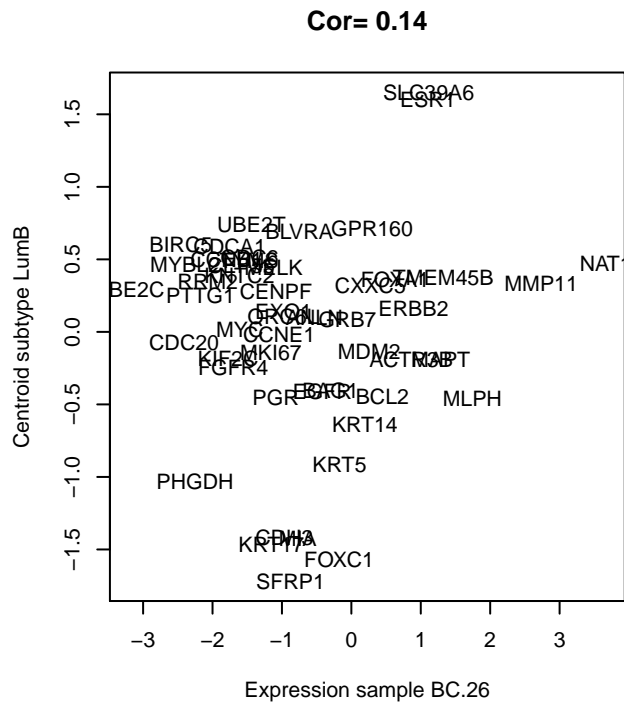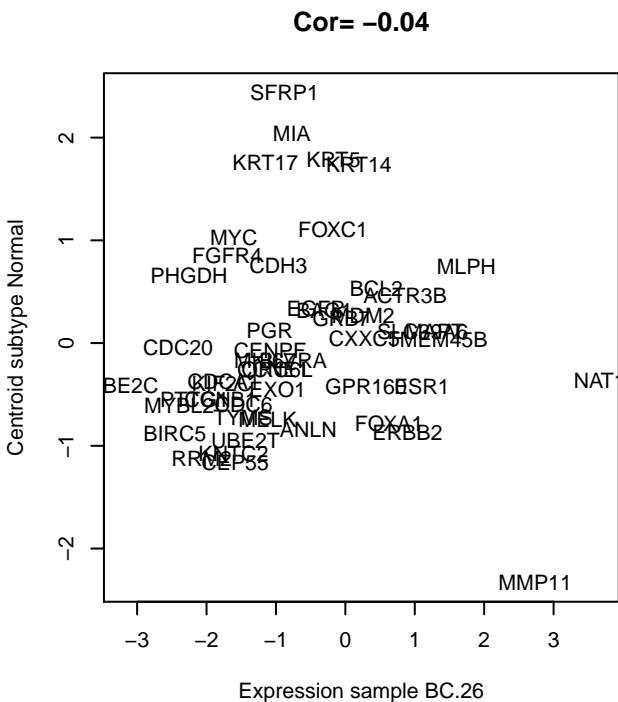

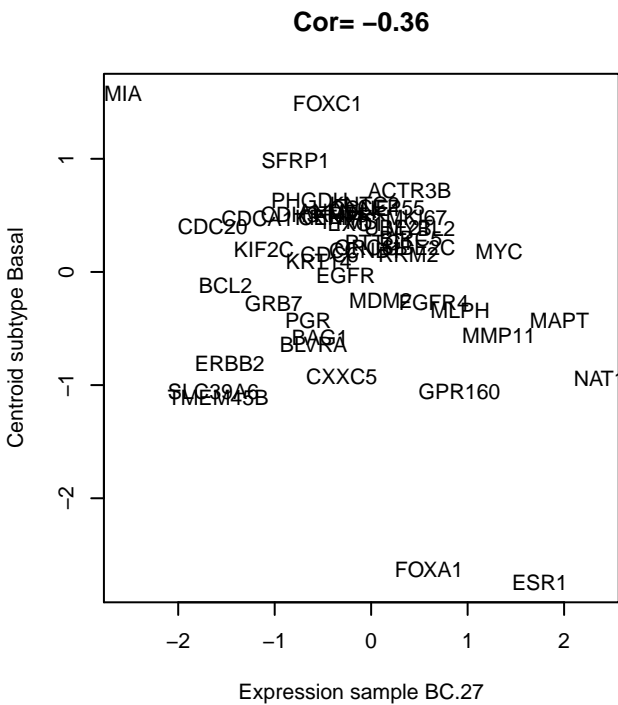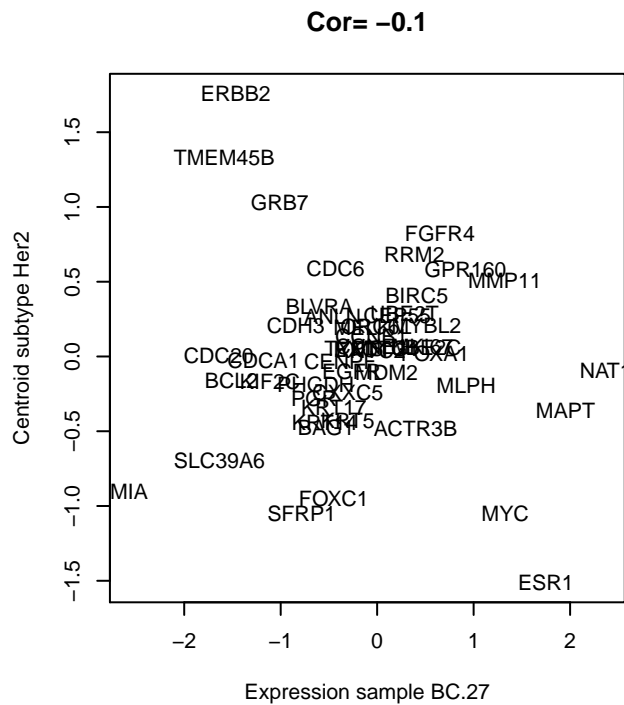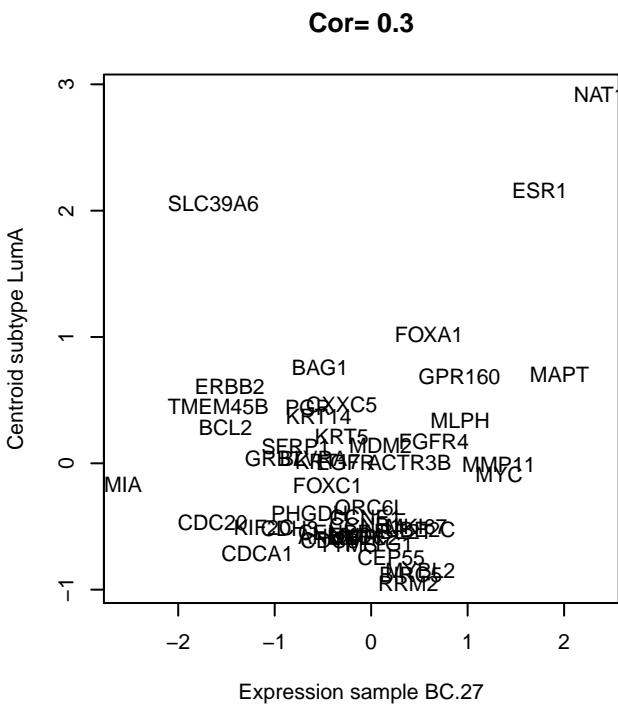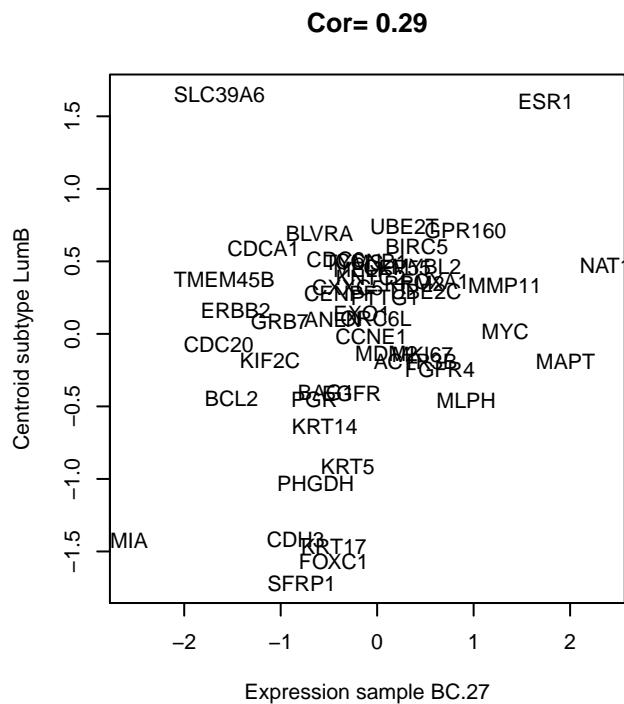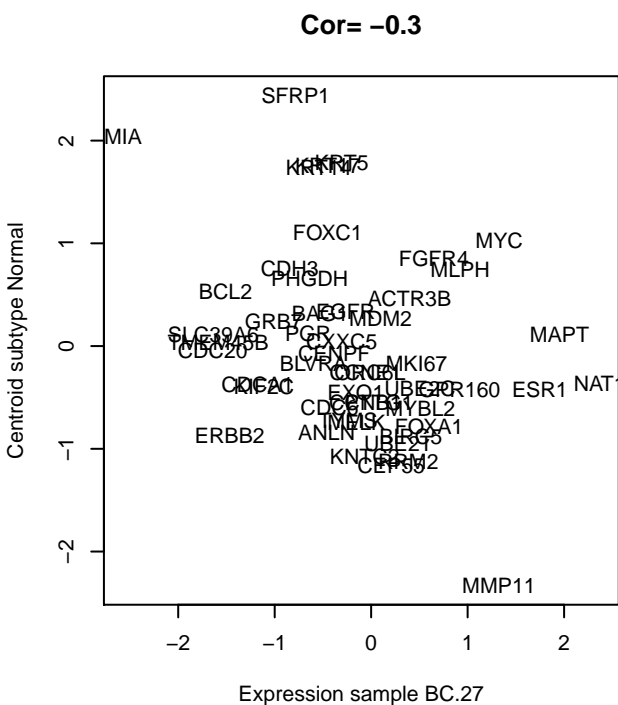

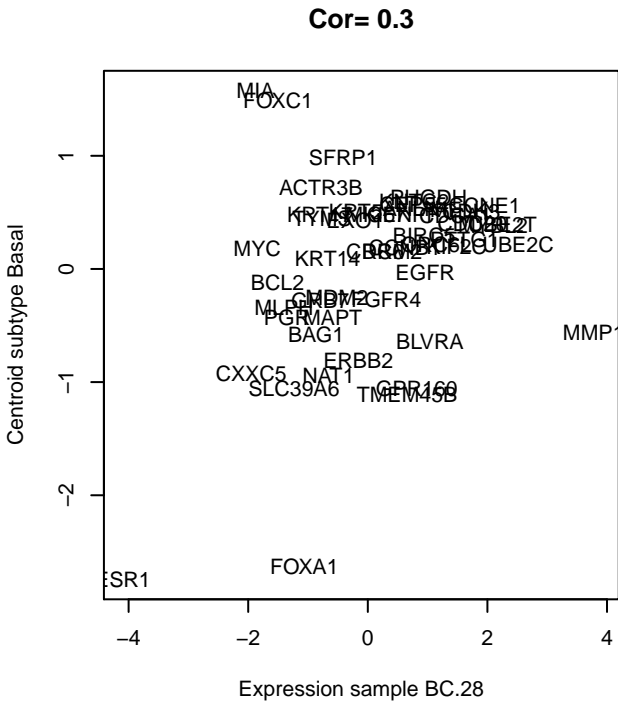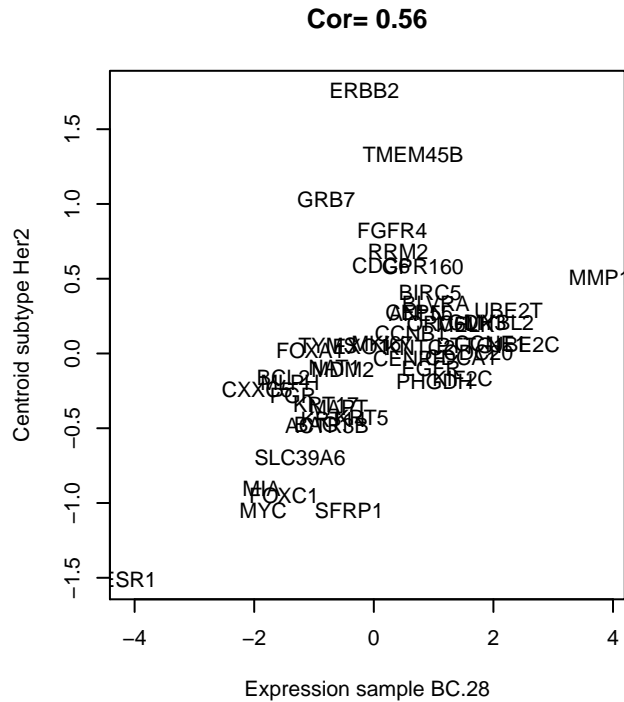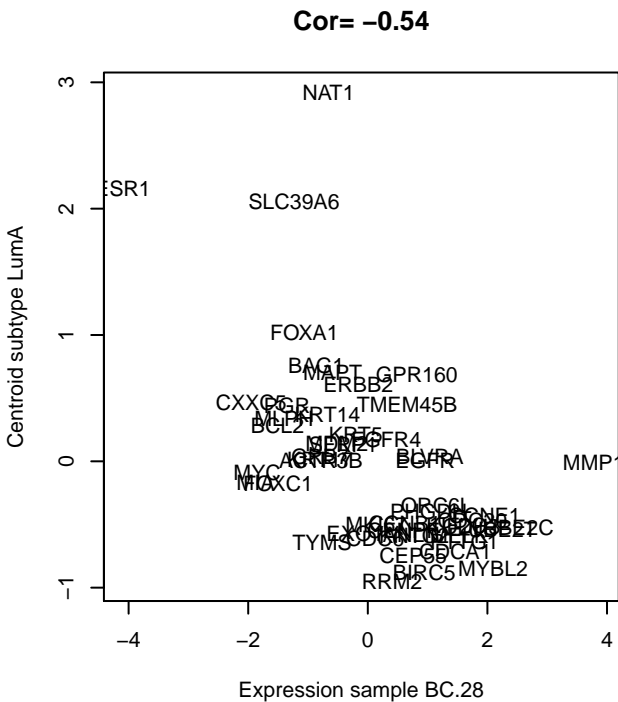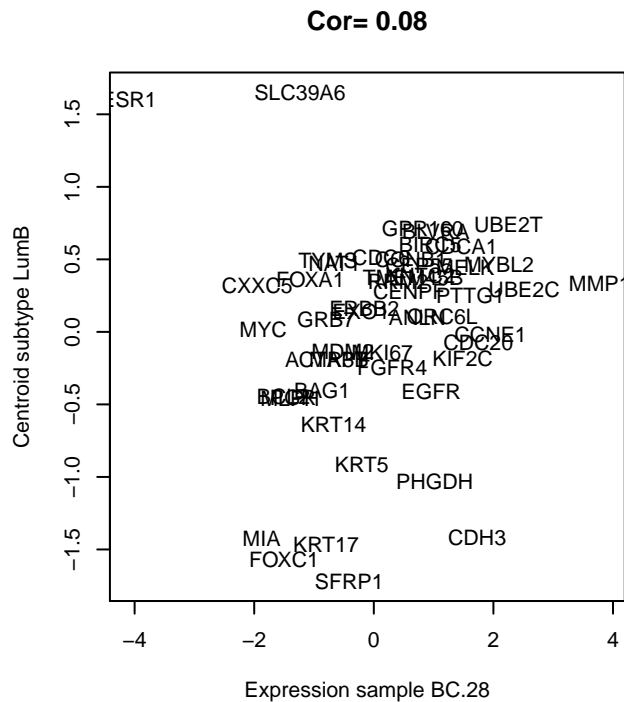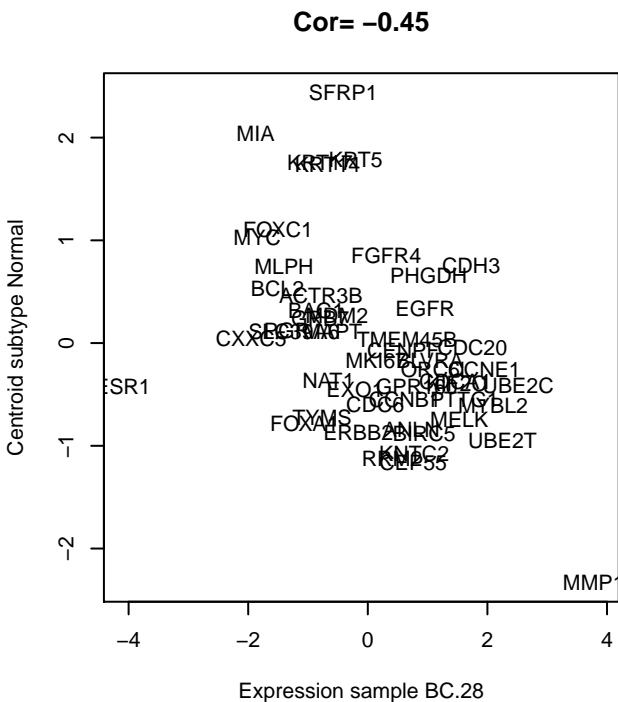

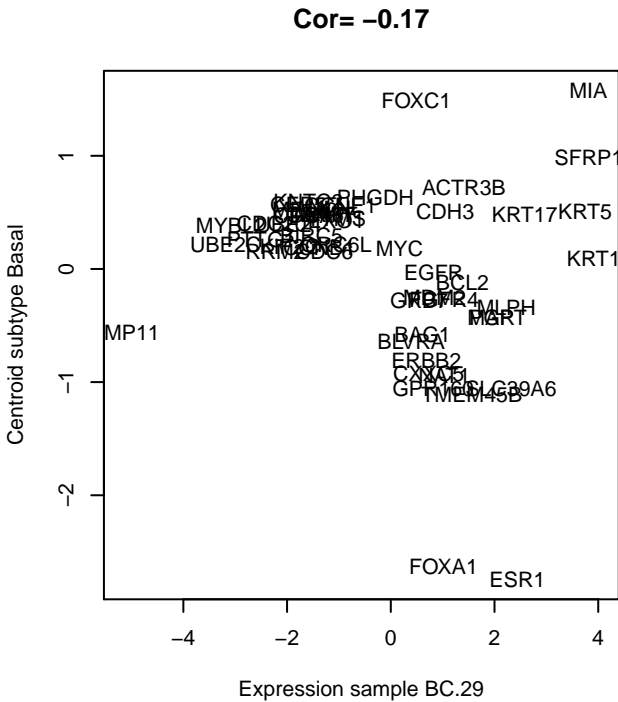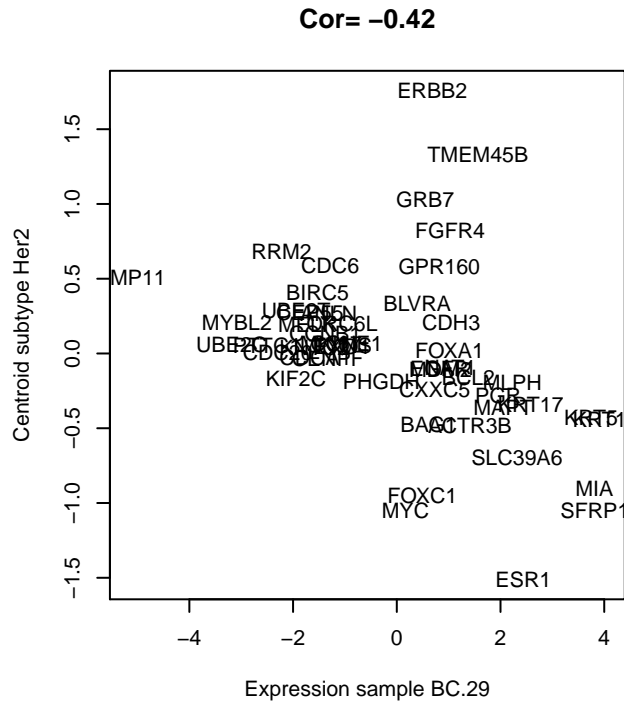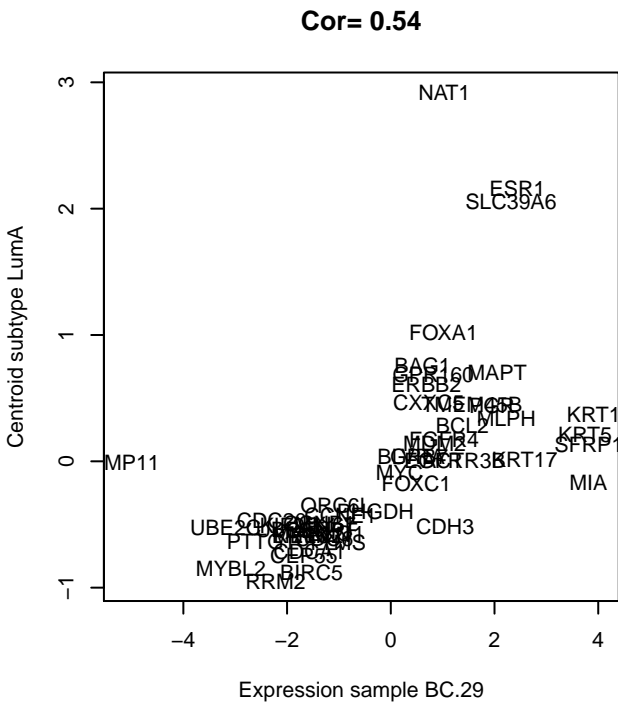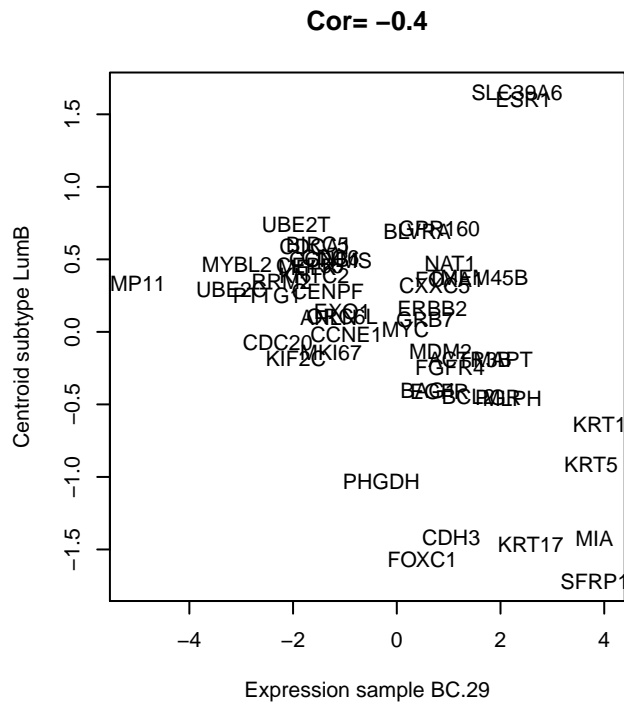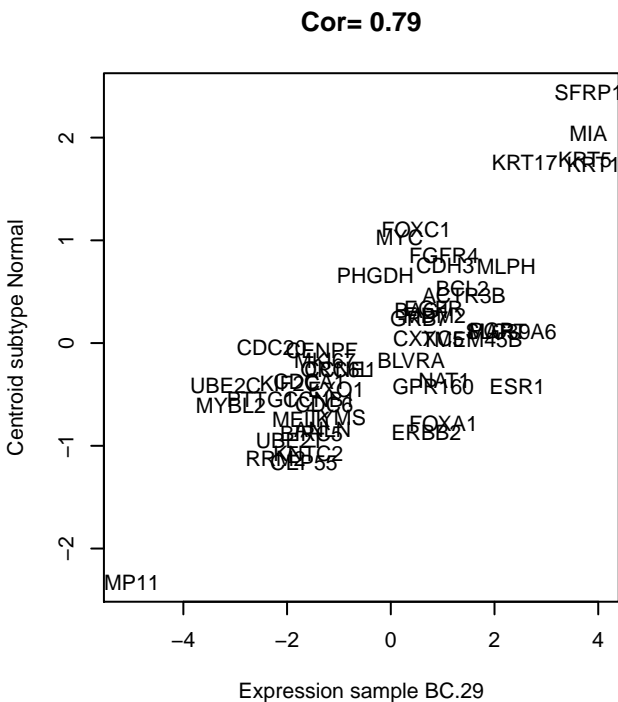

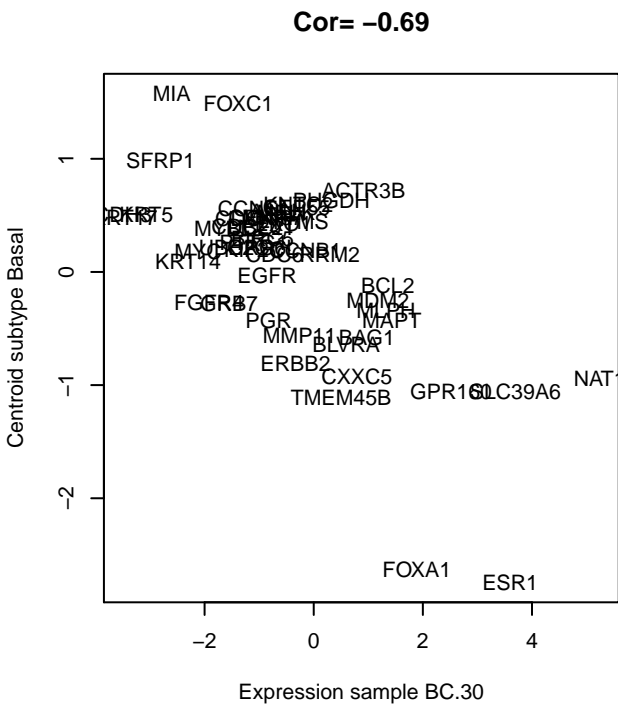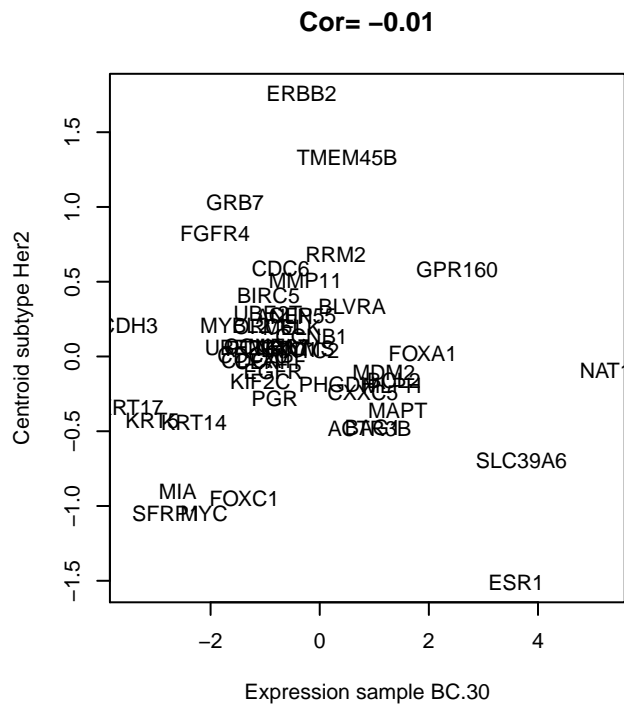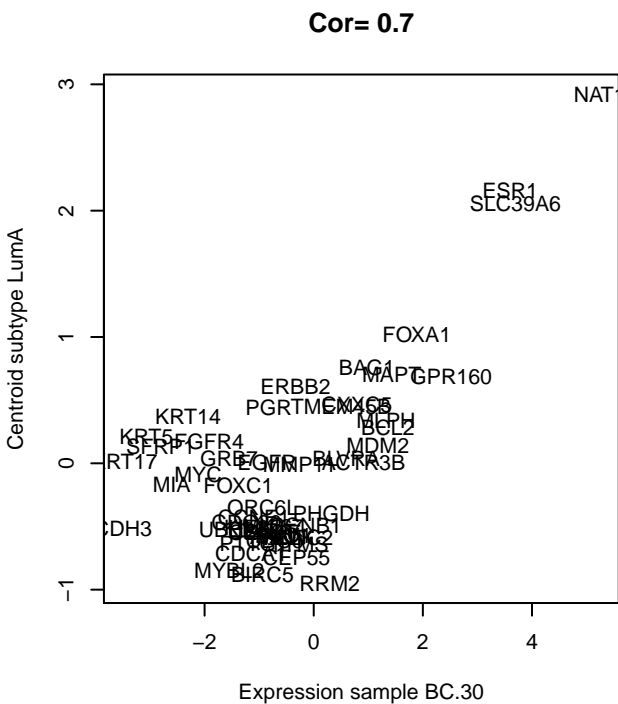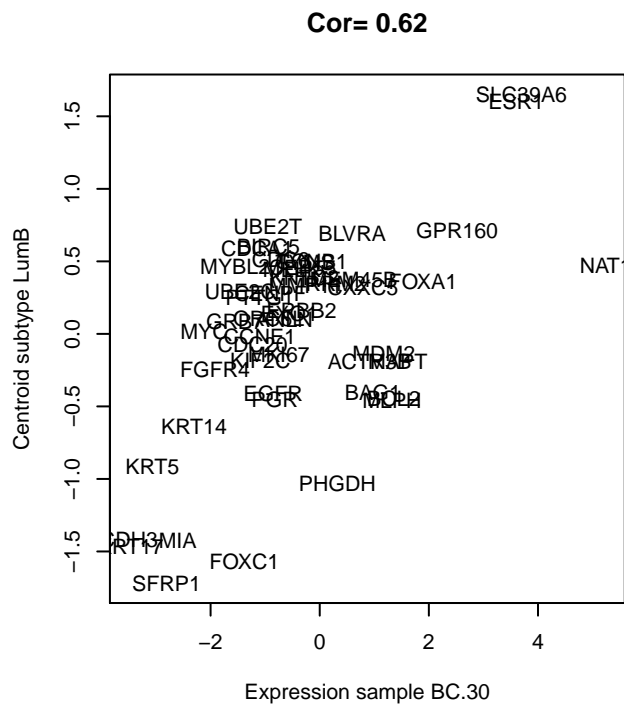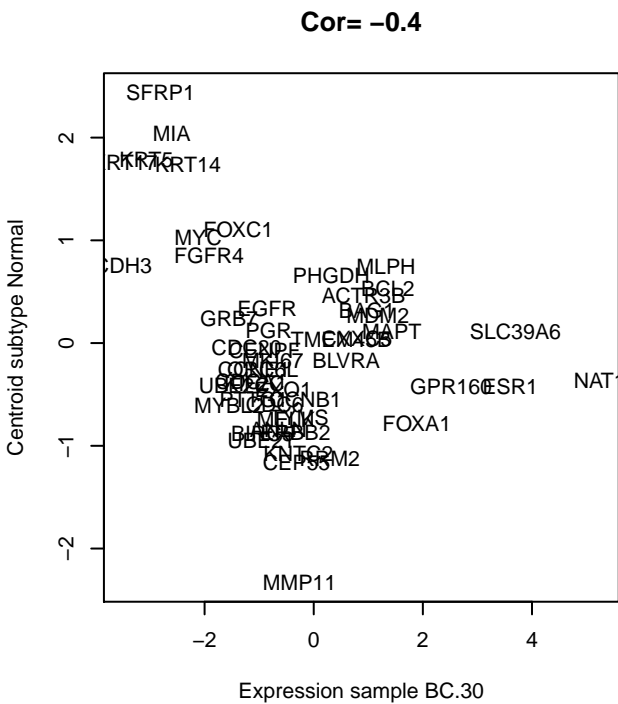

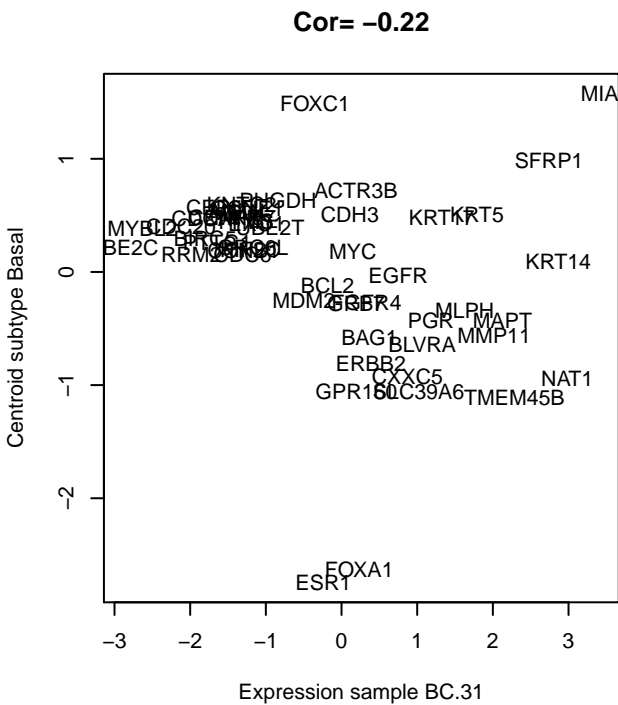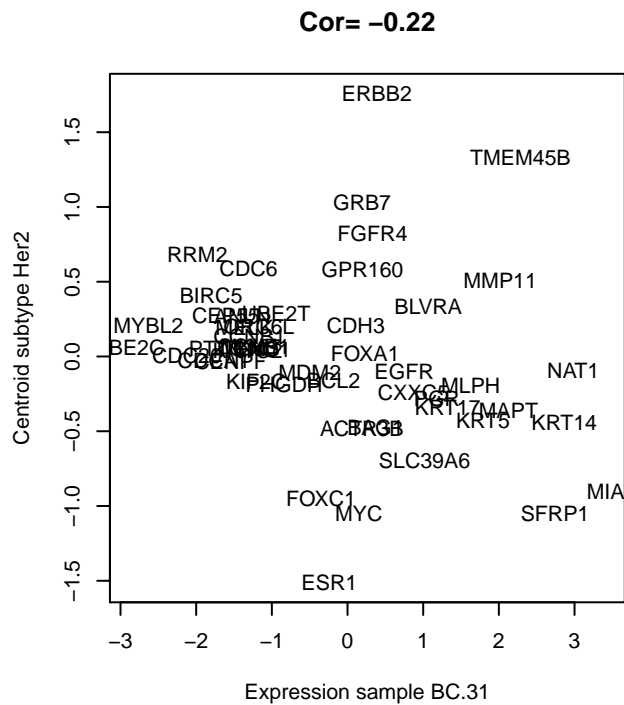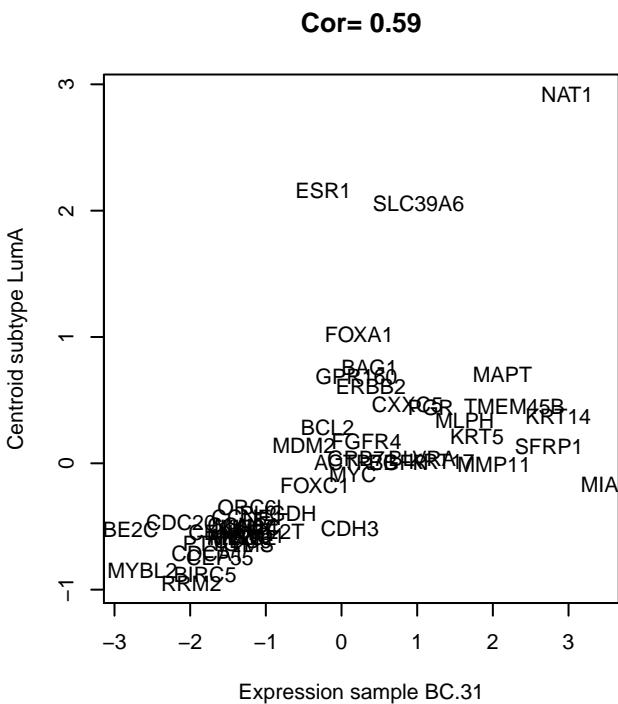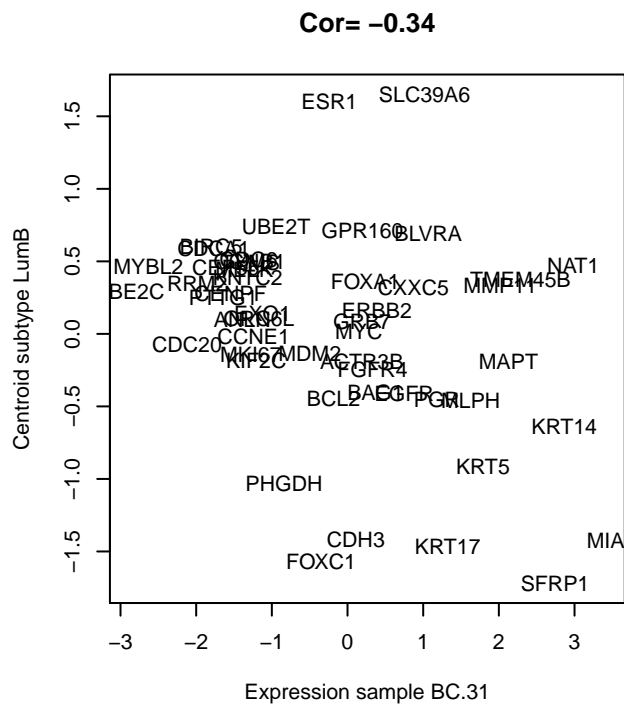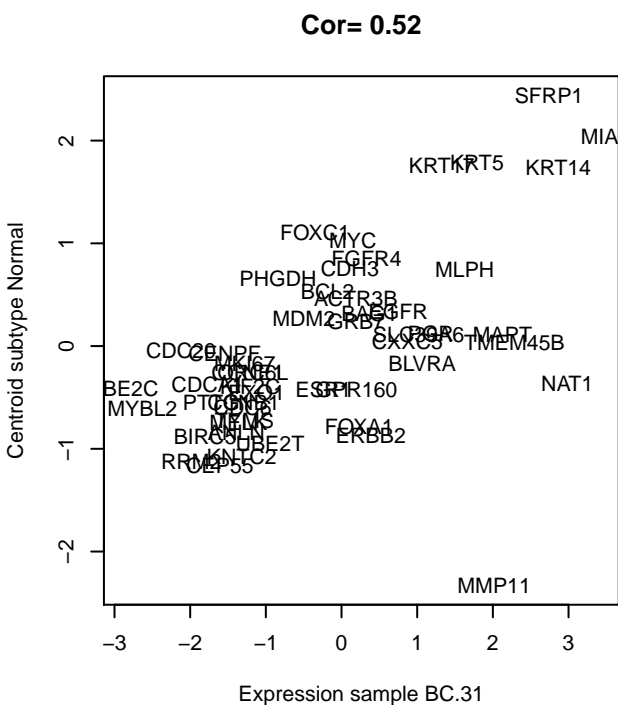

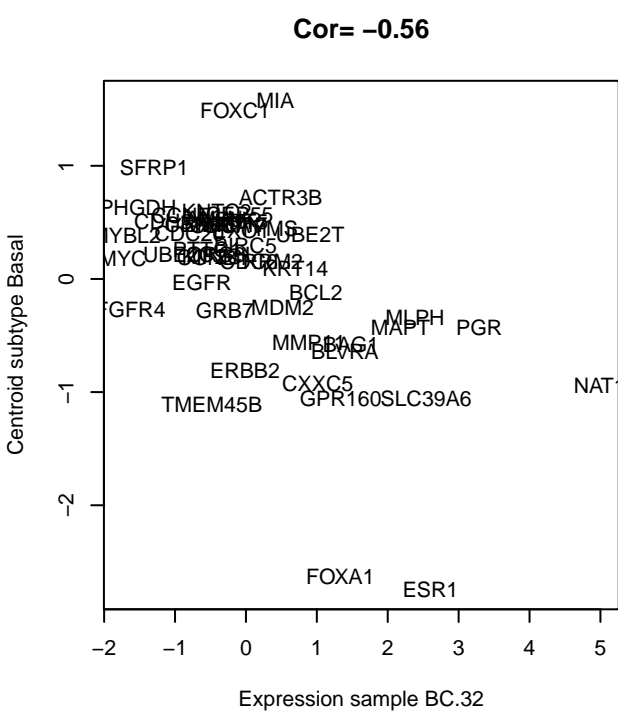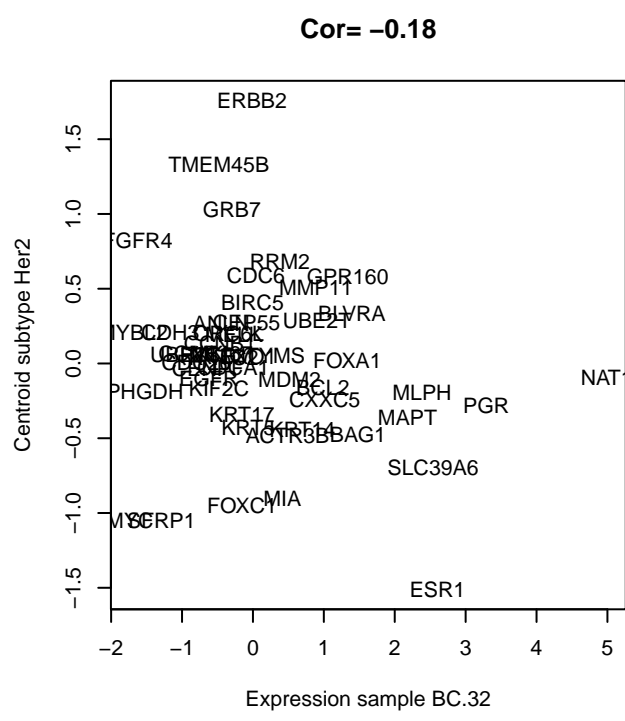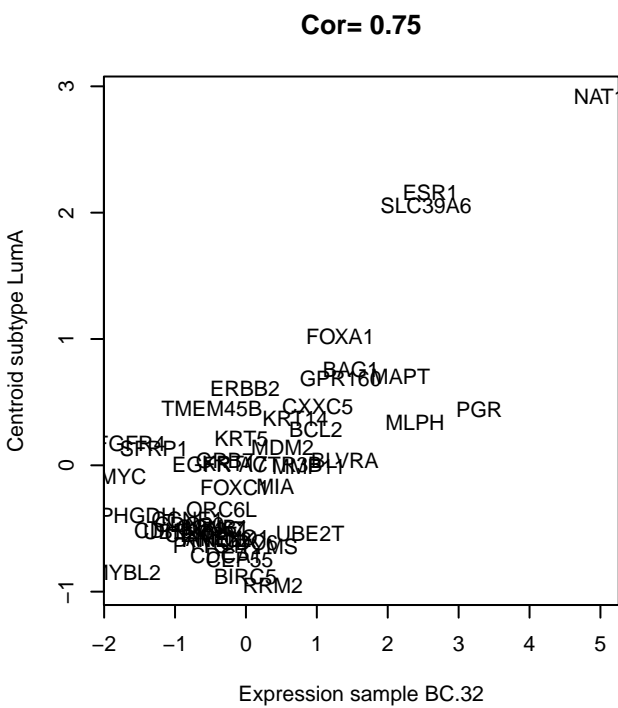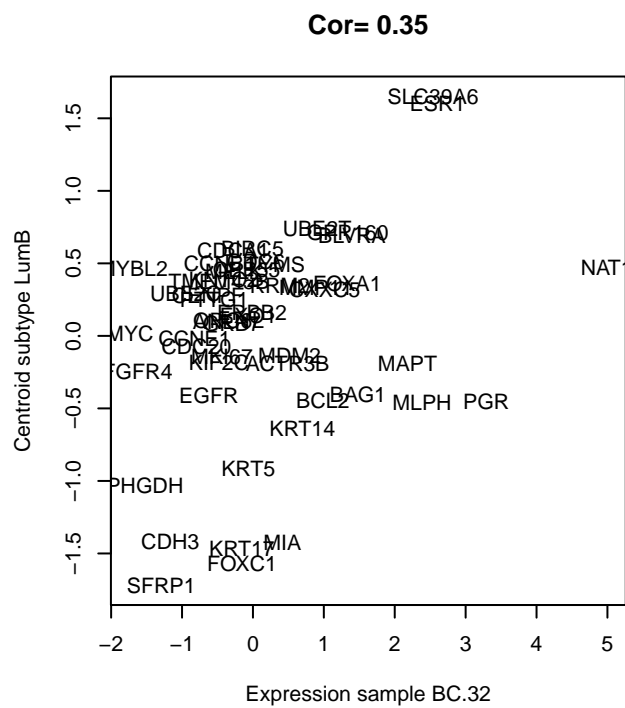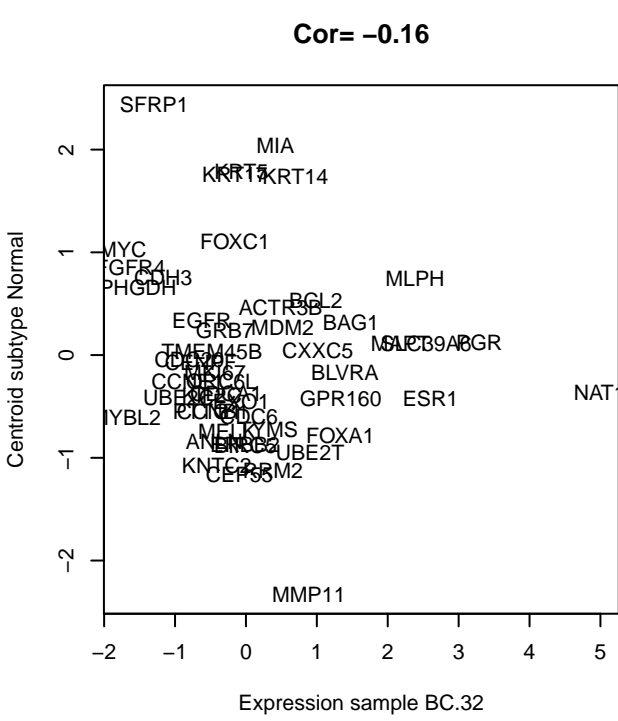

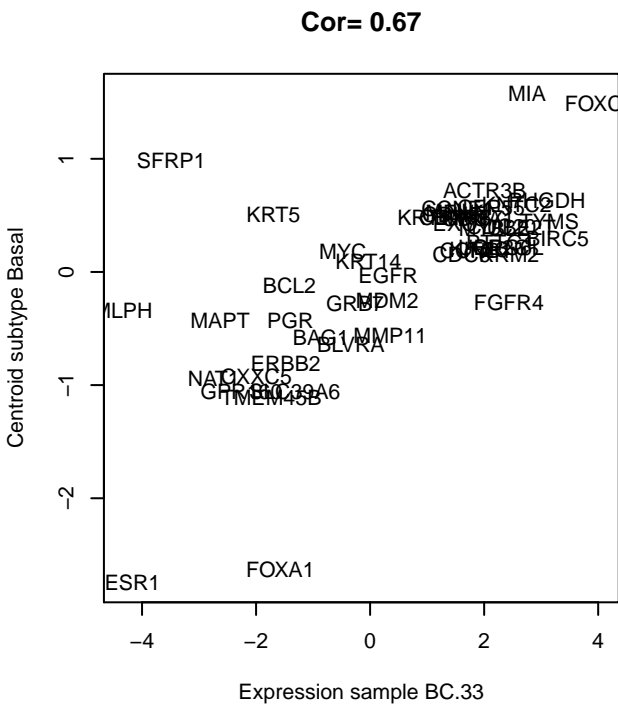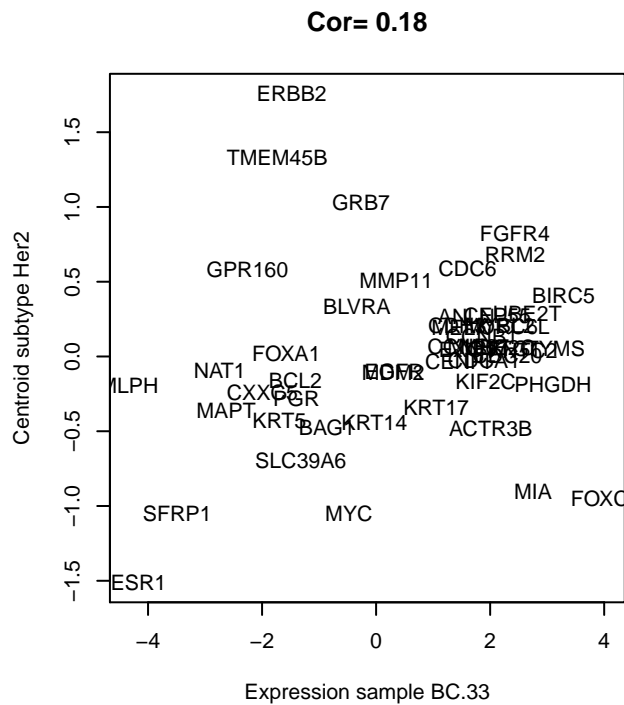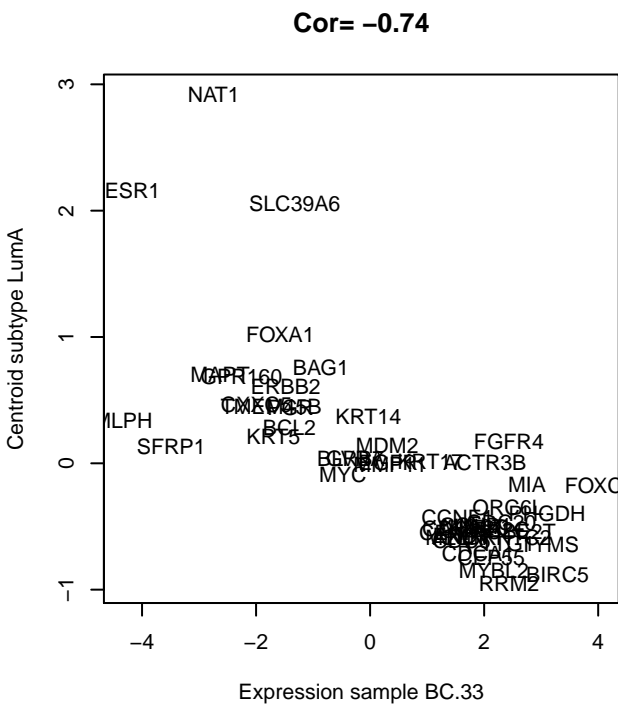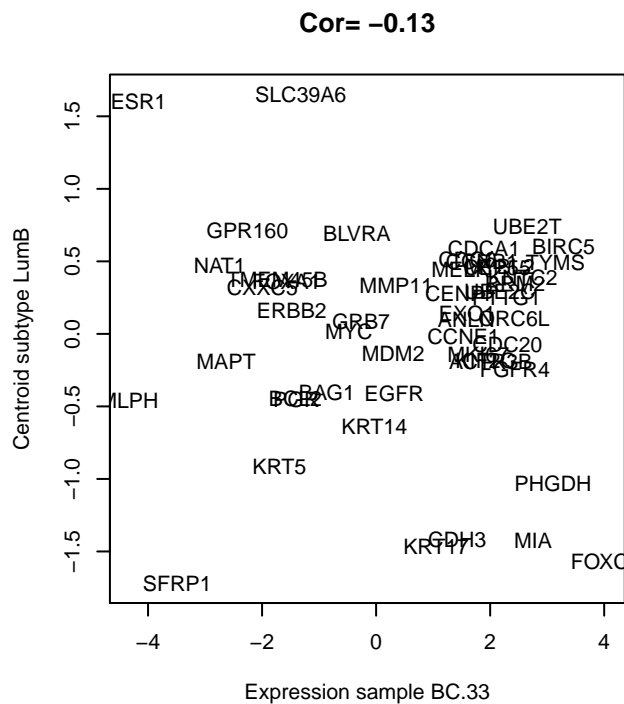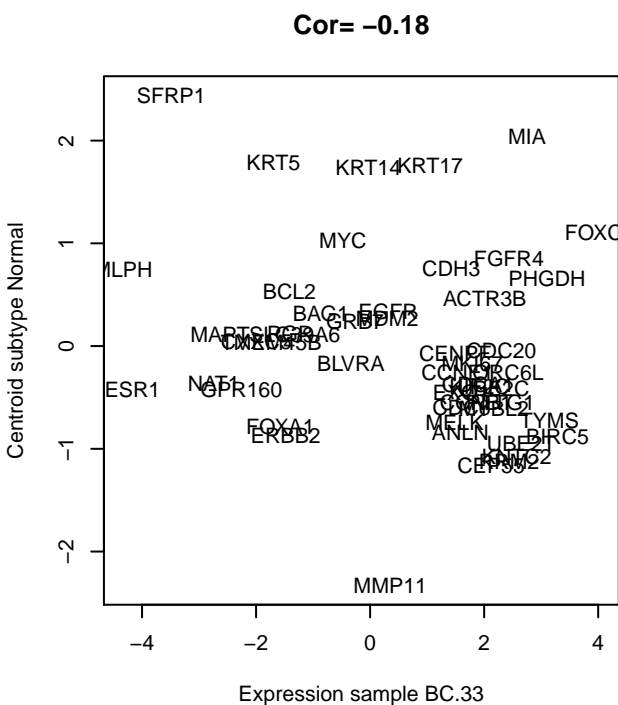

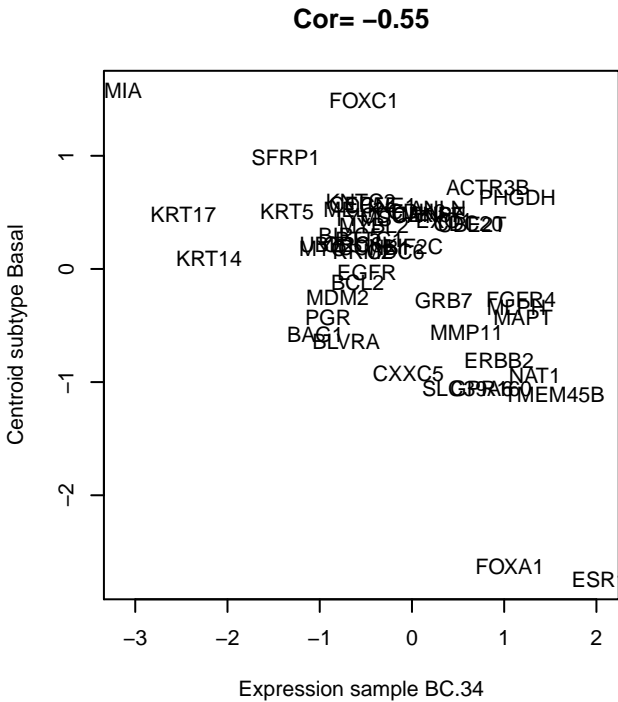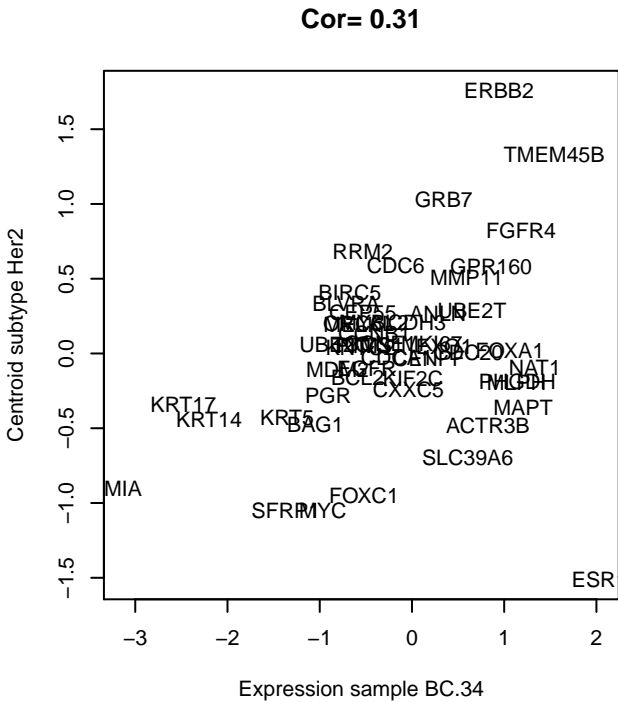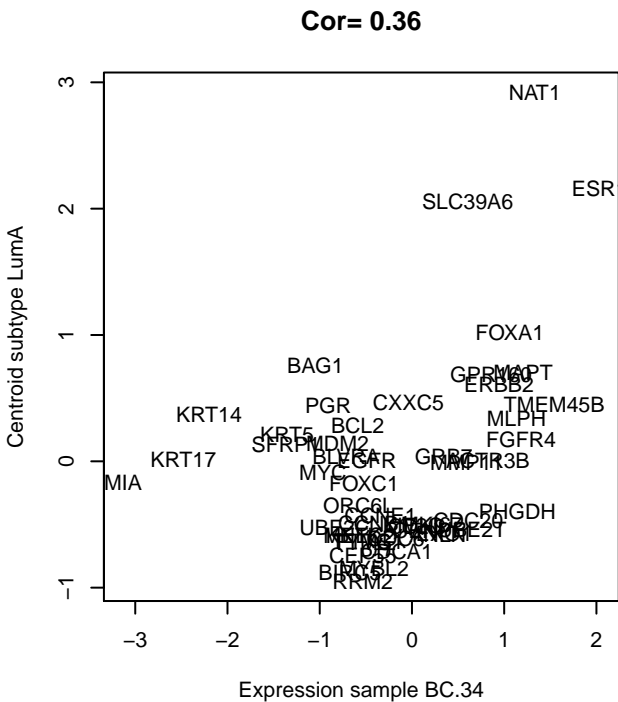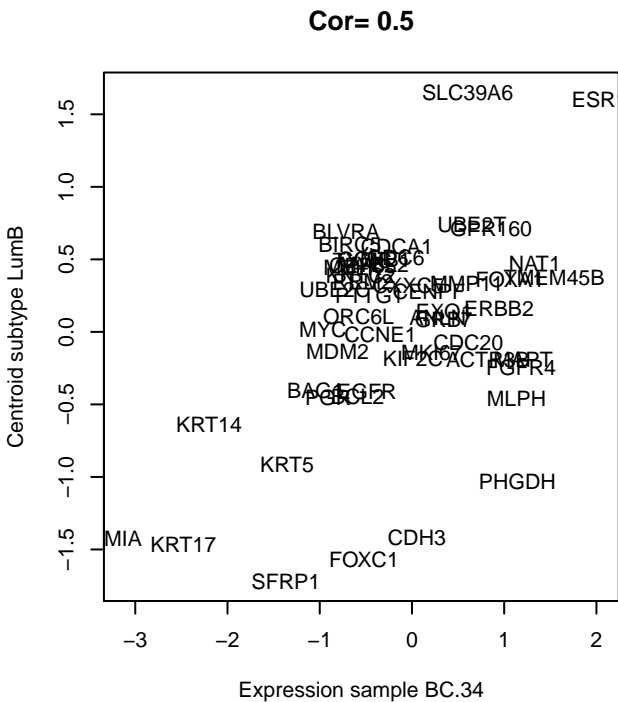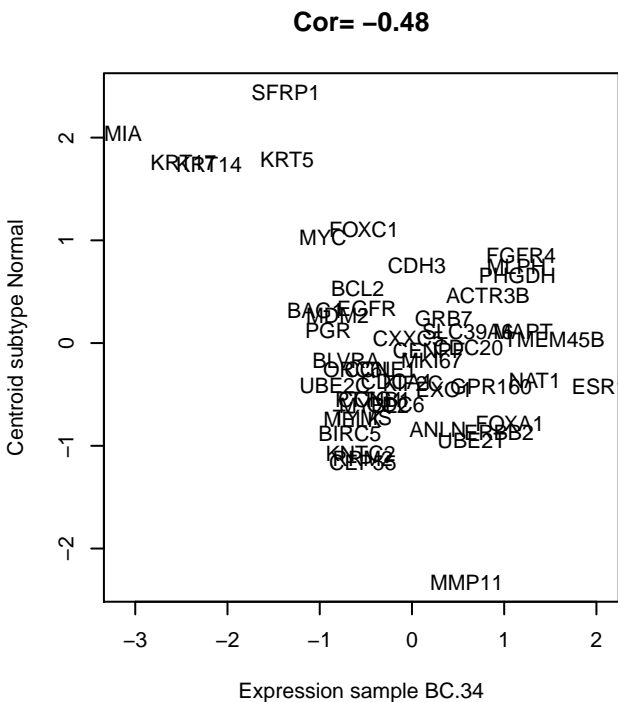

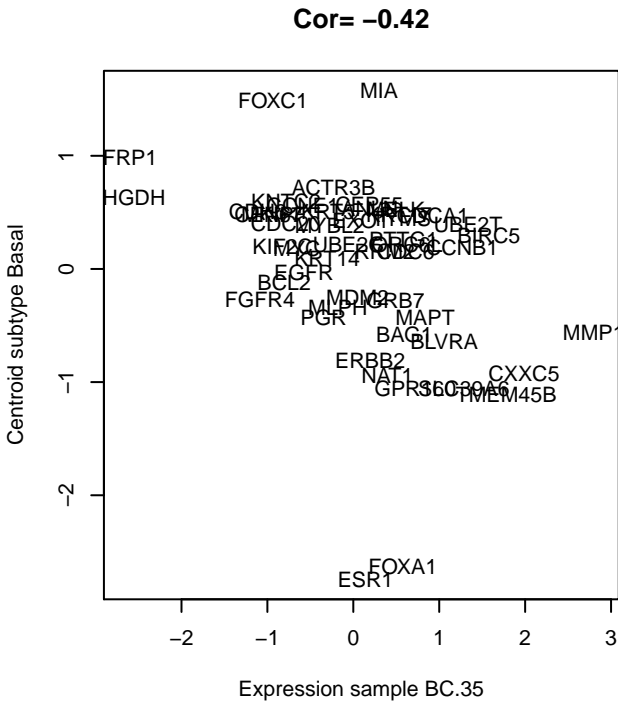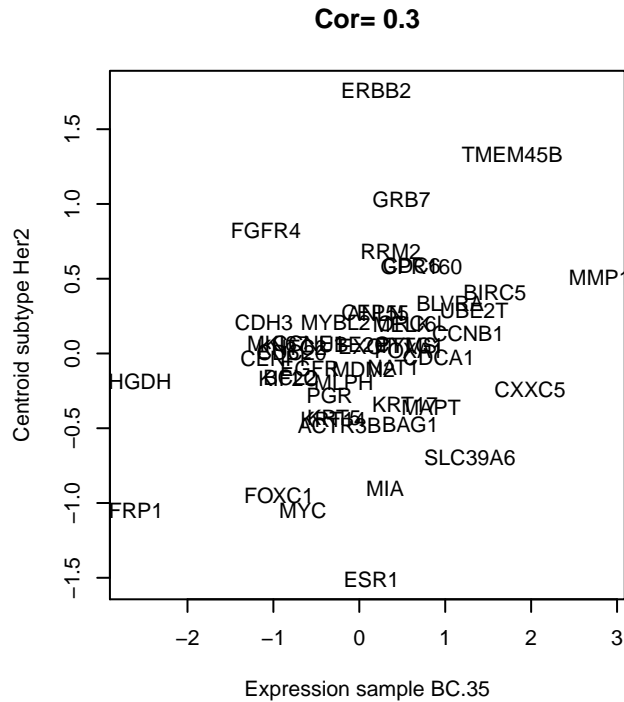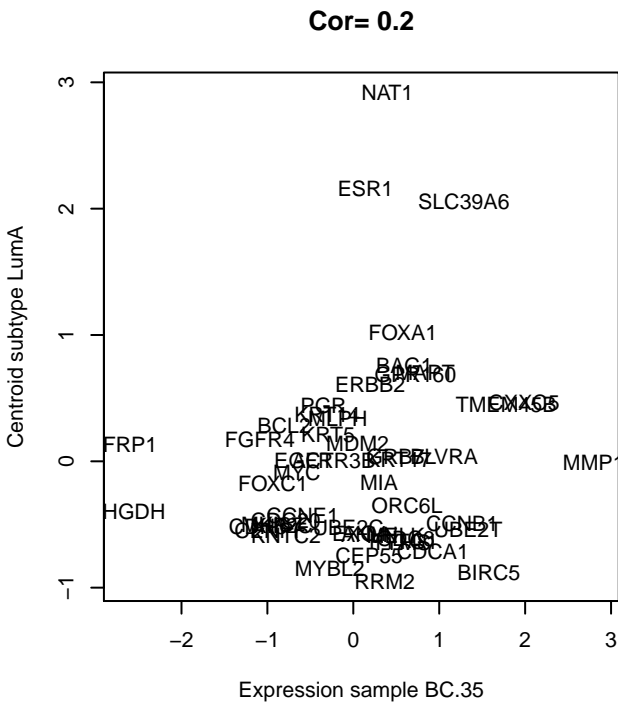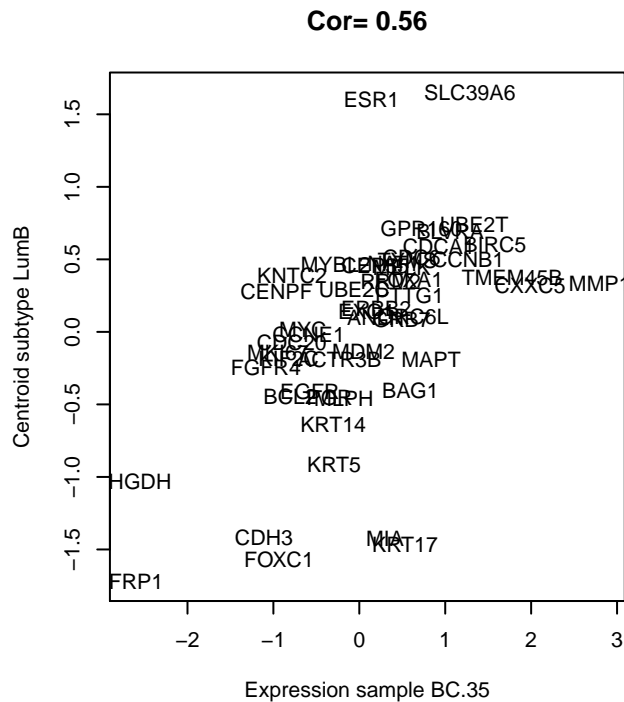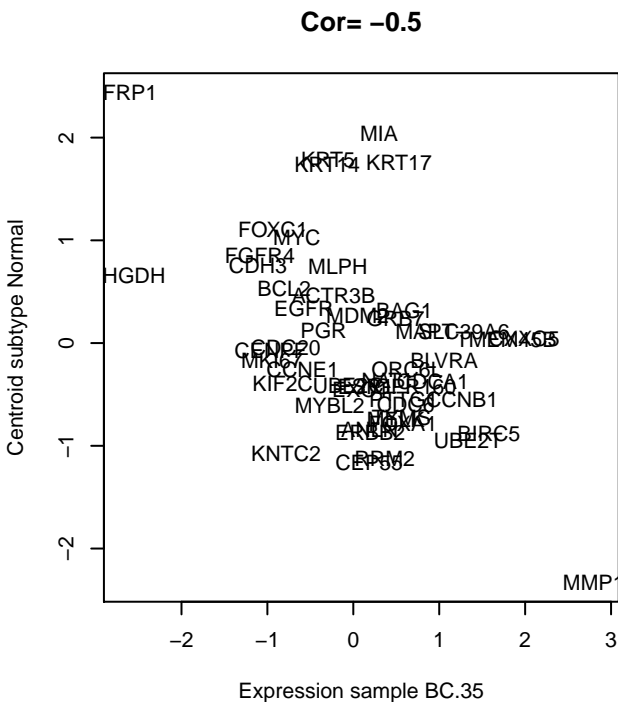

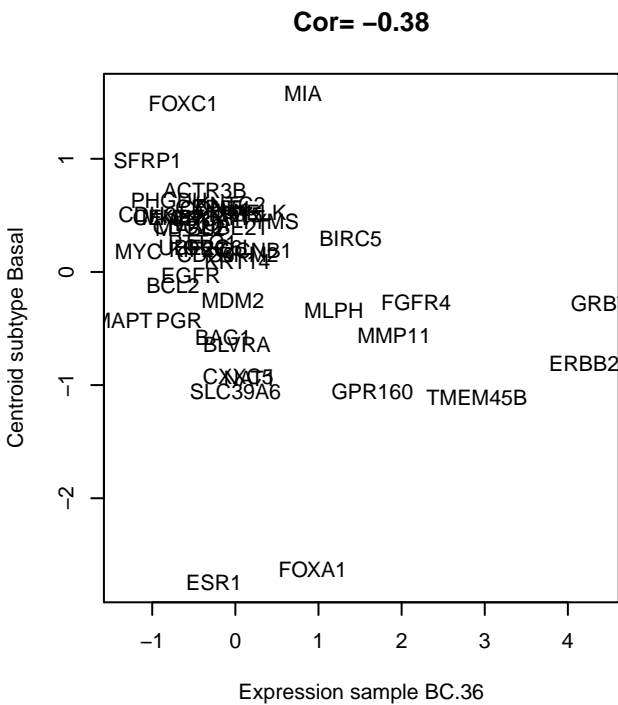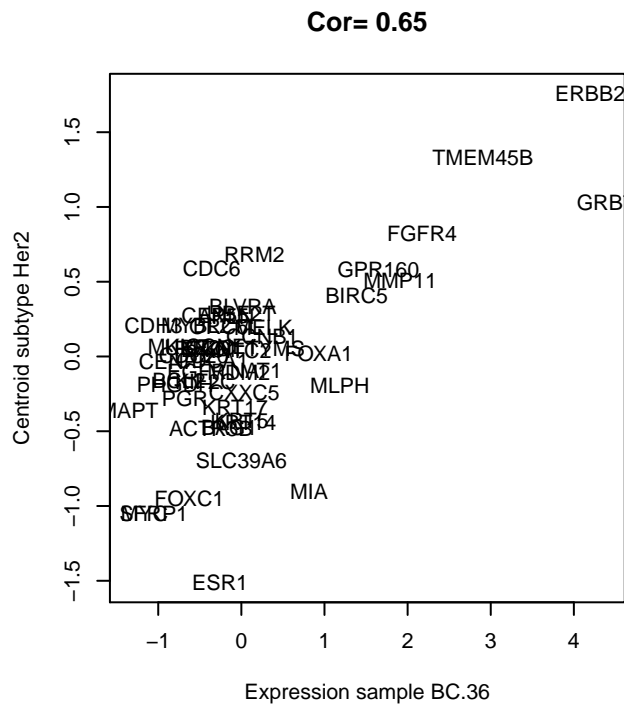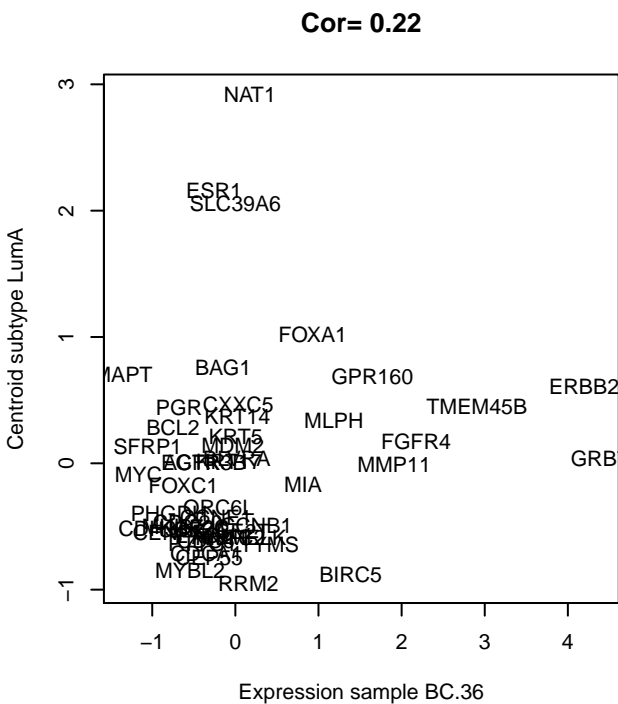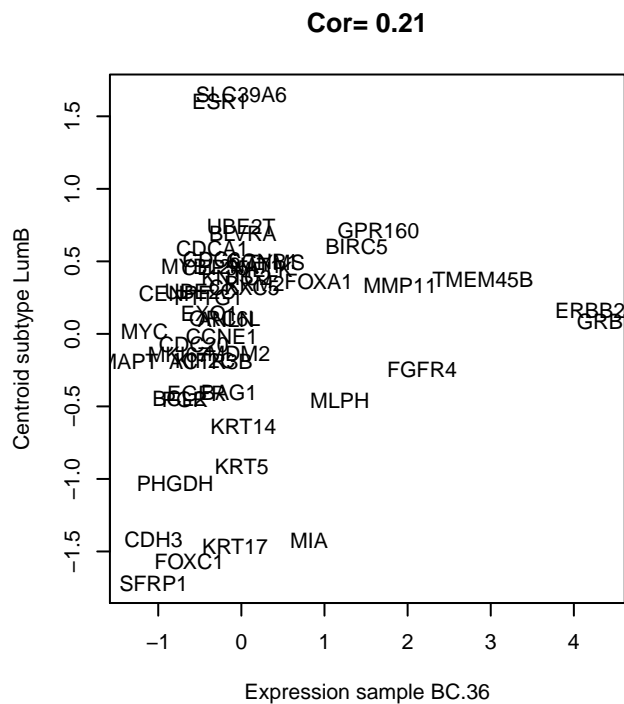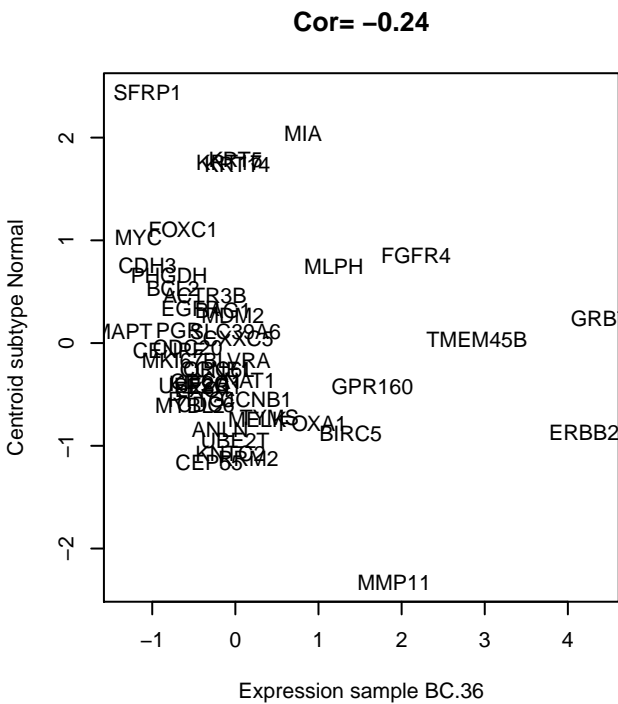

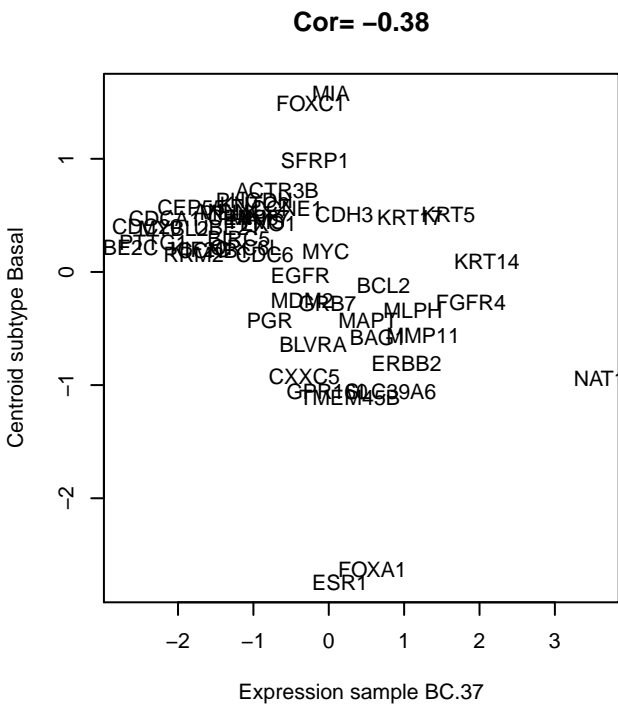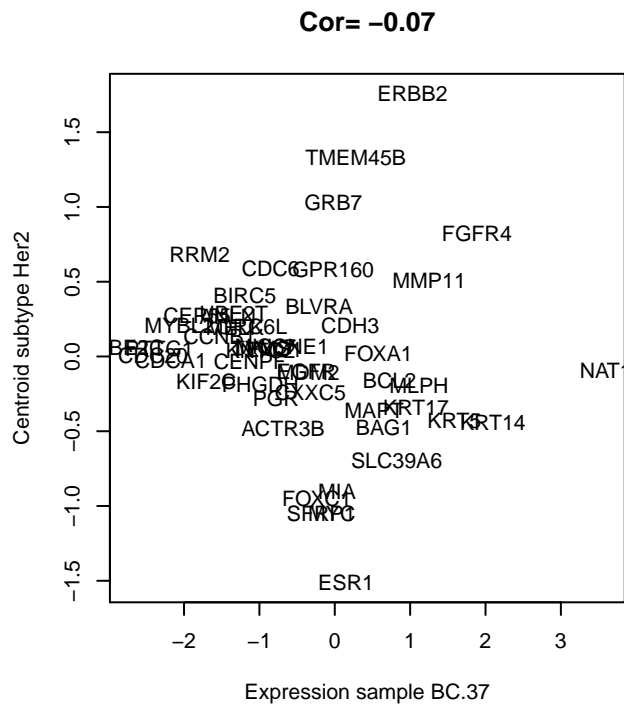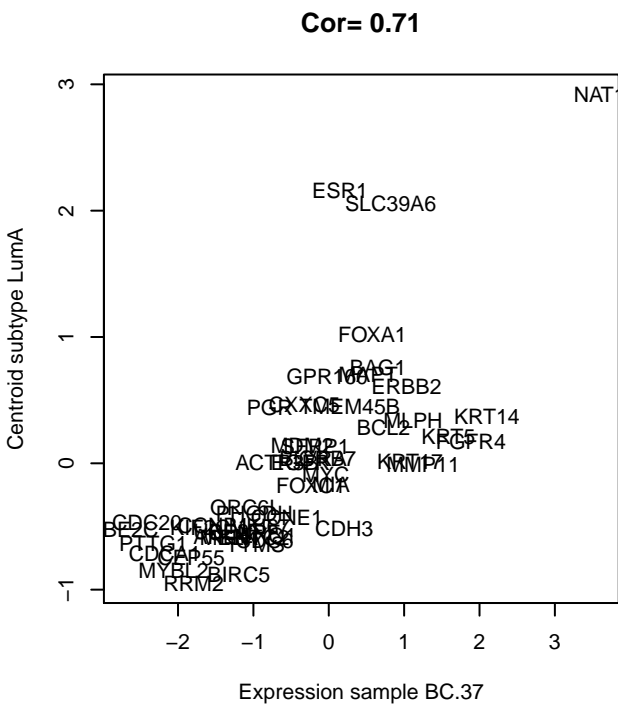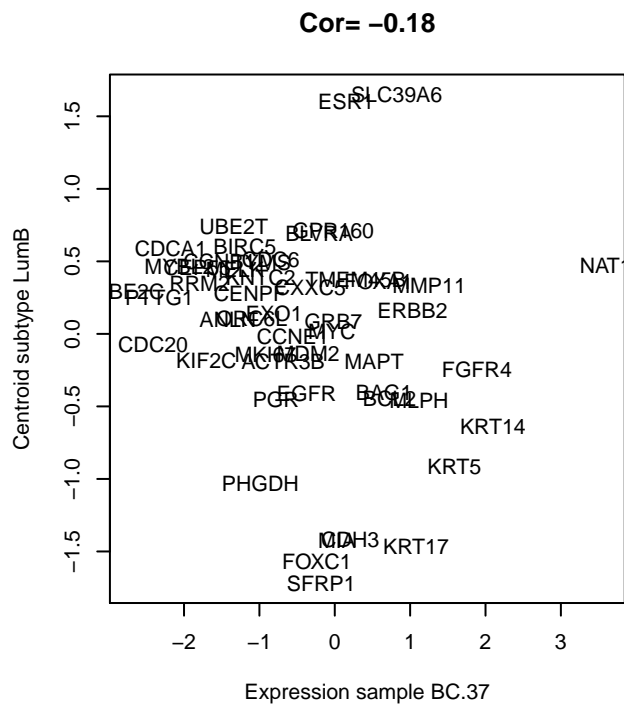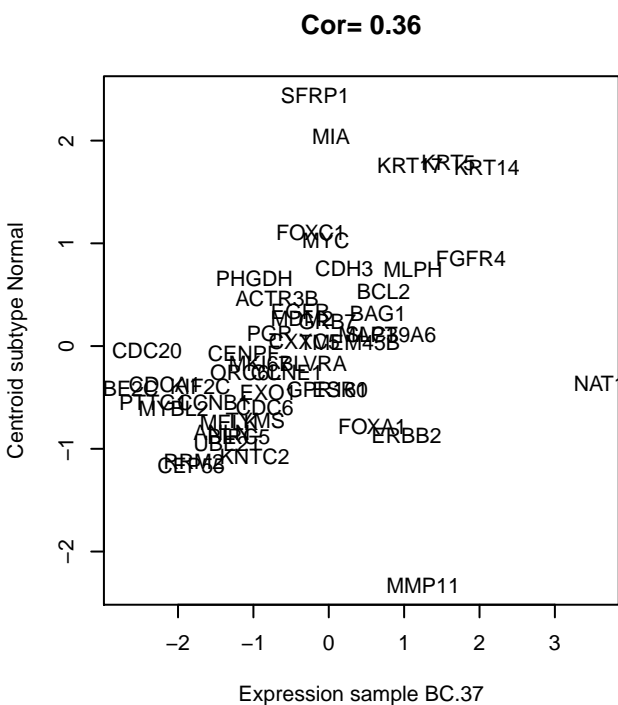

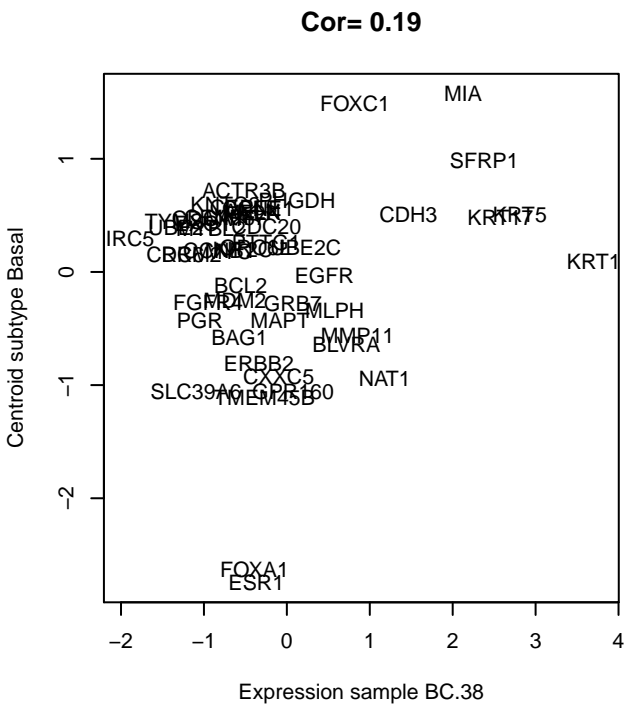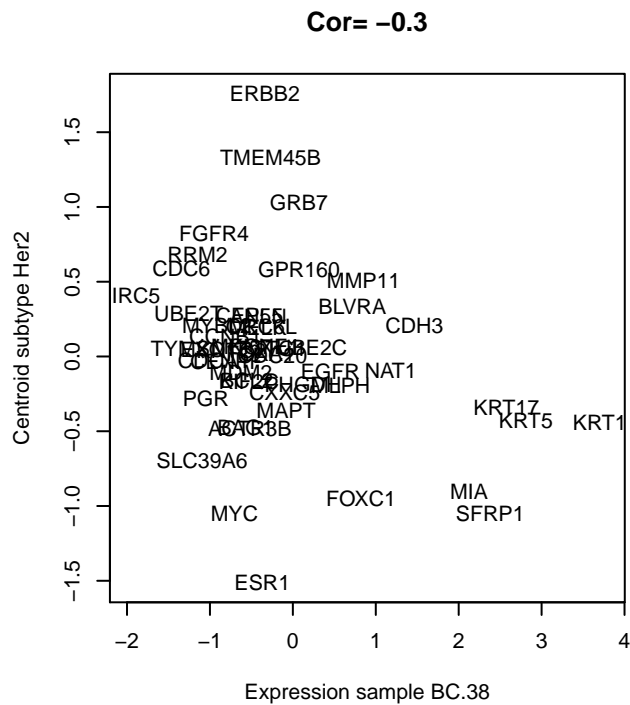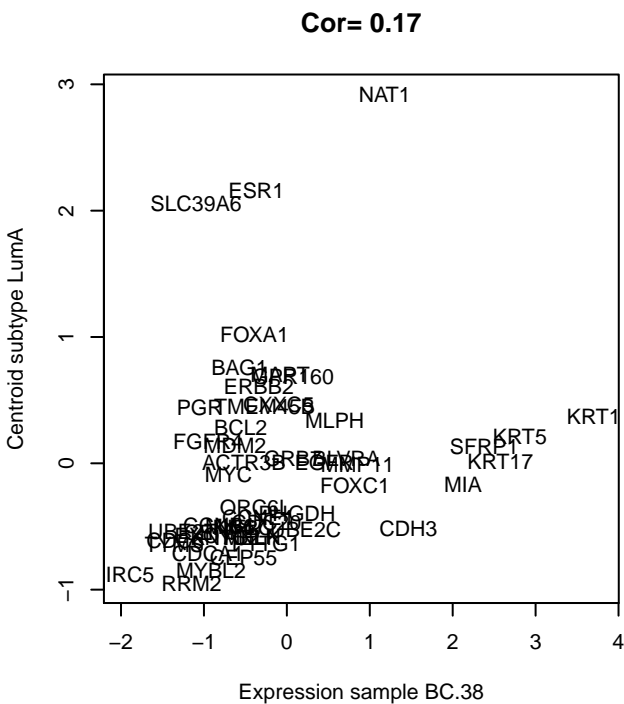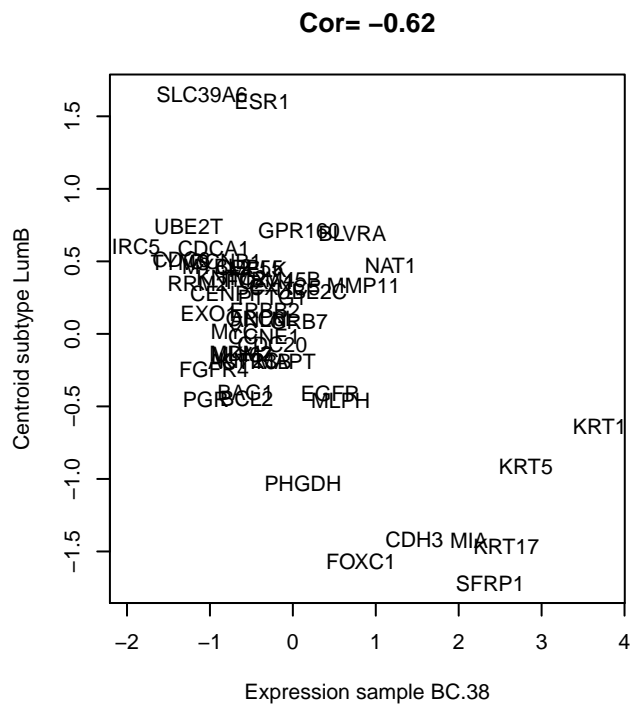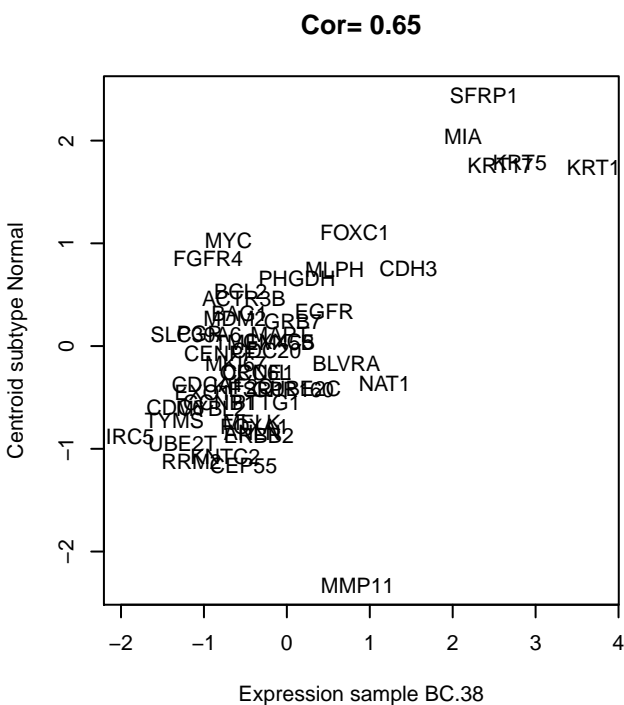

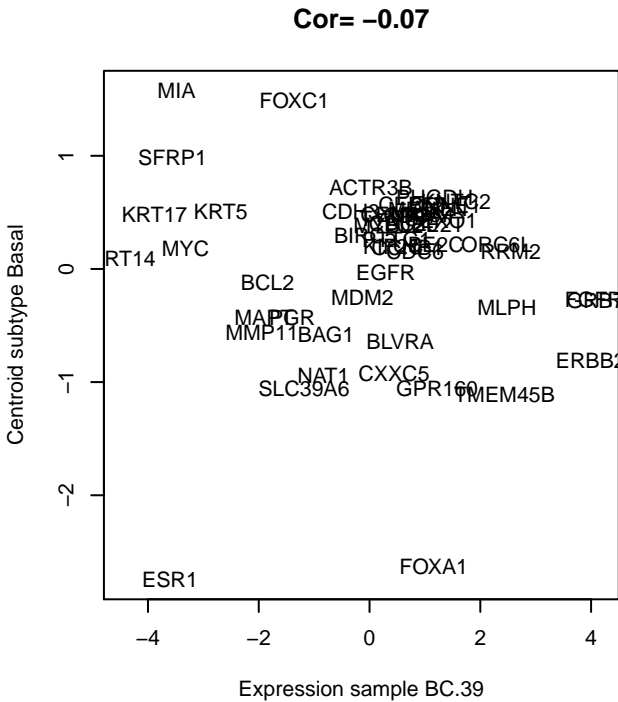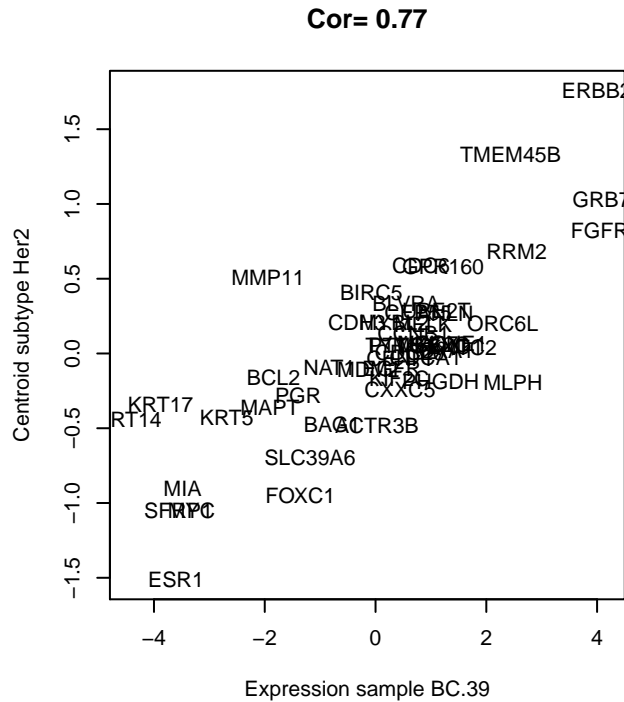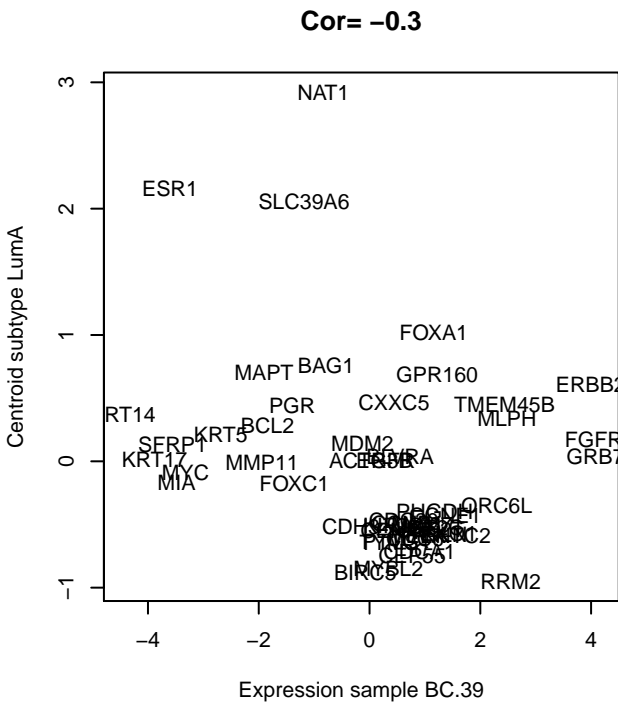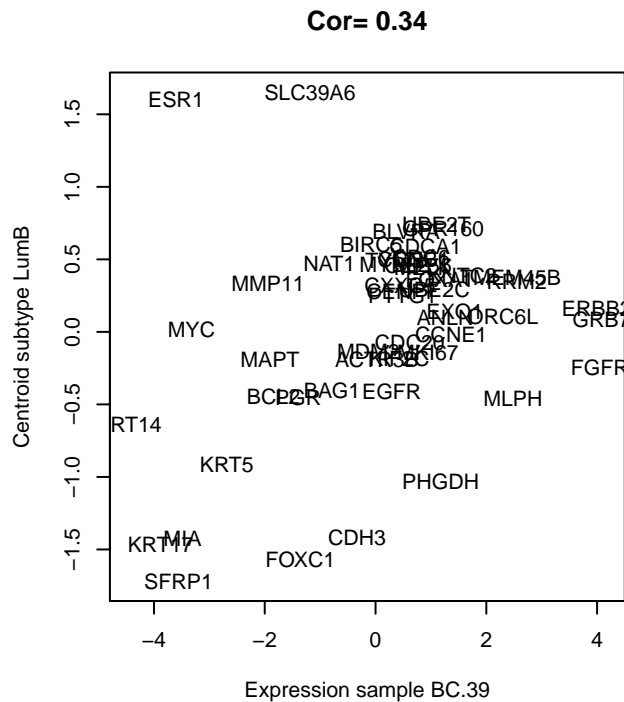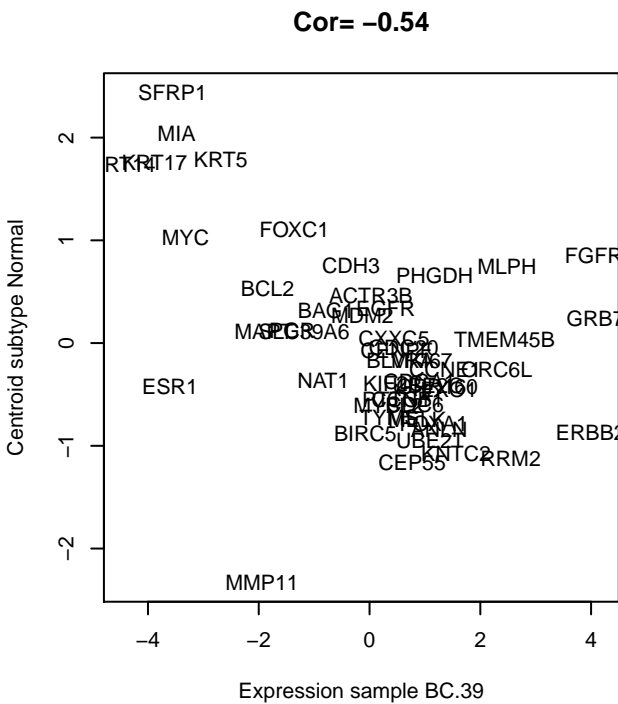

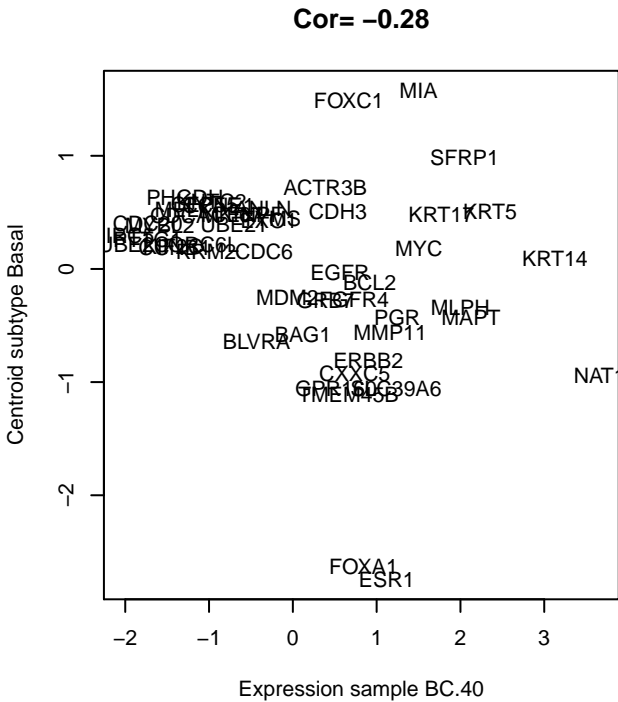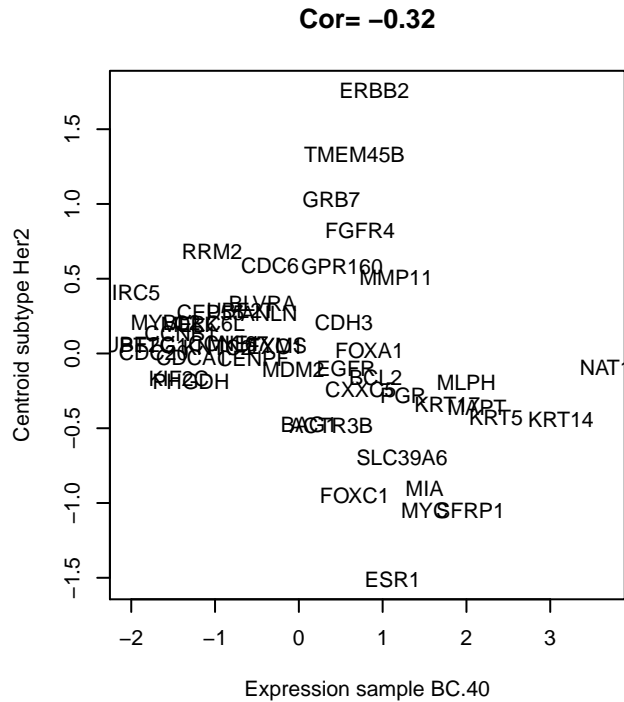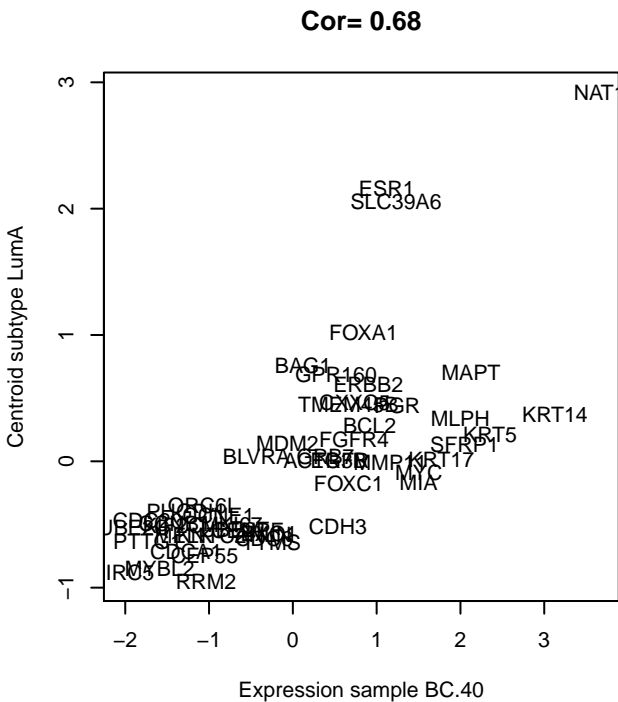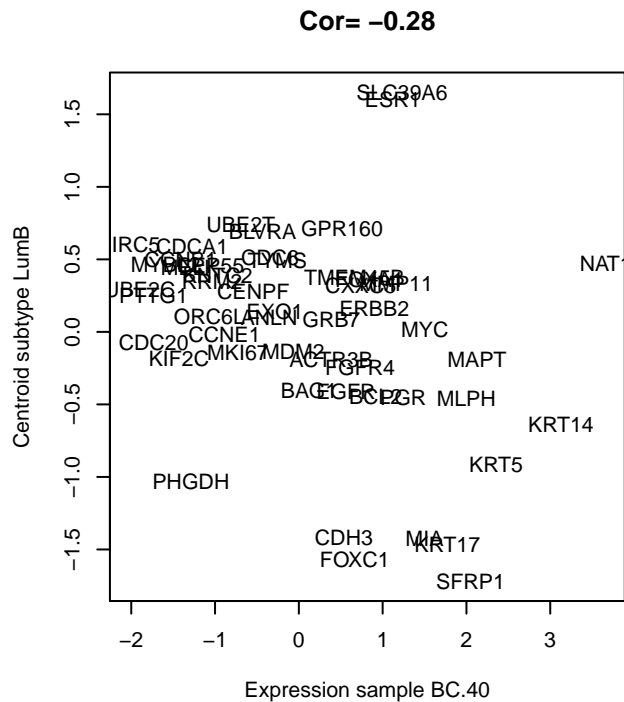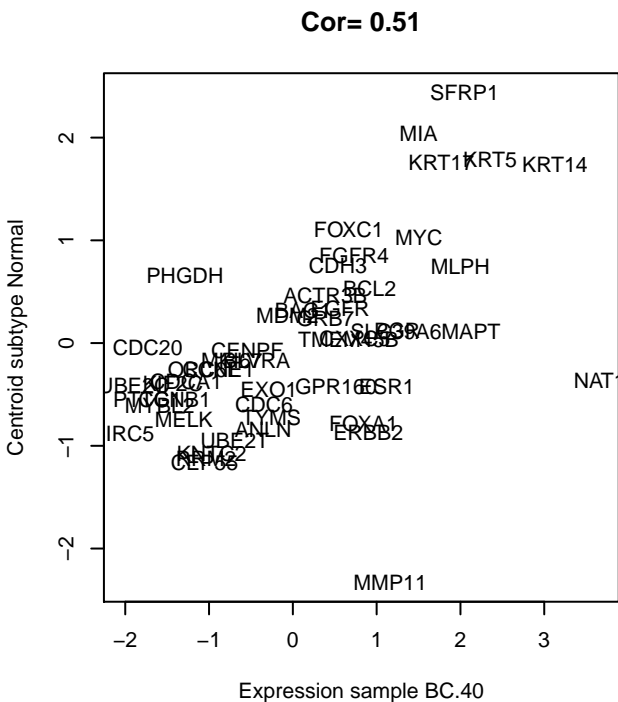

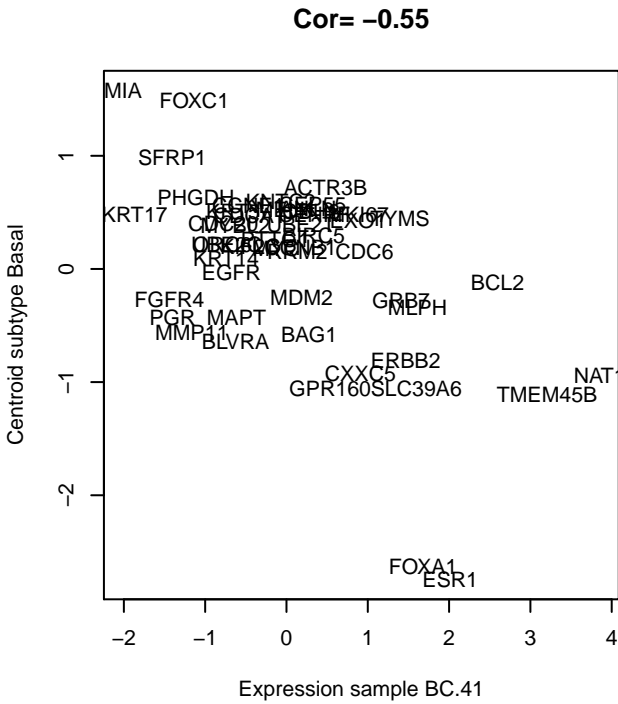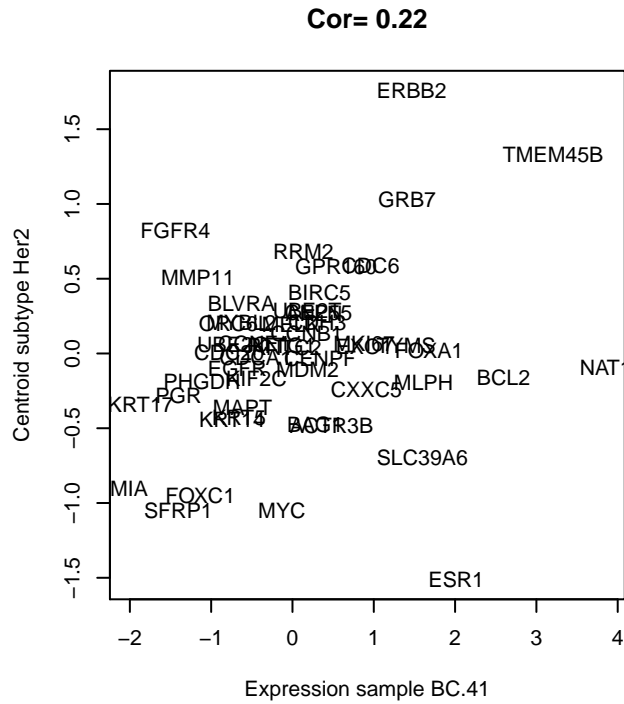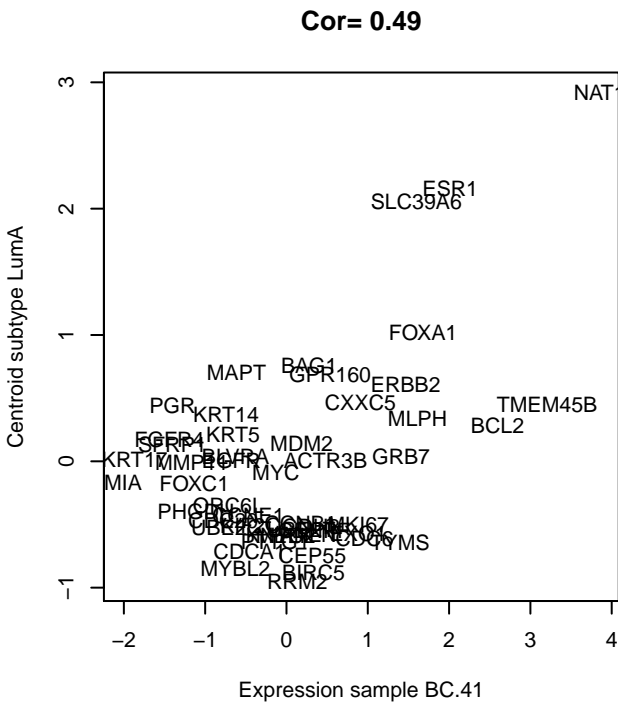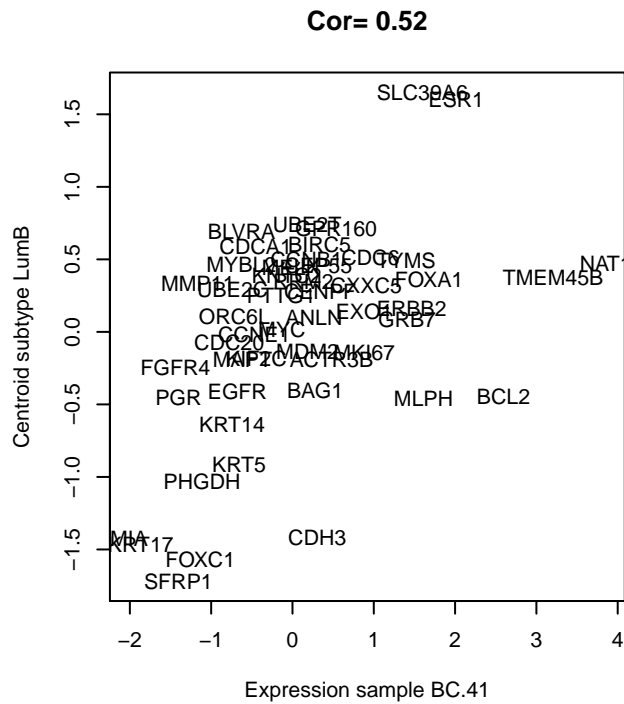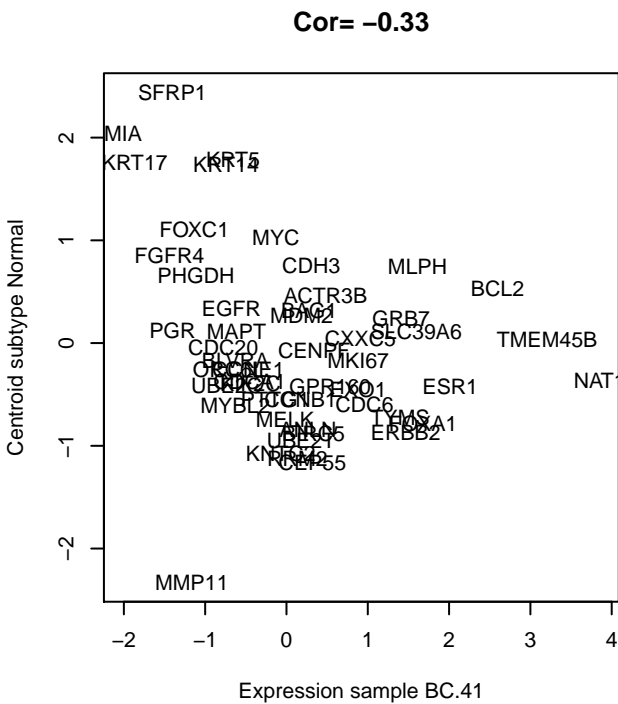

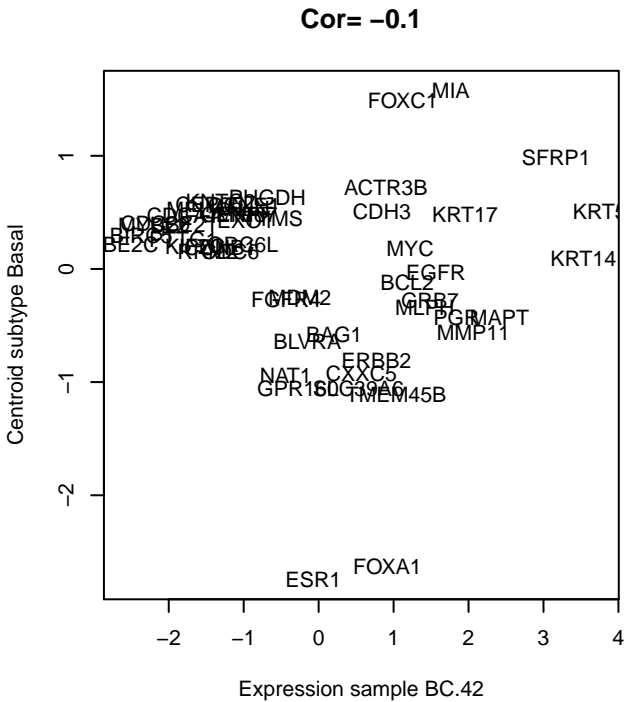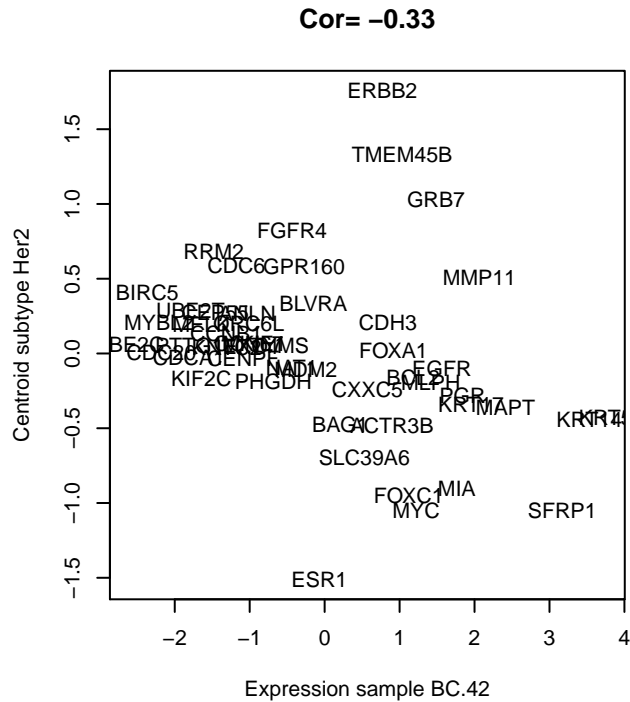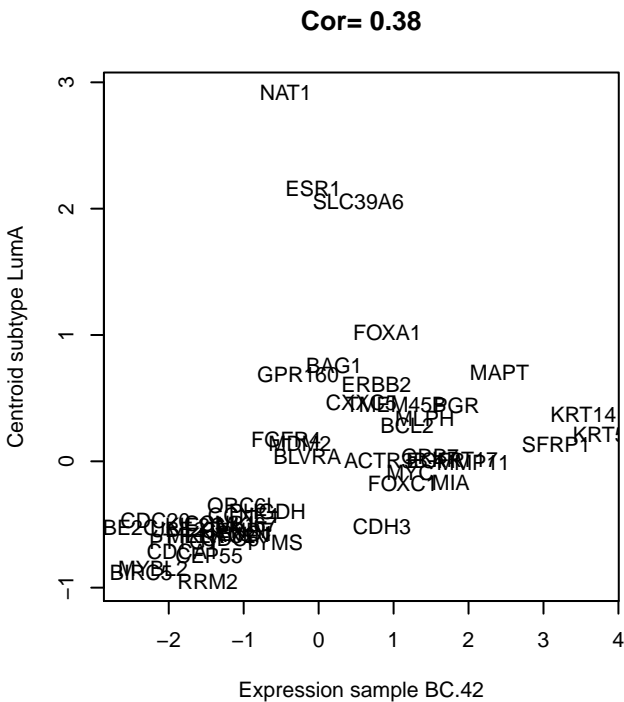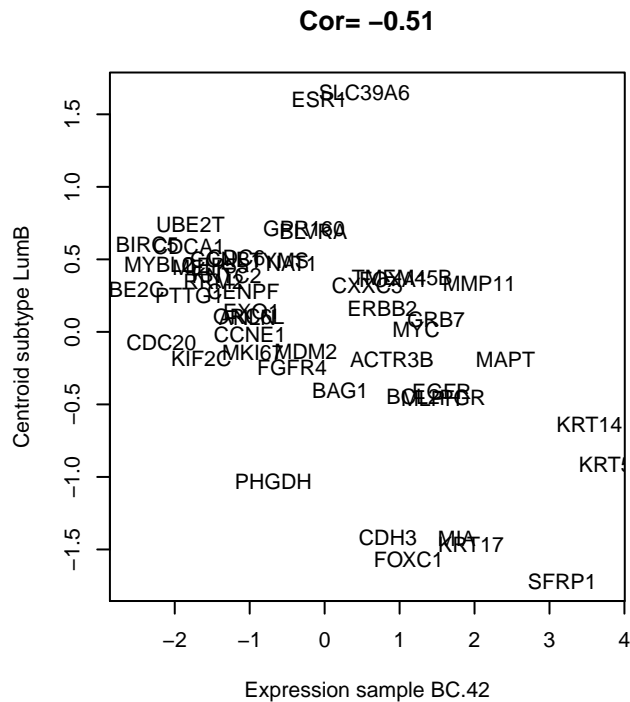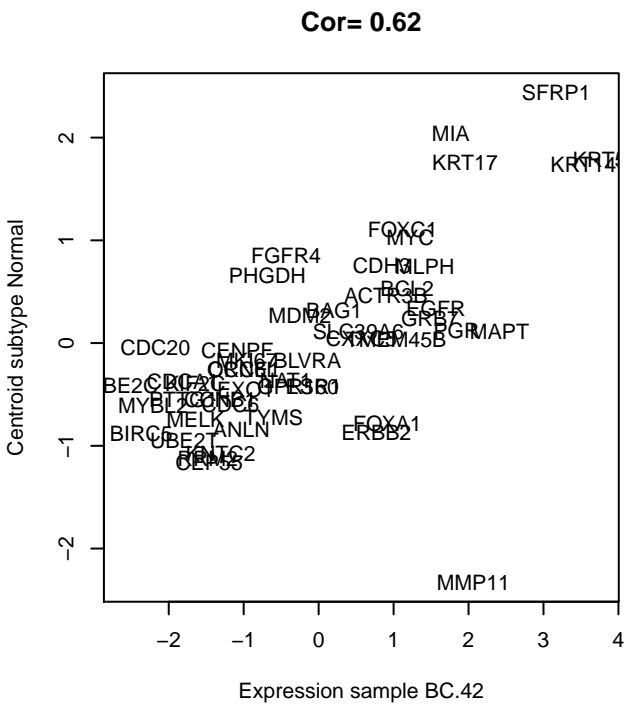

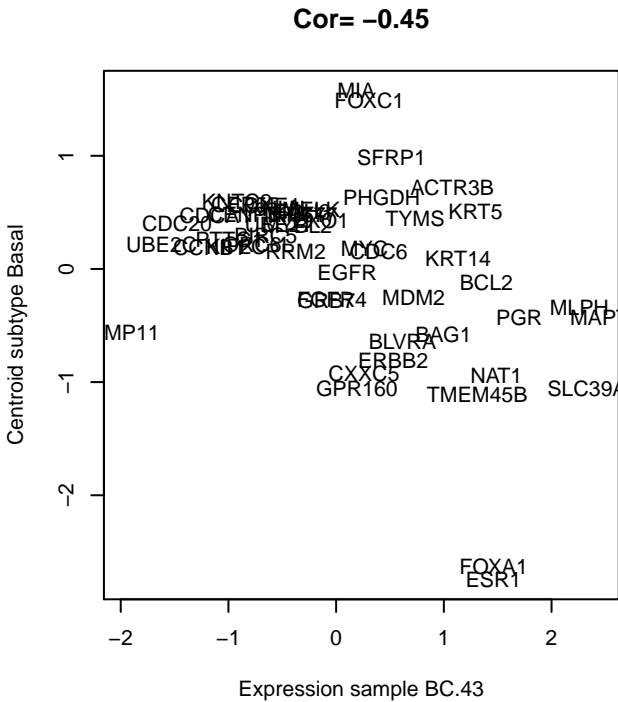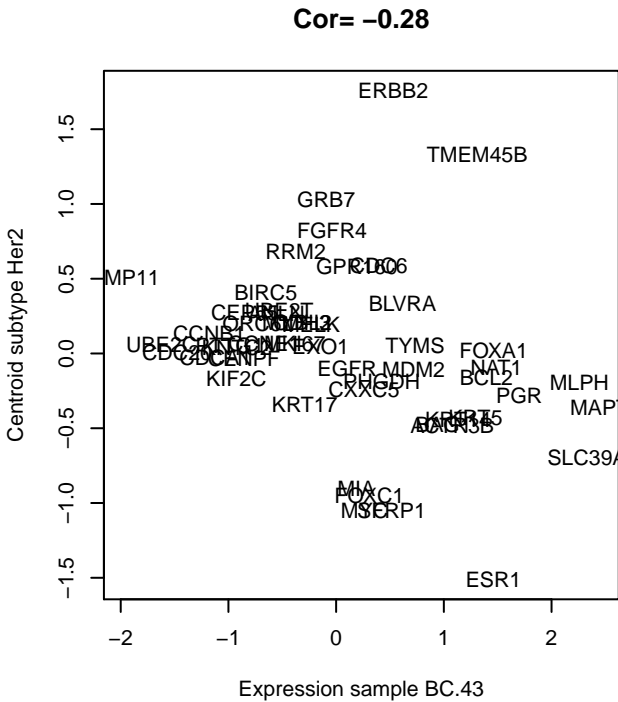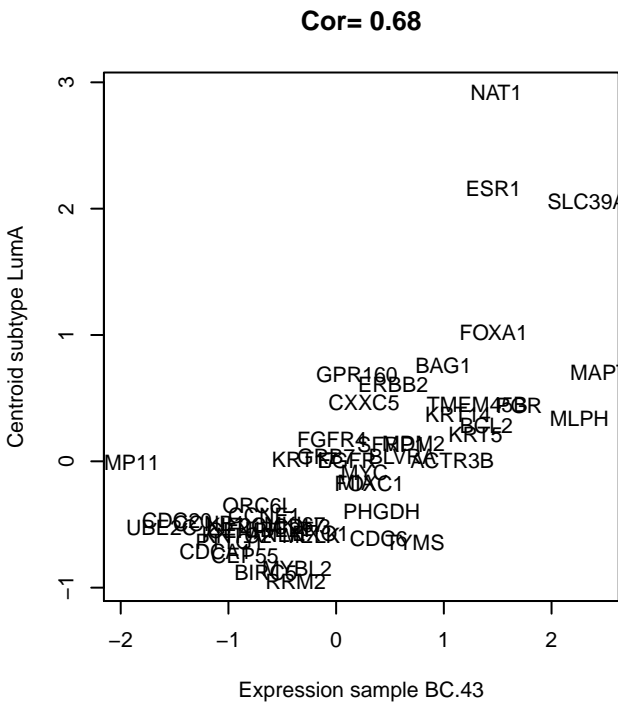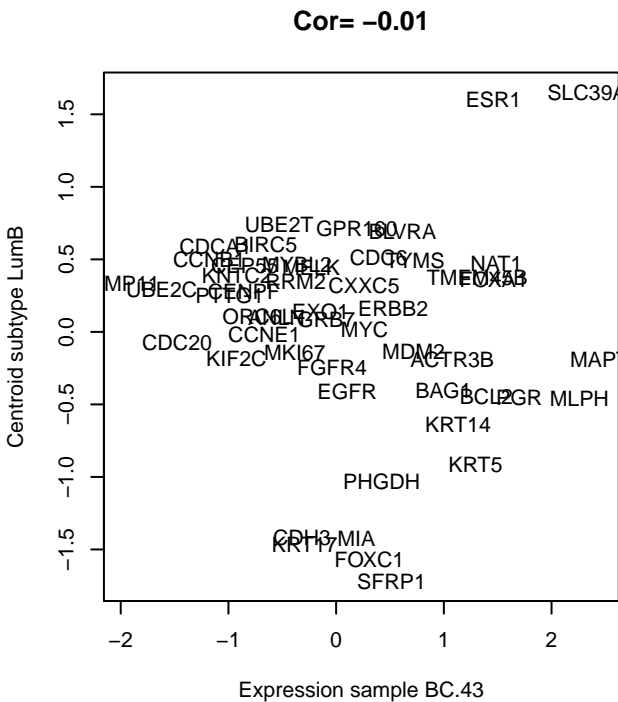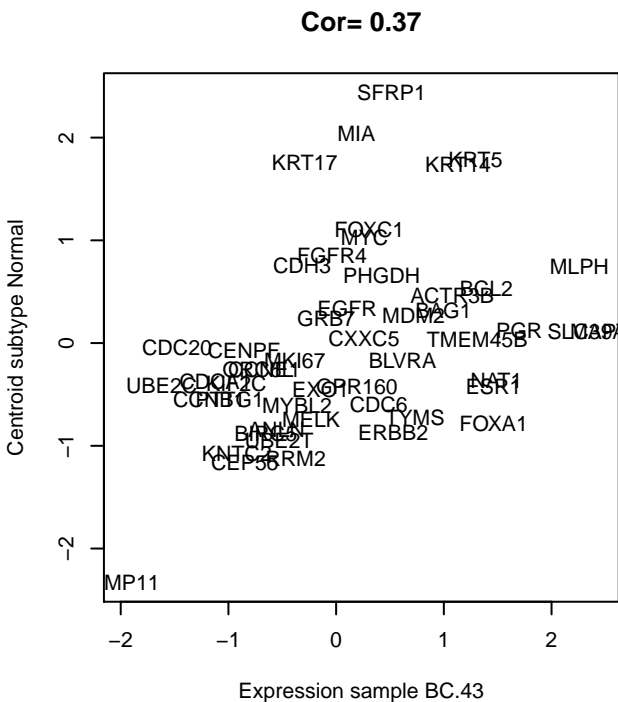

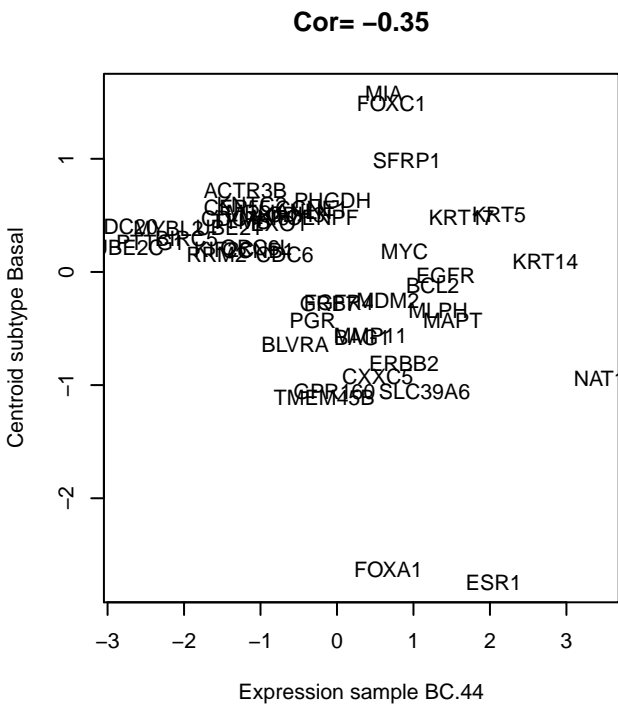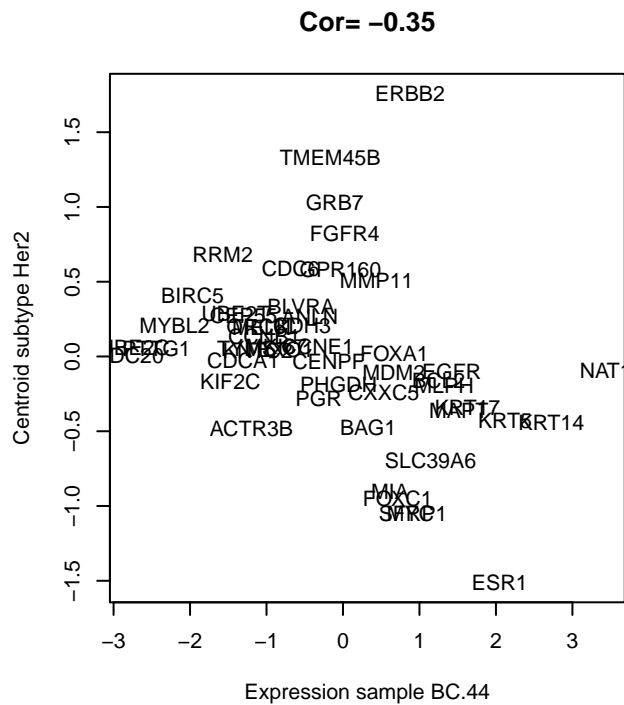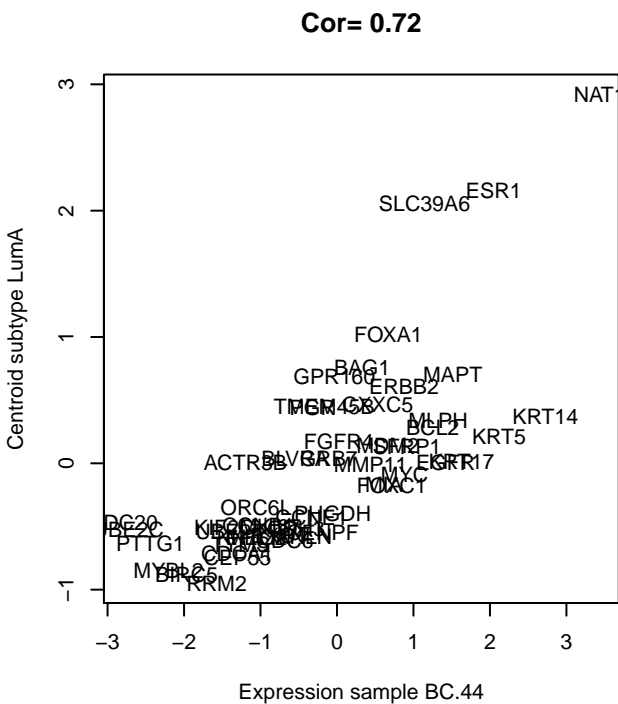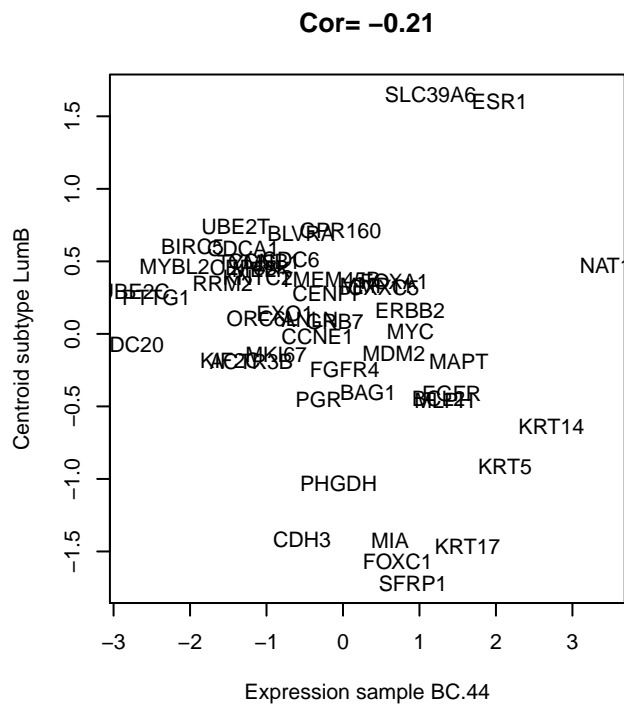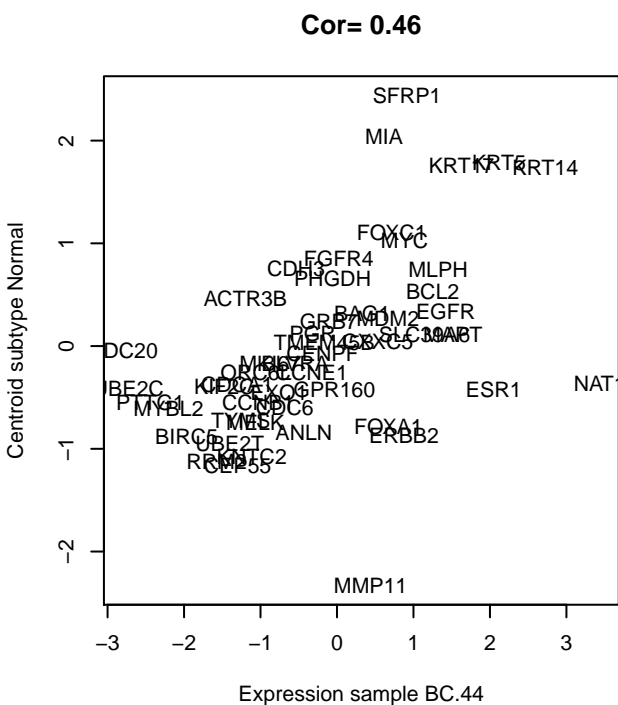

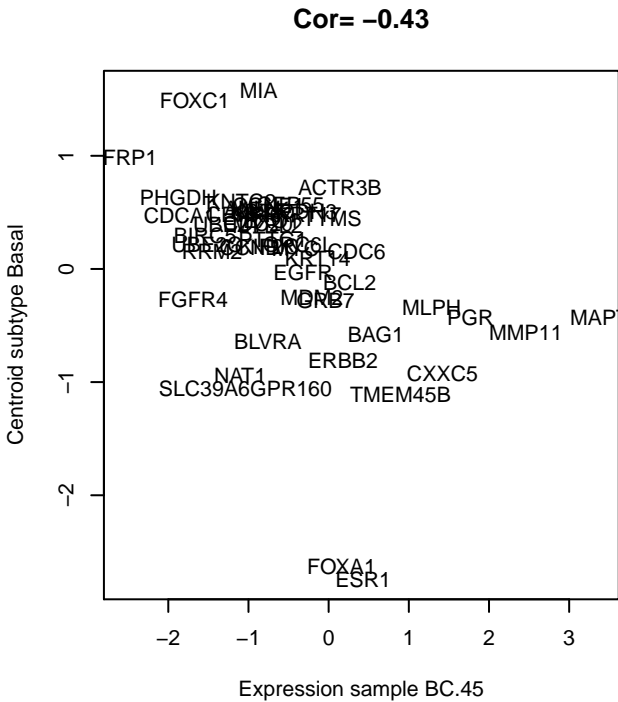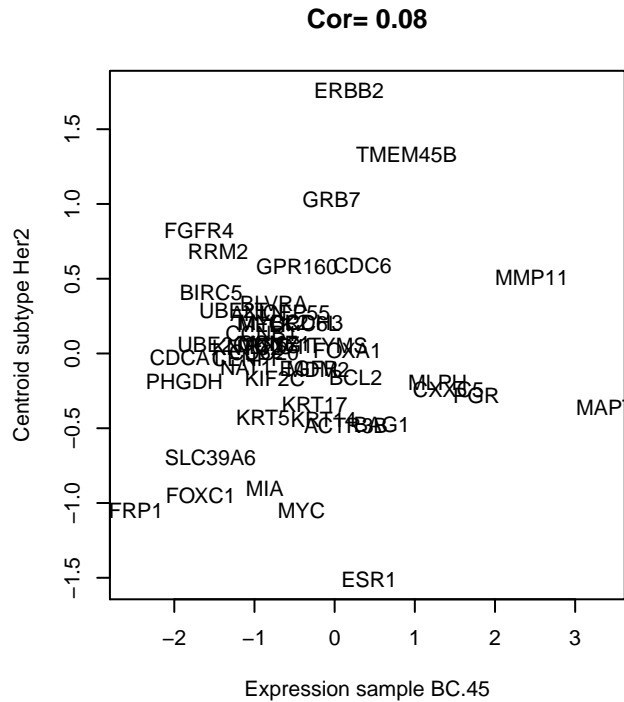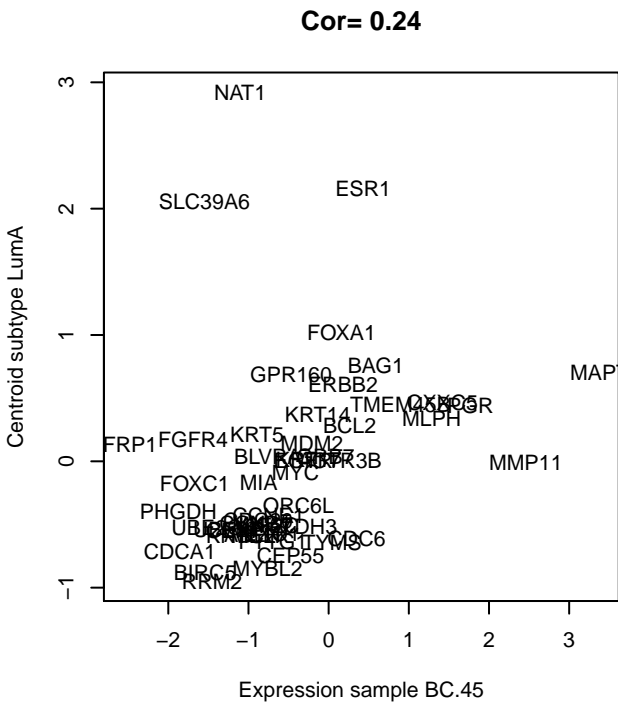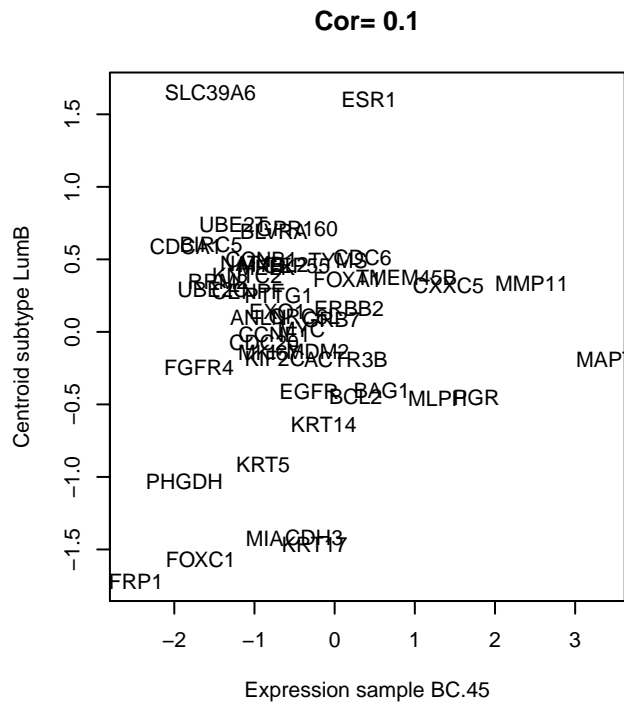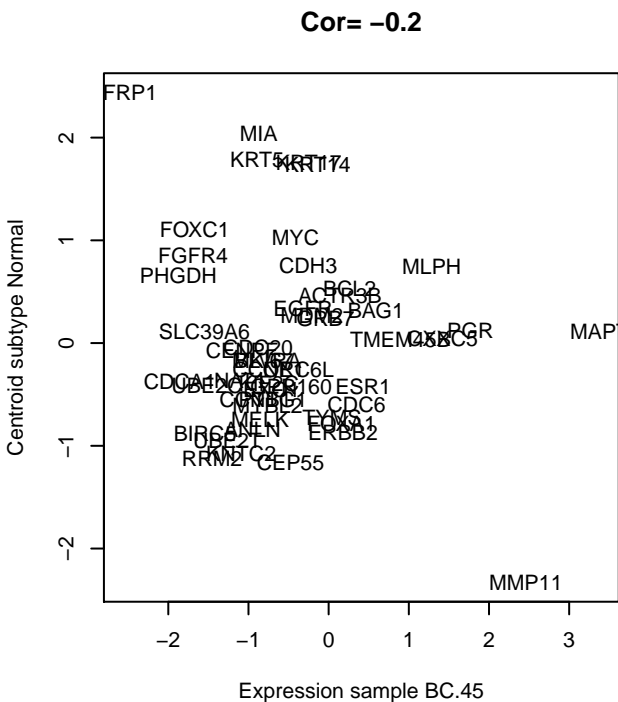





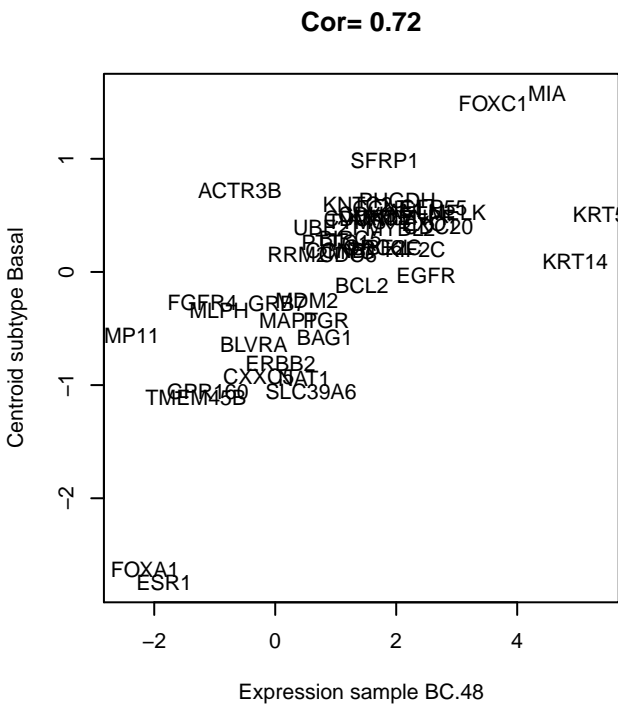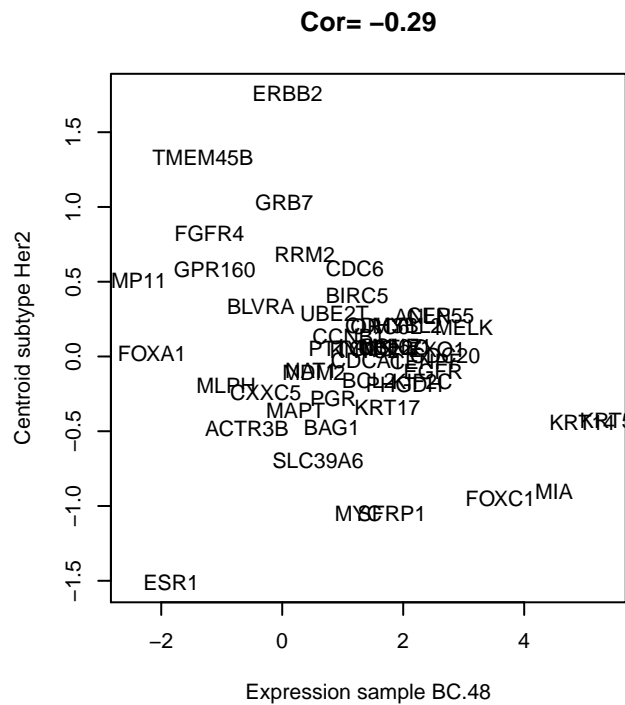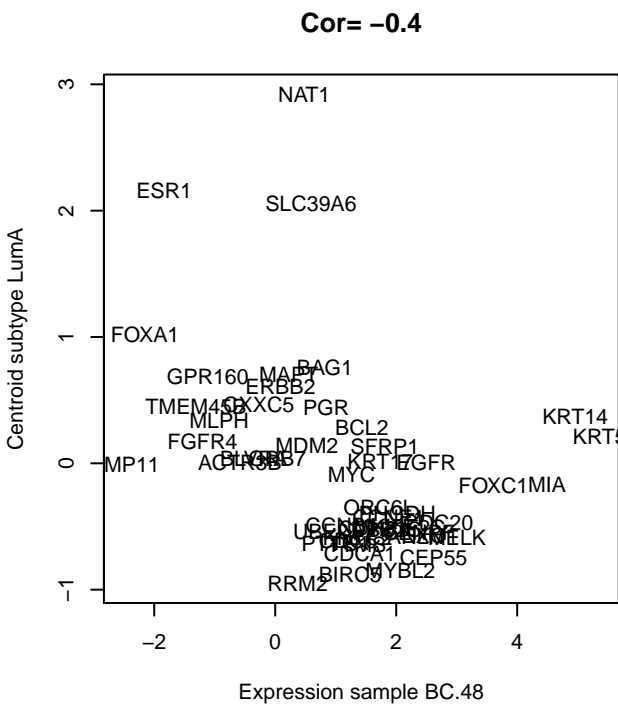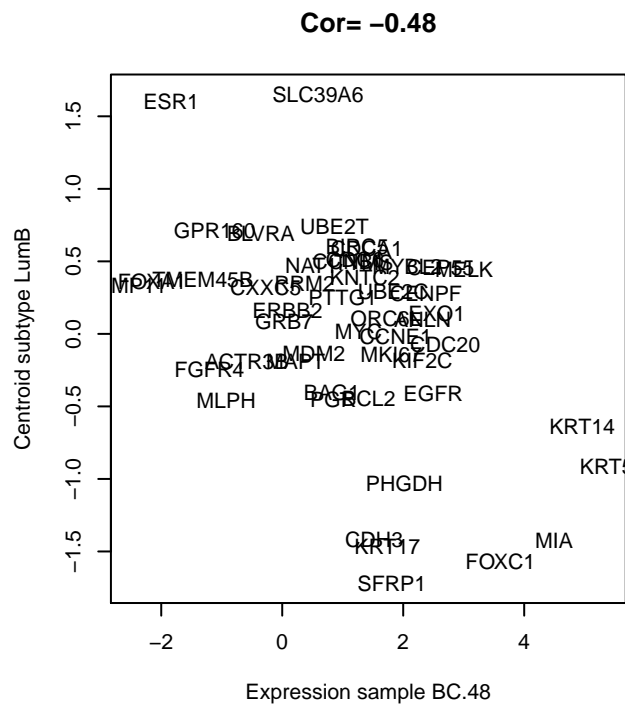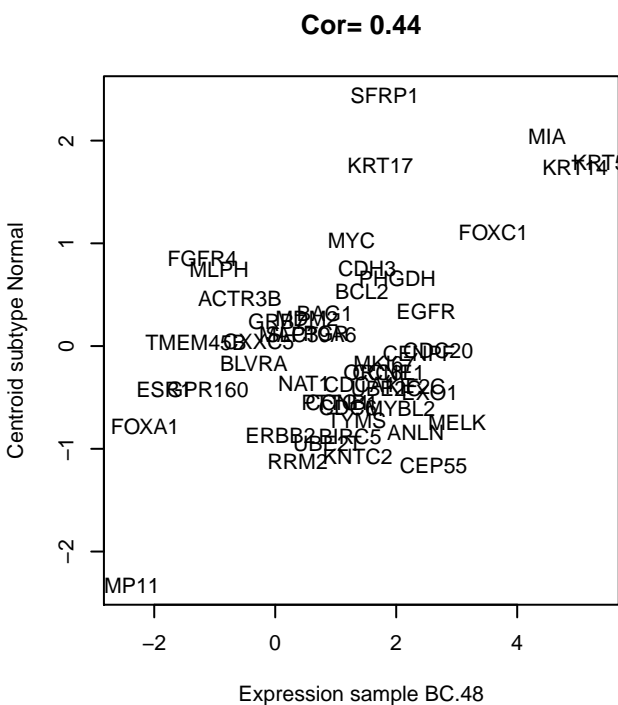

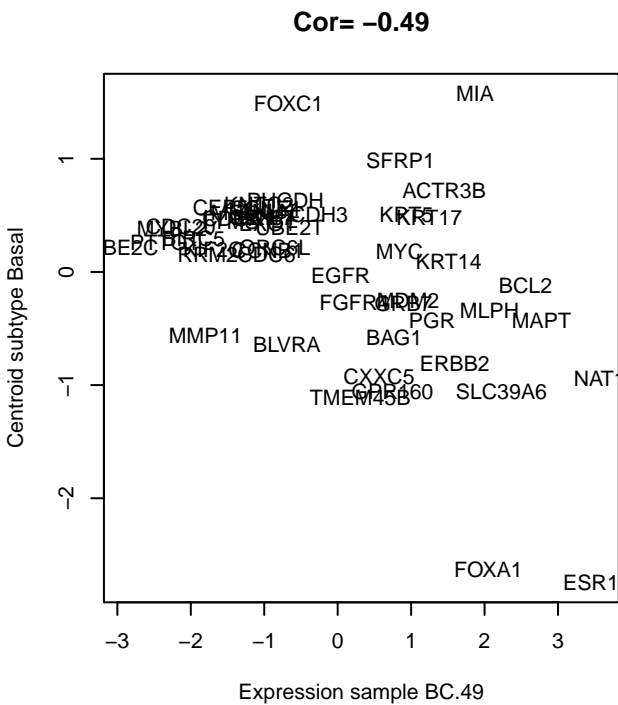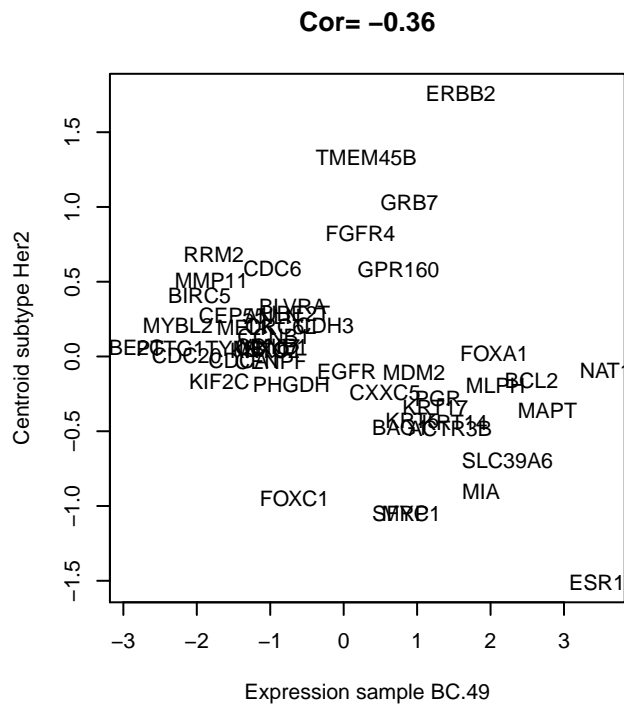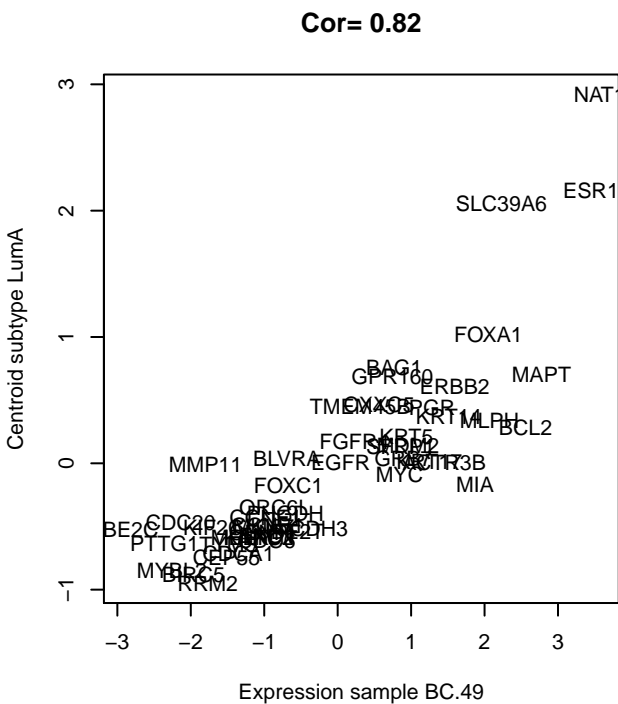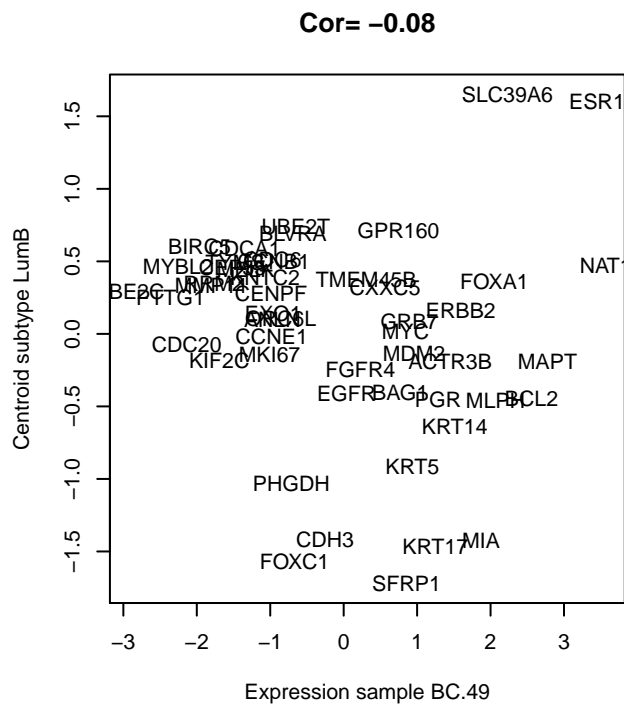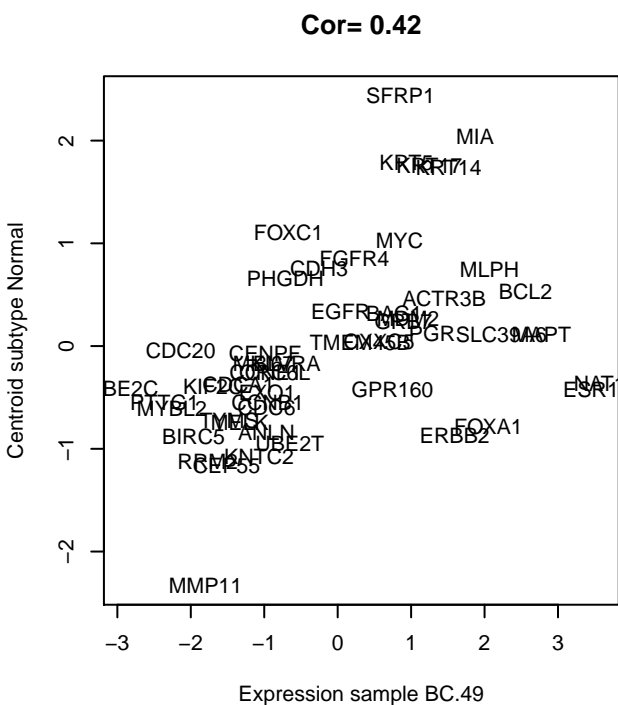



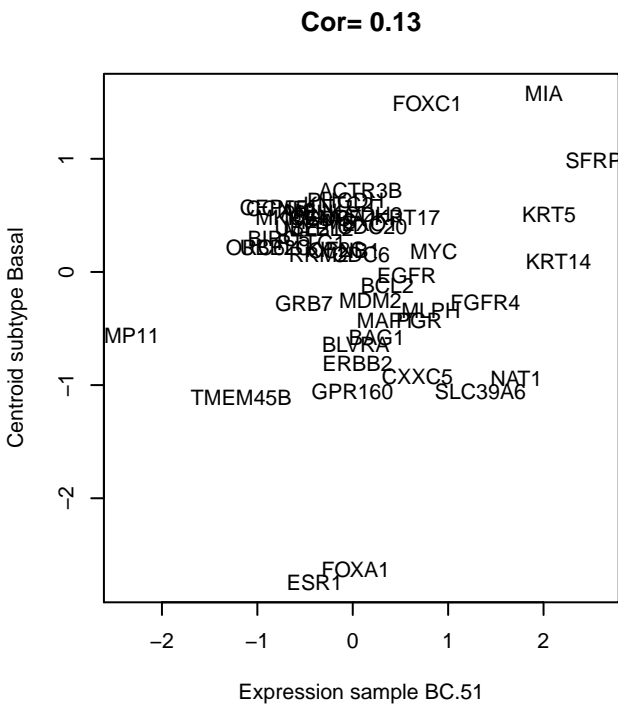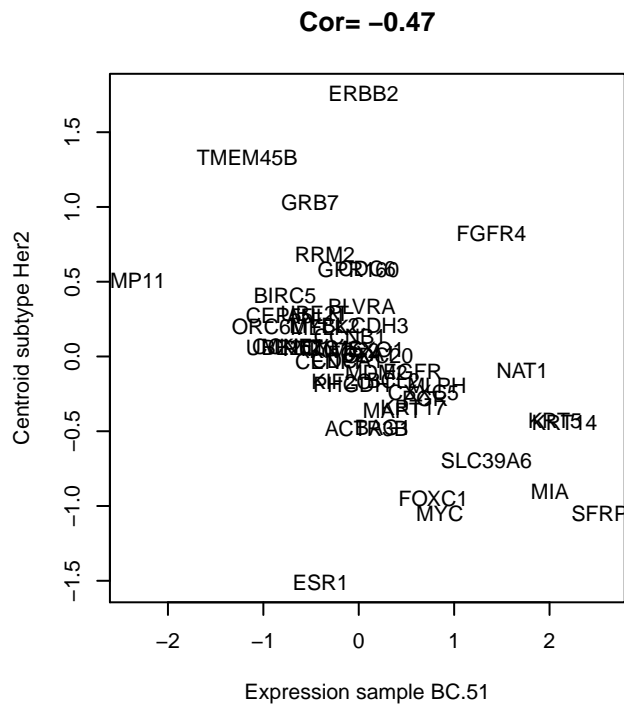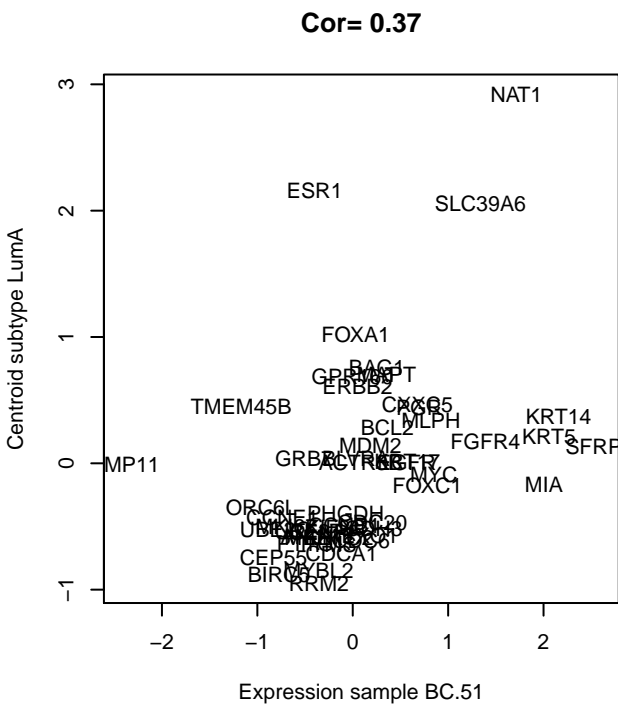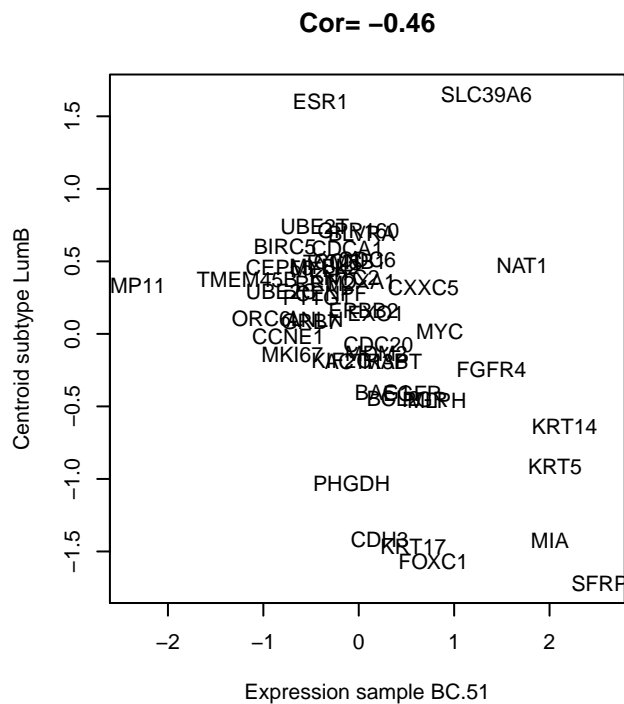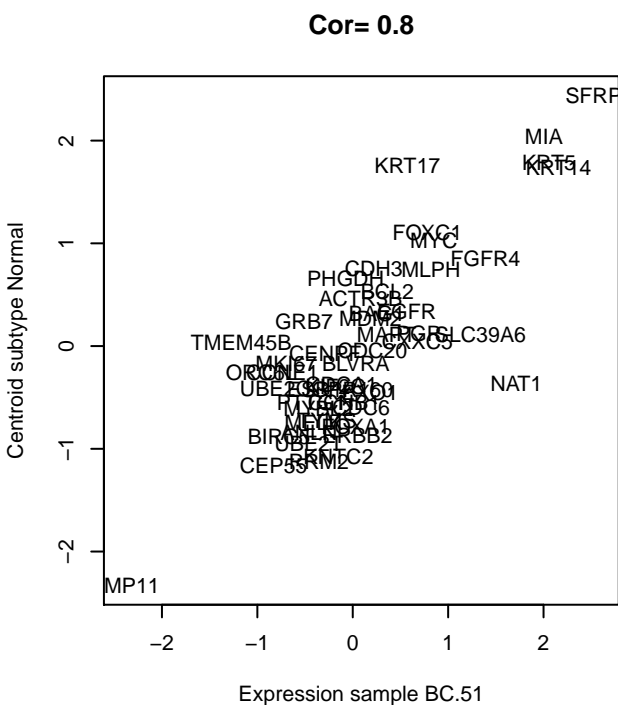



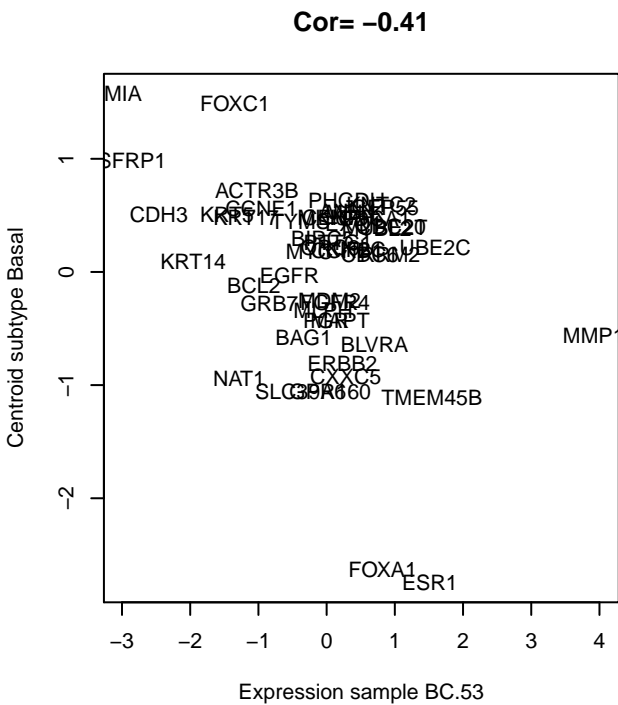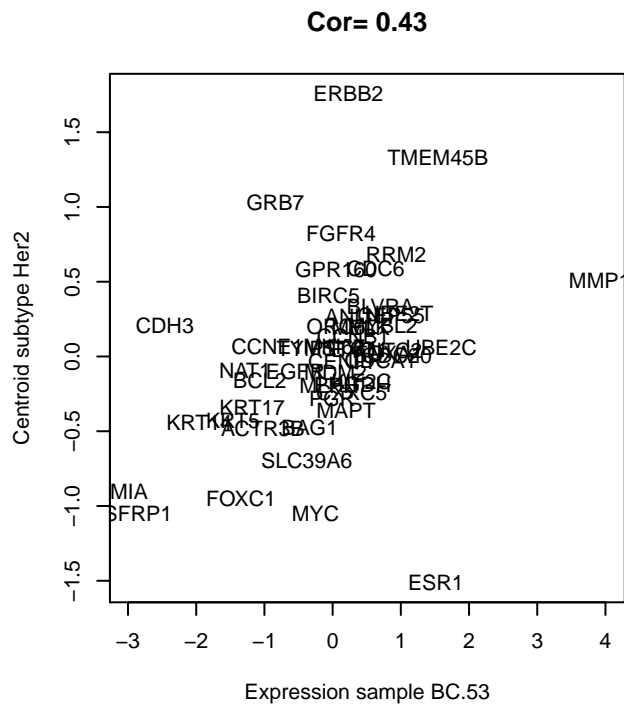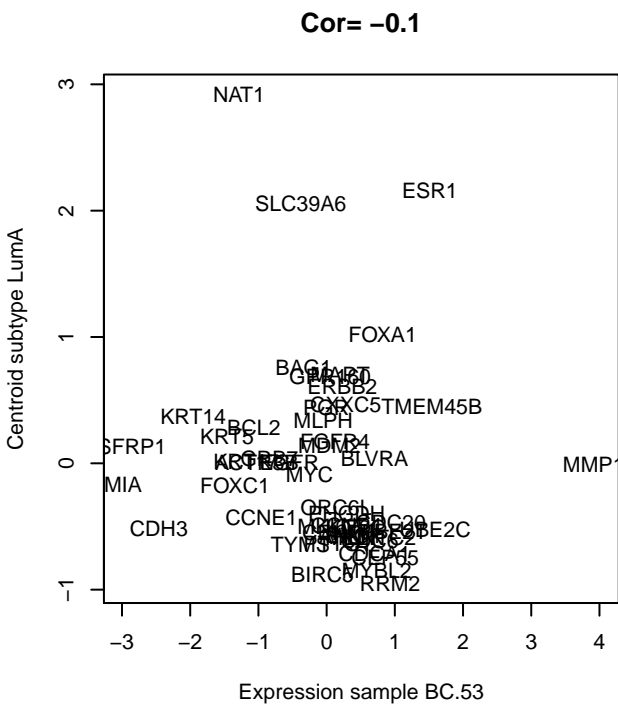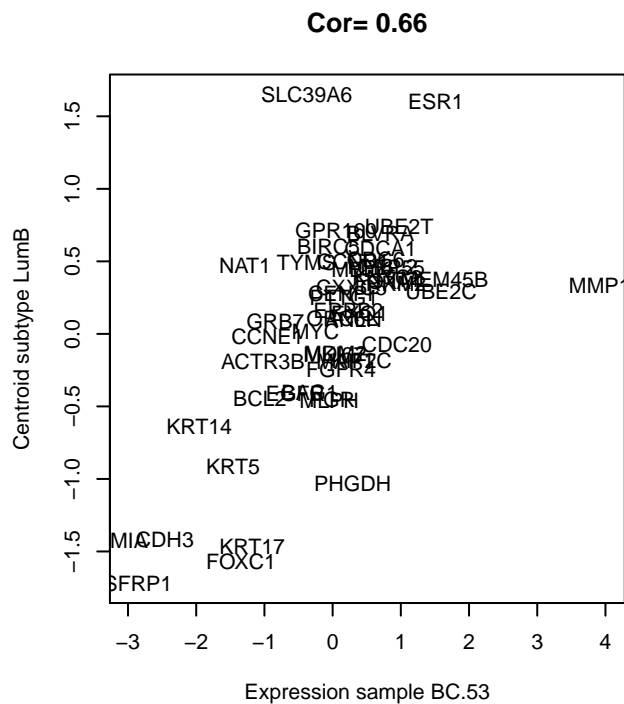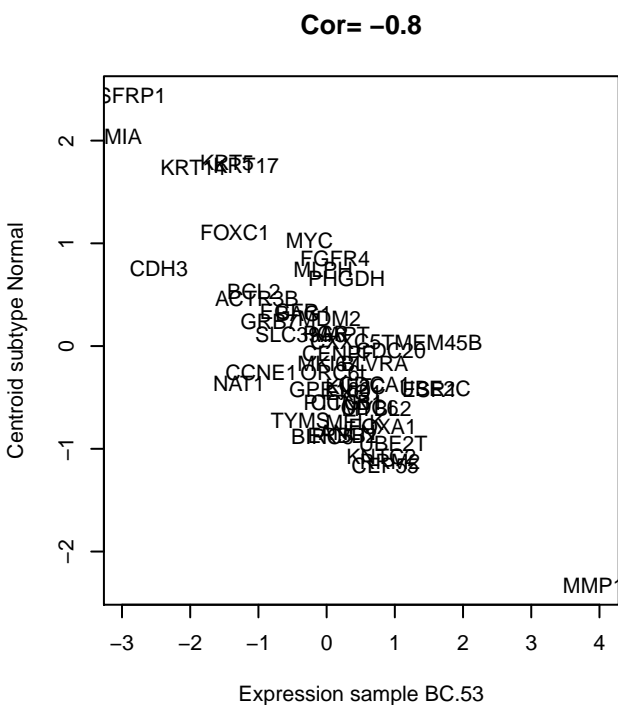

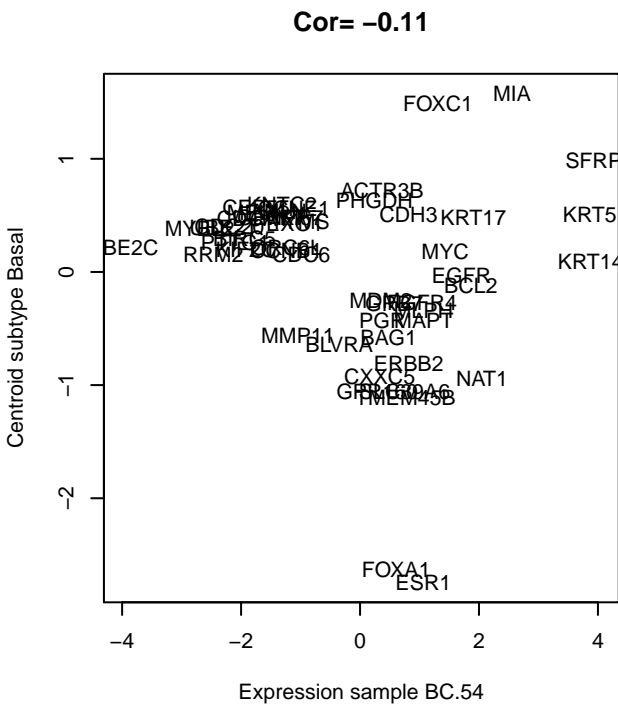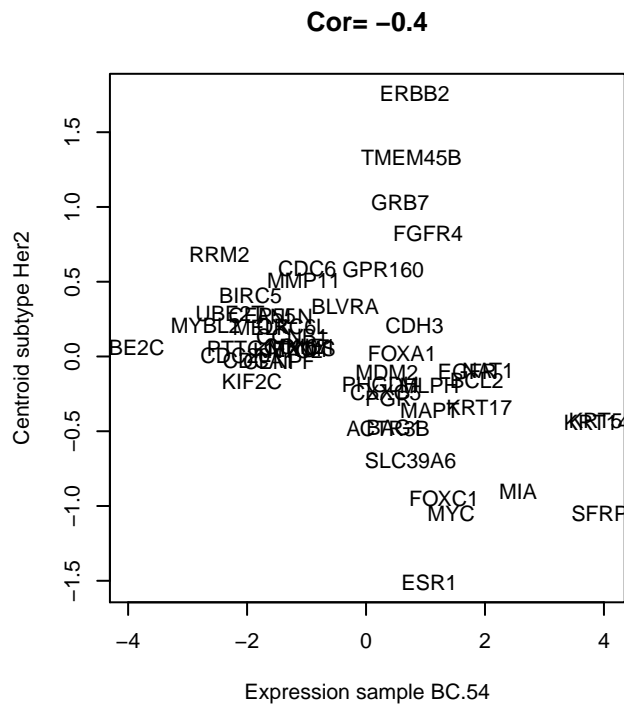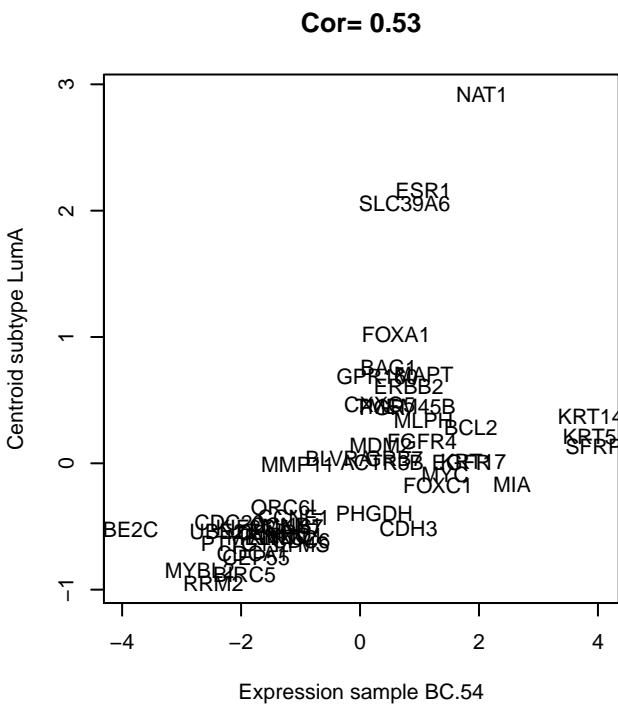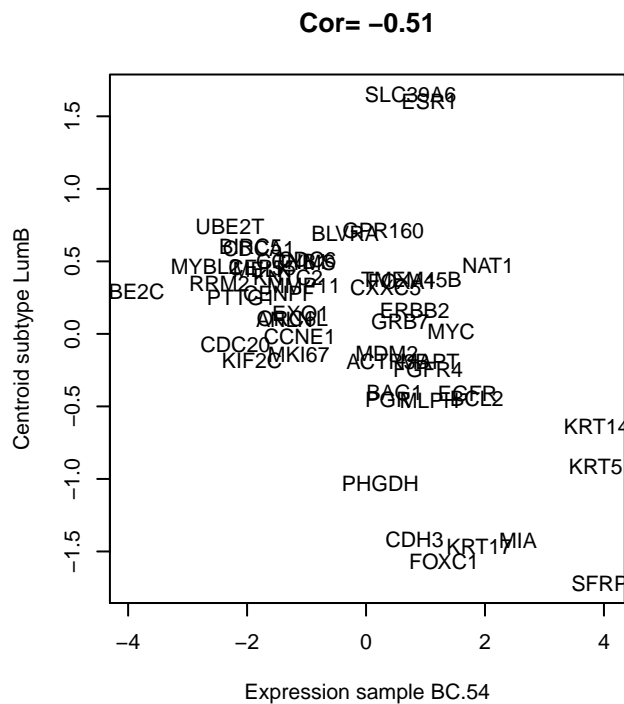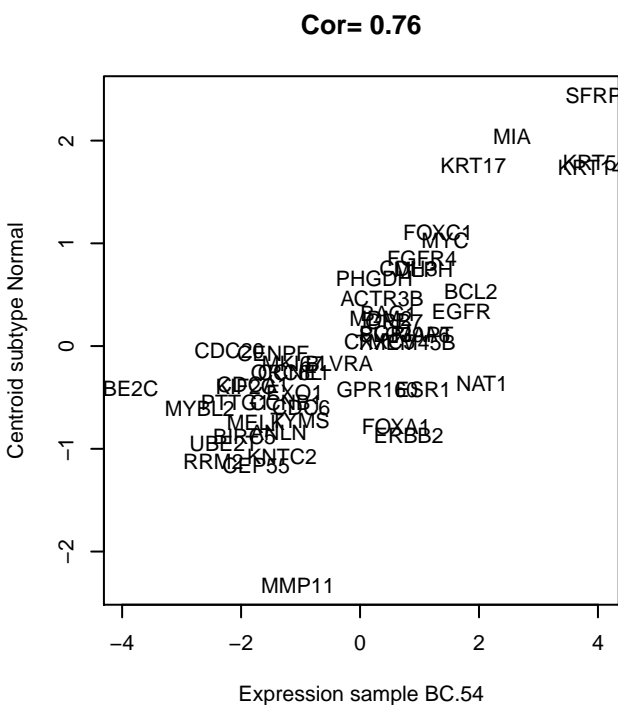

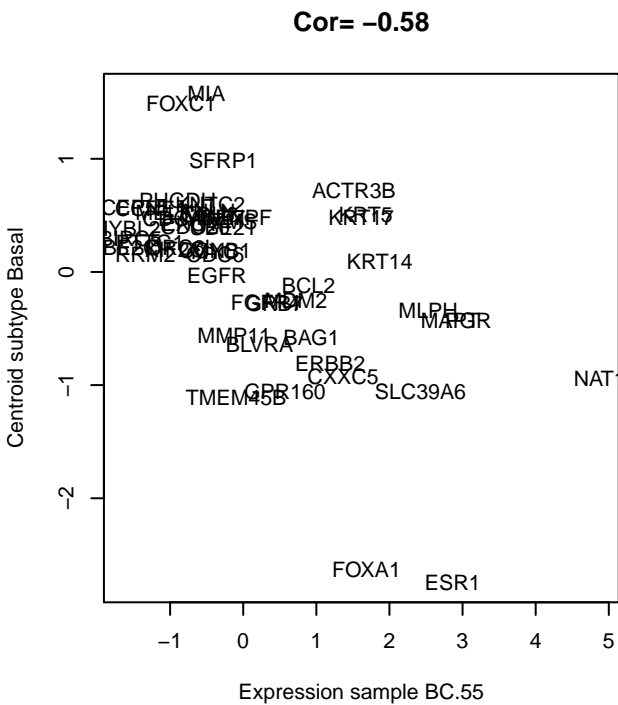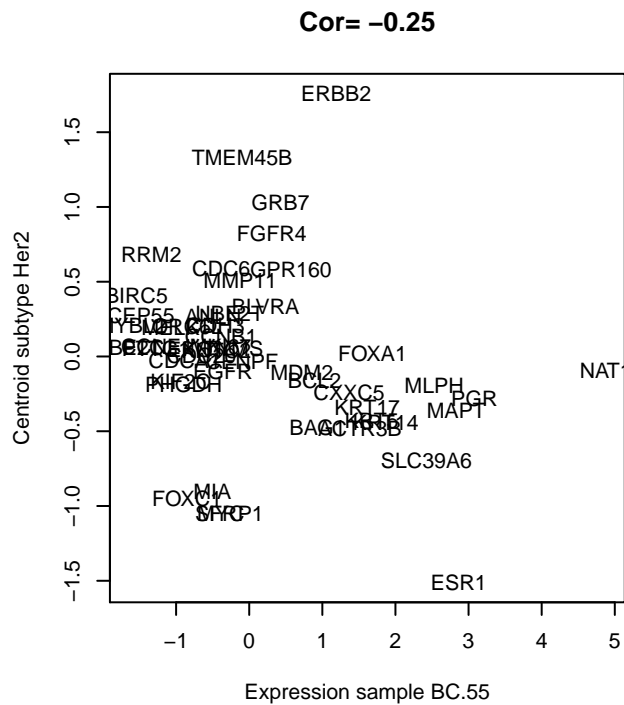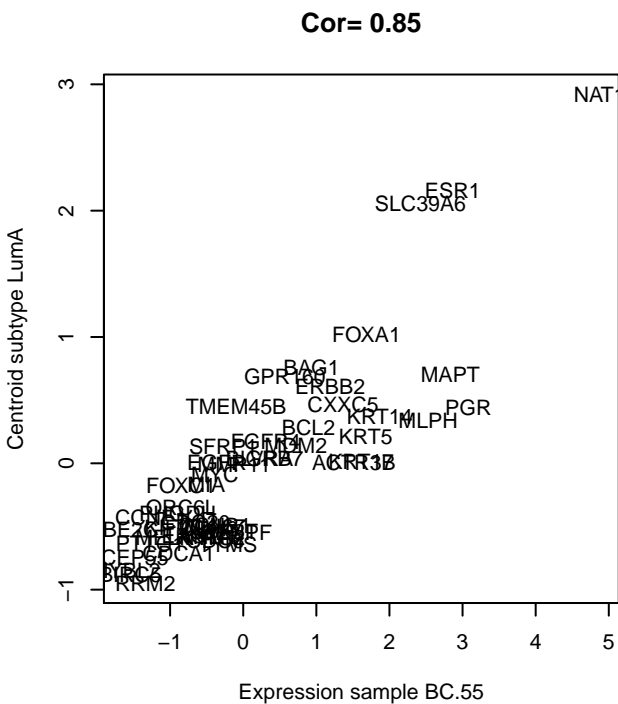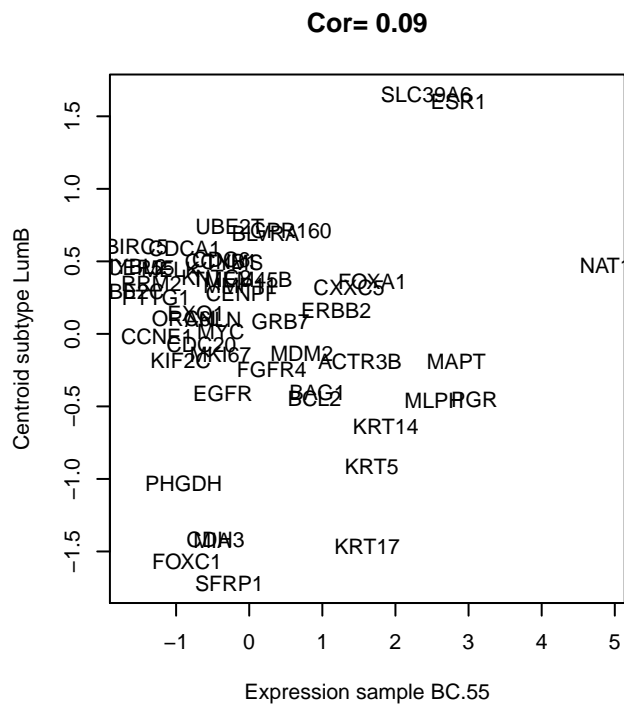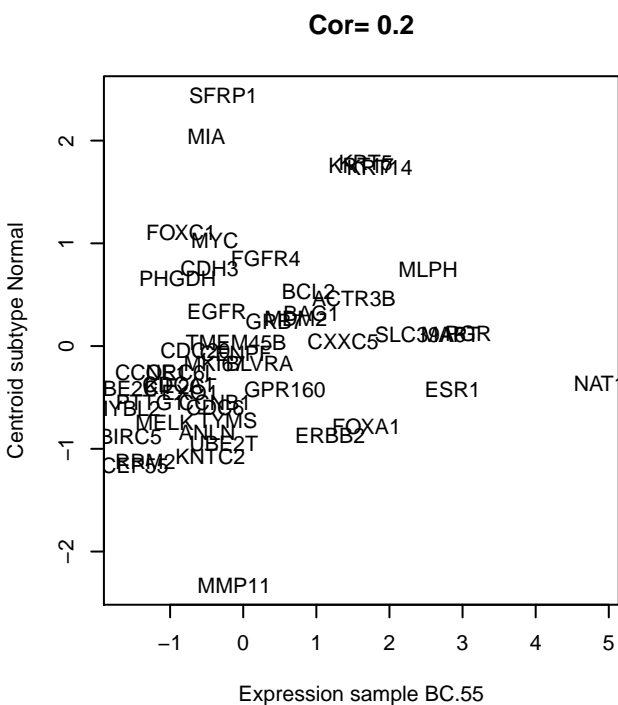



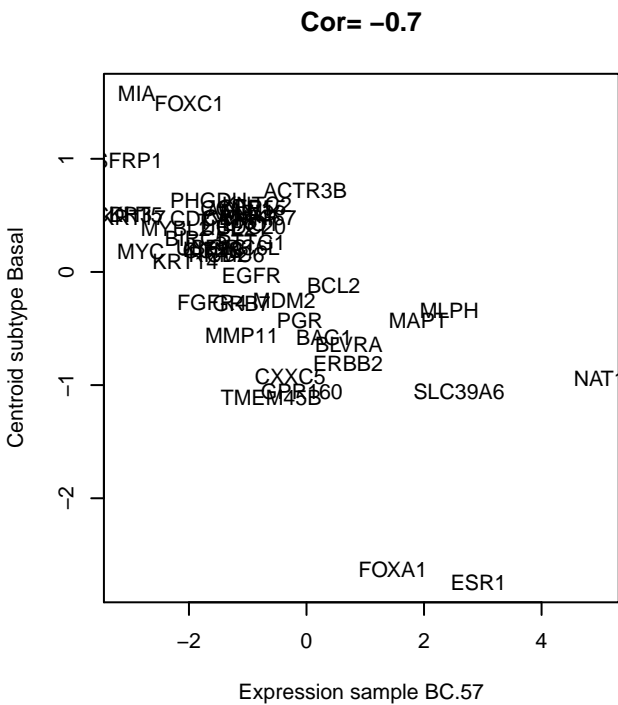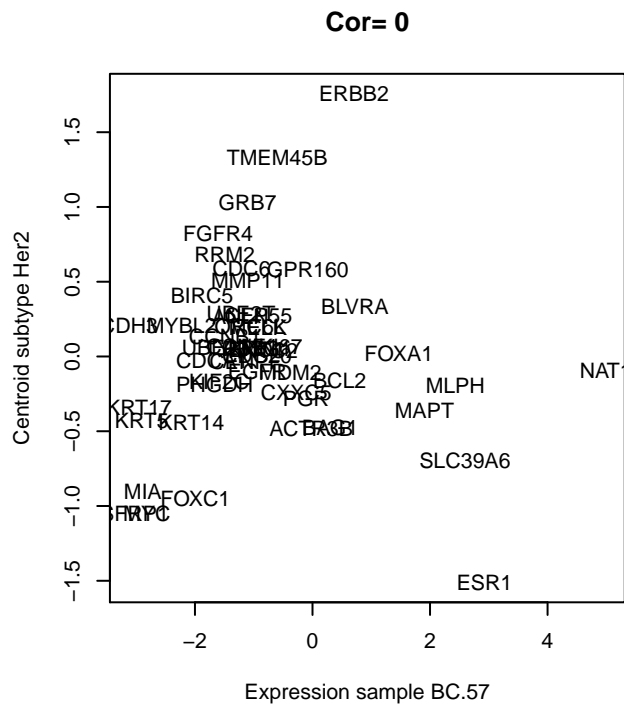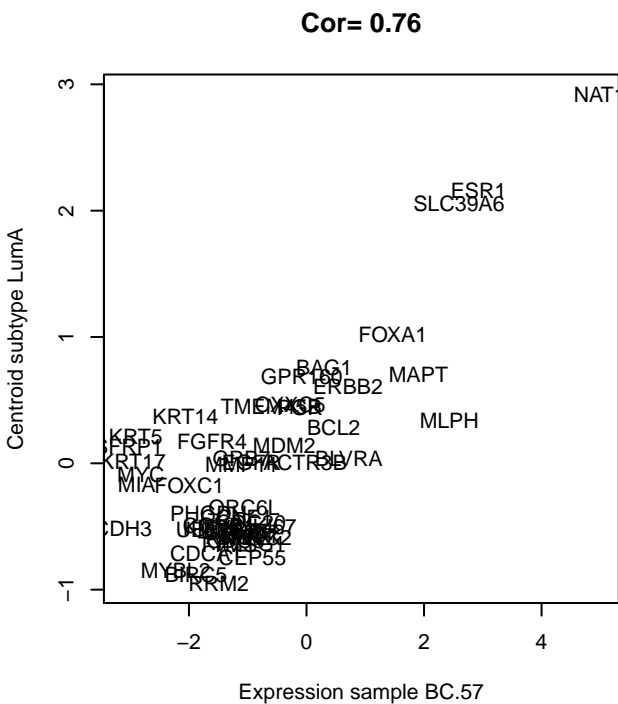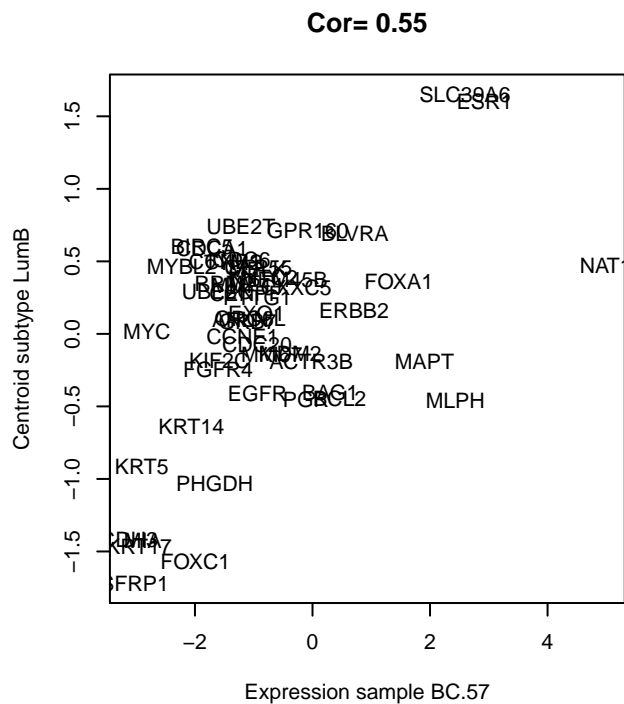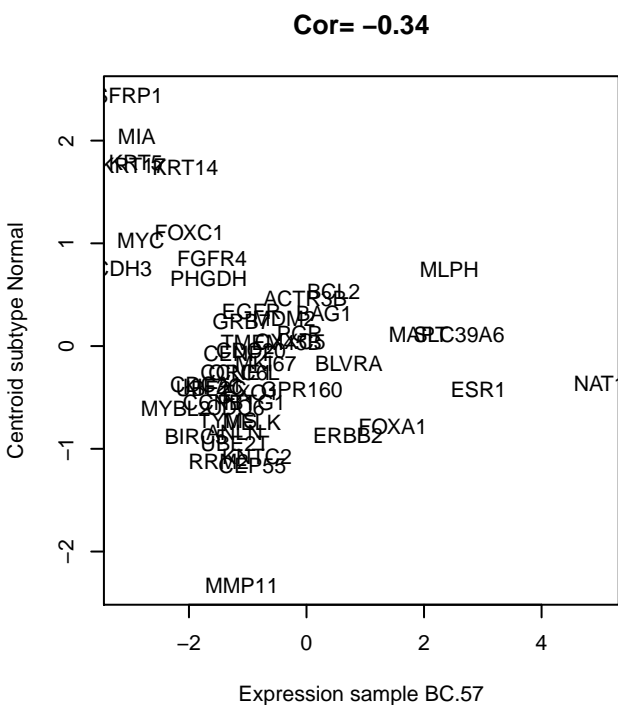



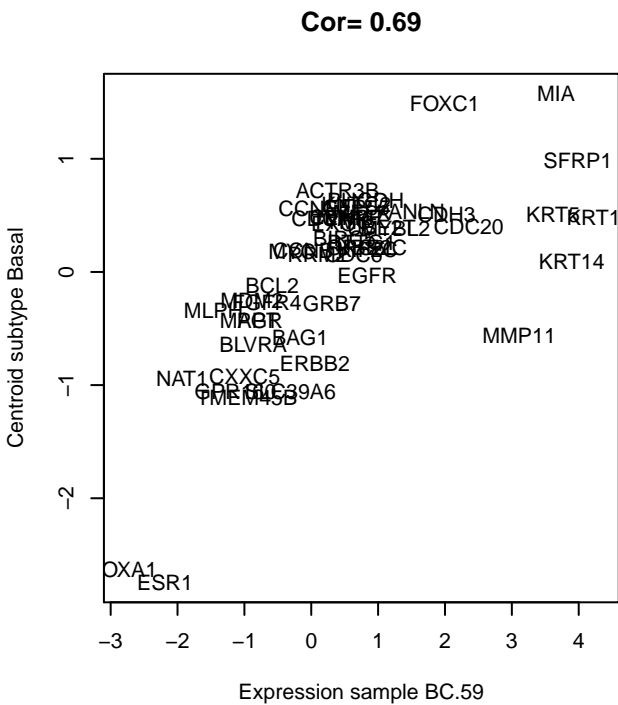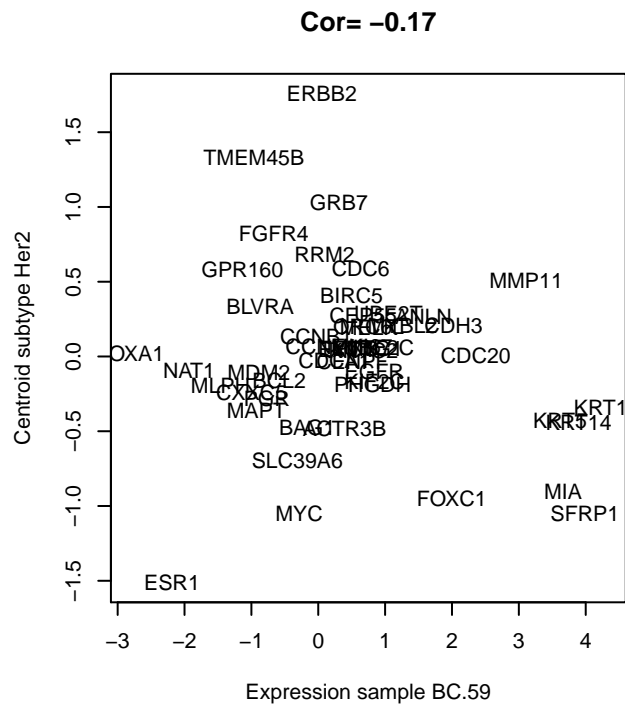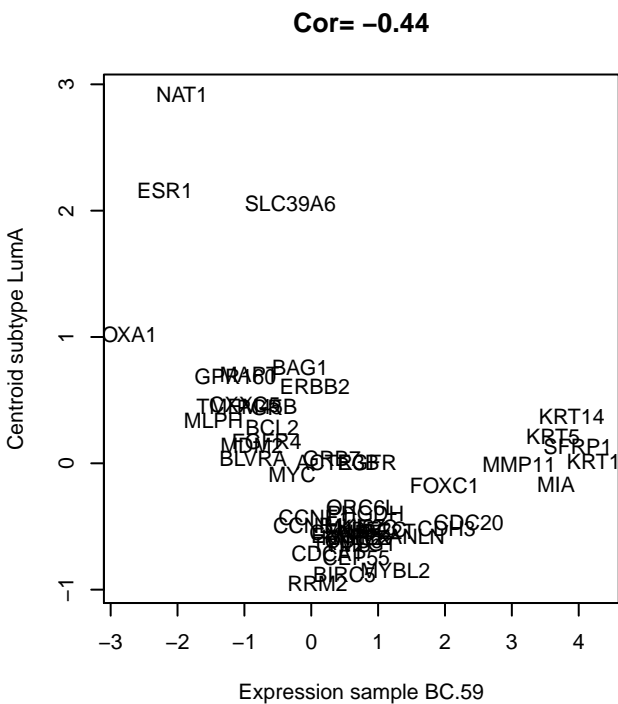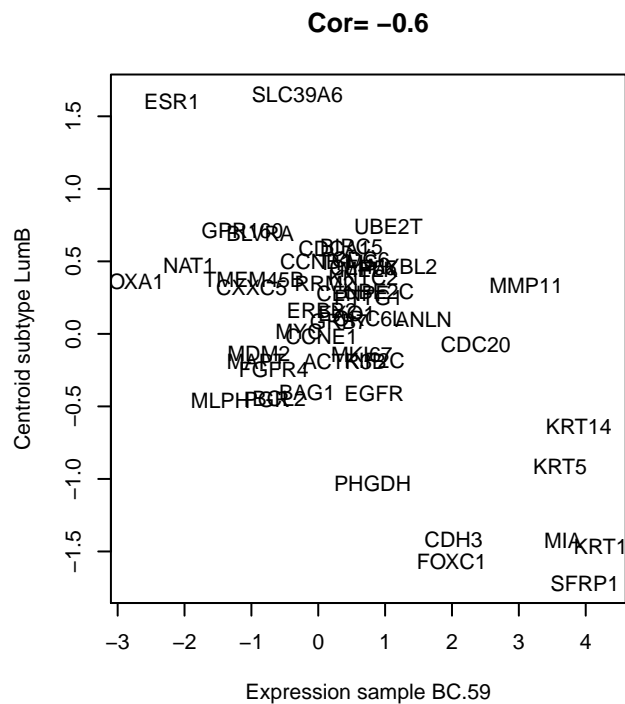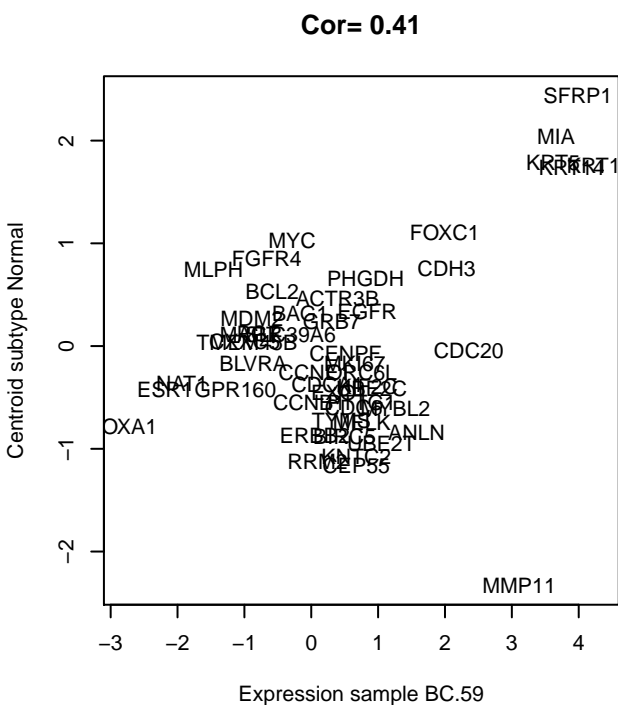



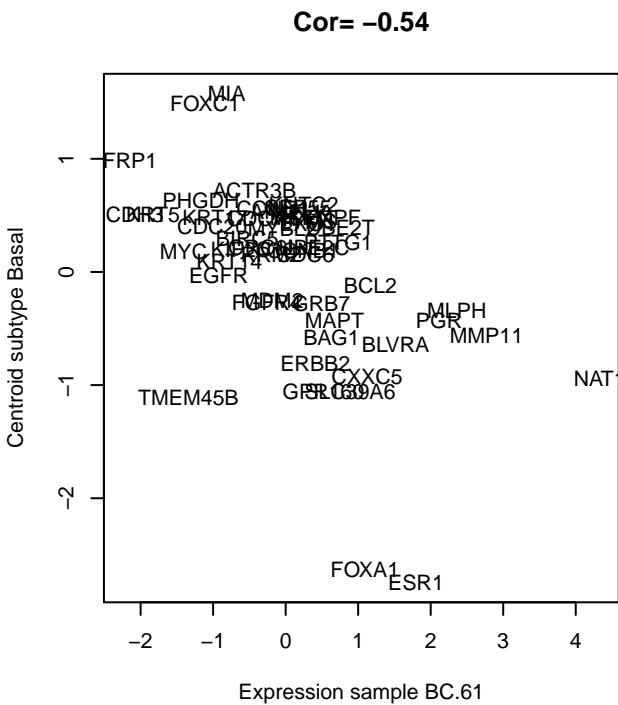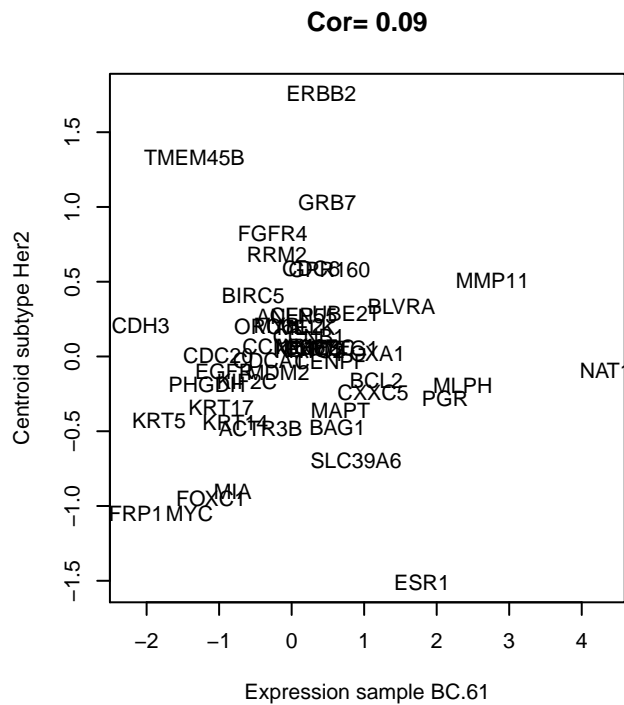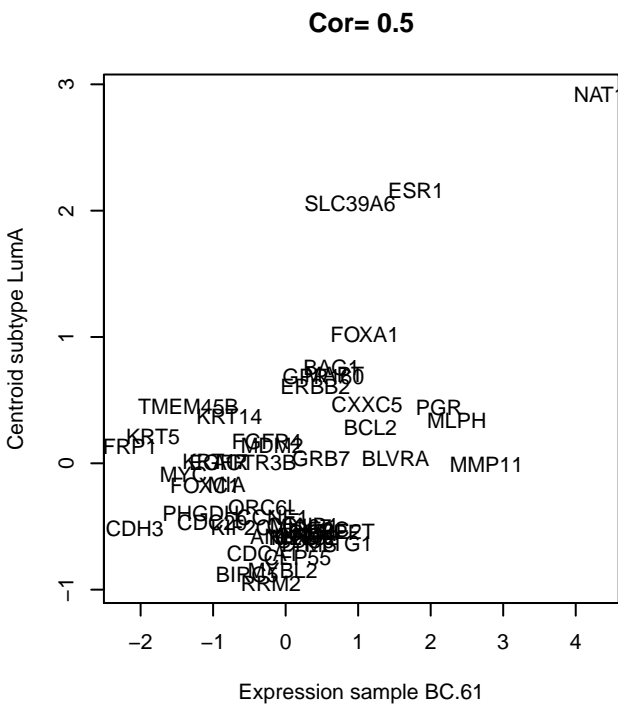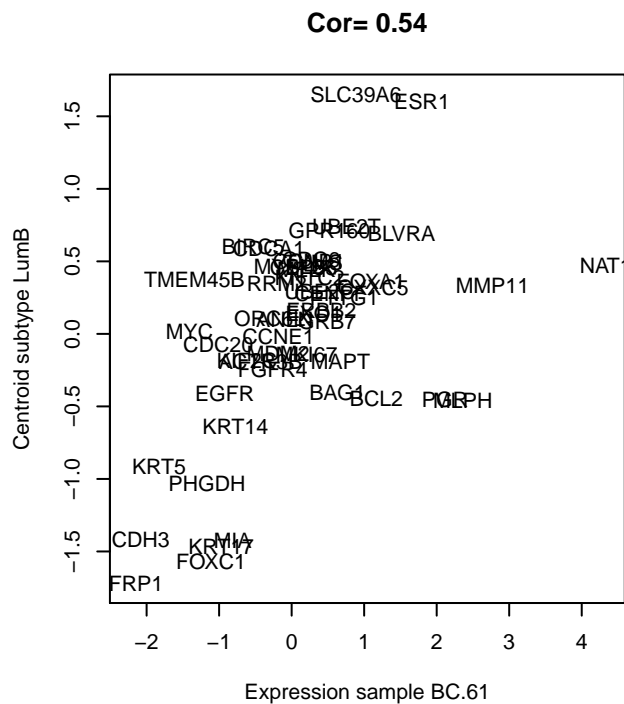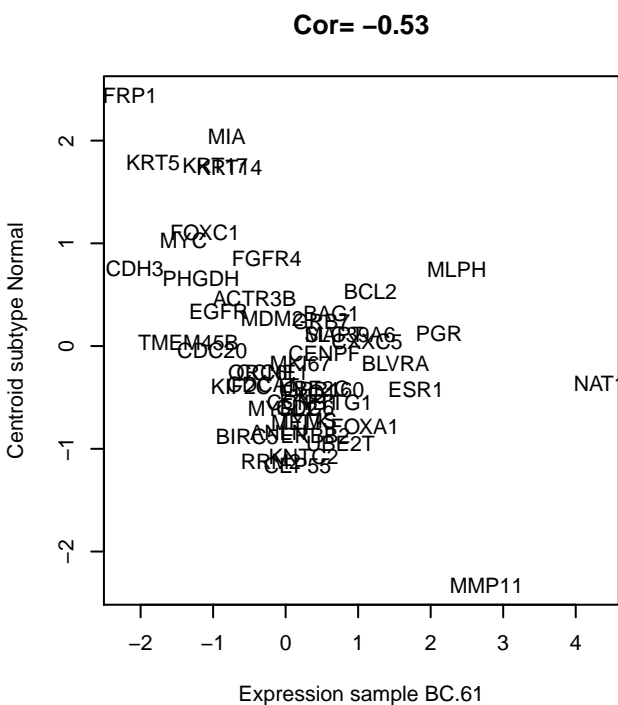



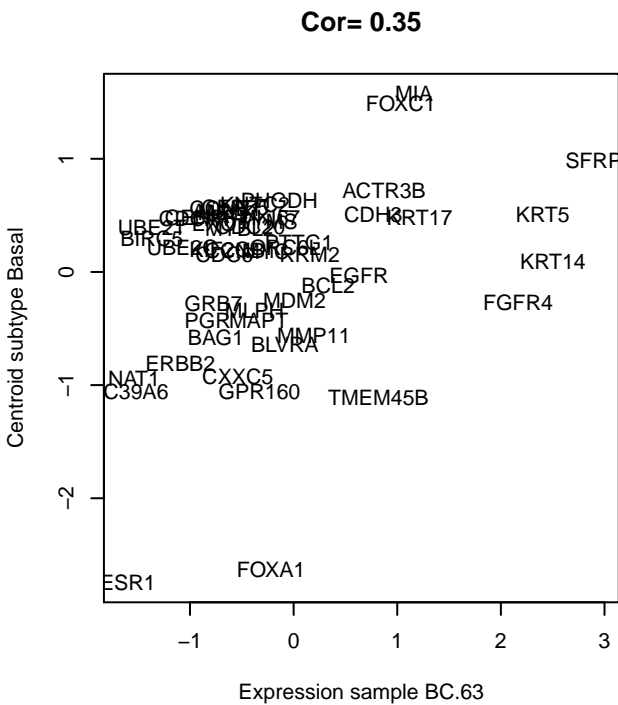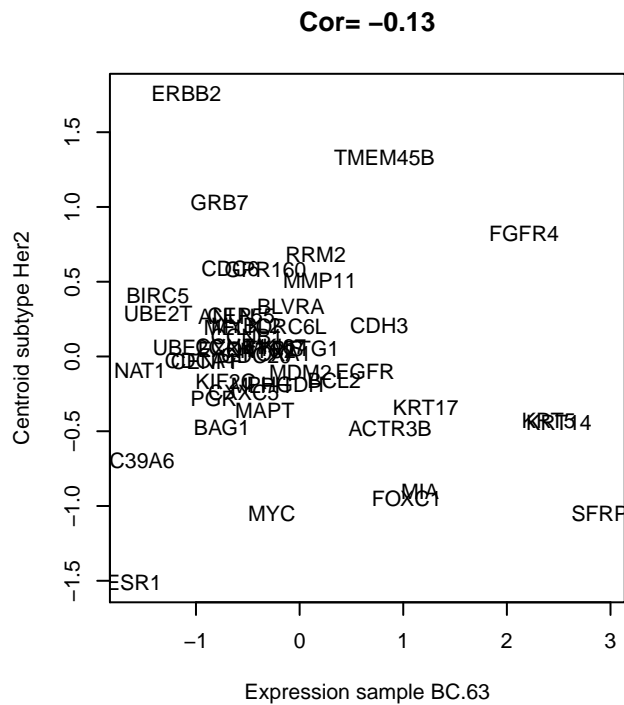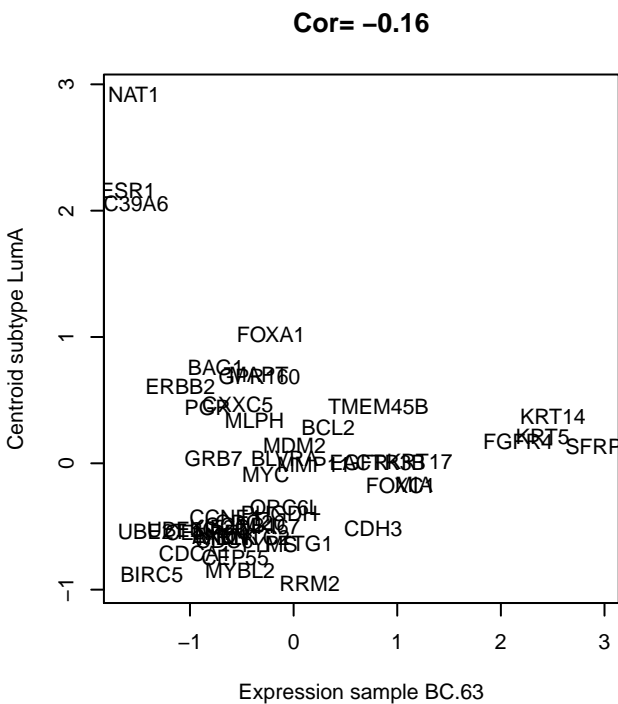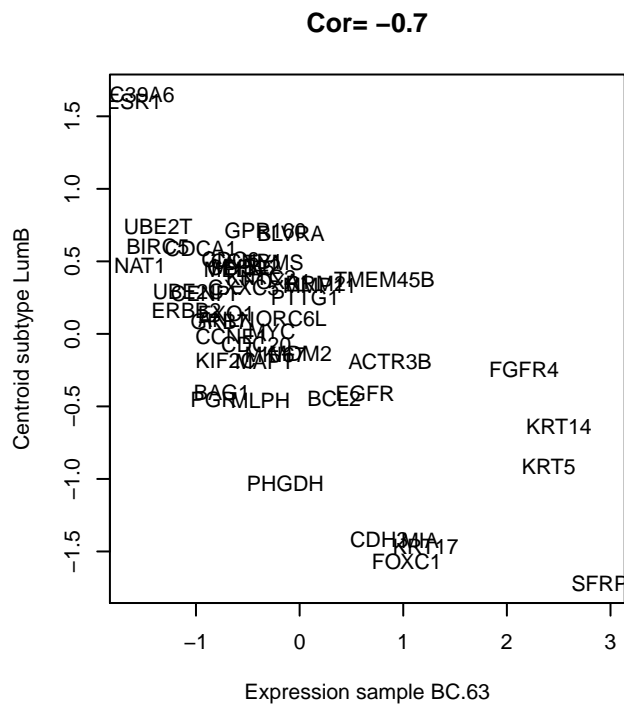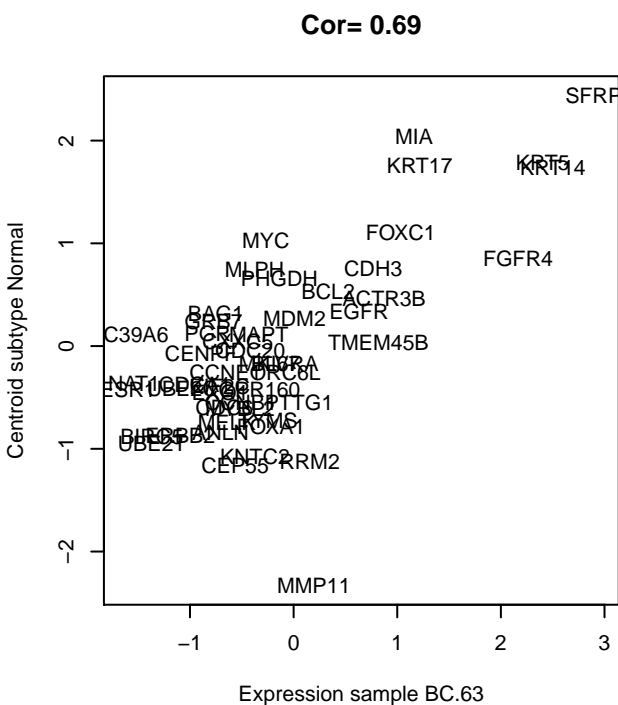



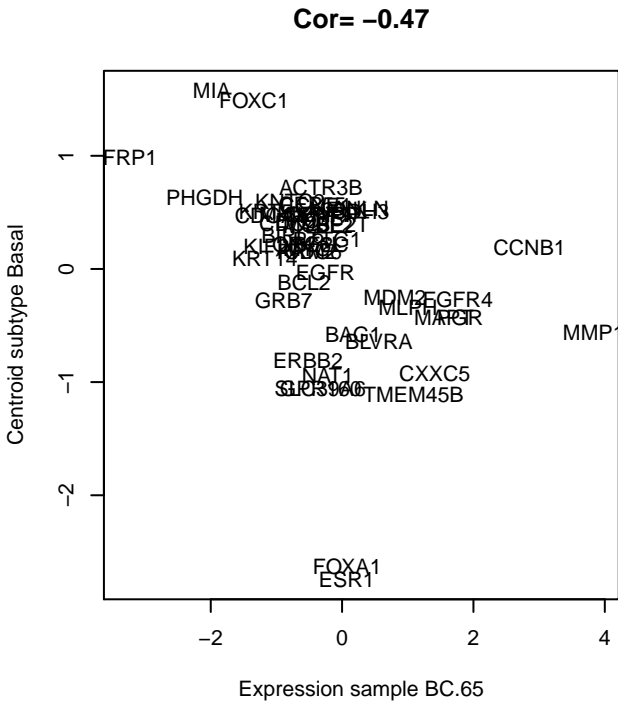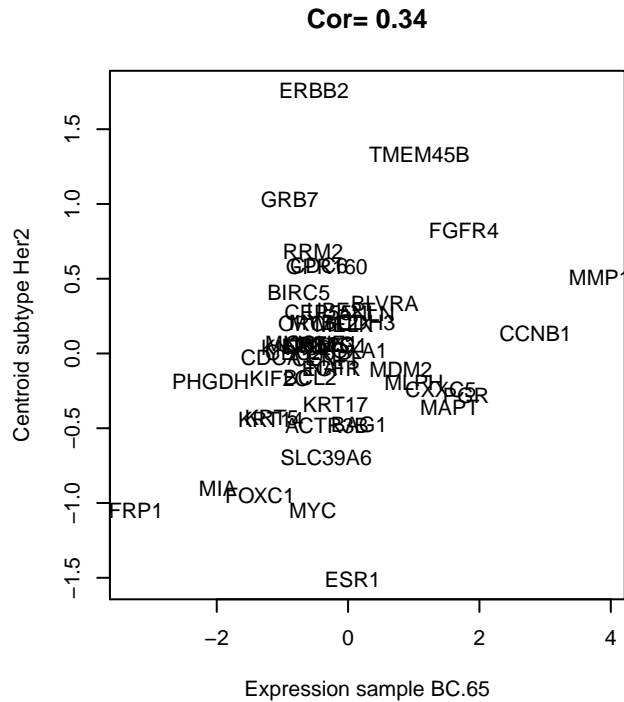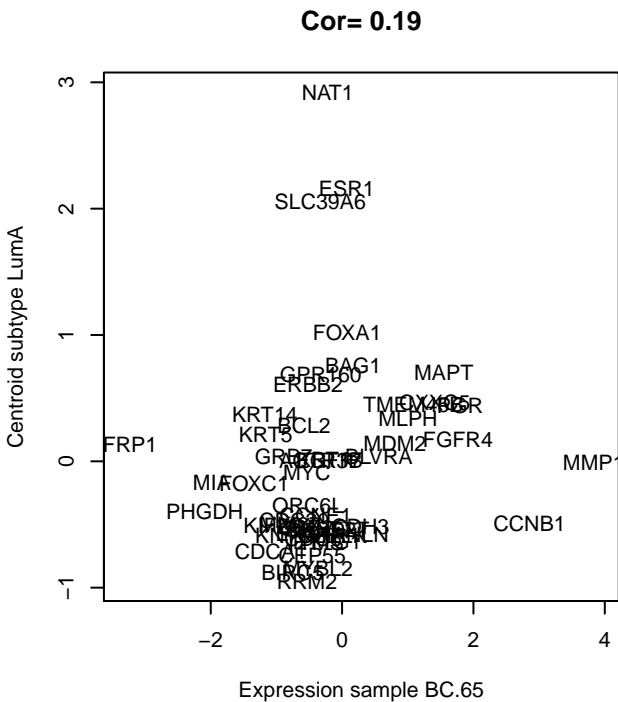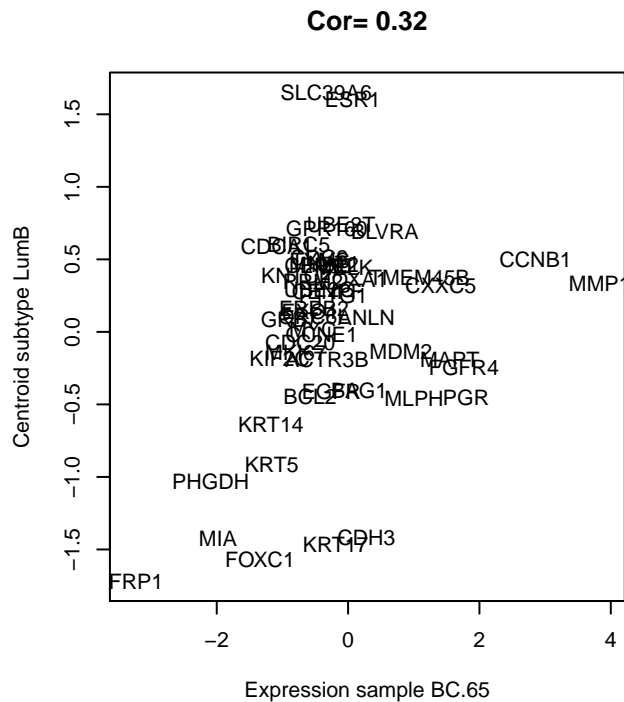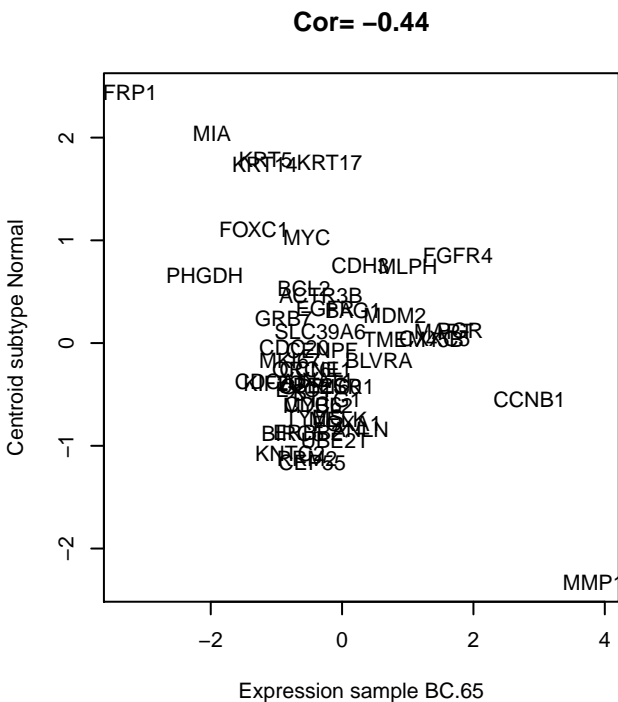



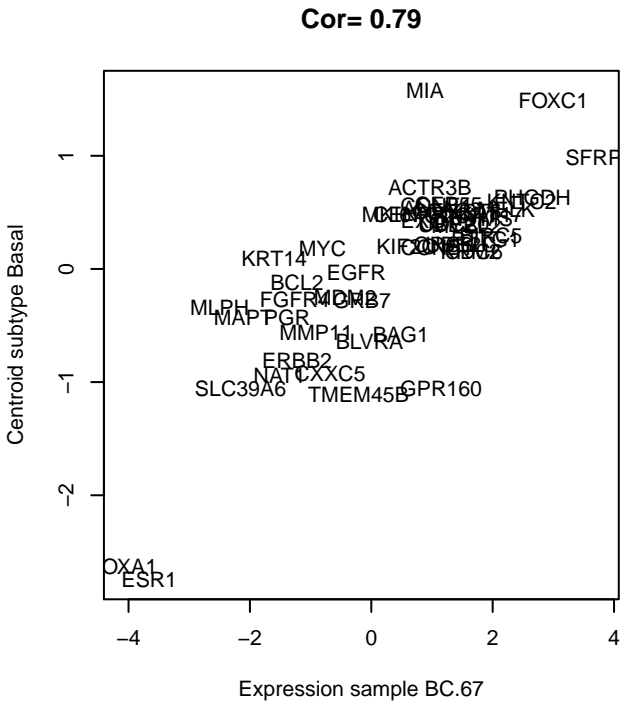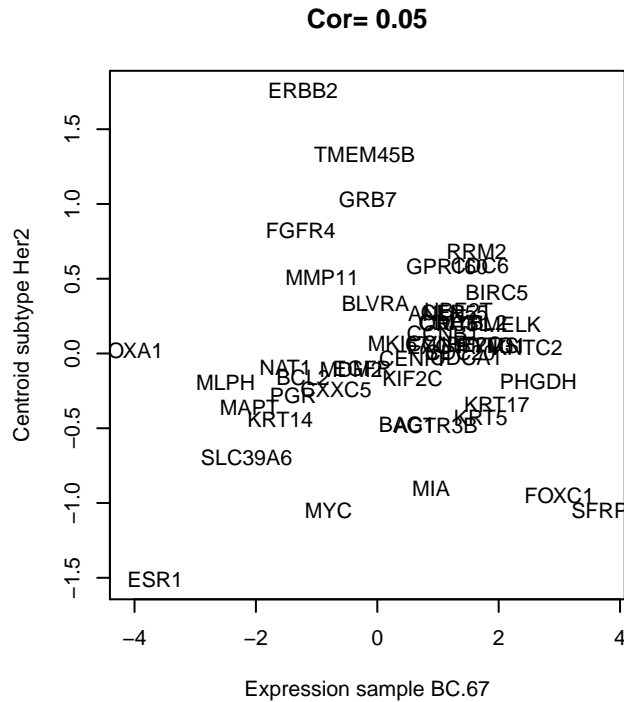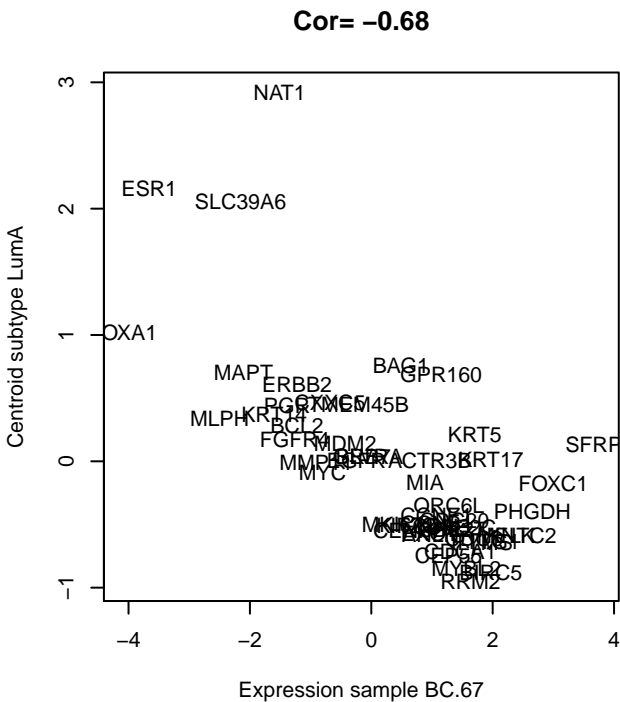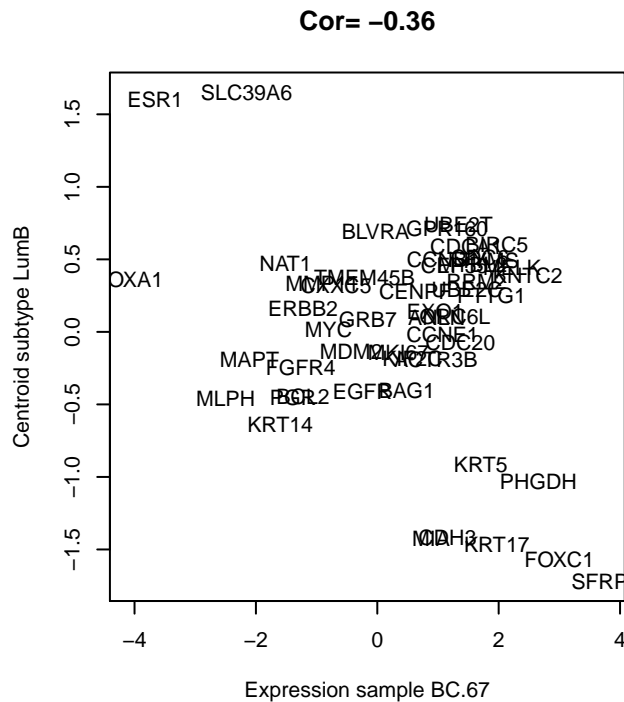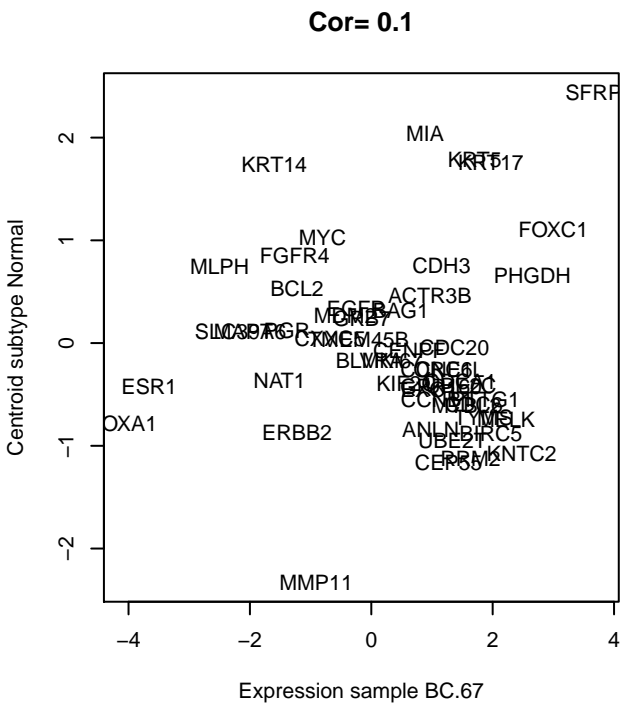

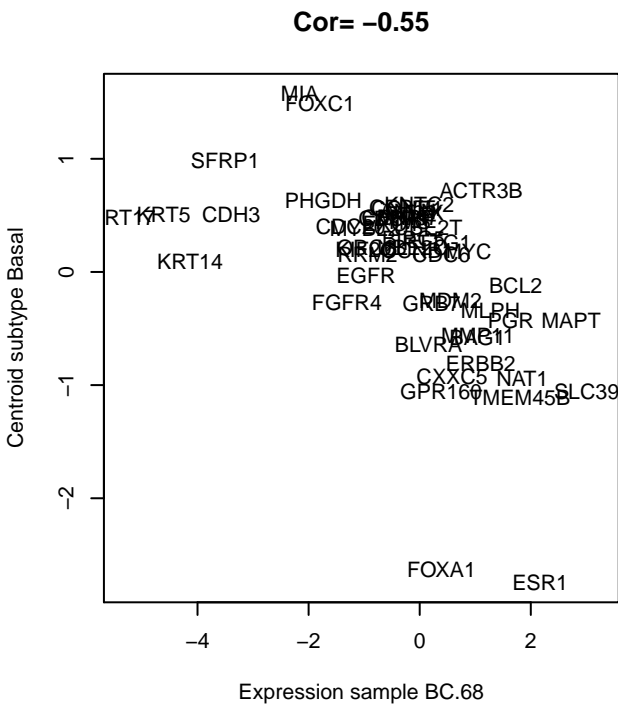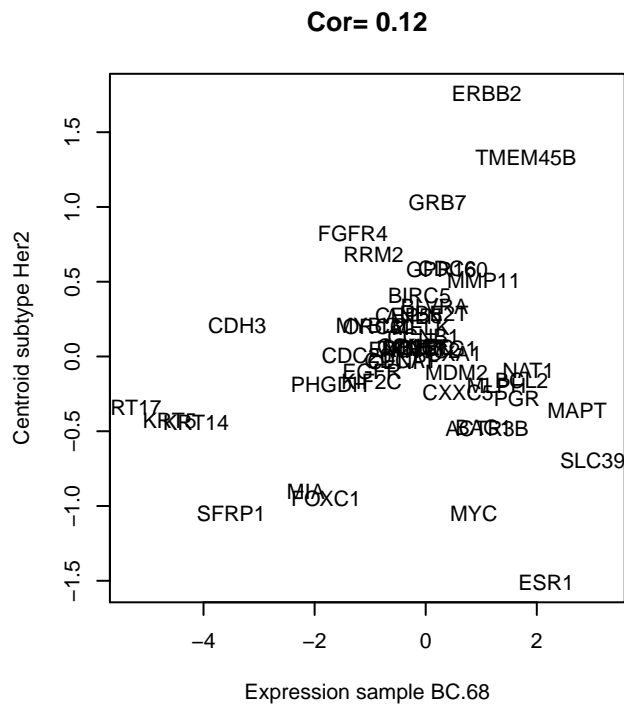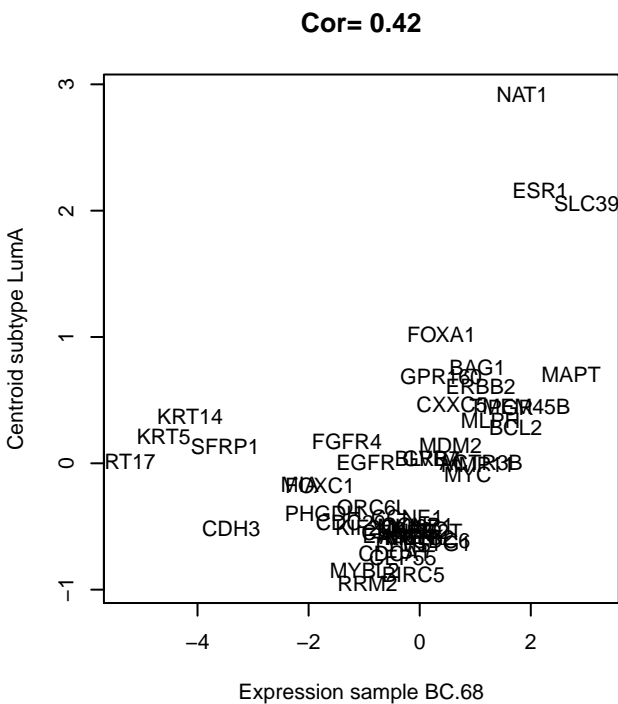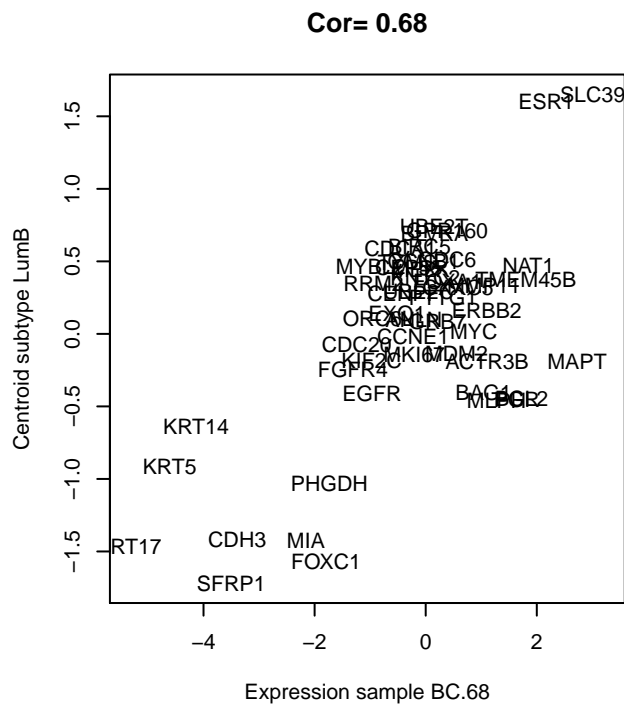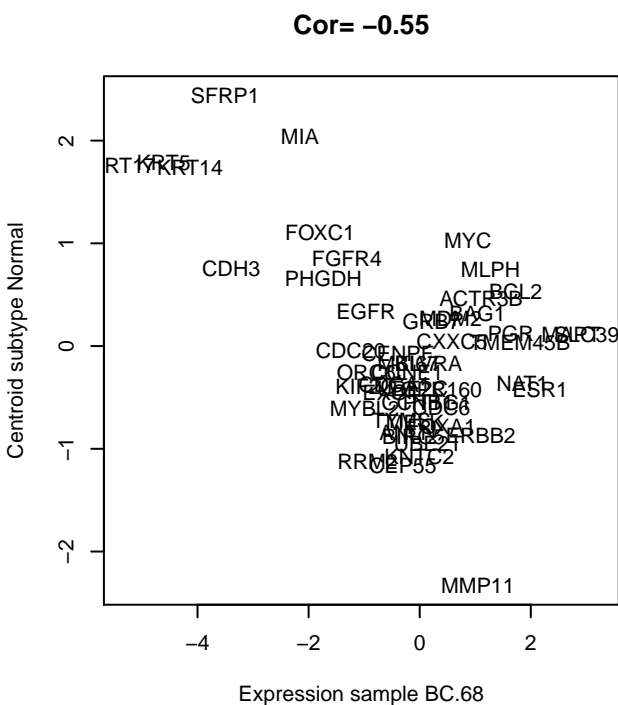

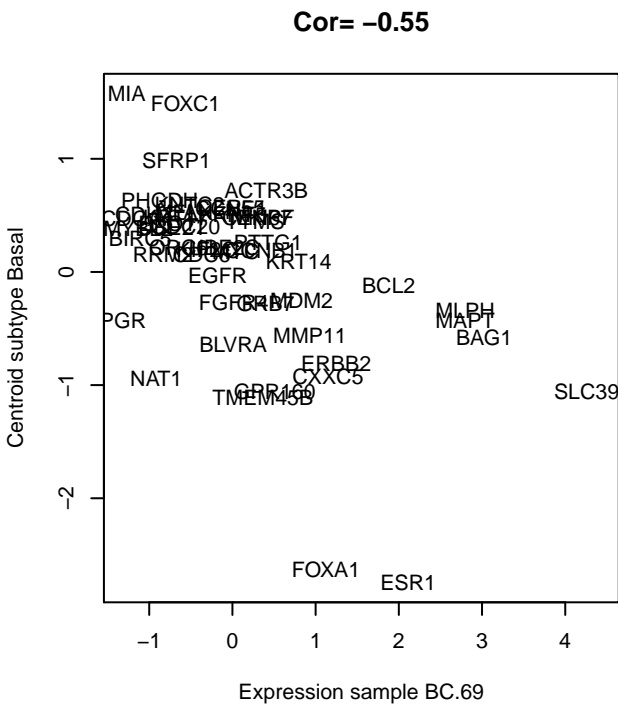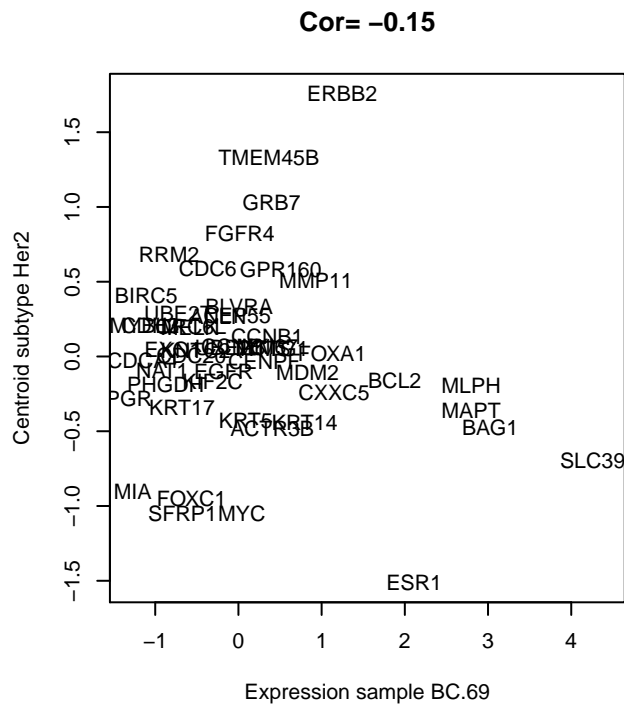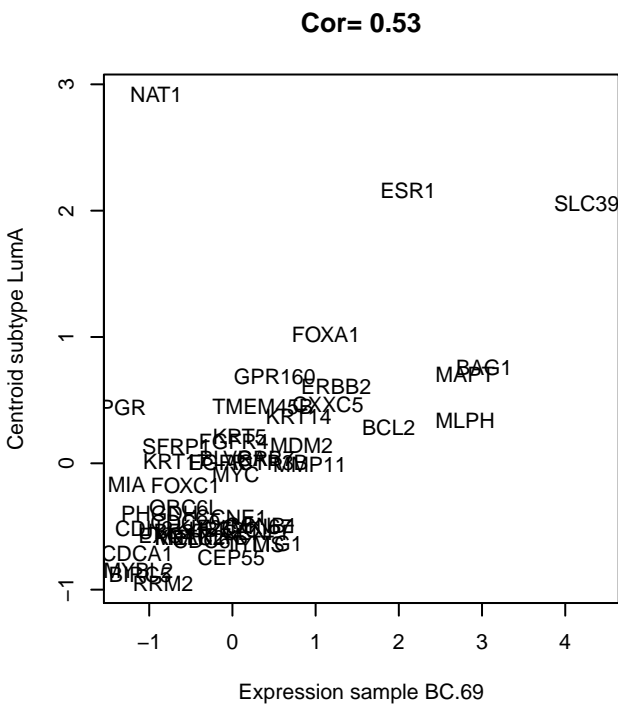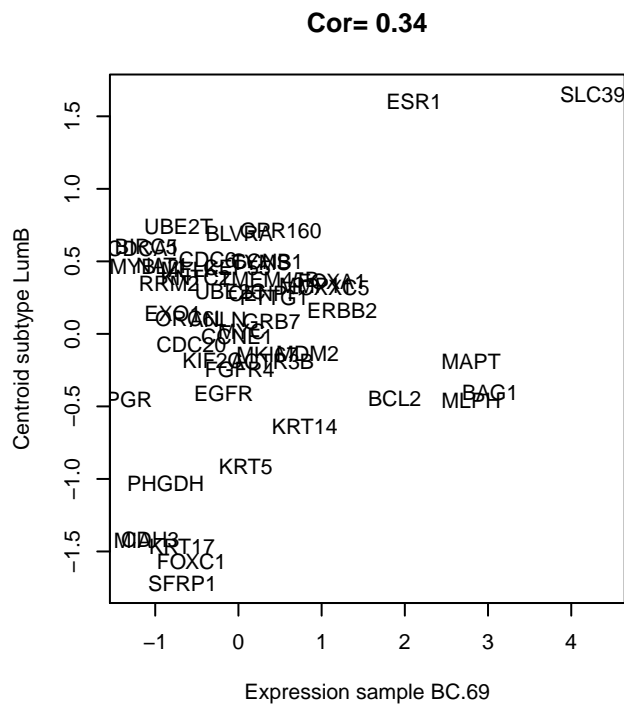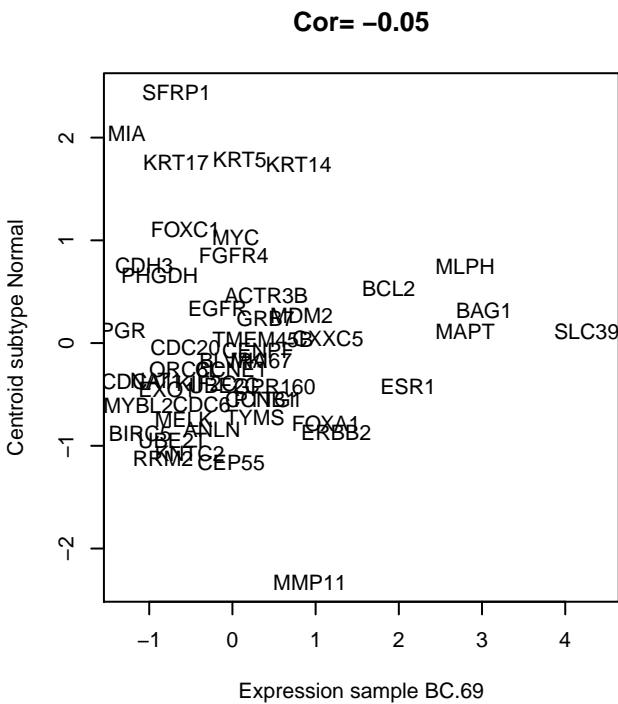



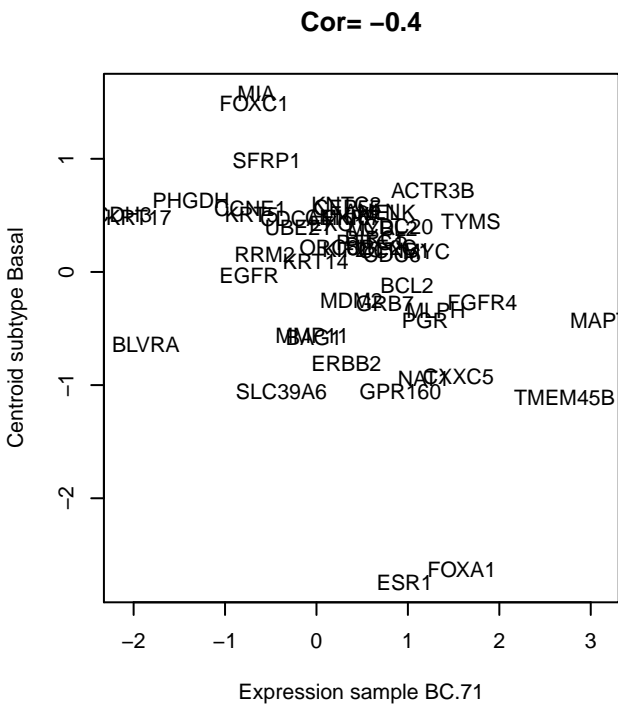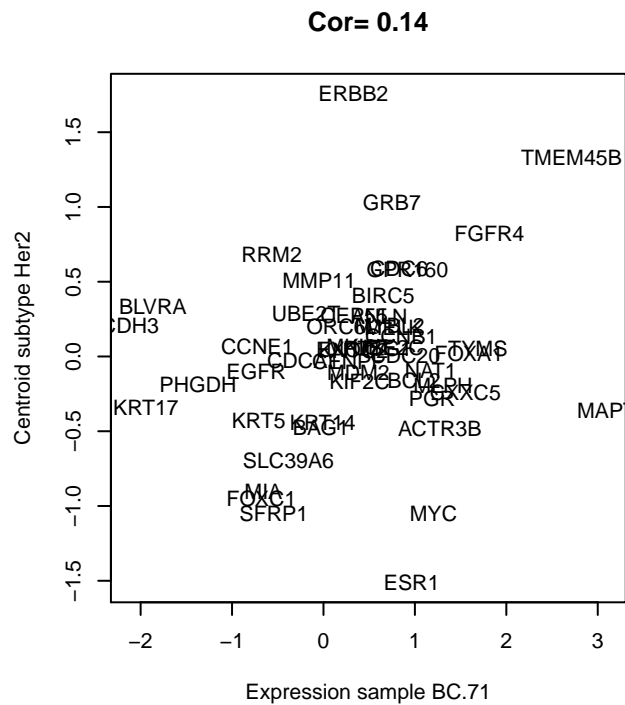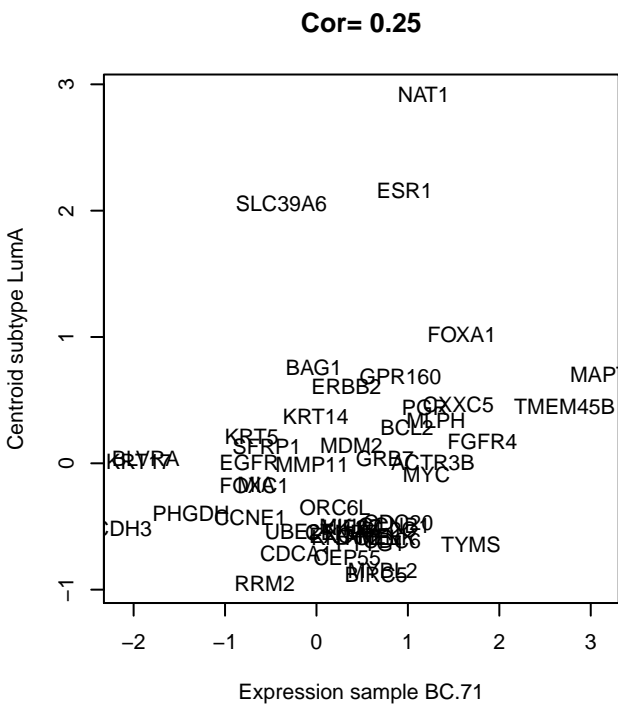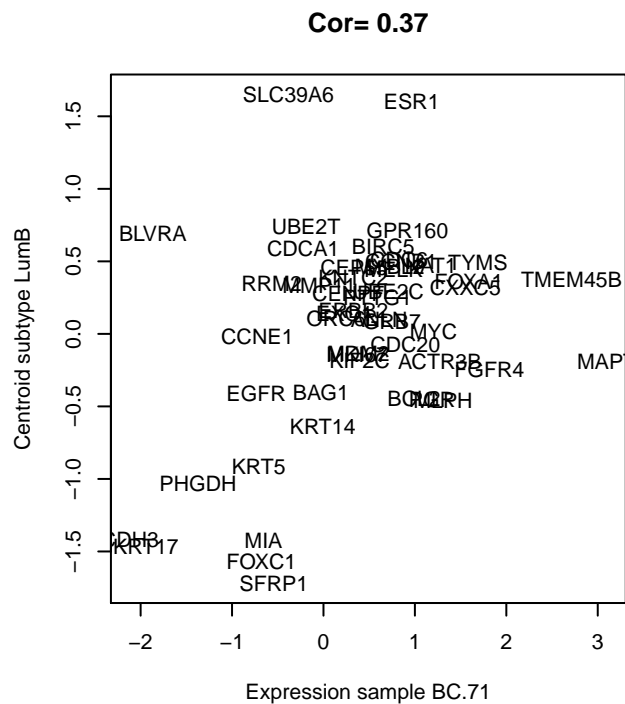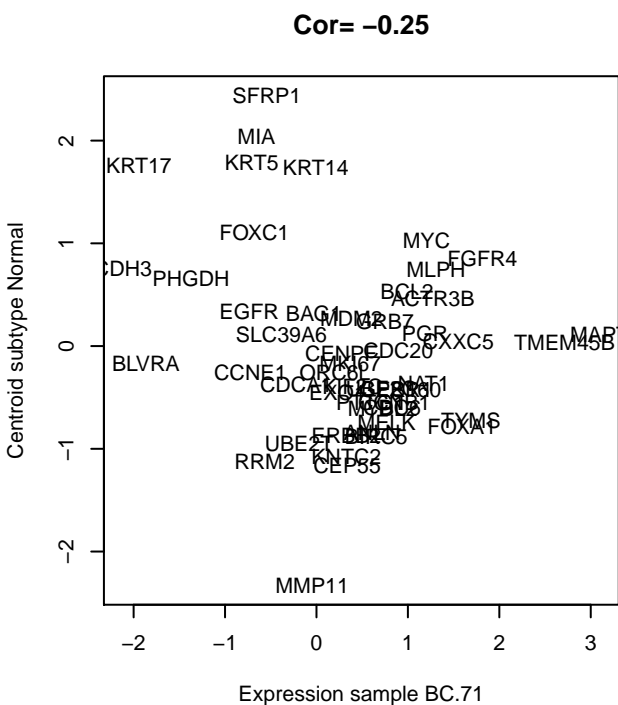

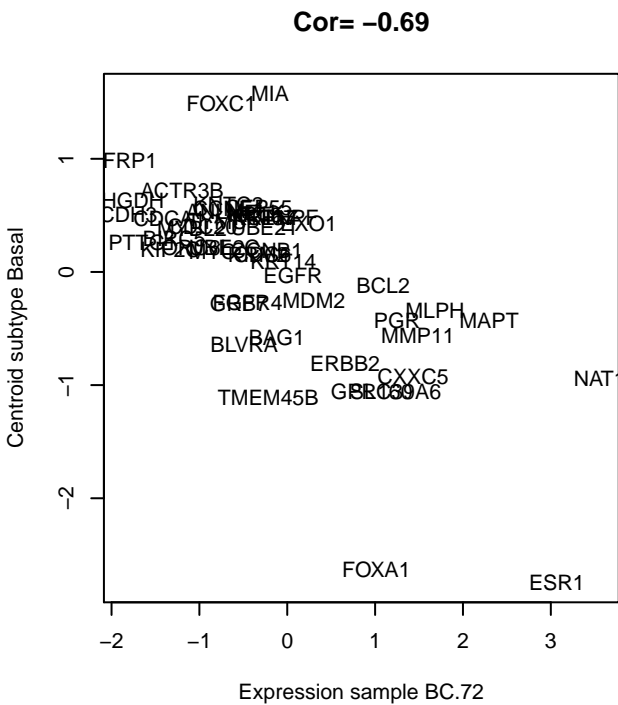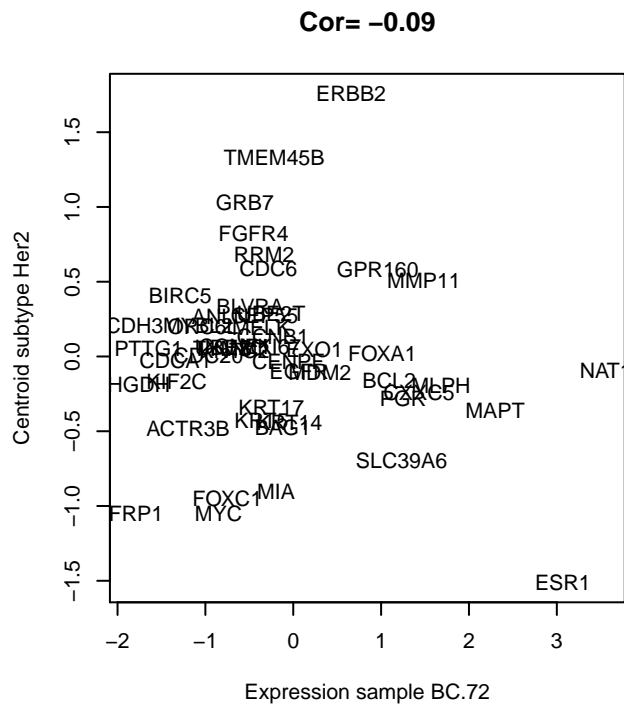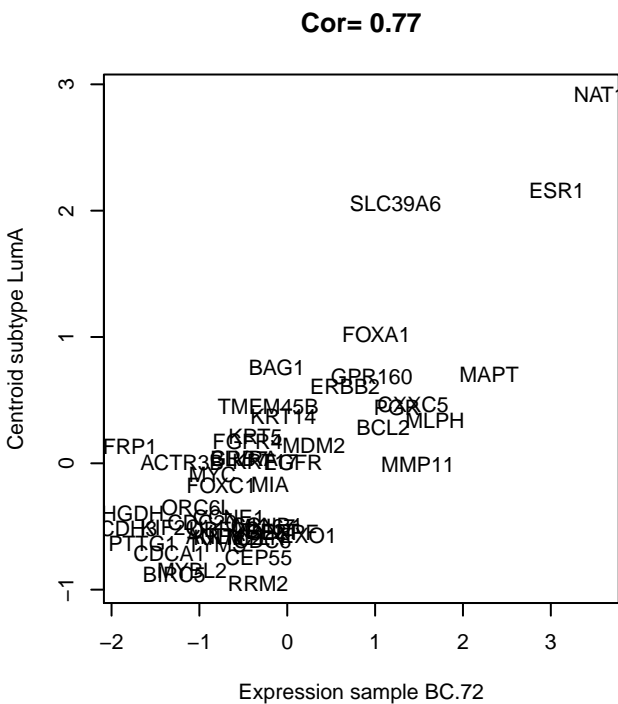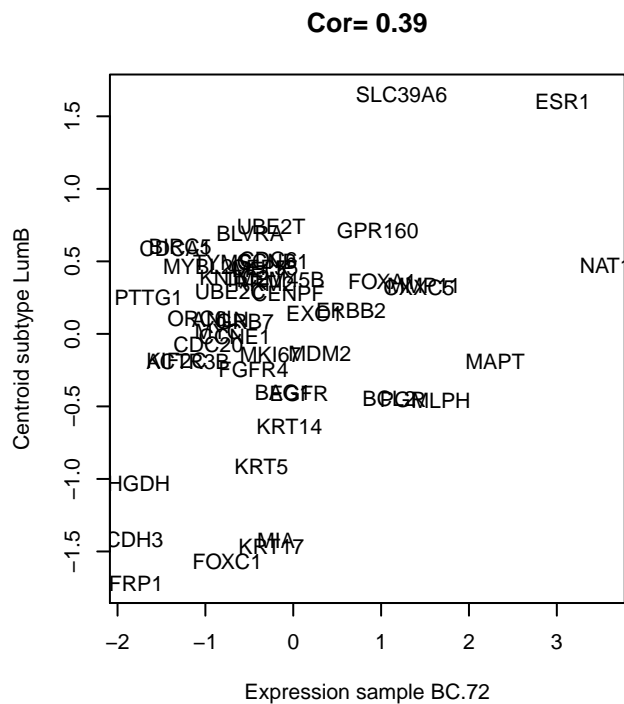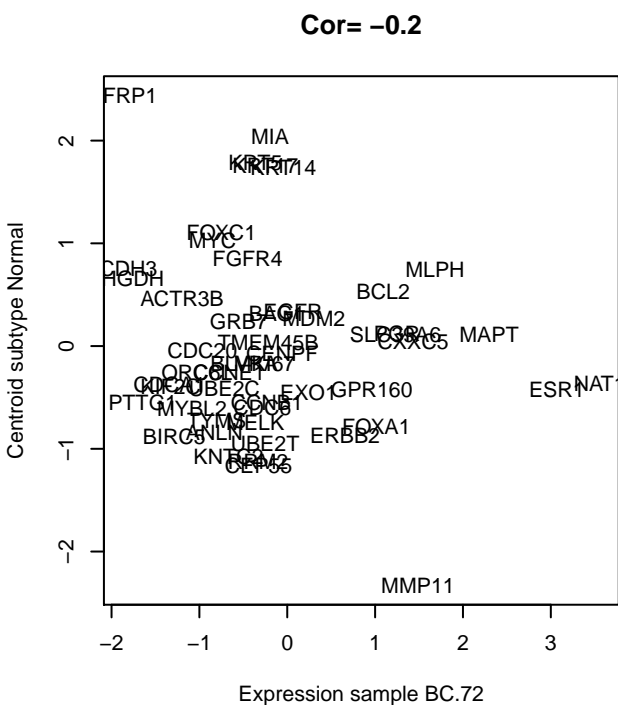

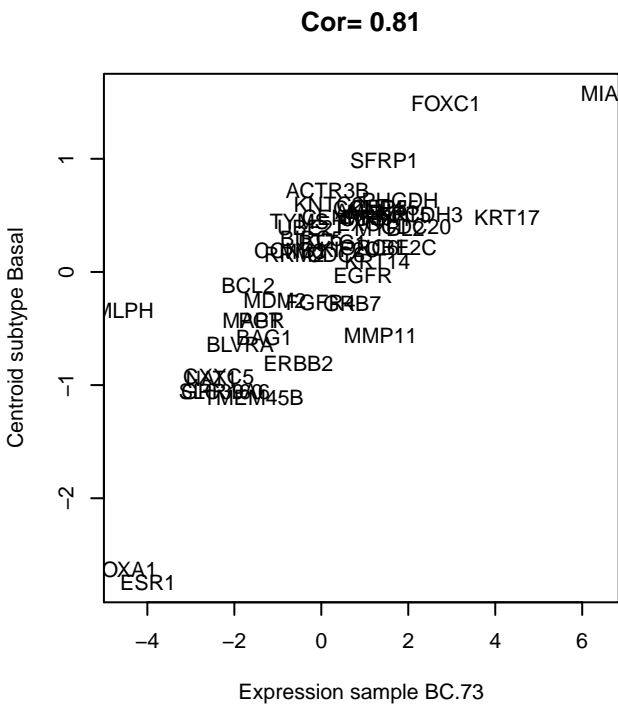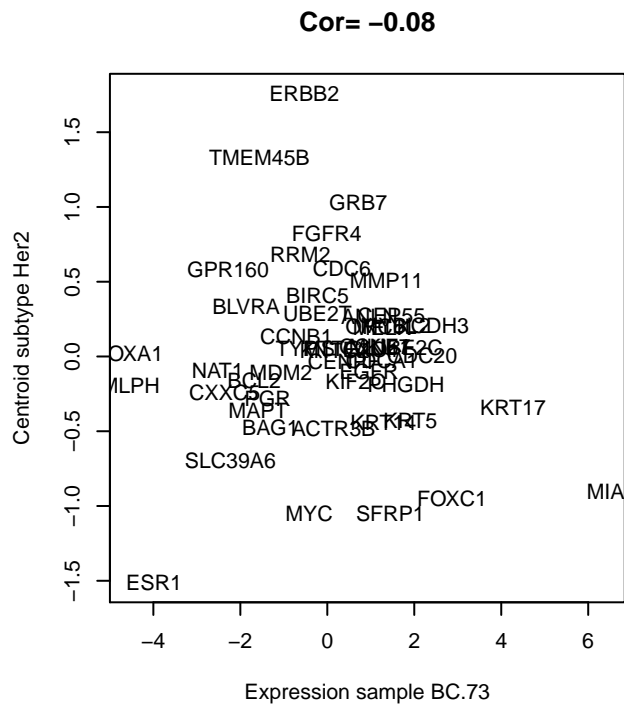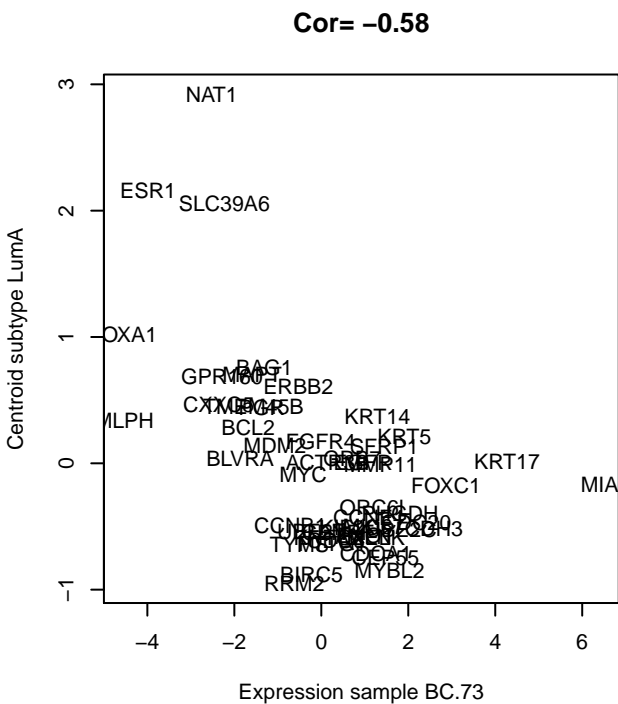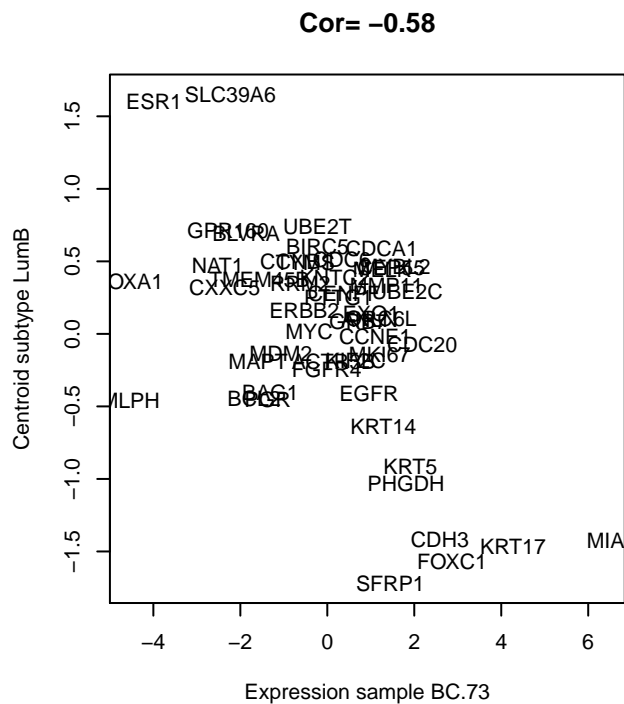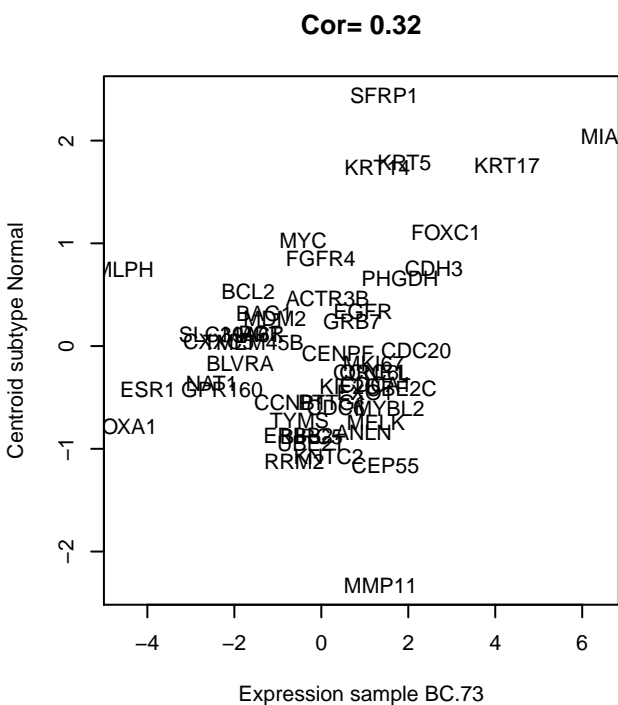

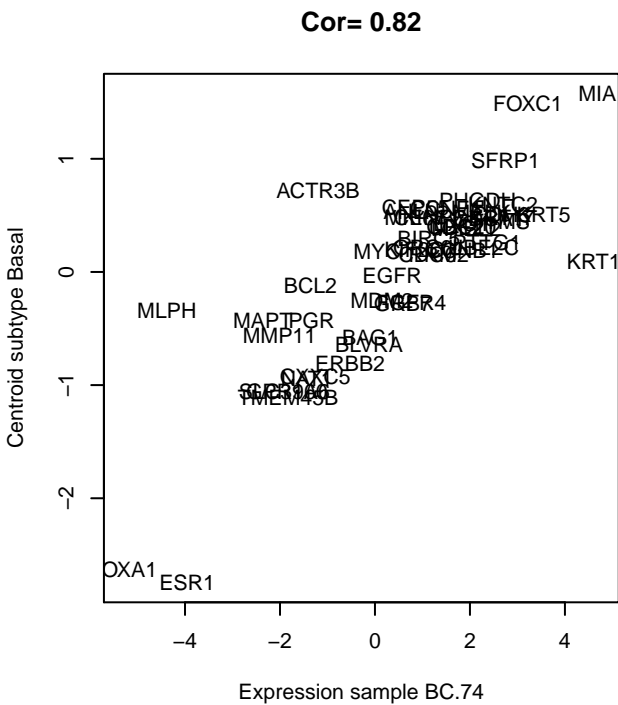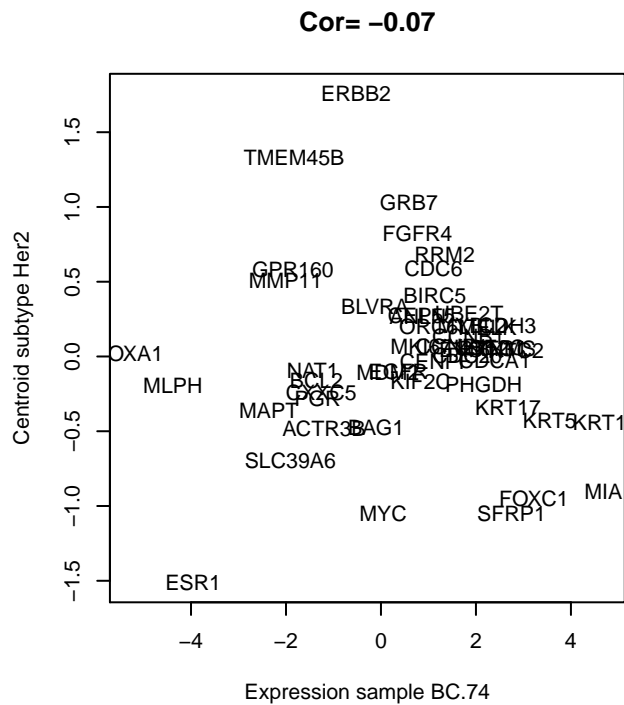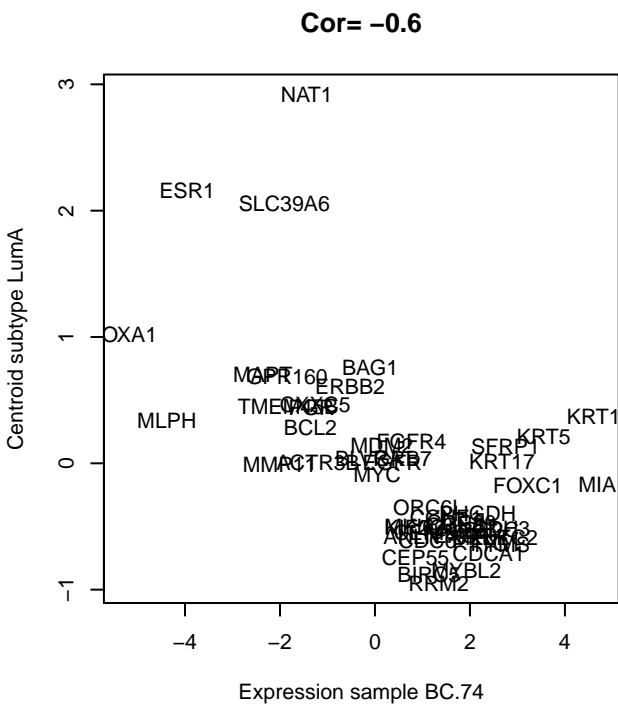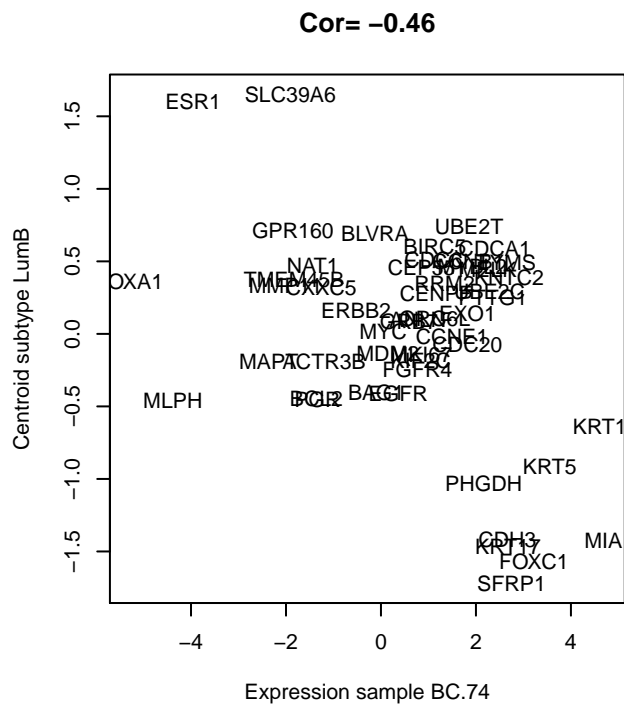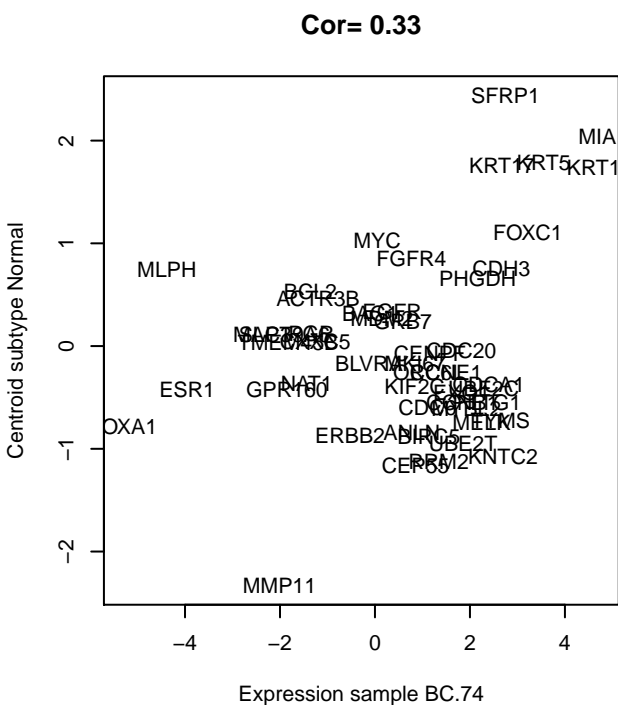

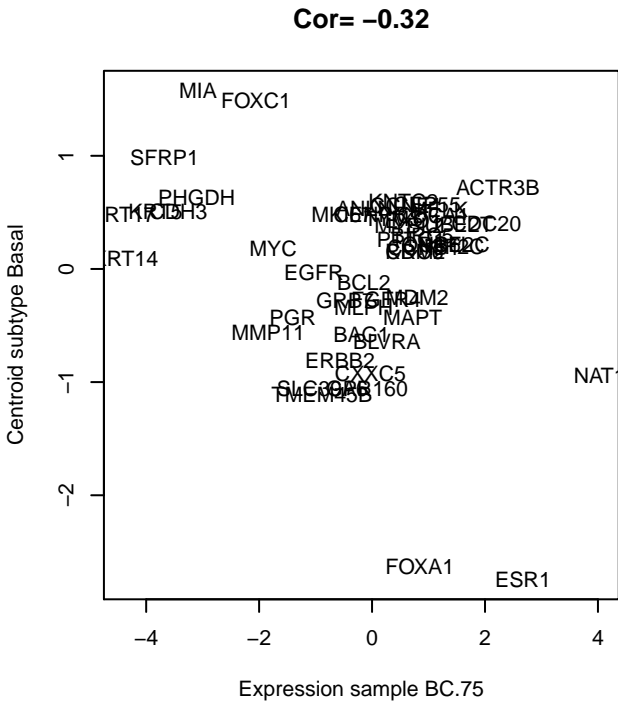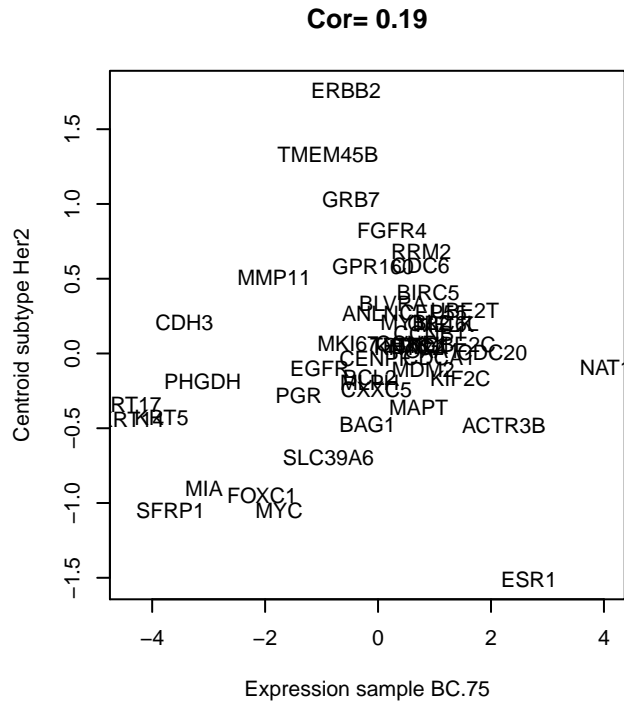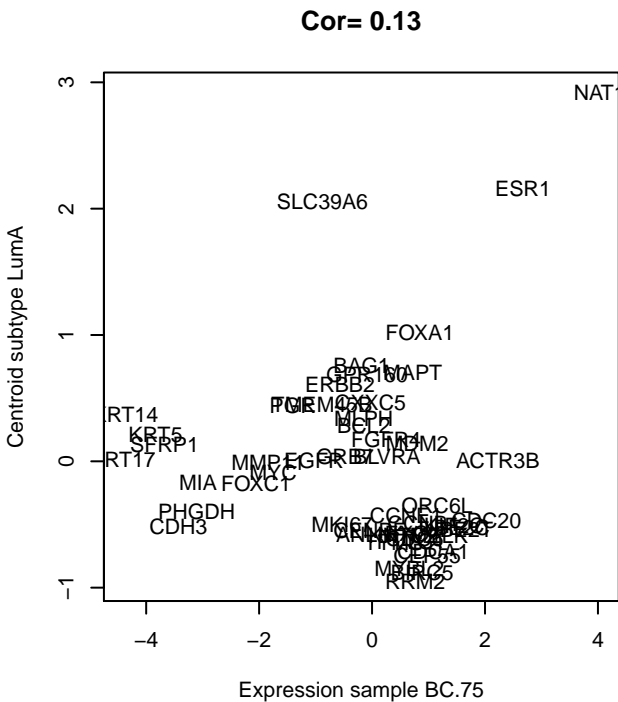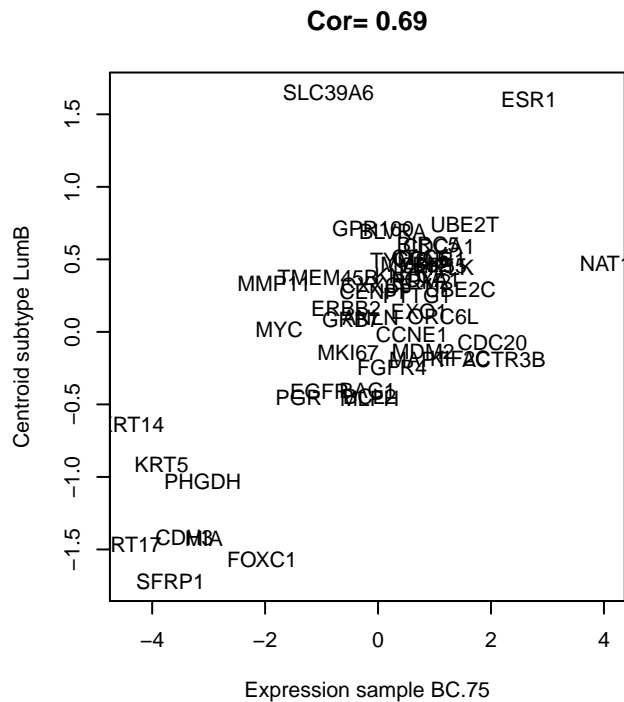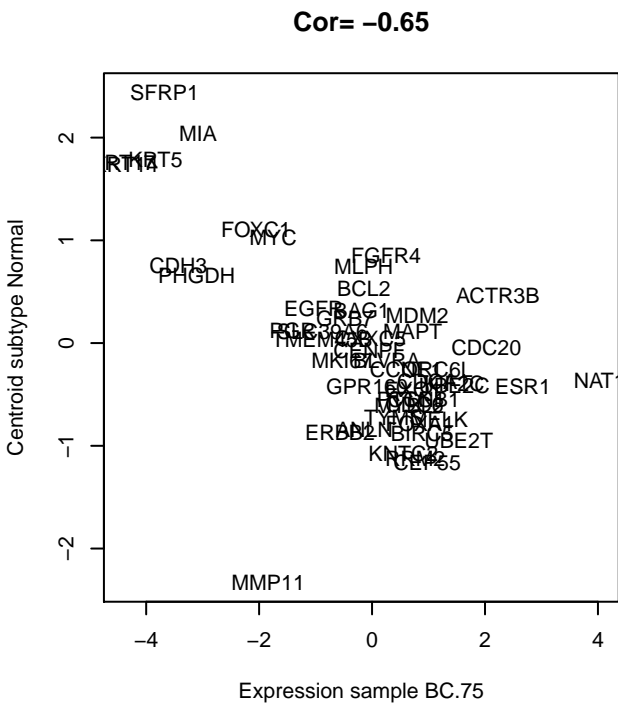

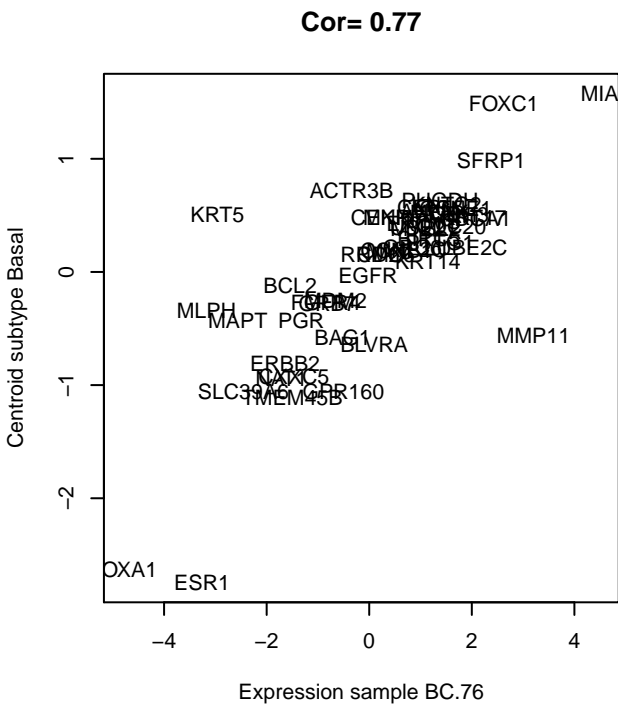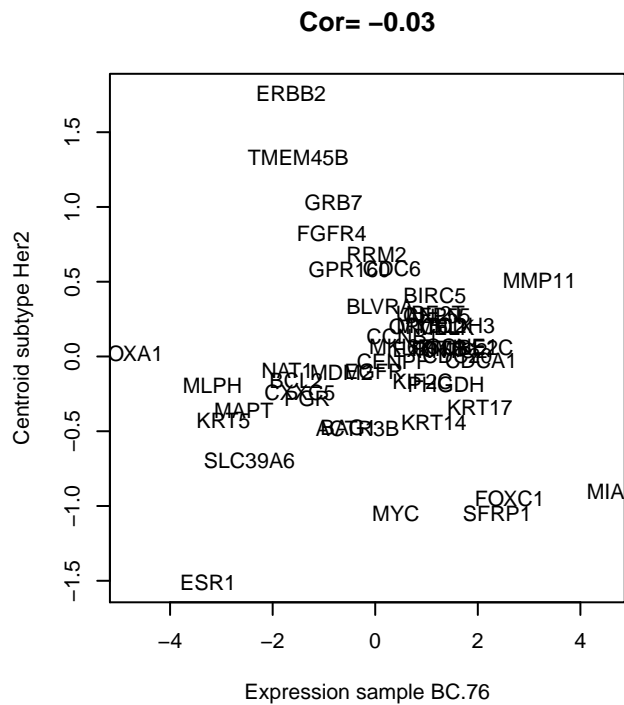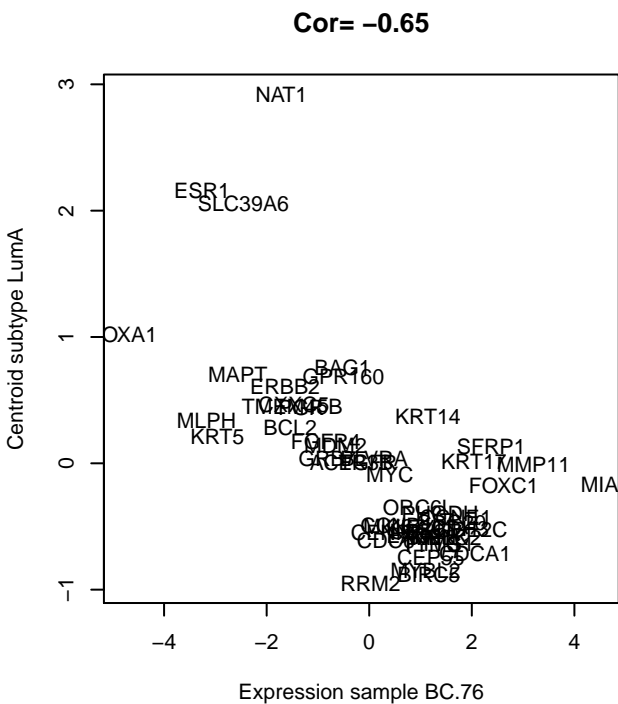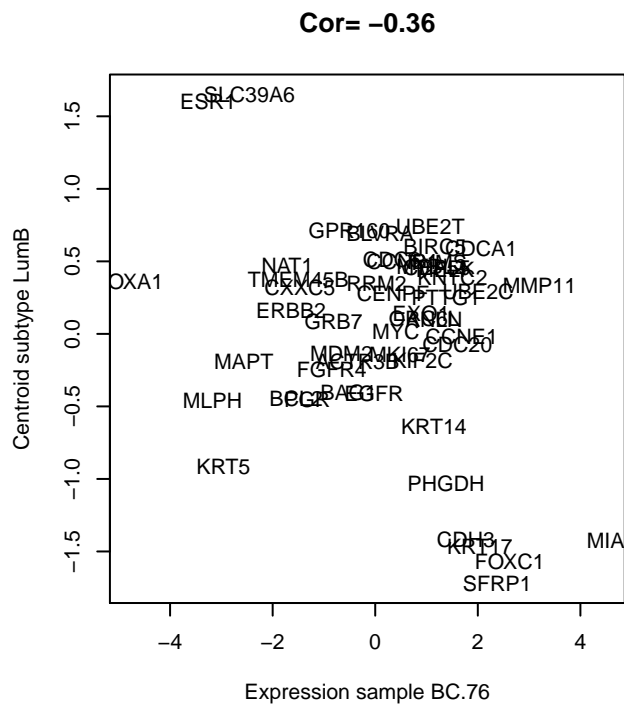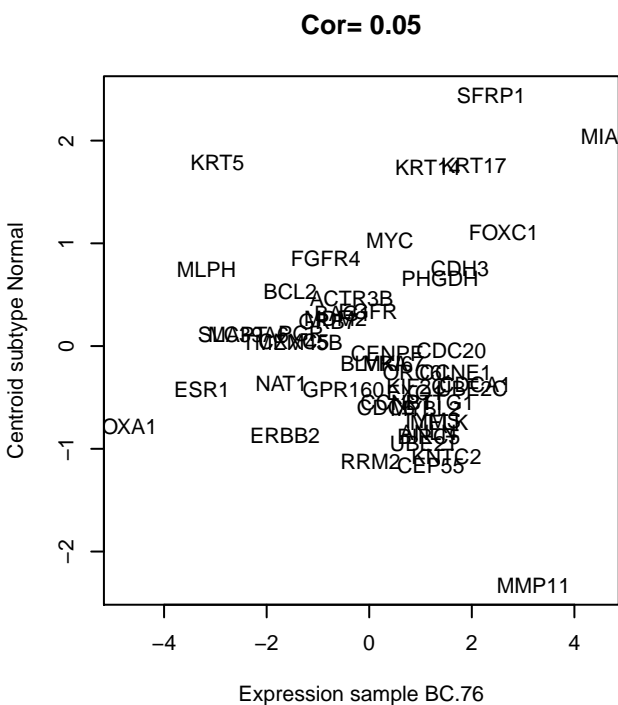

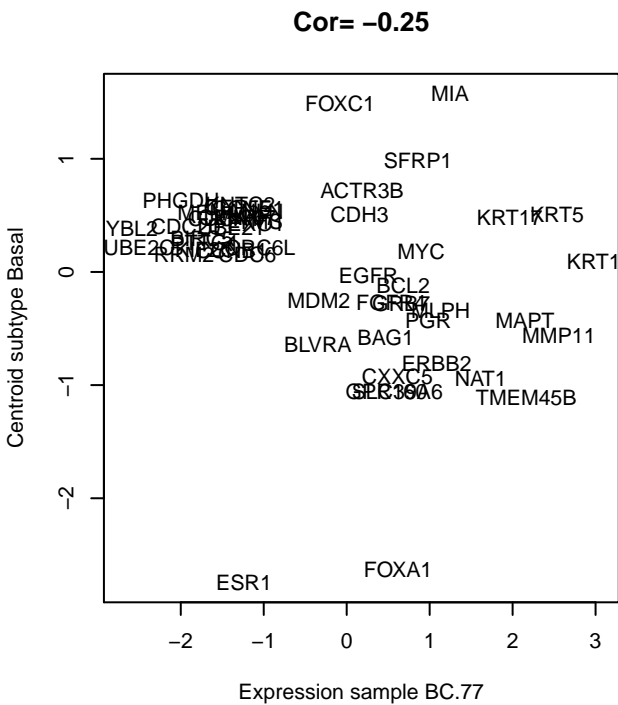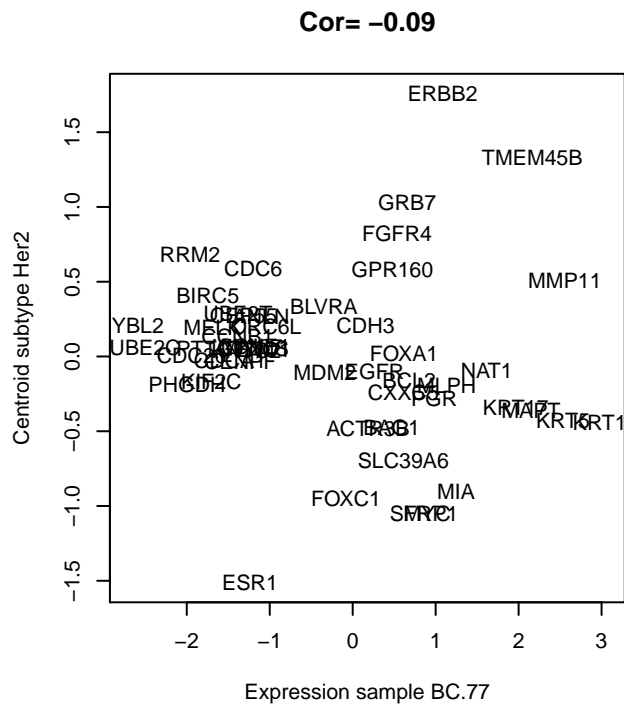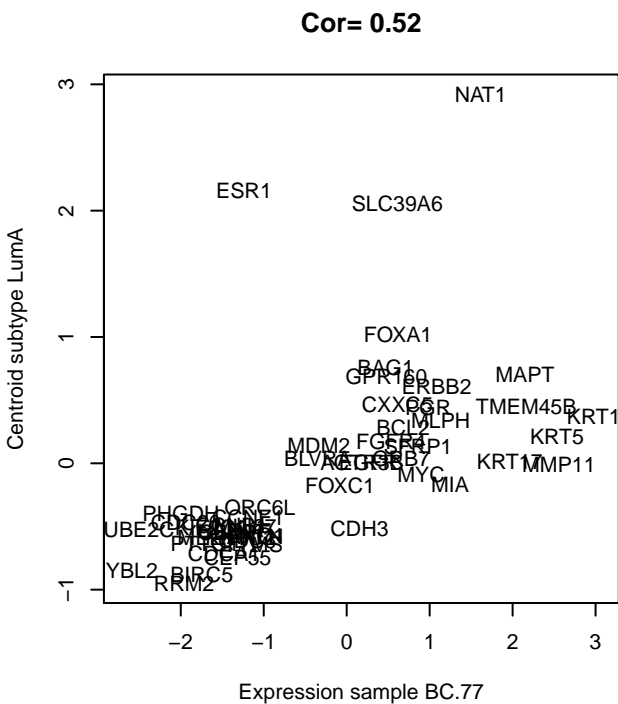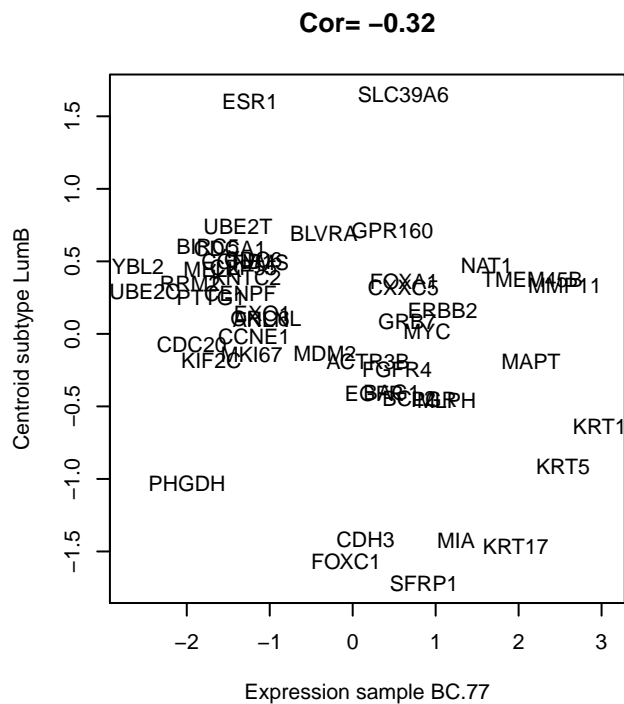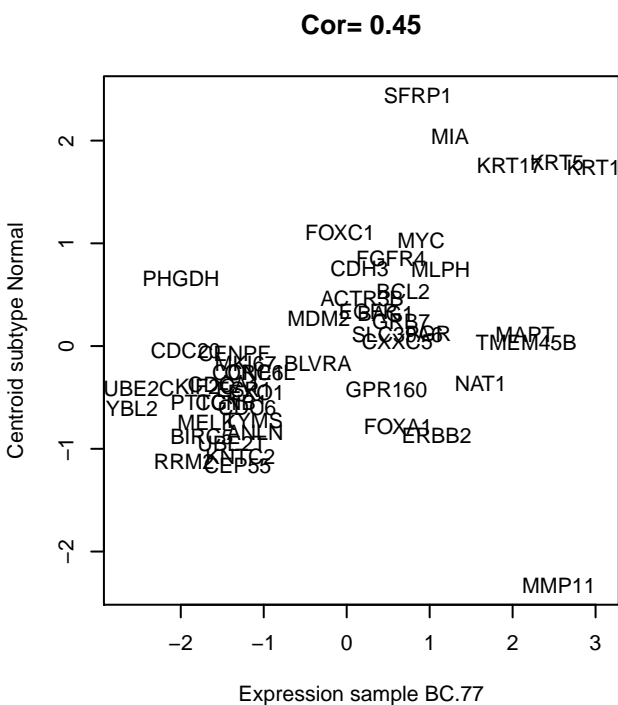

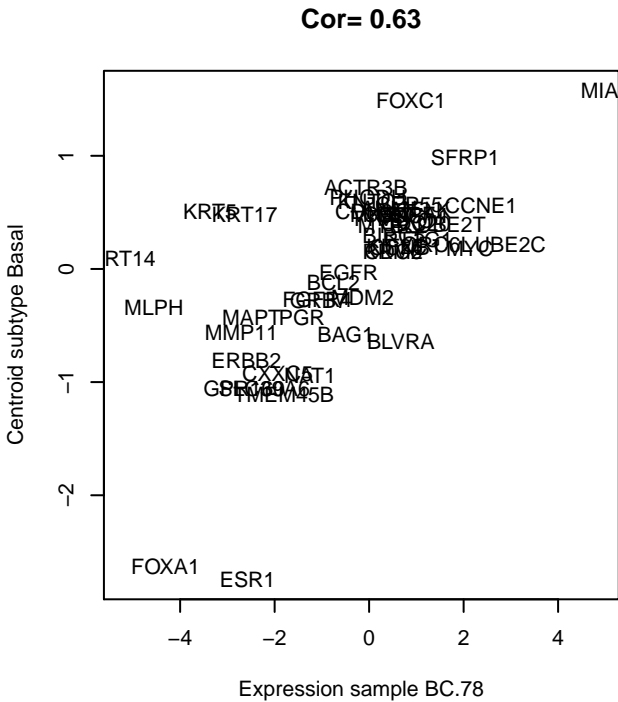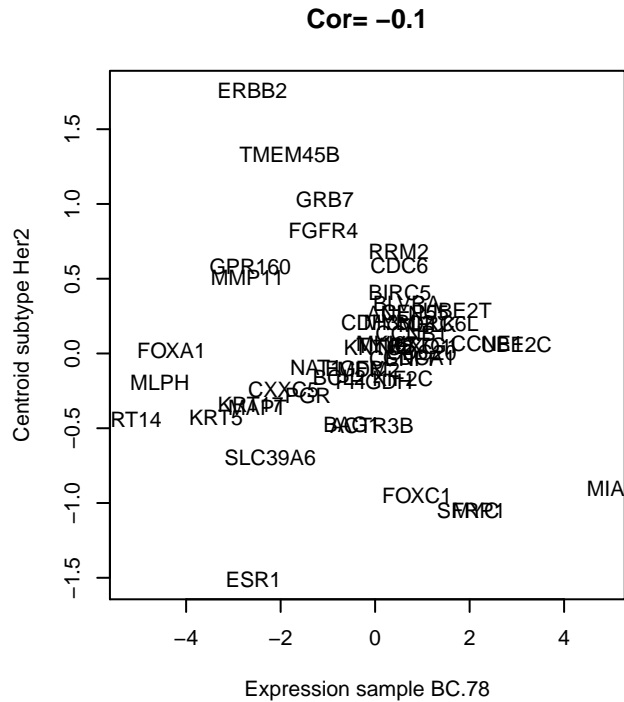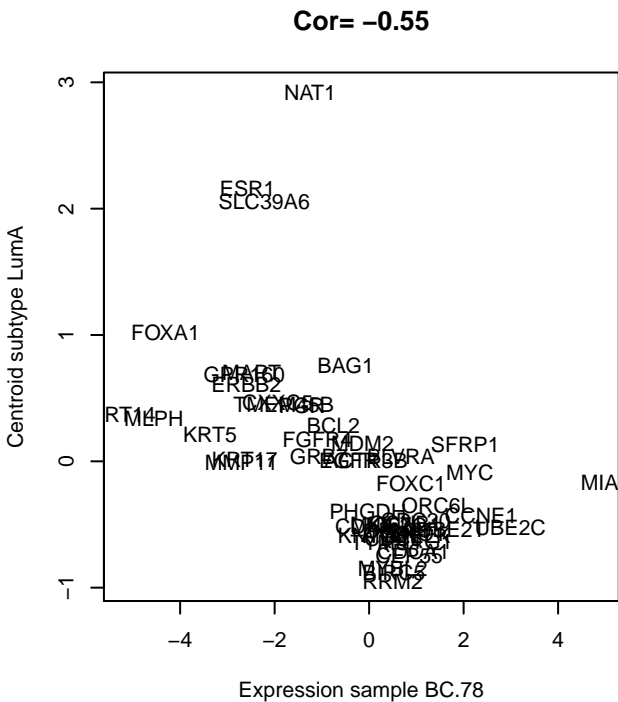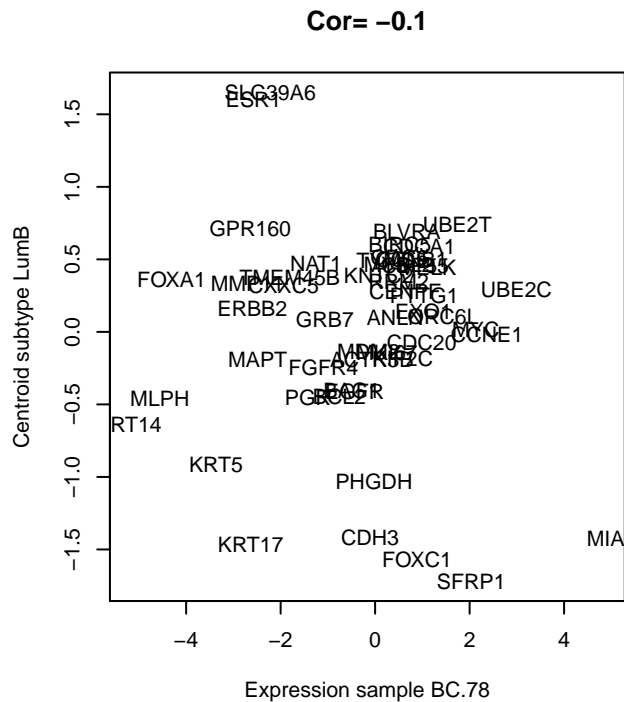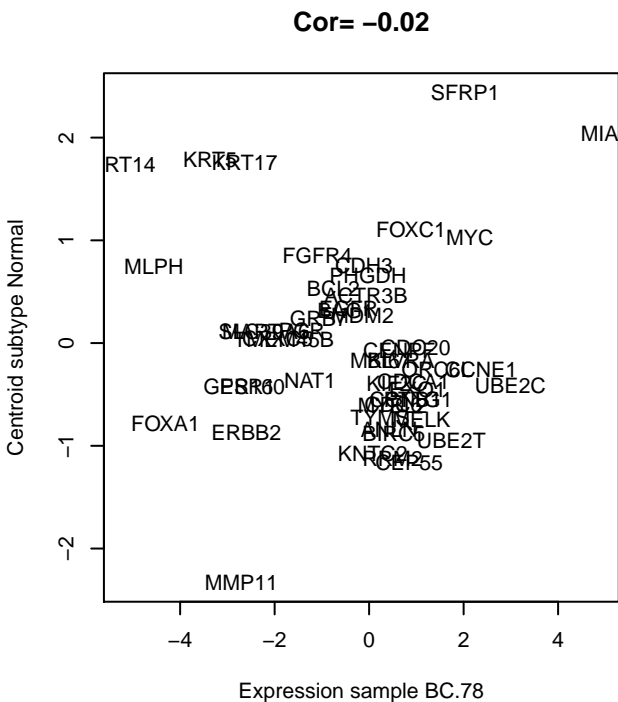

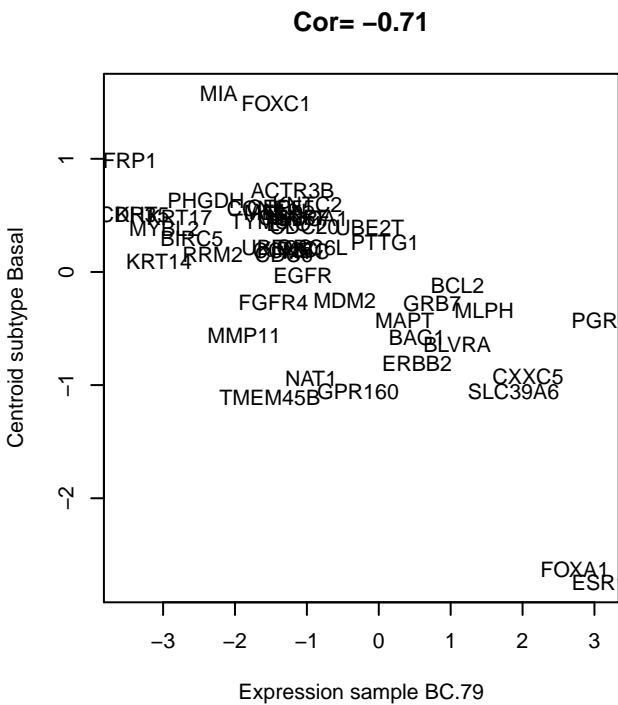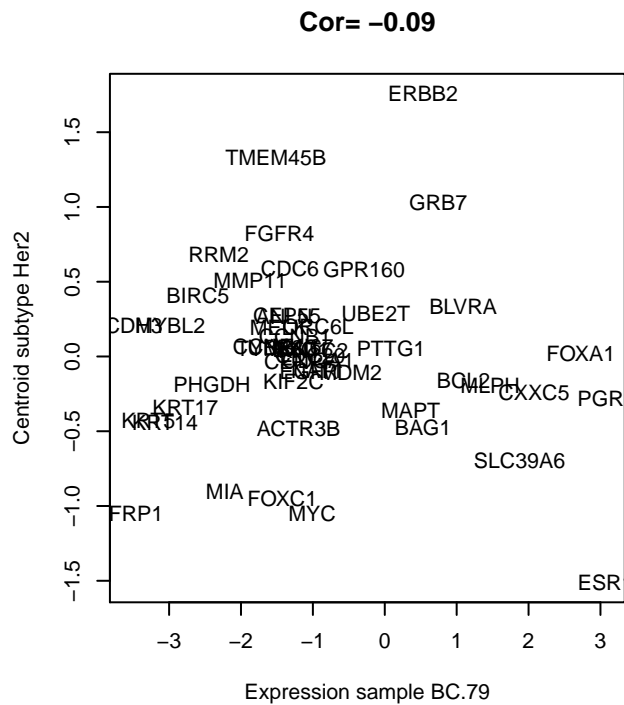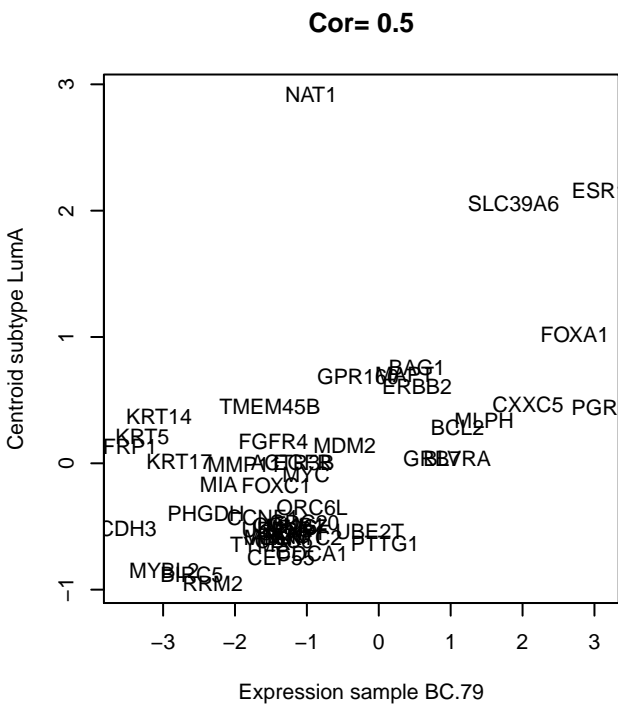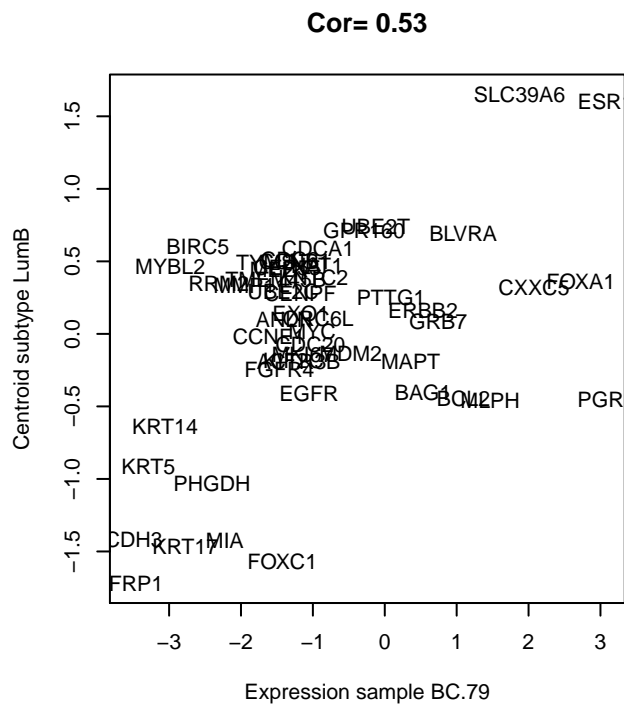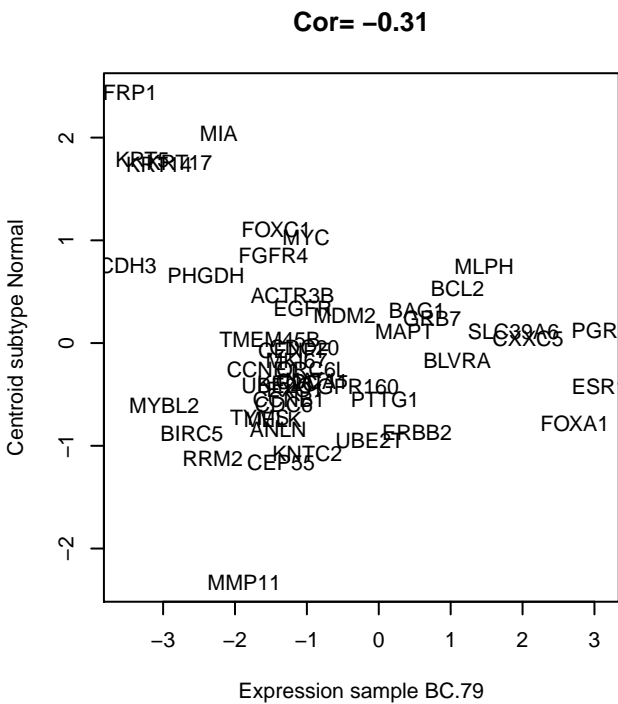

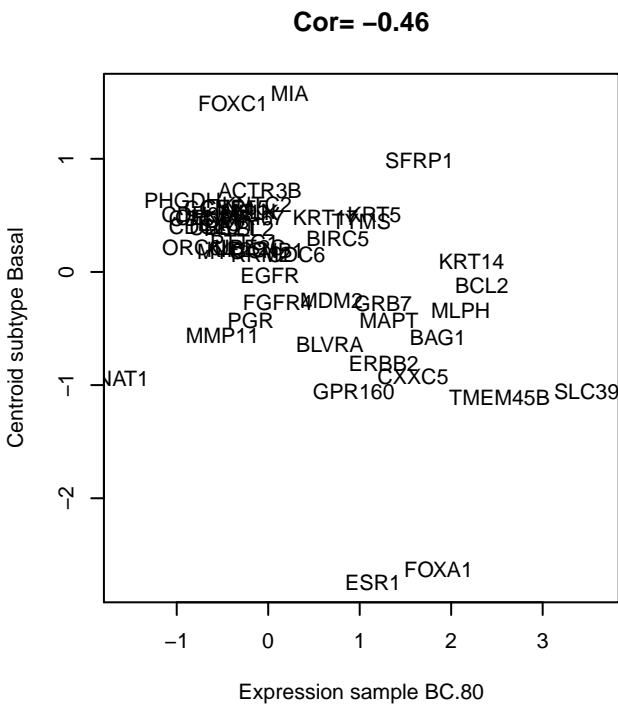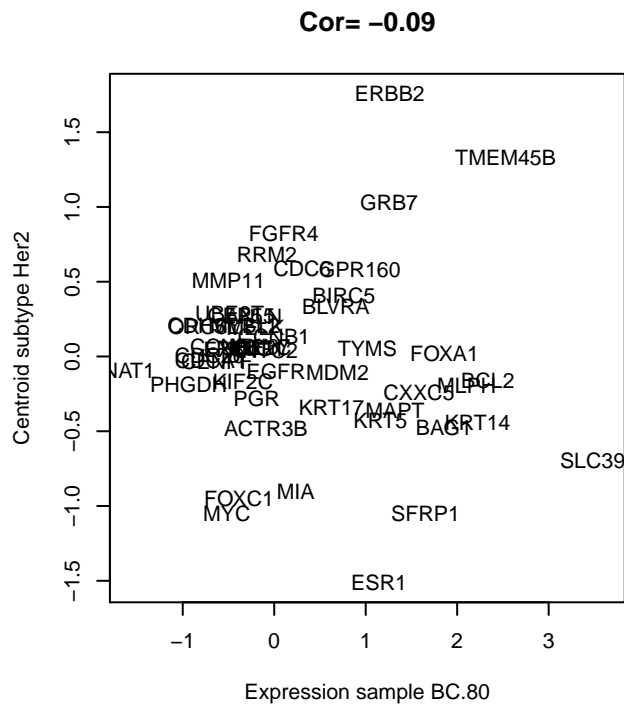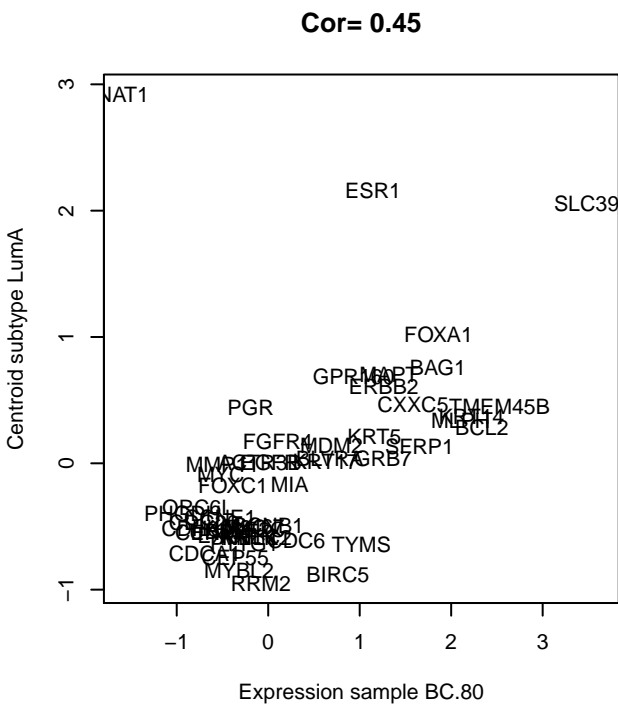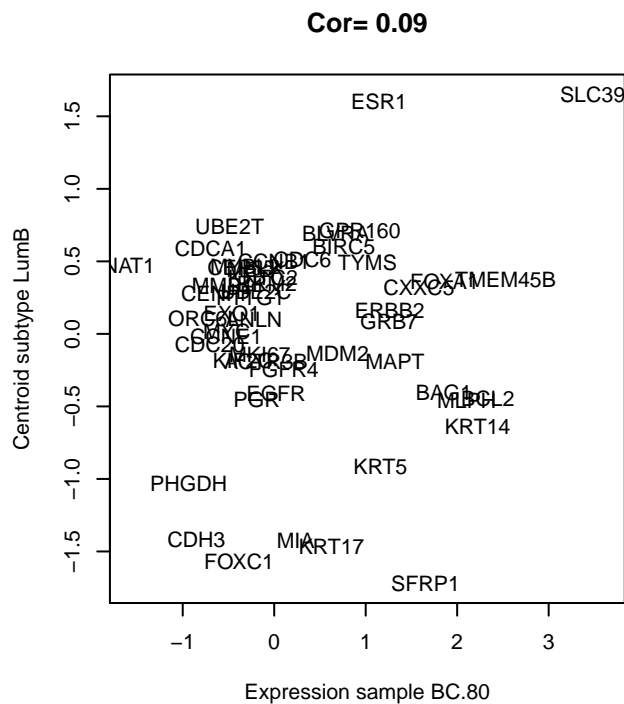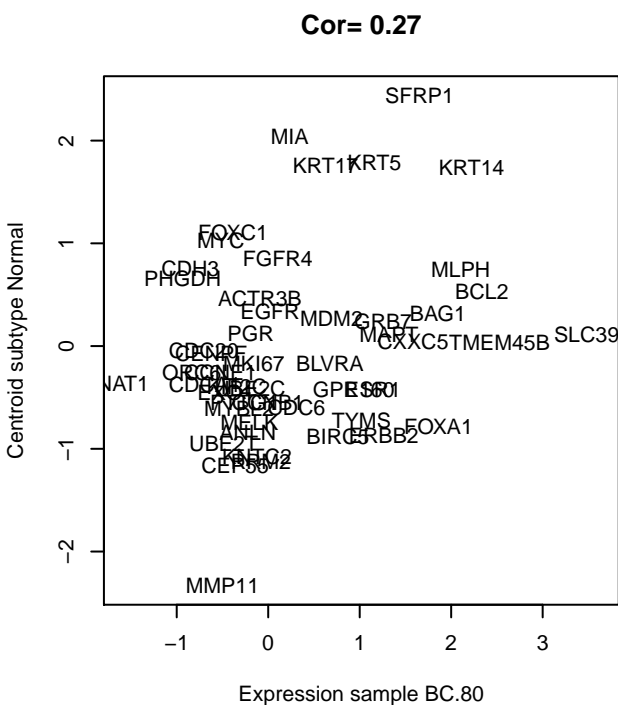







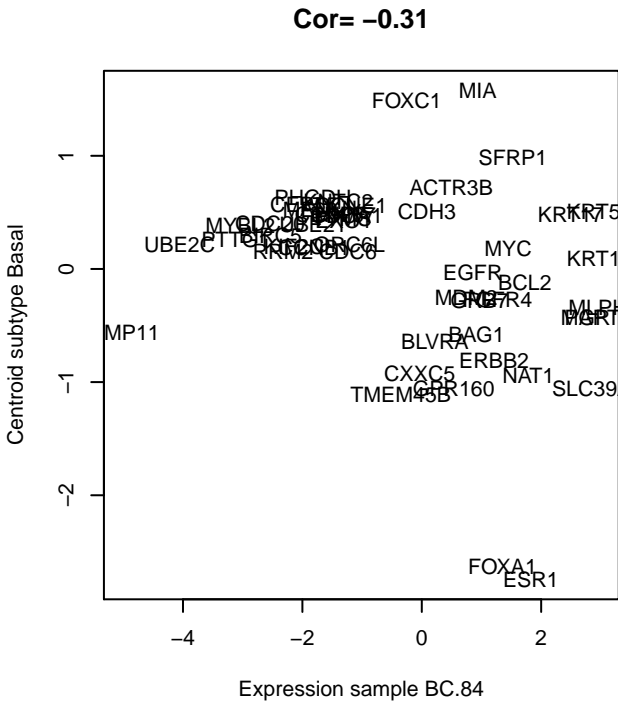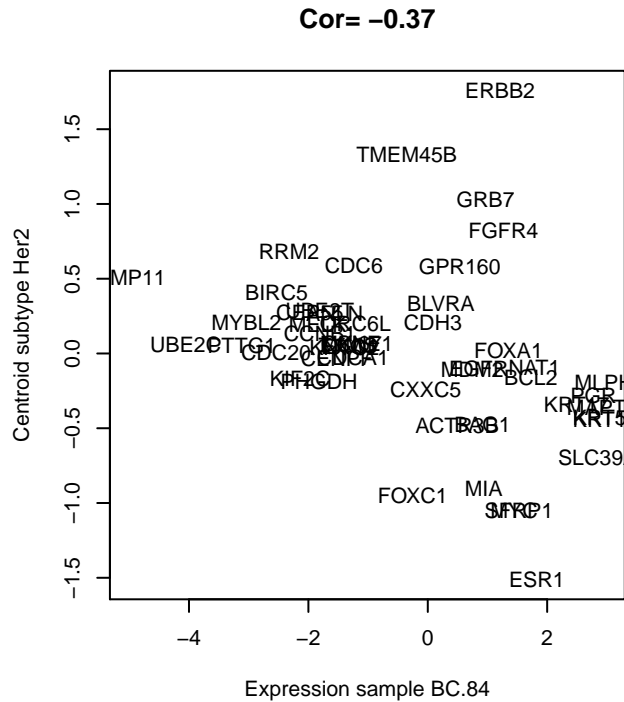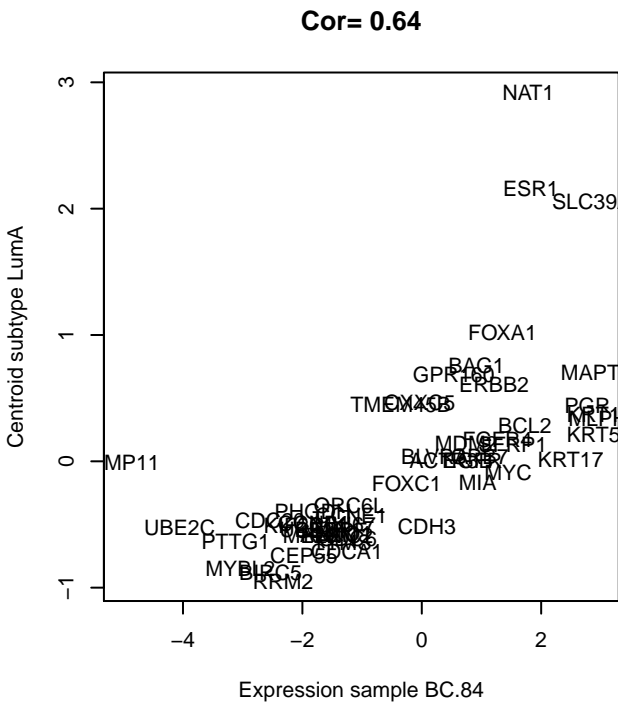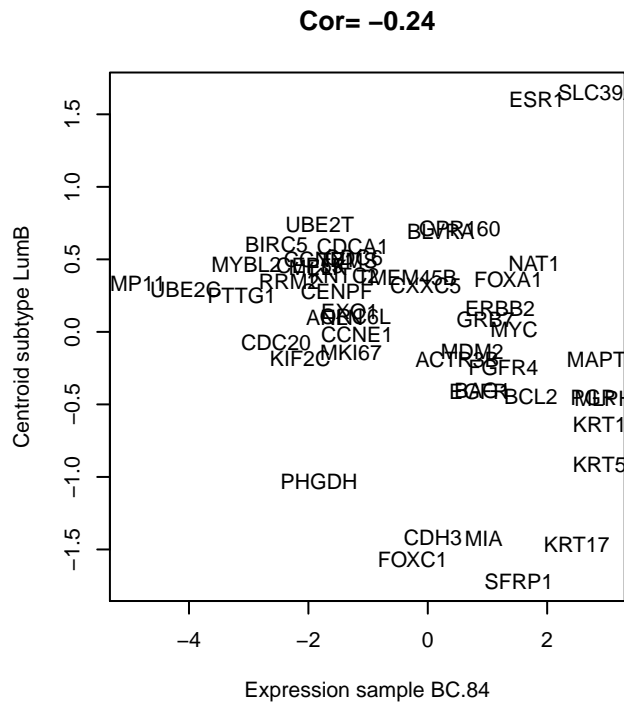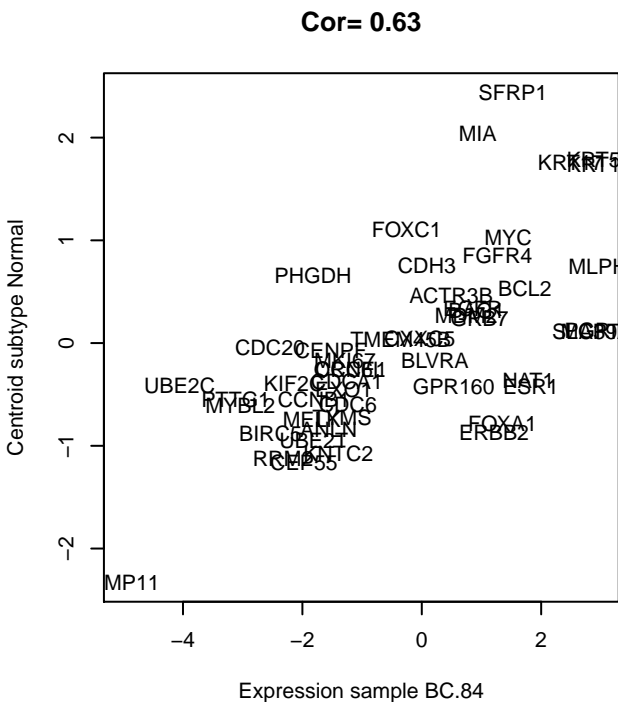

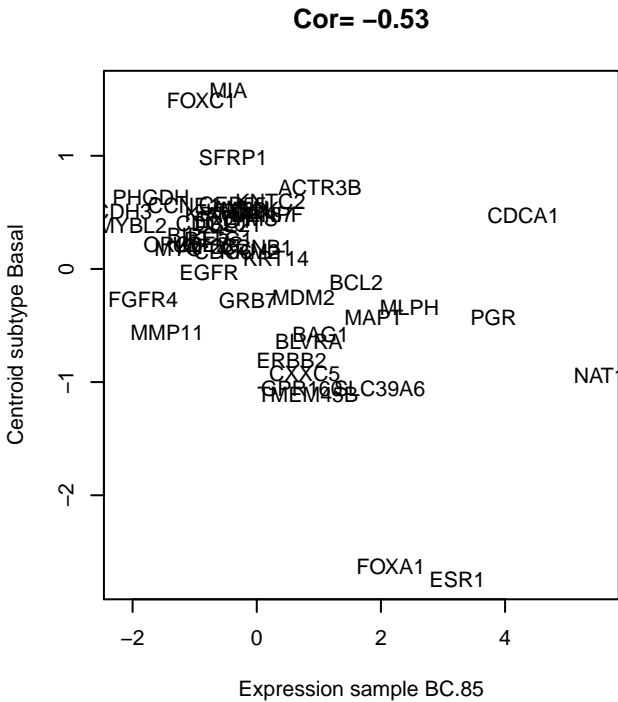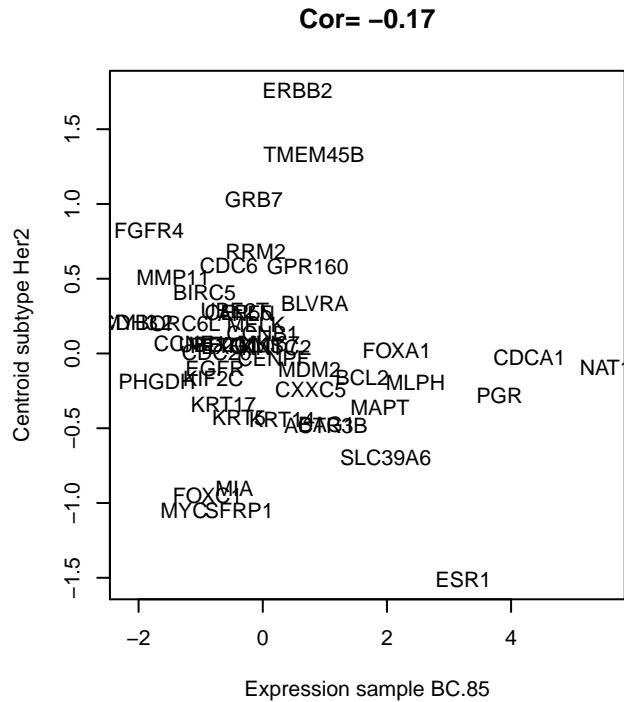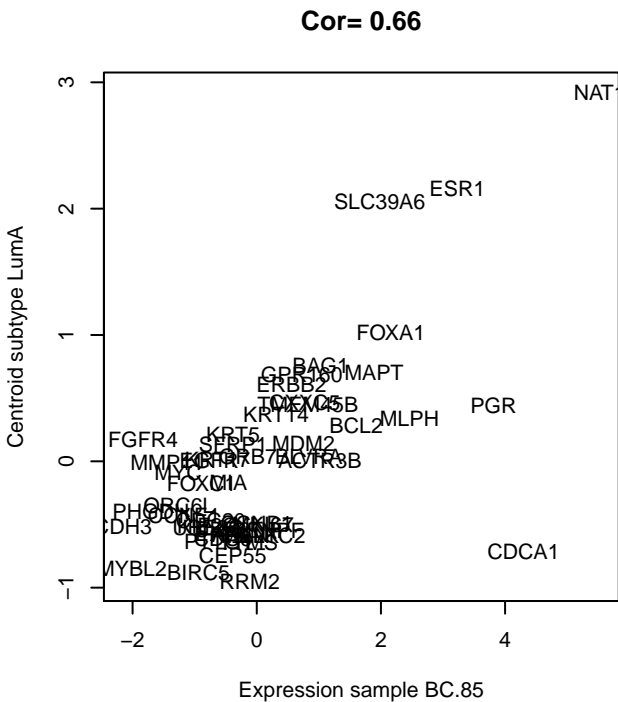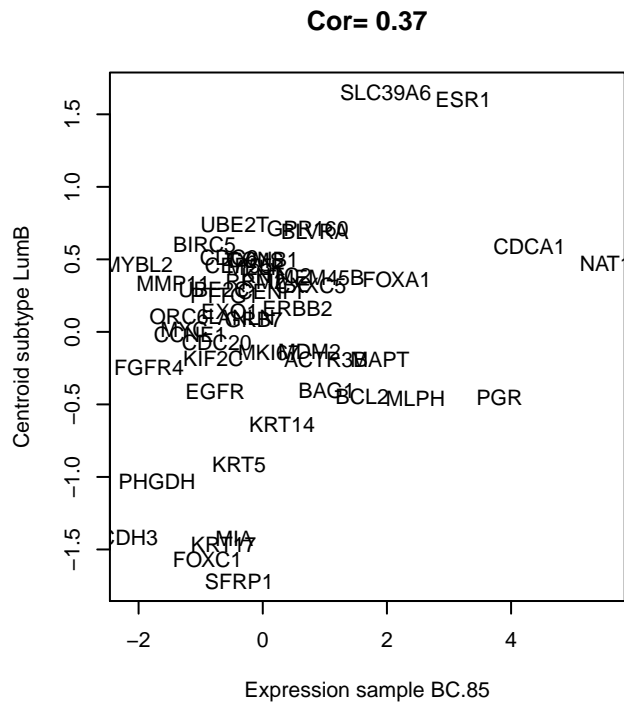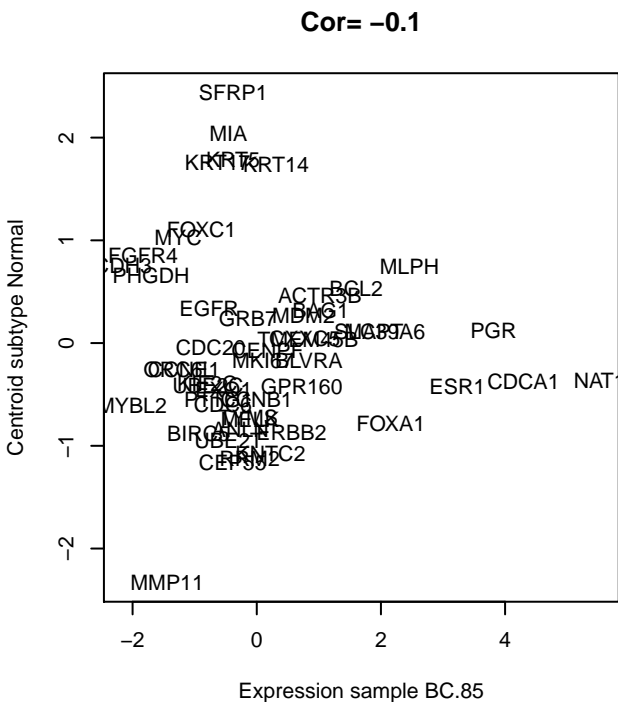

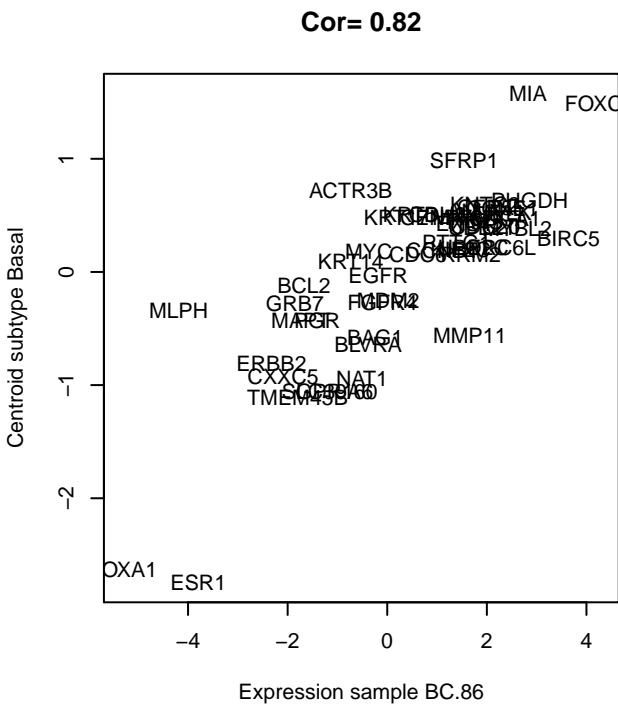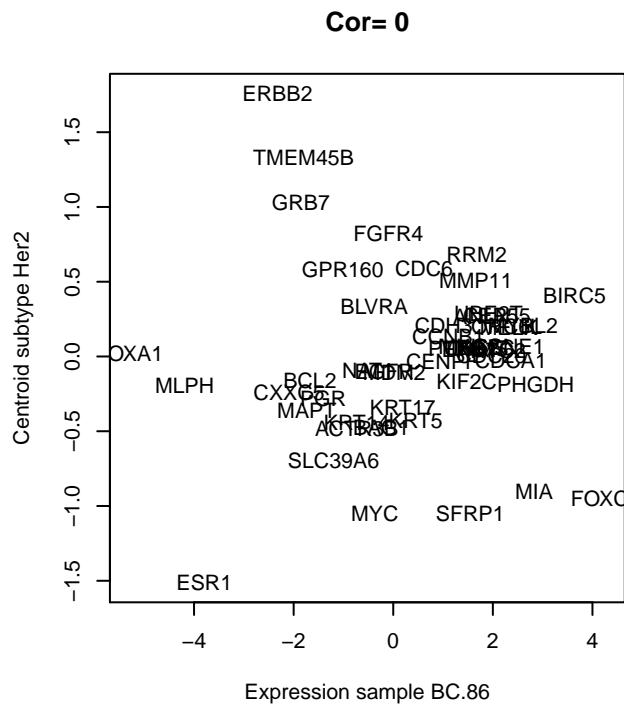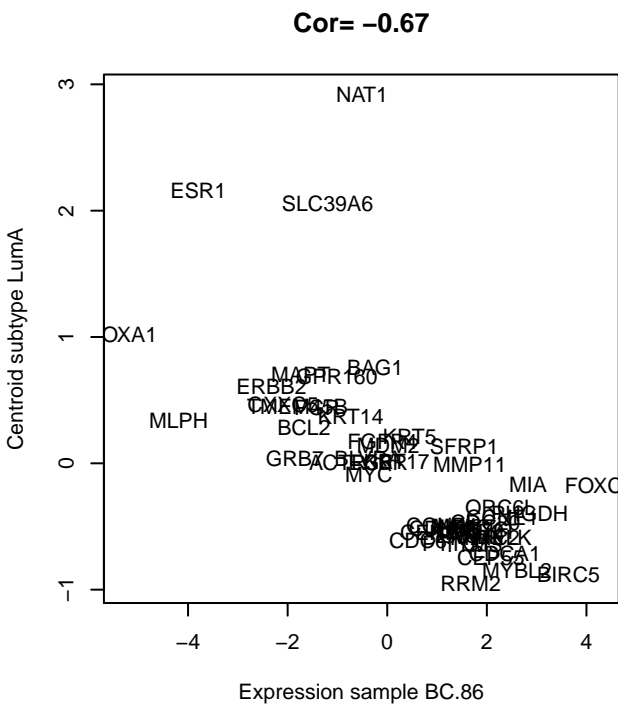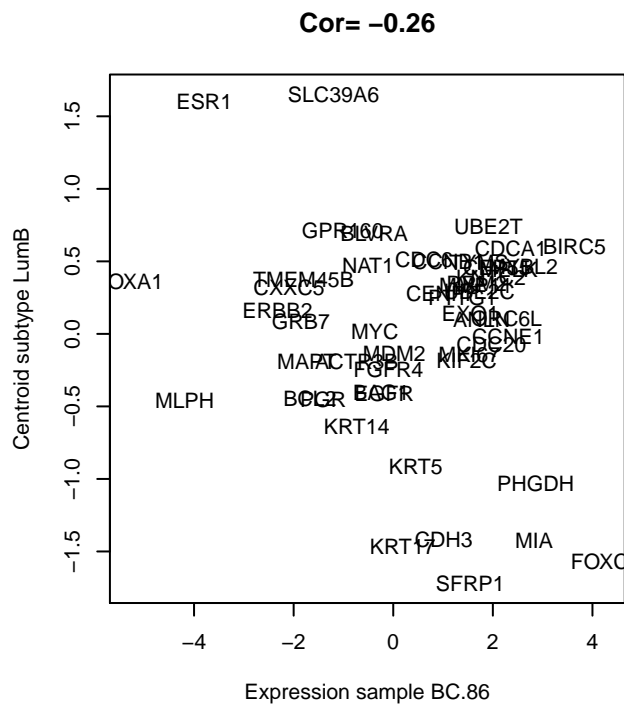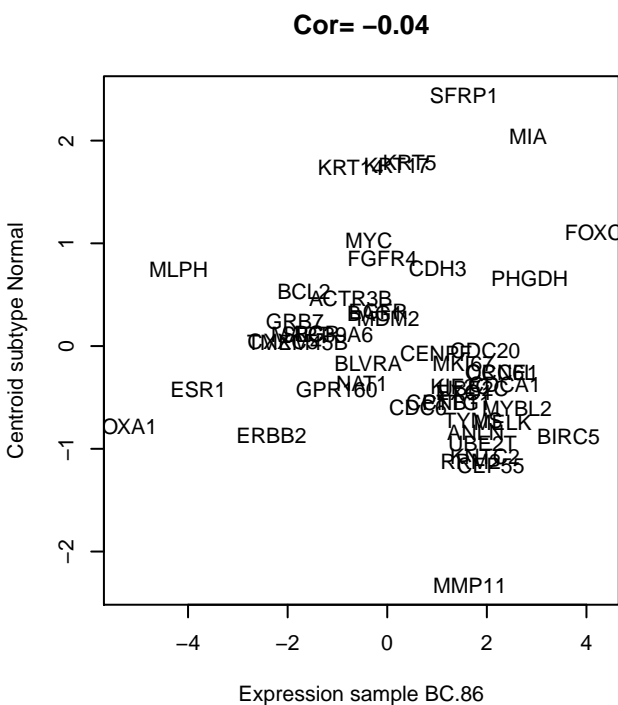

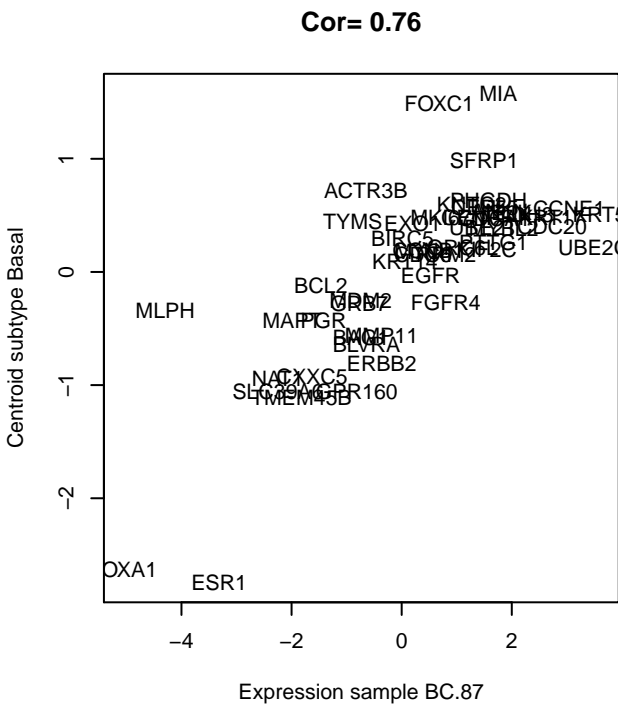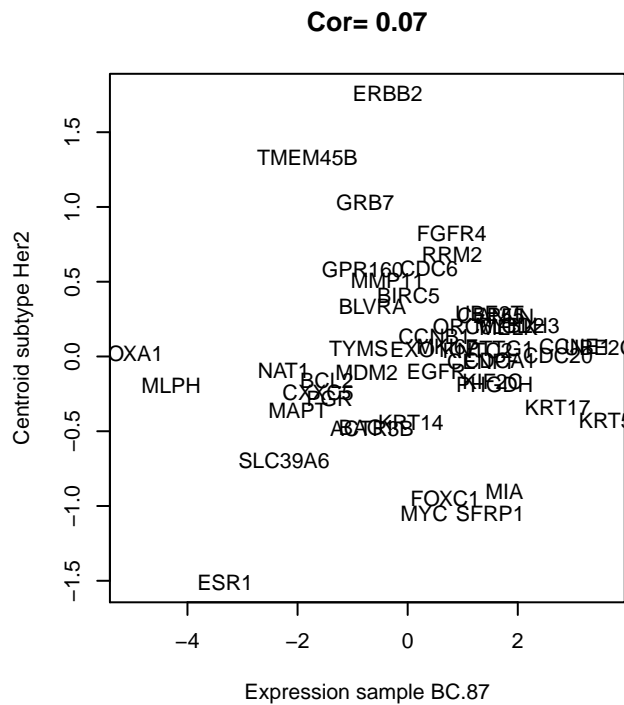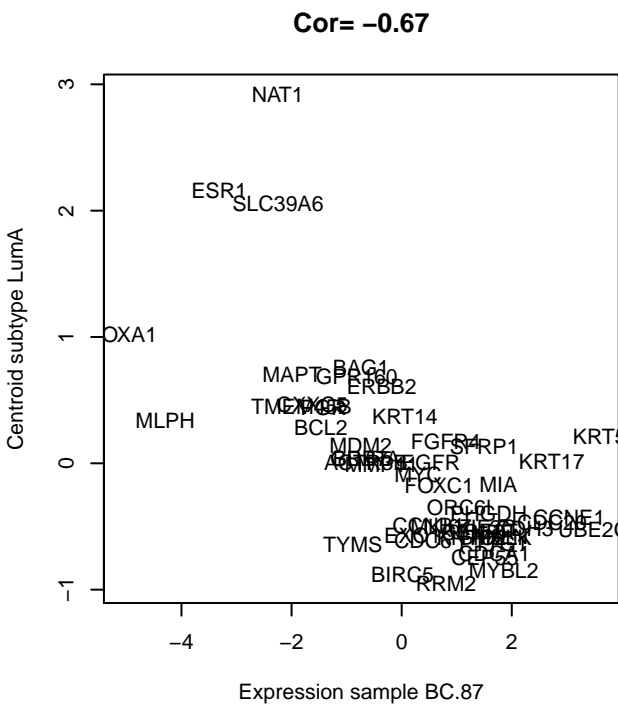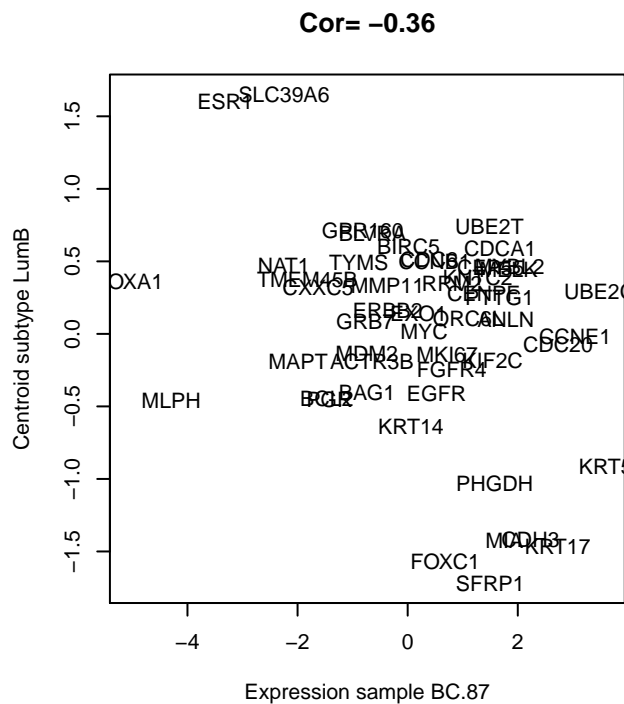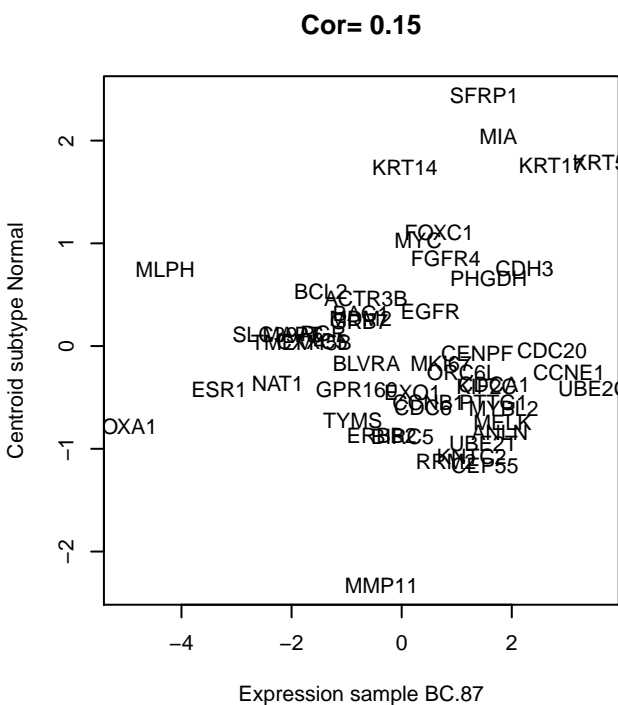

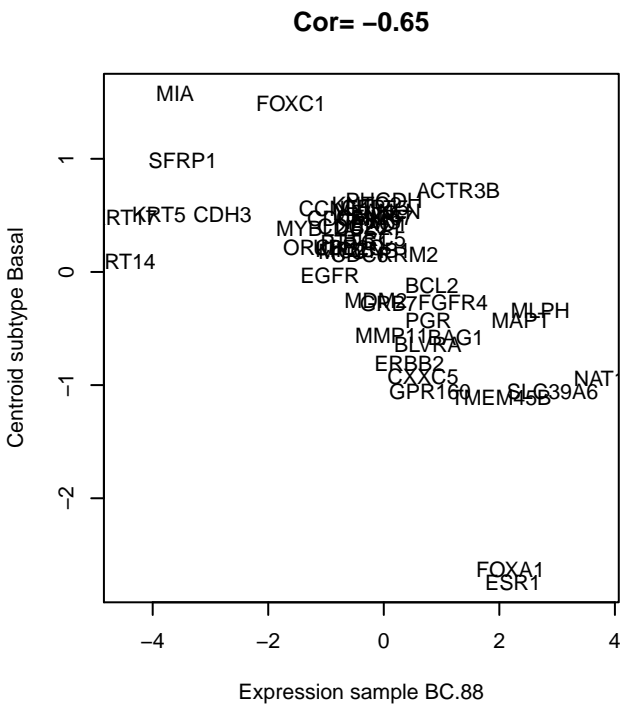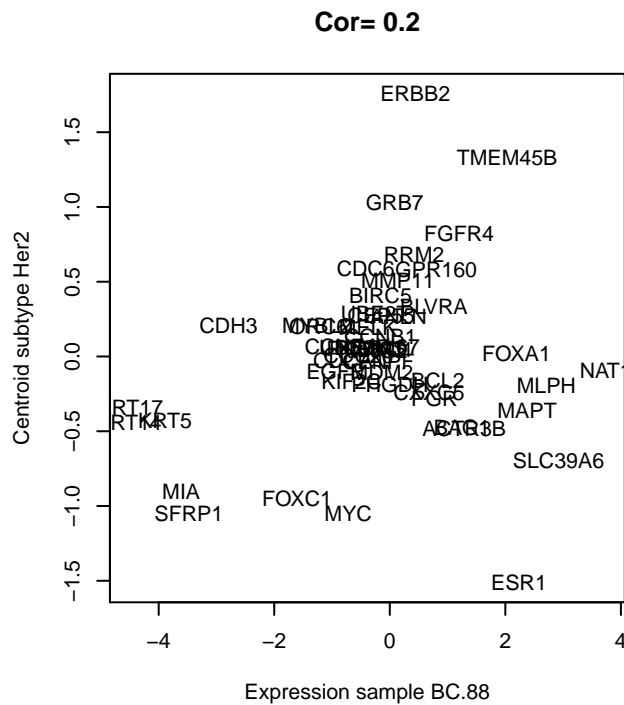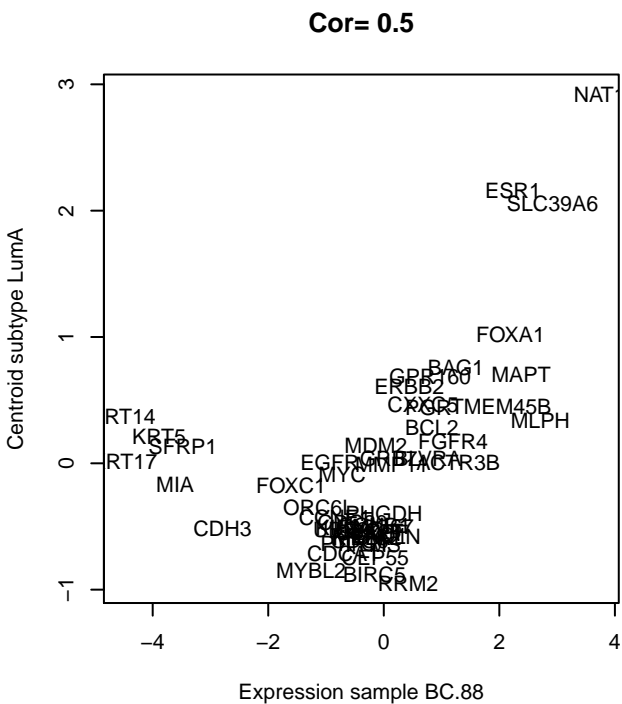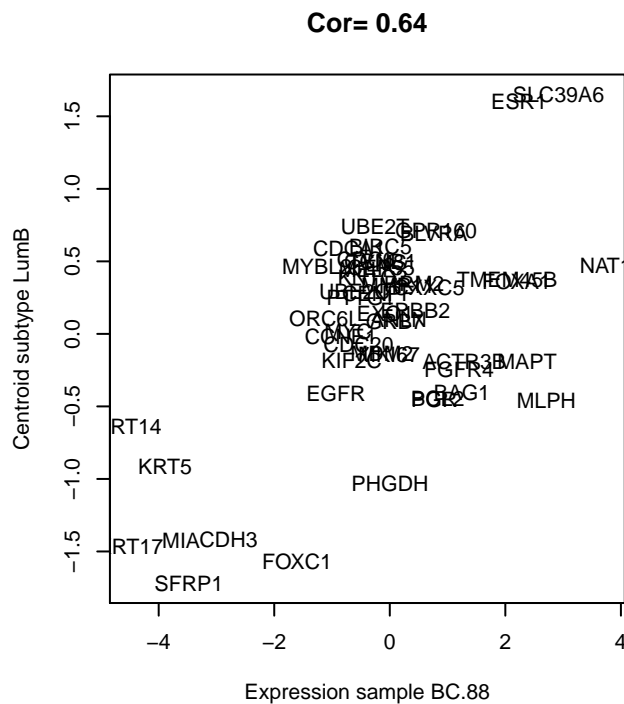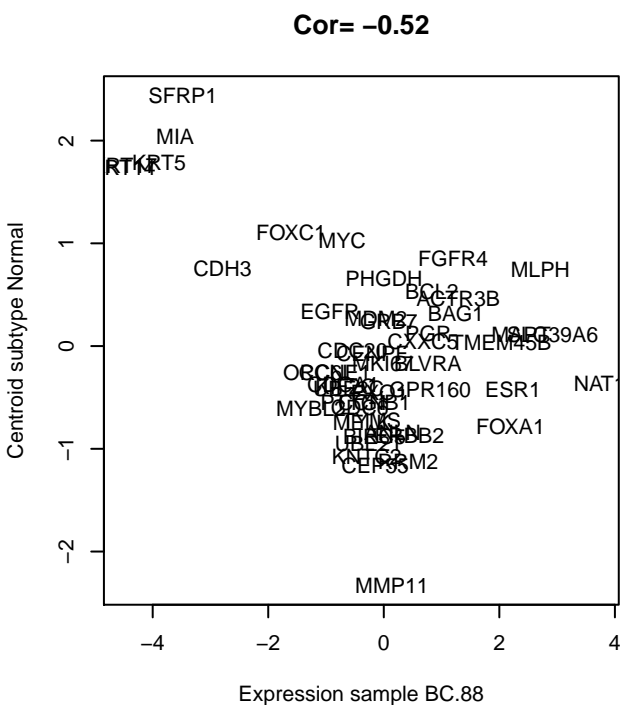





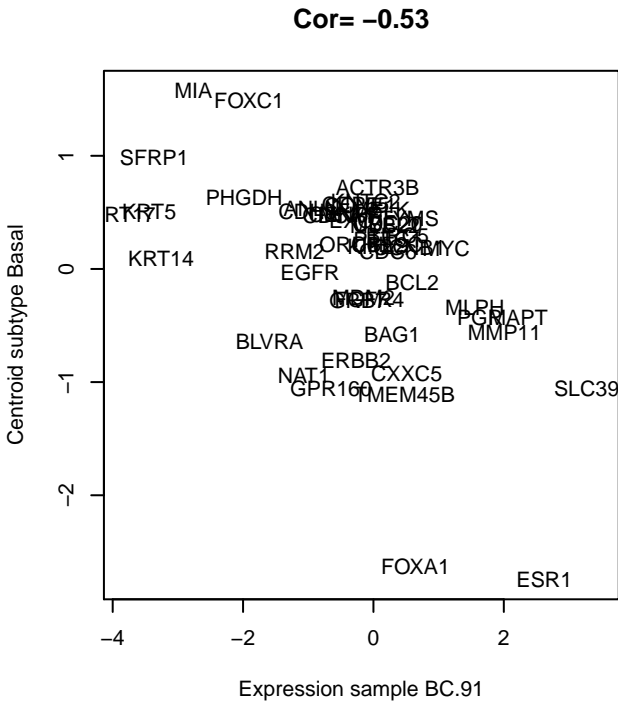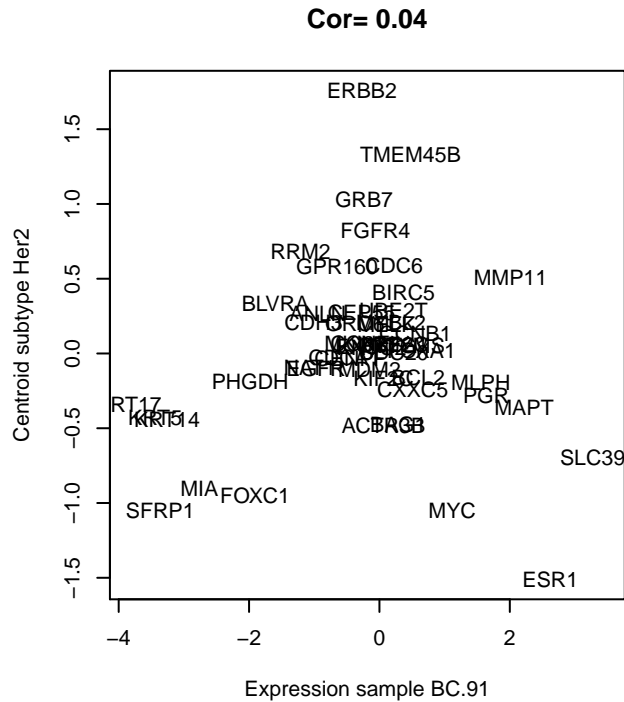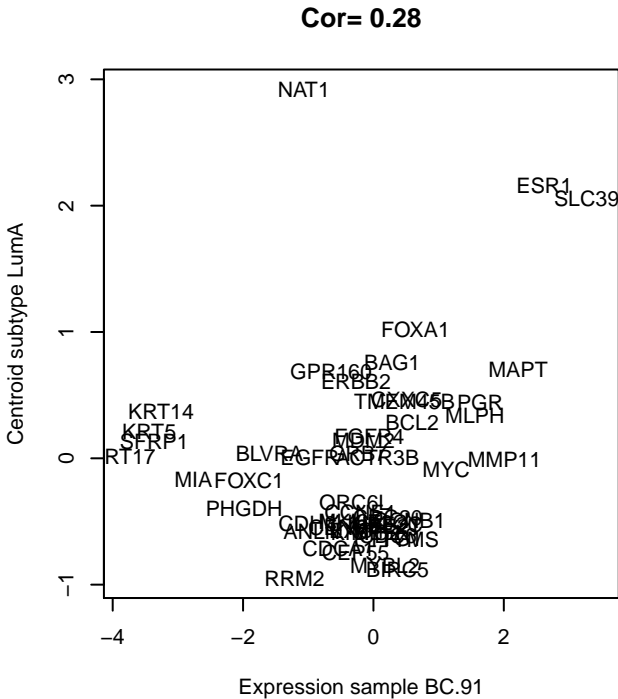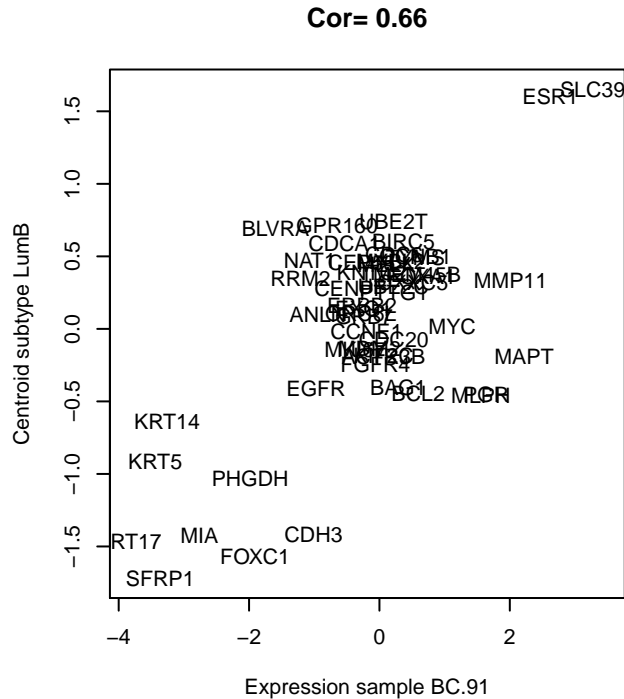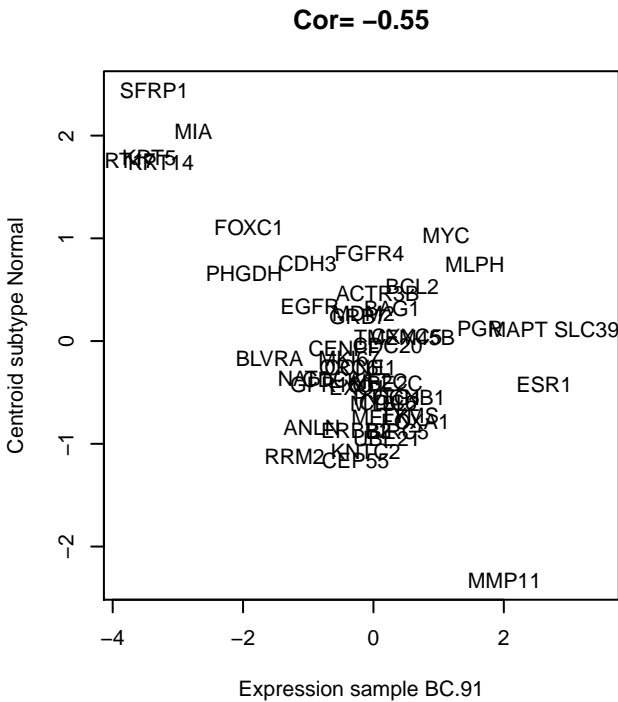

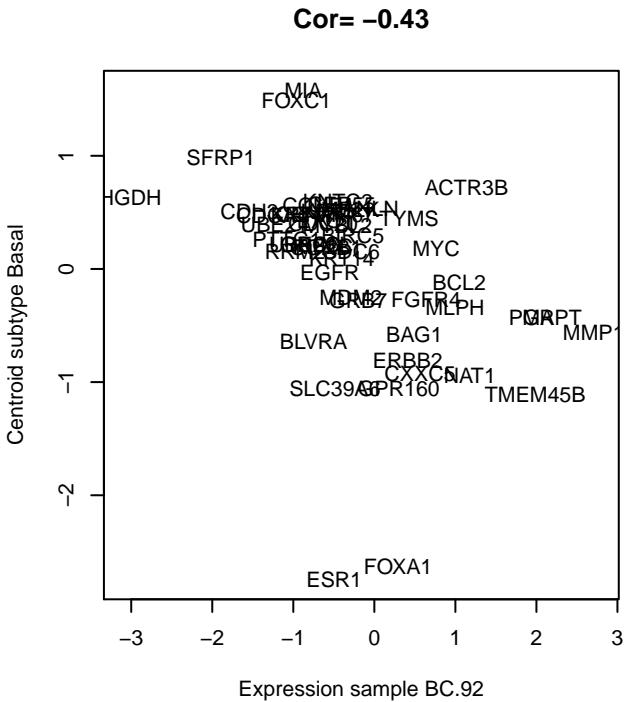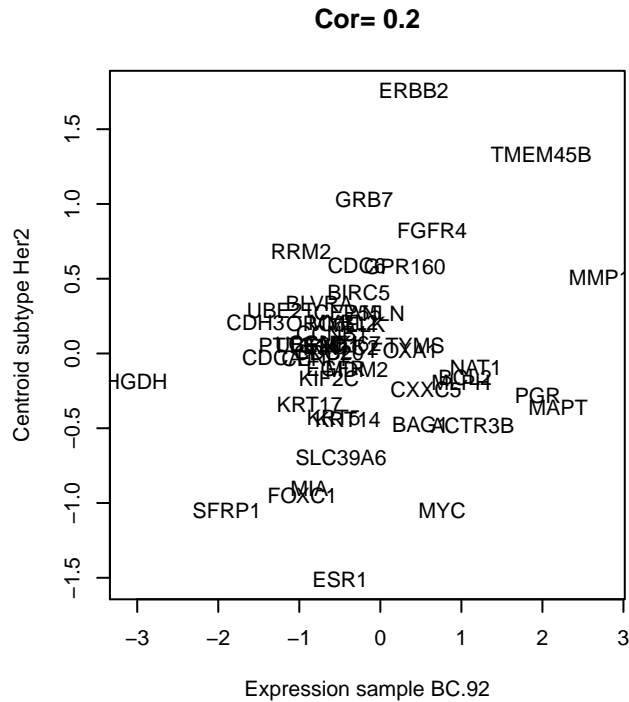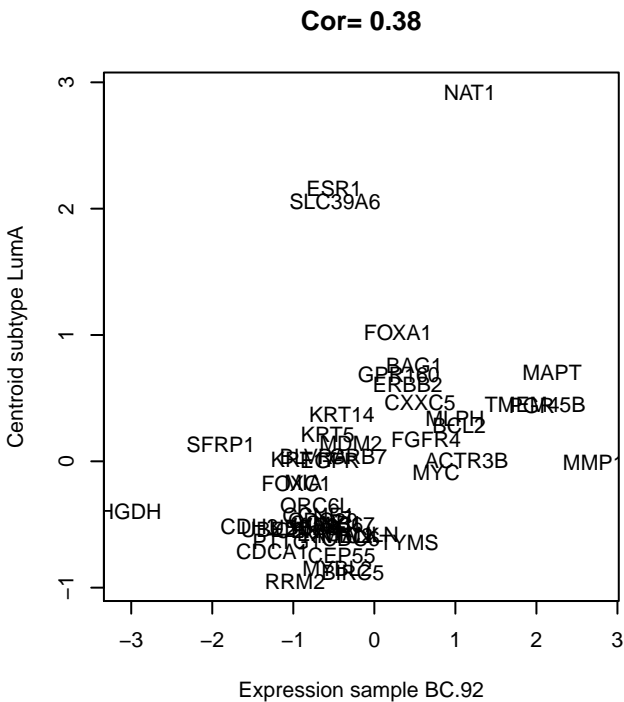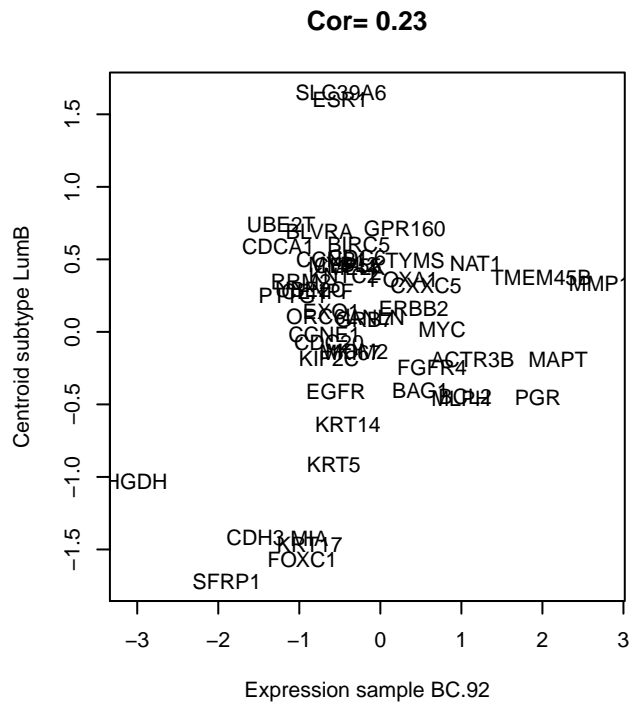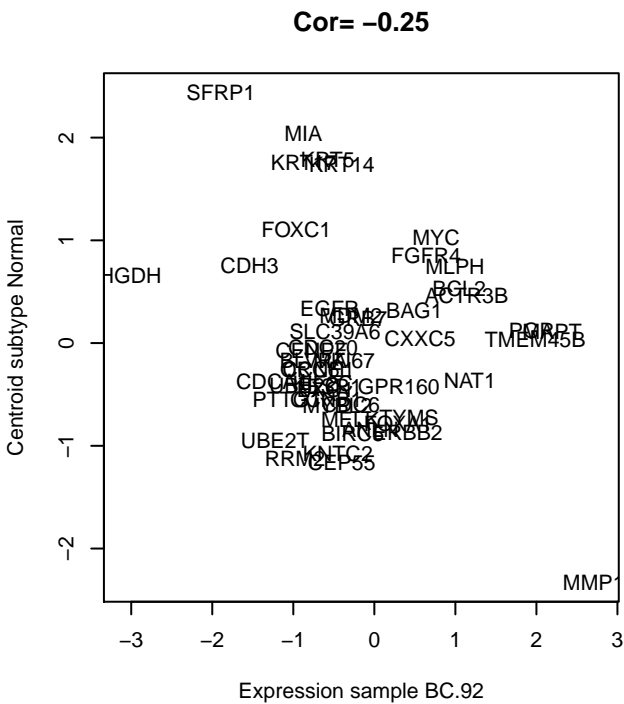



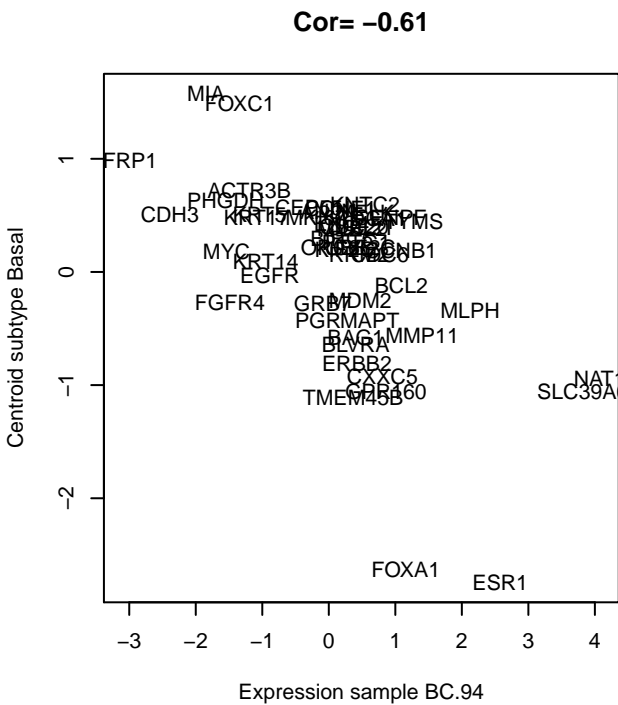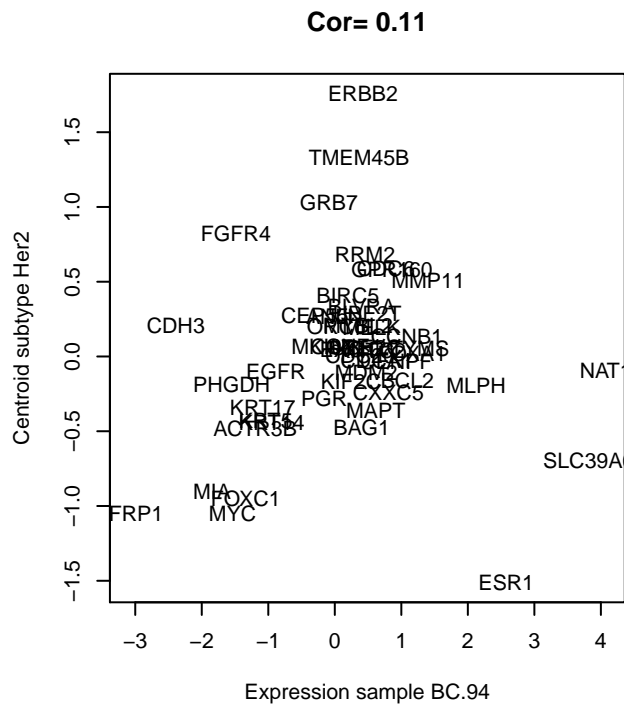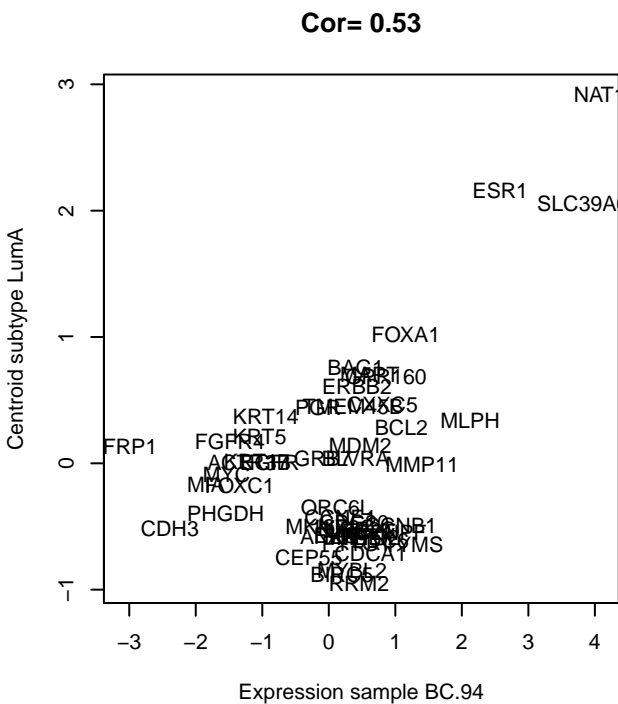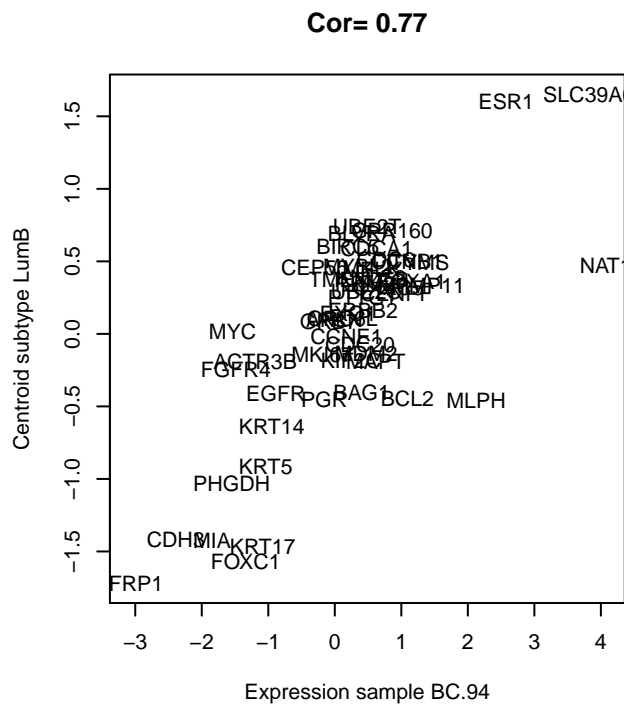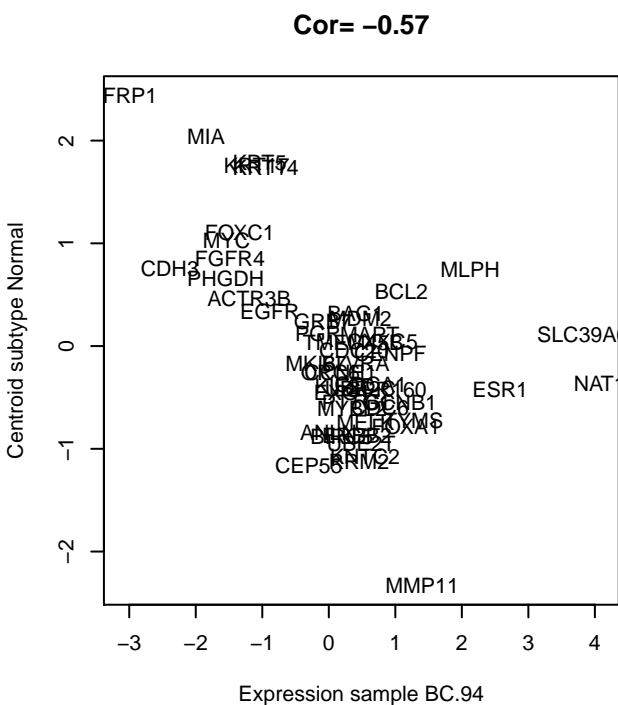

Supplement: Supplementary file 1 [file cancers-13-06118-s001.zip › SupplementaryCode/kodeTilSubmition/dataOutput/bulk/ScatterPlot_expressionPerSample_EachCentroid.pdf]

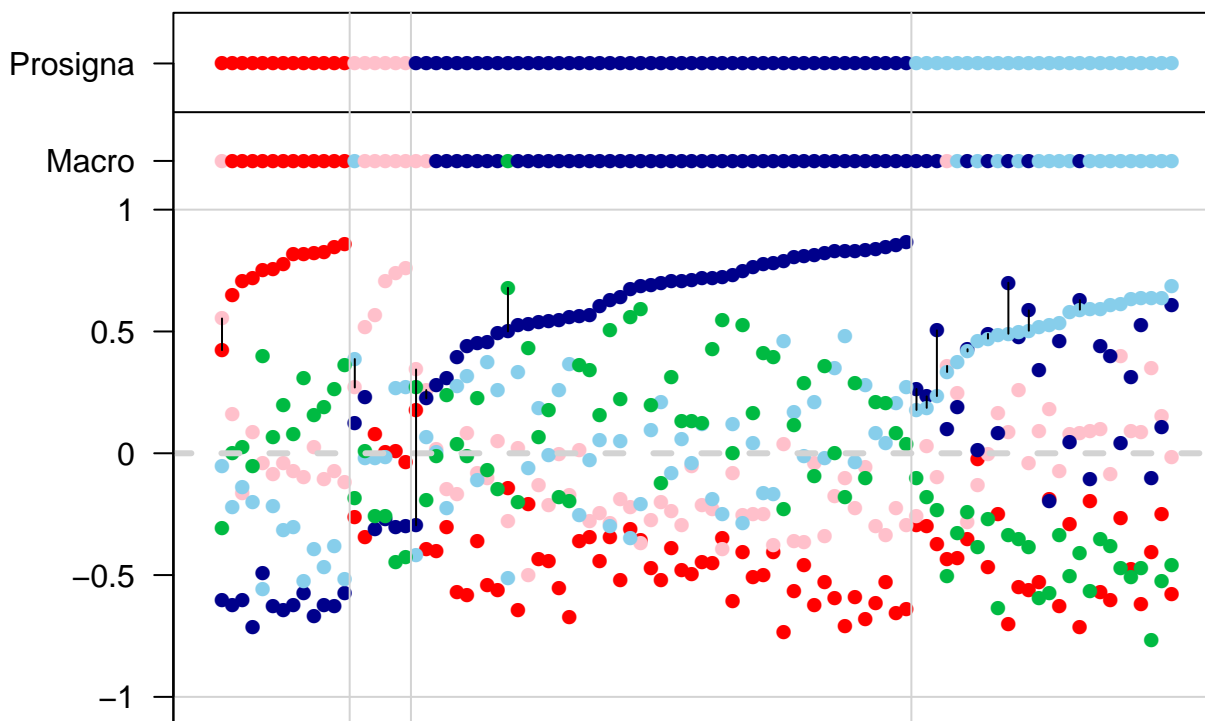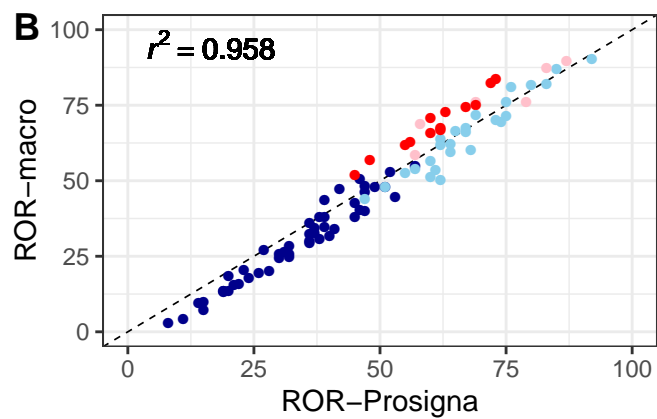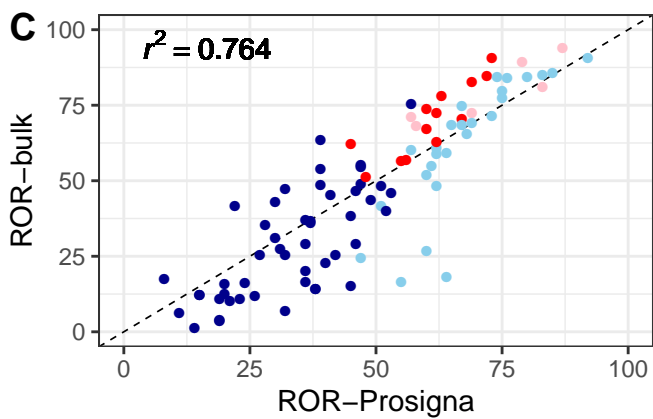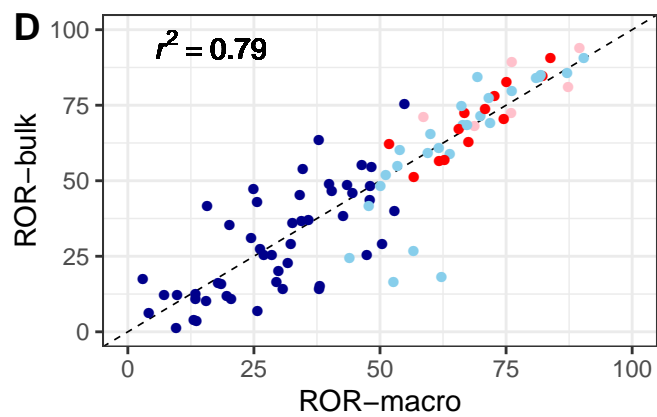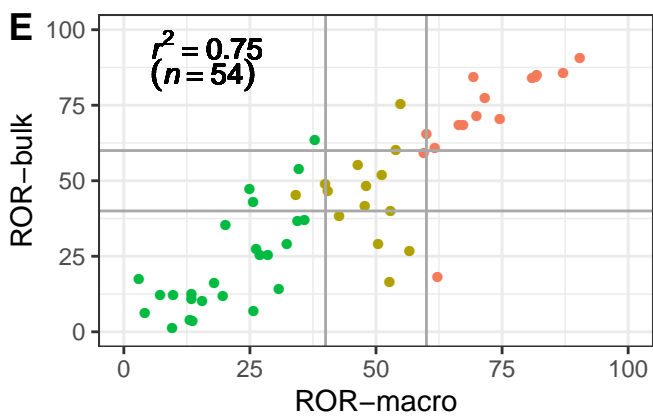

Supplement: Supplementary file 1 [file cancers-13-06118-s001.zip › SupplementaryCode/kodeTilSubmition/dataOutput/Fig2.pdf]

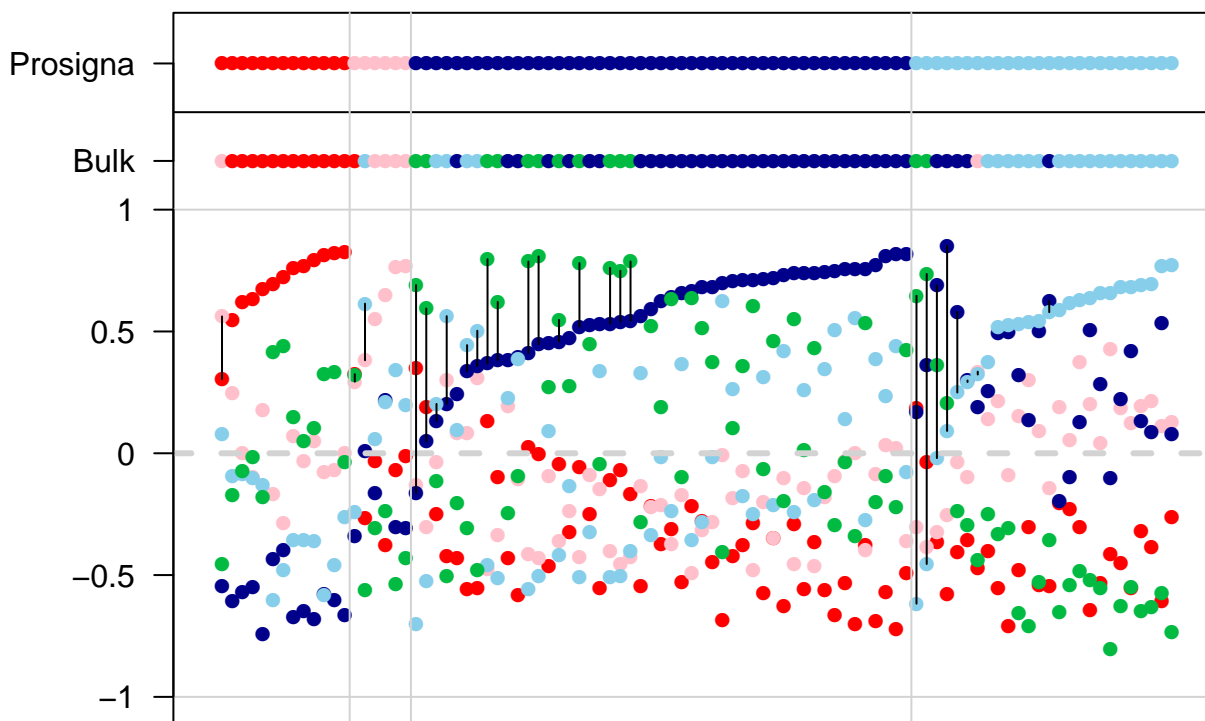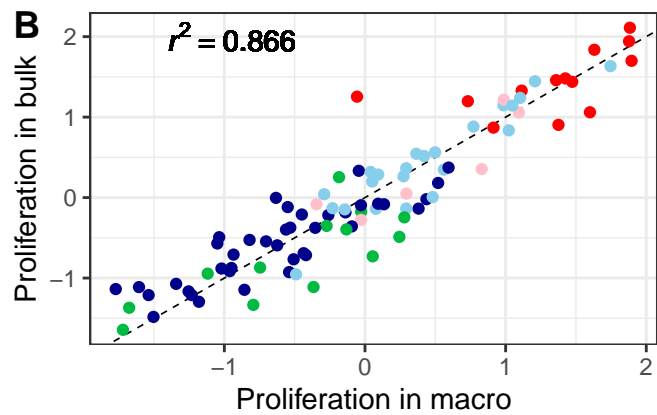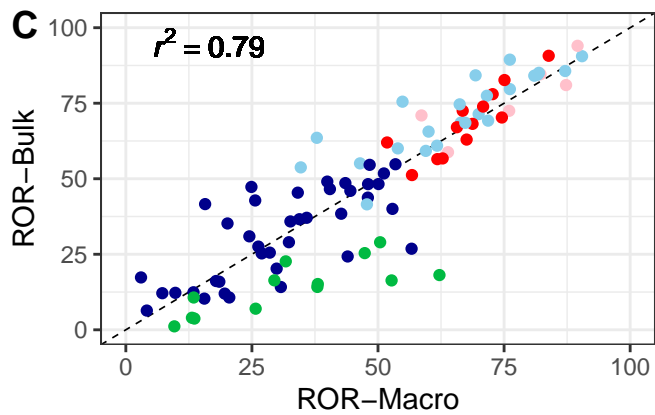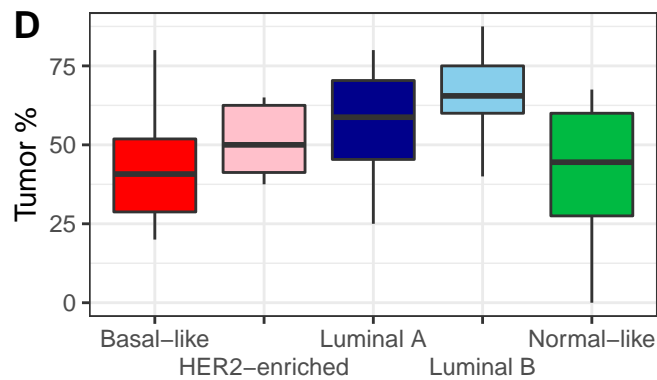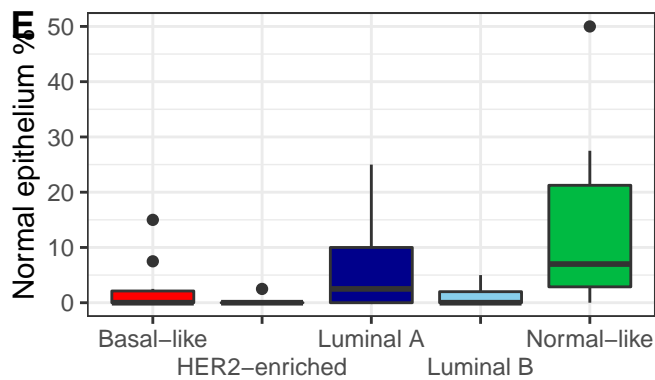

Supplement: Supplementary file 1 [file cancers-13-06118-s001.zip › SupplementaryCode/kodeTilSubmition/dataOutput/Fig3.pdf]

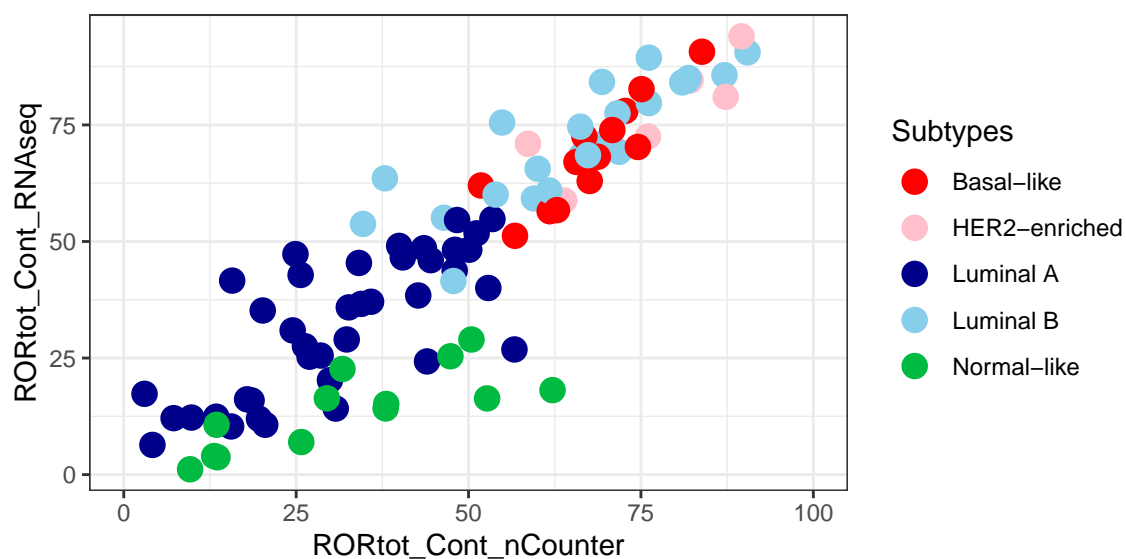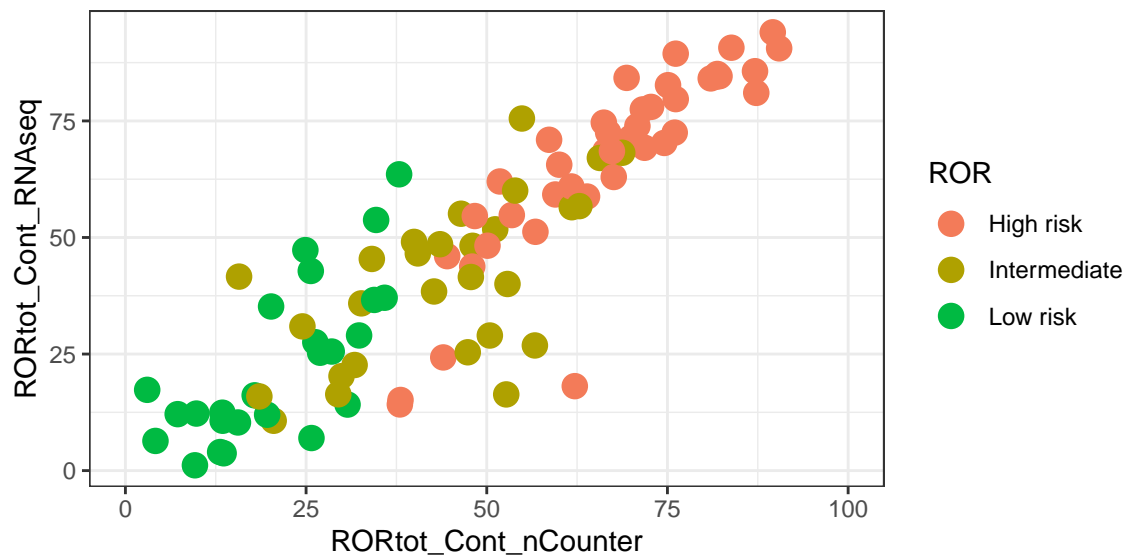

Supplement: Supplementary file 1 [file cancers-13-06118-s001.zip › SupplementaryCode/kodeTilSubmition/dataOutput/legends.pdf]

# ESR1 with ER cut off at 8.71

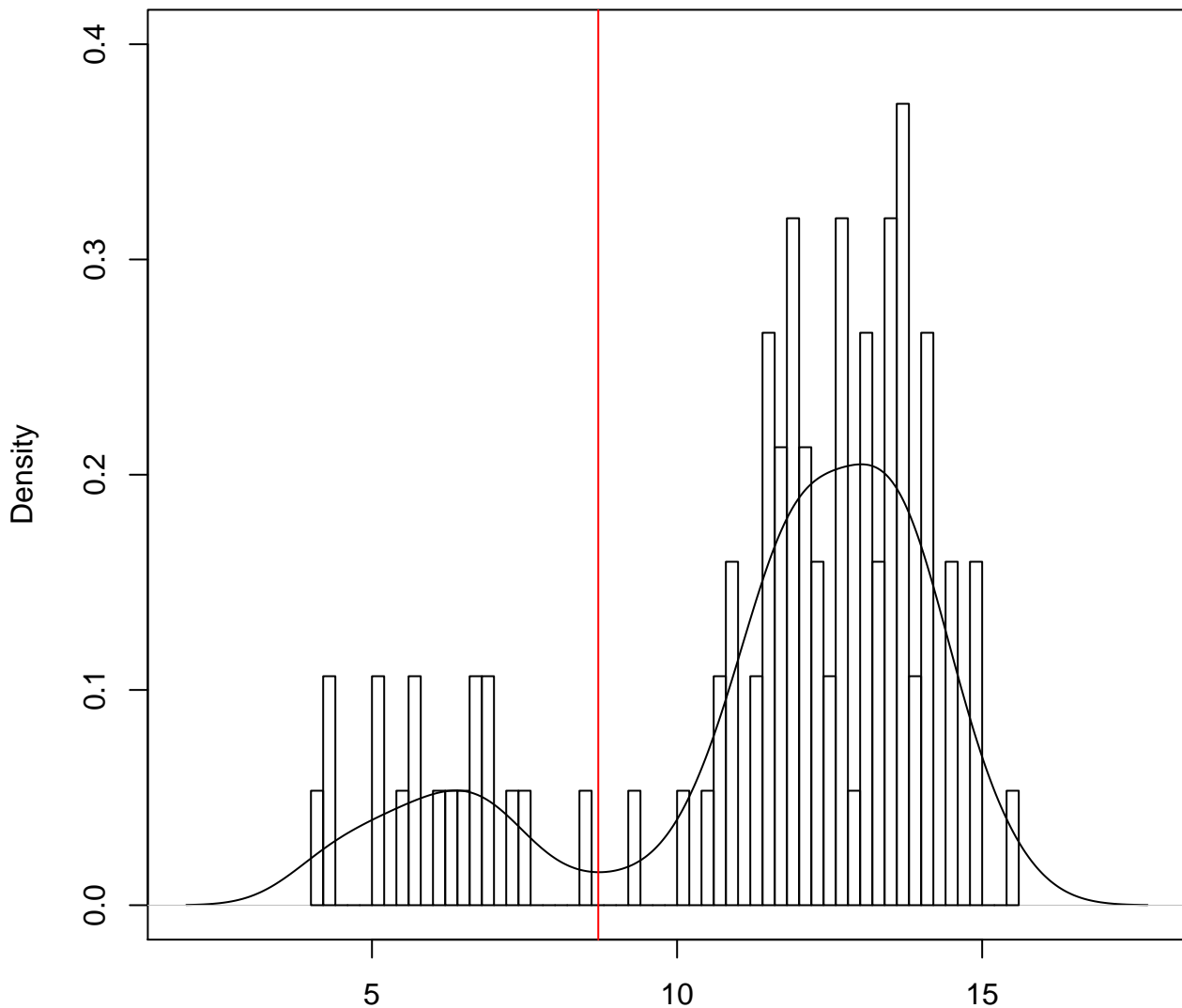

Supplement: Supplementary file 1 [file cancers-13-06118-s001.zip › SupplementaryCode/kodeTilSubmition/dataOutput/macro/distribution_ESR1.pdf]

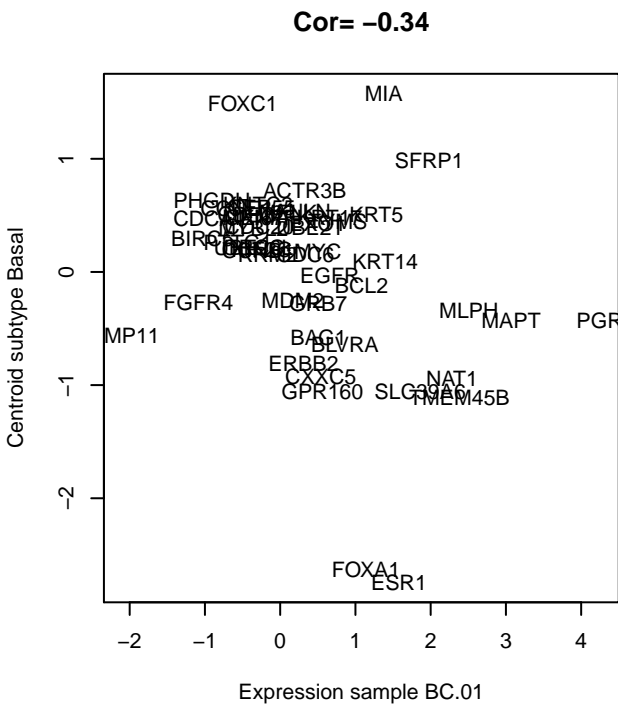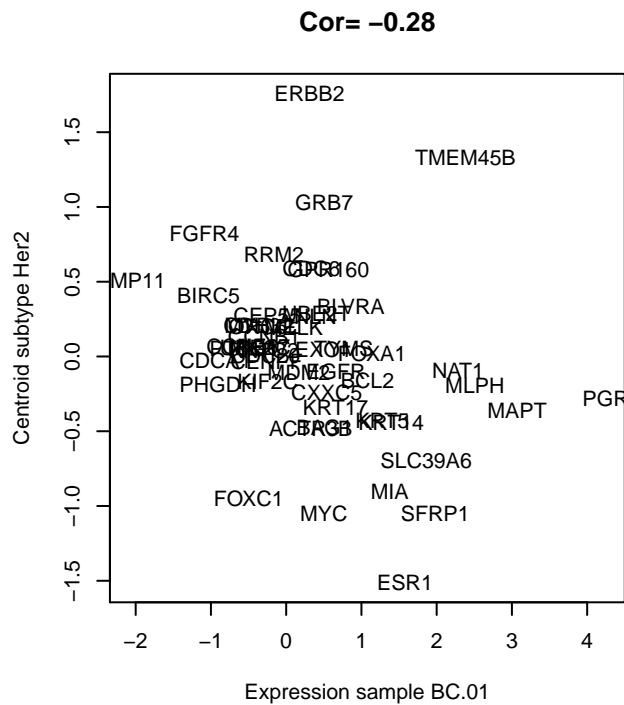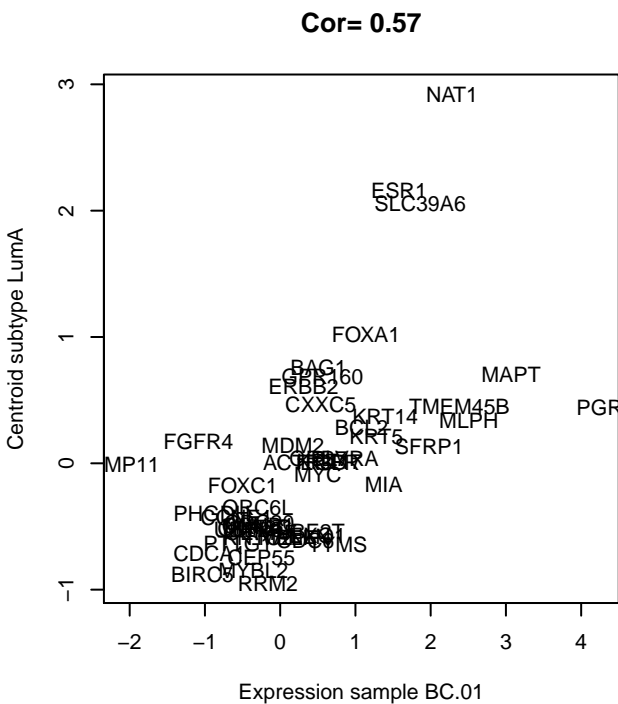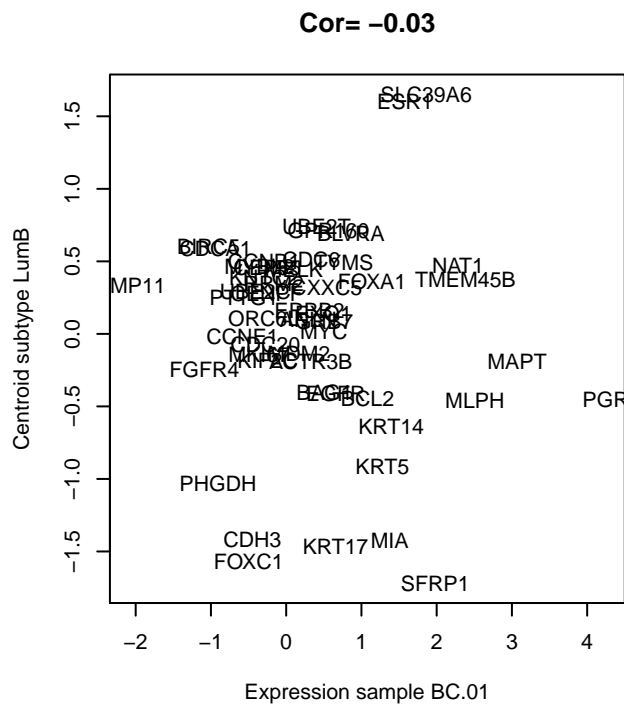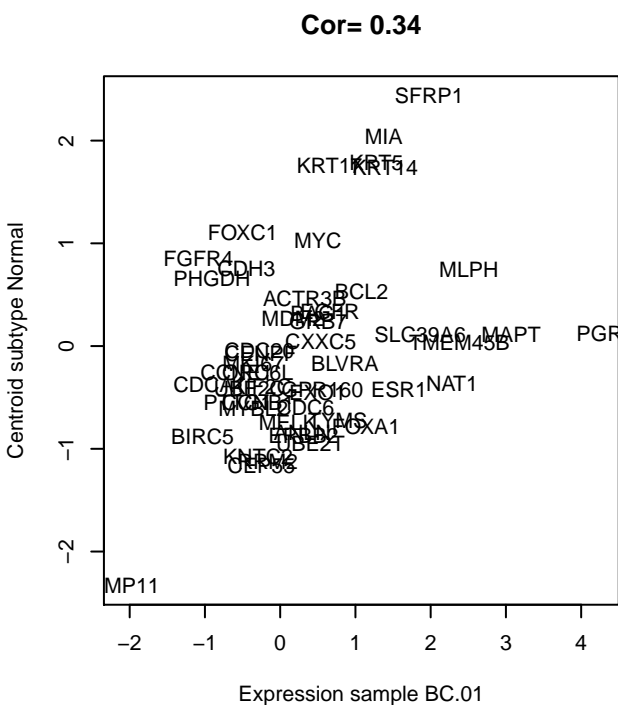



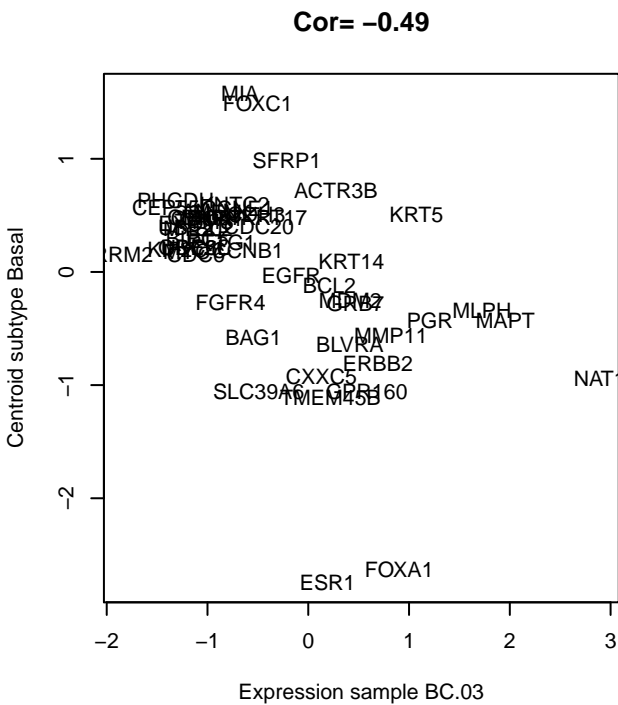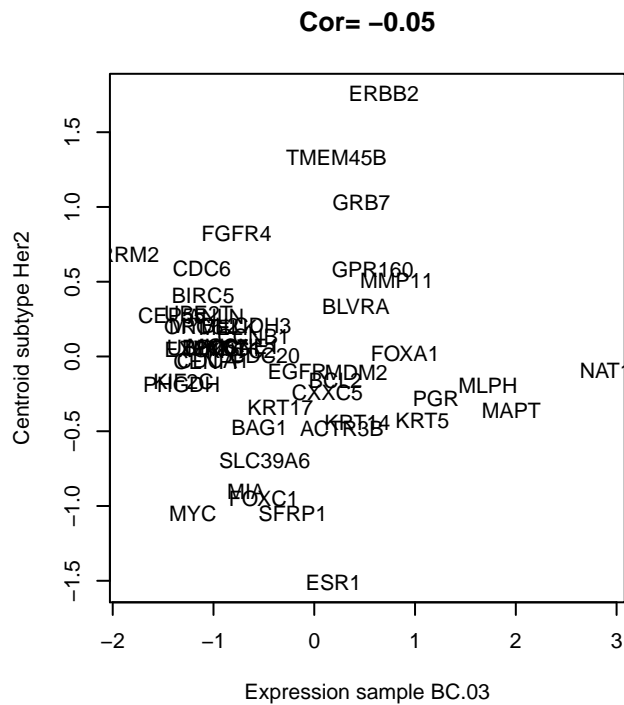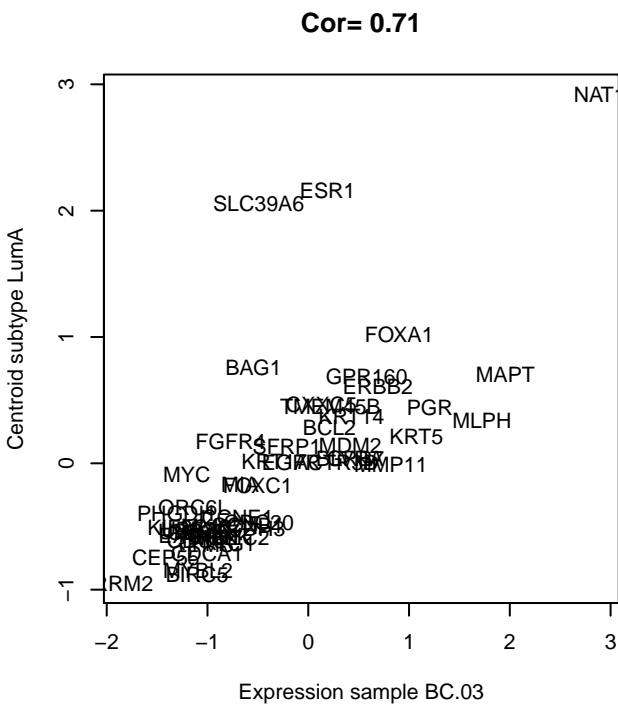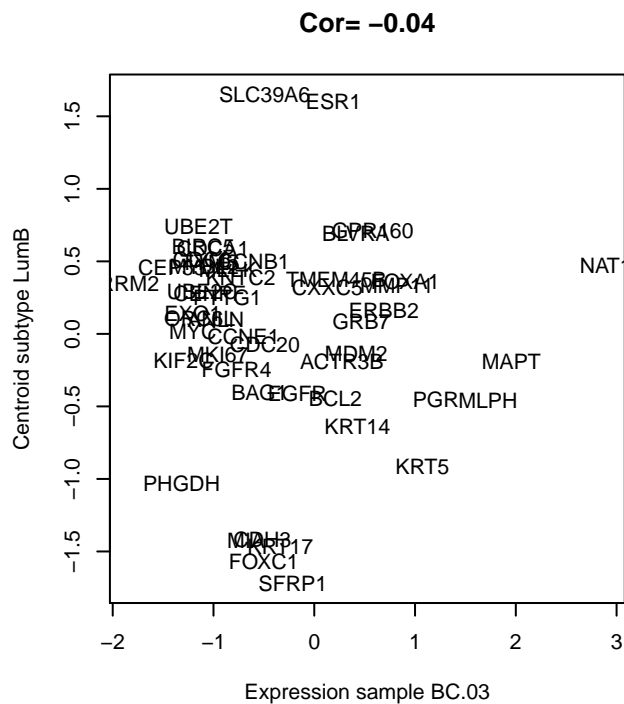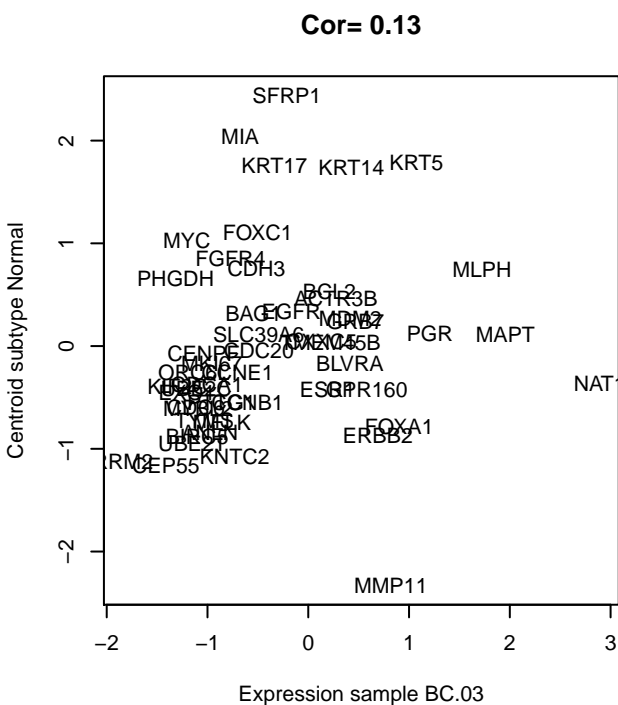

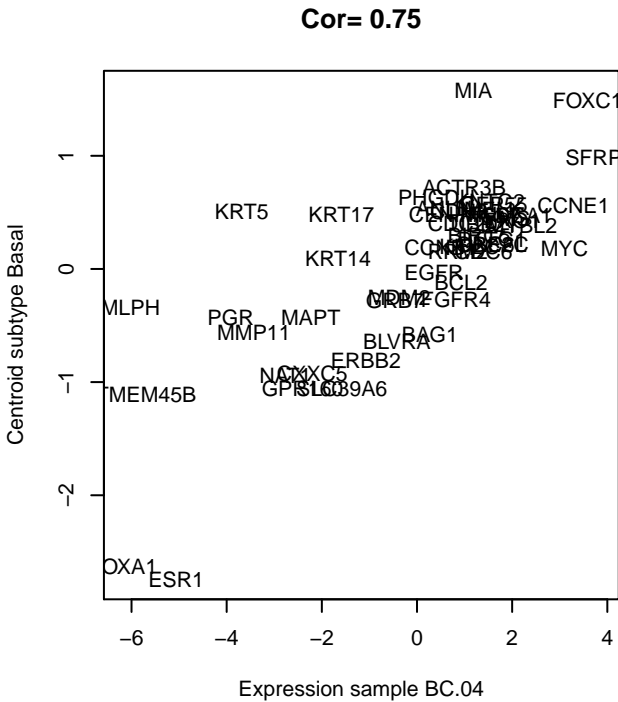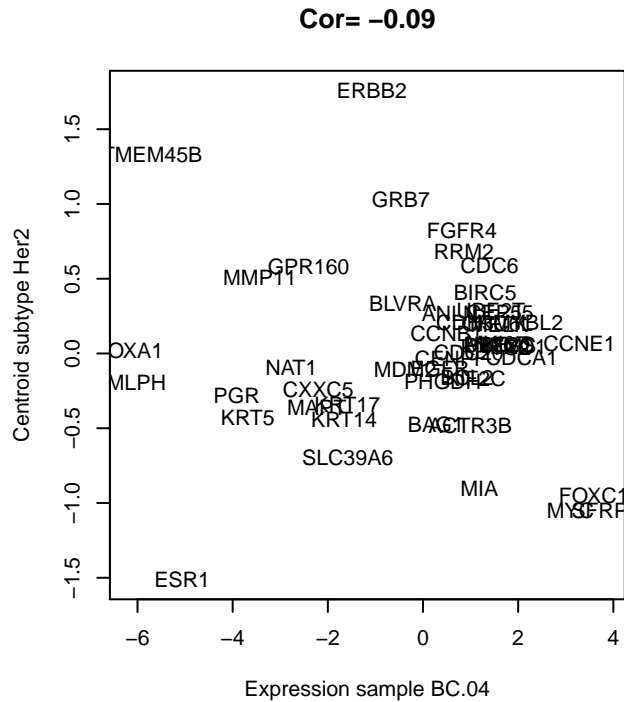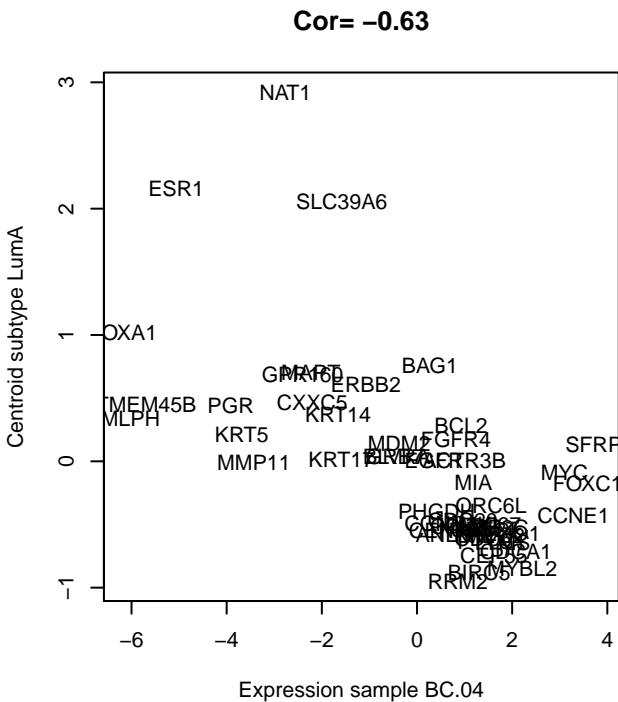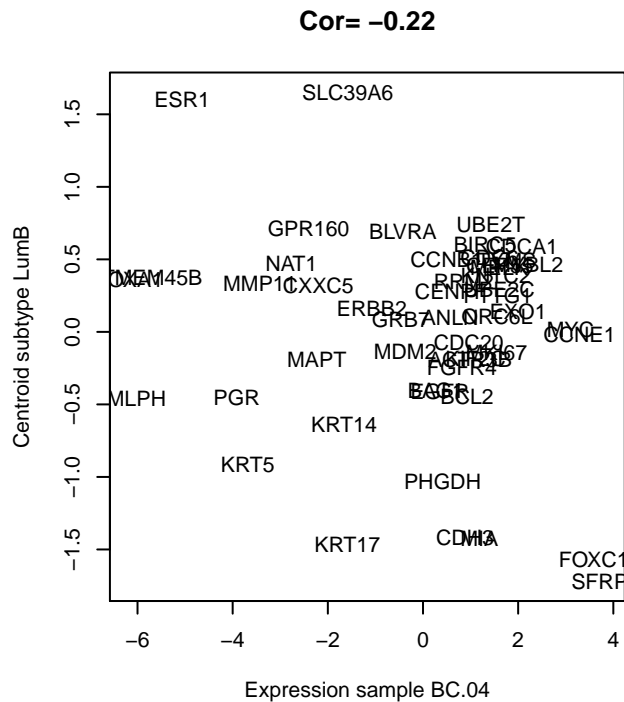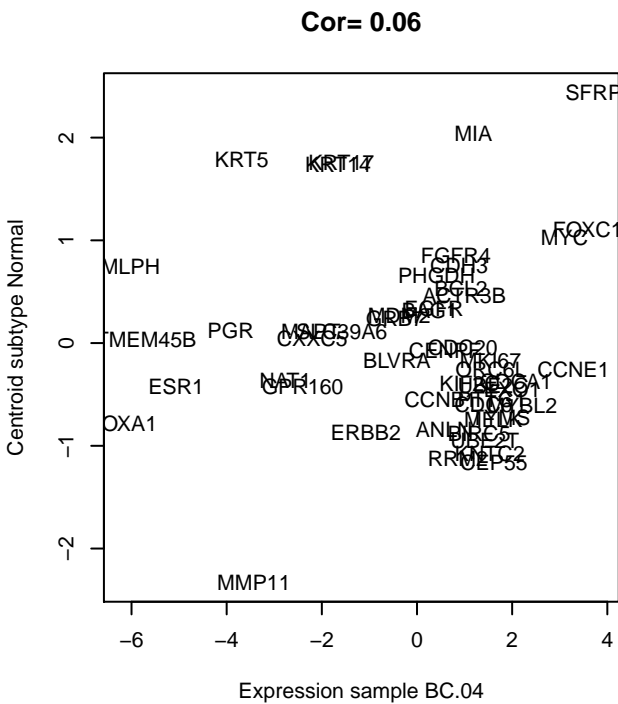

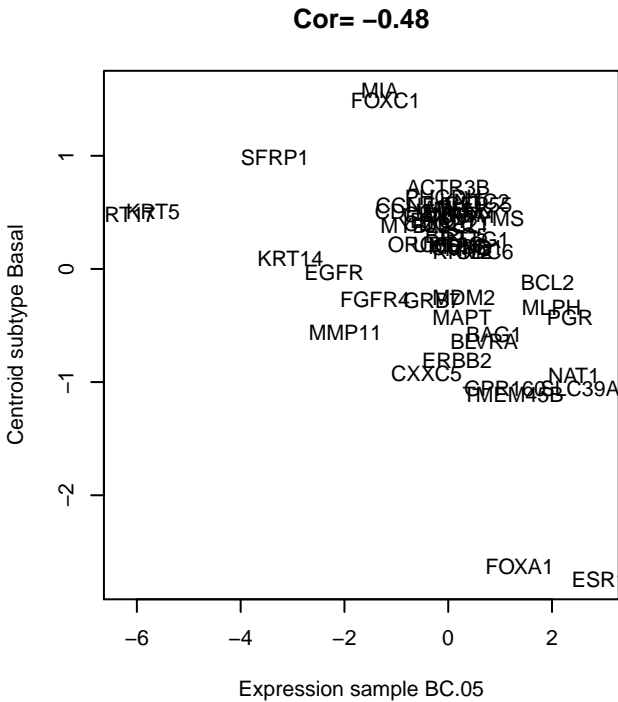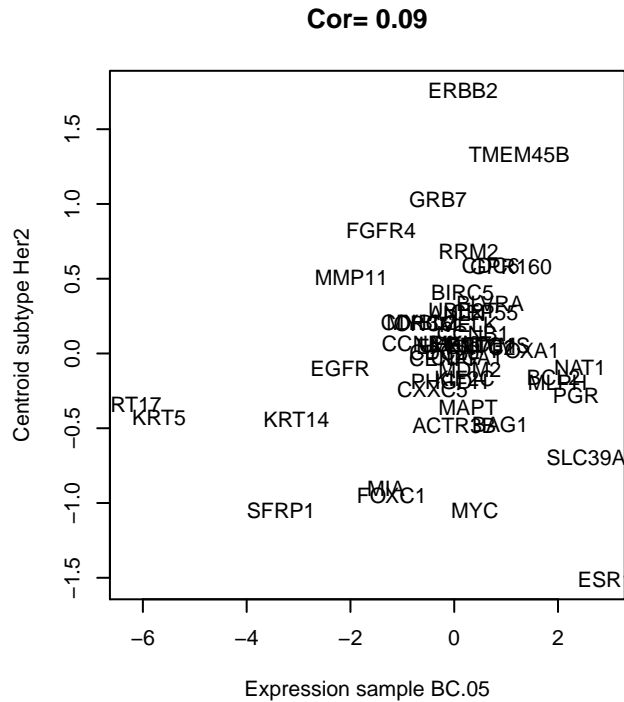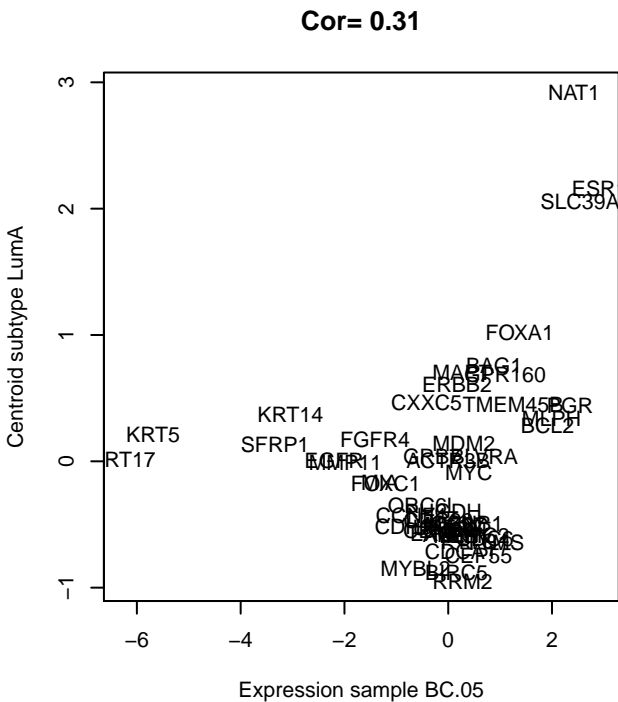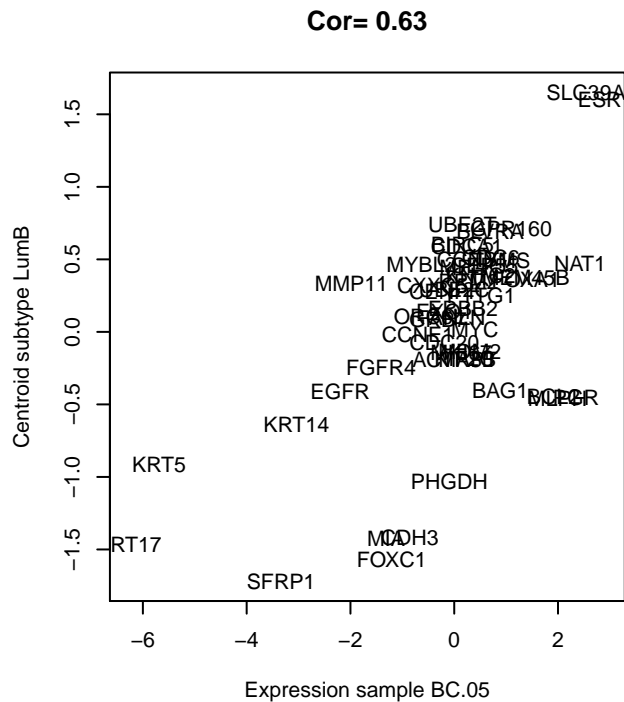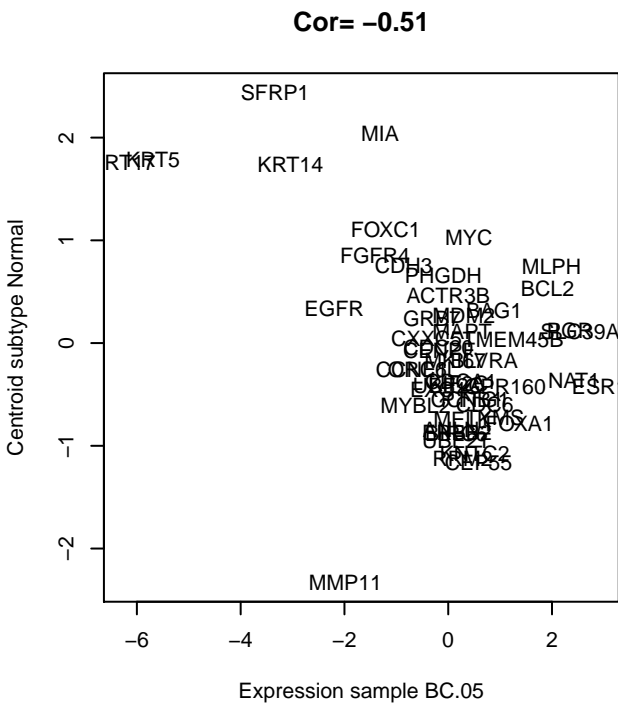

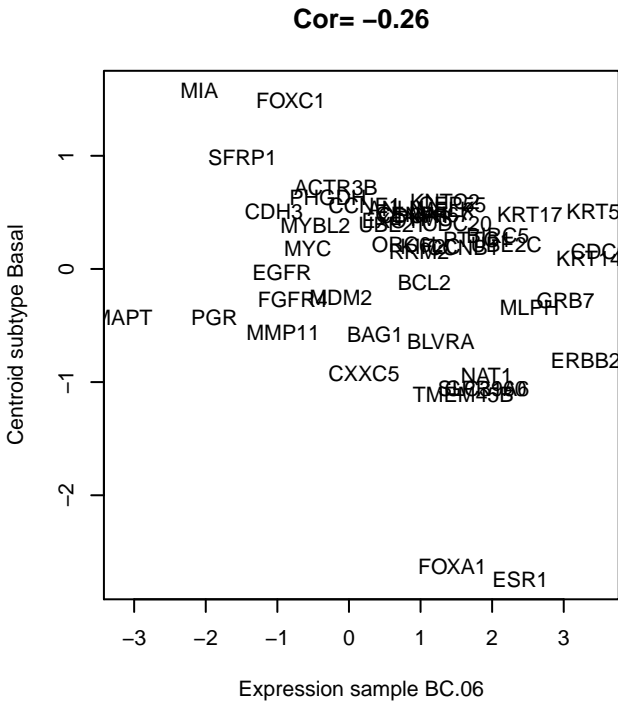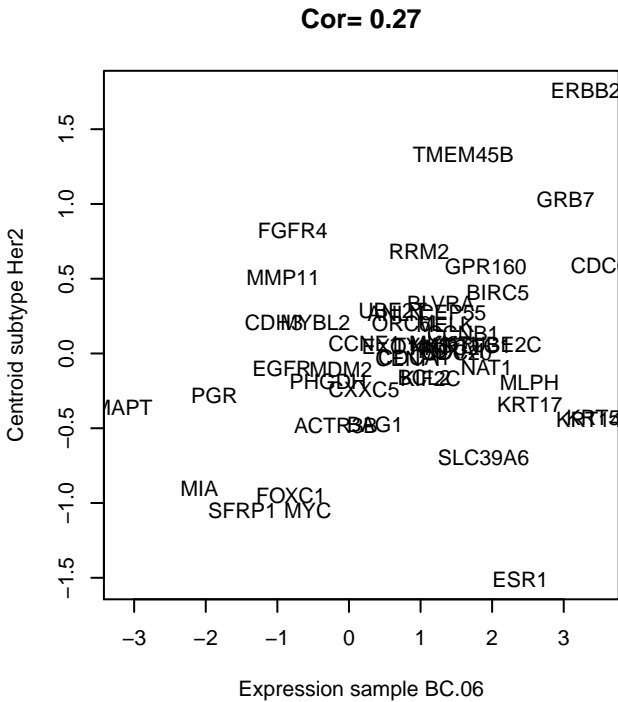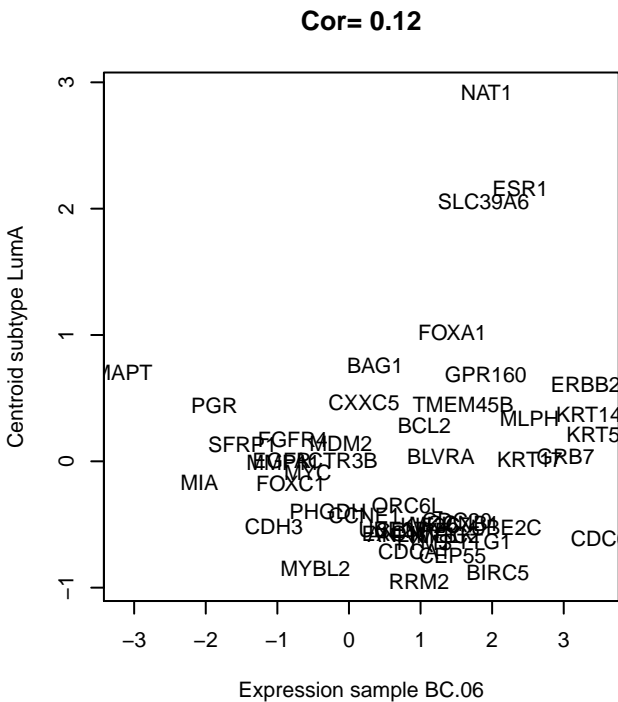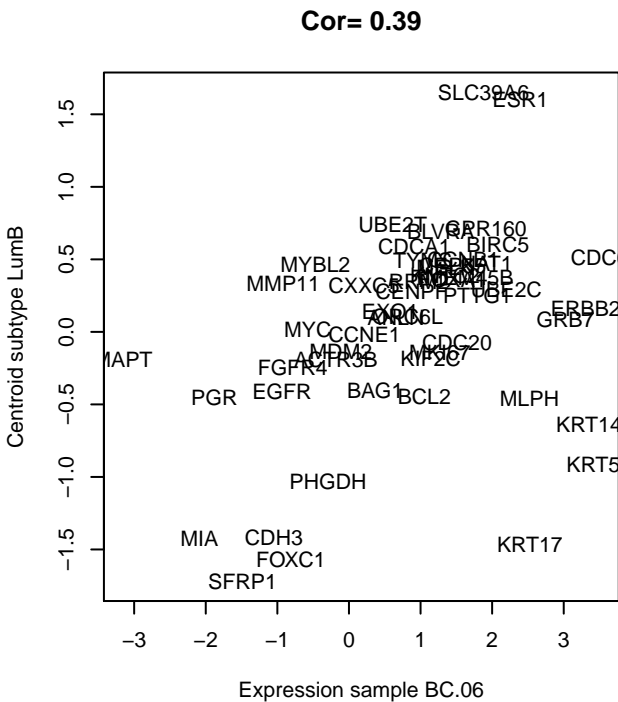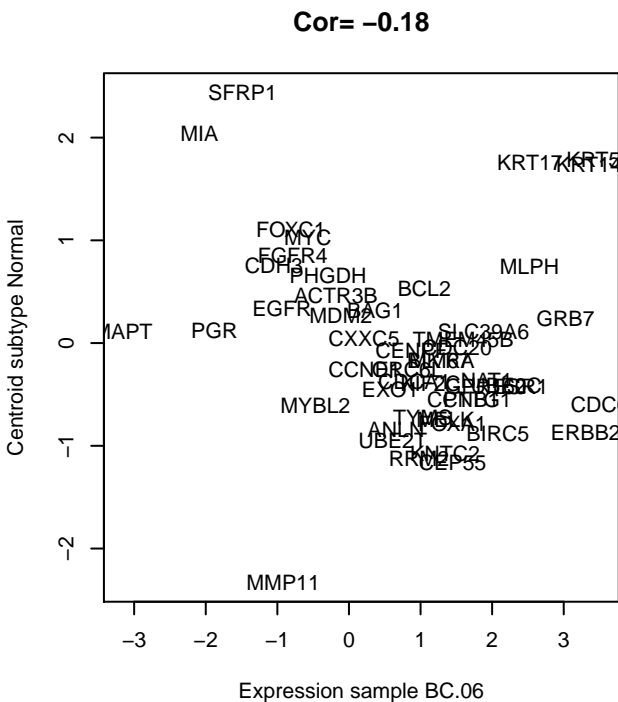

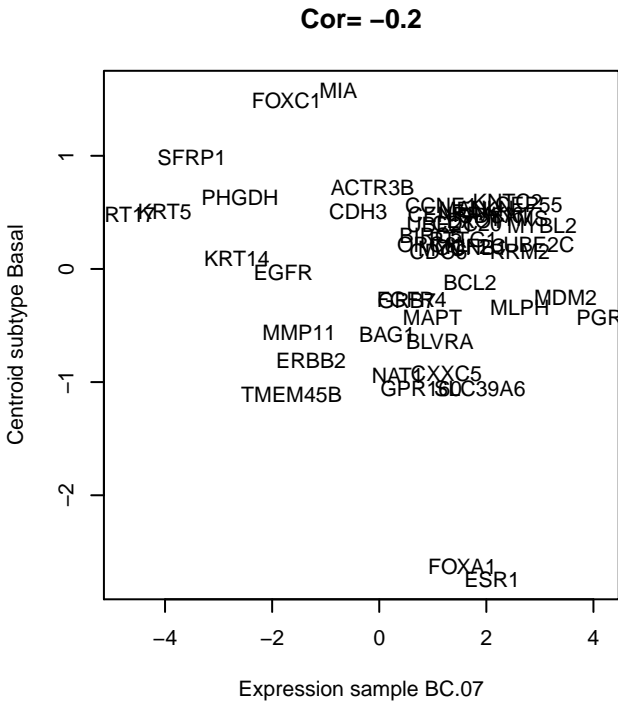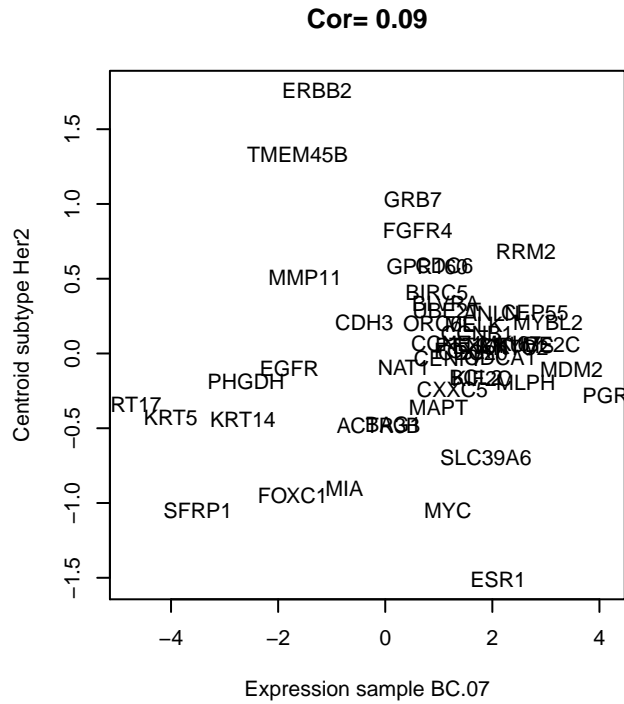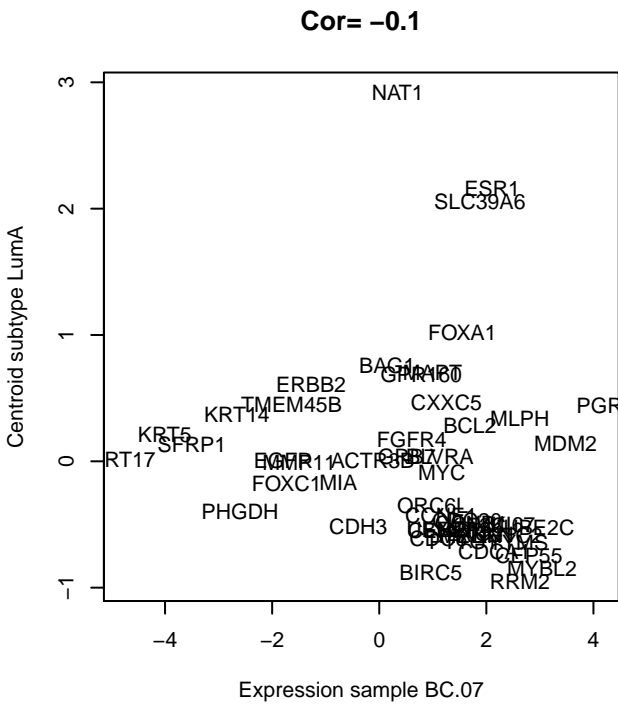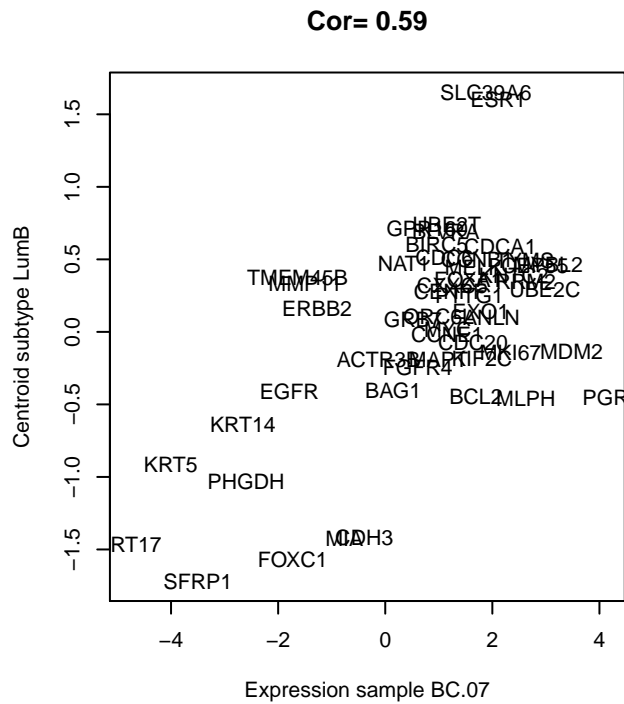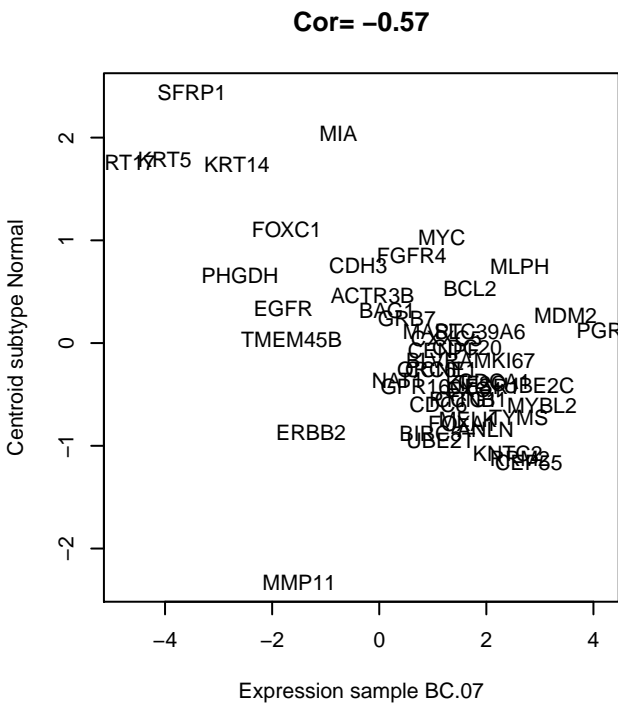

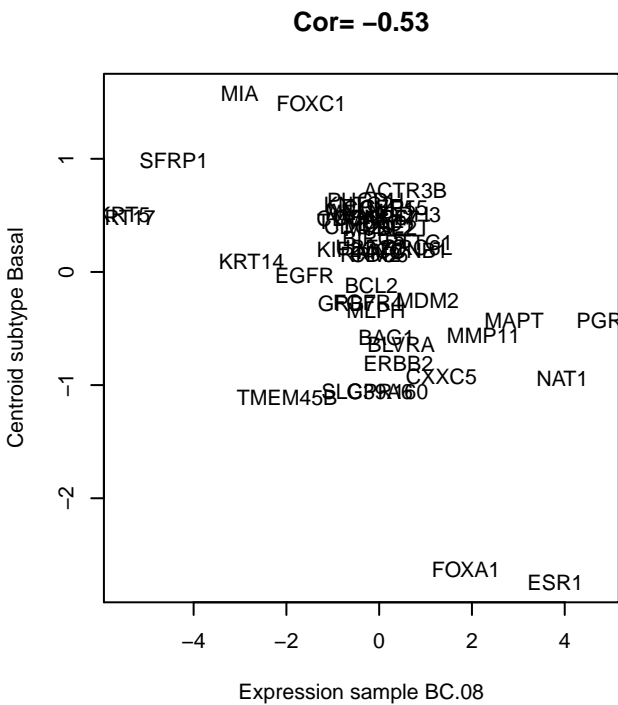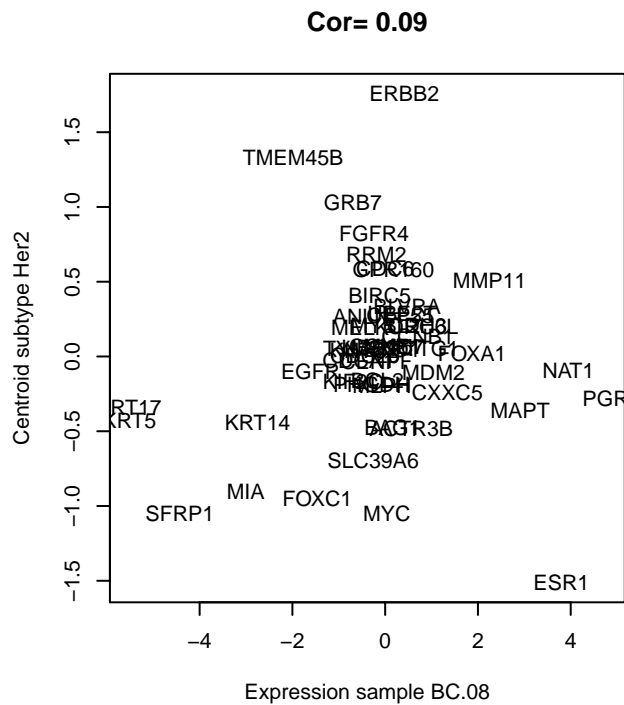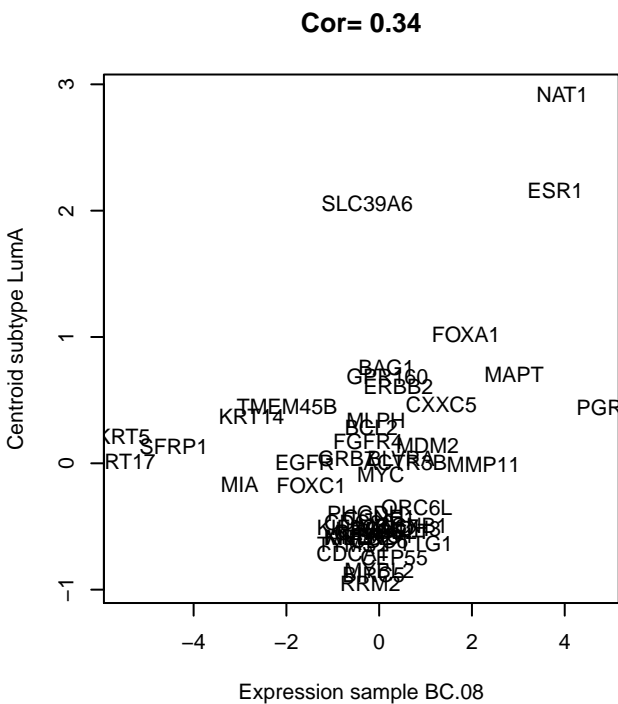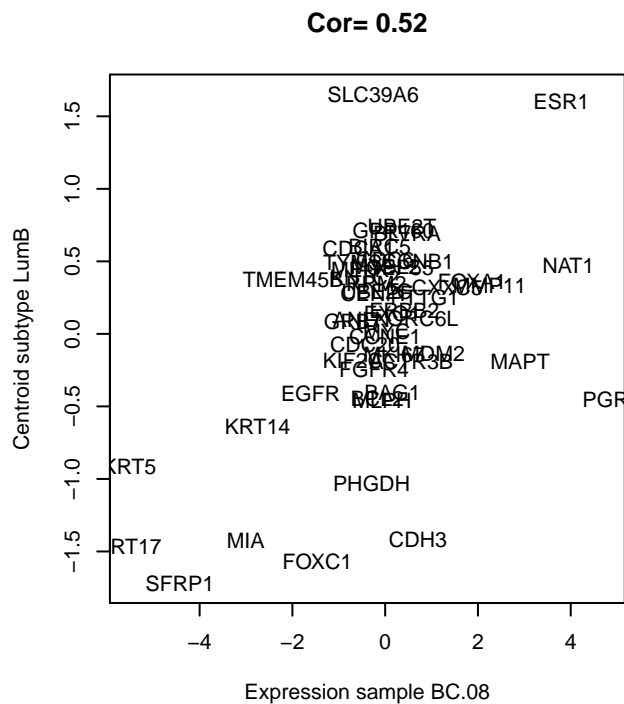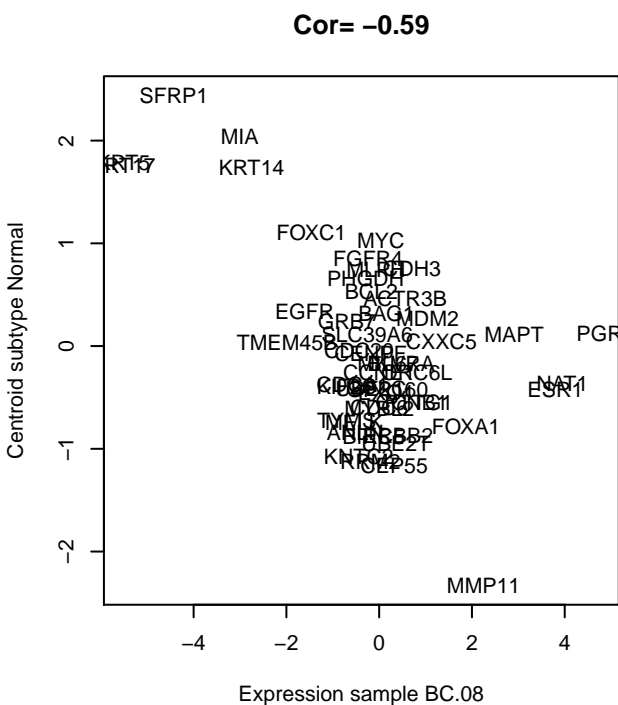

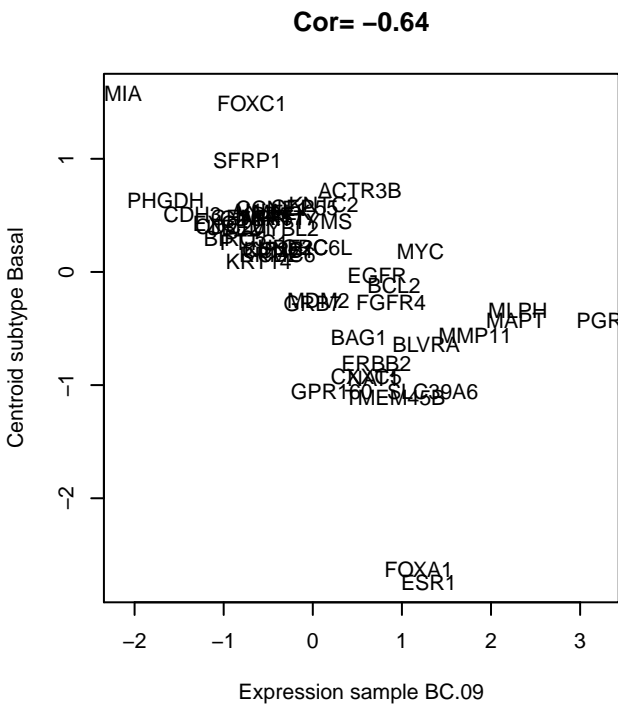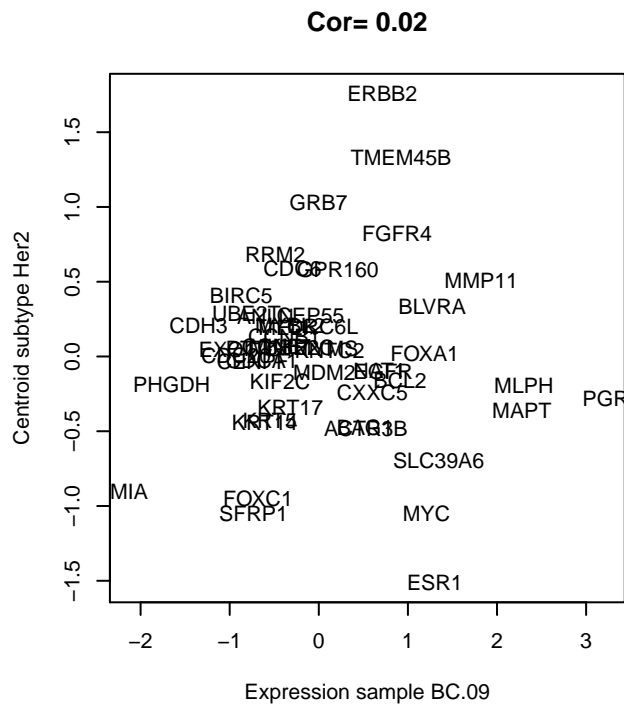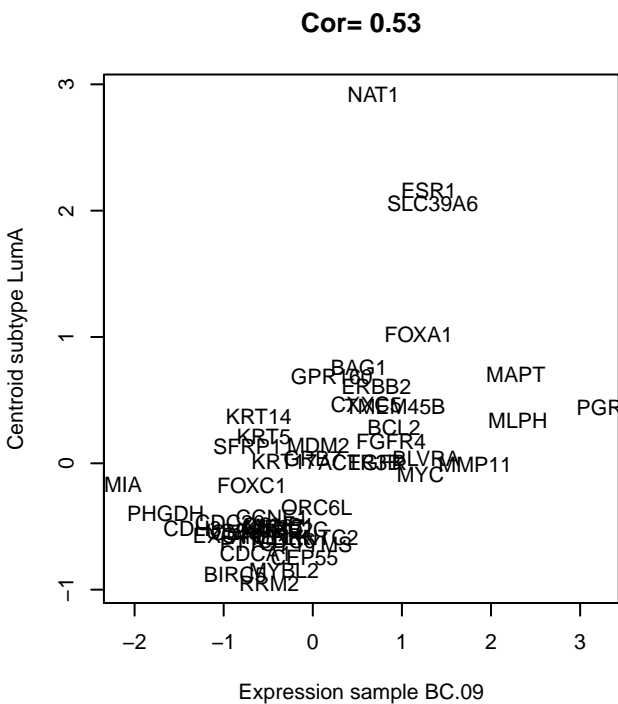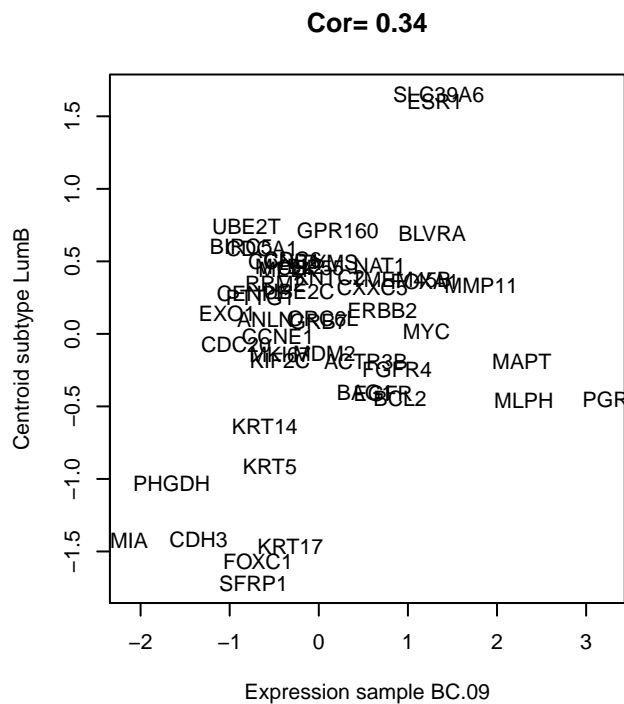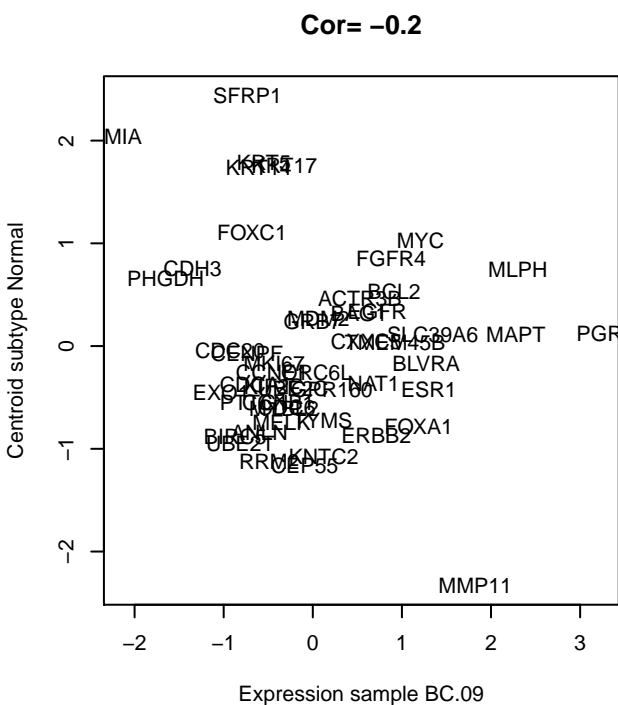

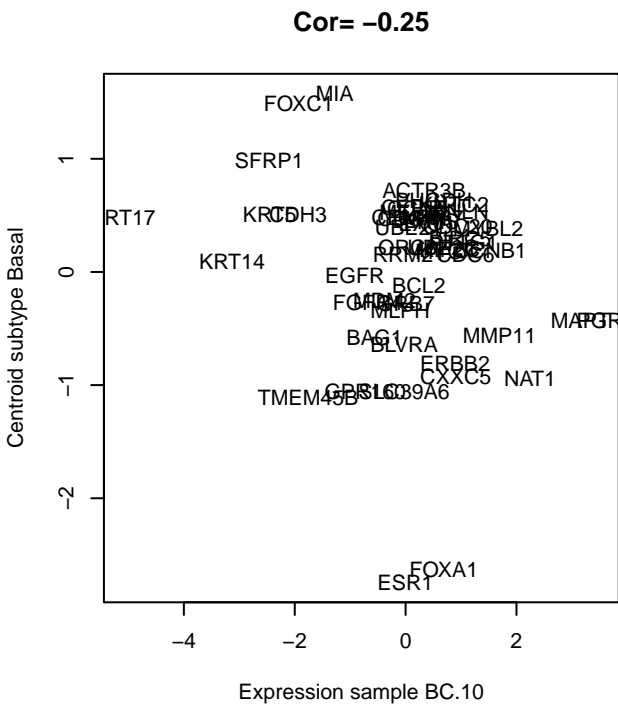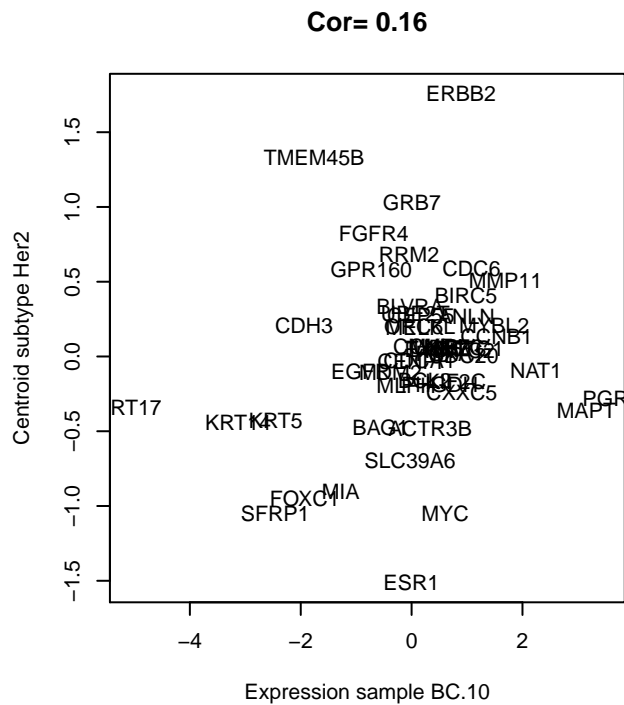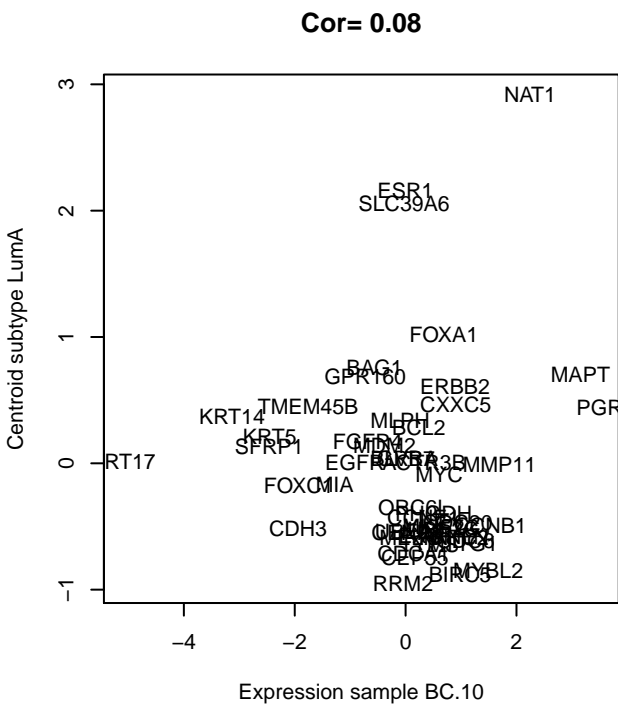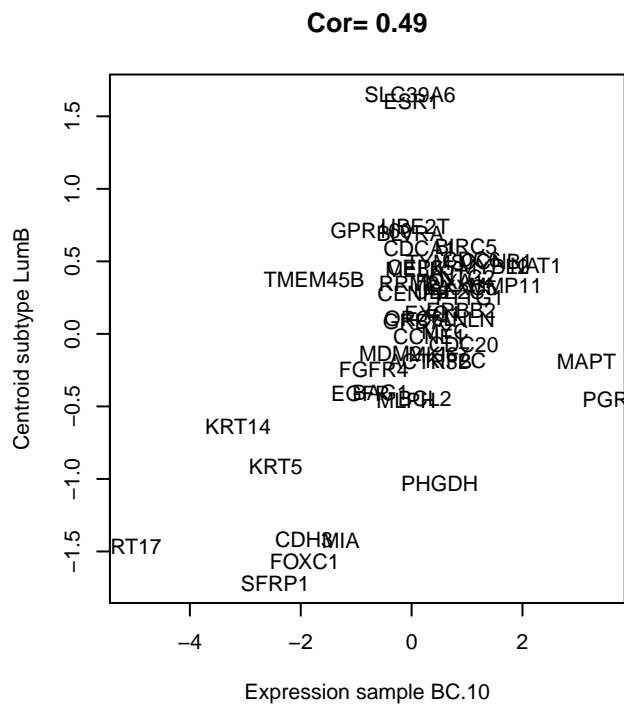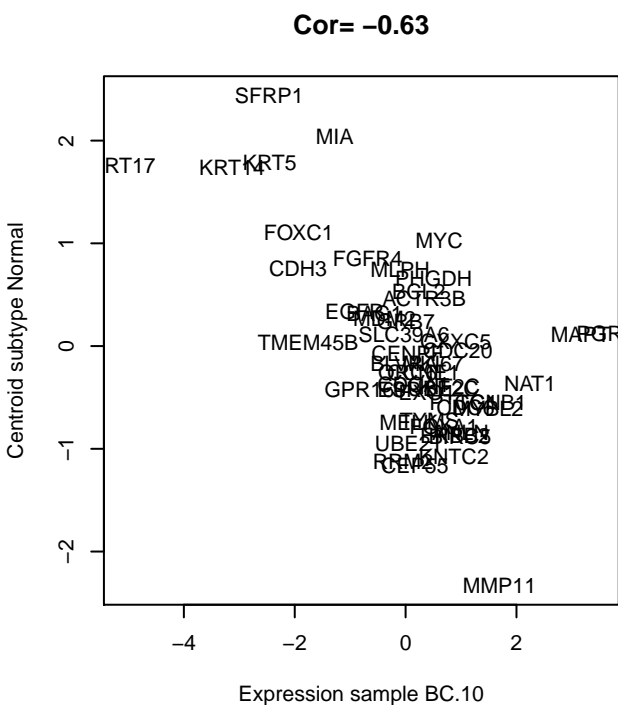

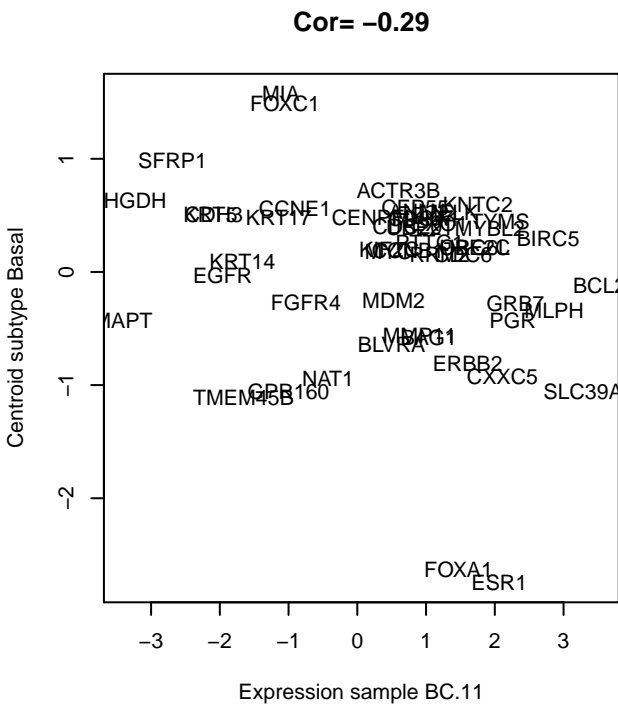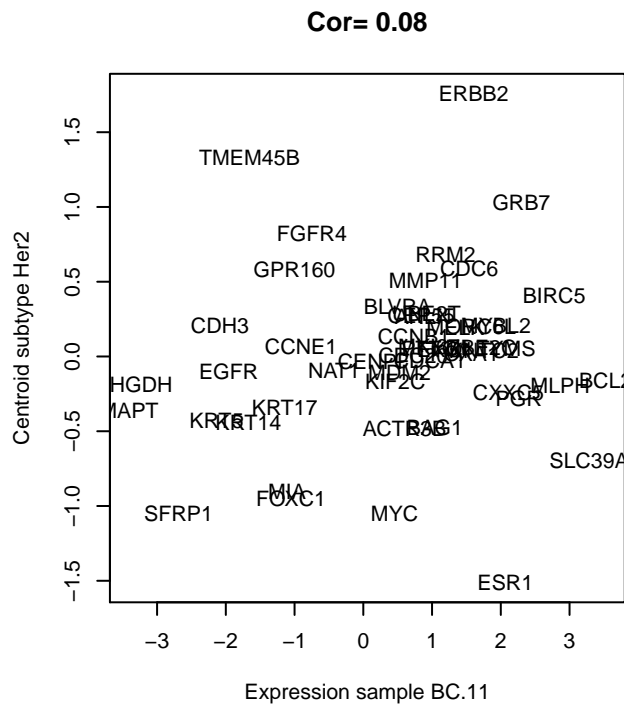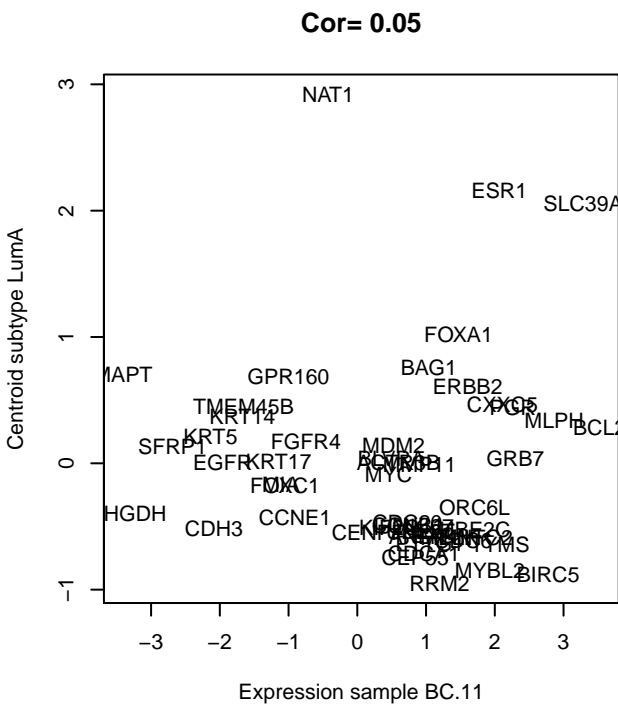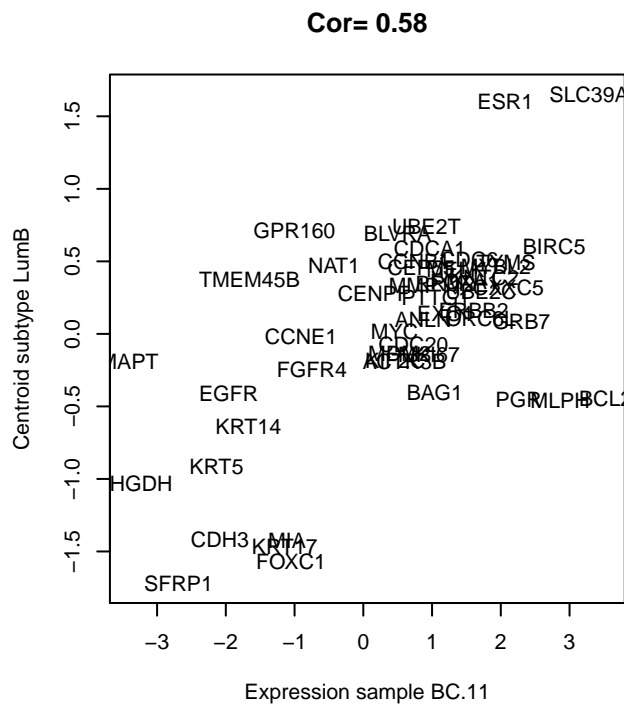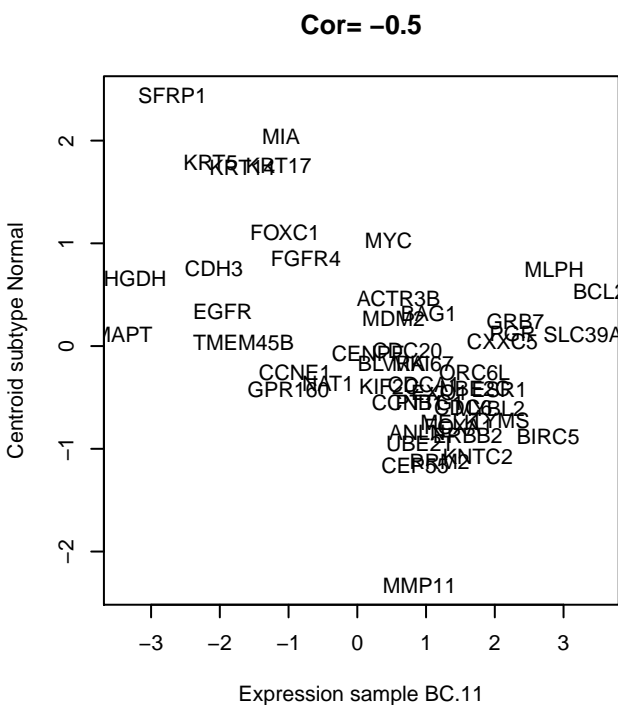

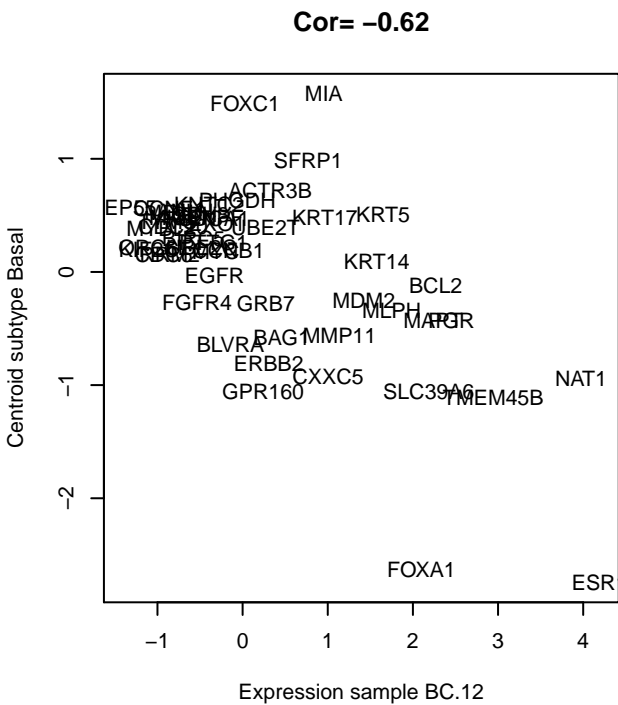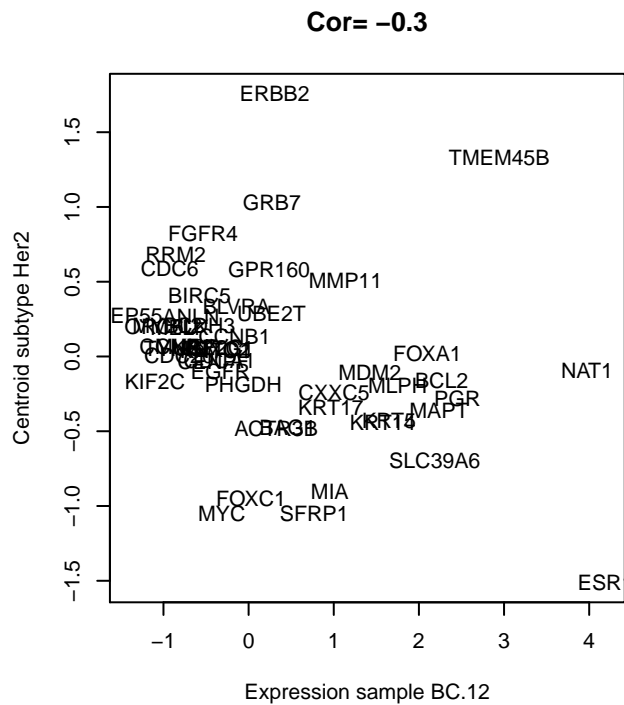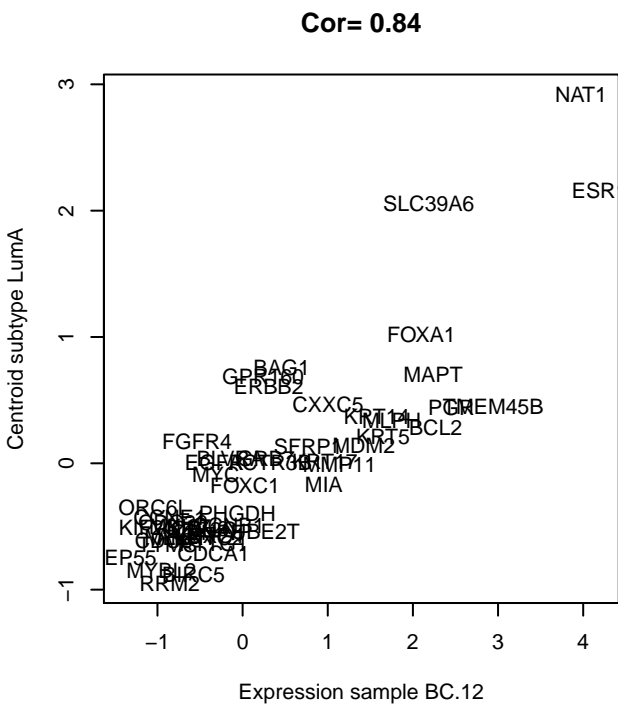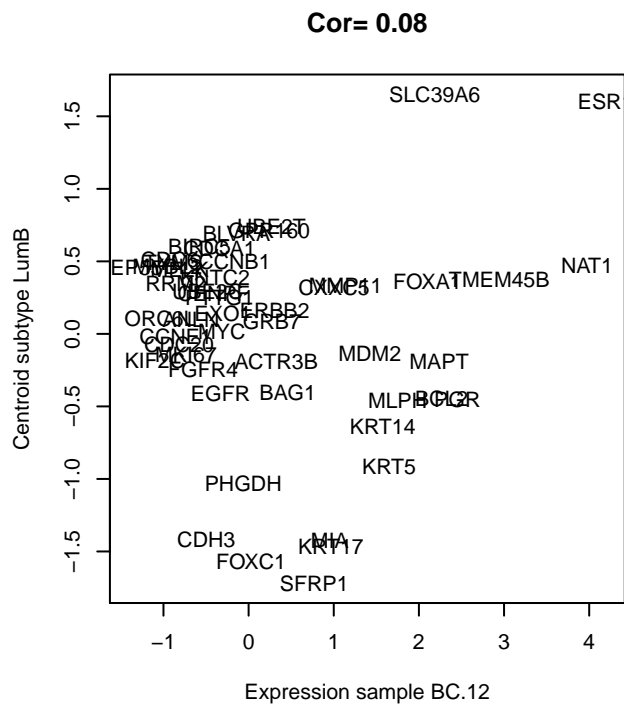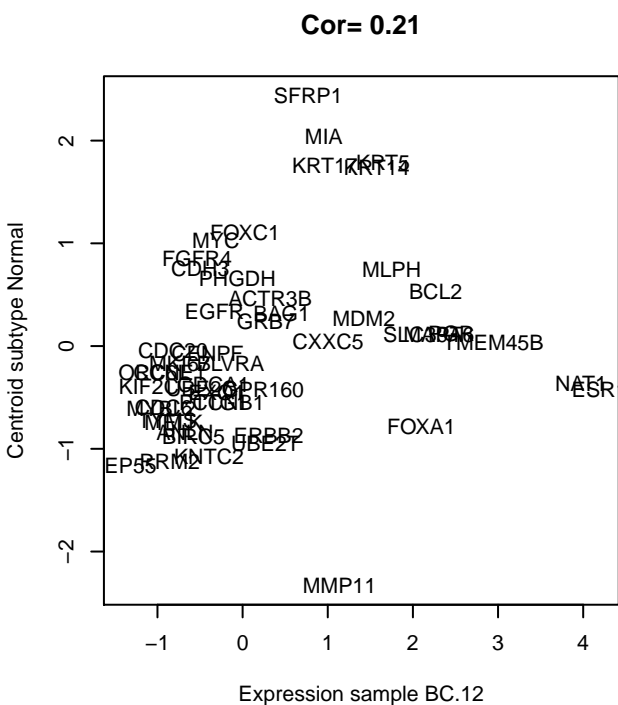

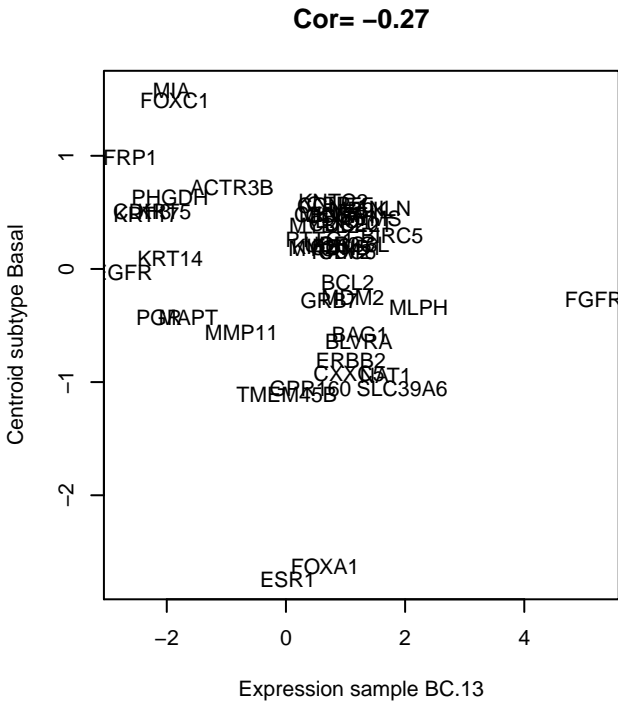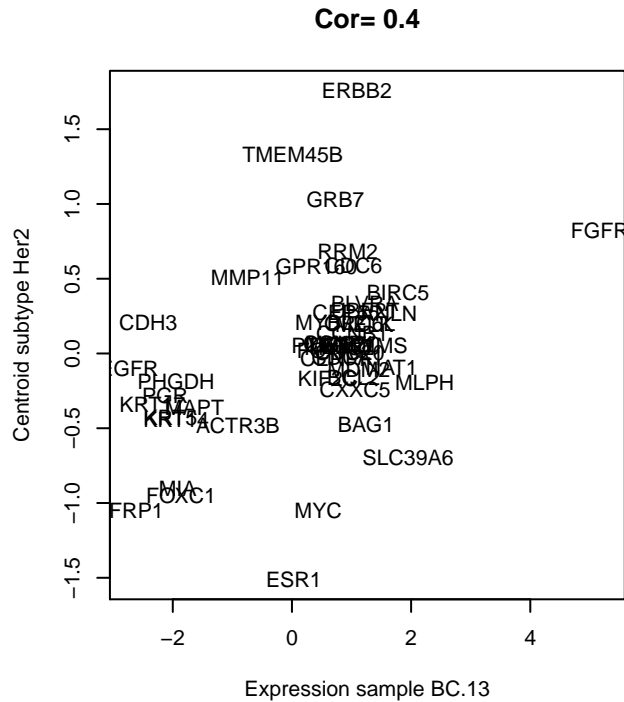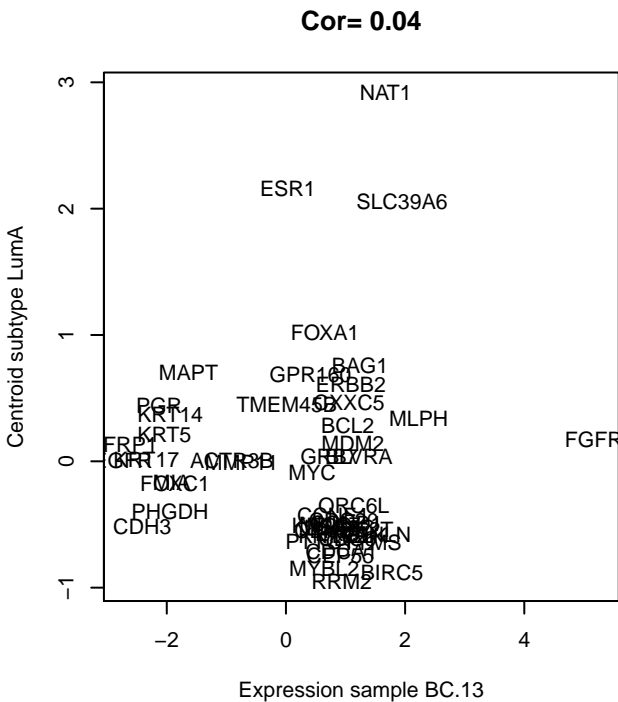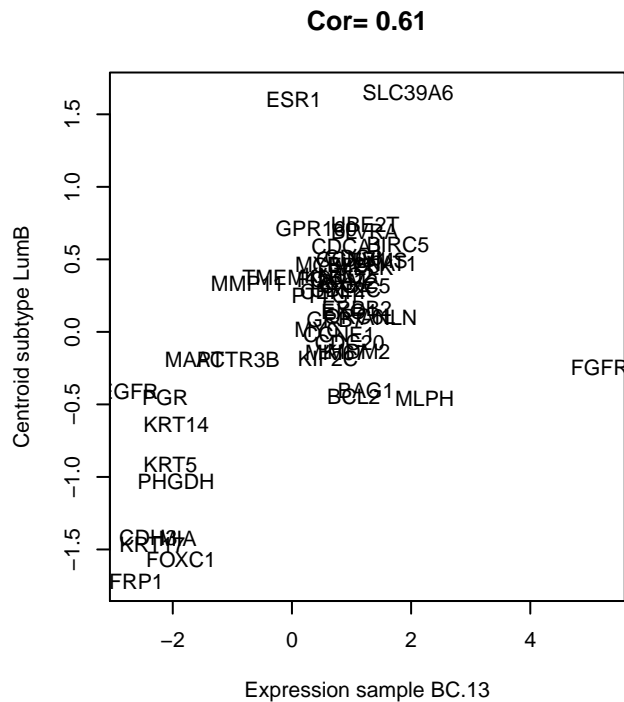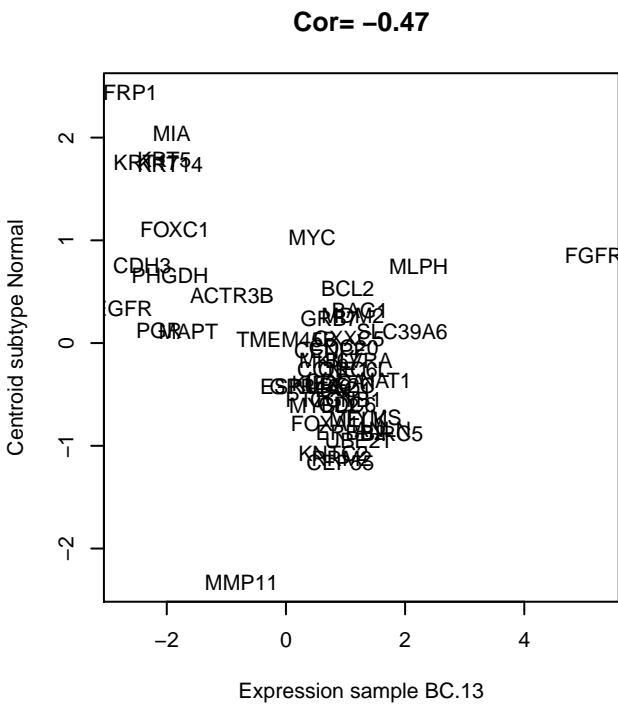

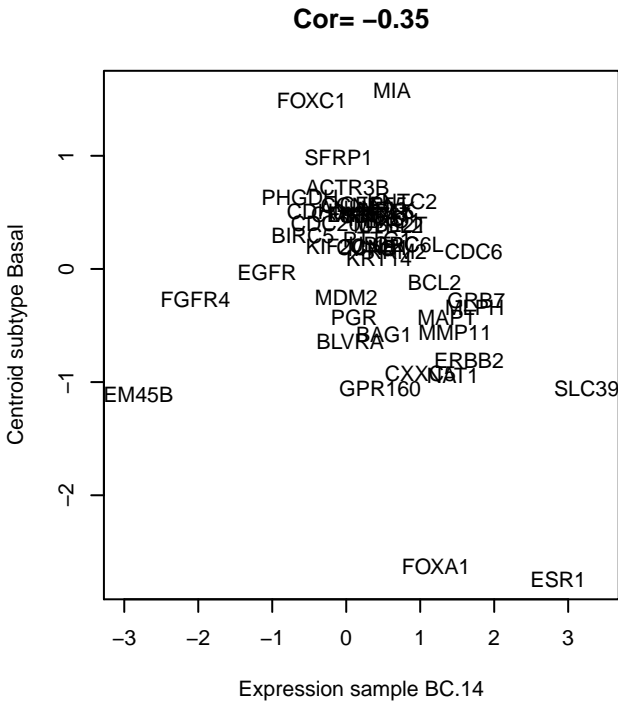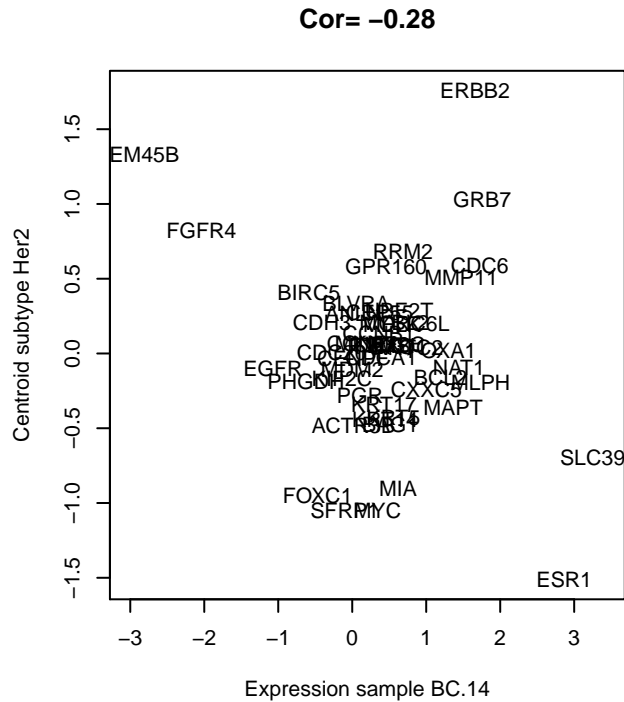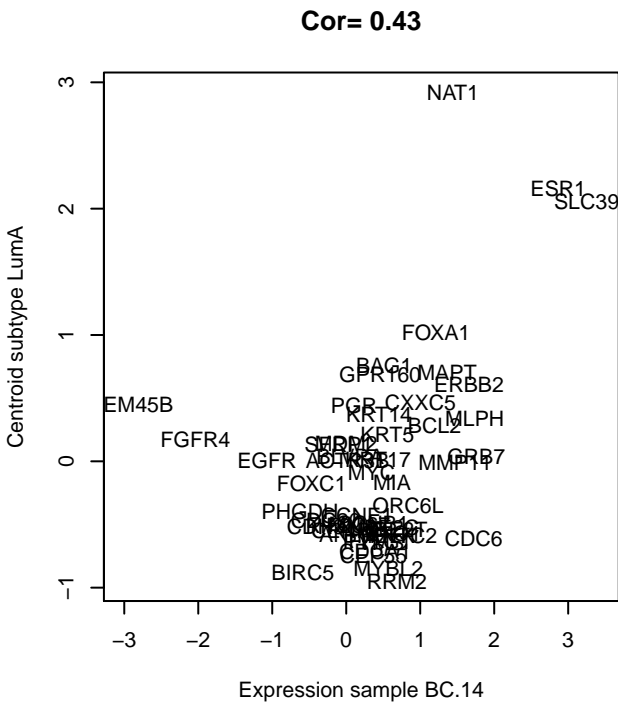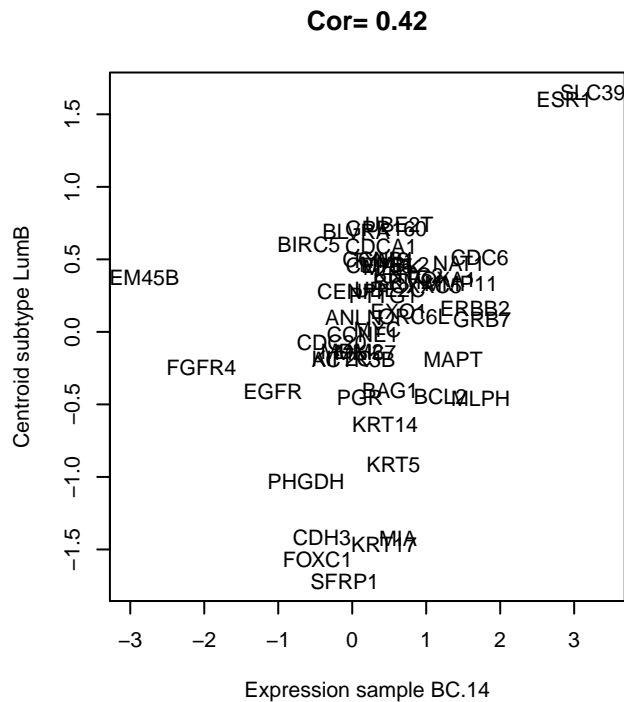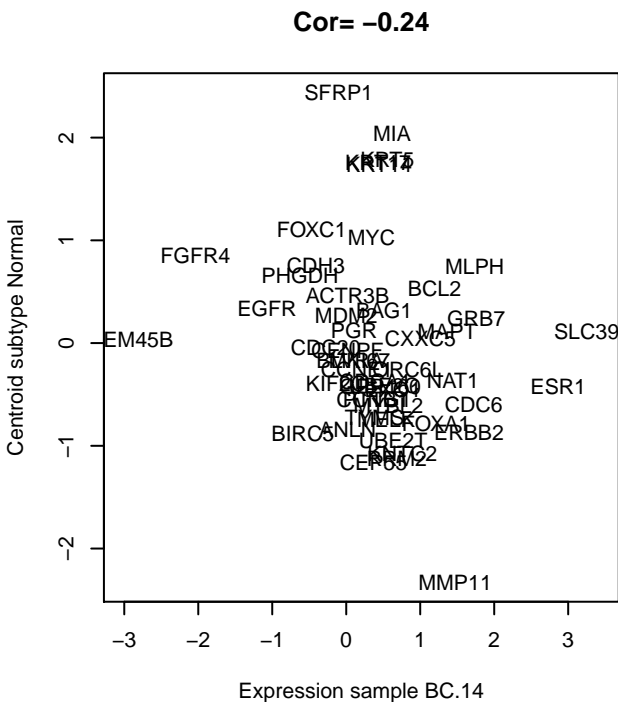

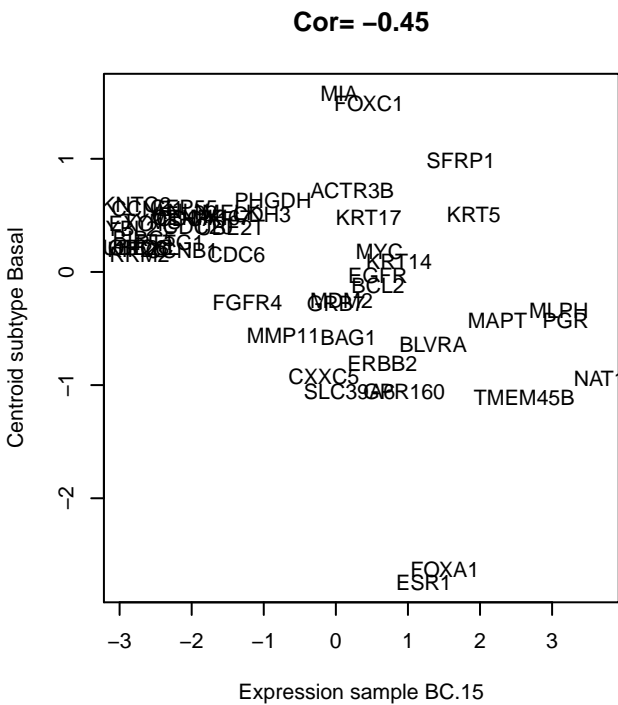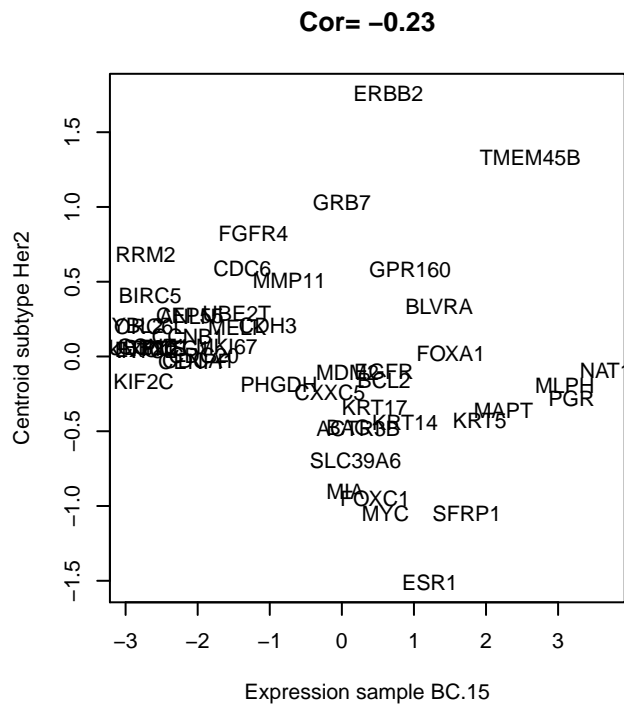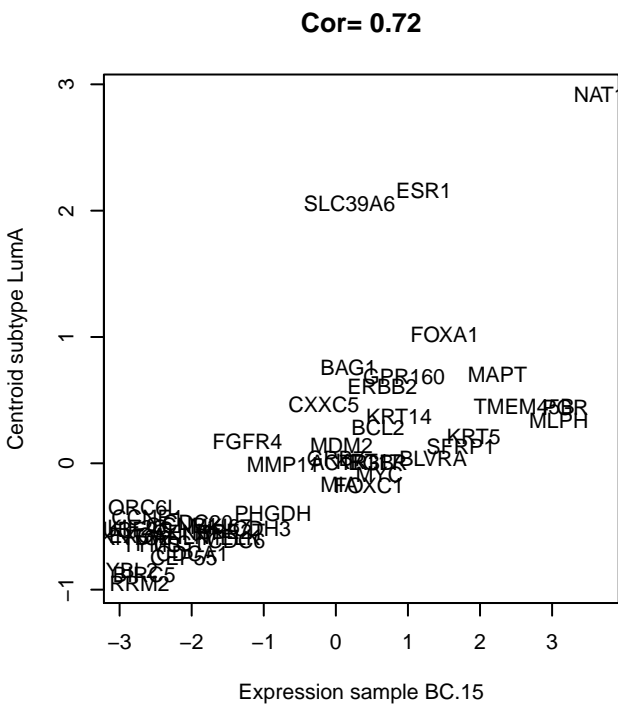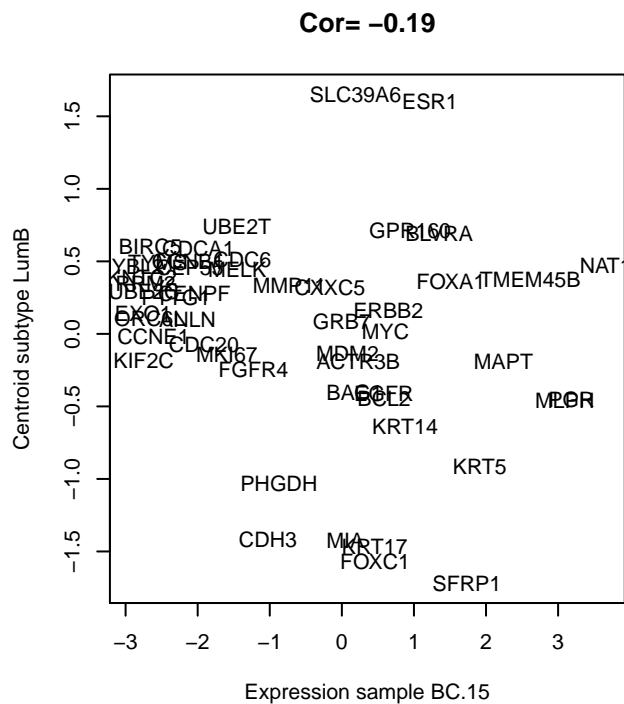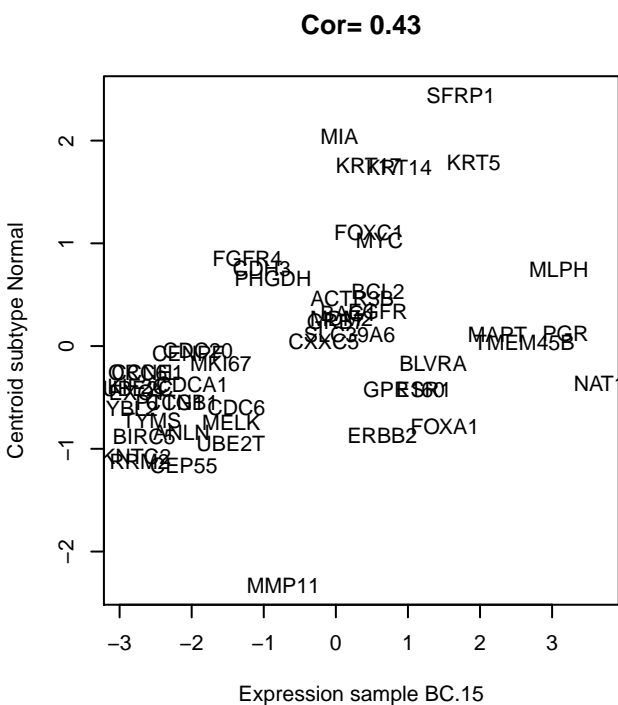



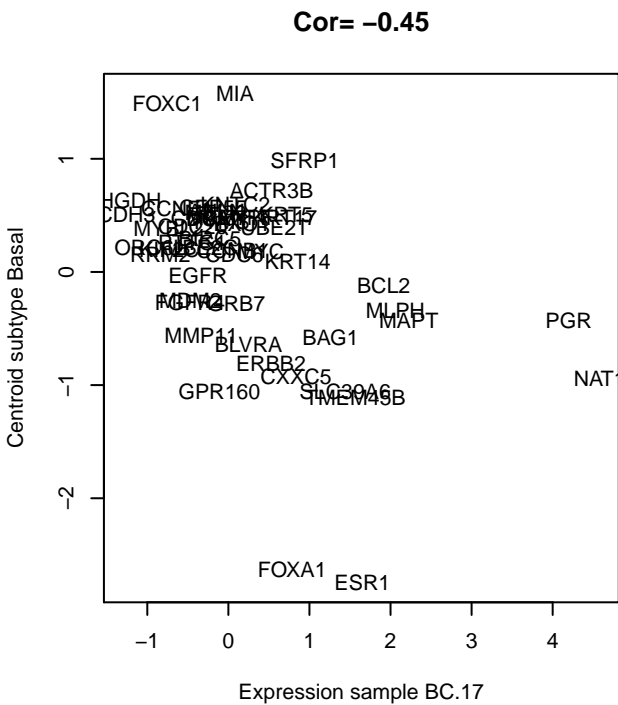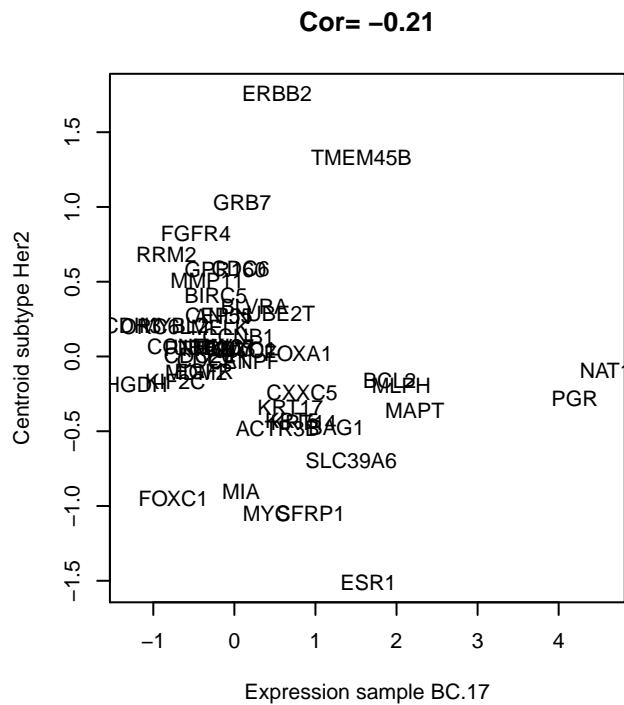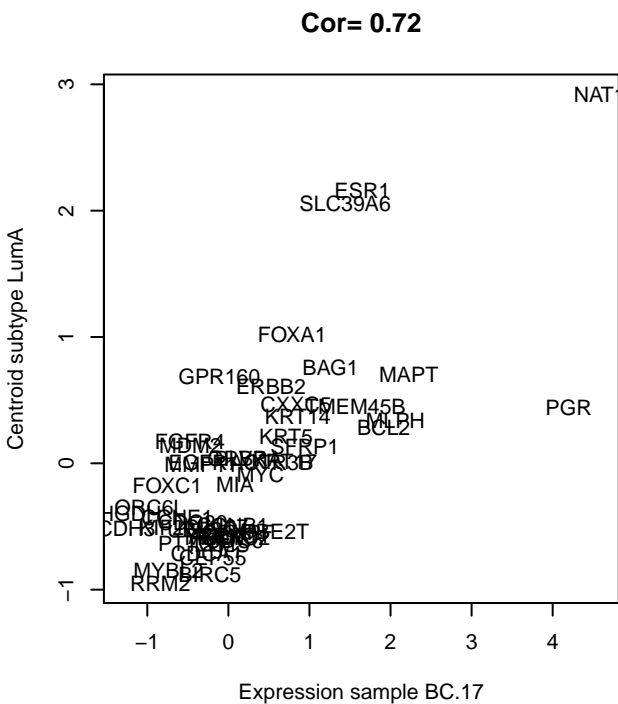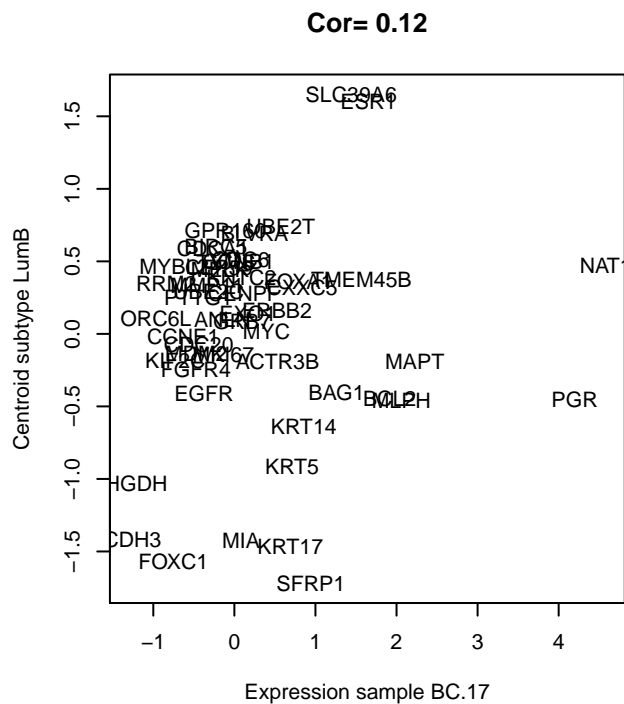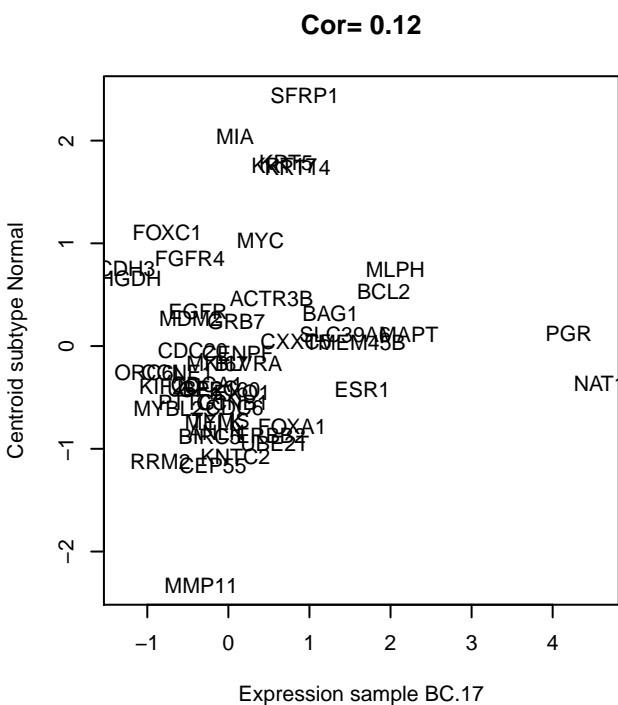

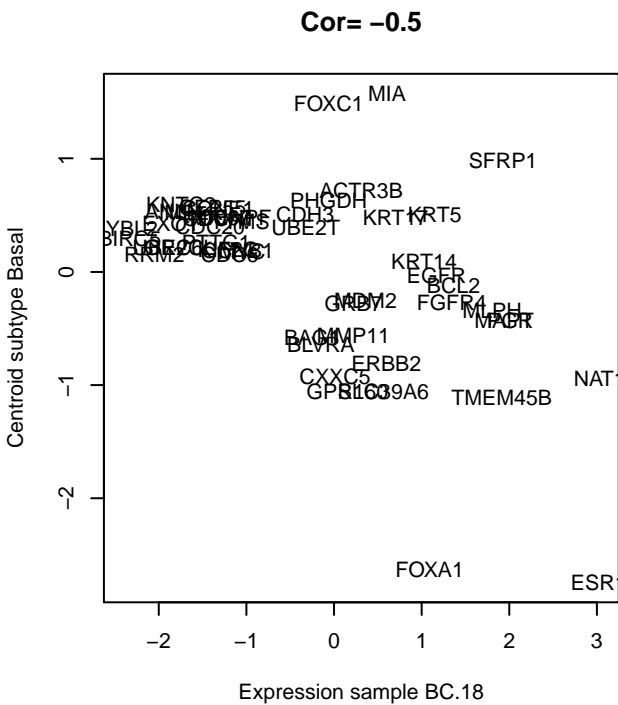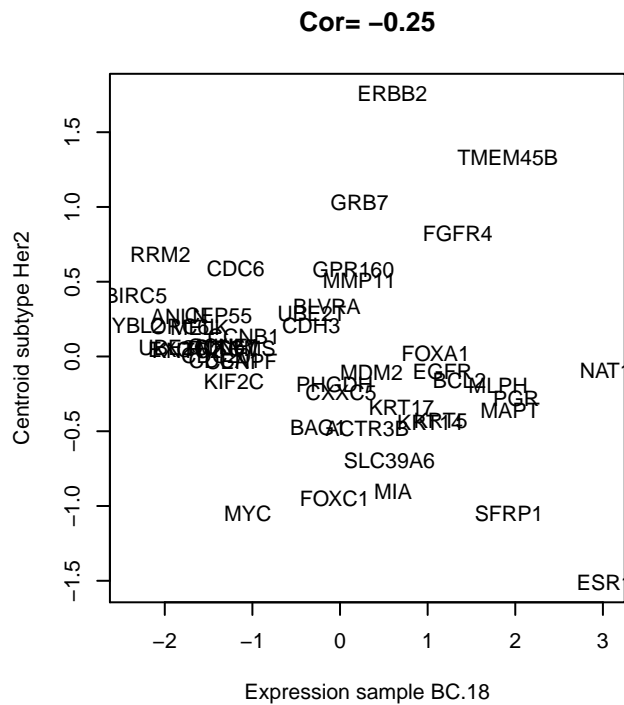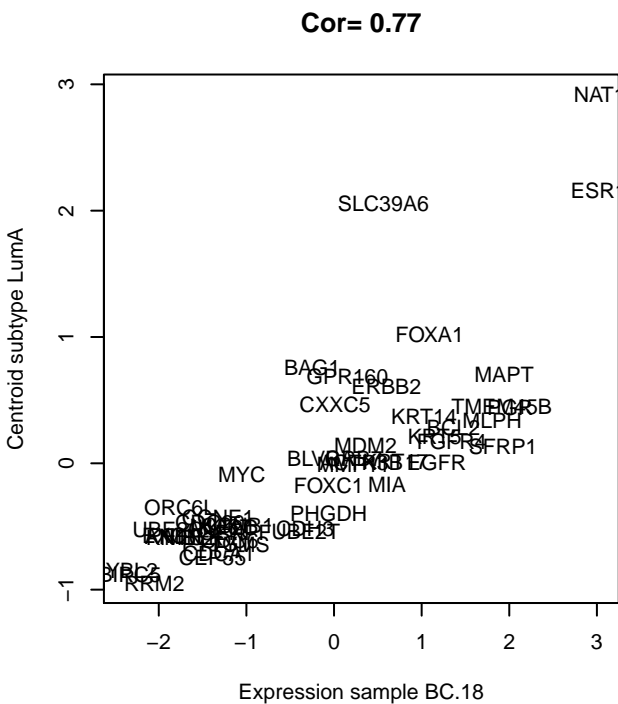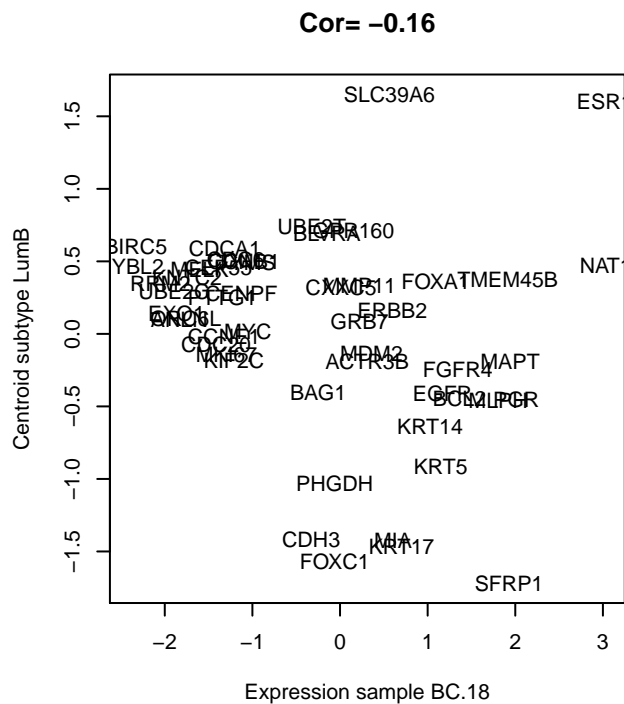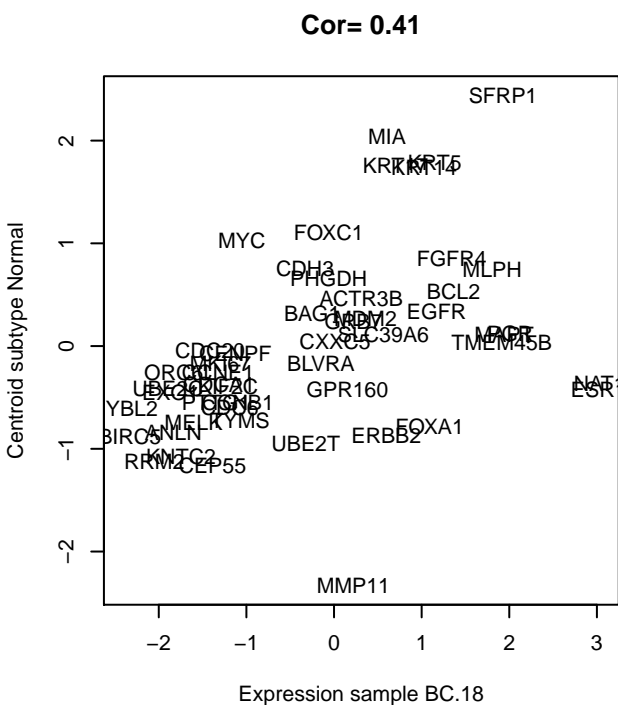

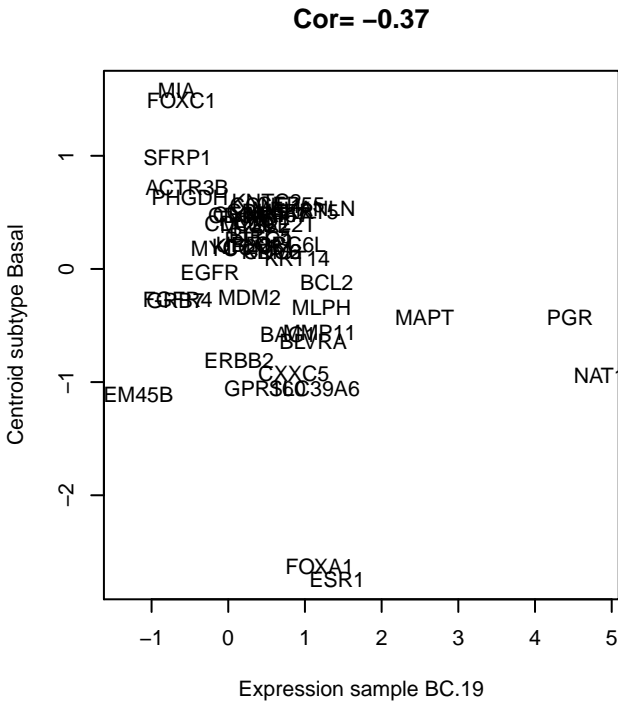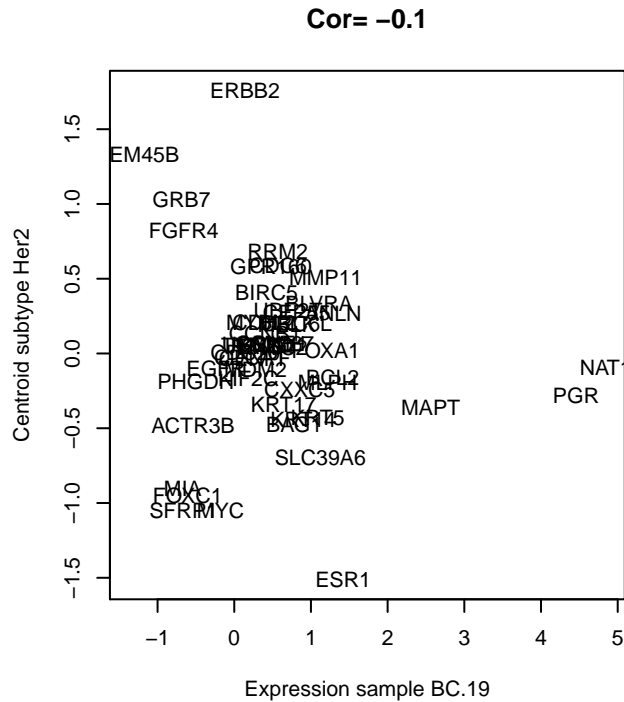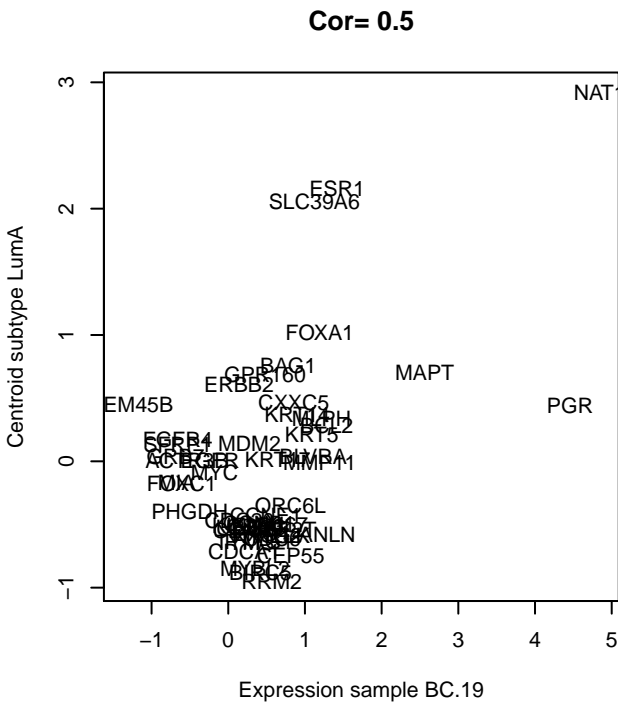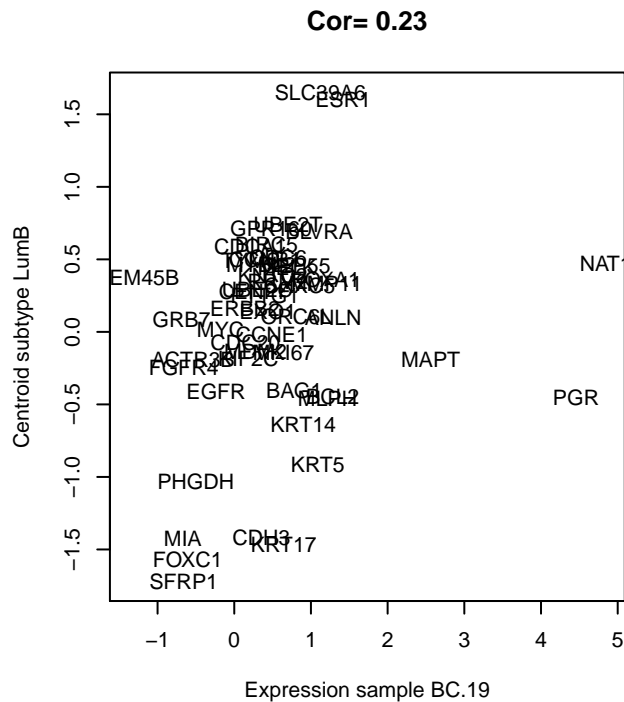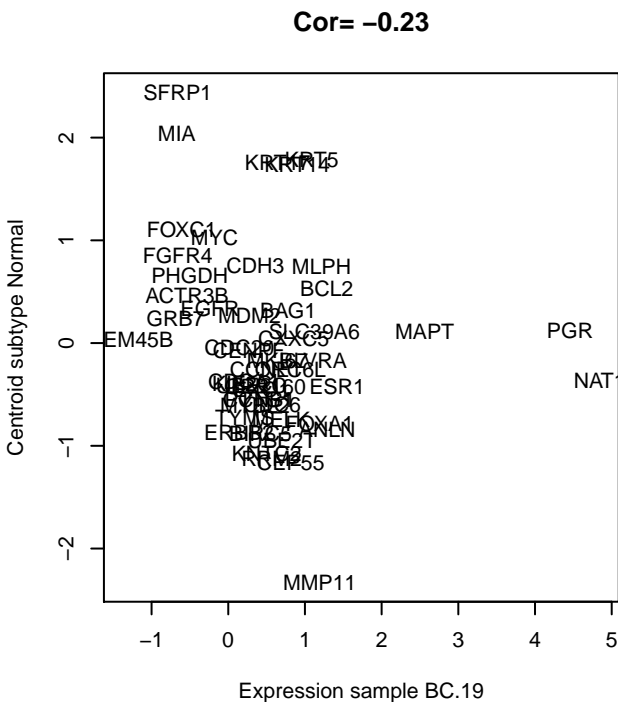

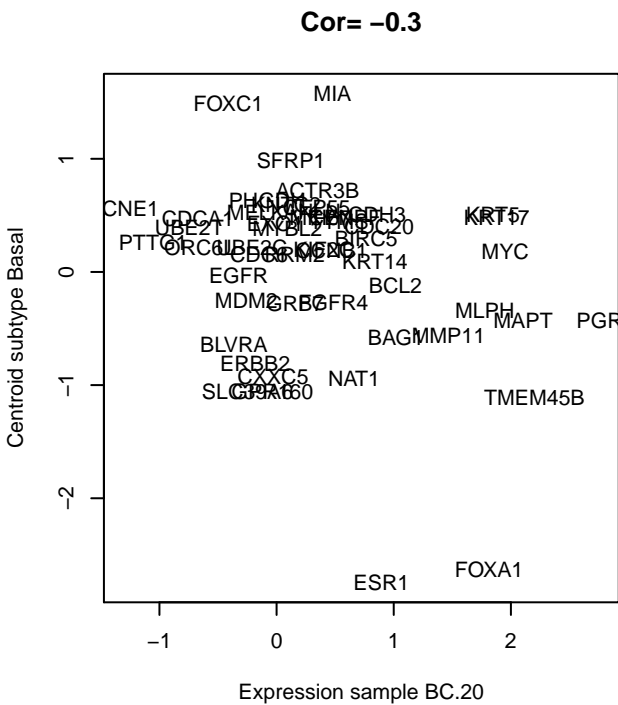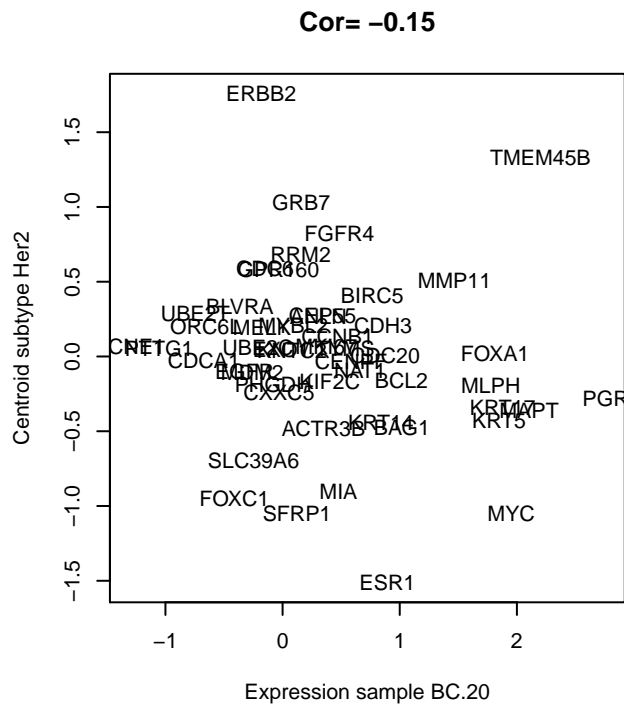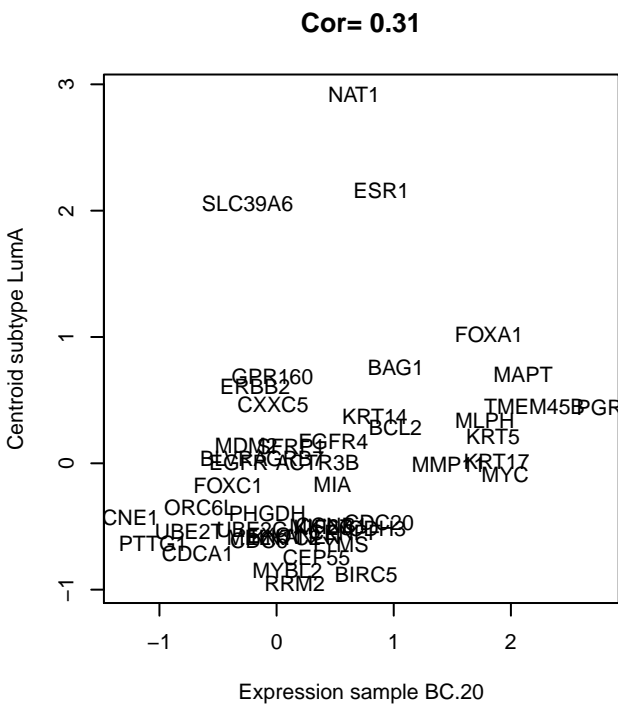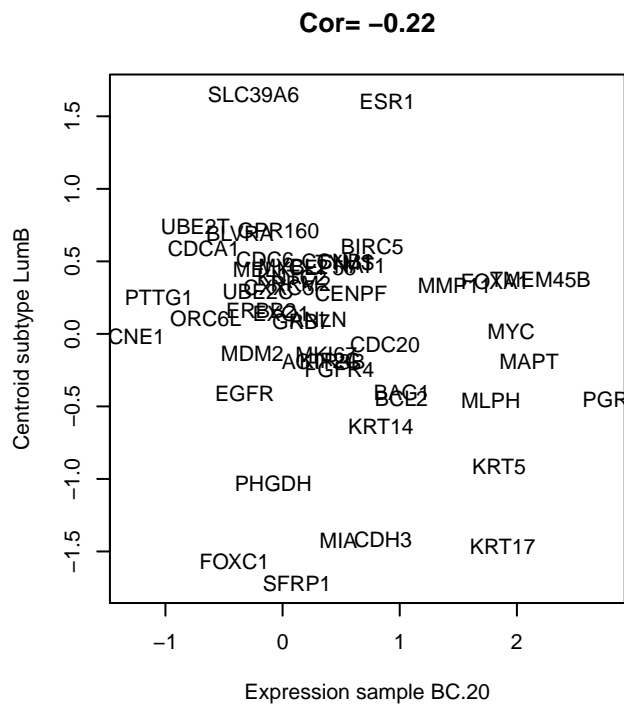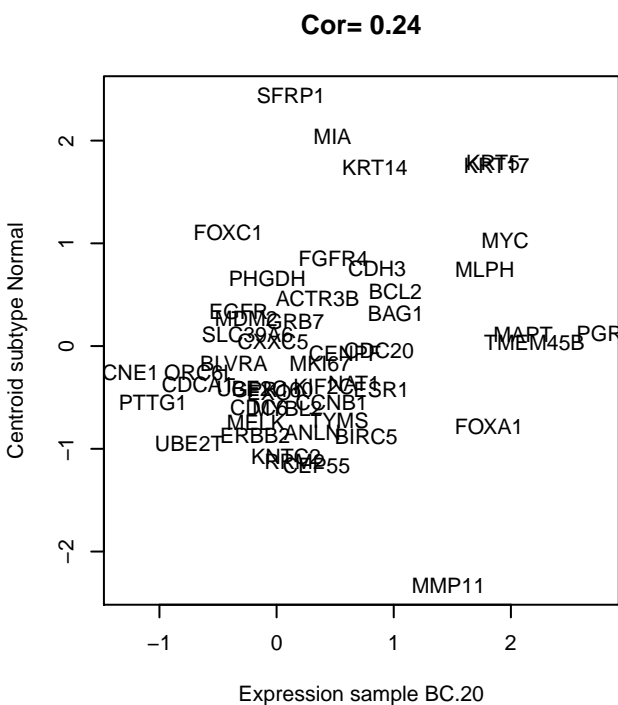

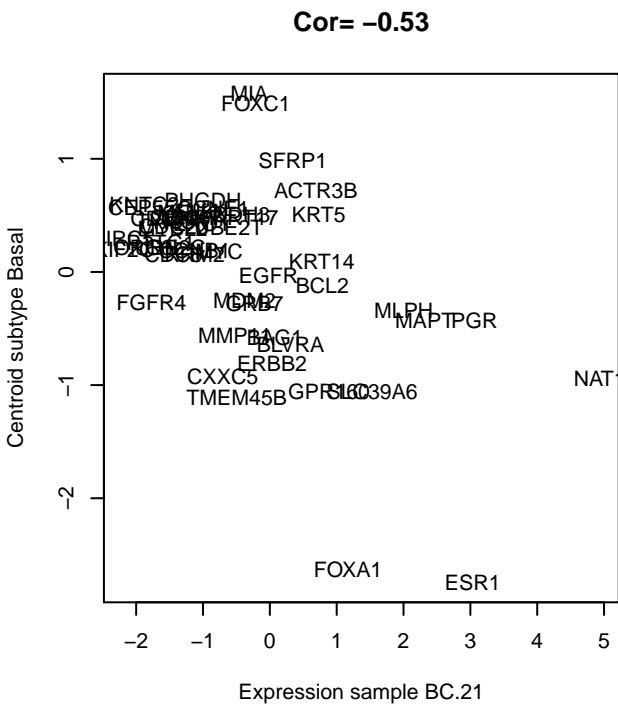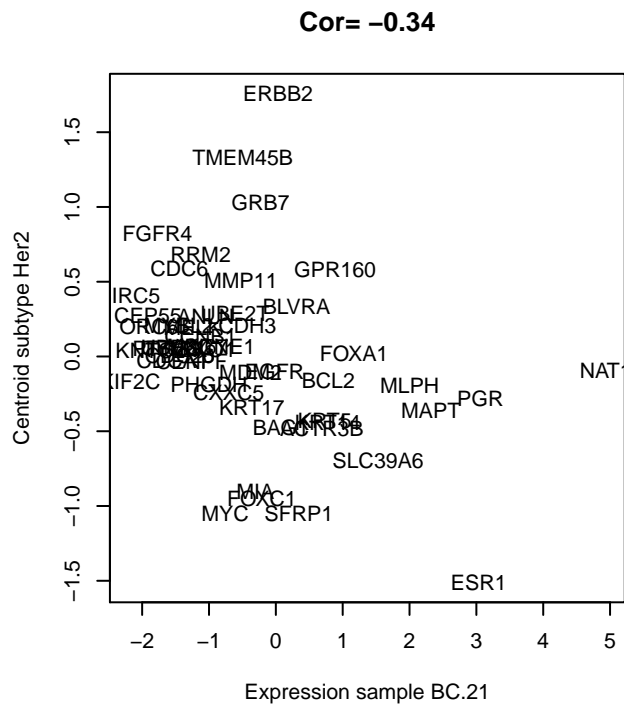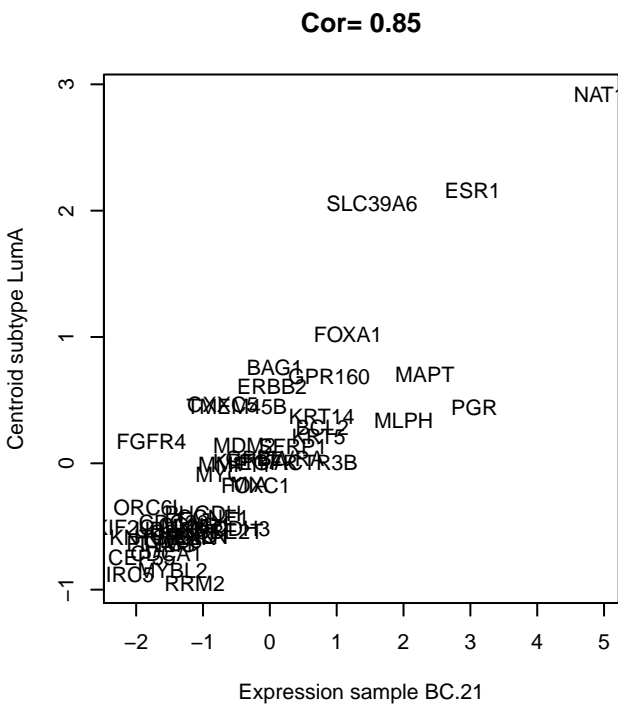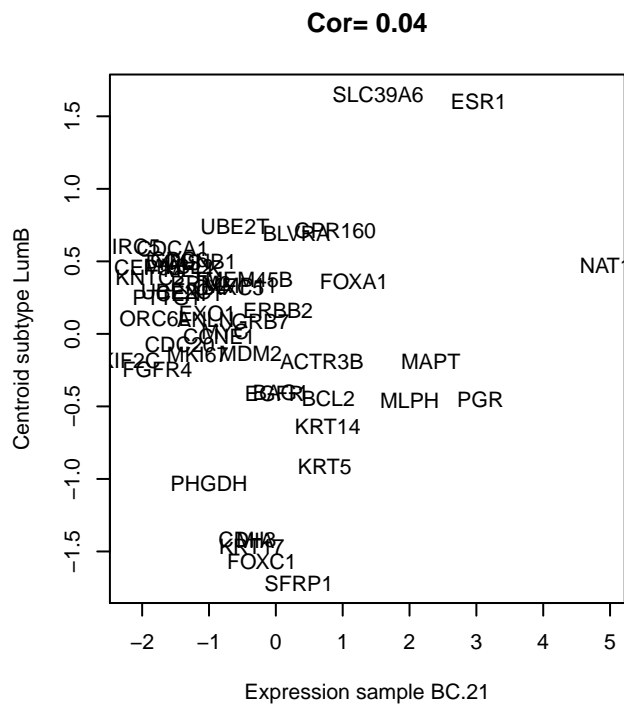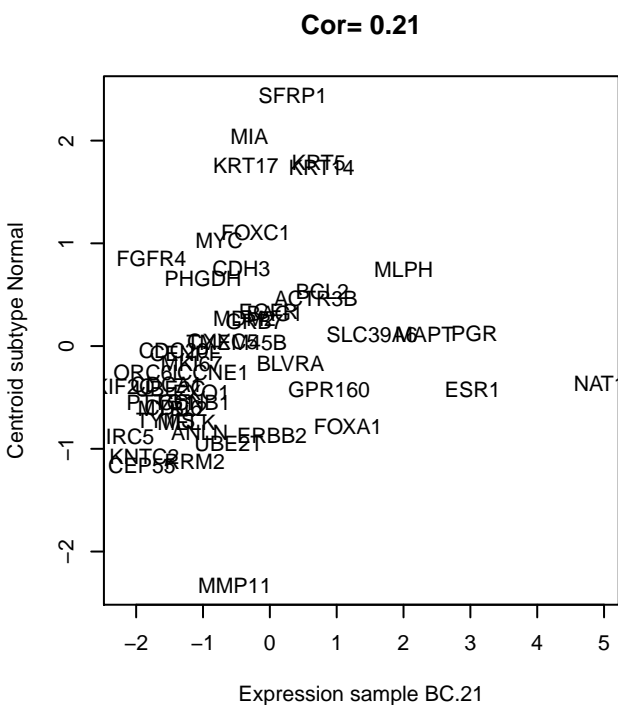

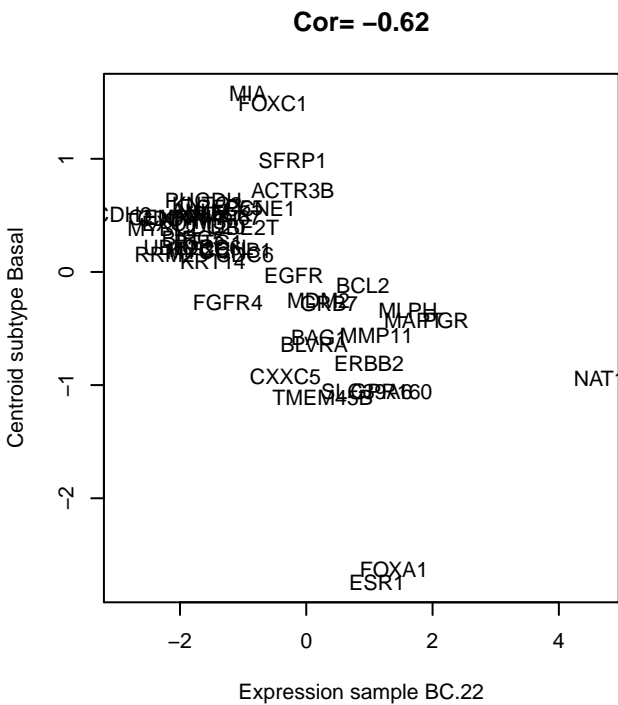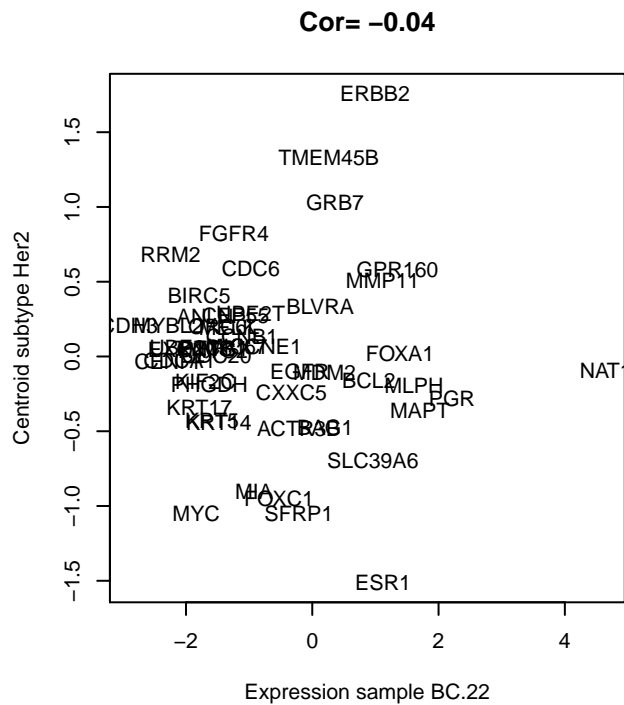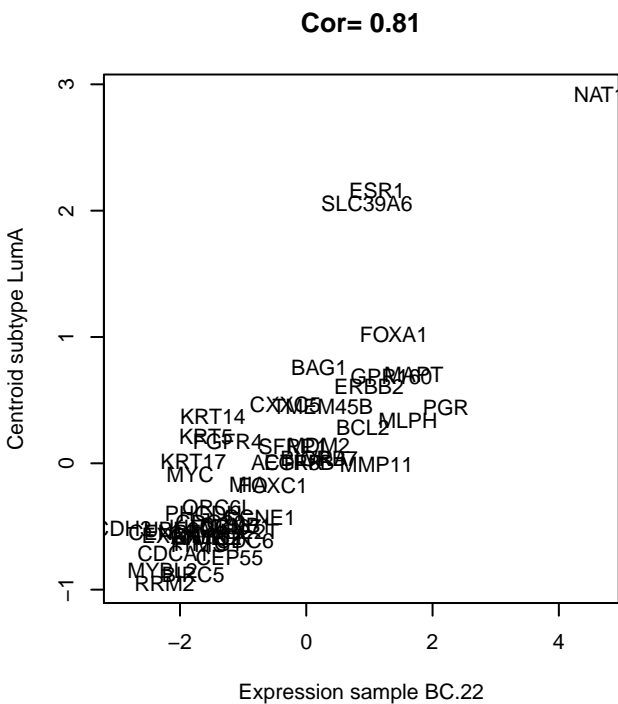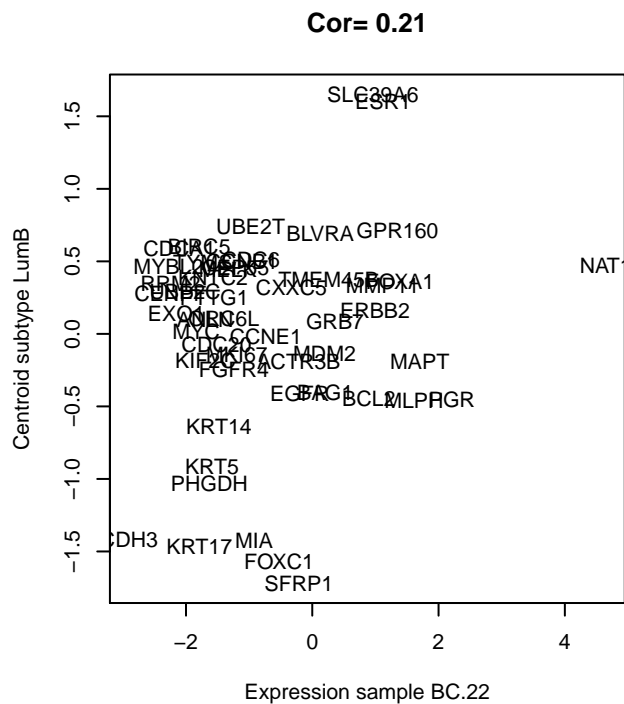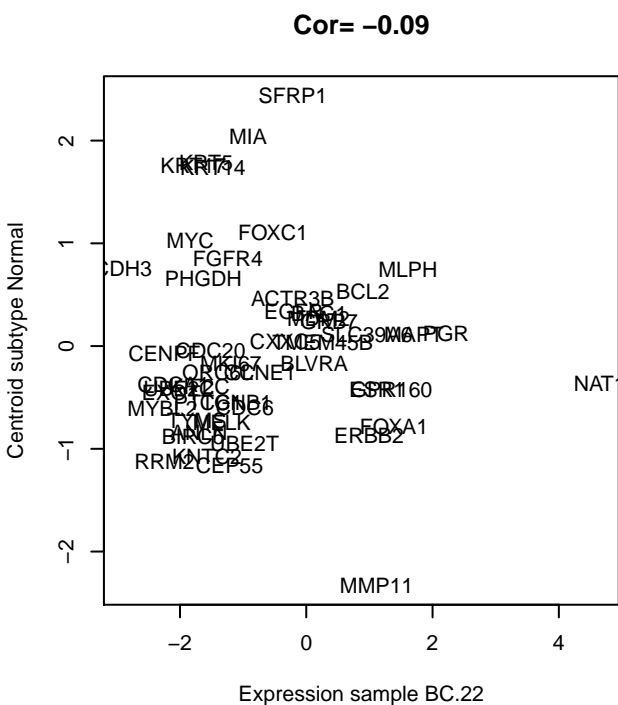

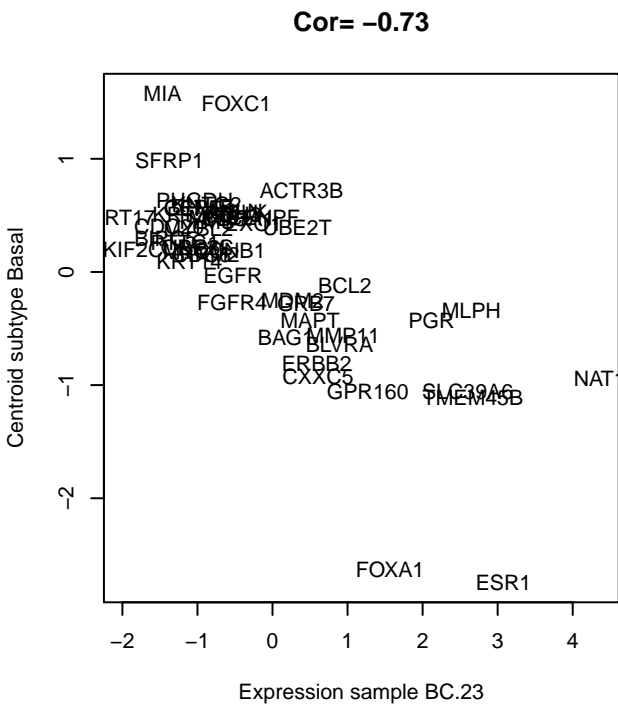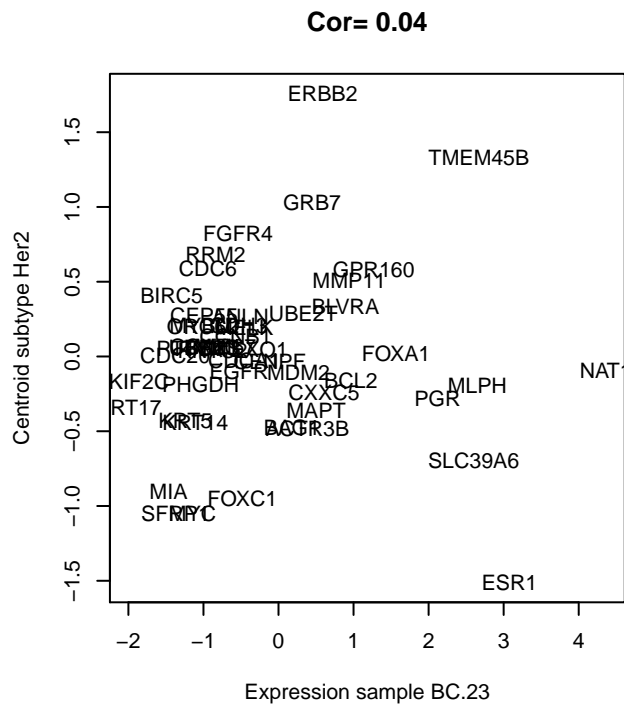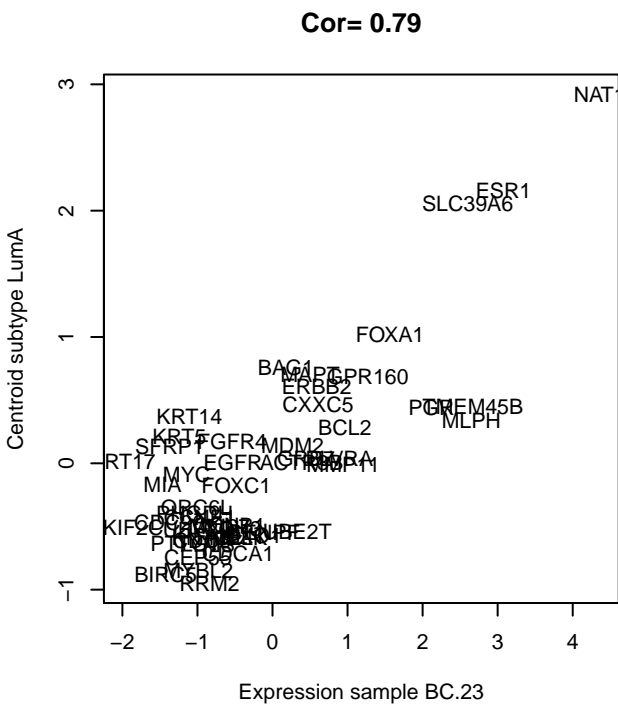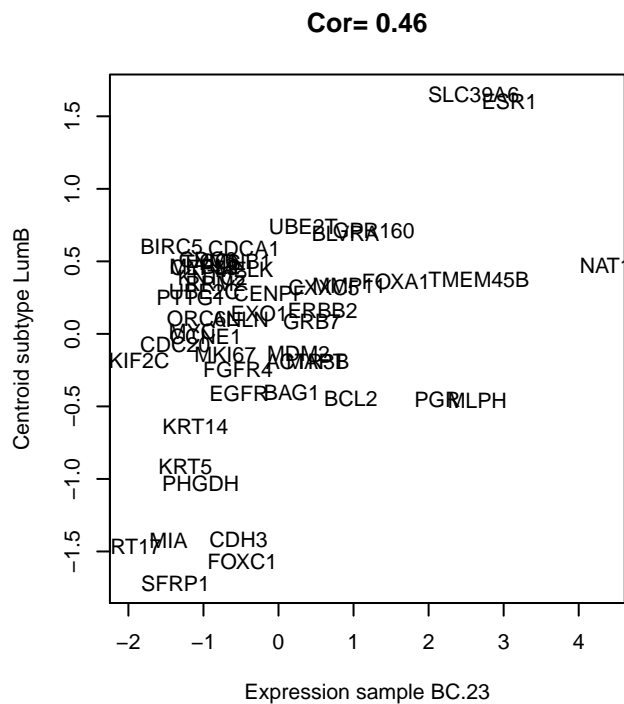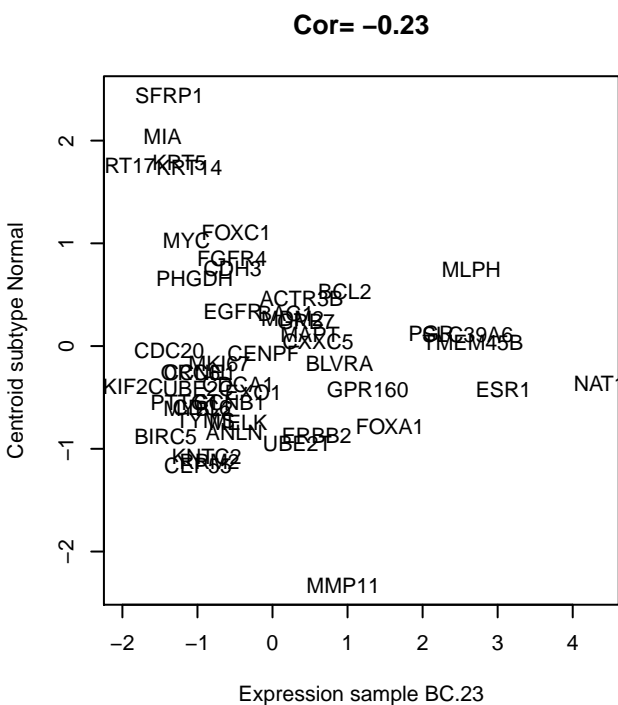

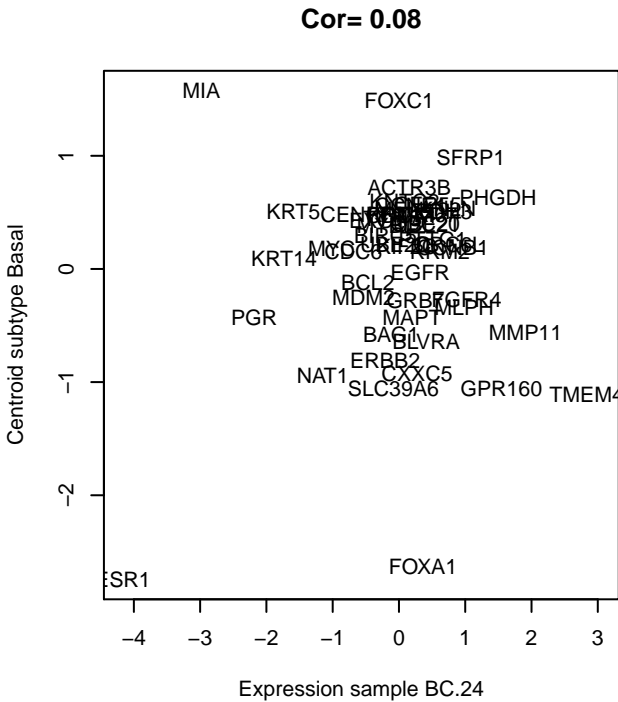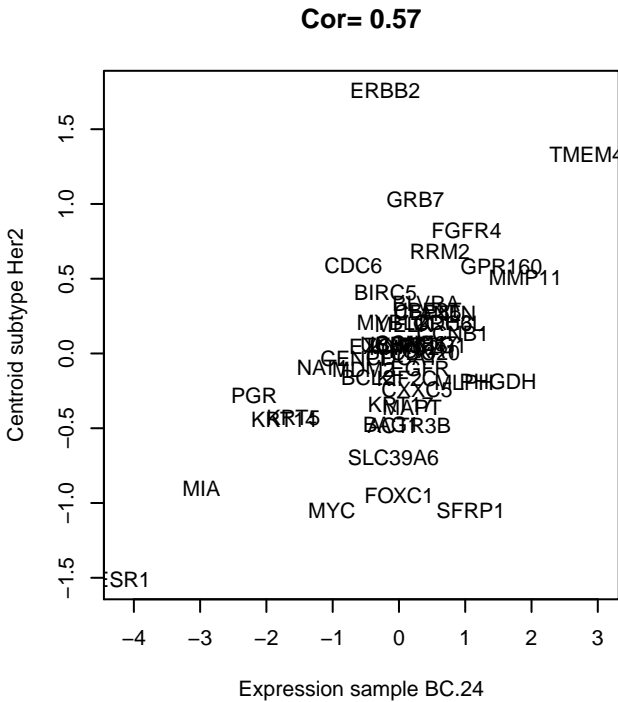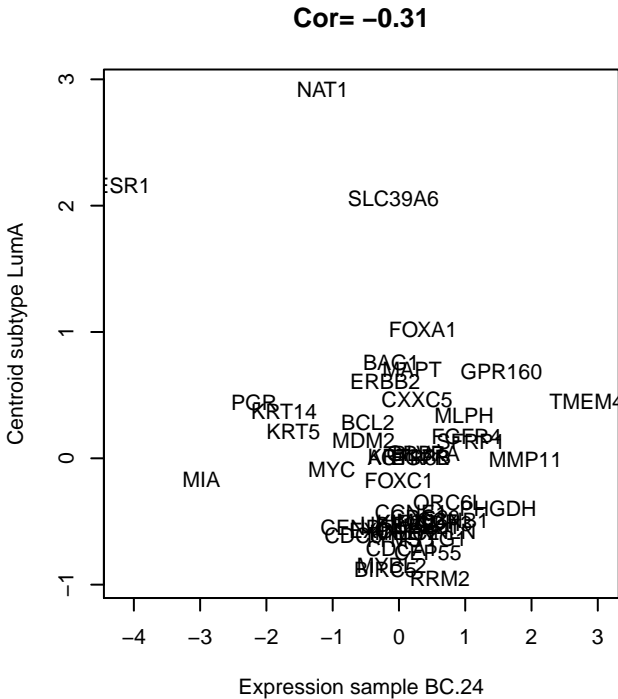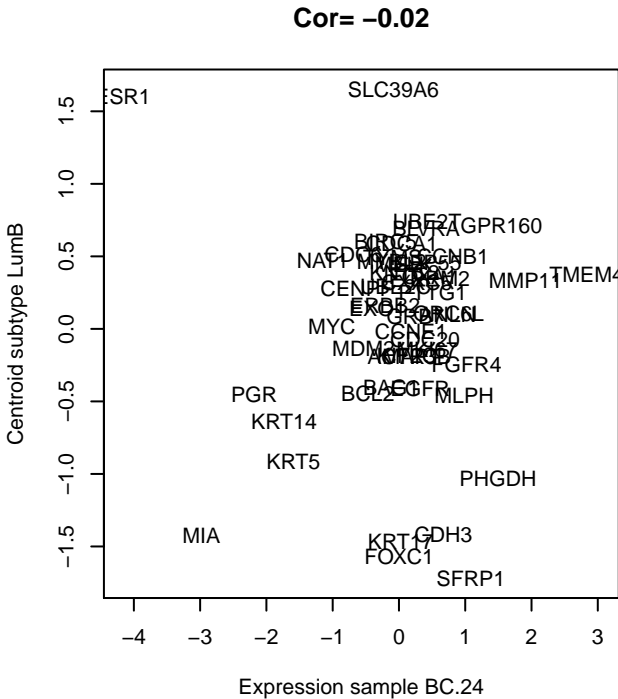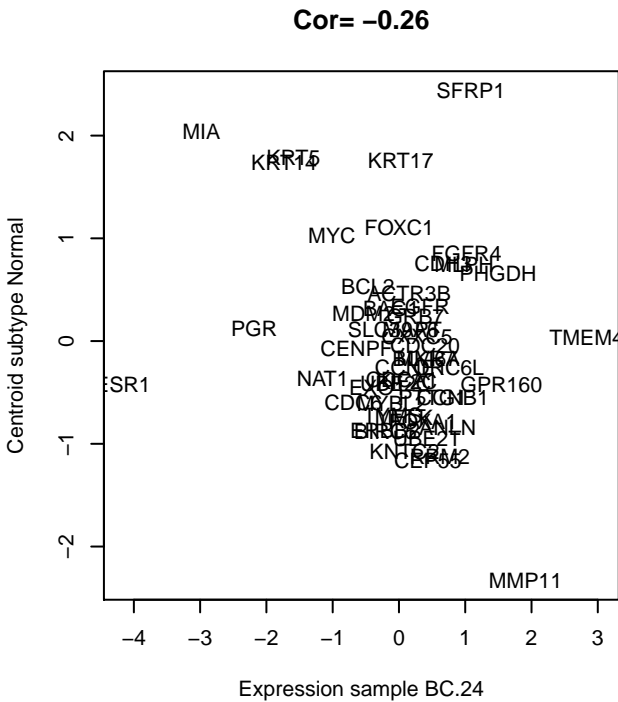

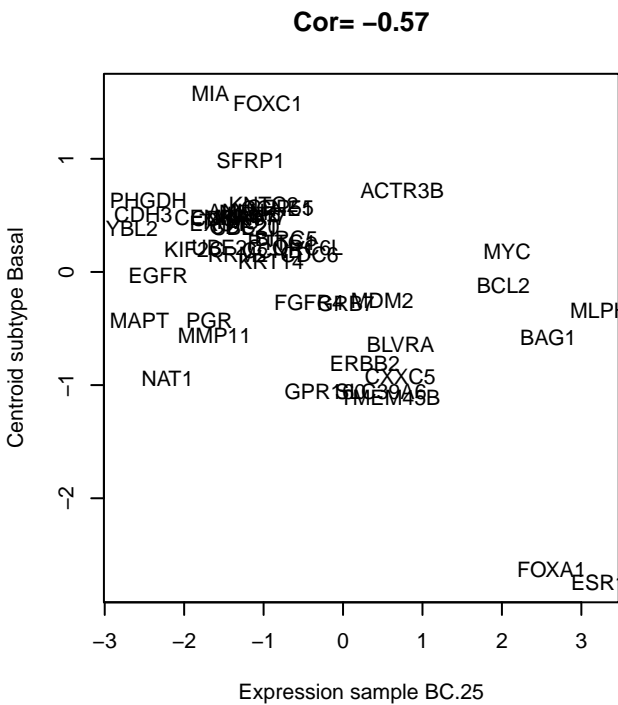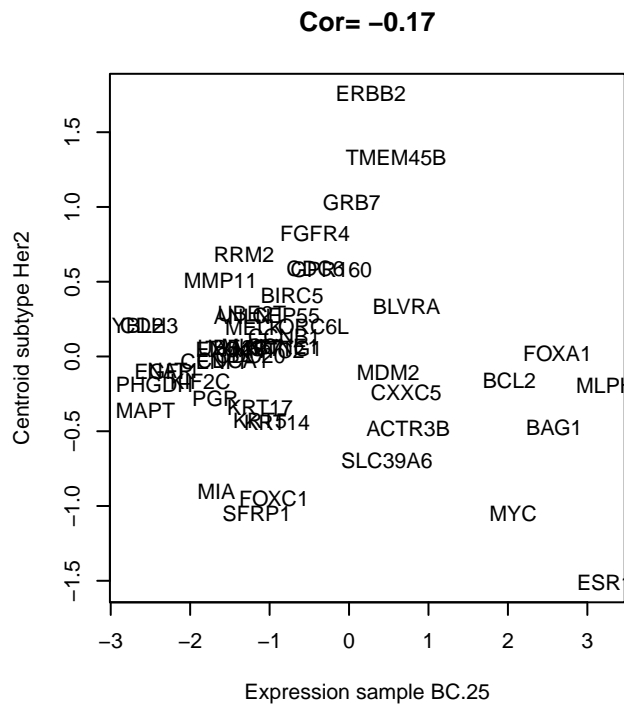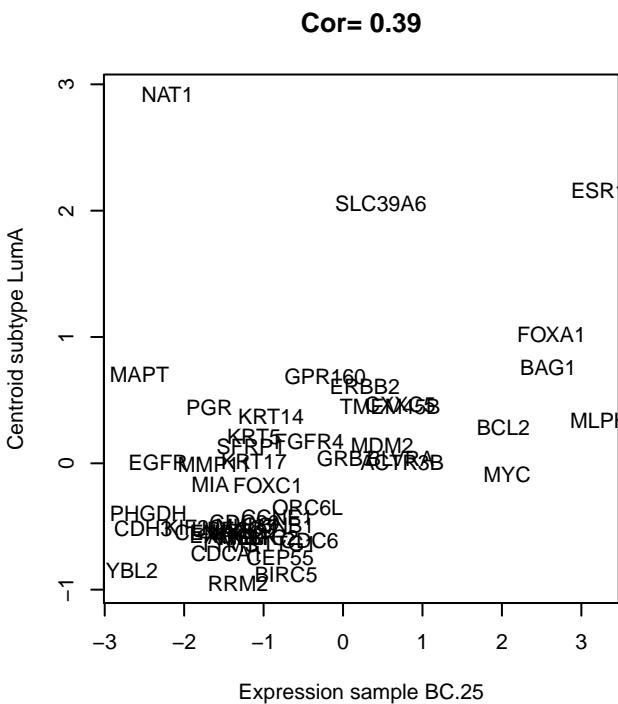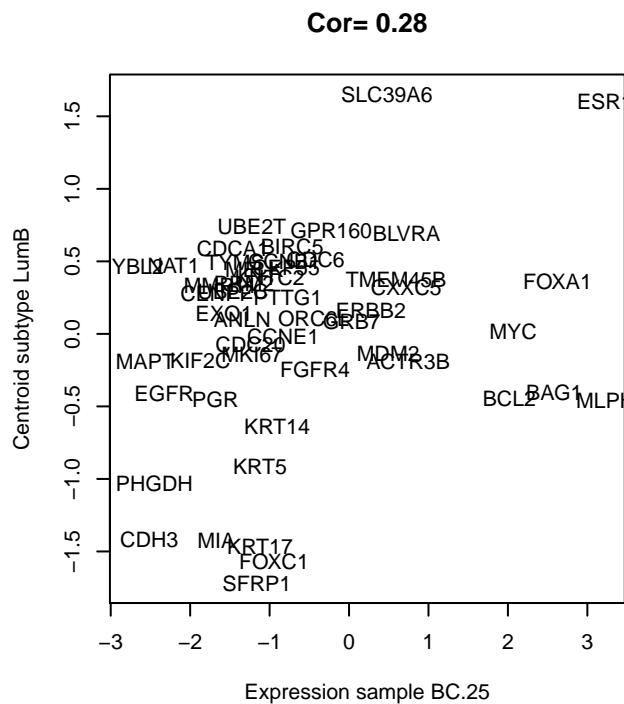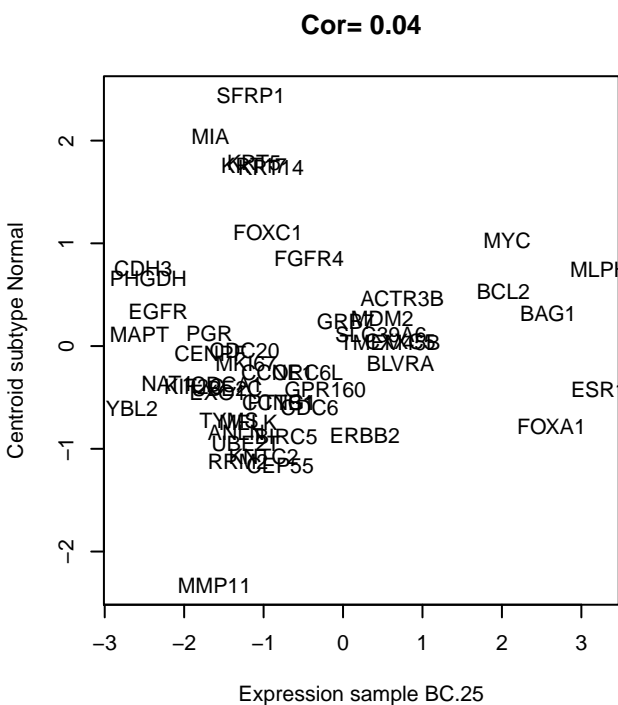

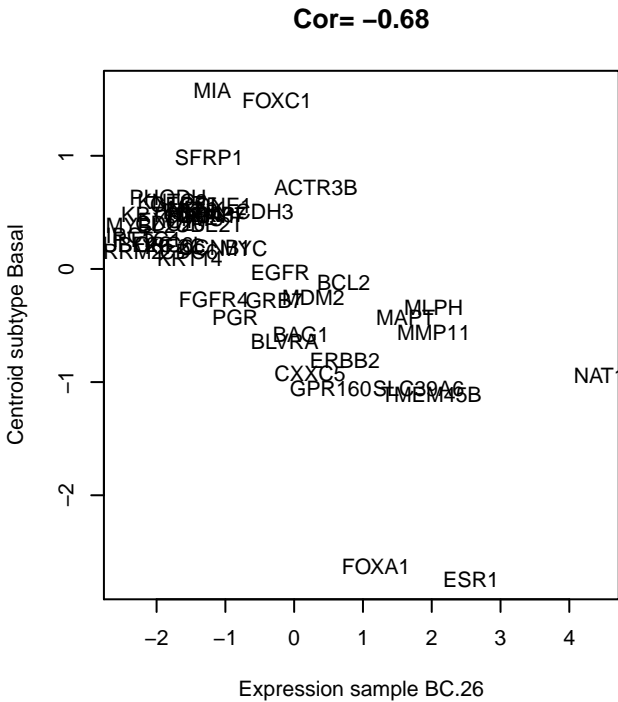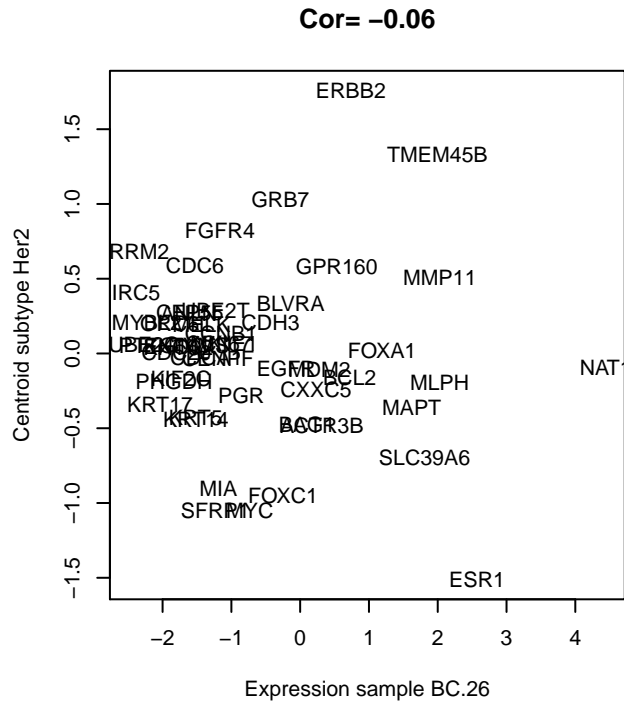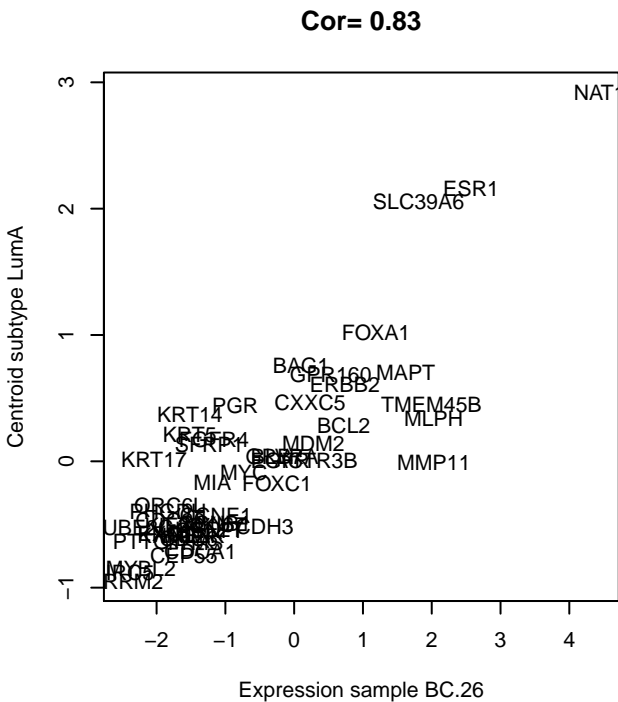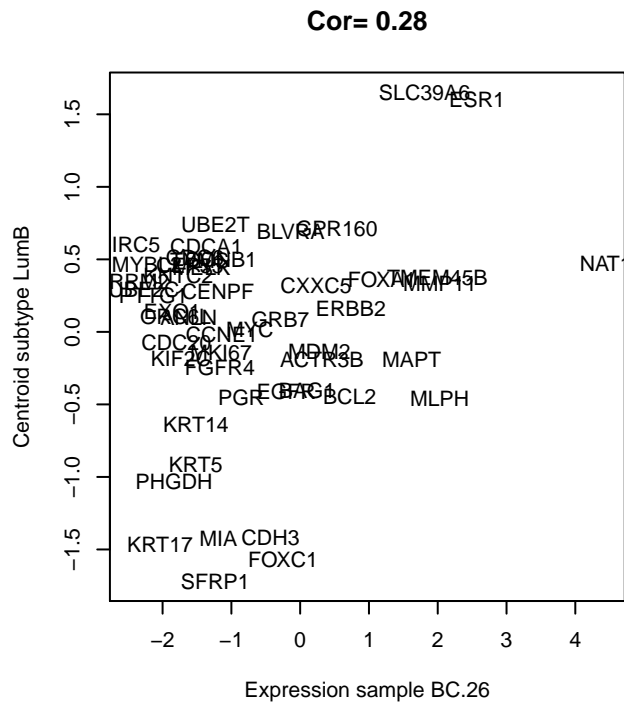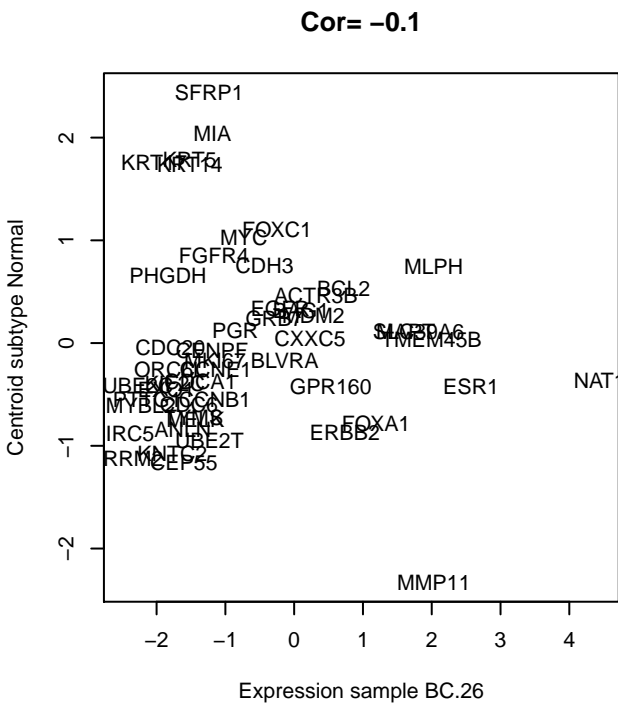

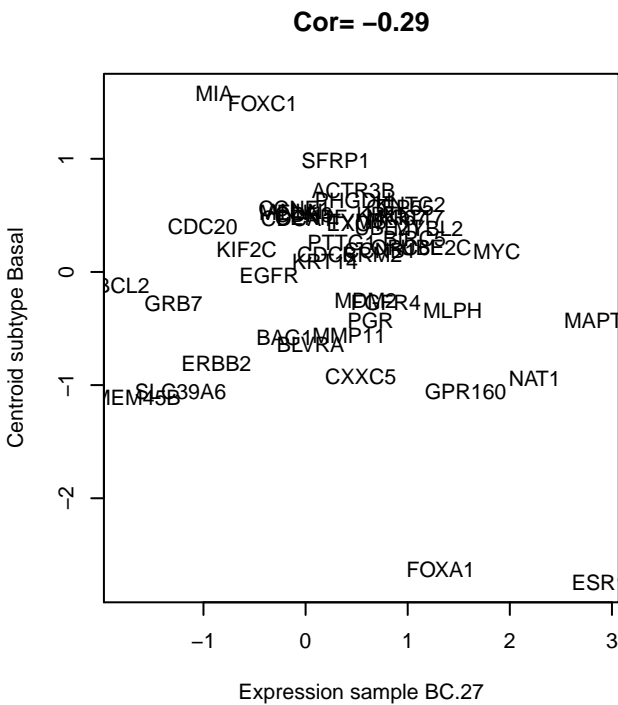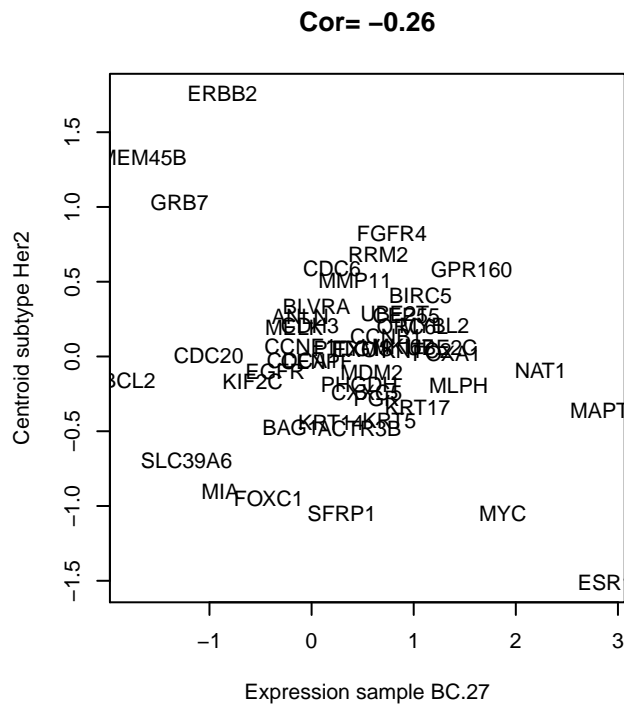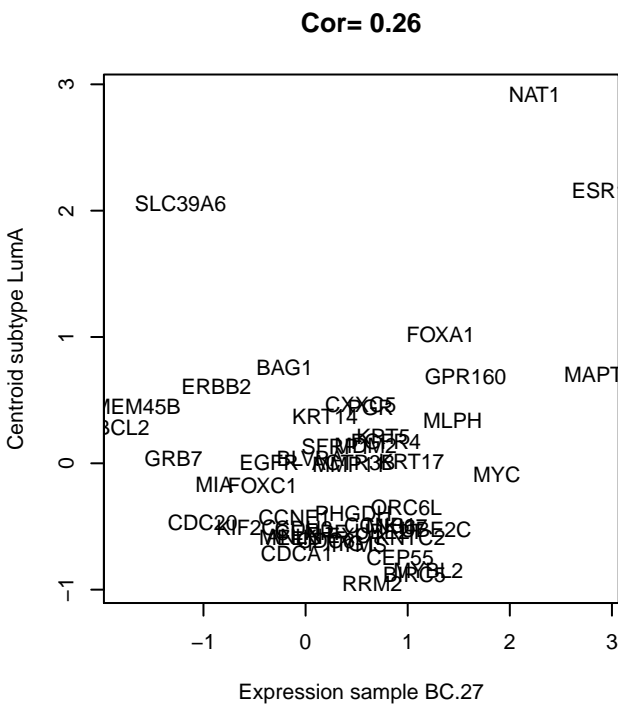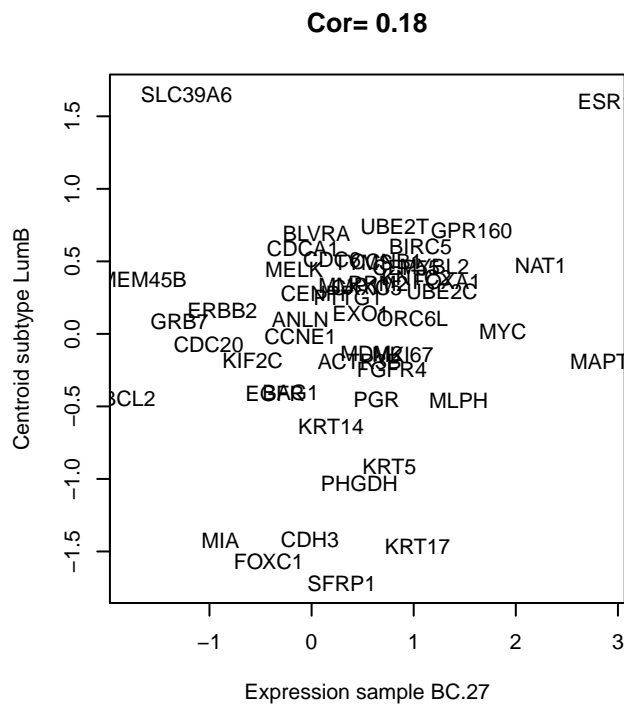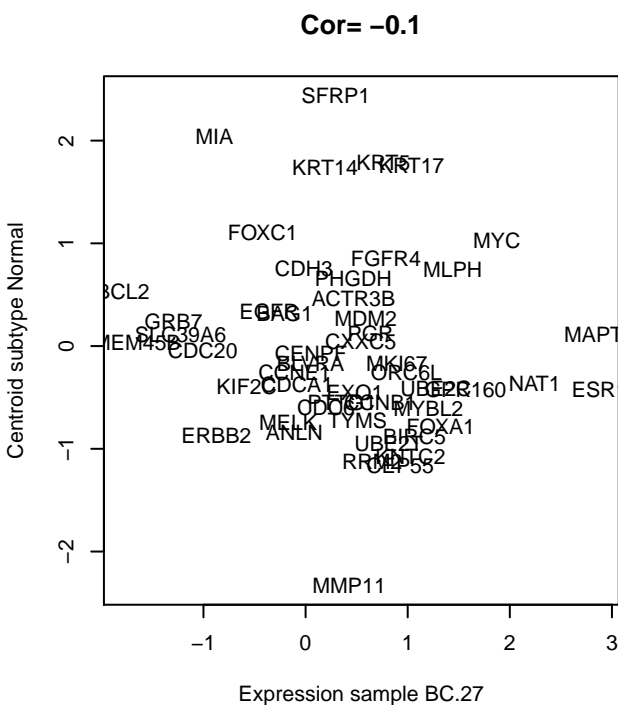

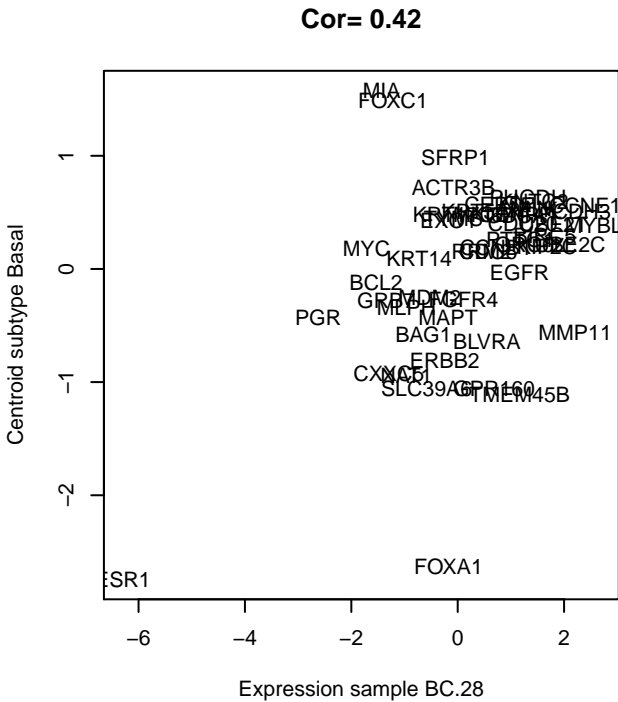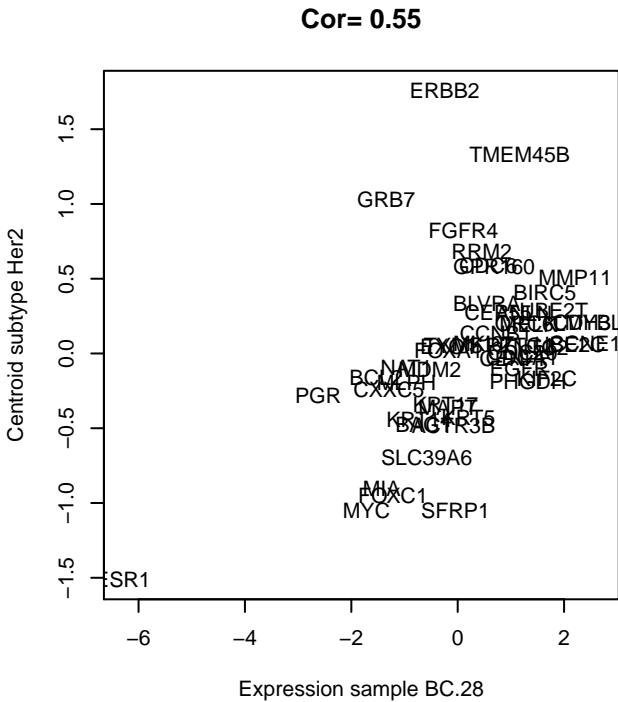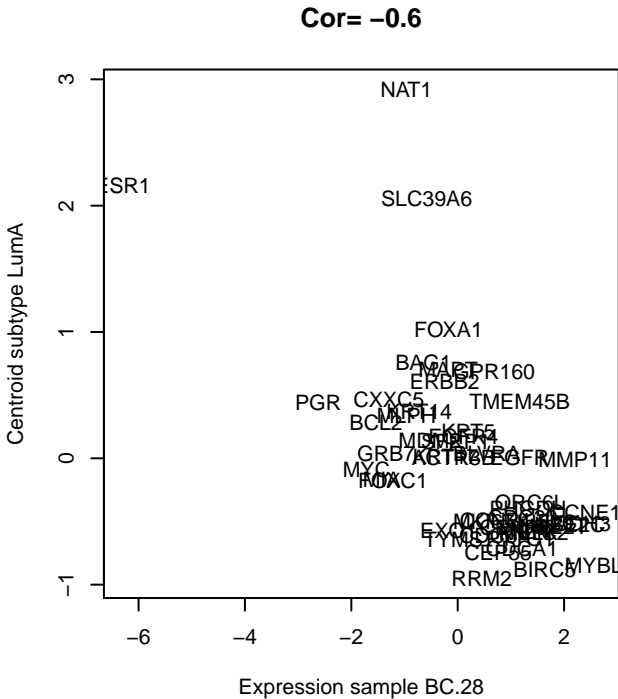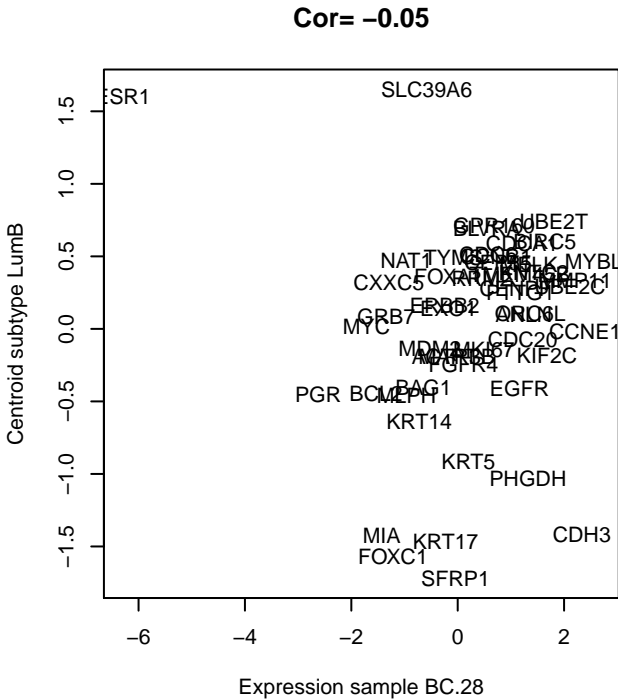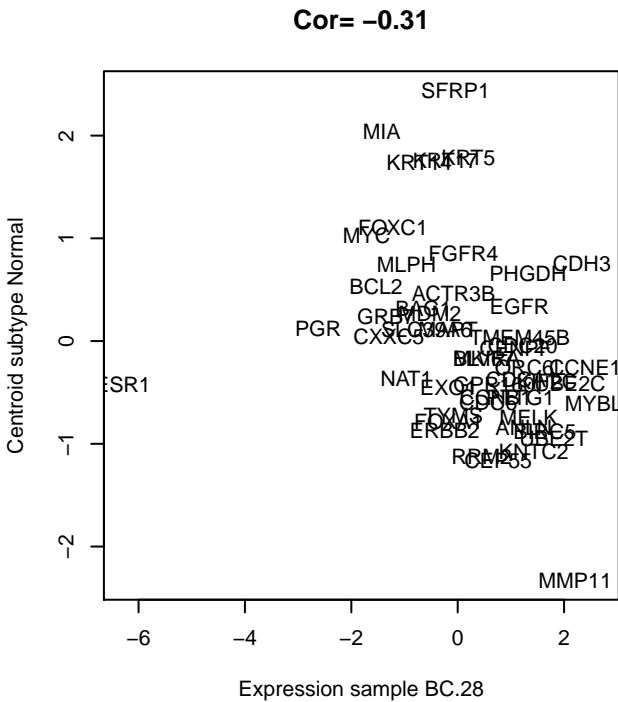

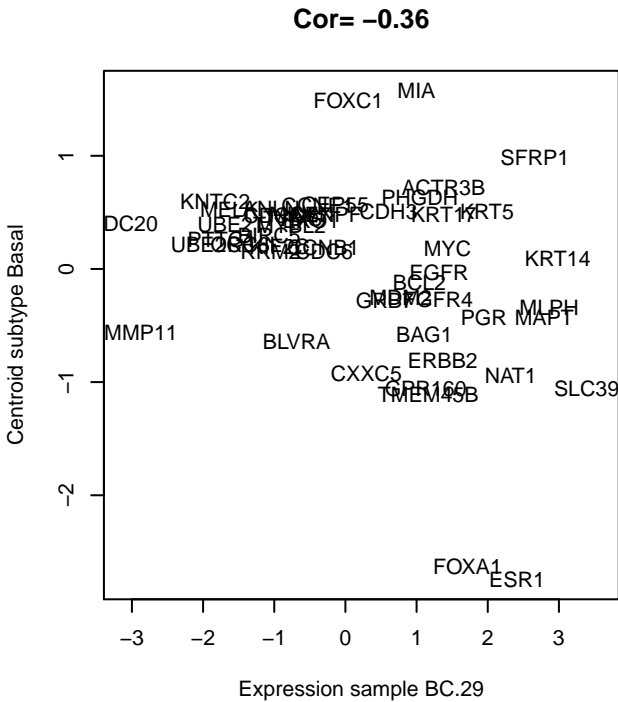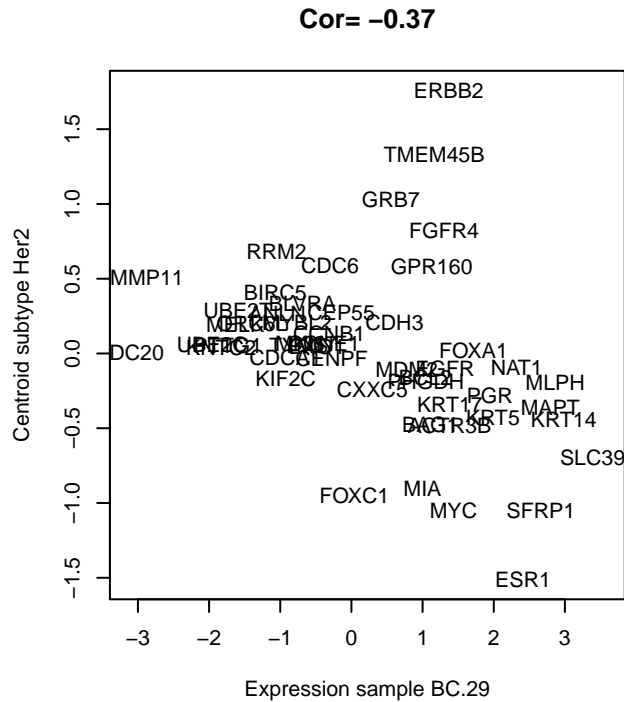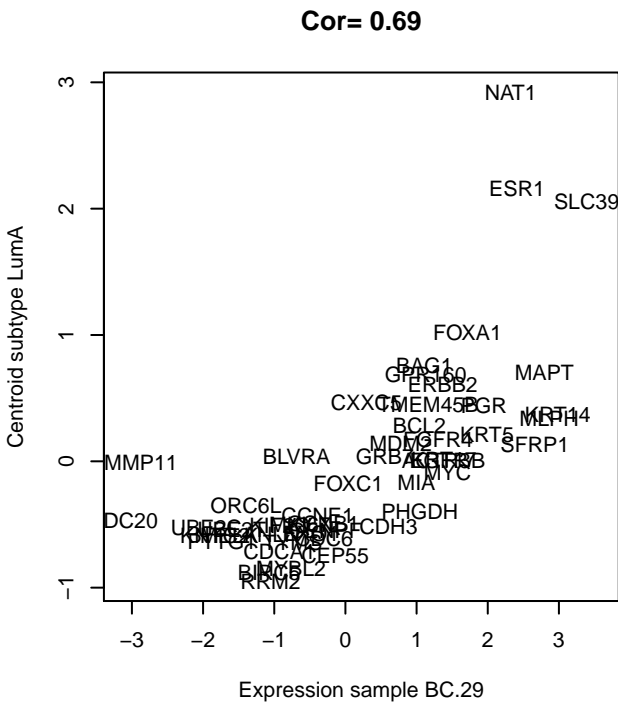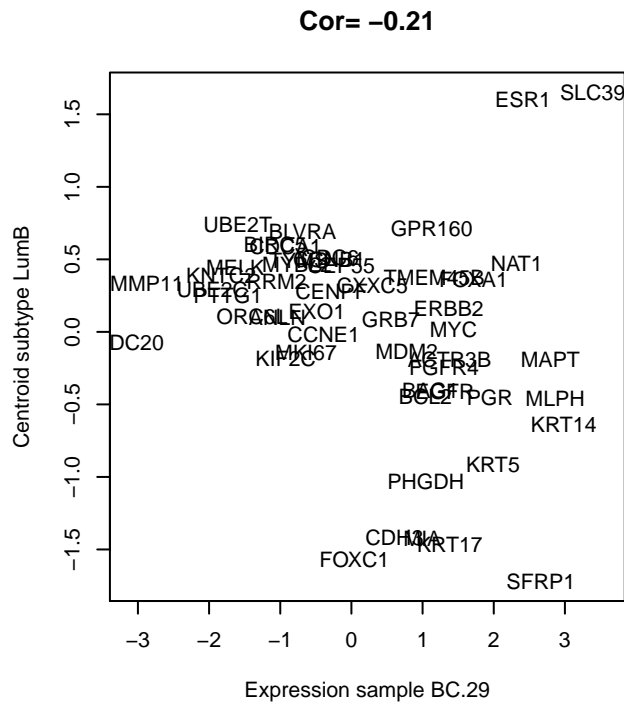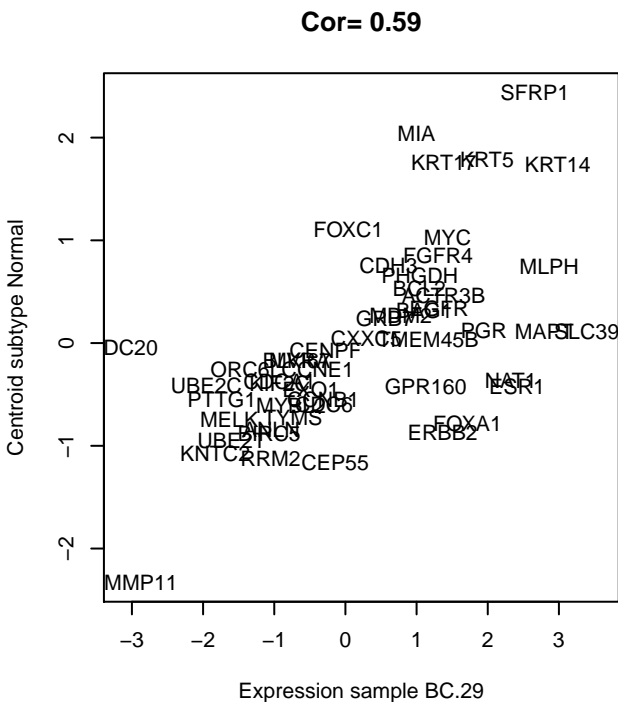

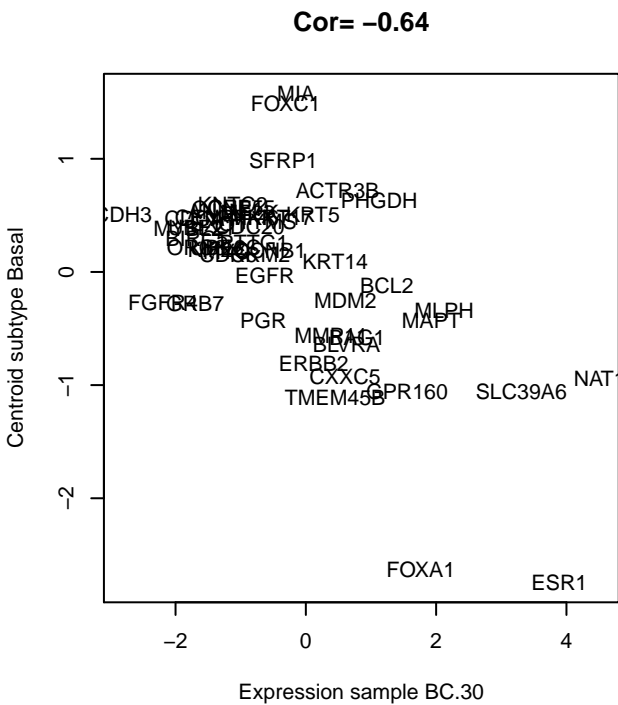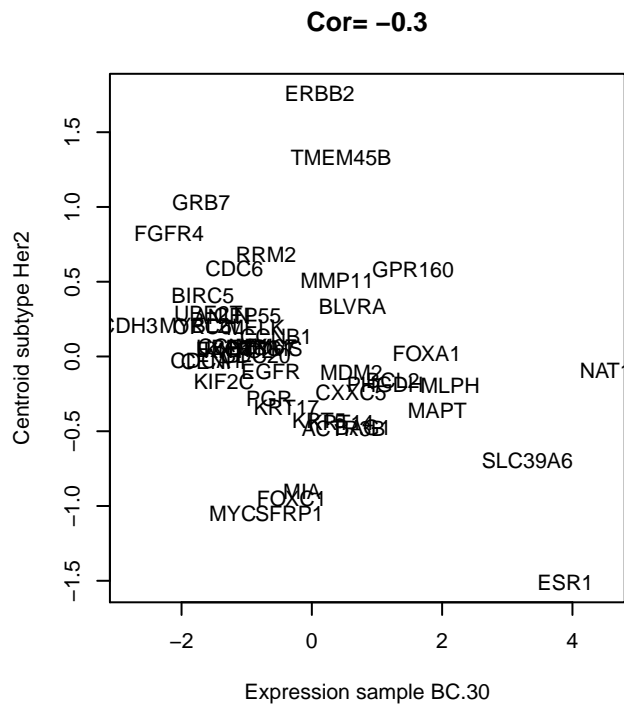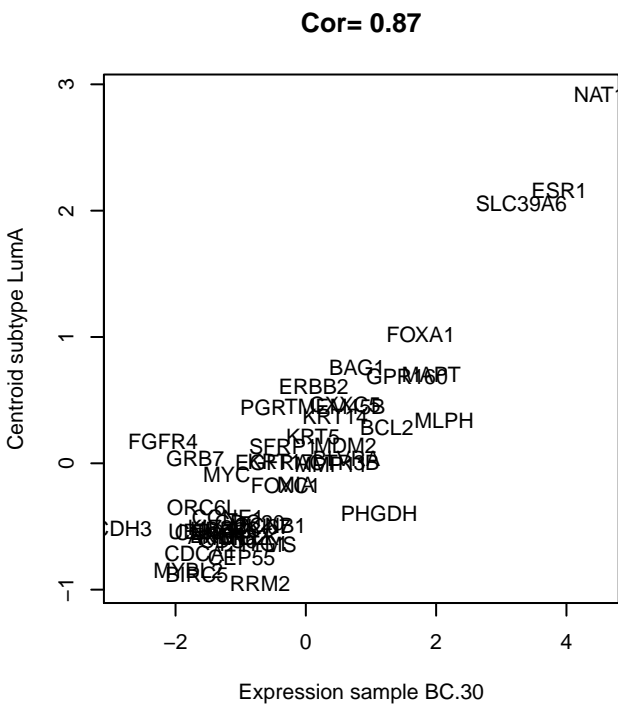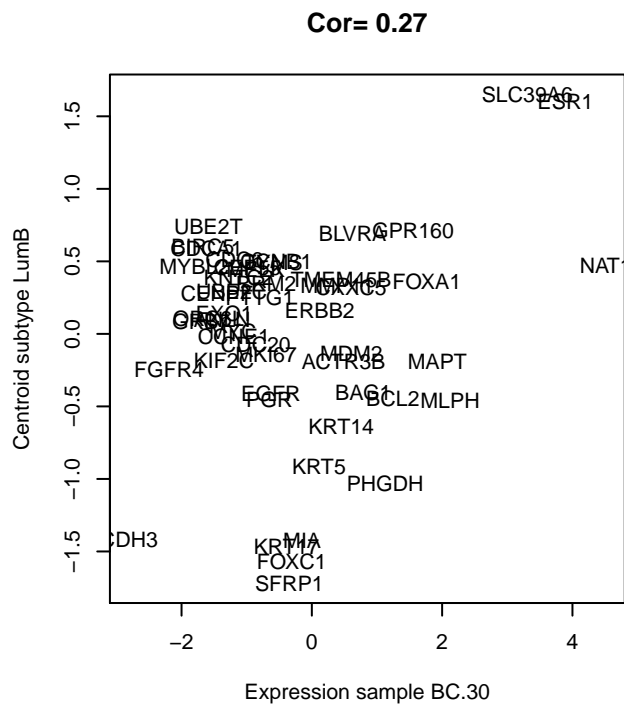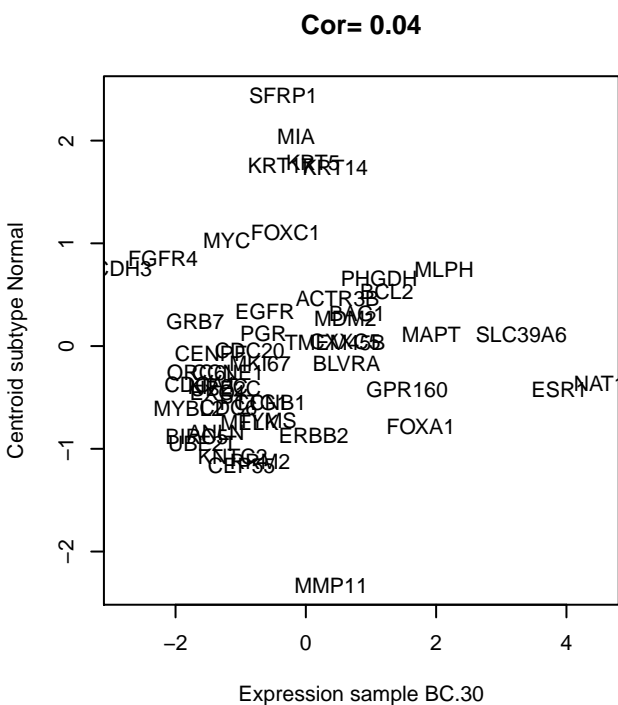

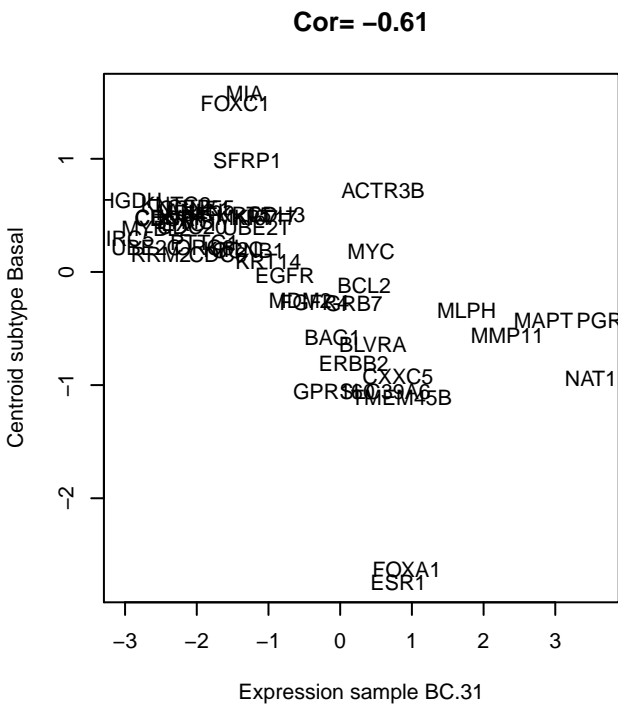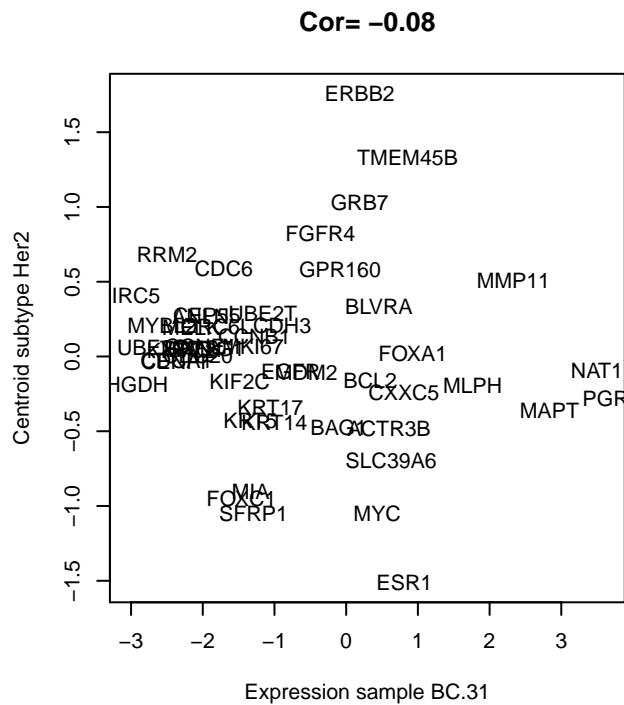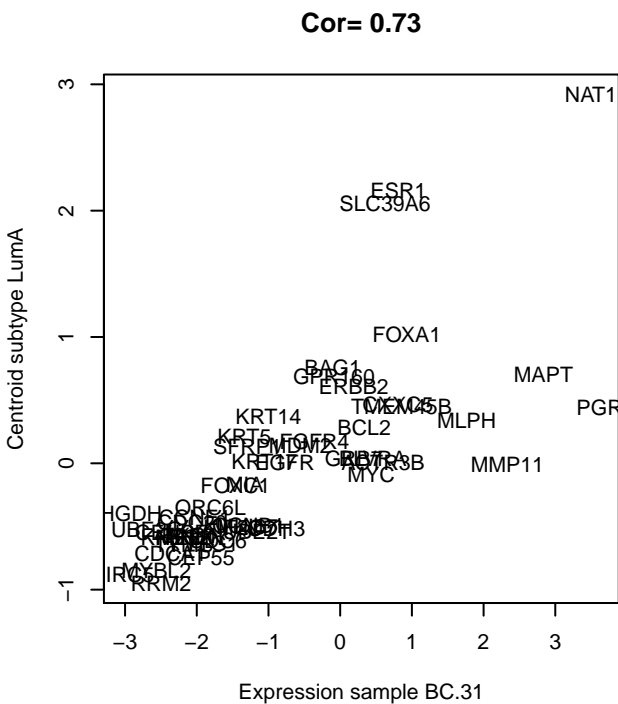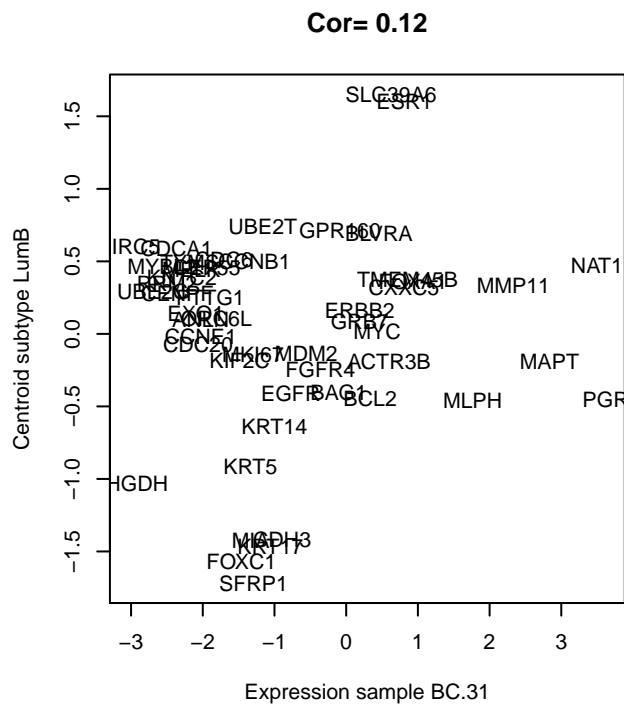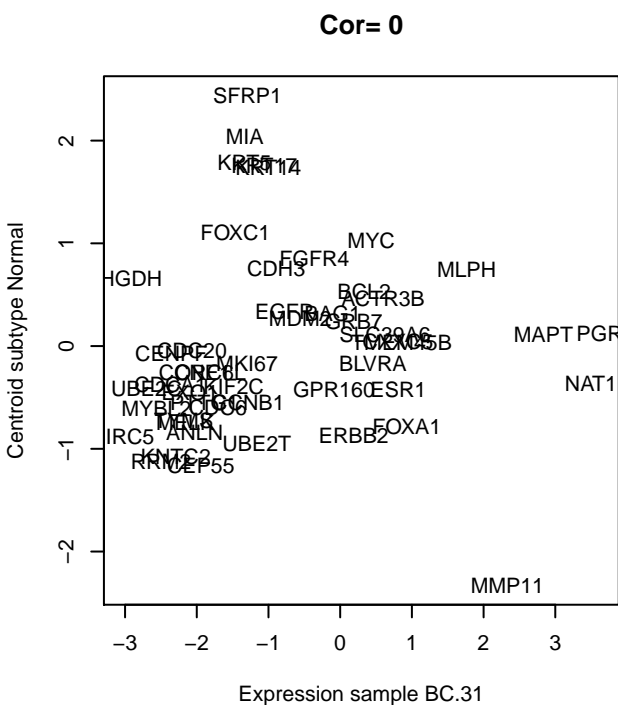

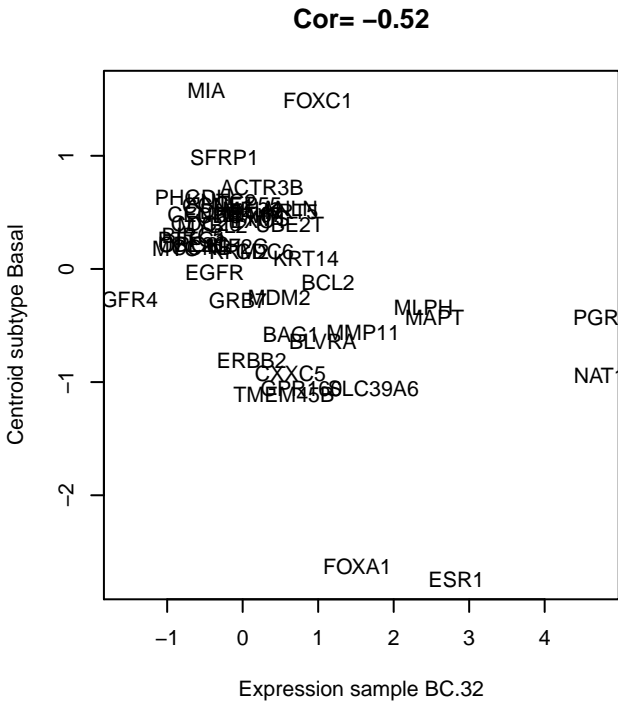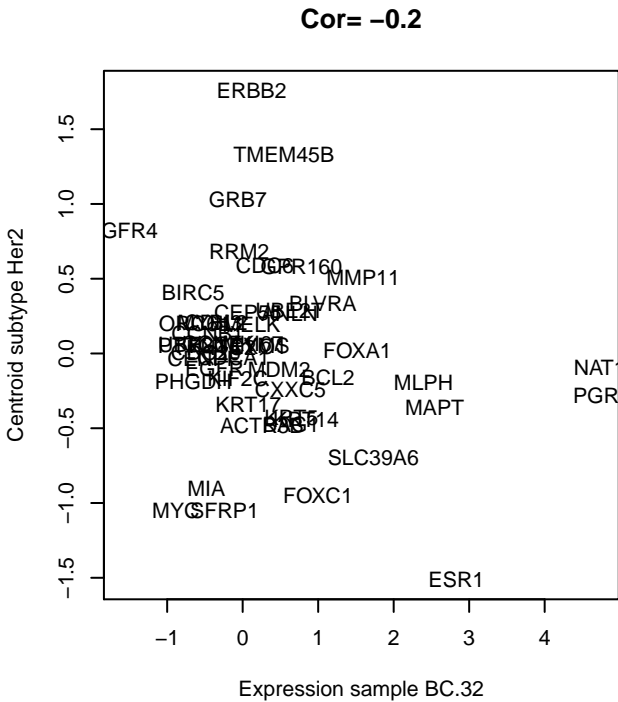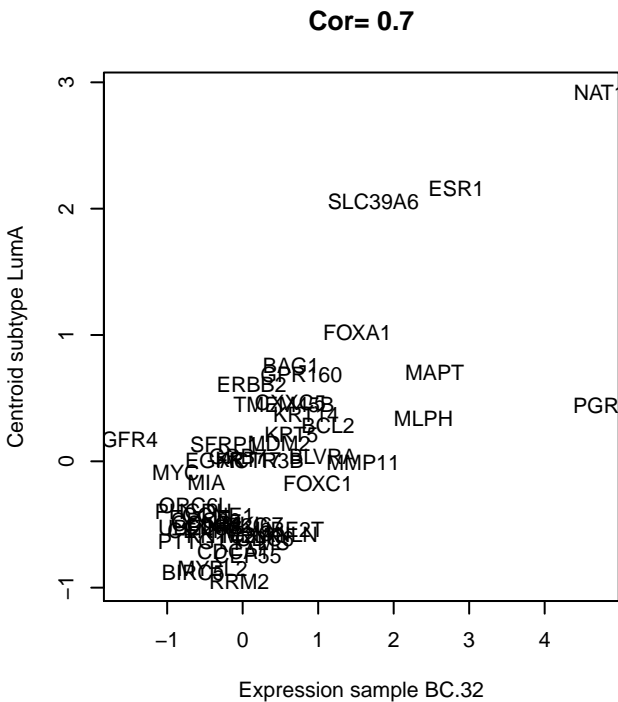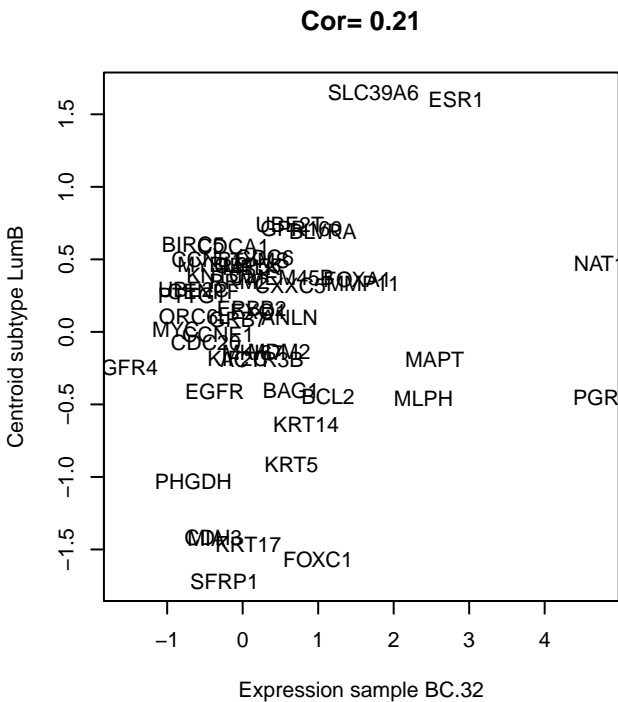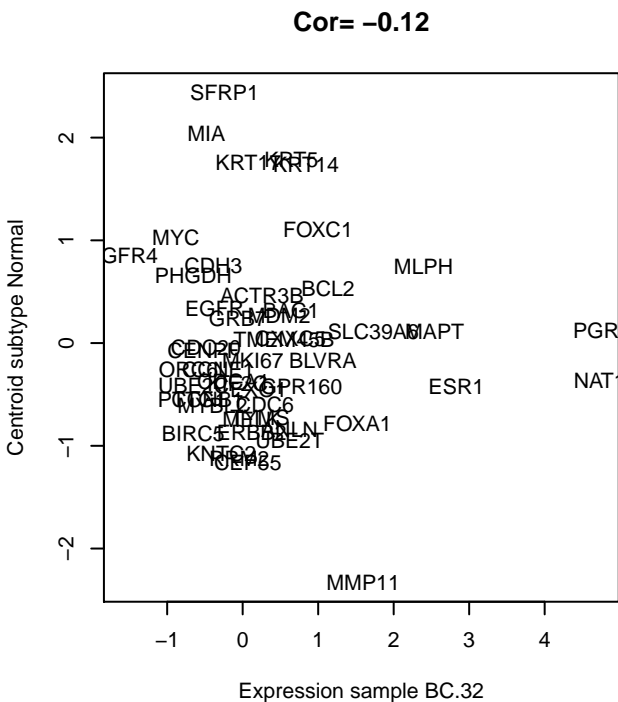



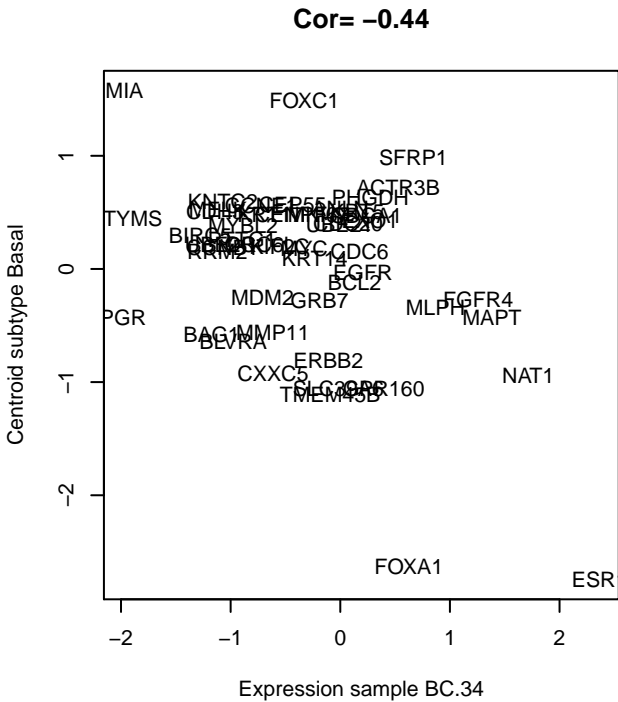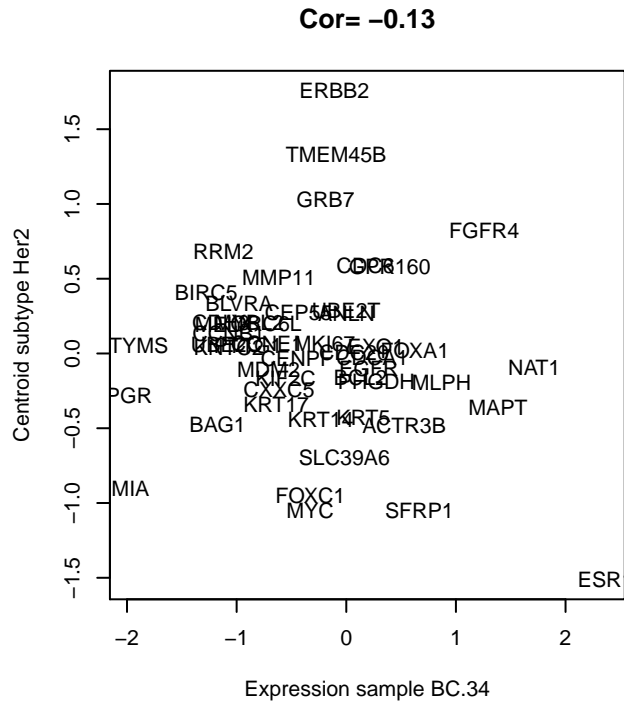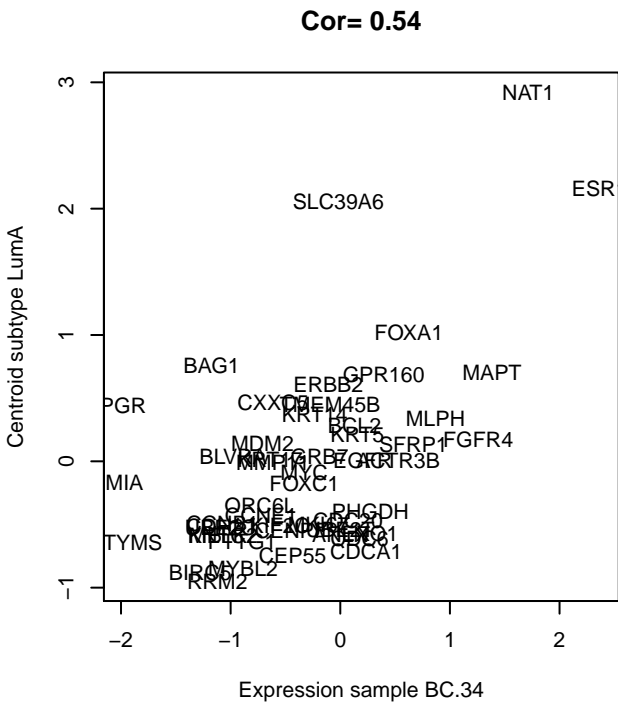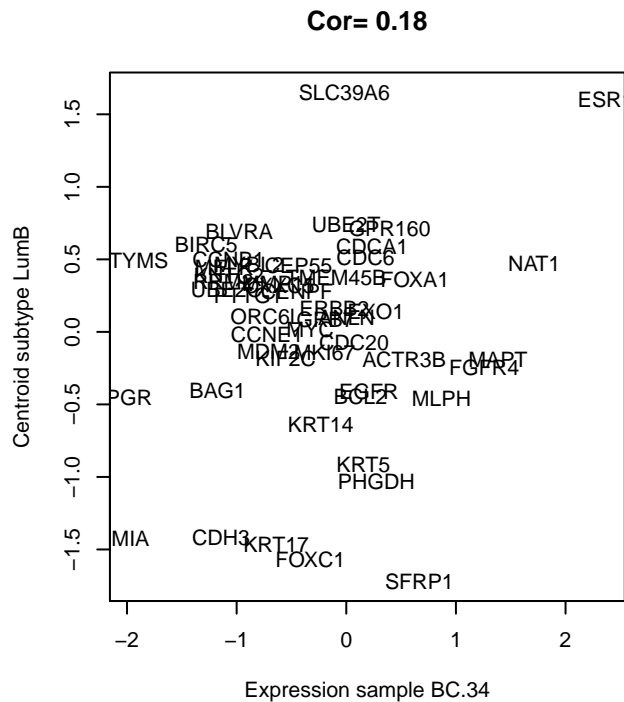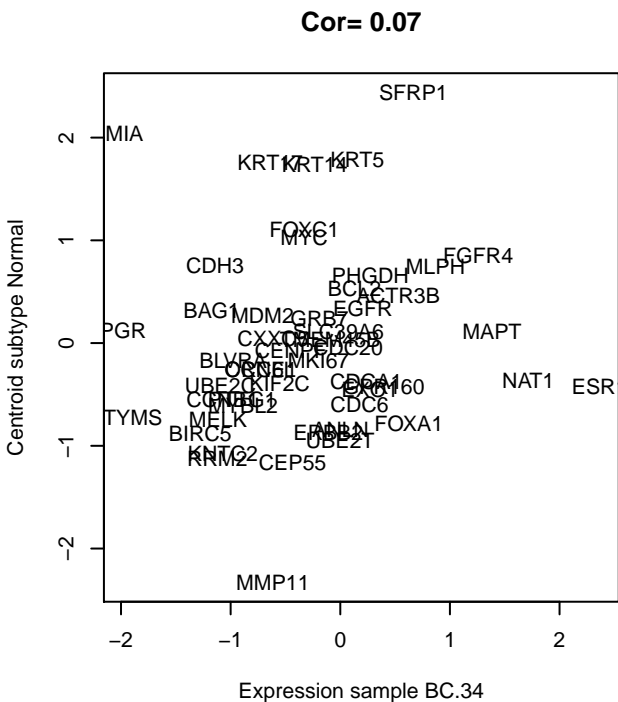

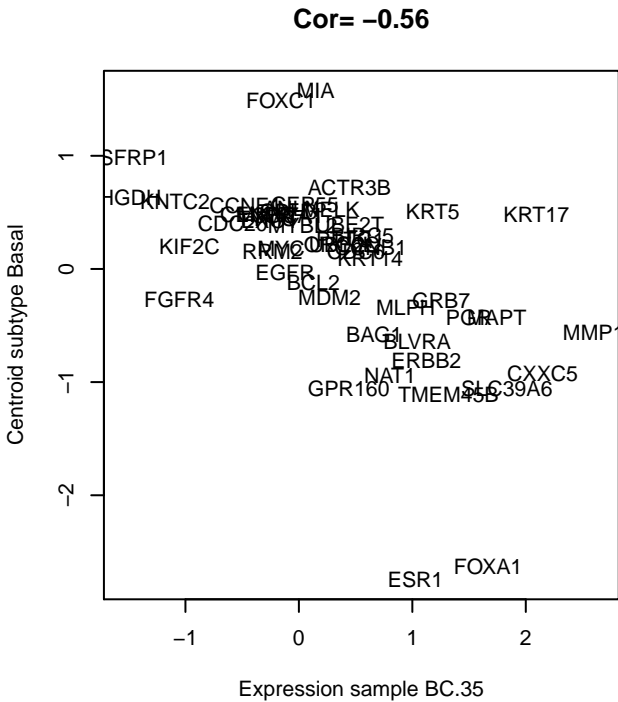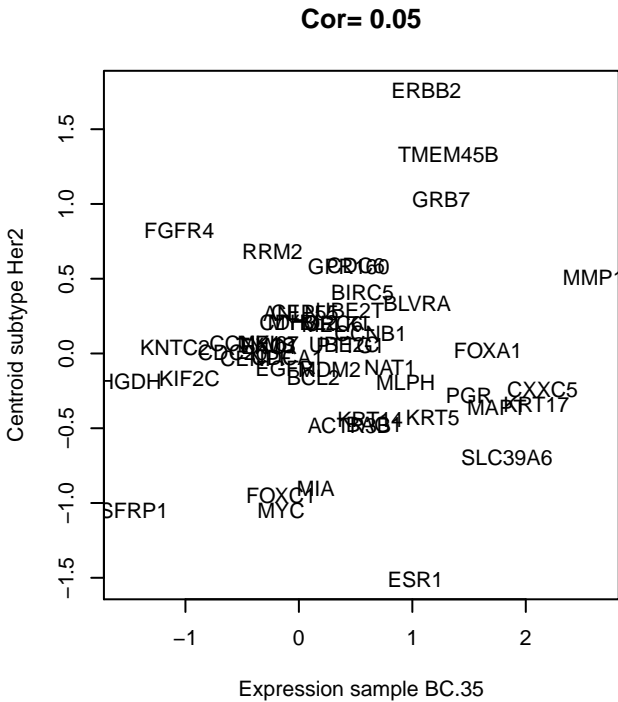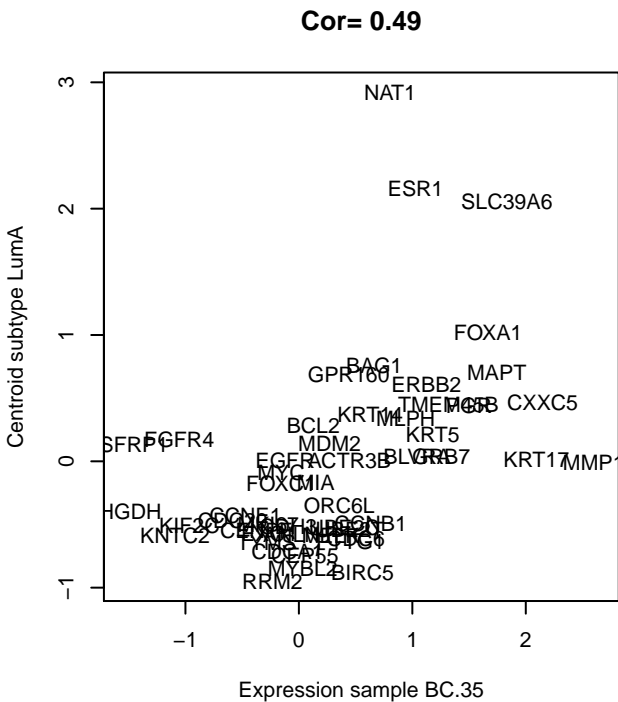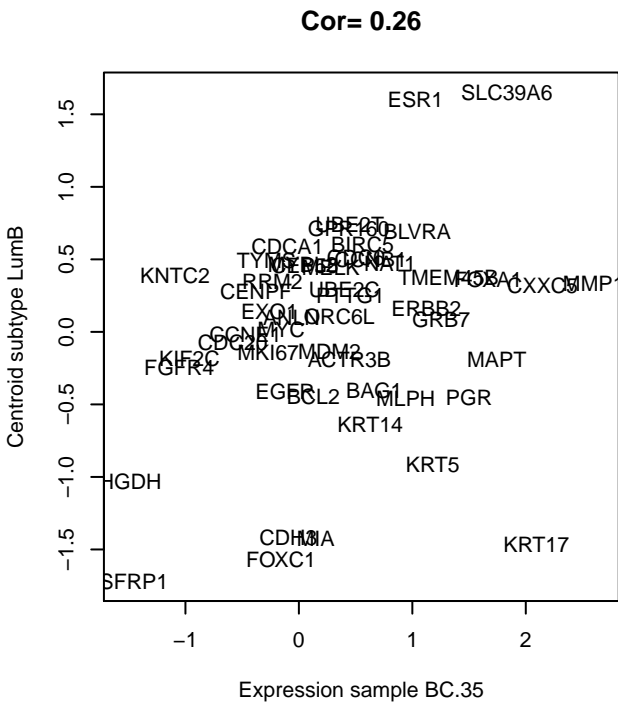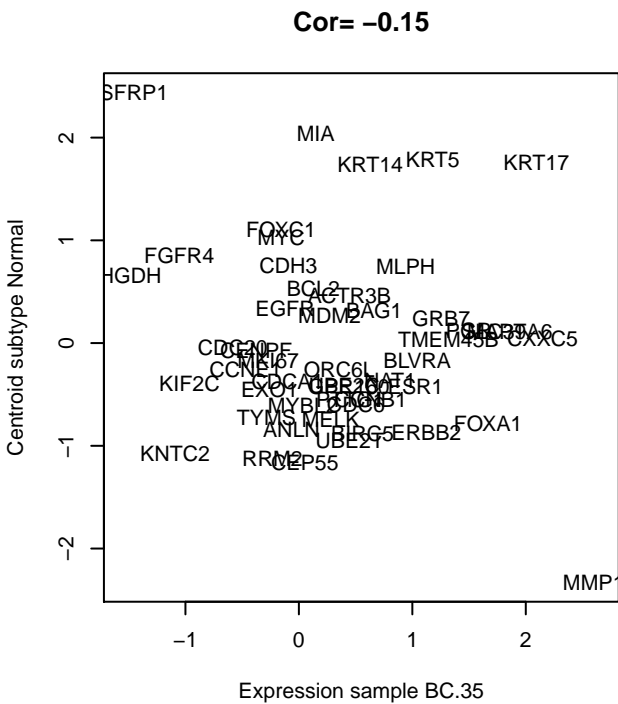

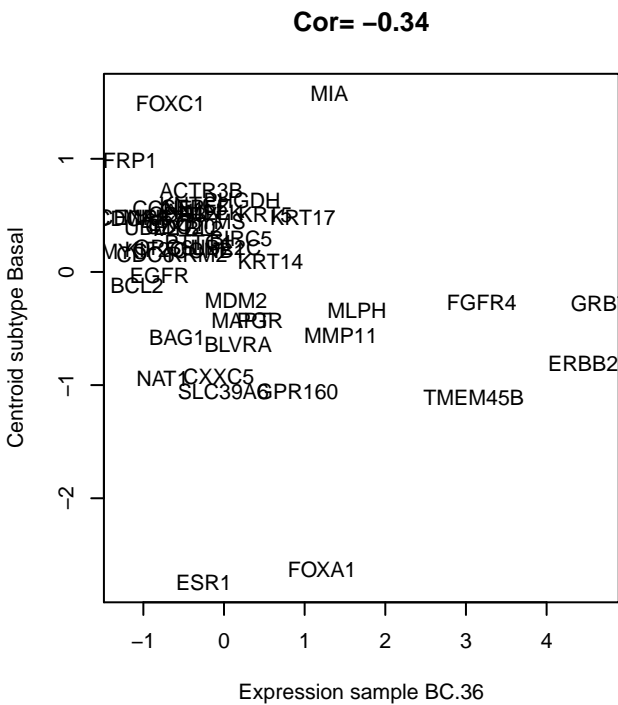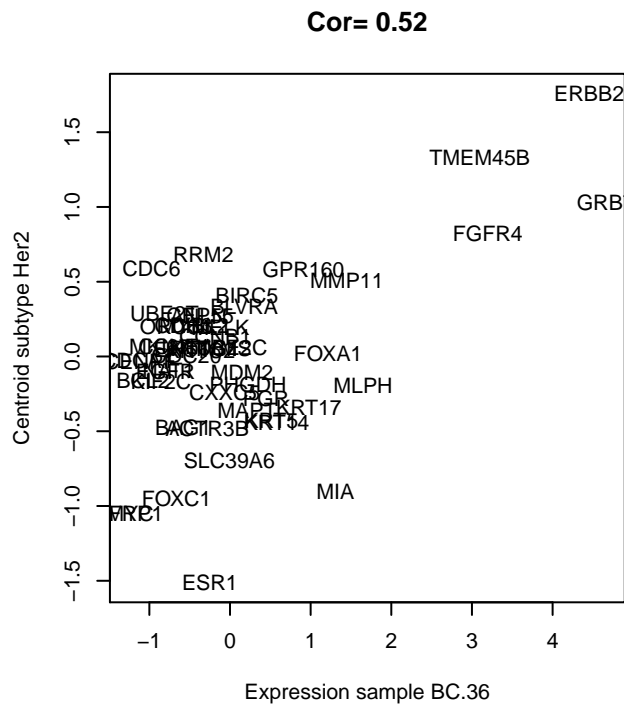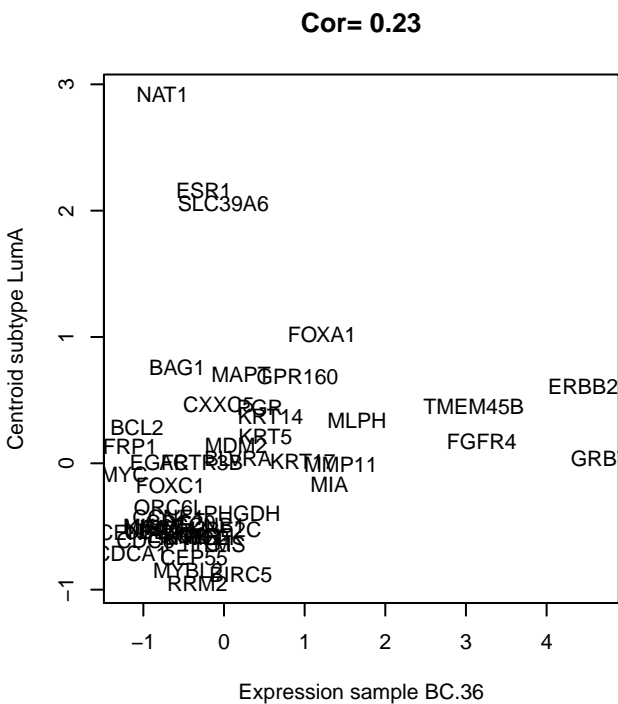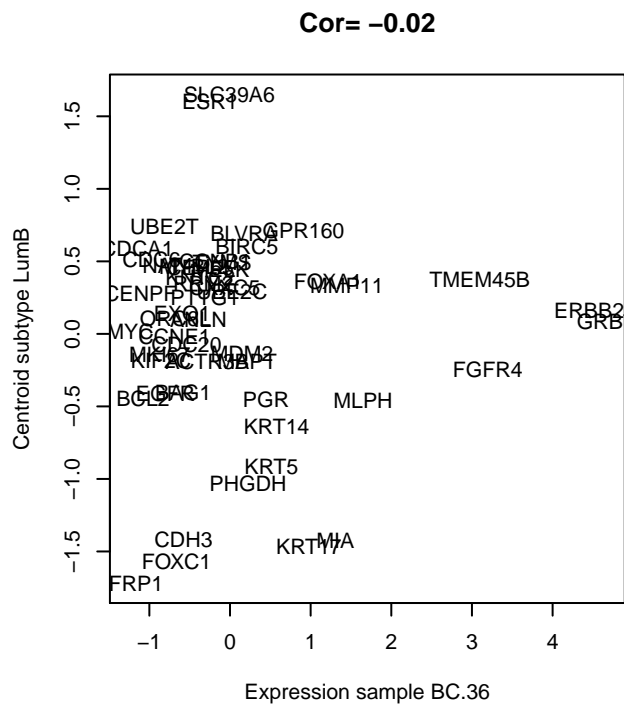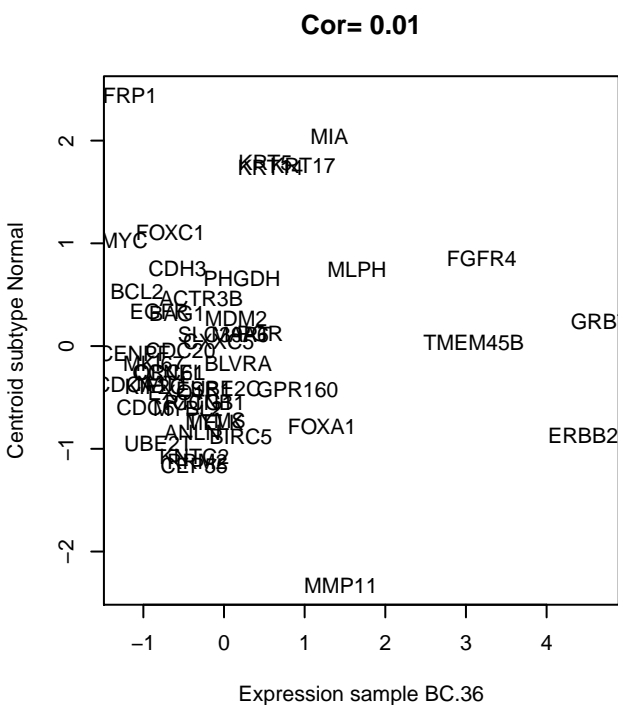

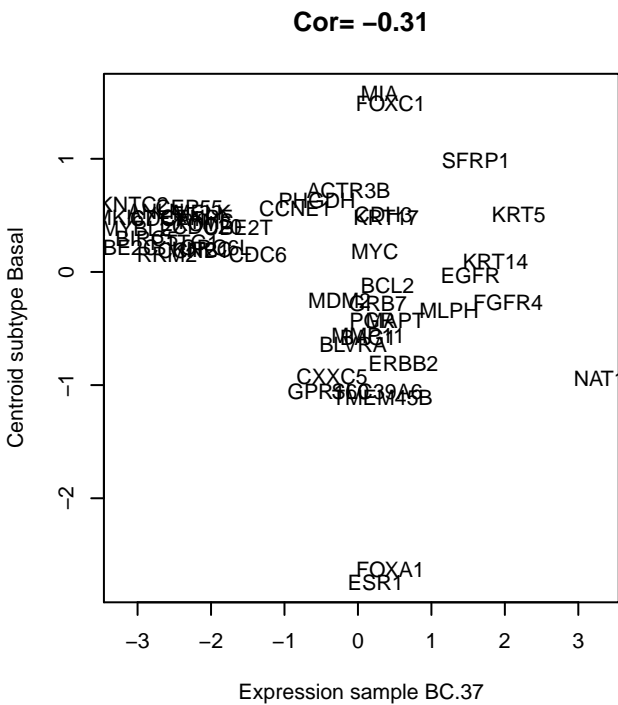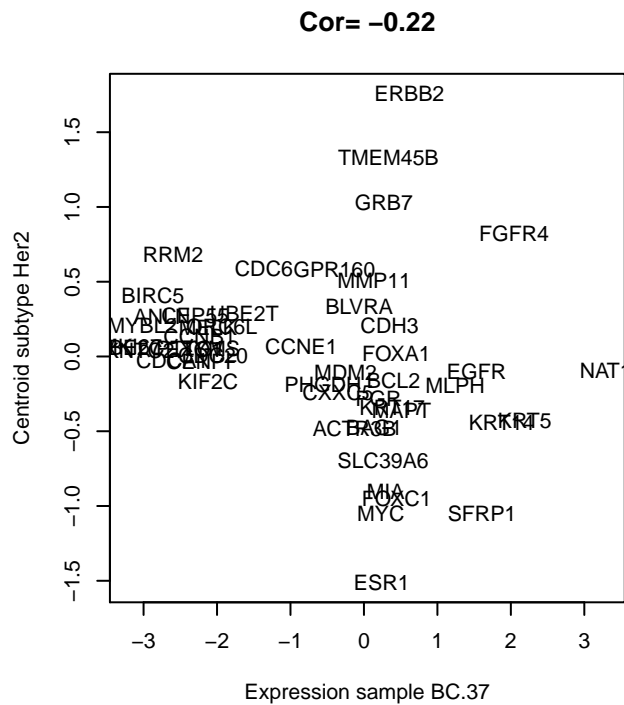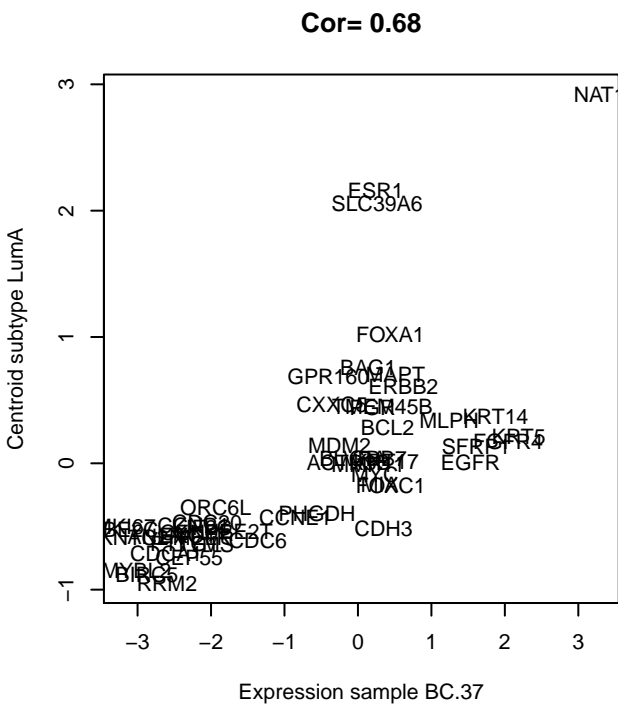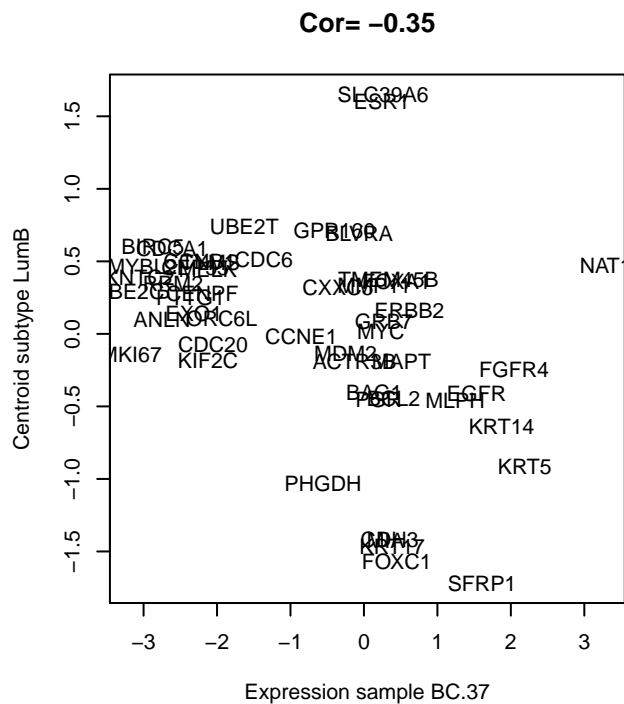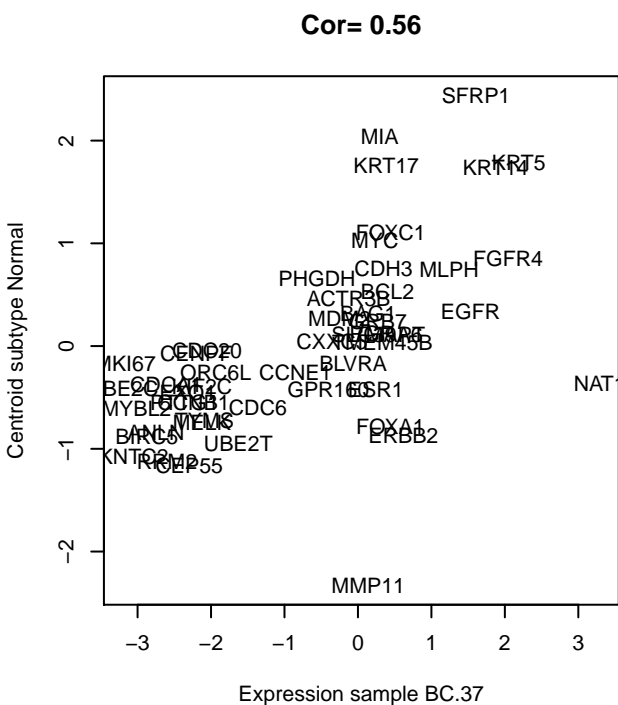

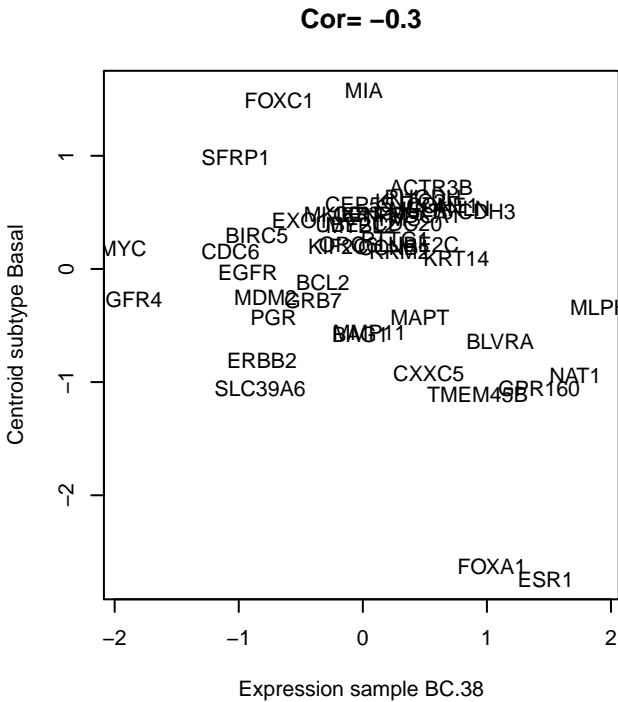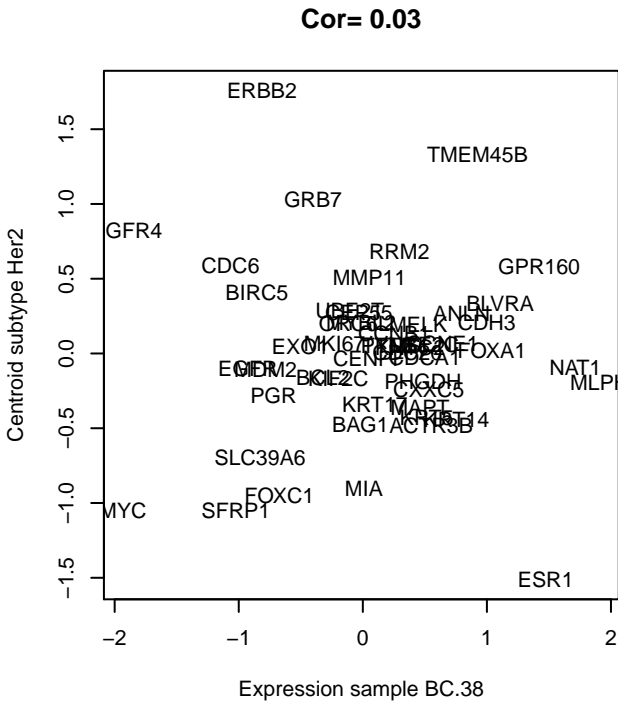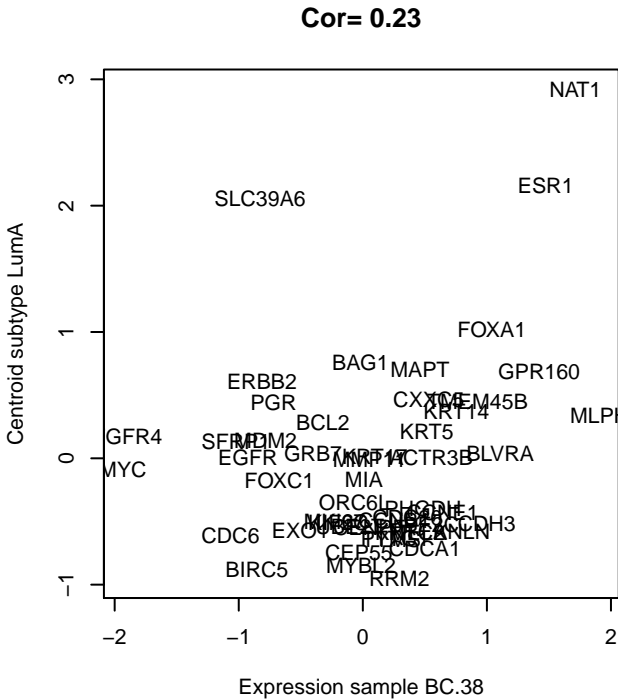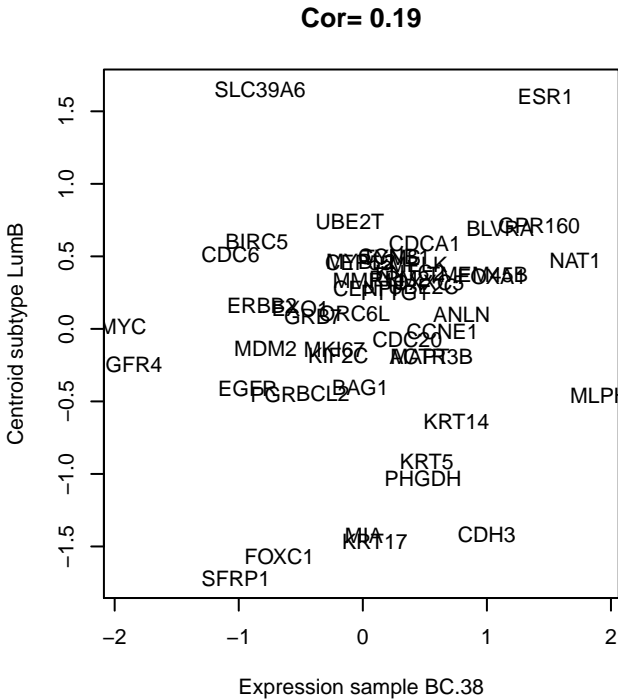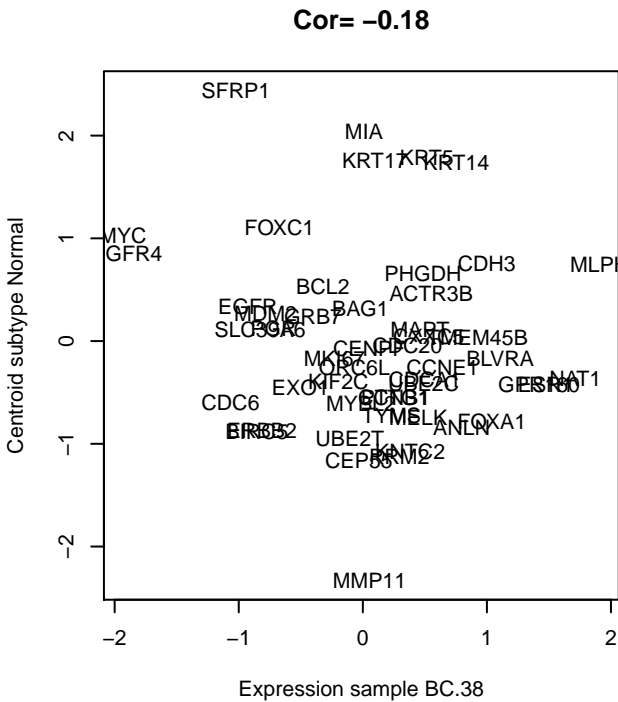



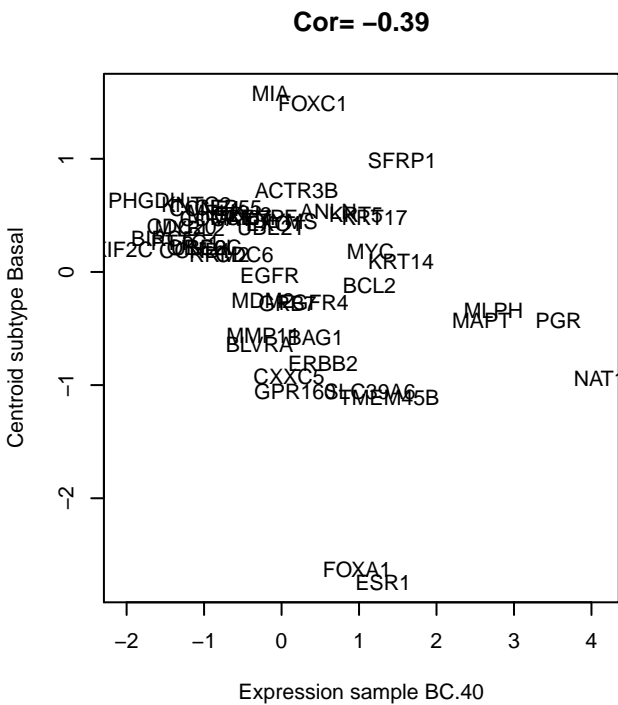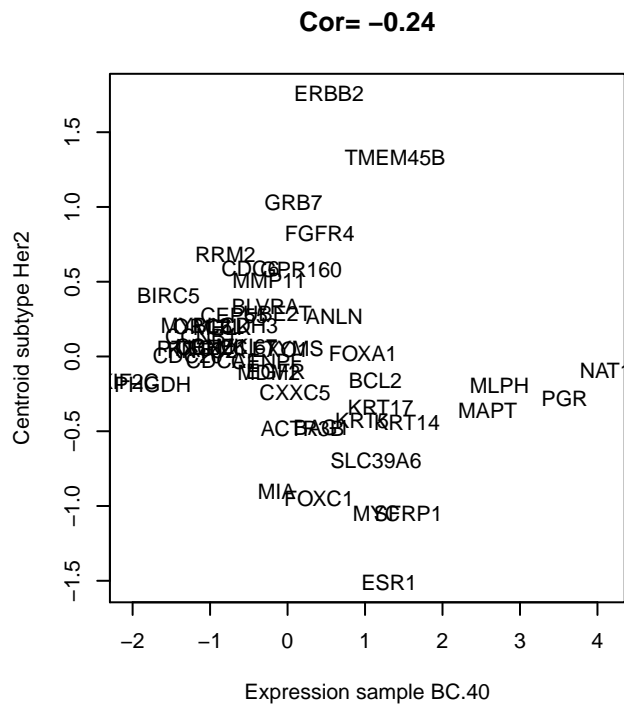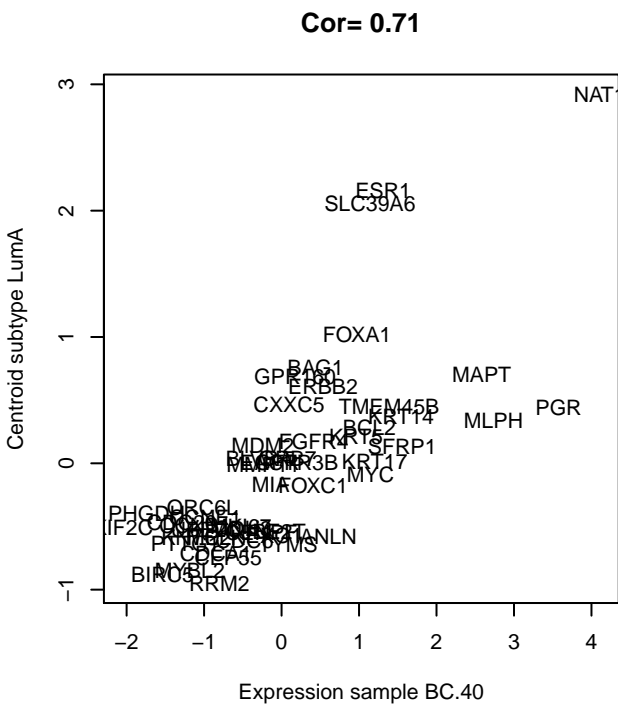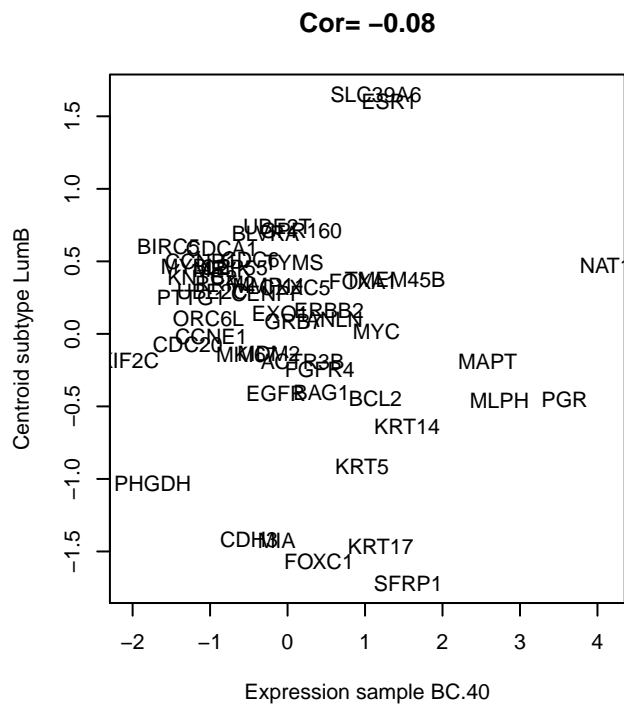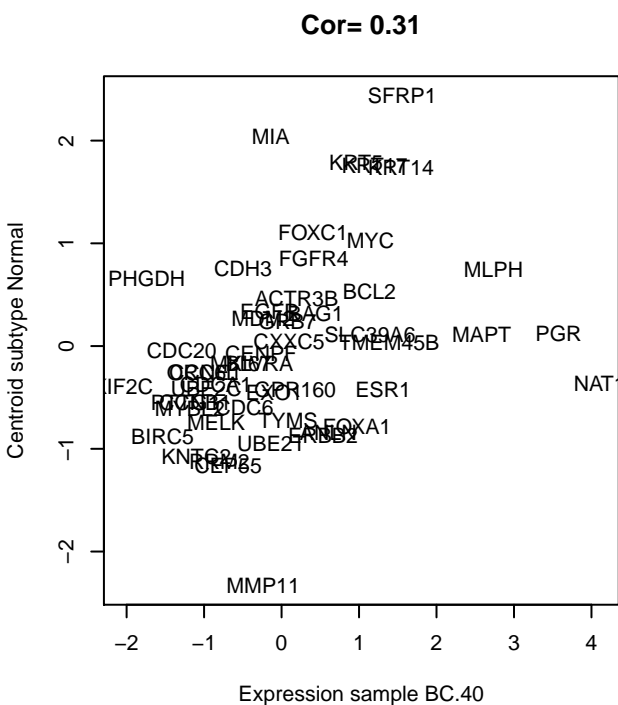

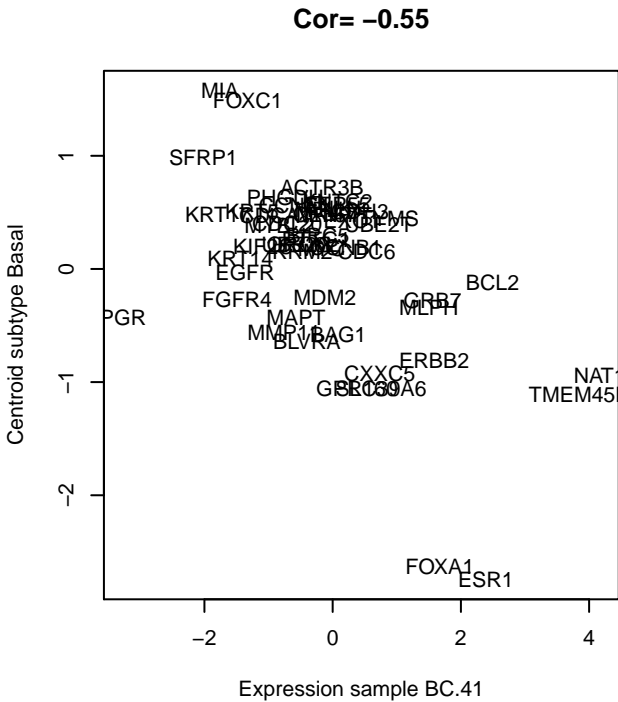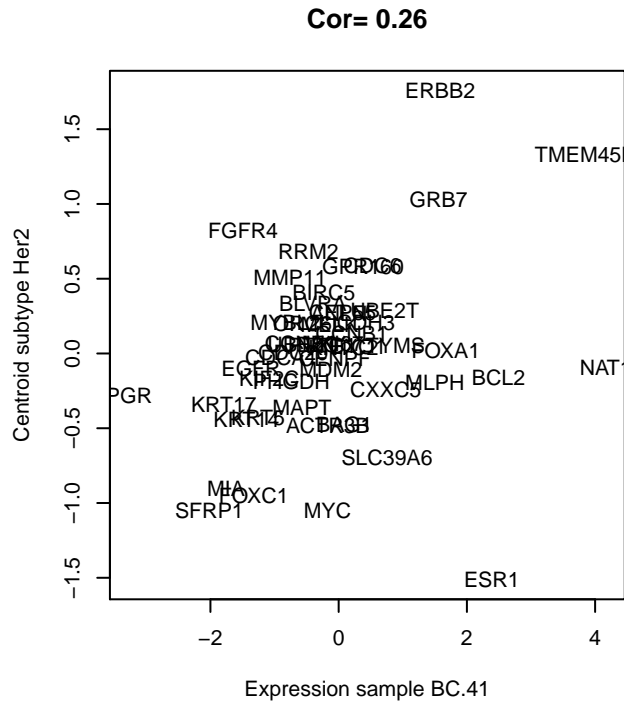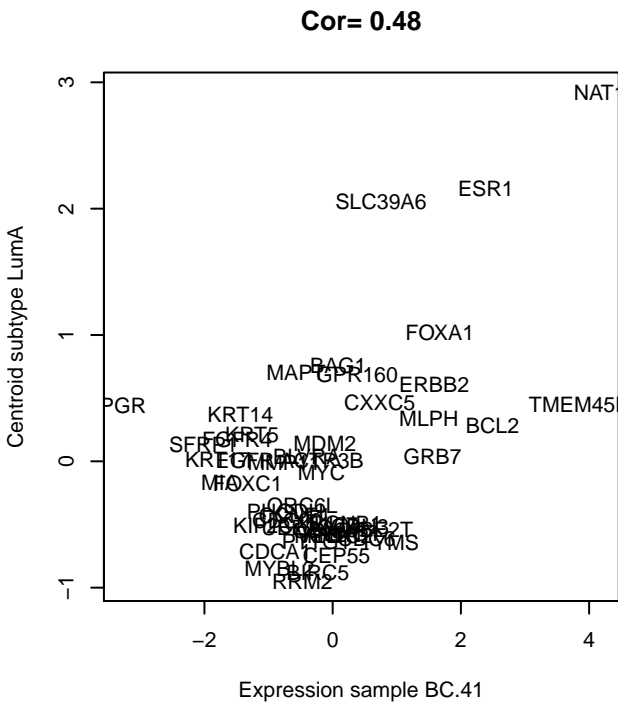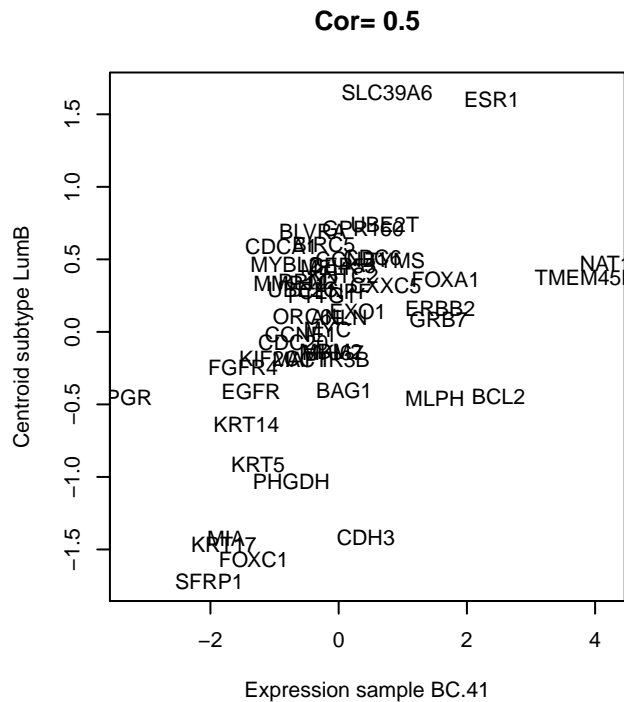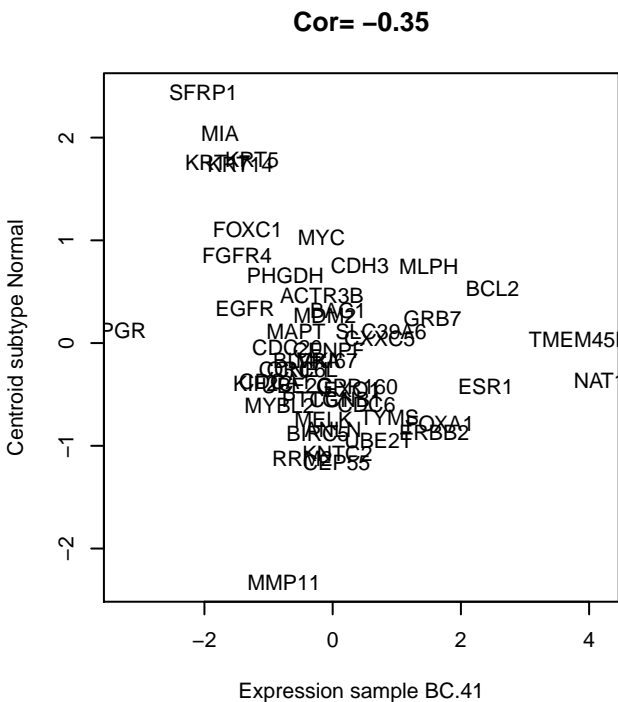

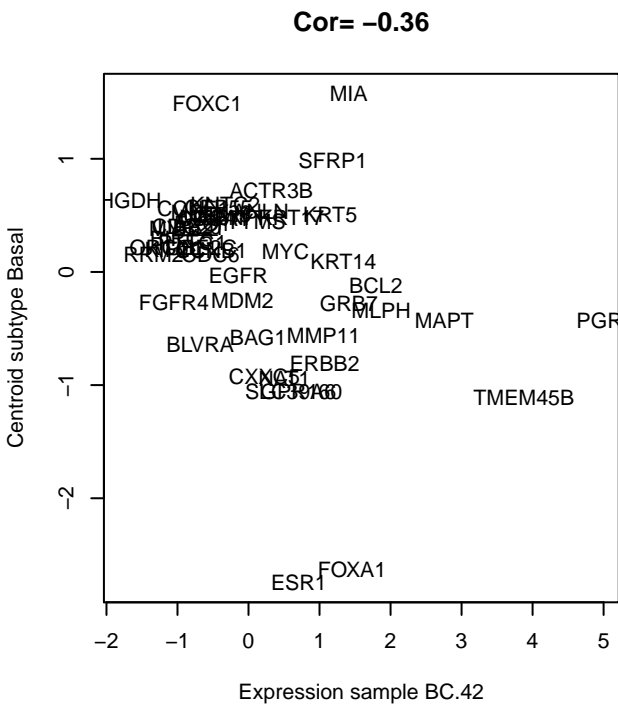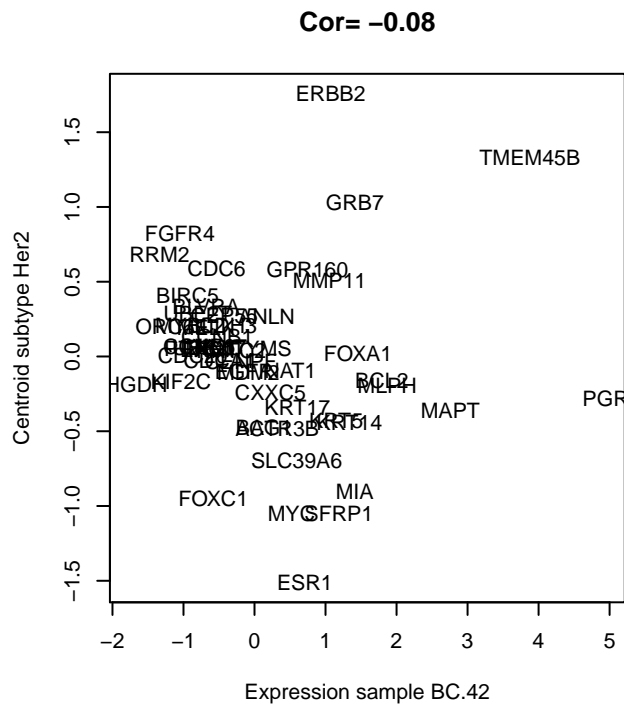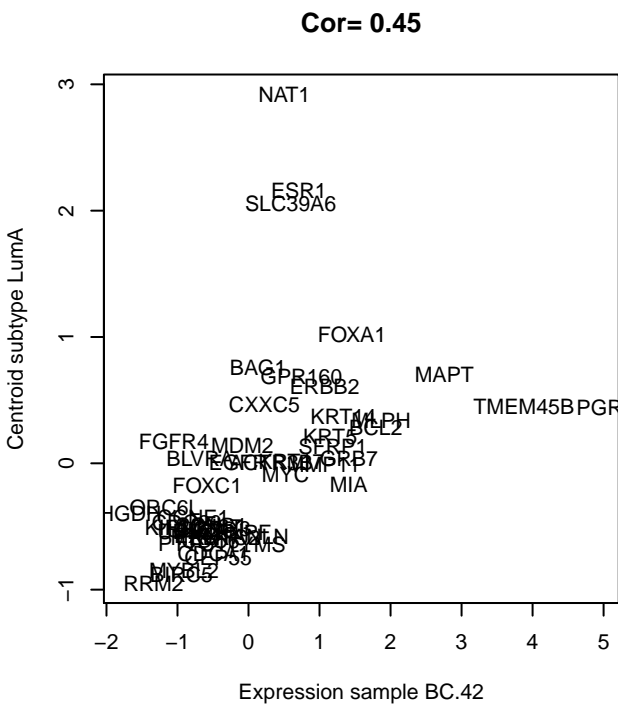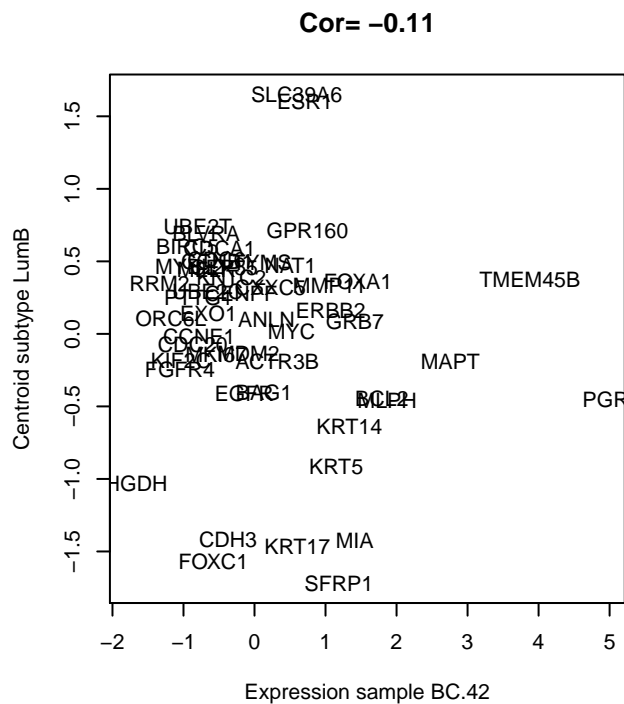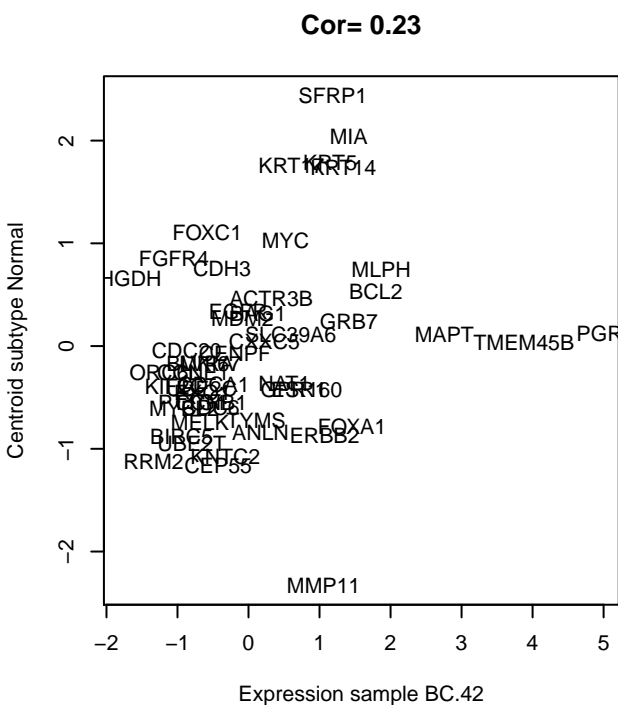

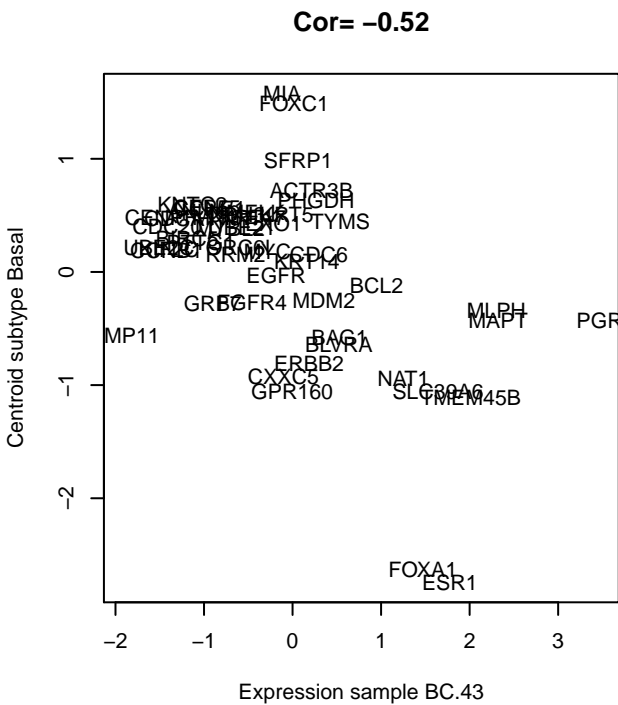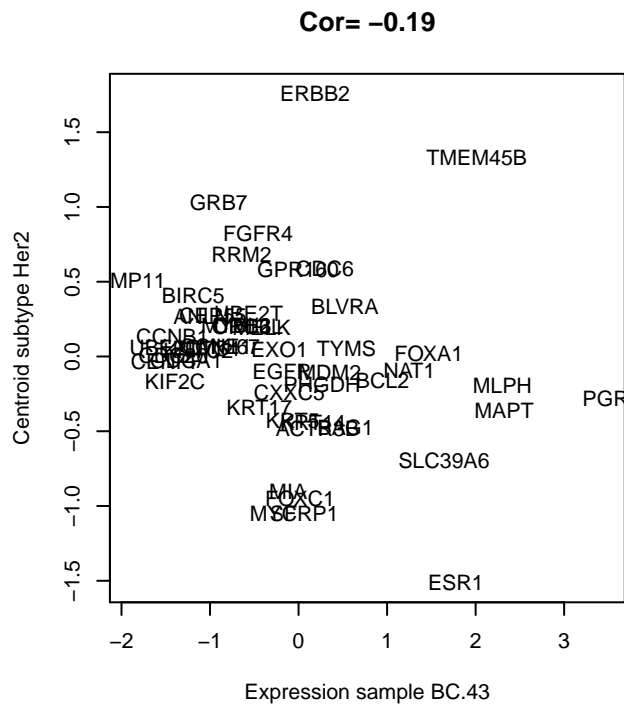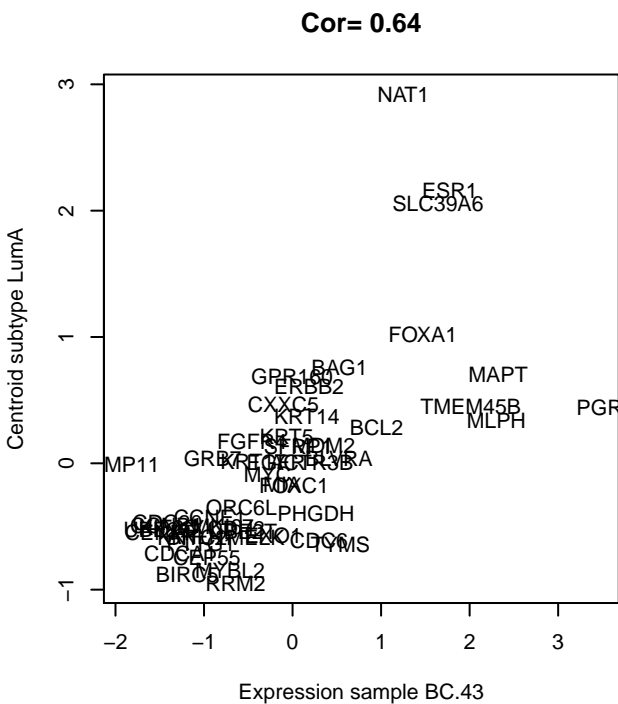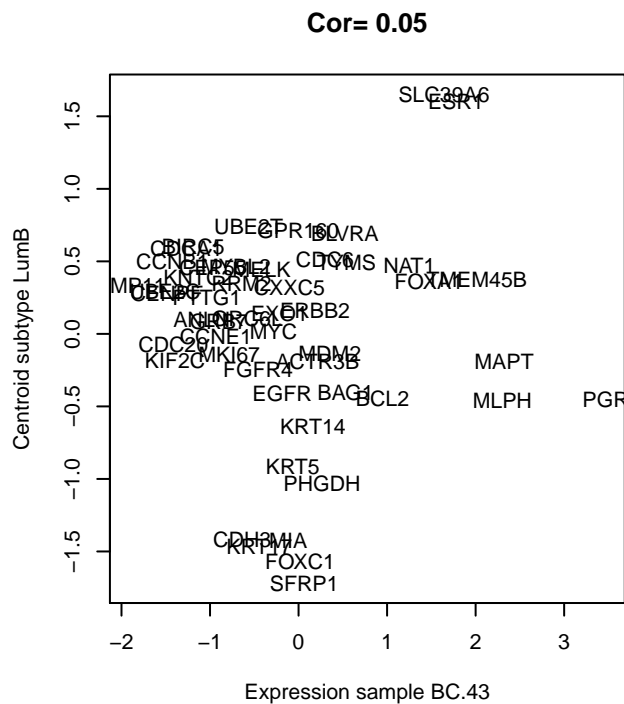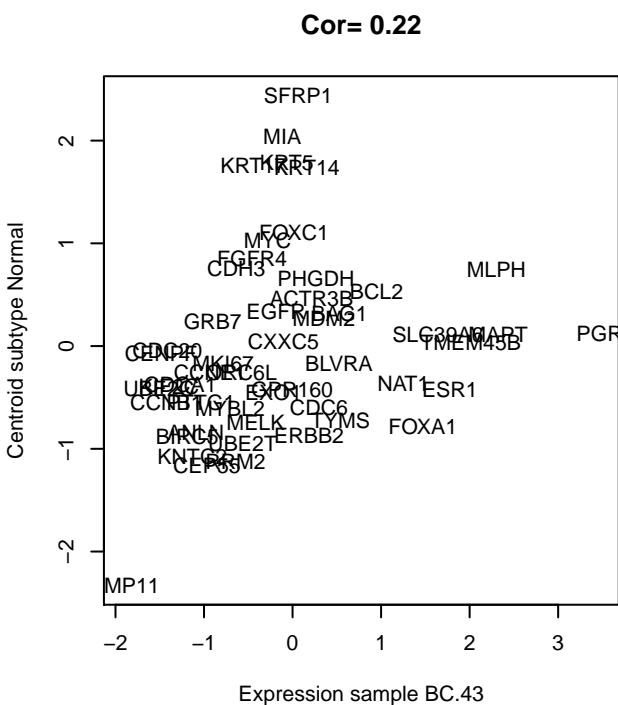

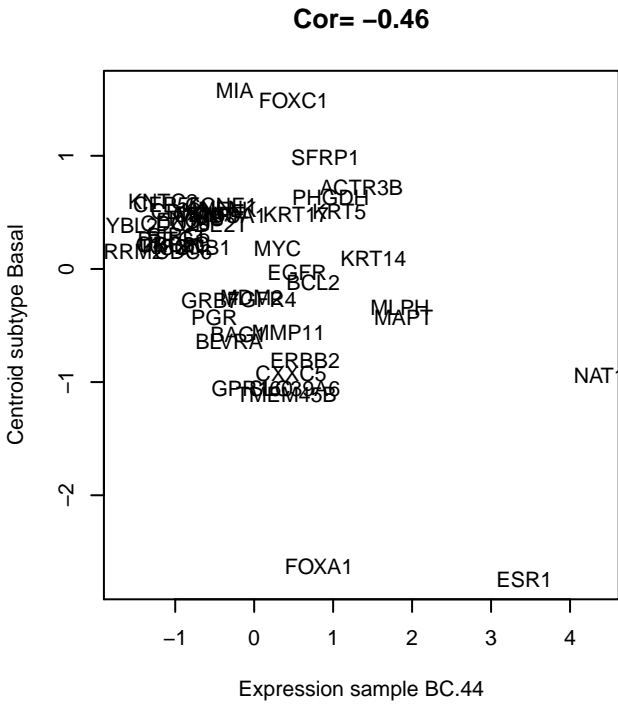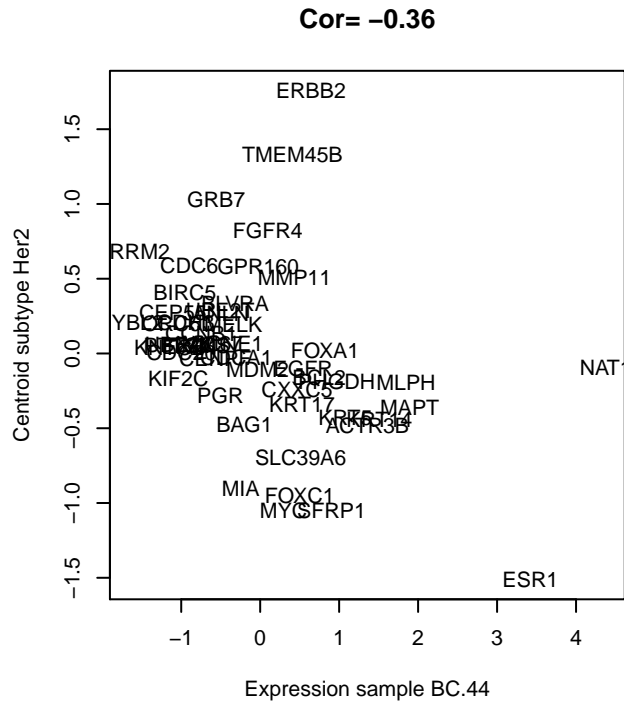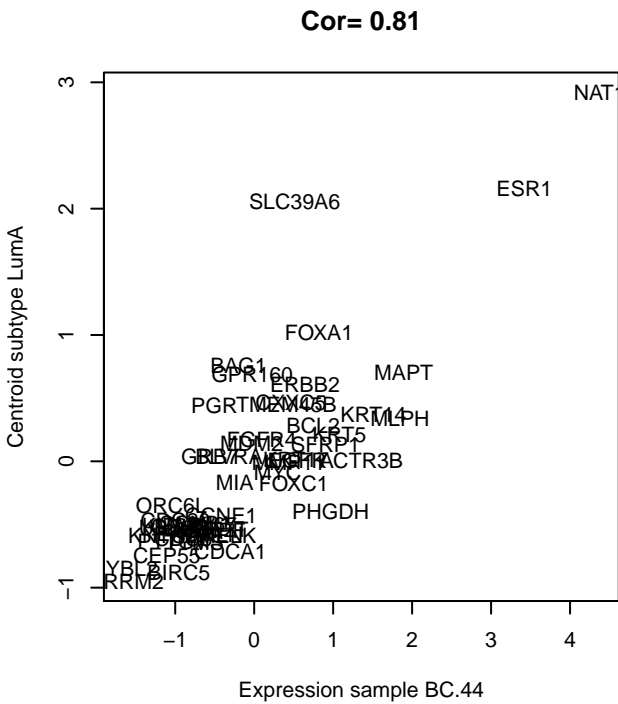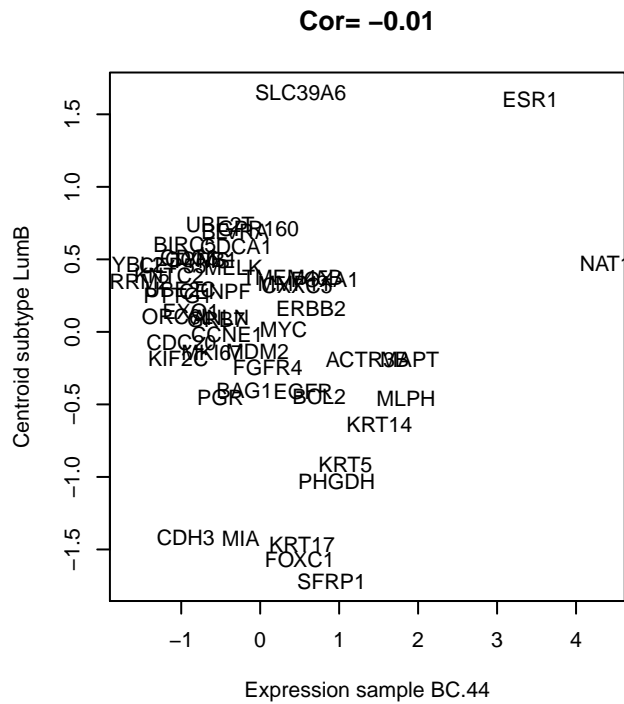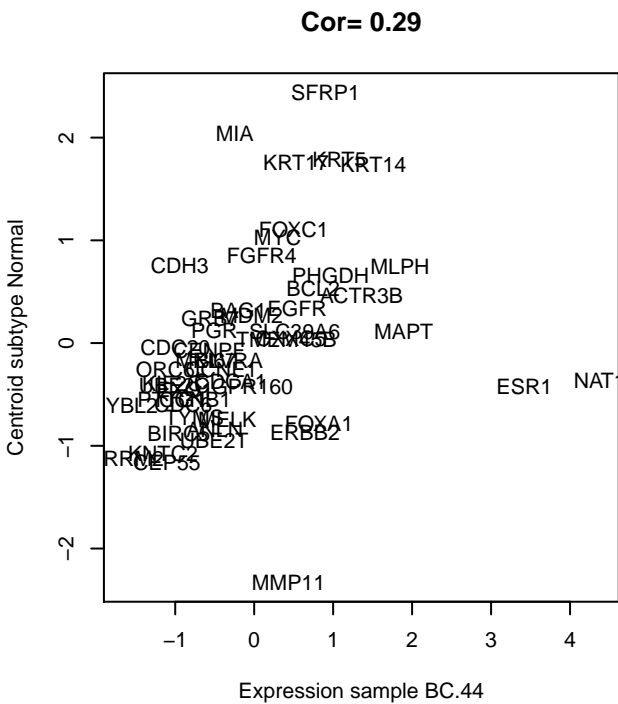

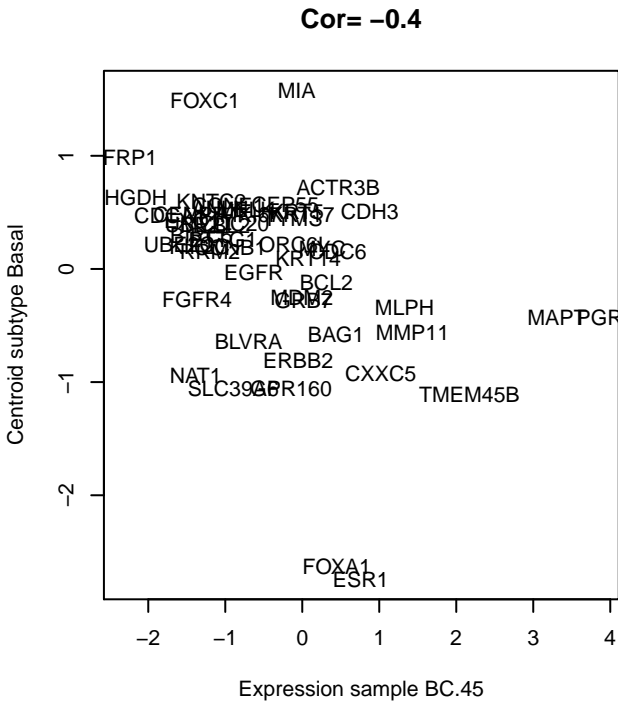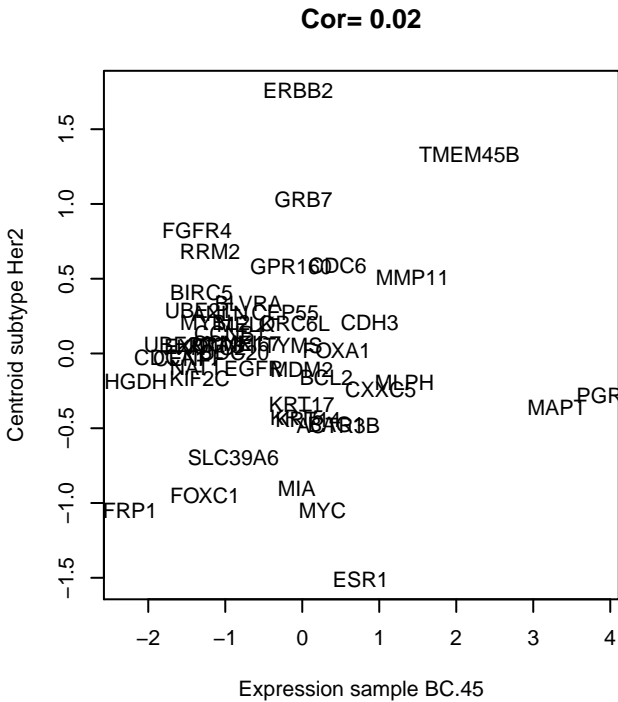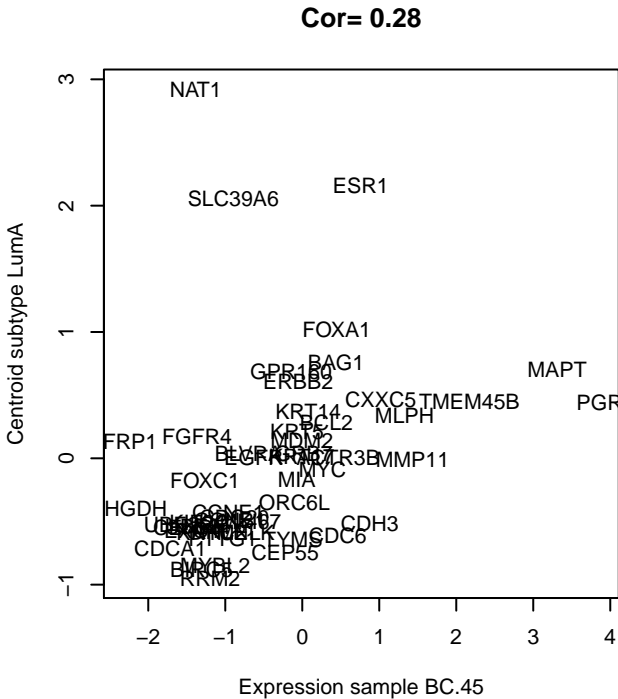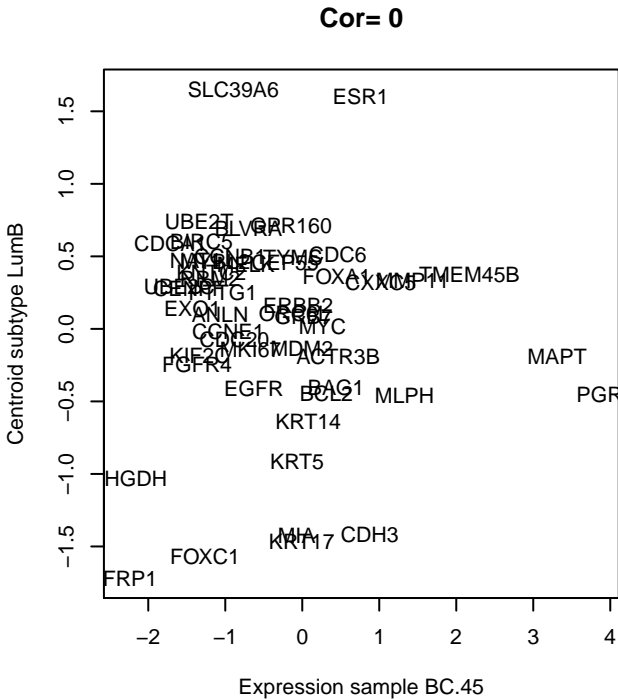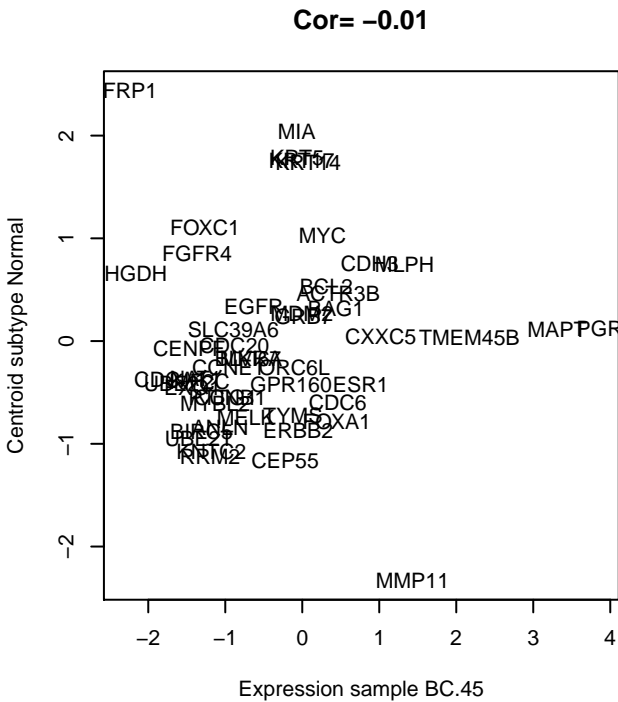

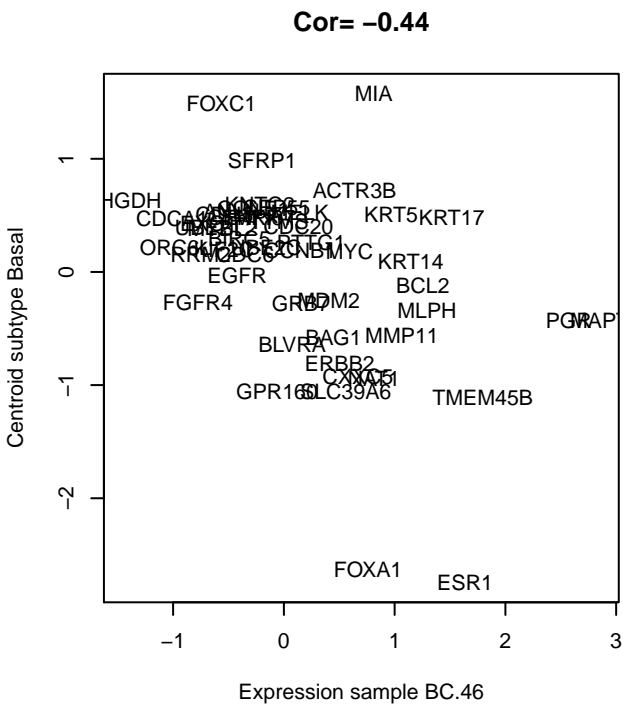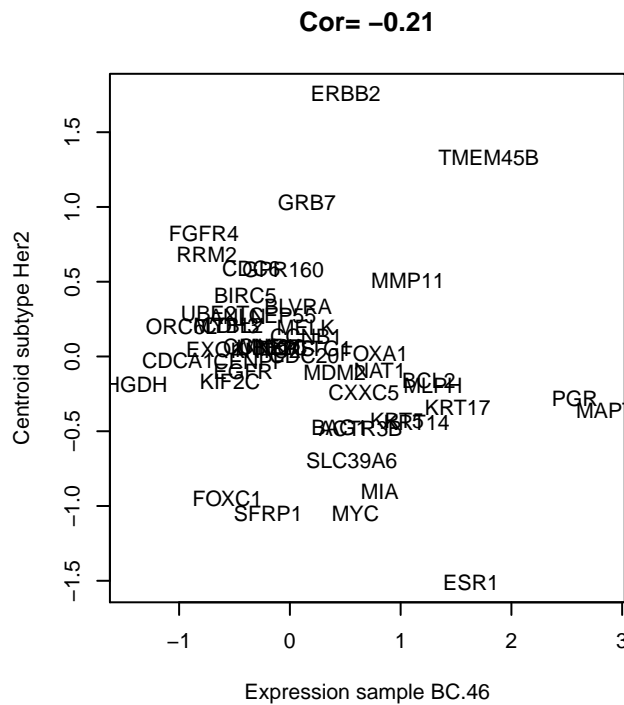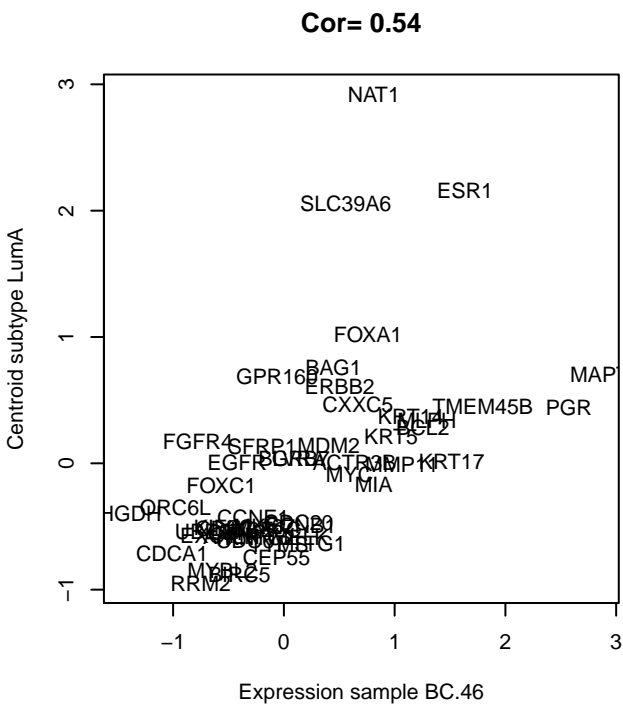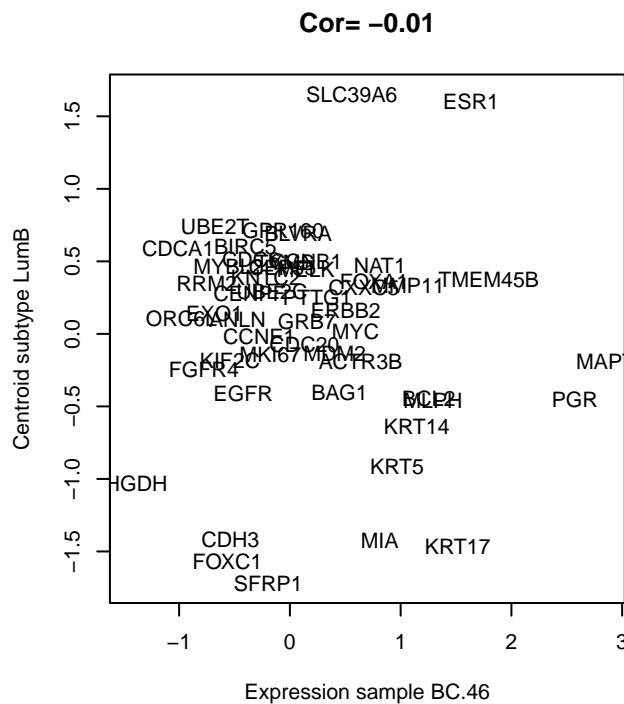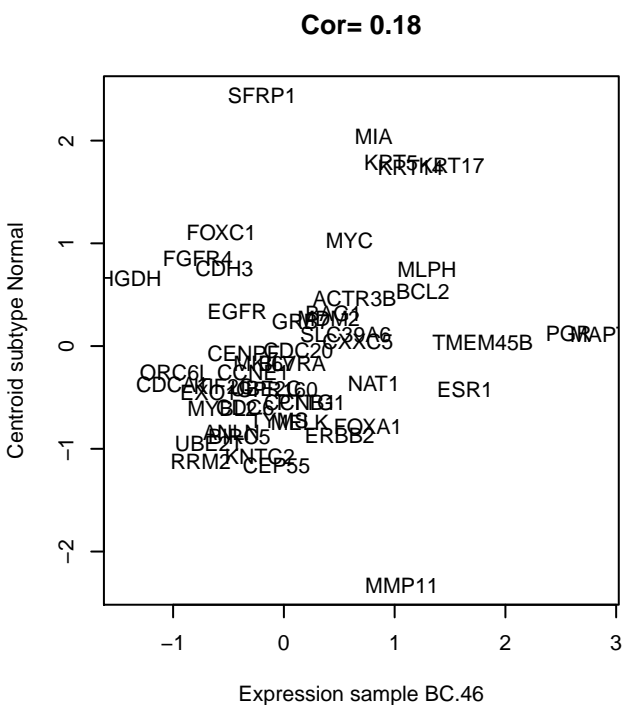

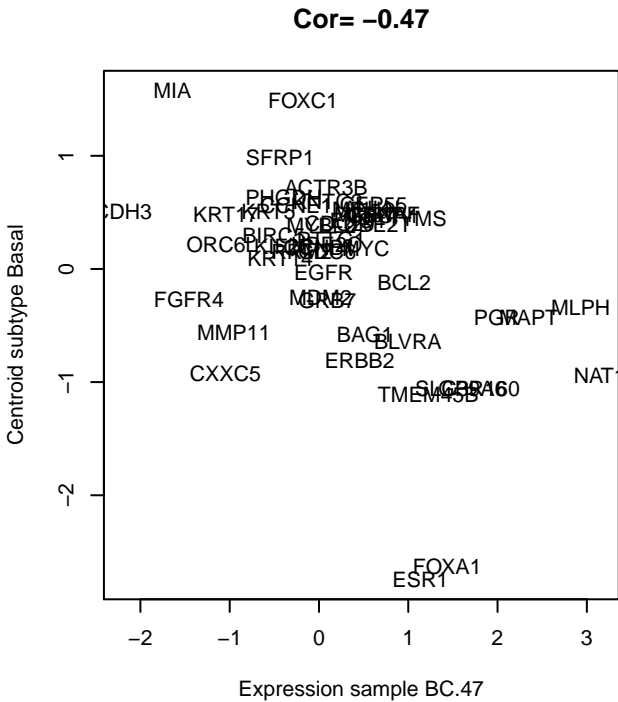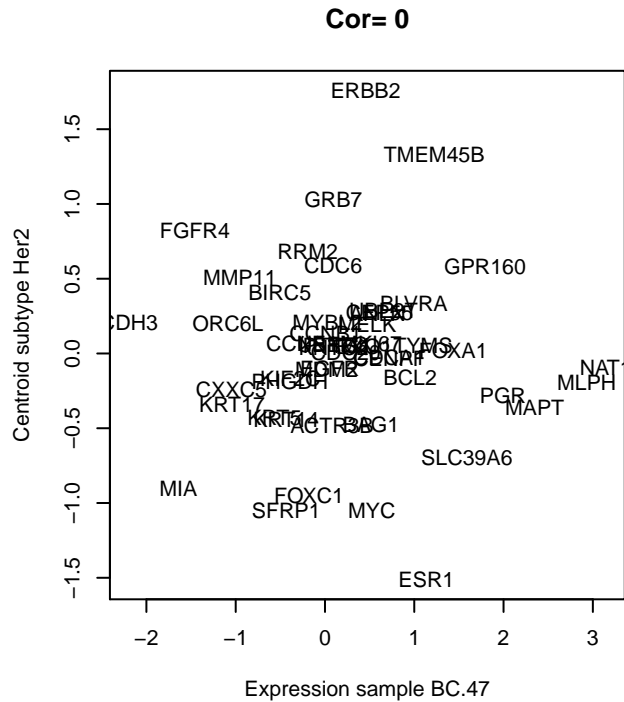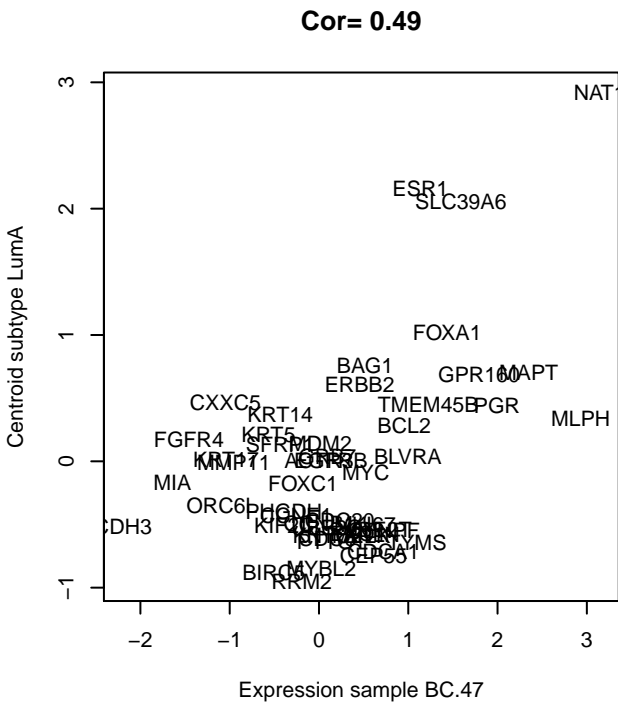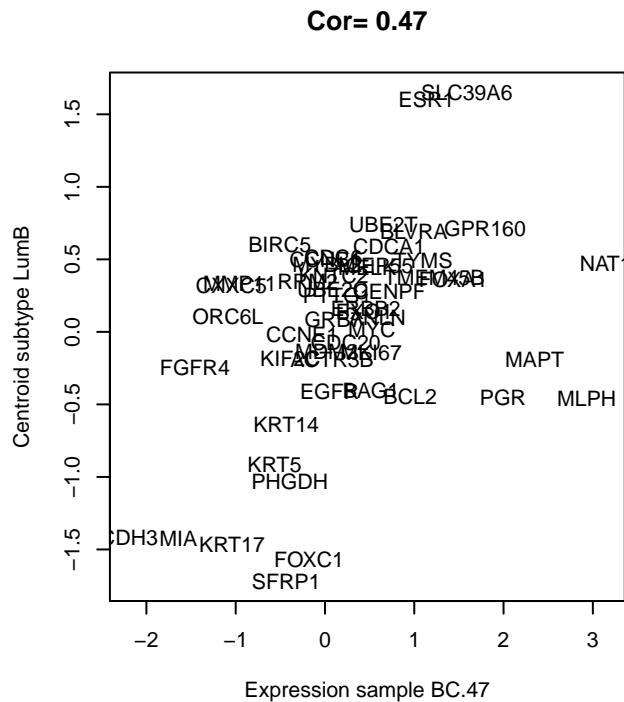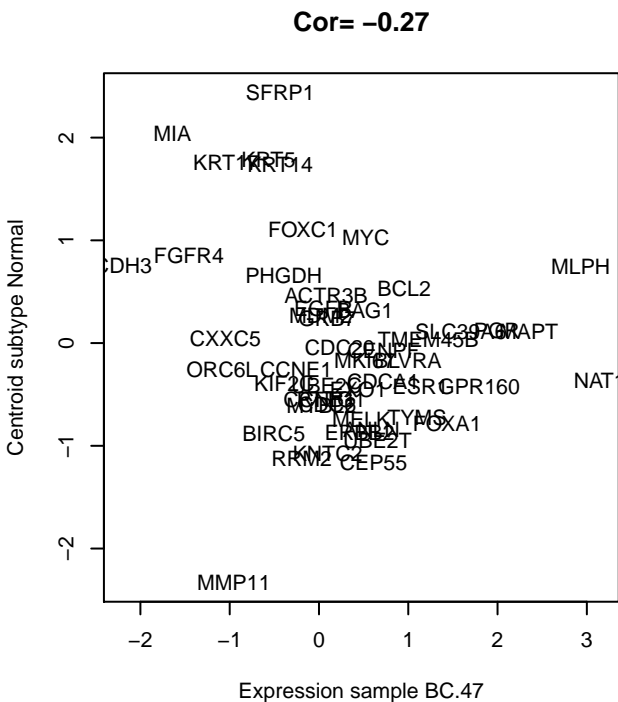

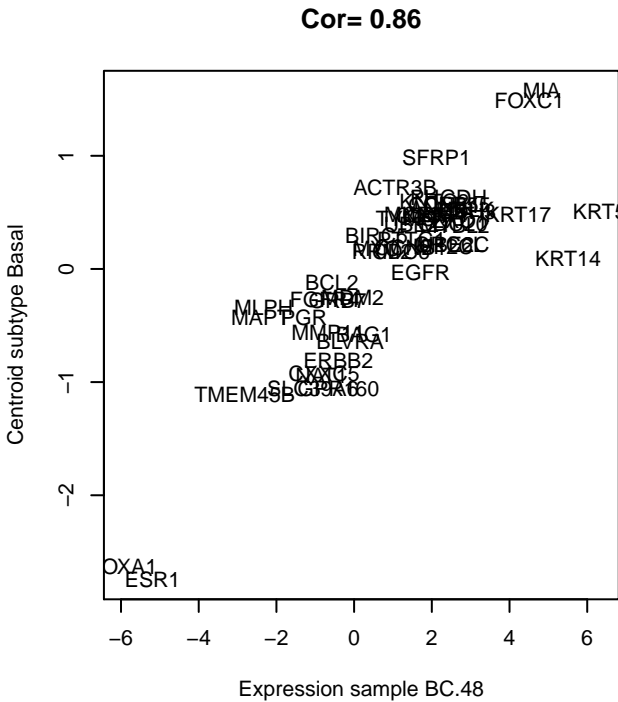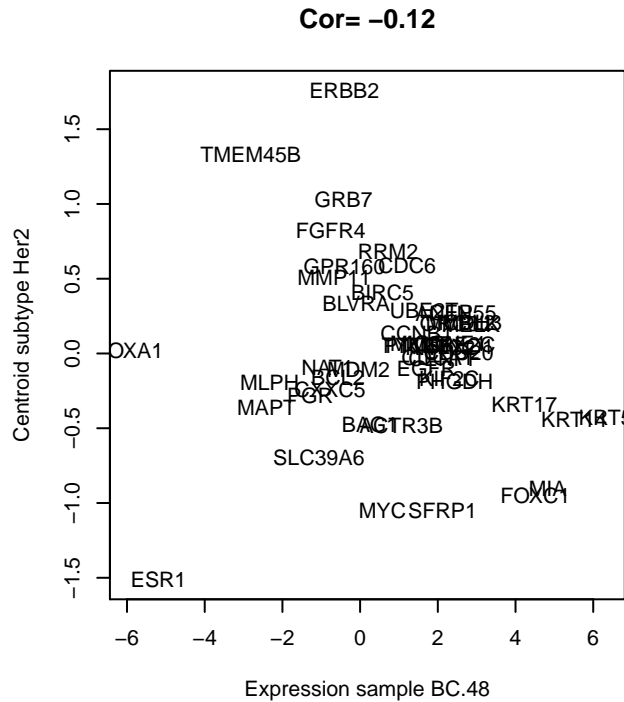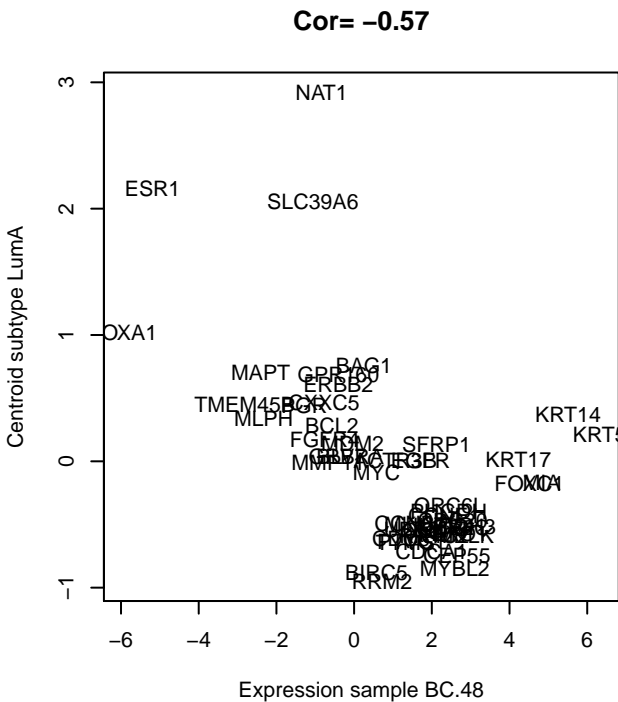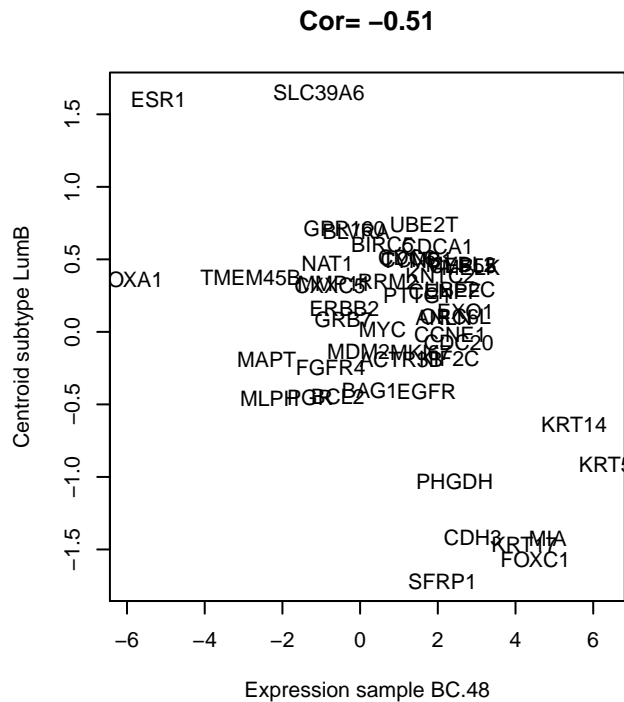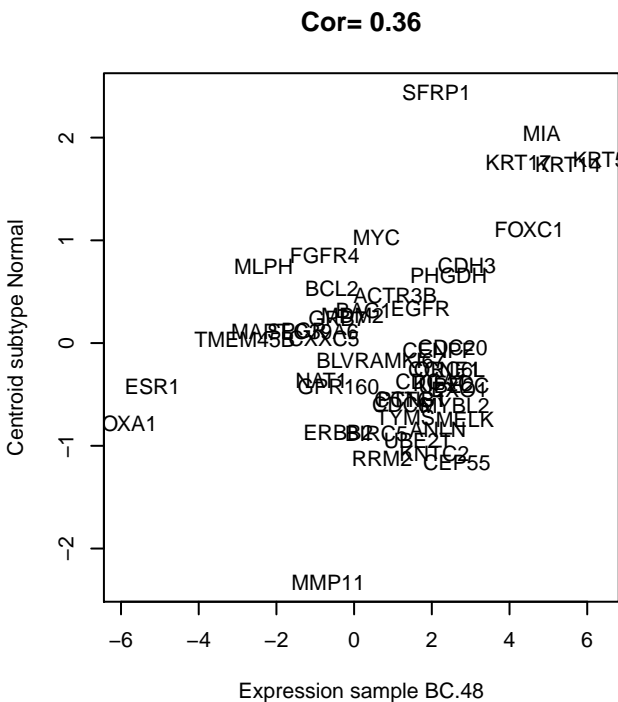



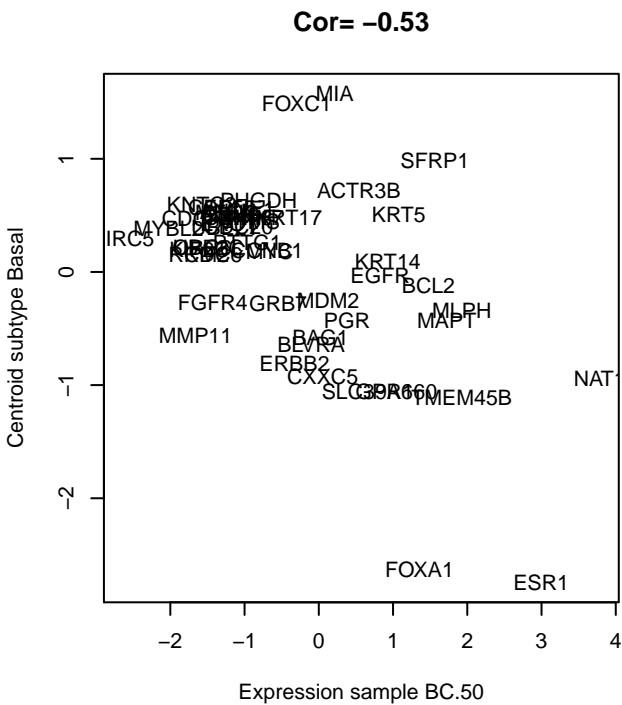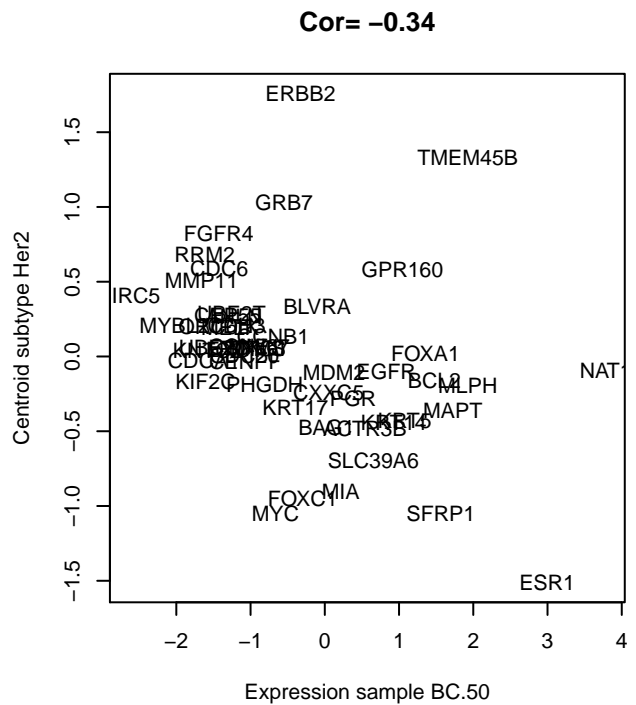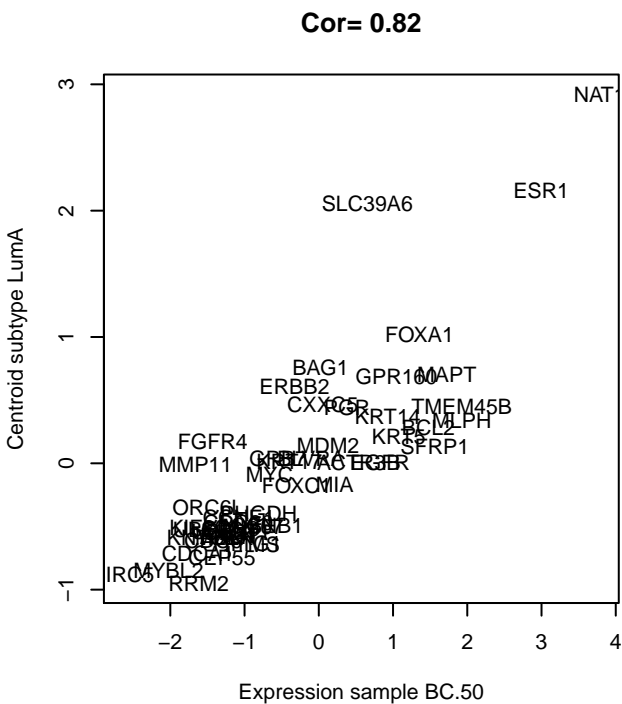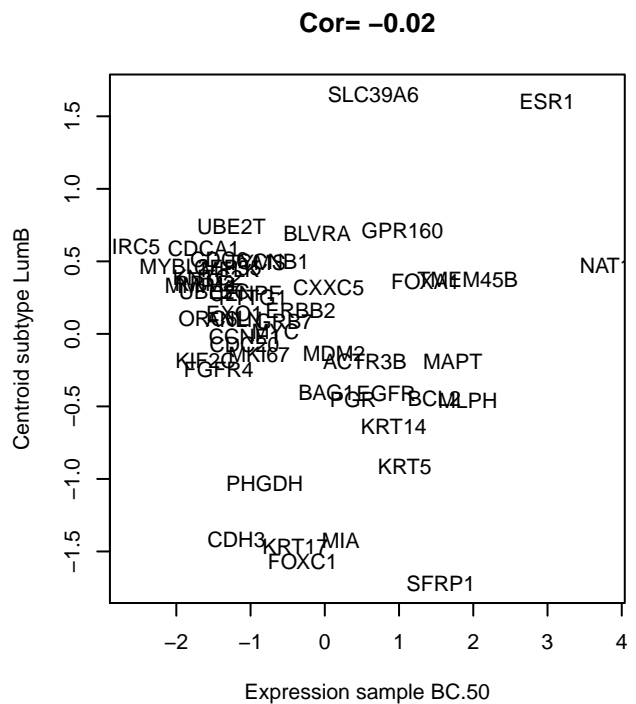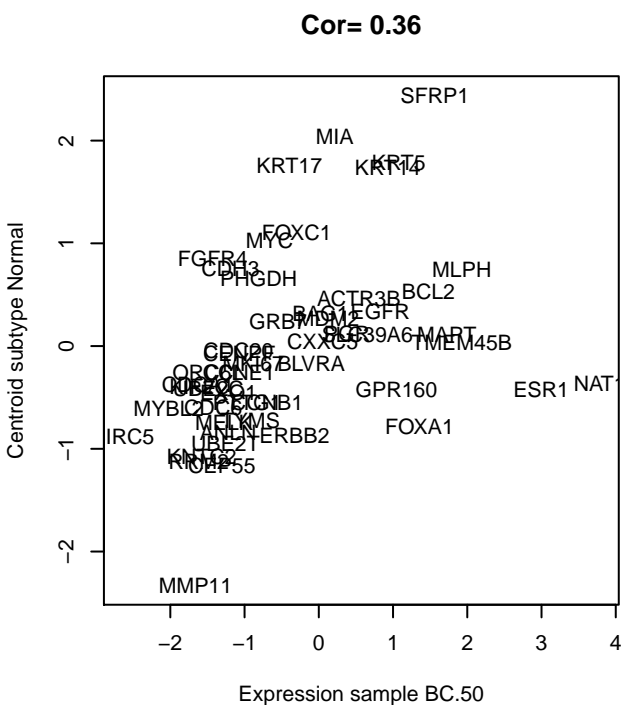



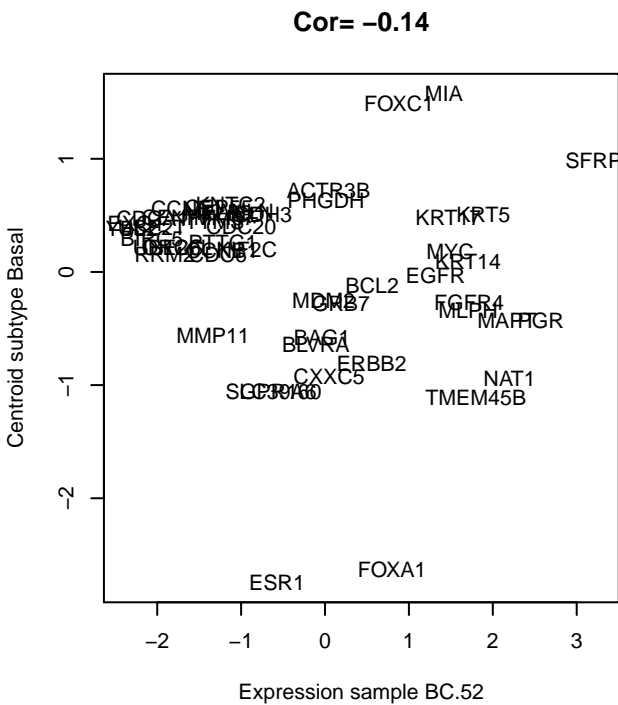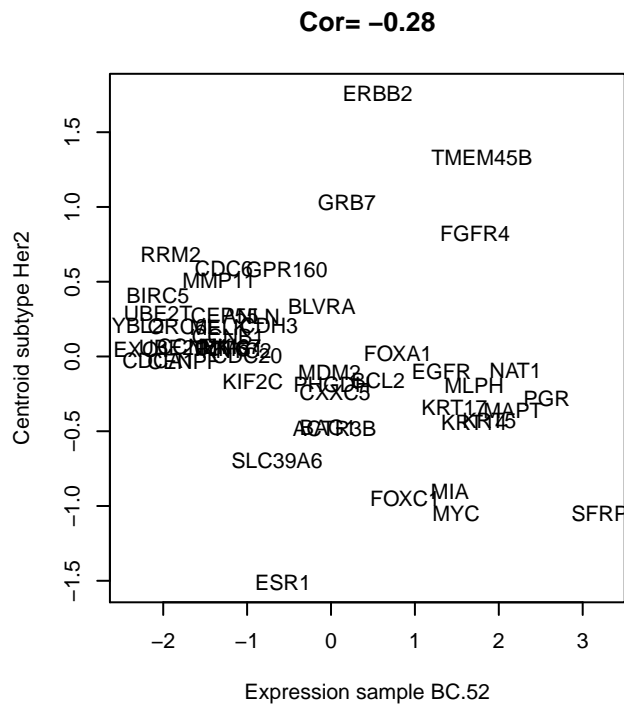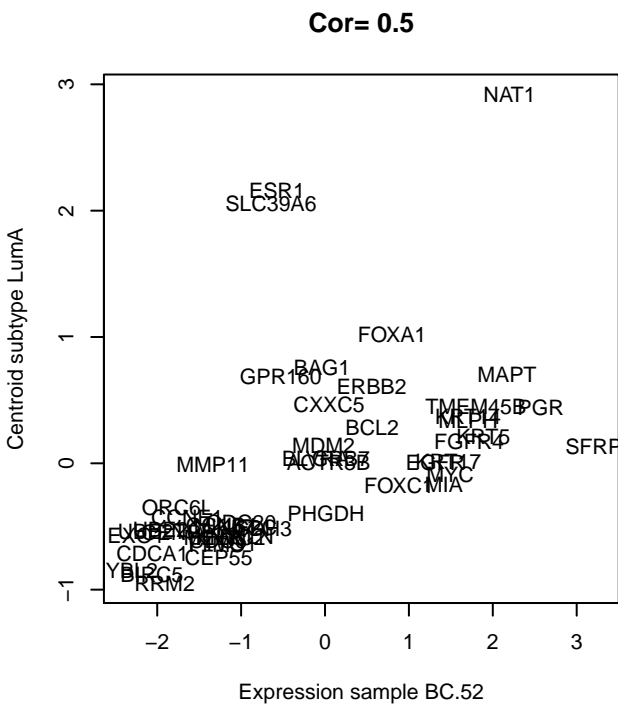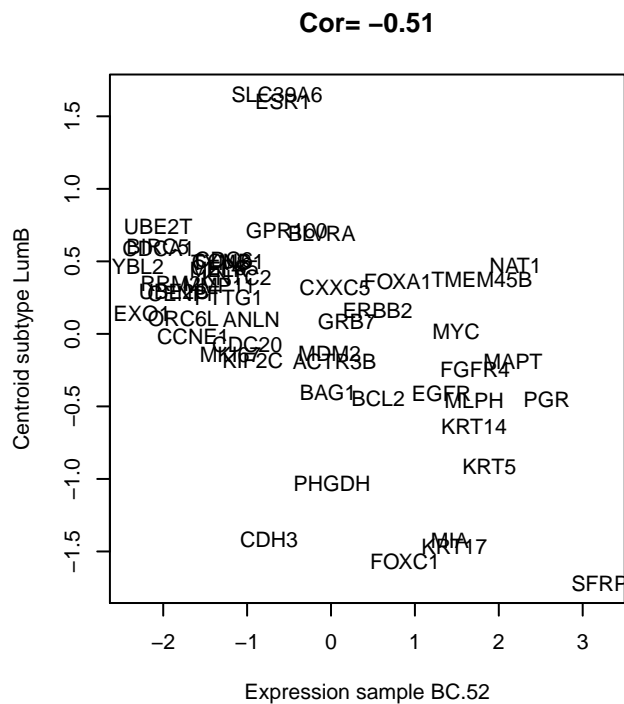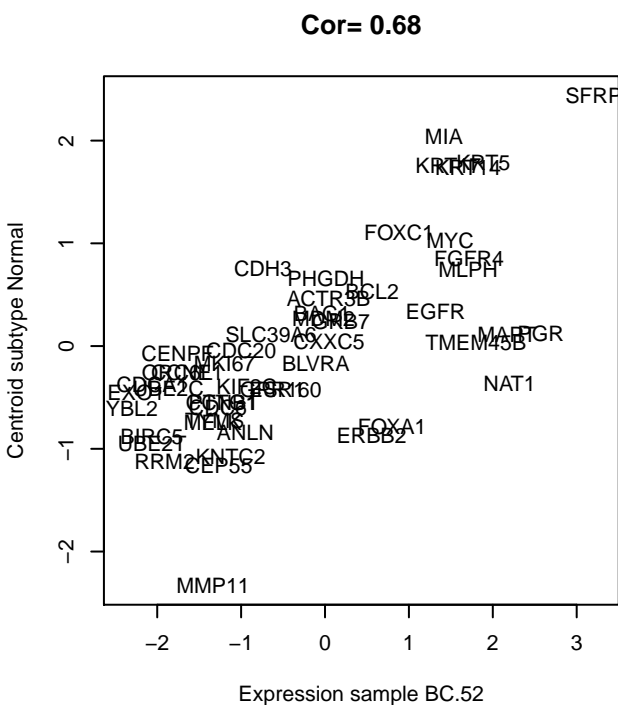

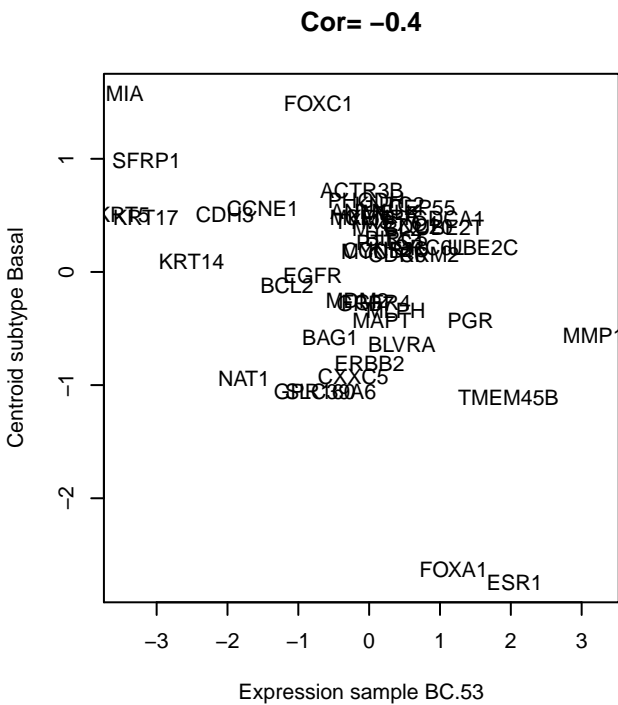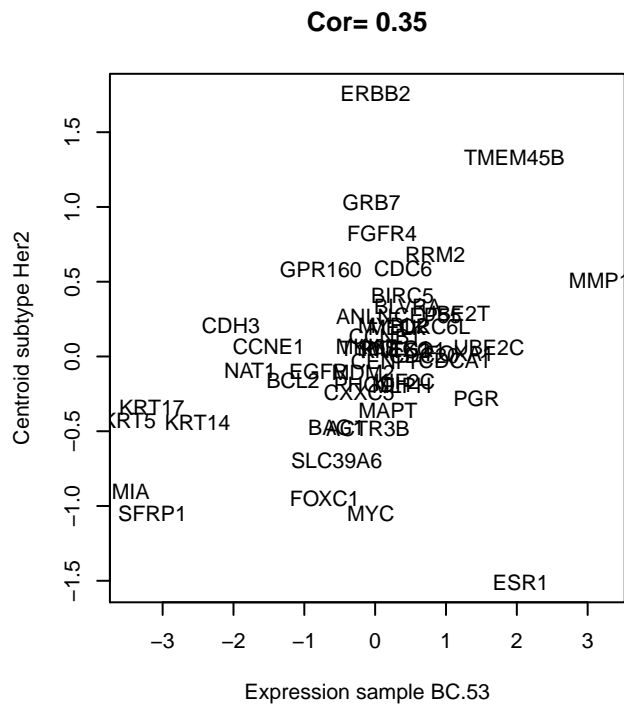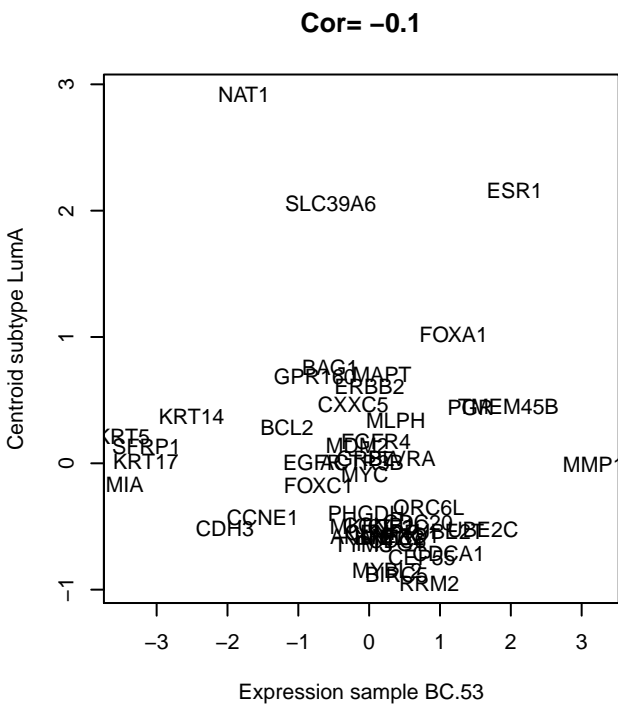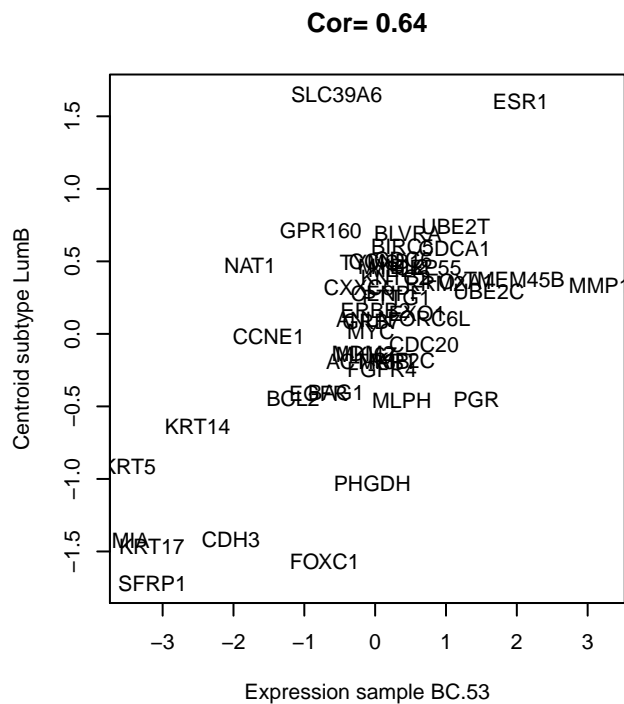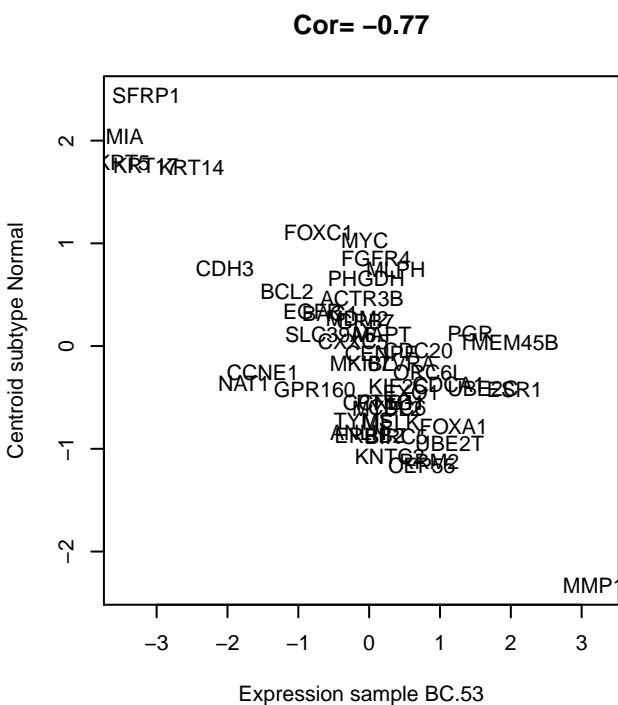

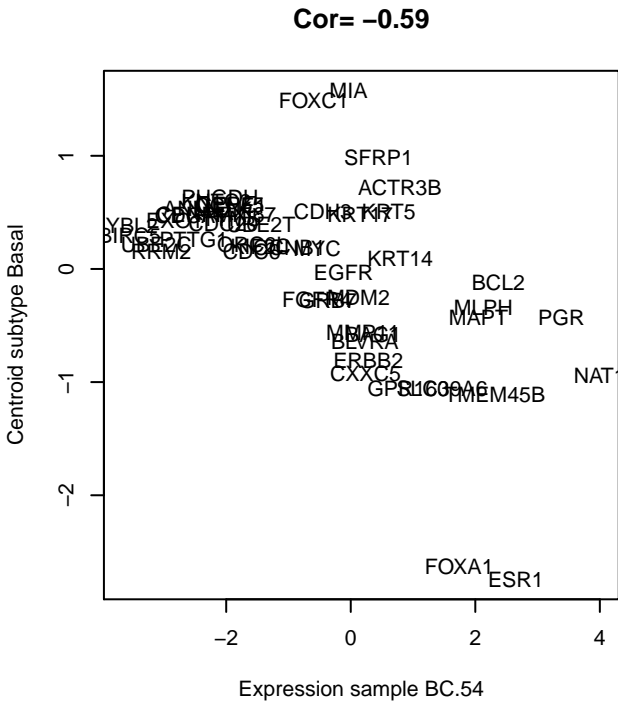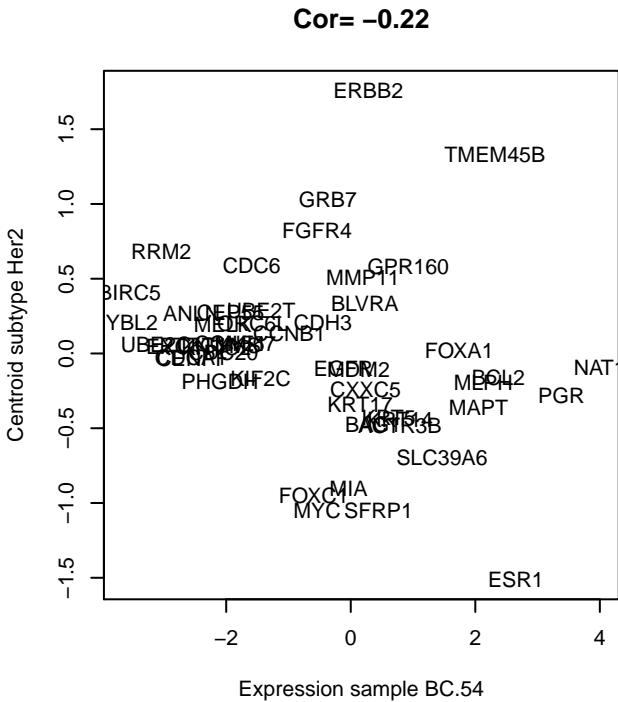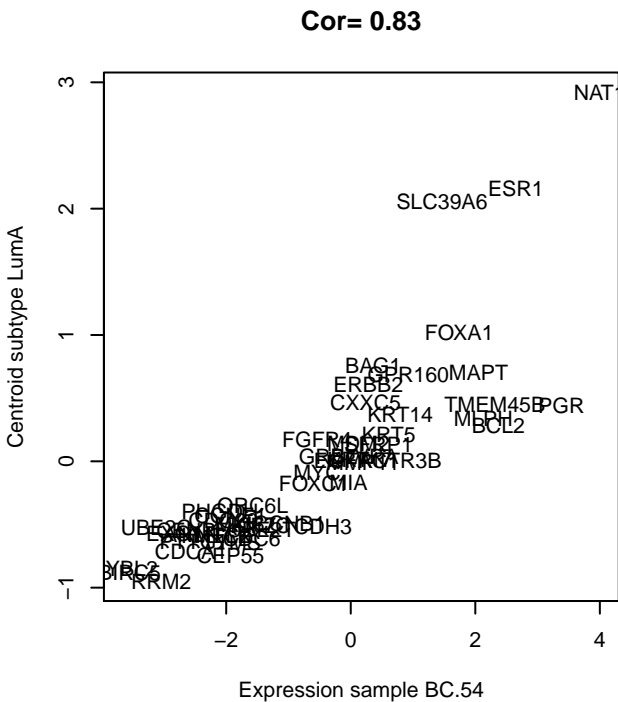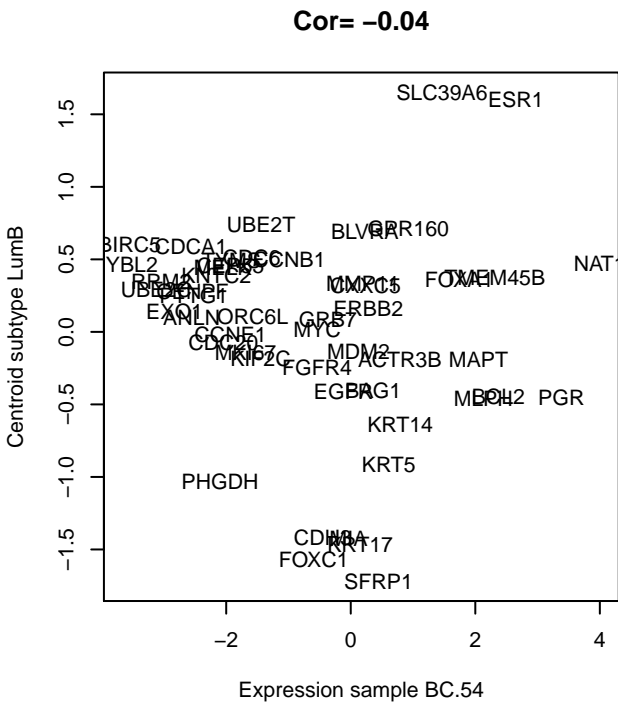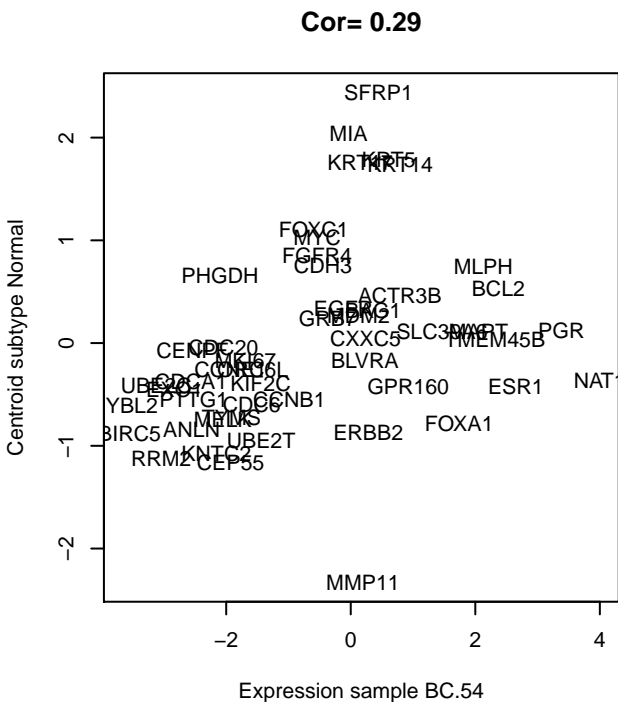

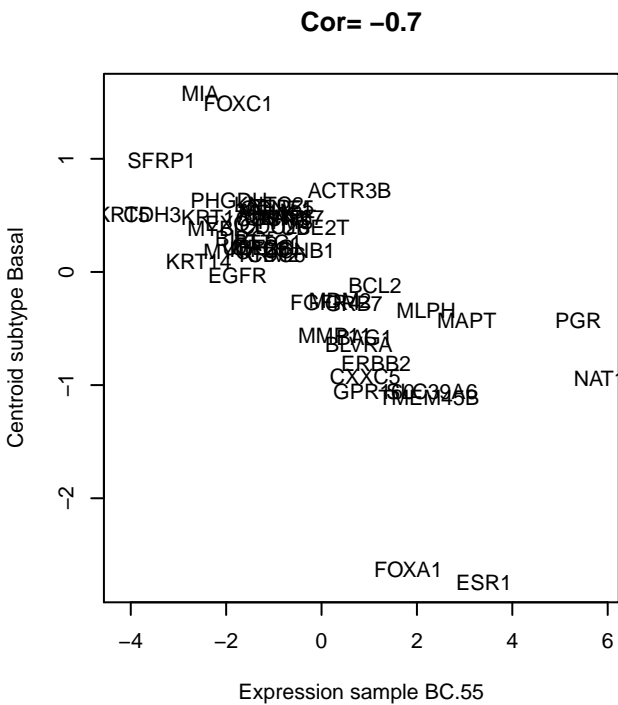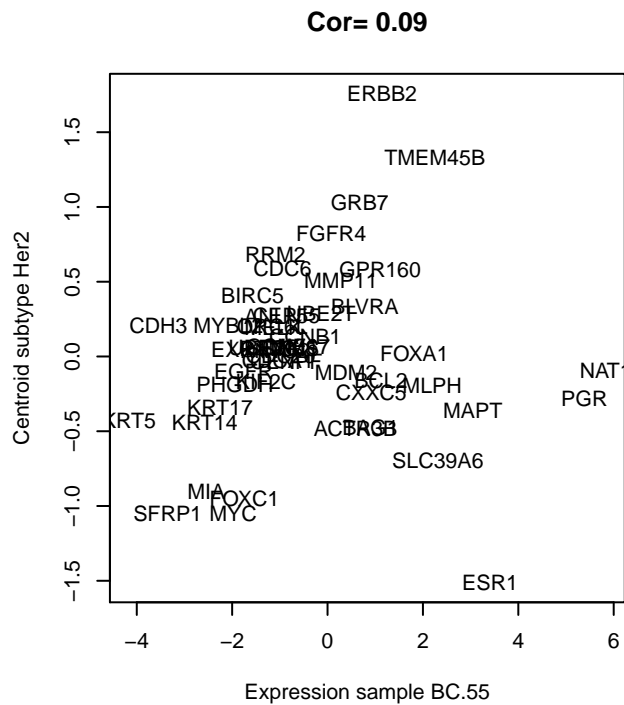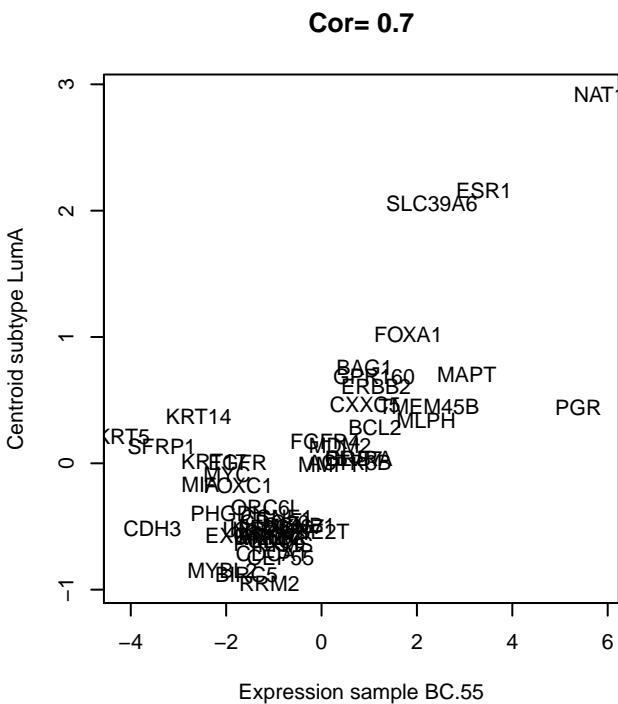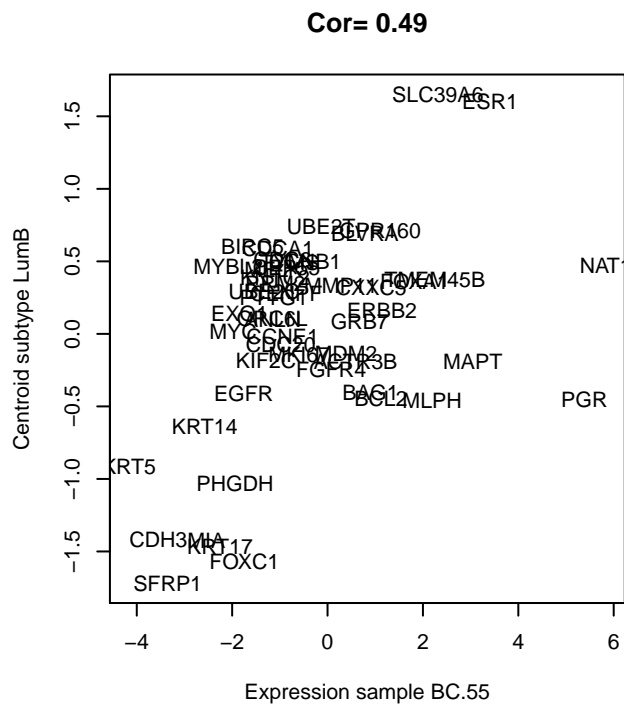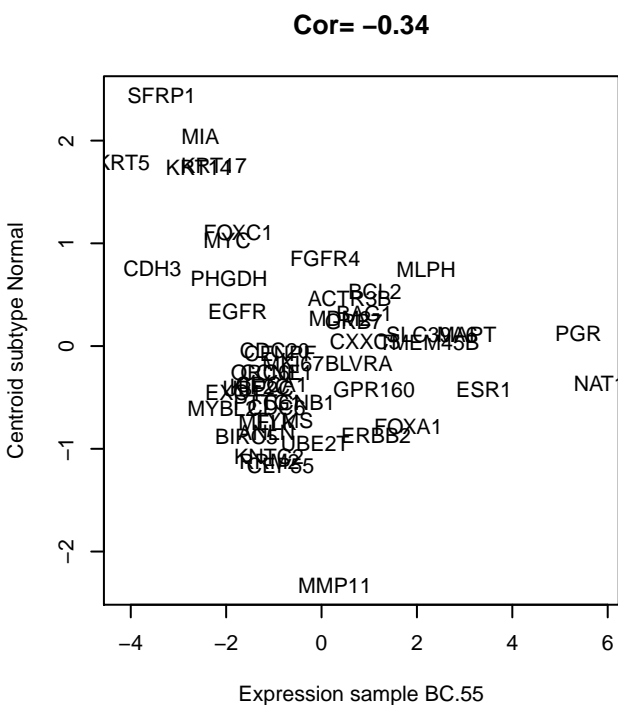

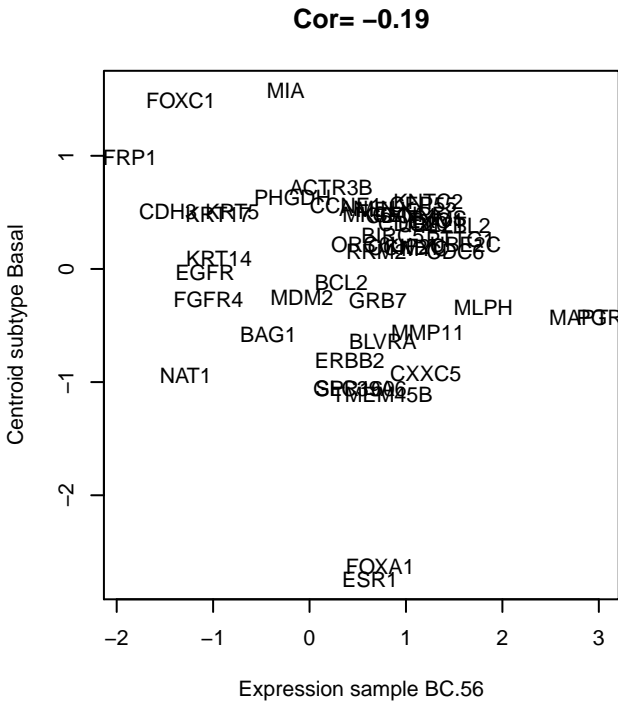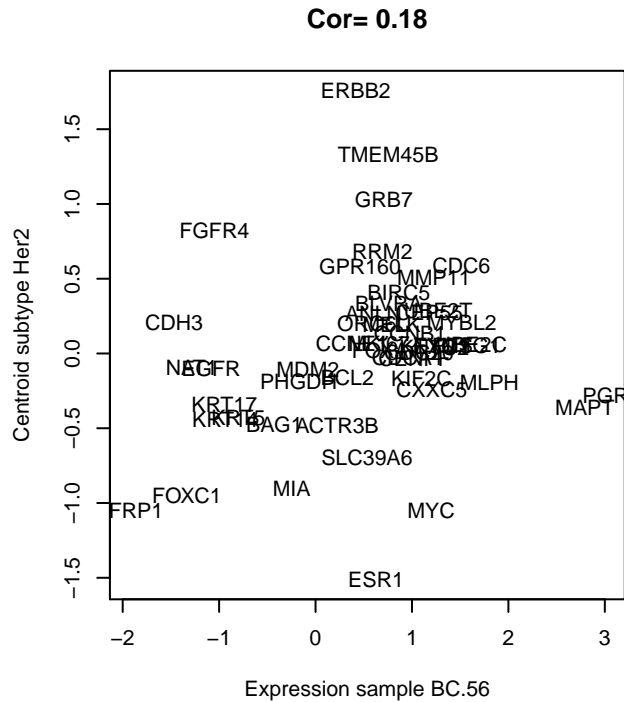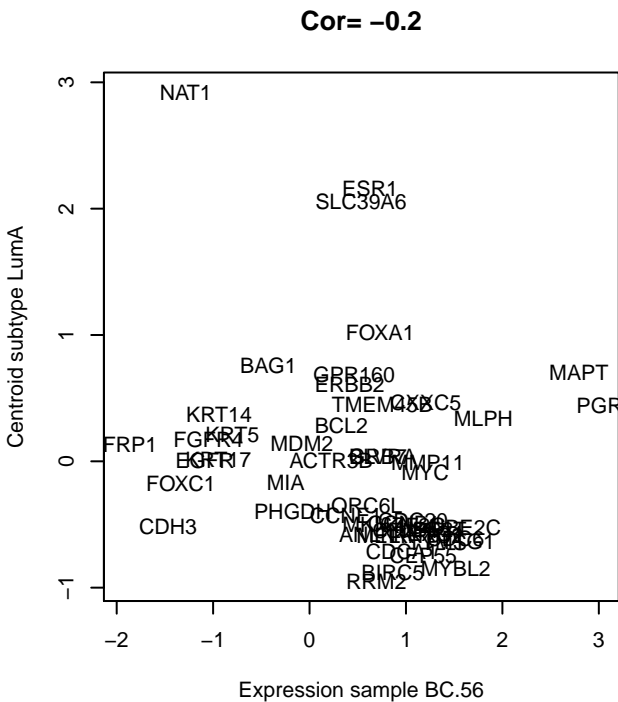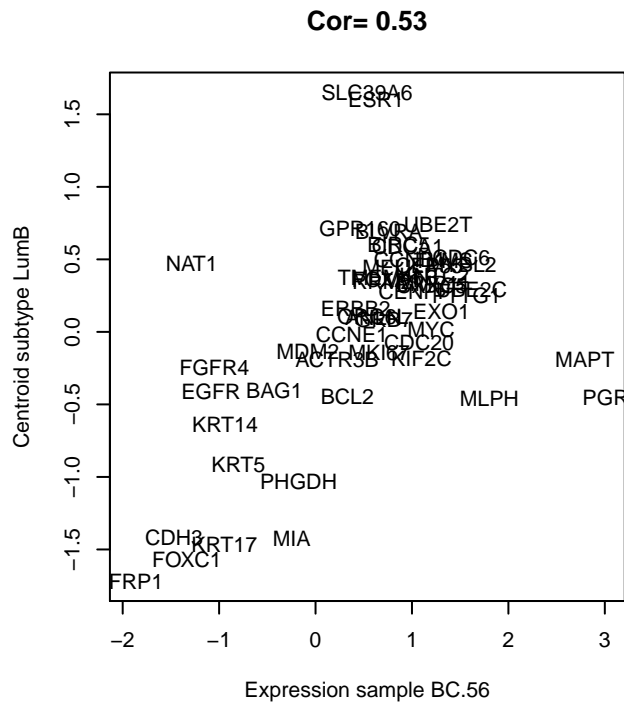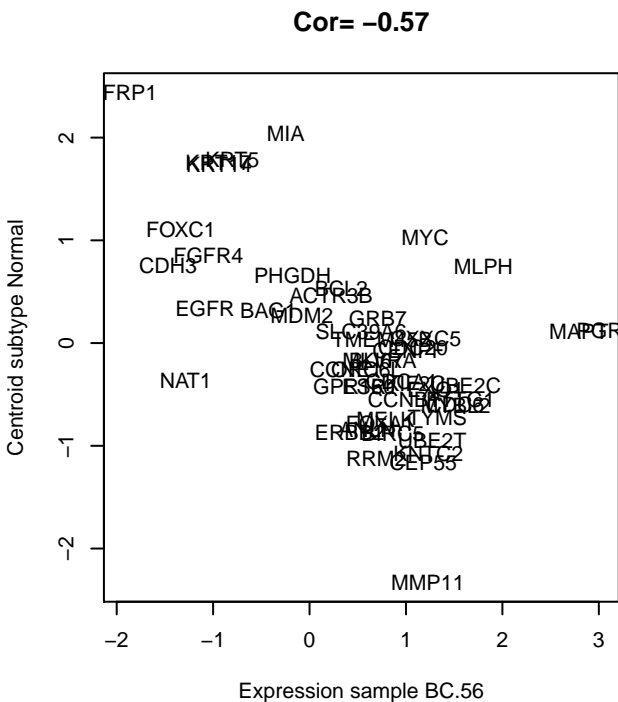

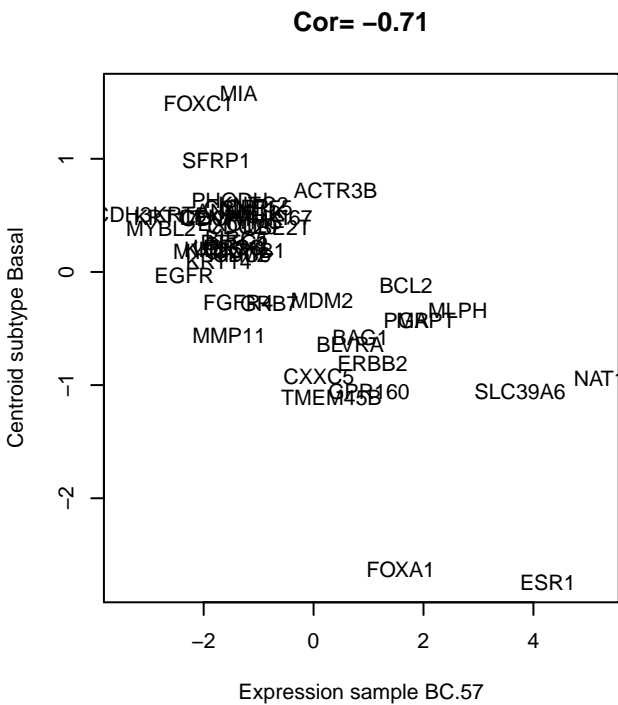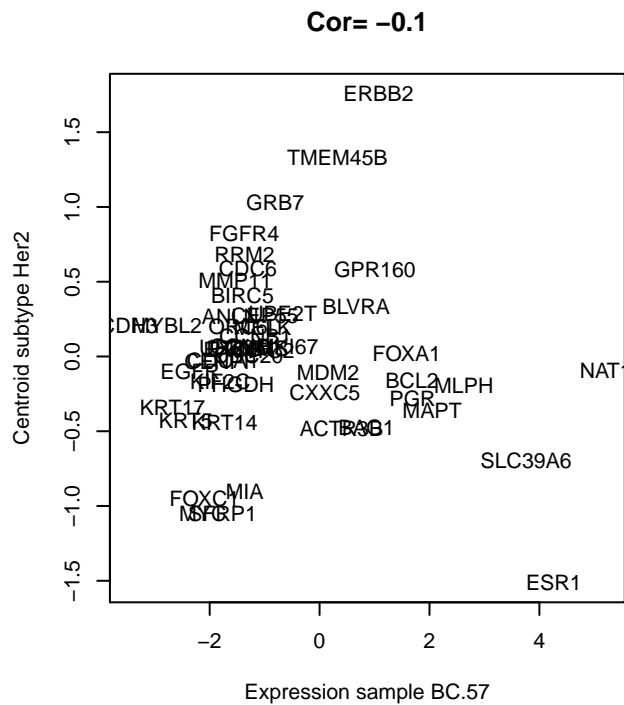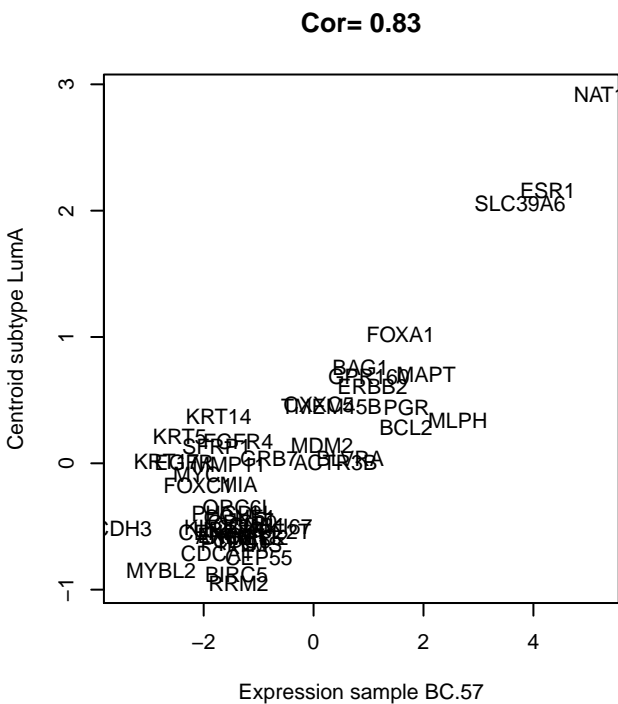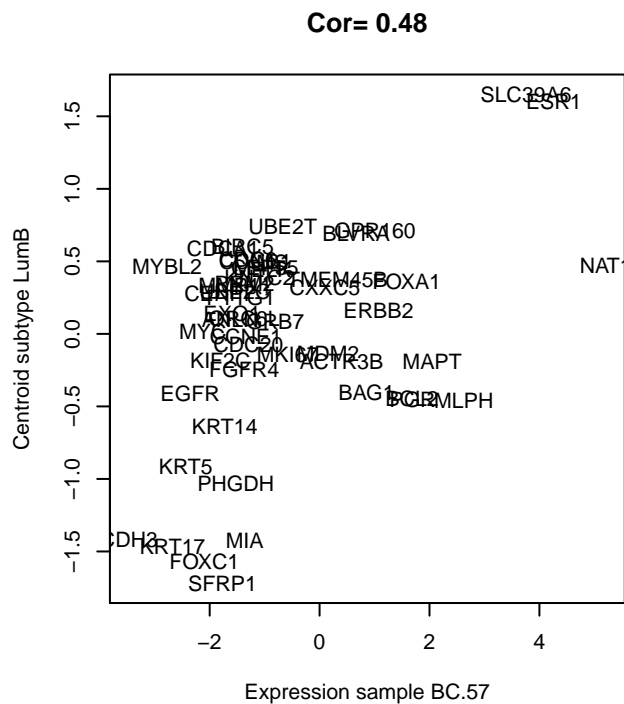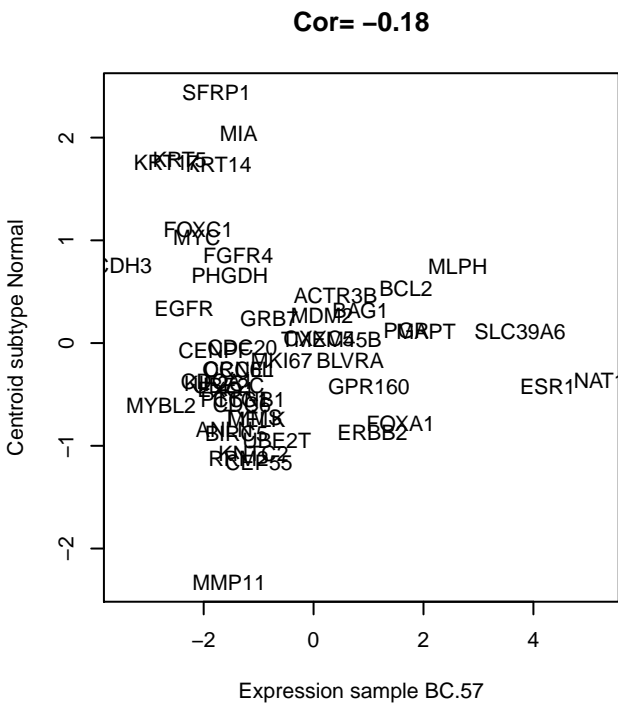

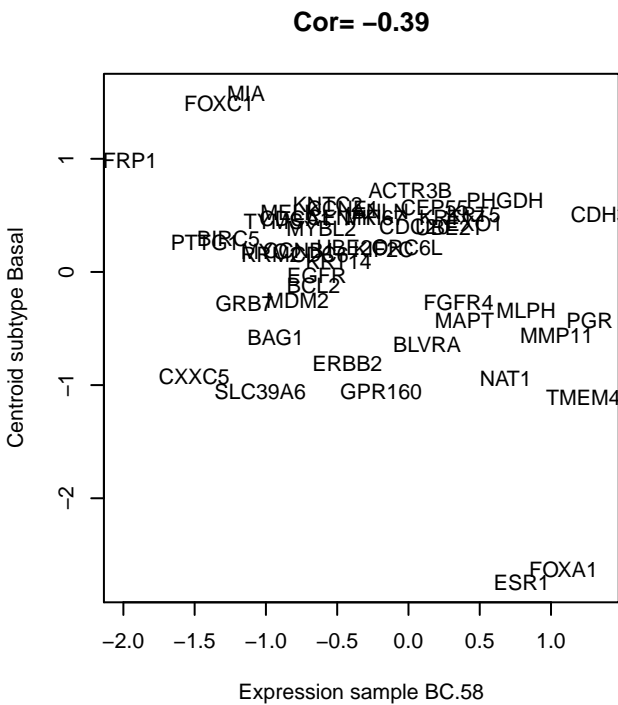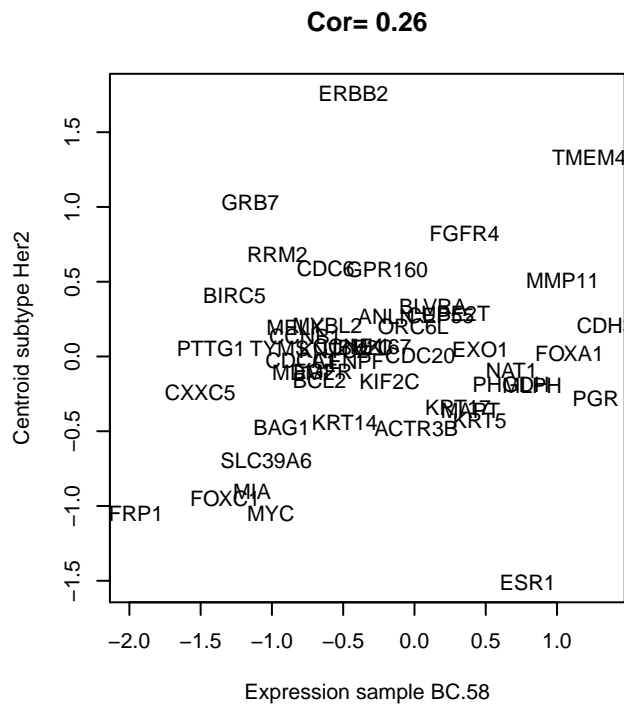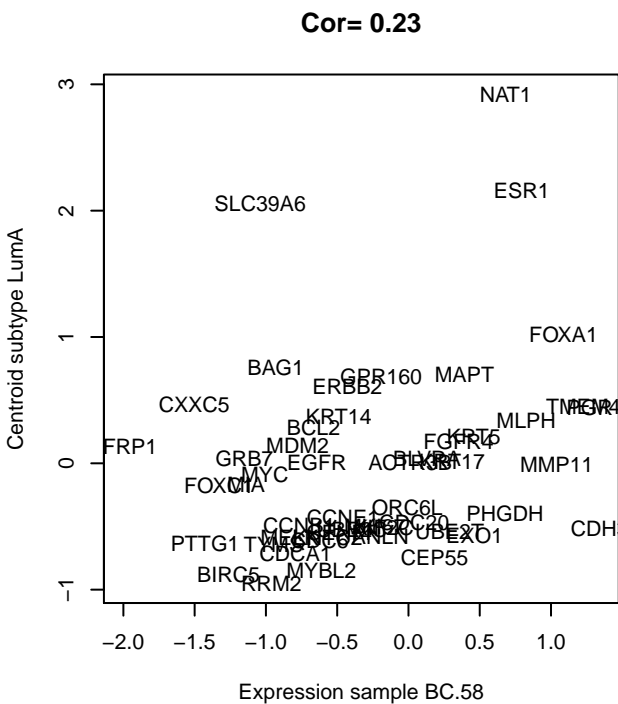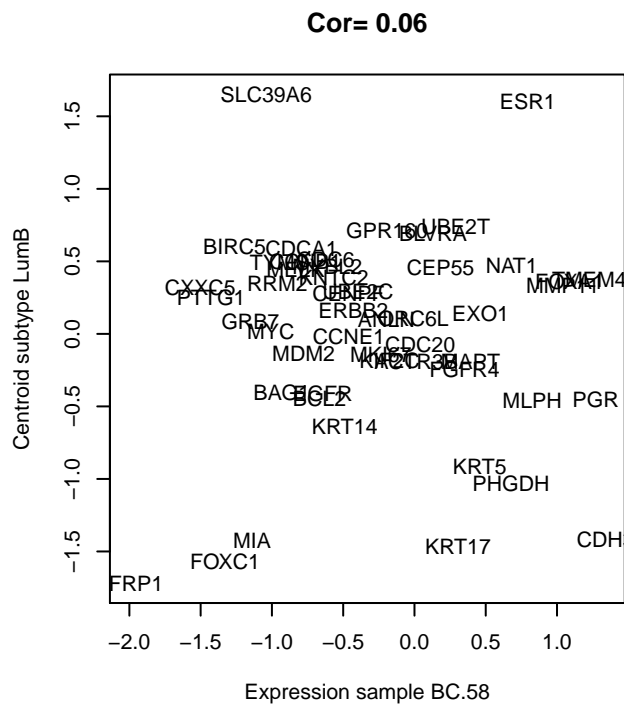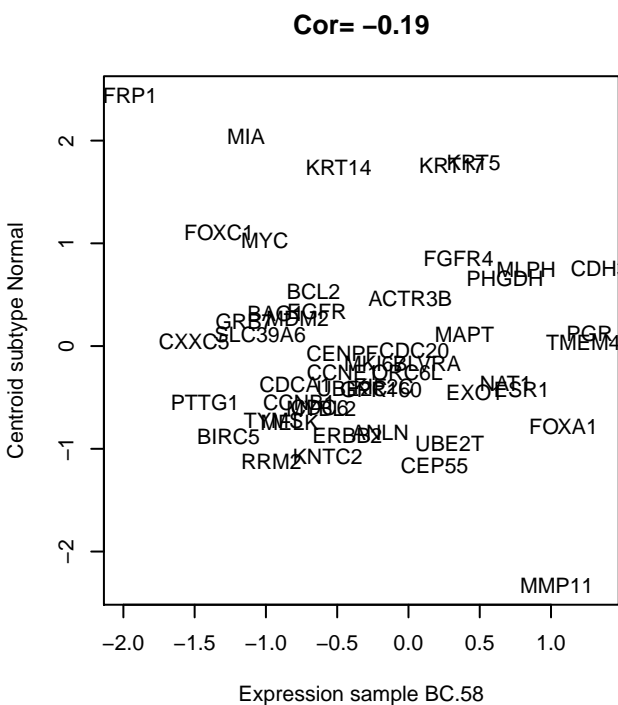

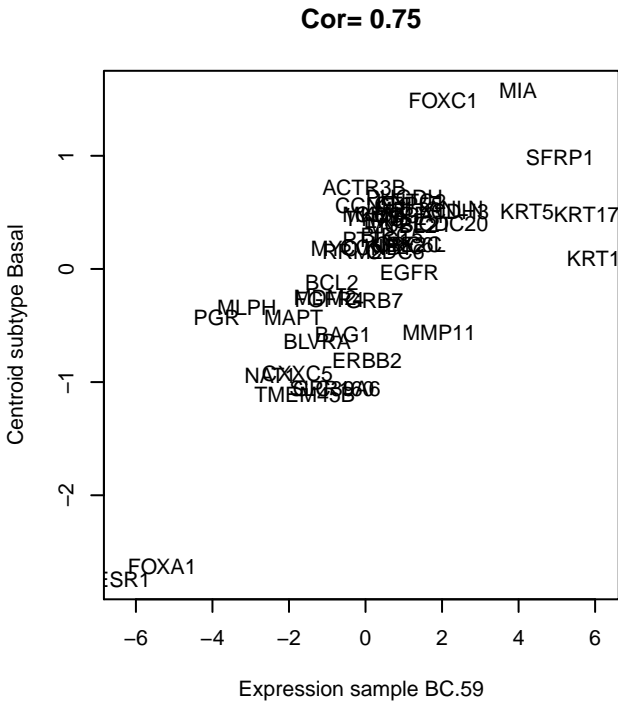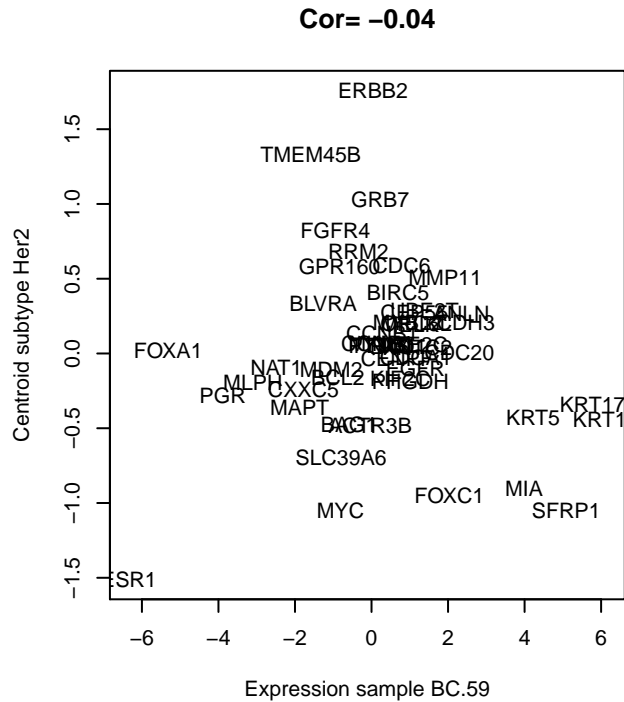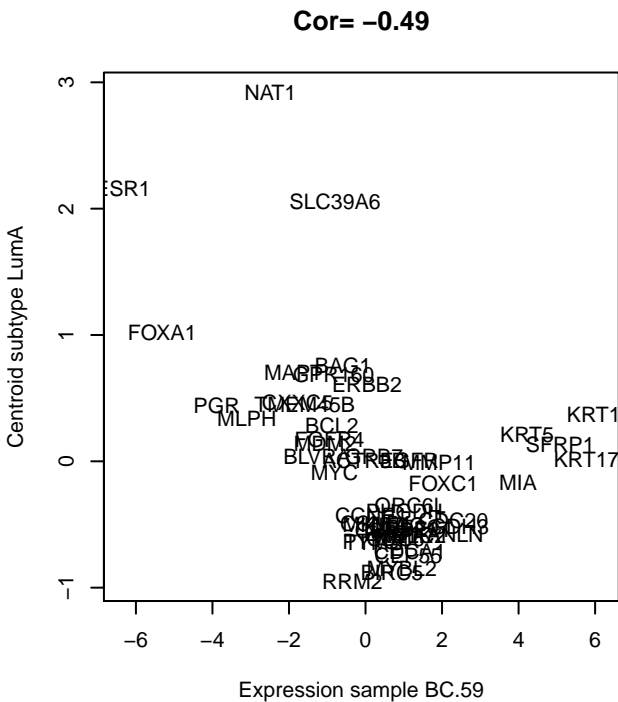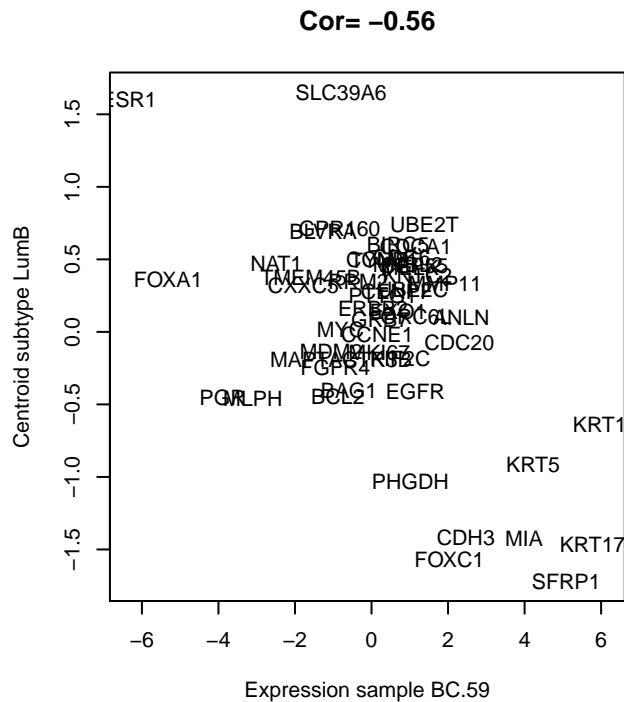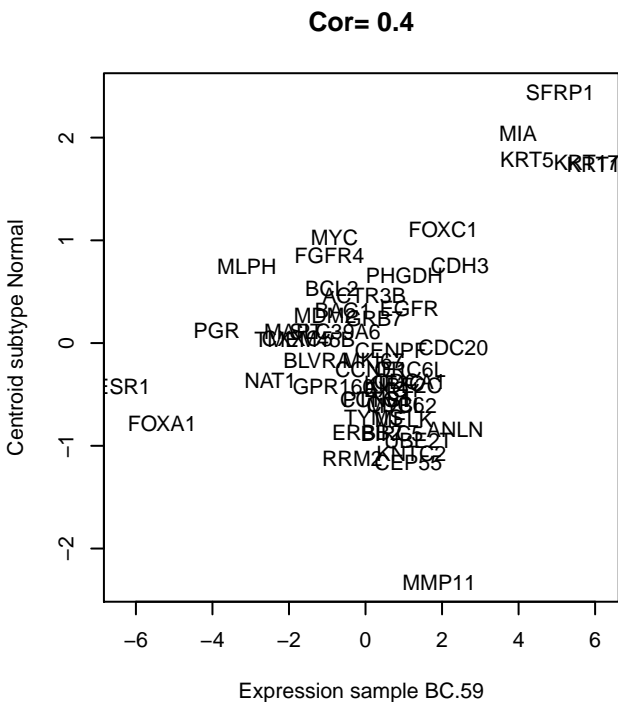

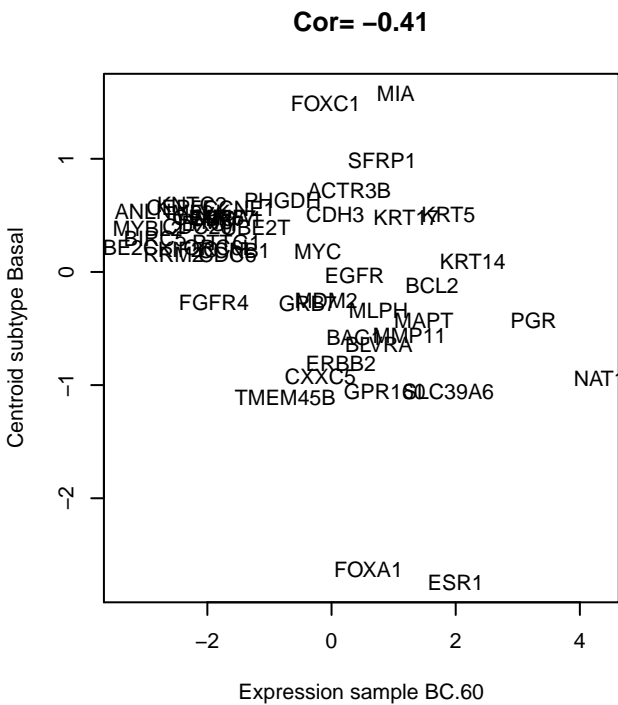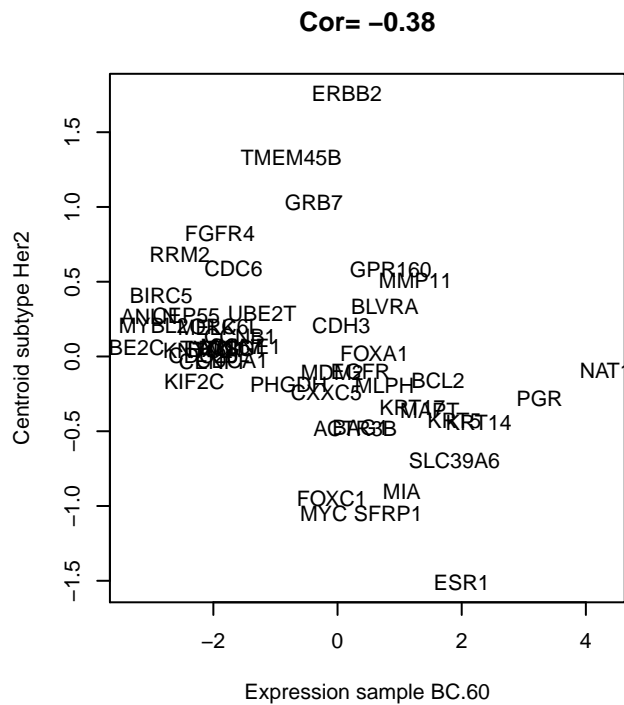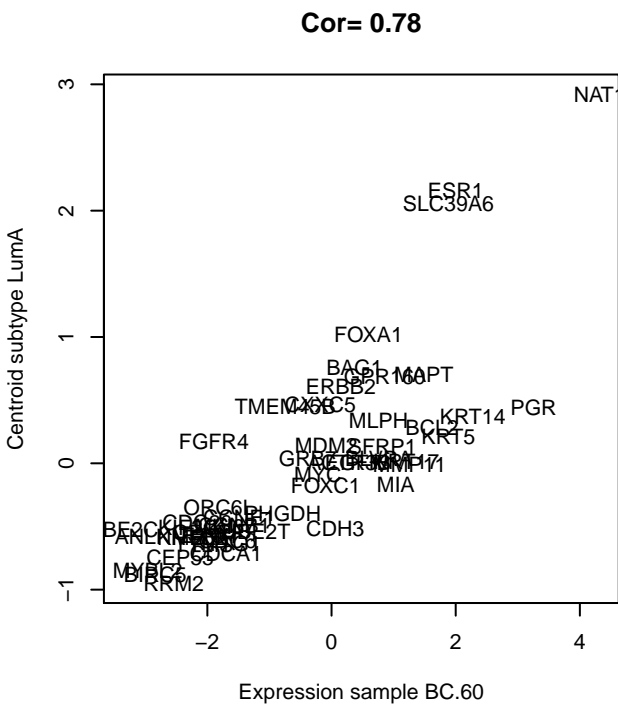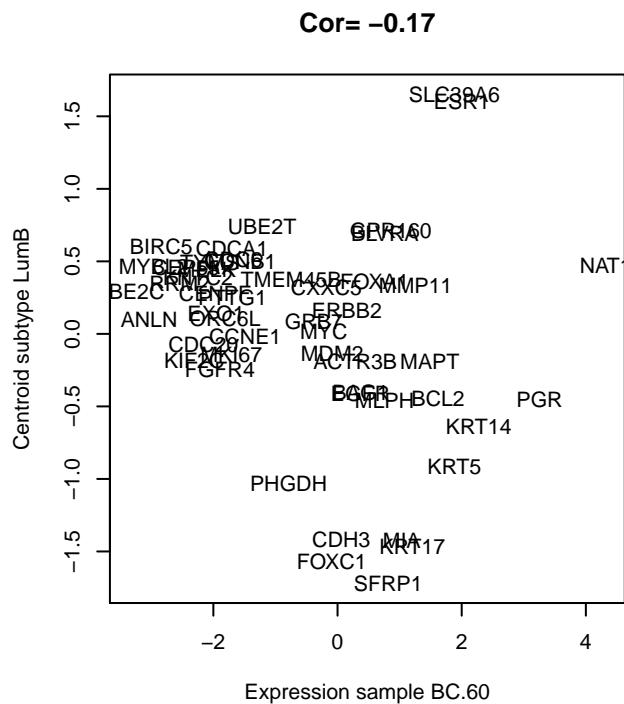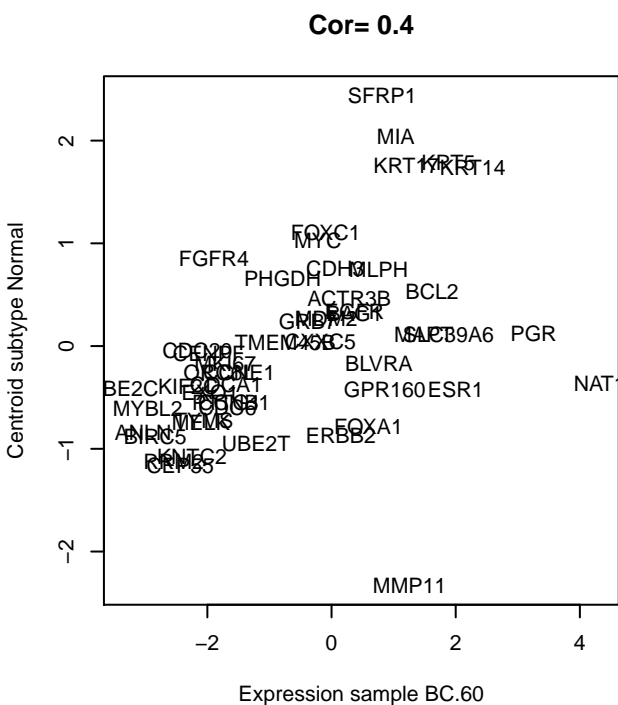

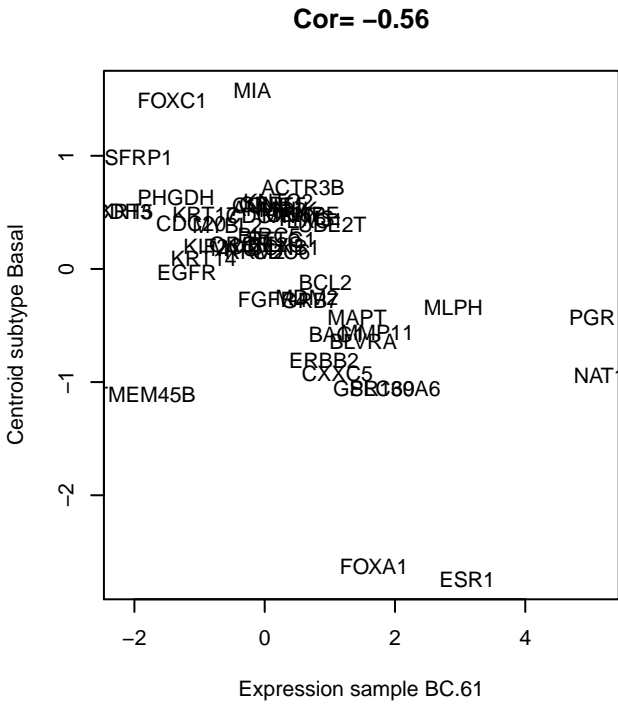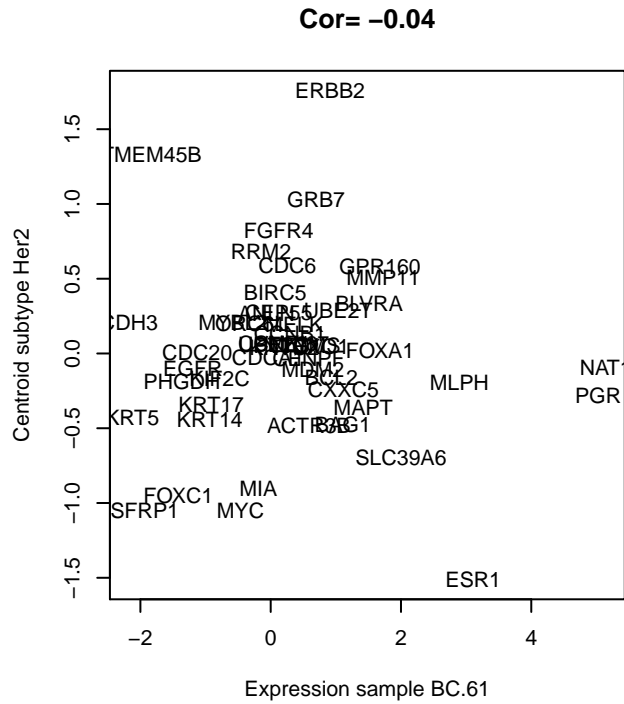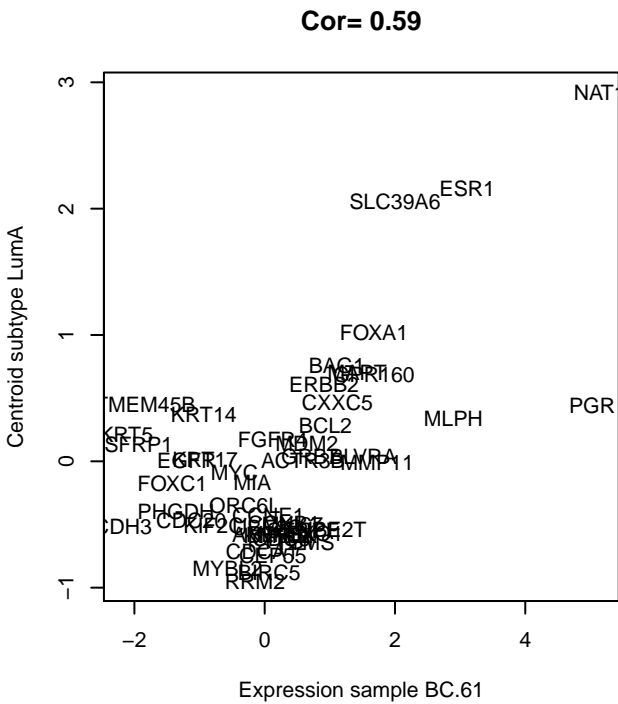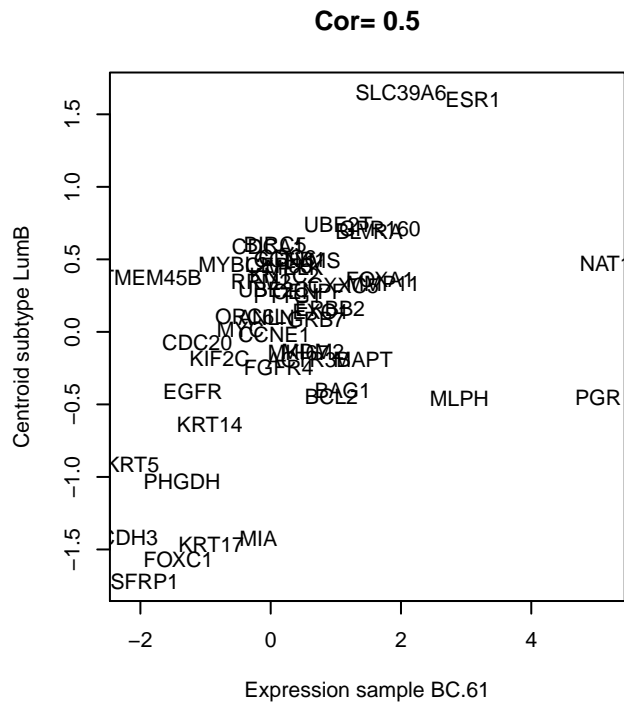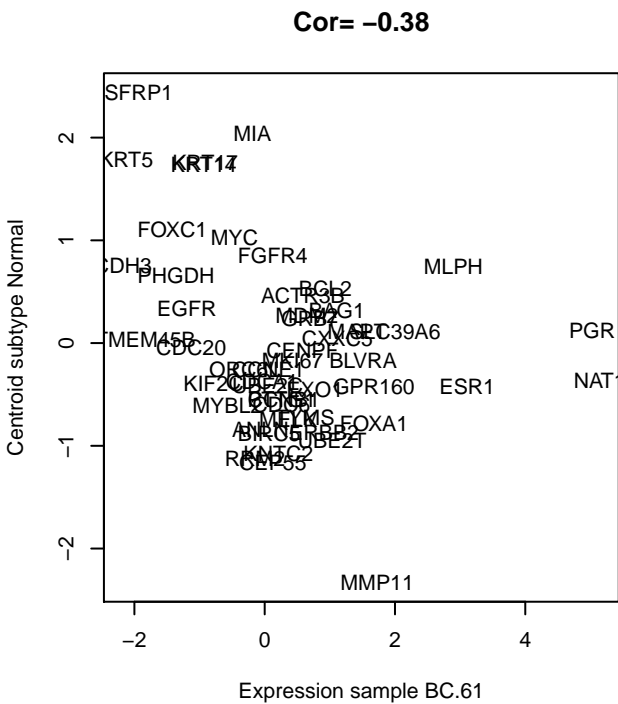



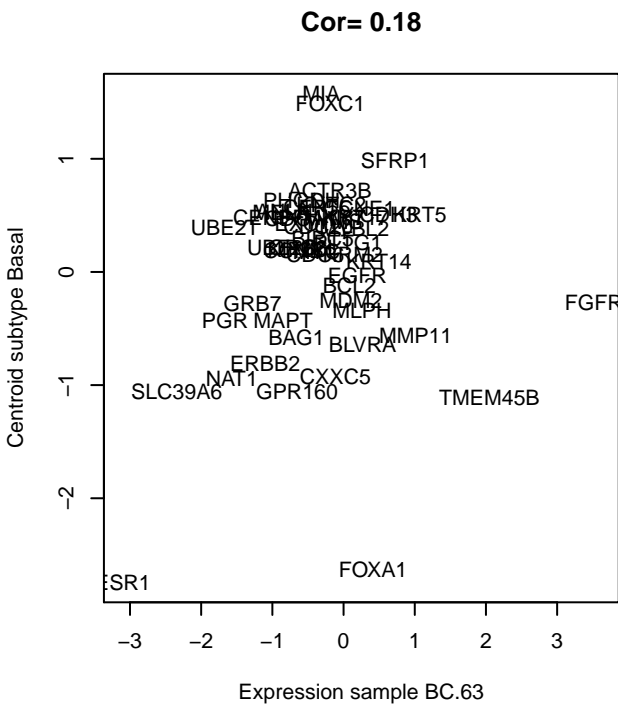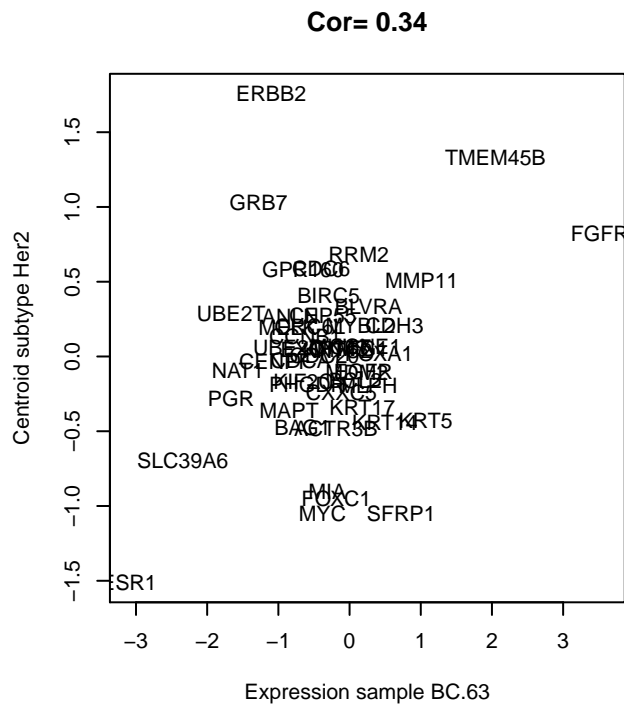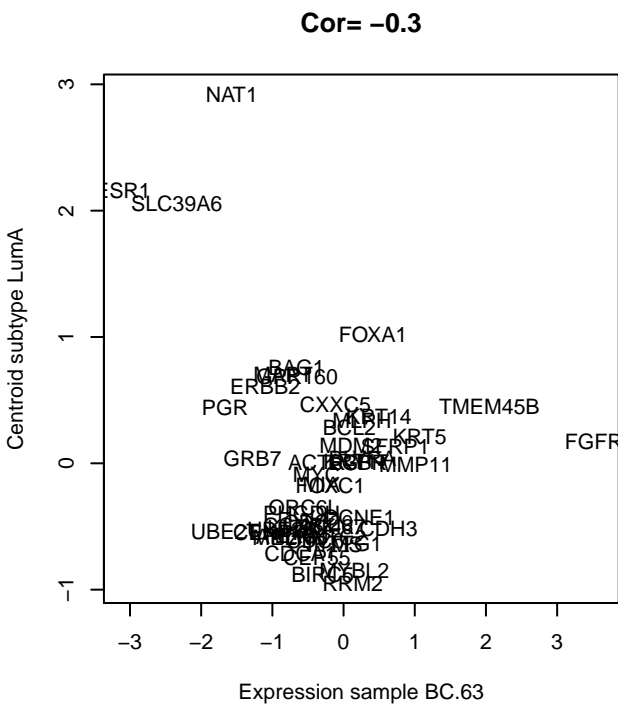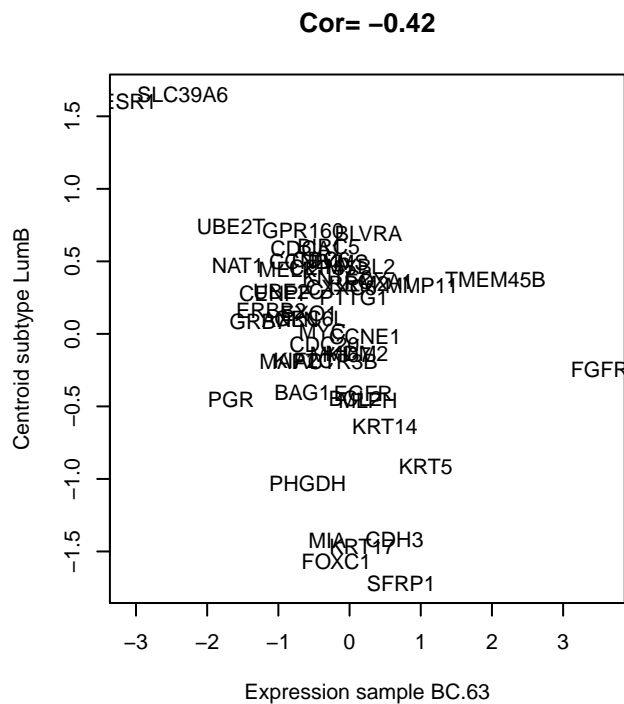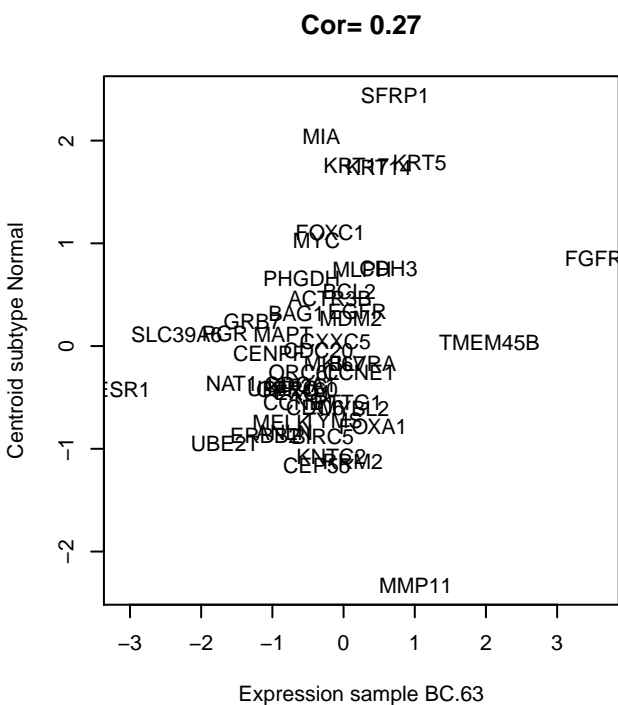

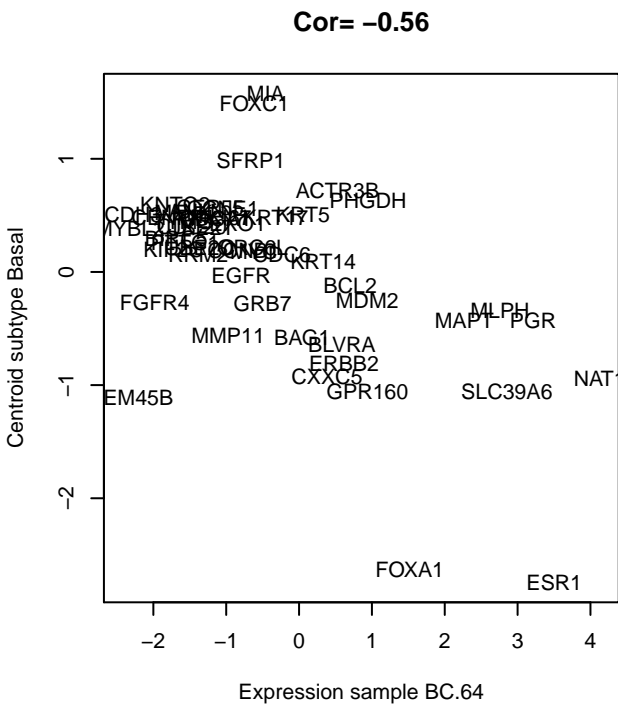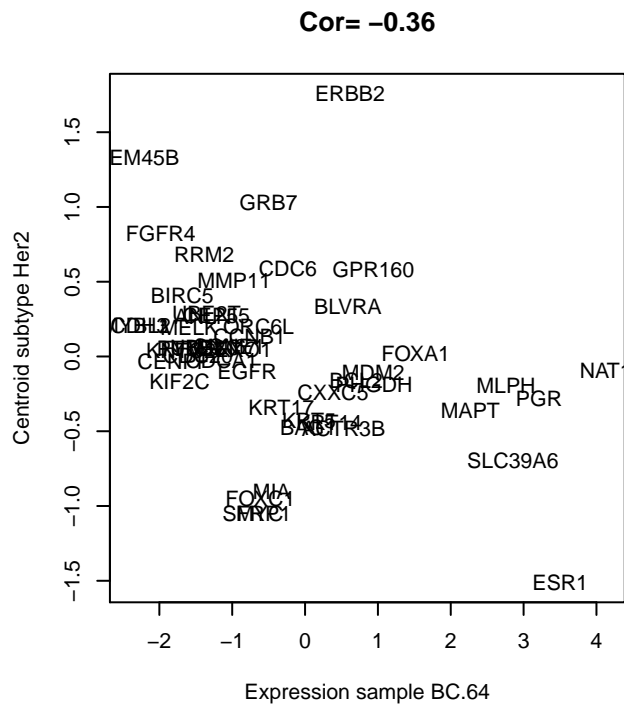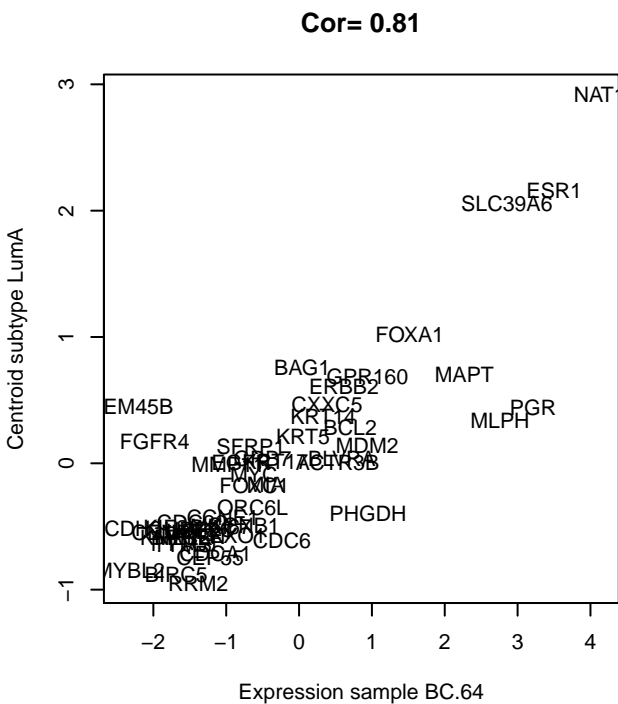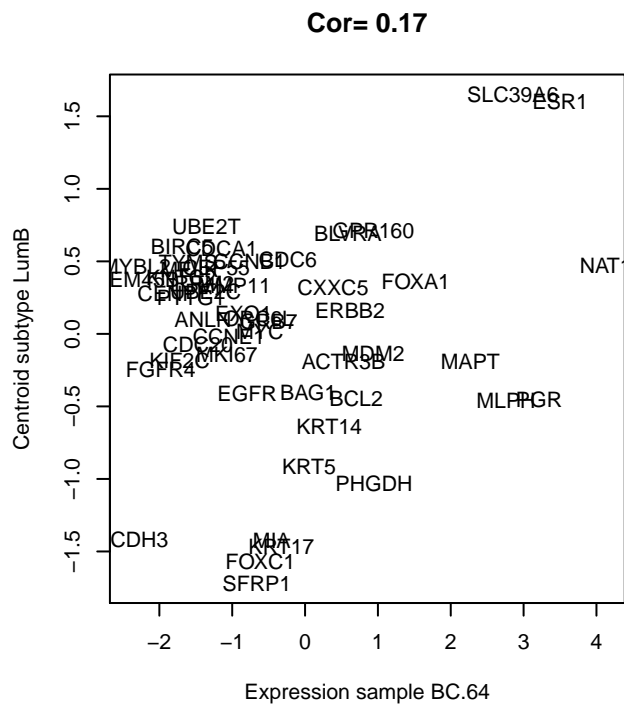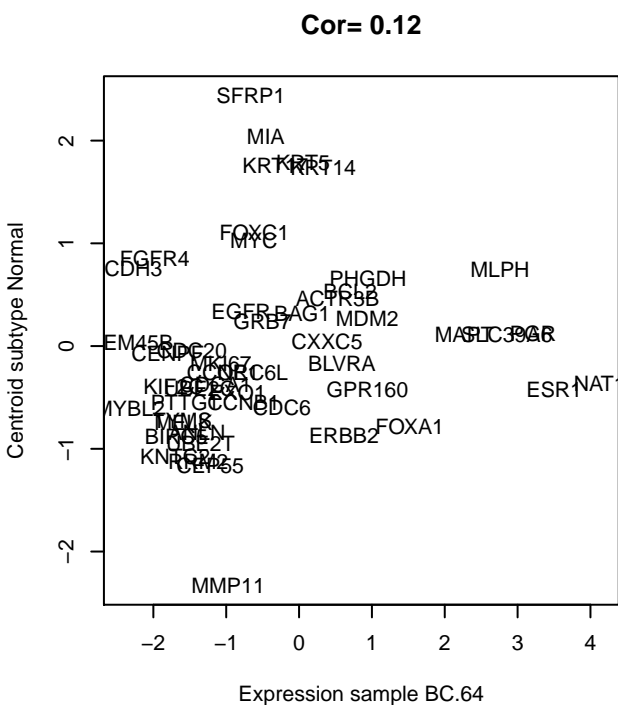

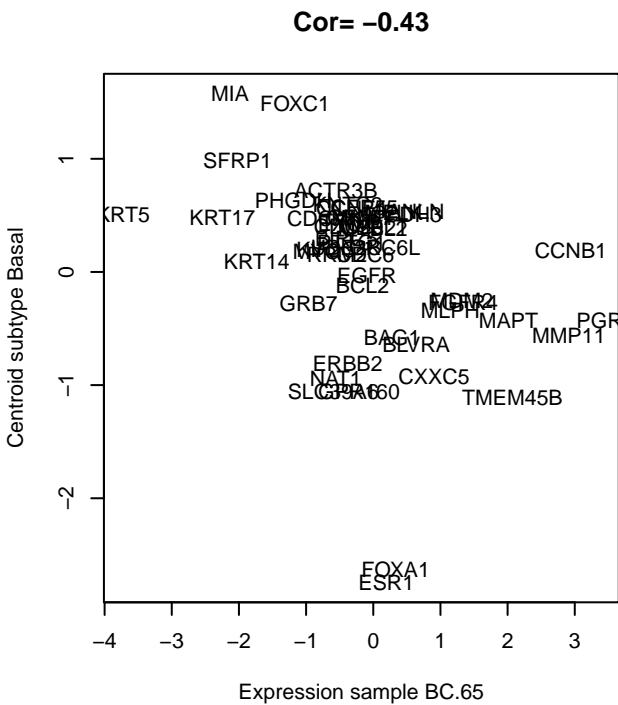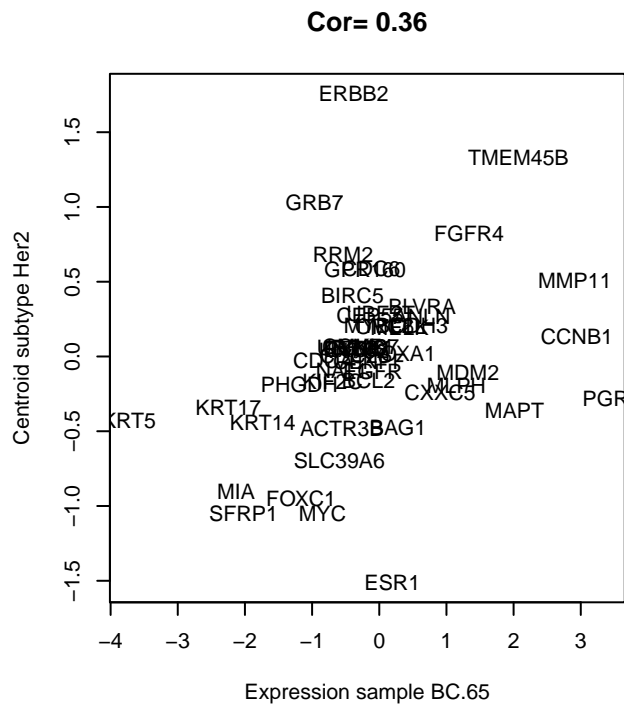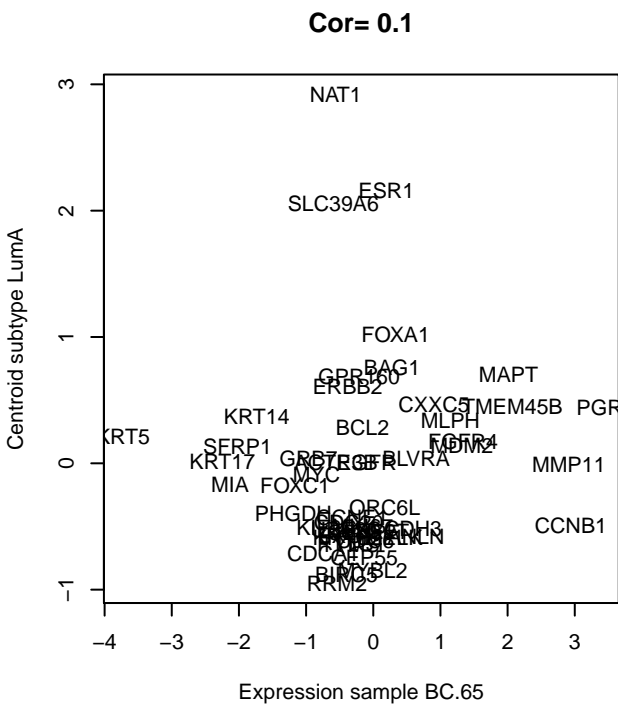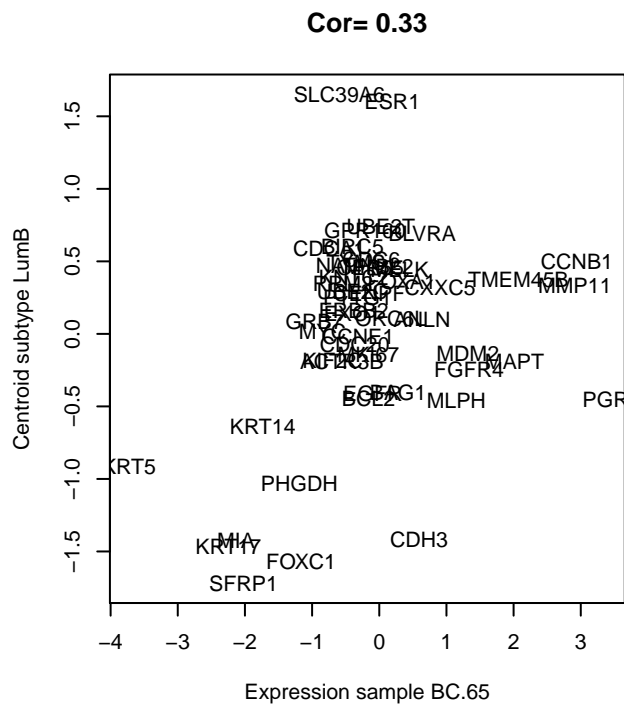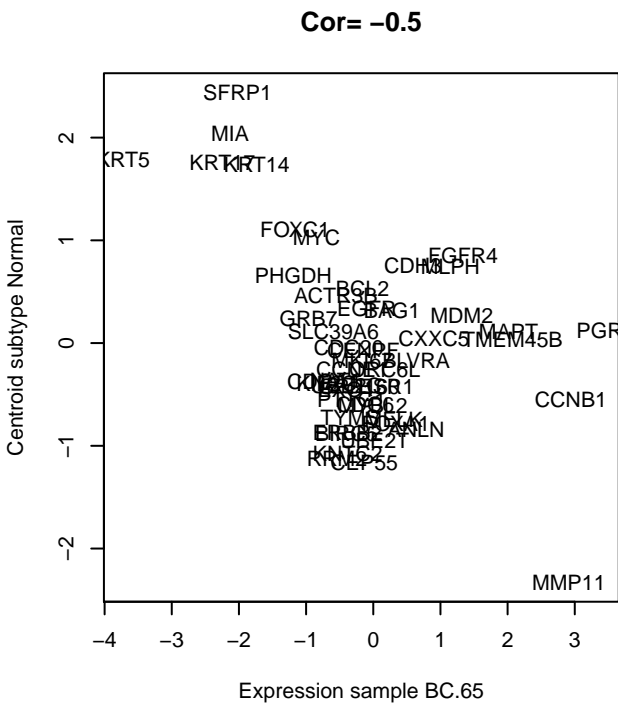





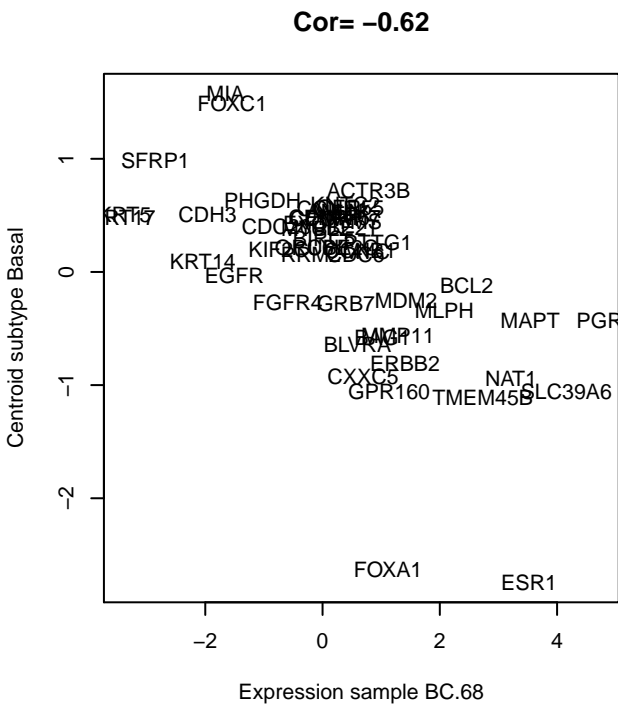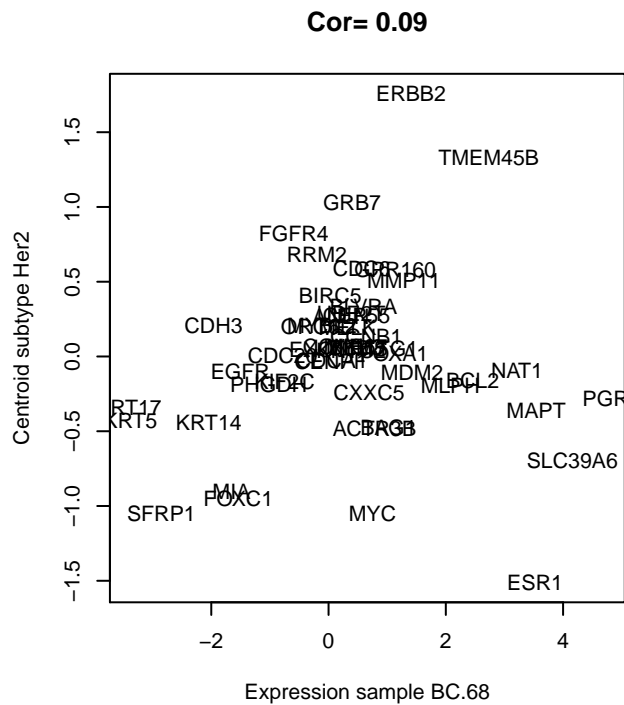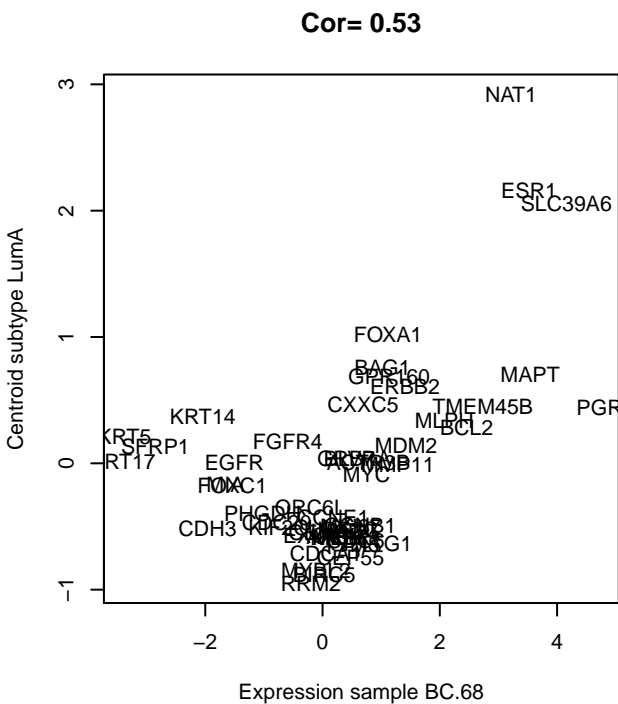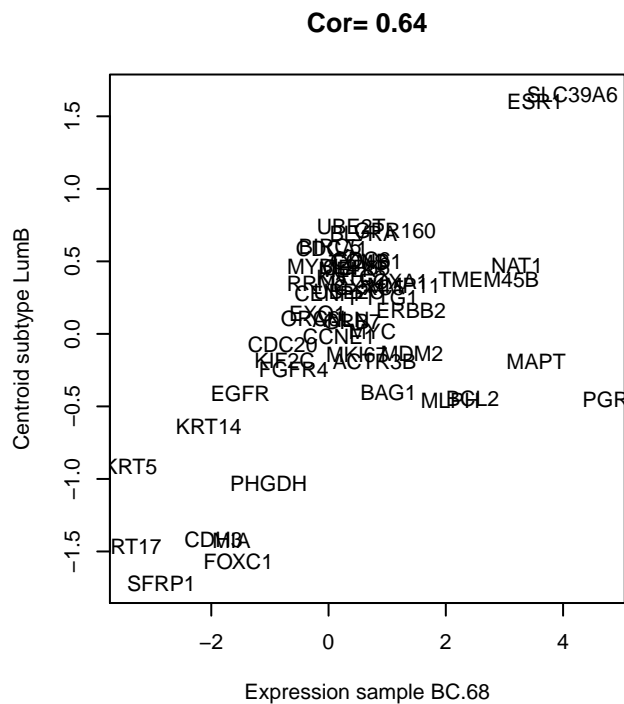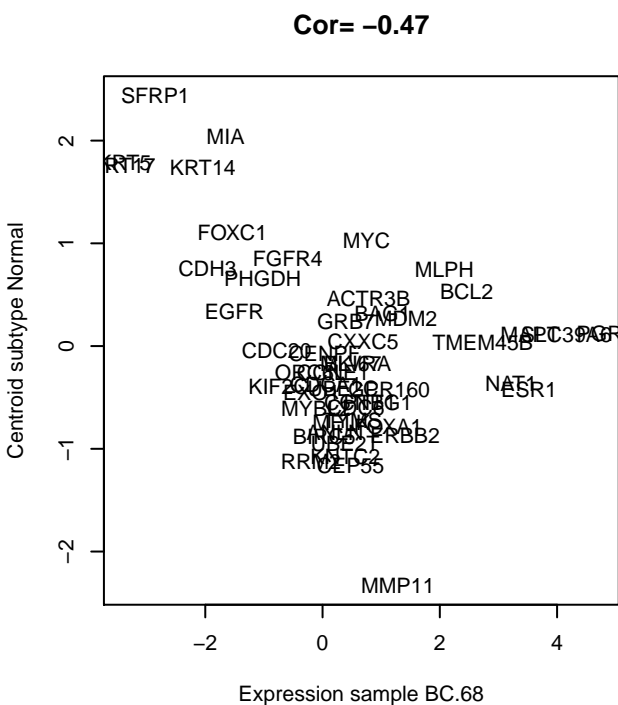

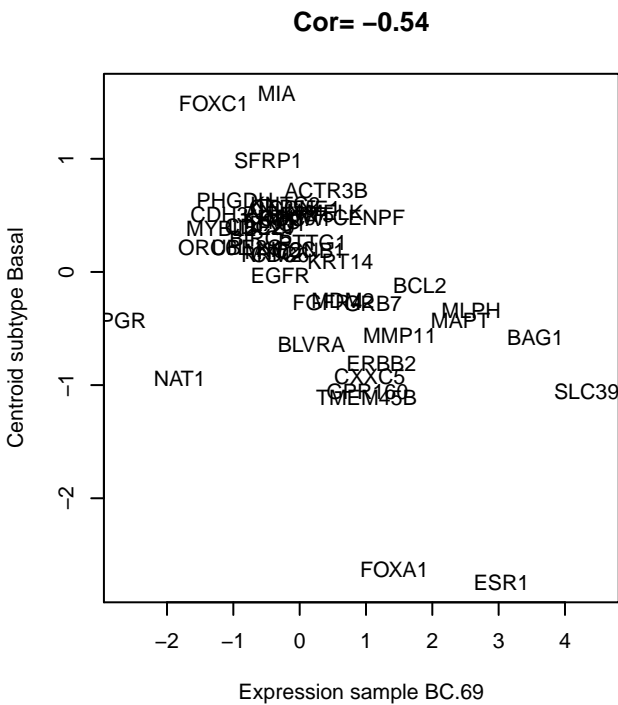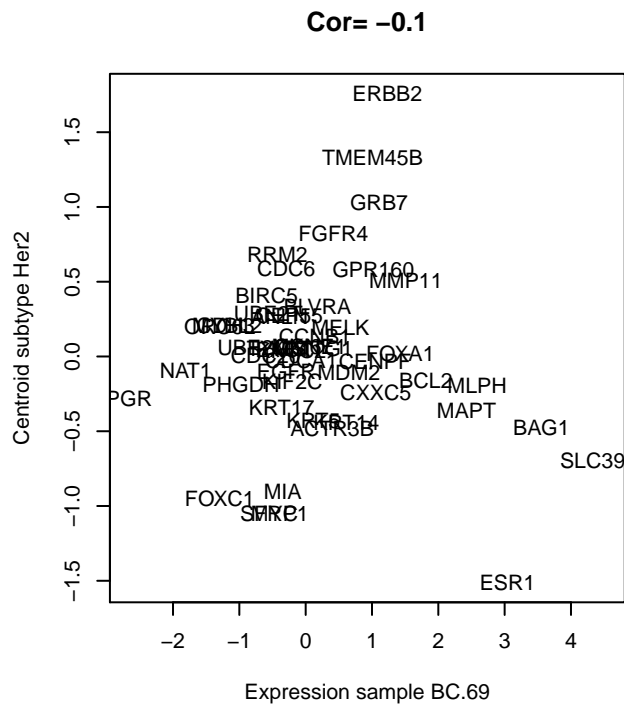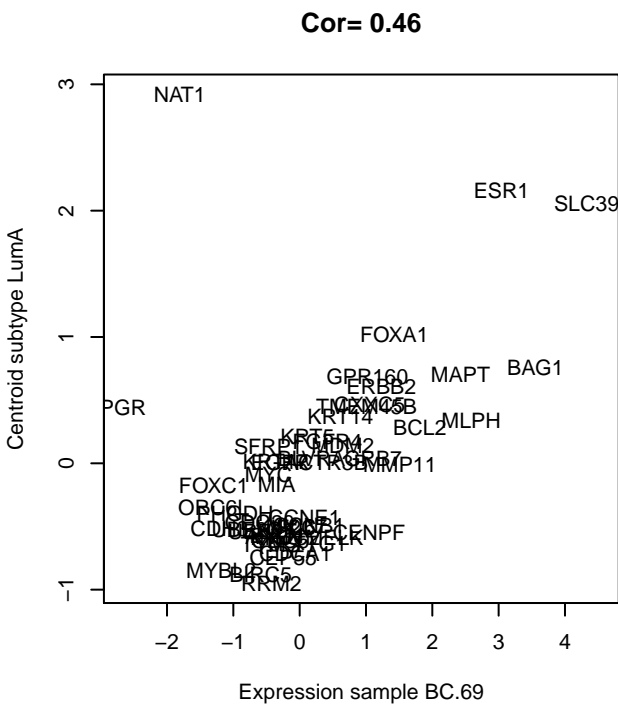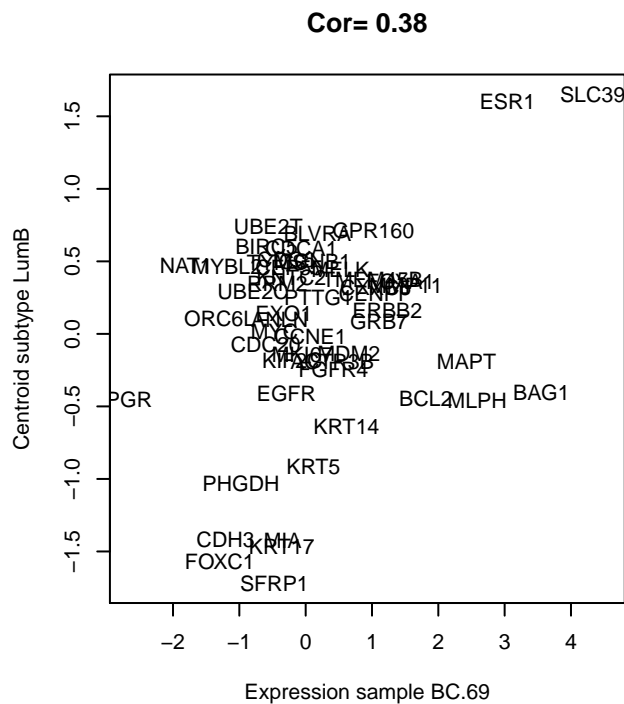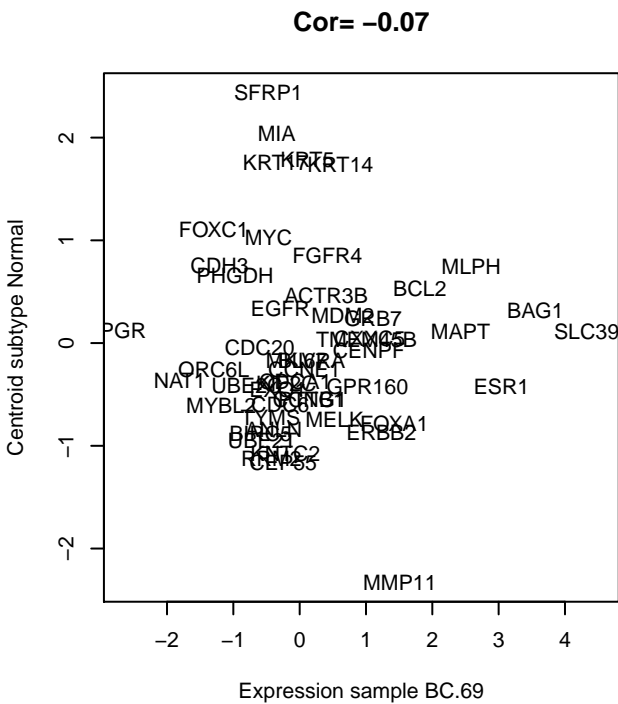

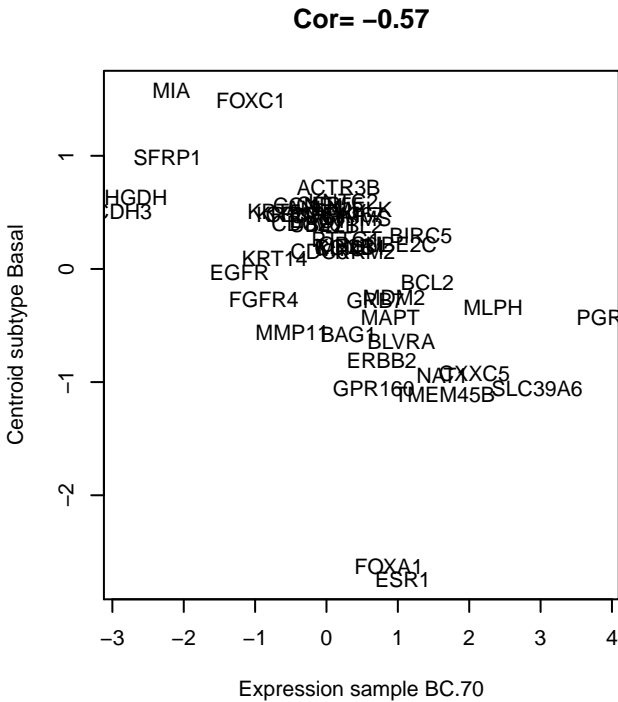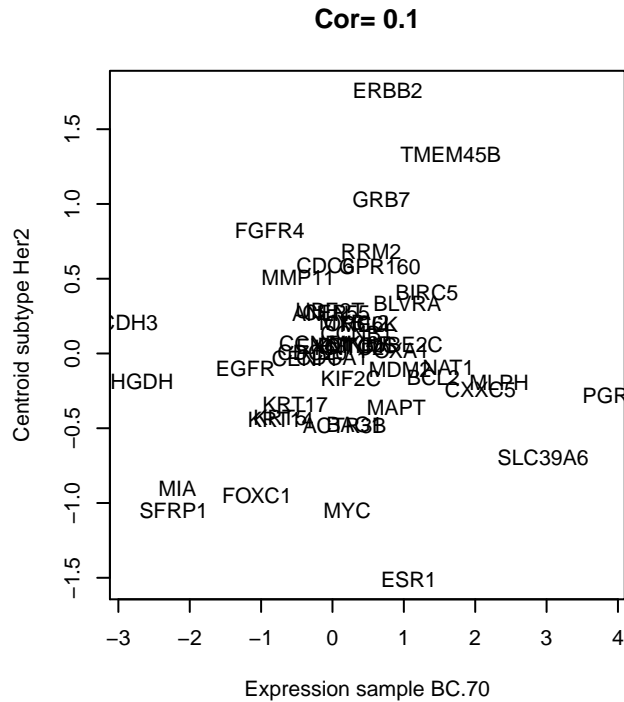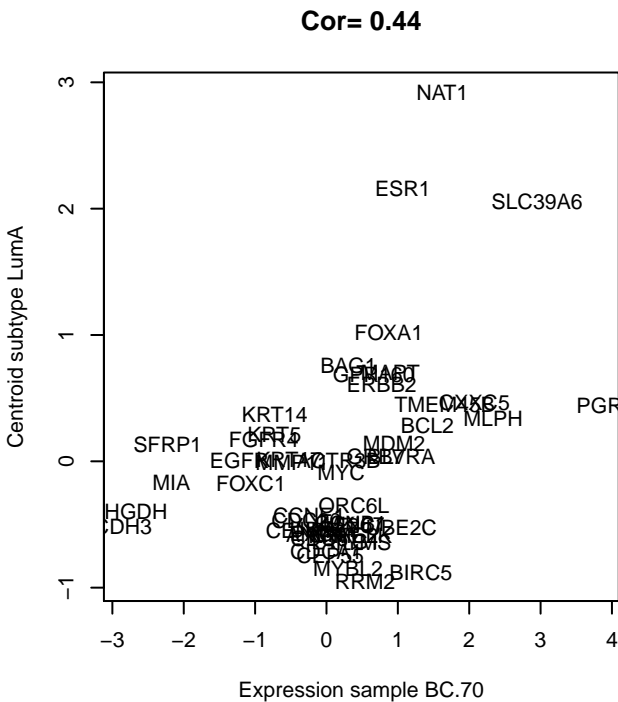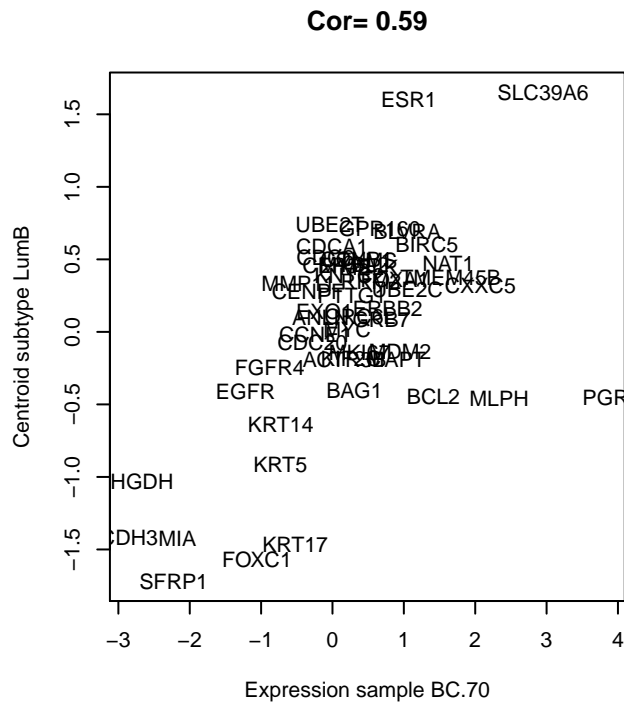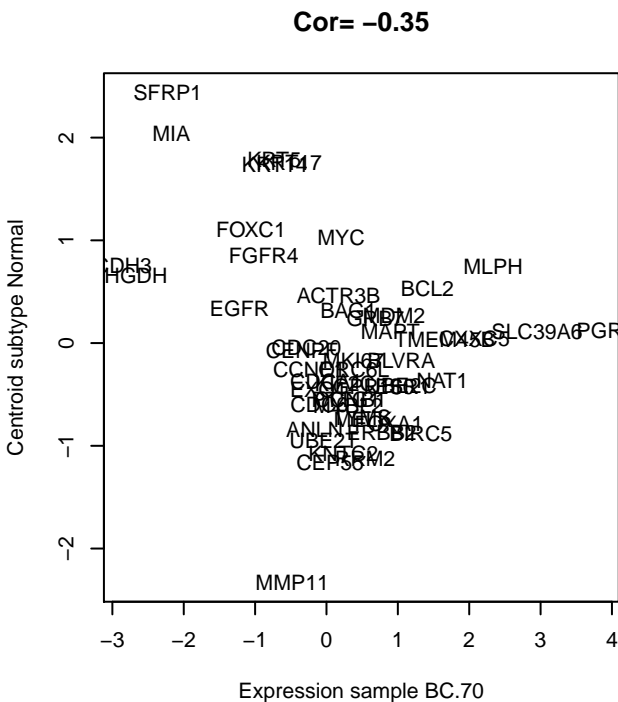

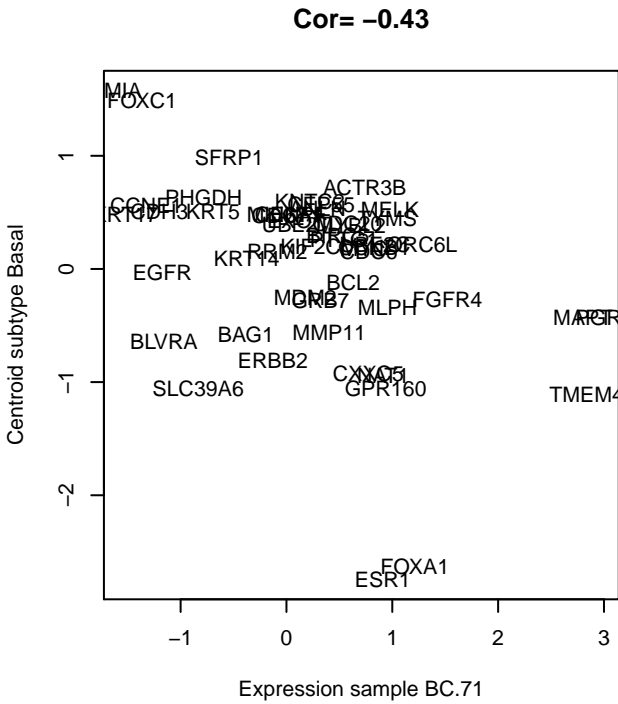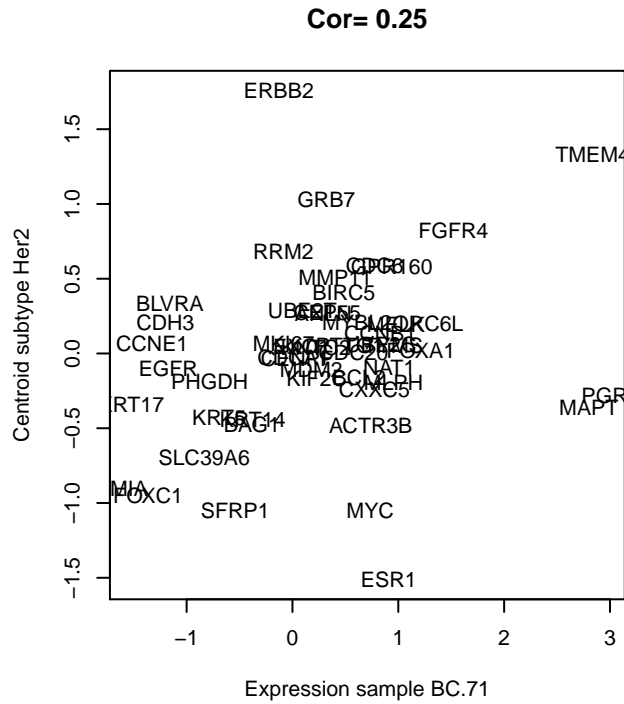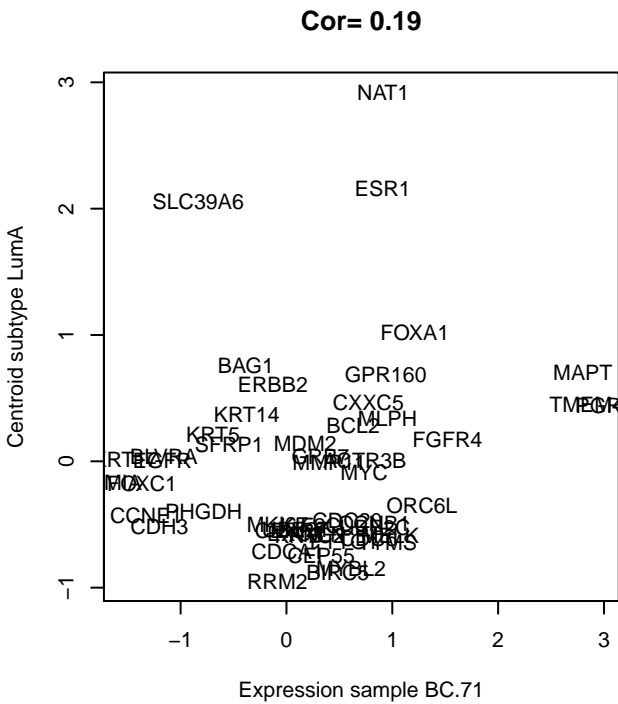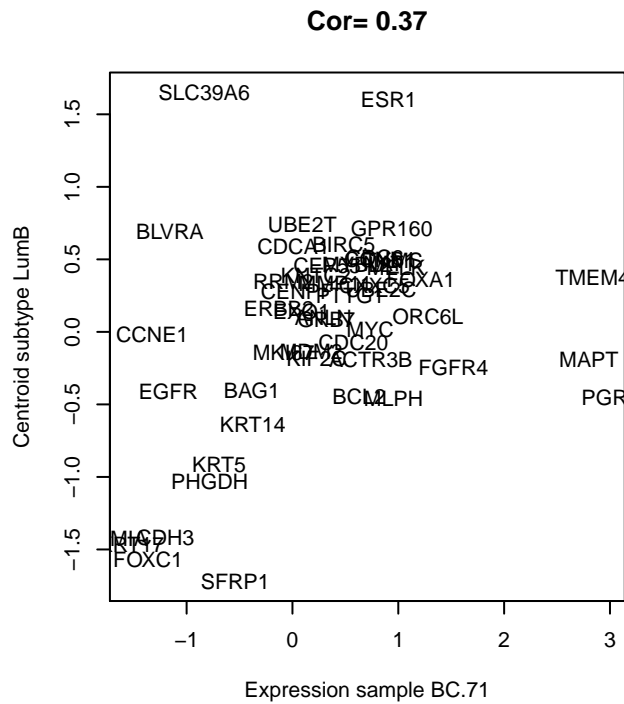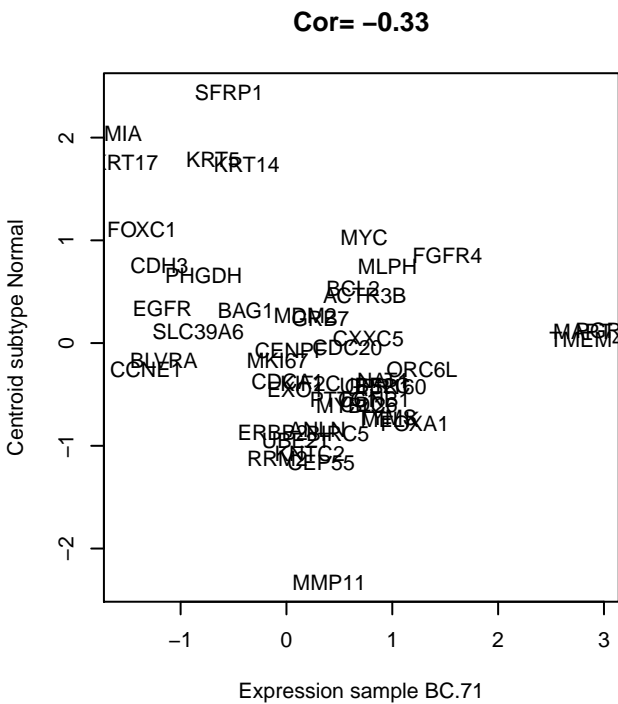

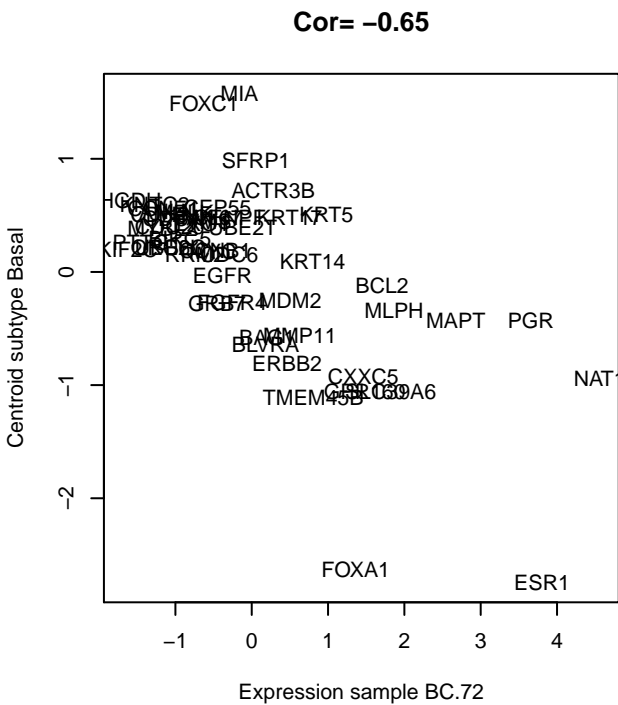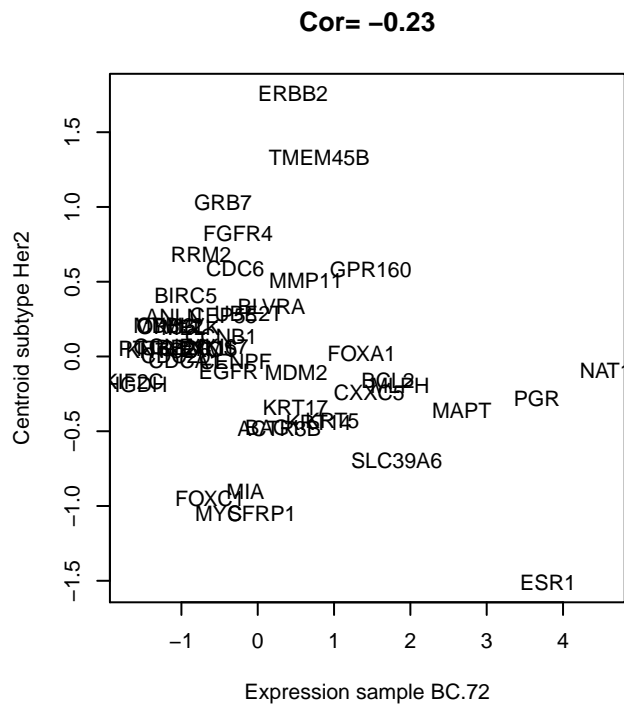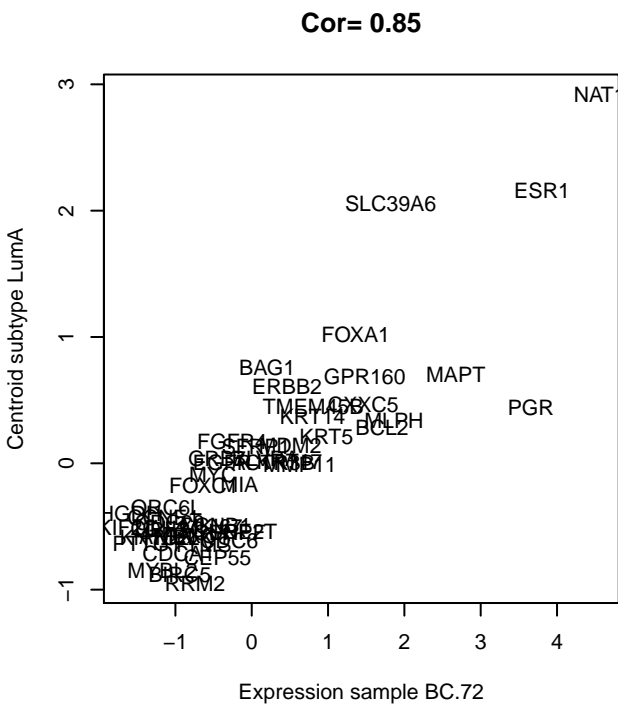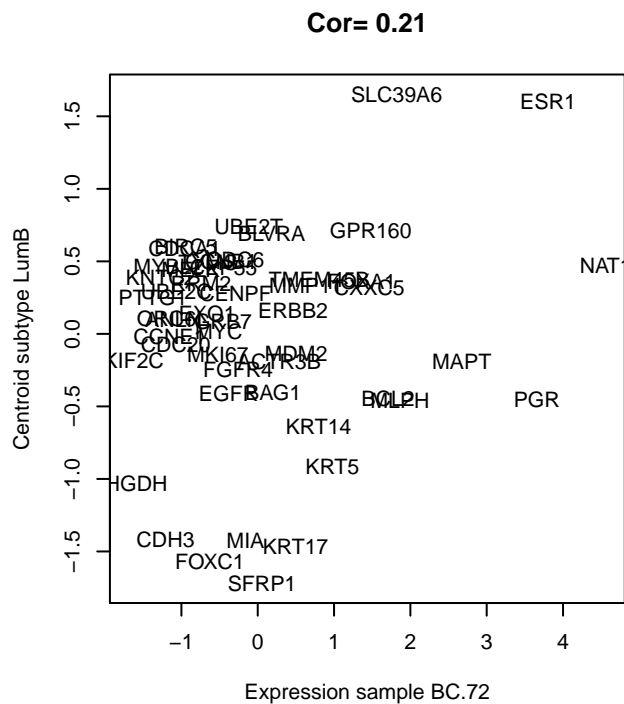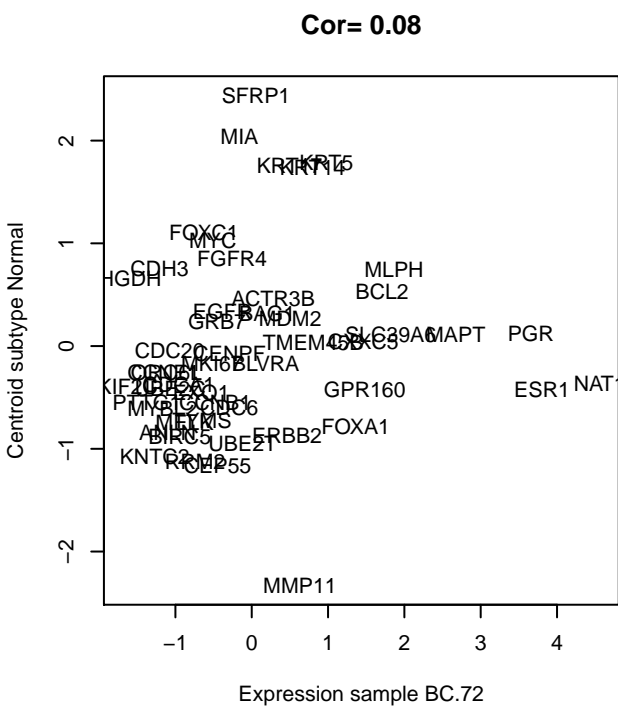

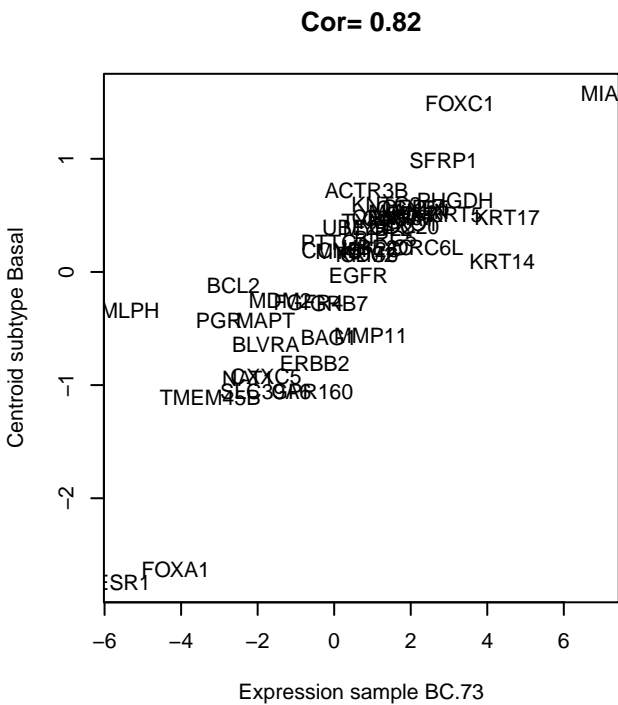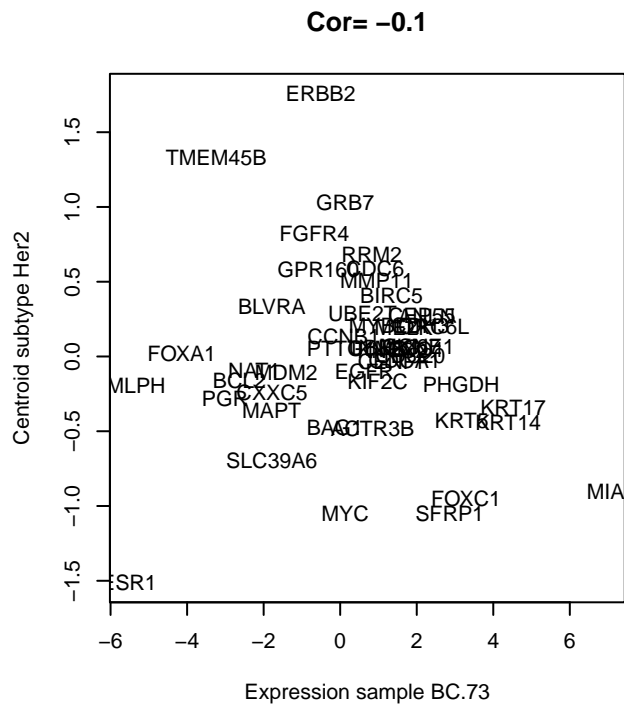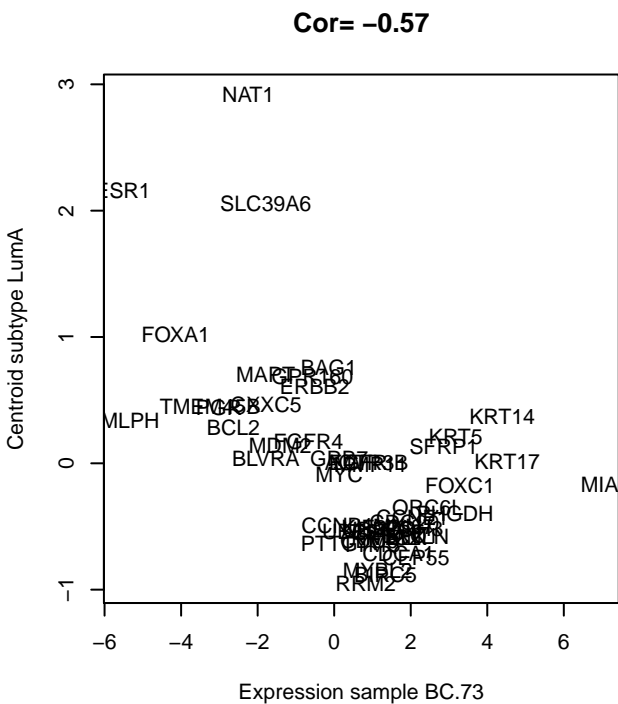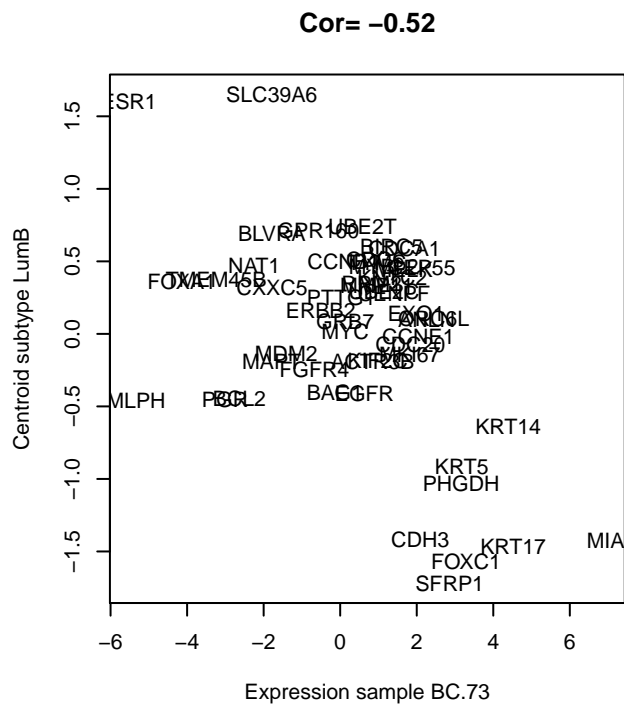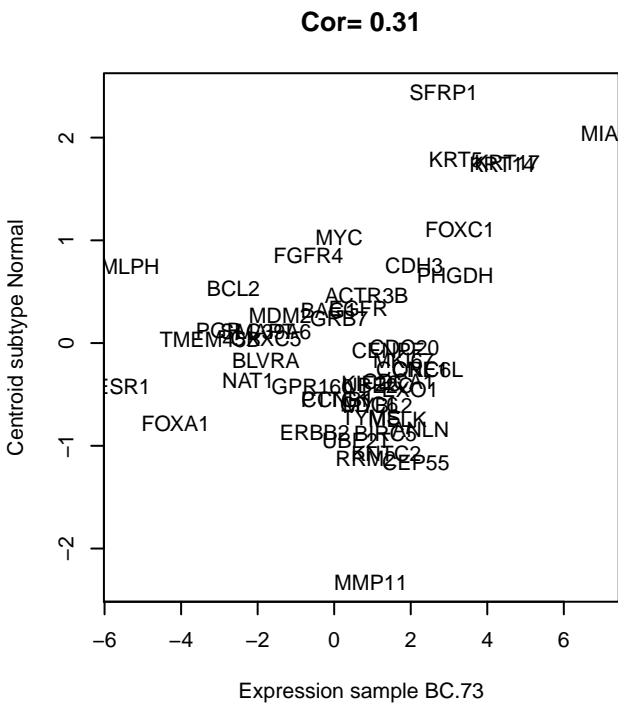

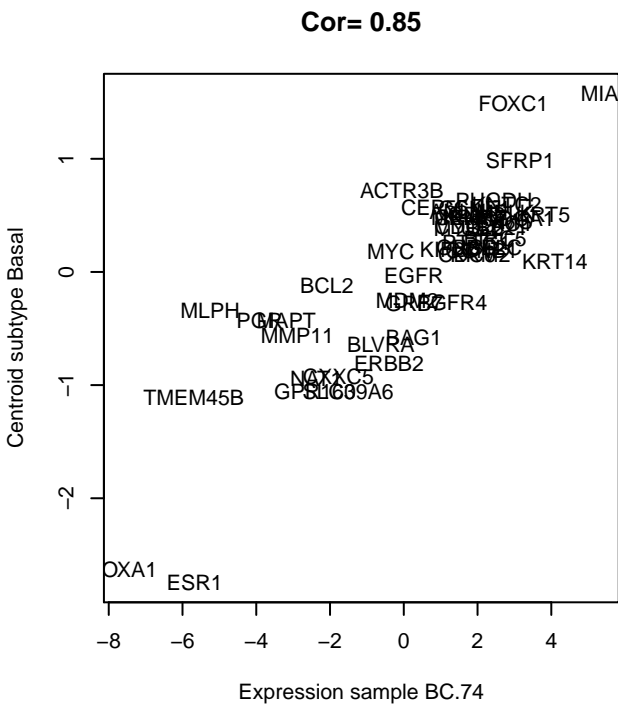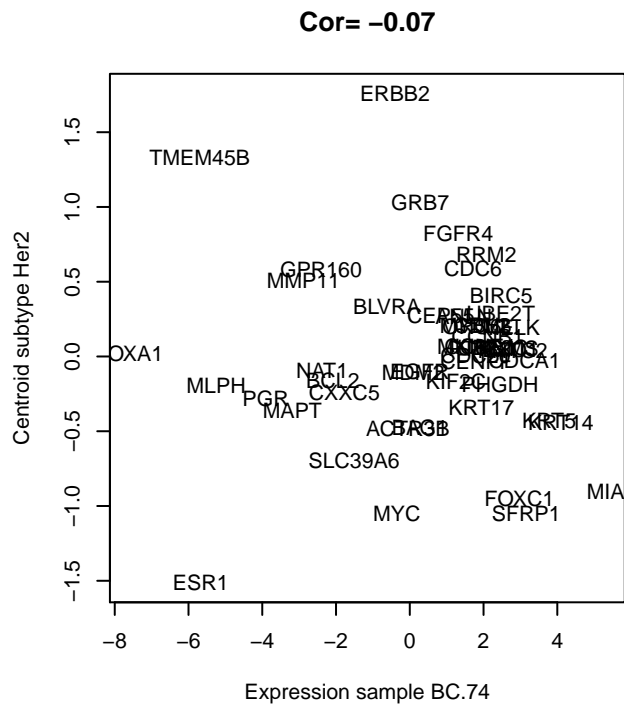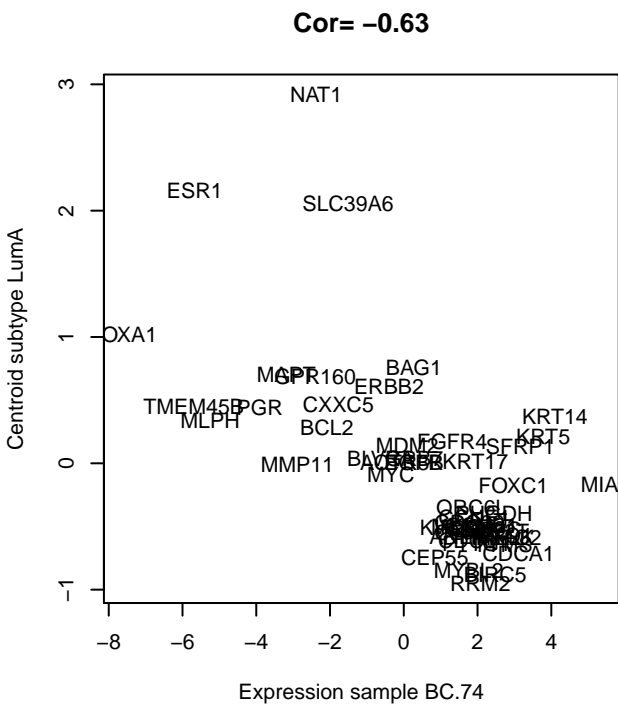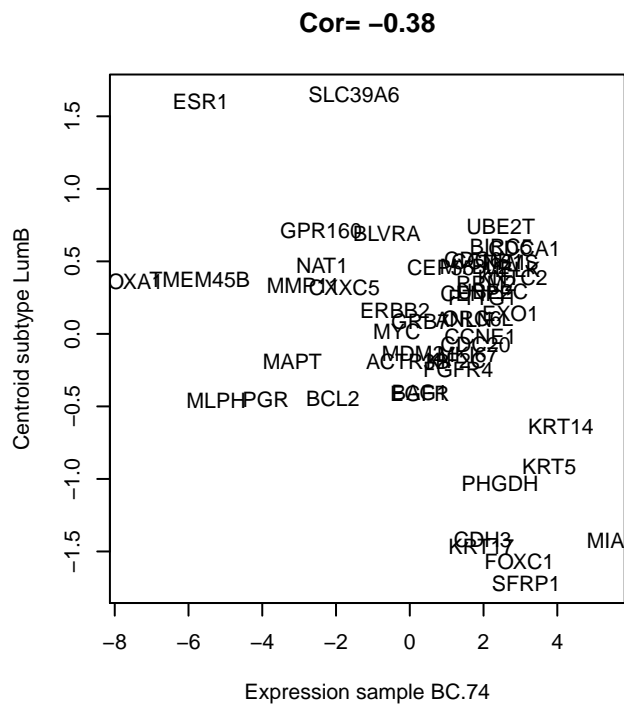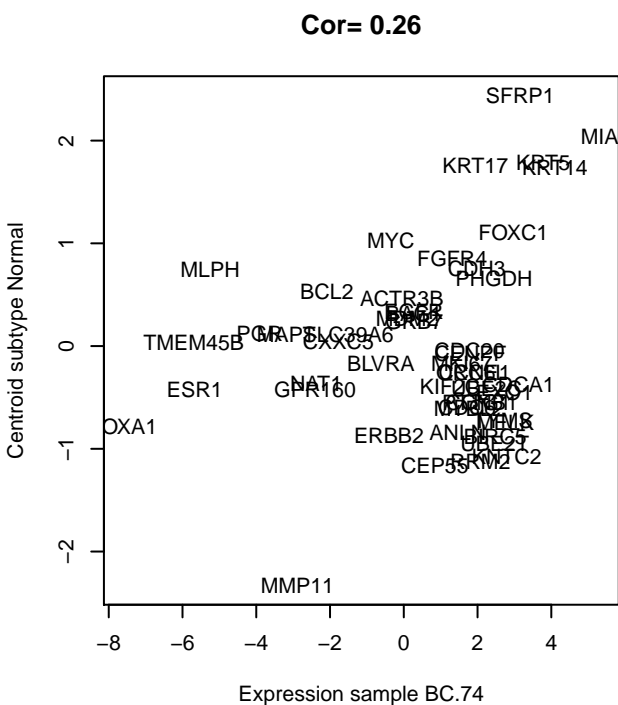



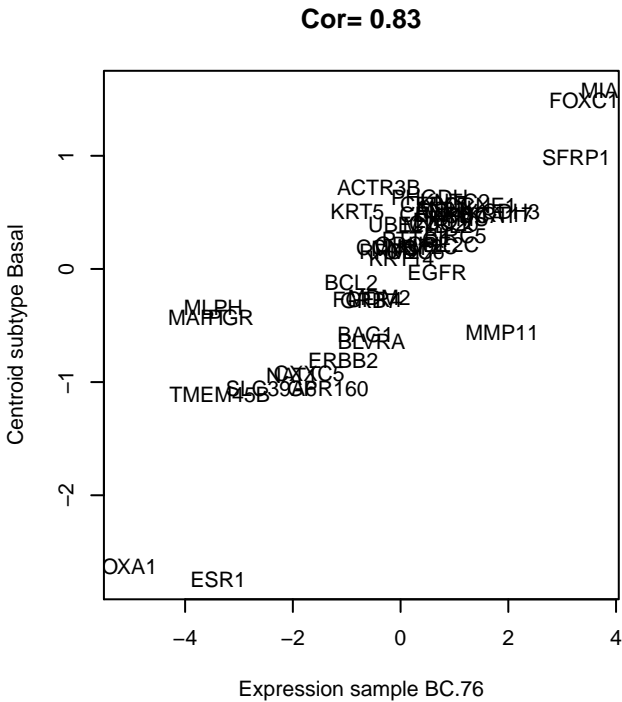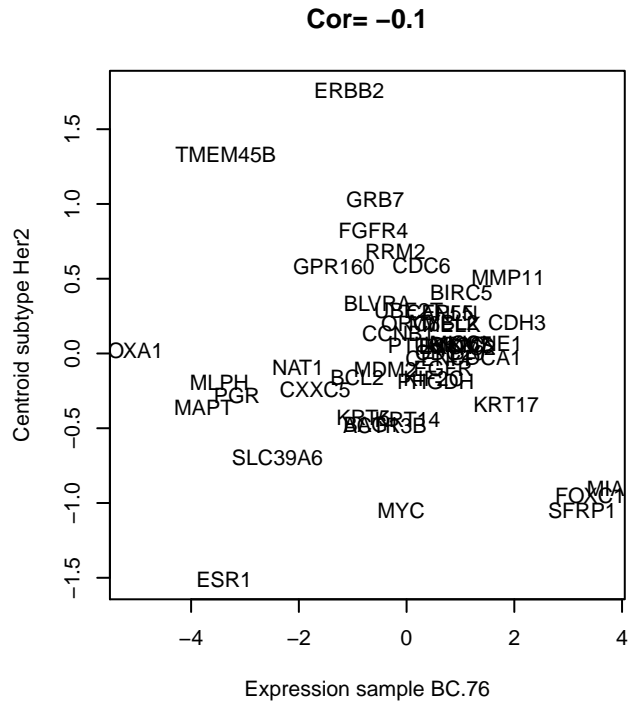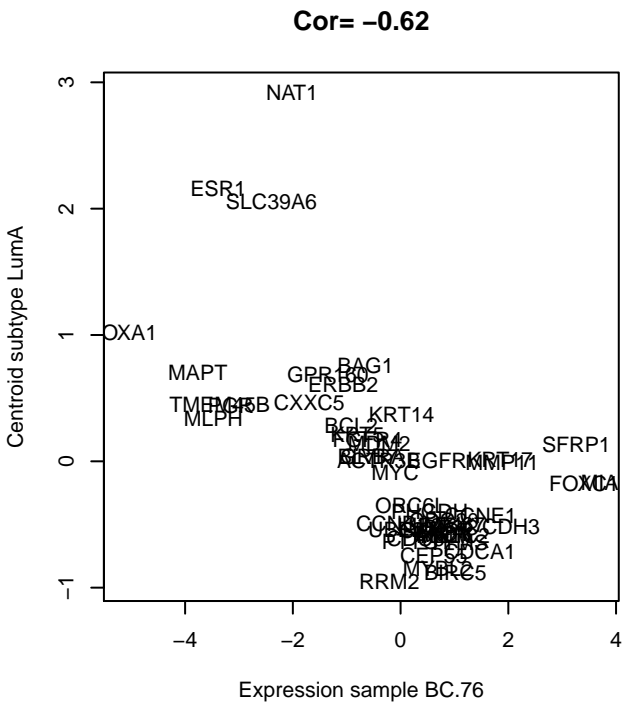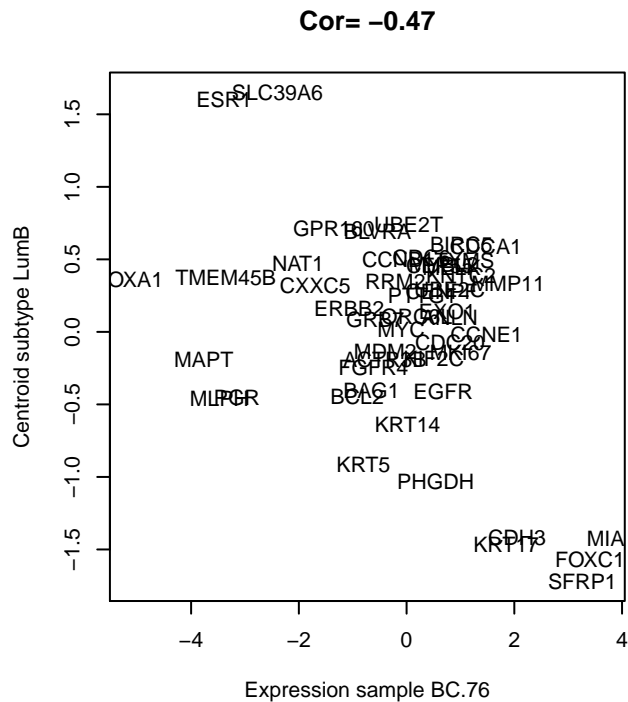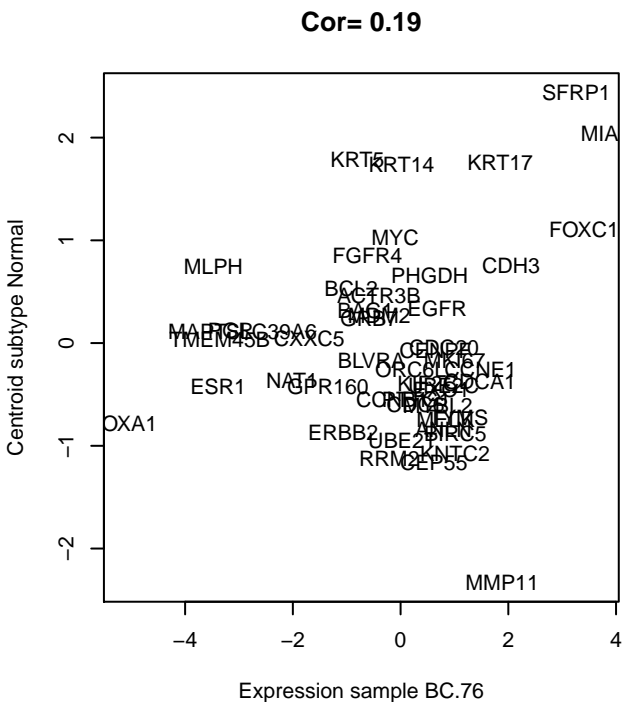

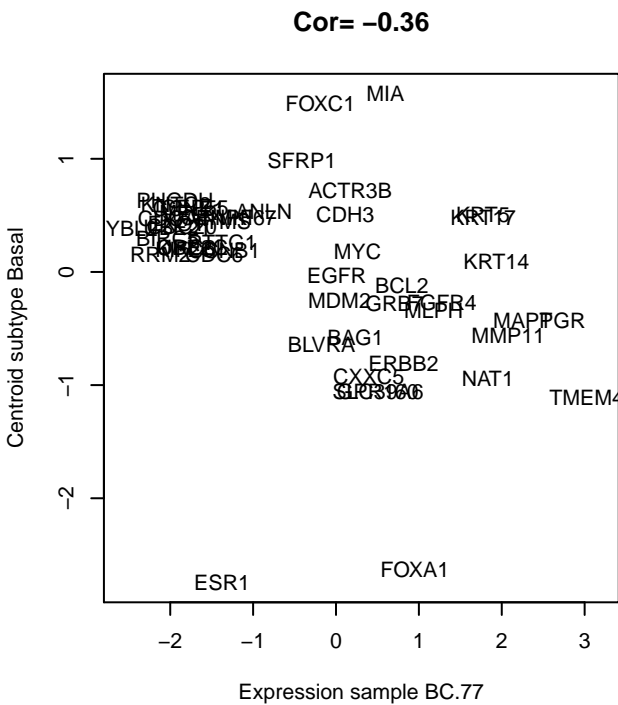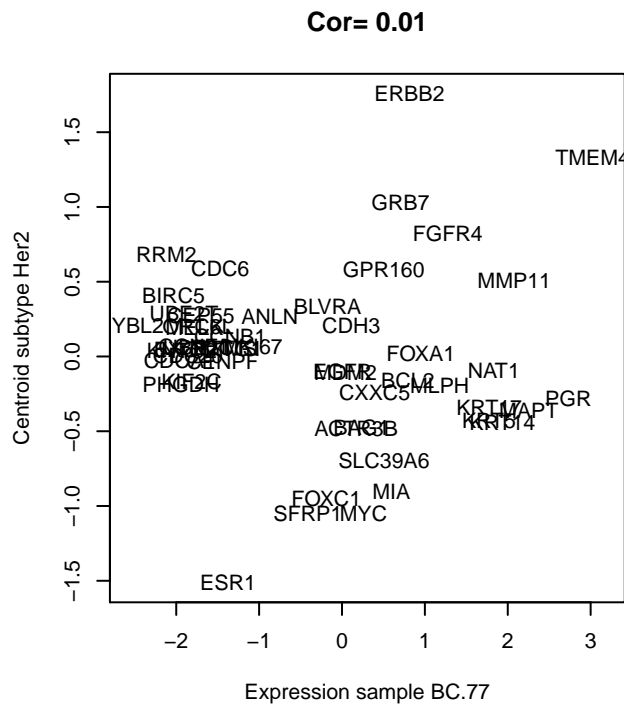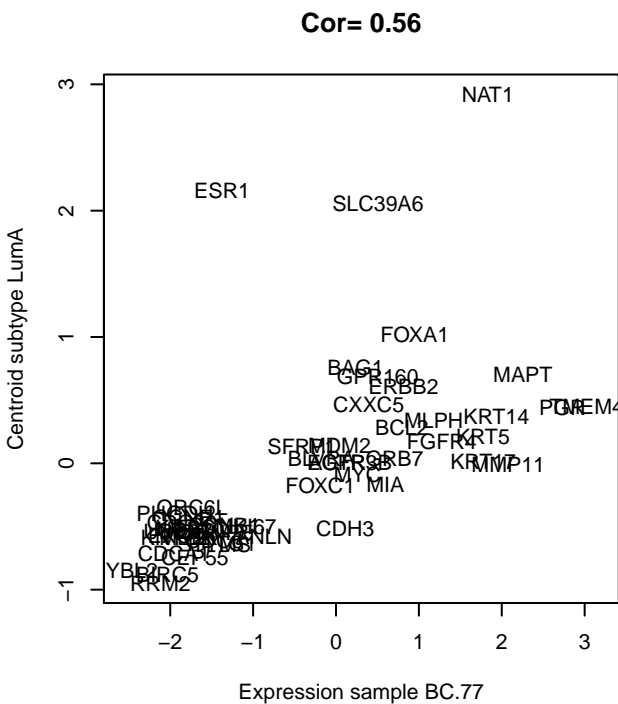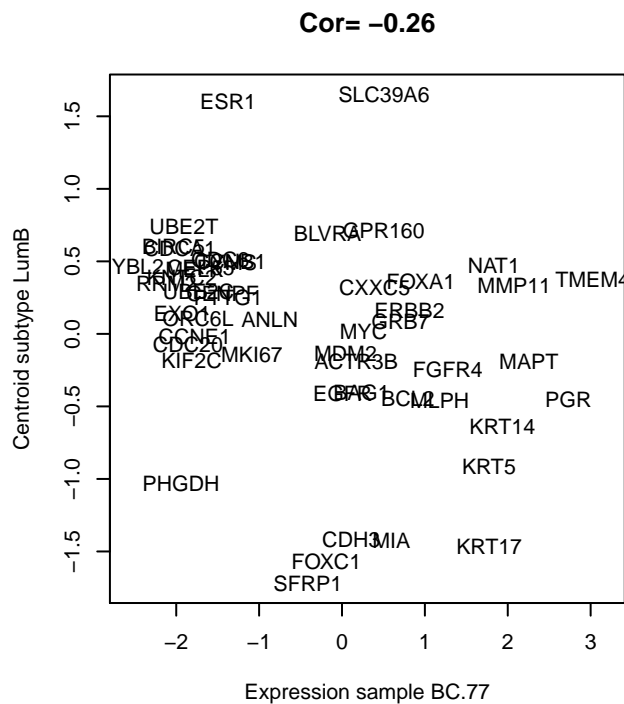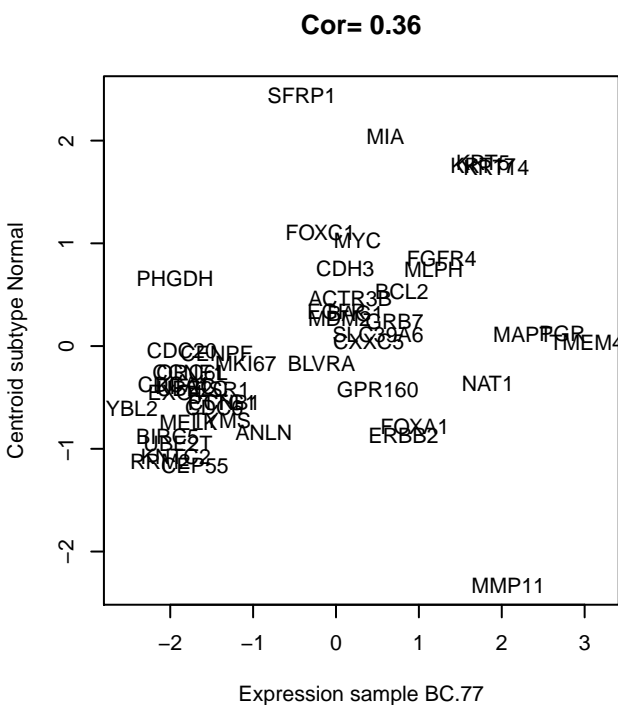



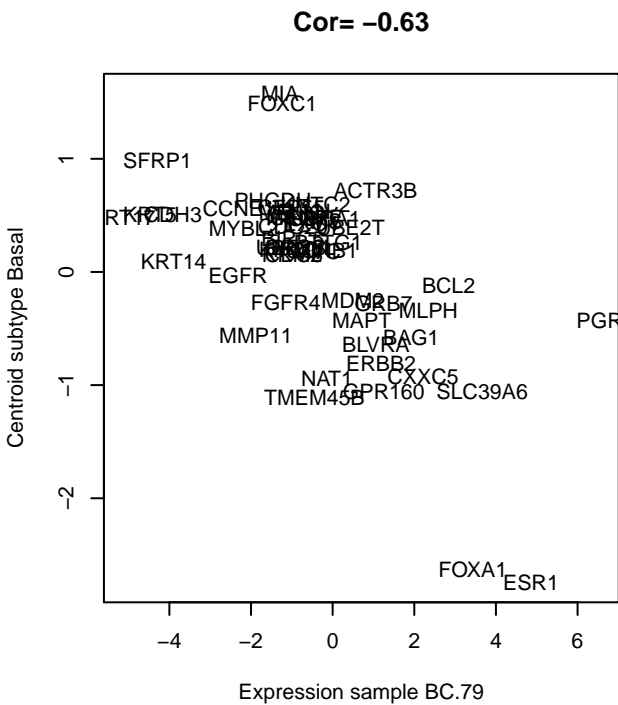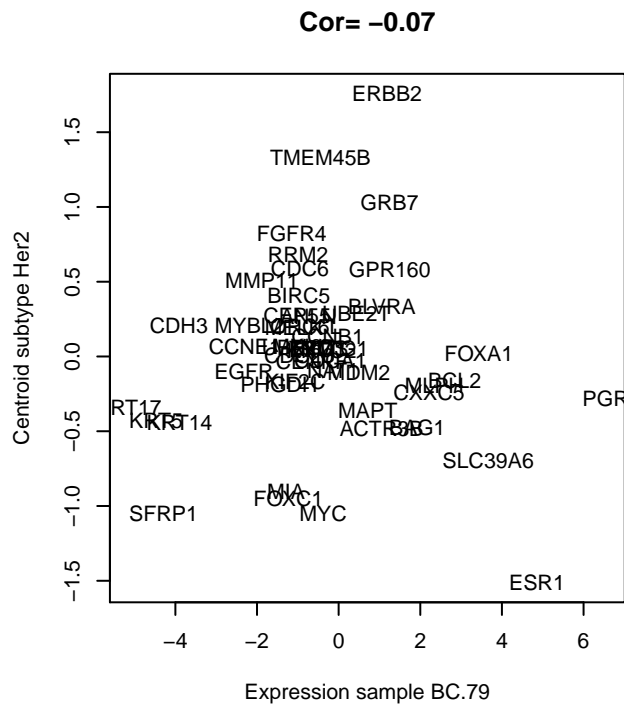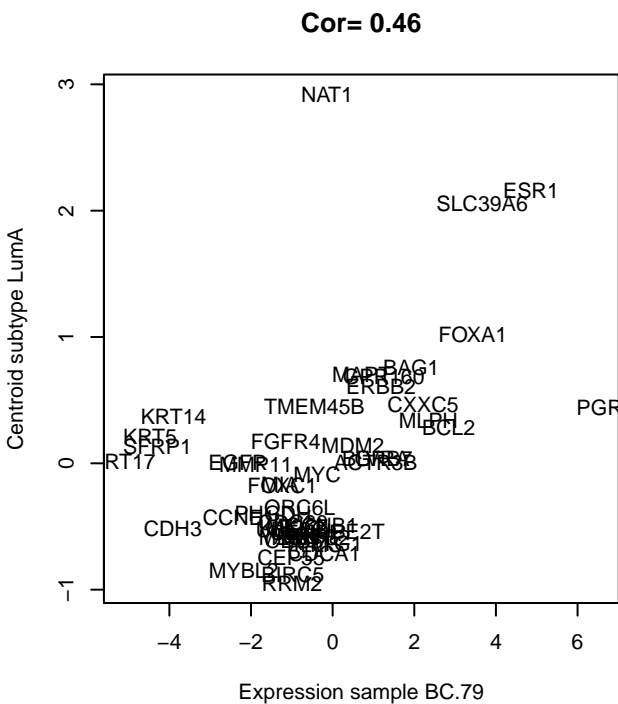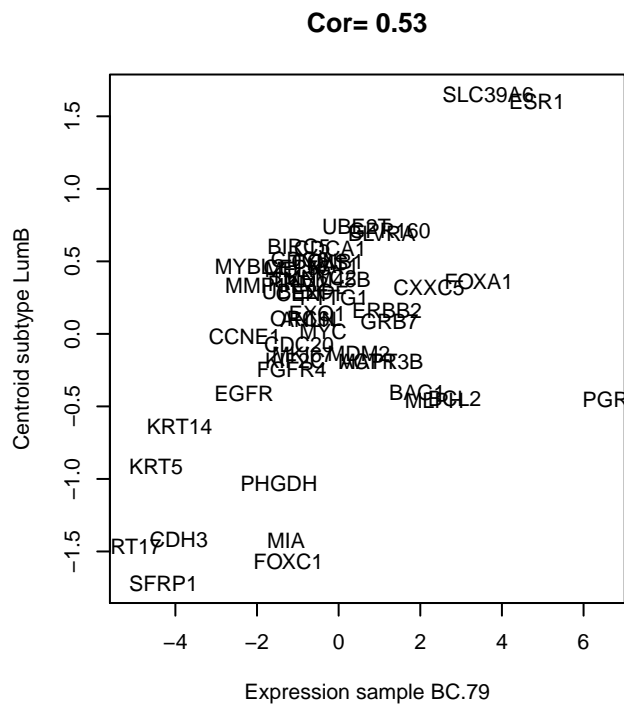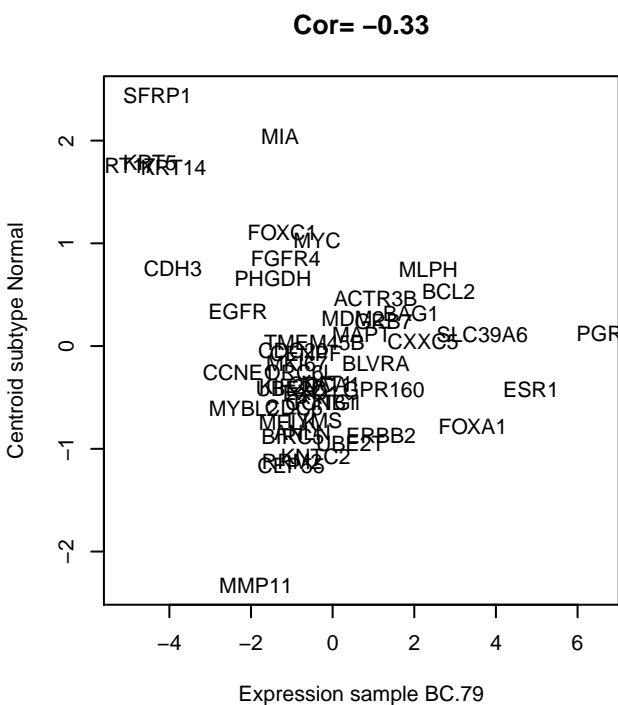

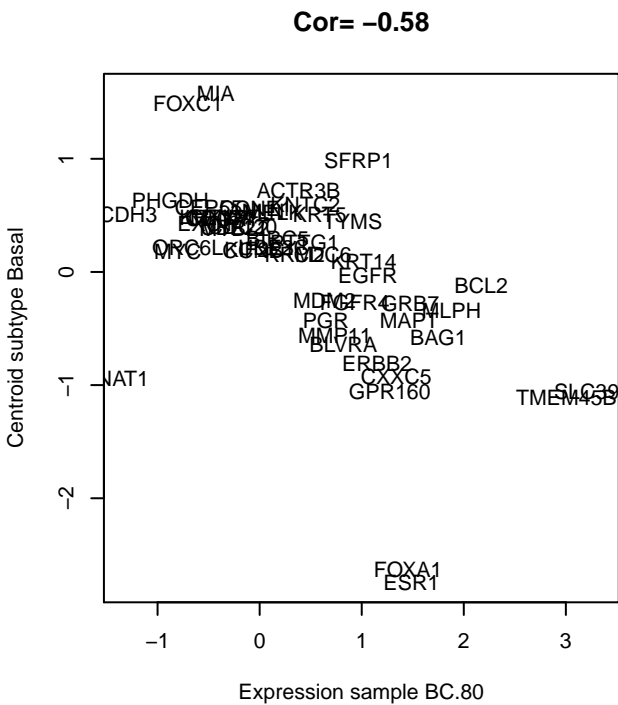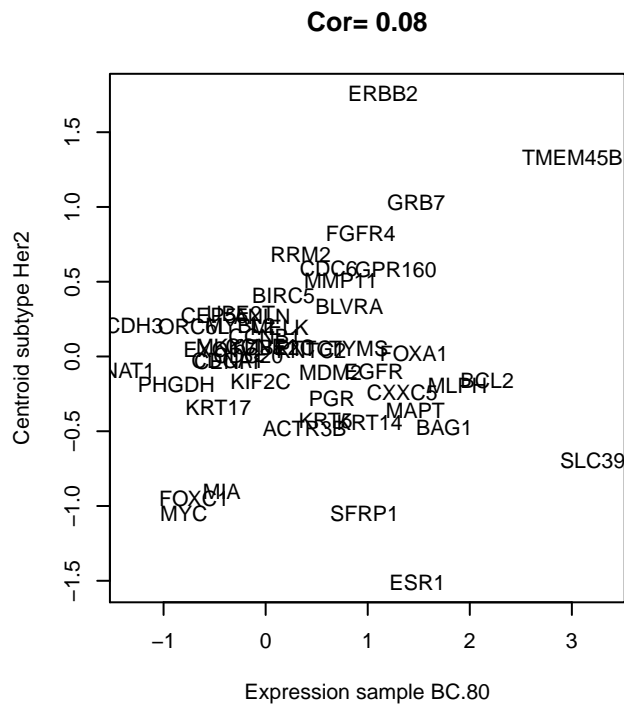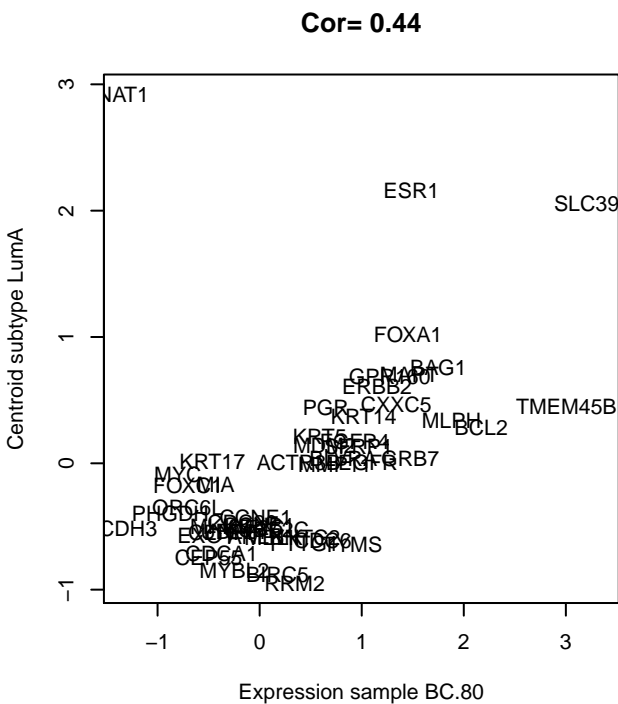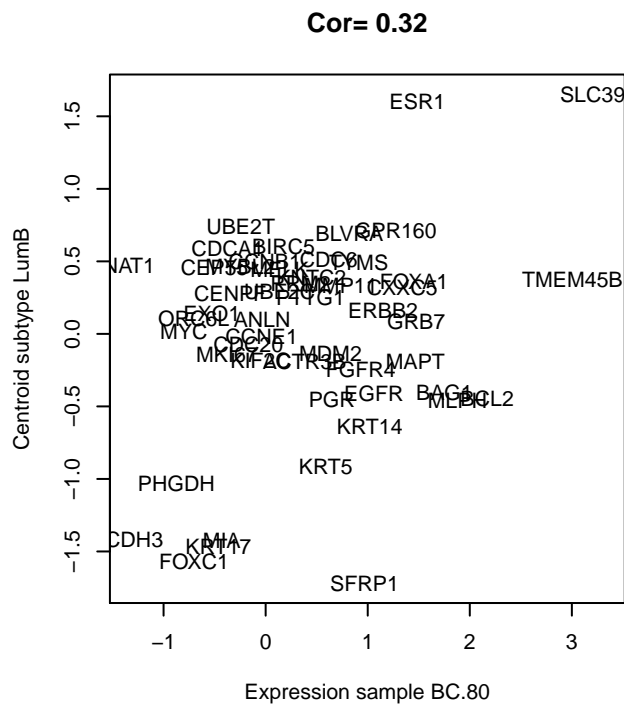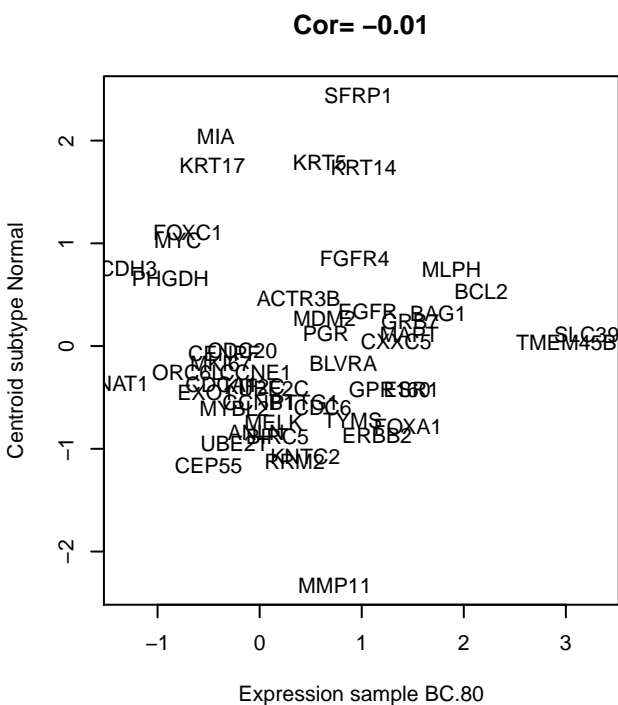

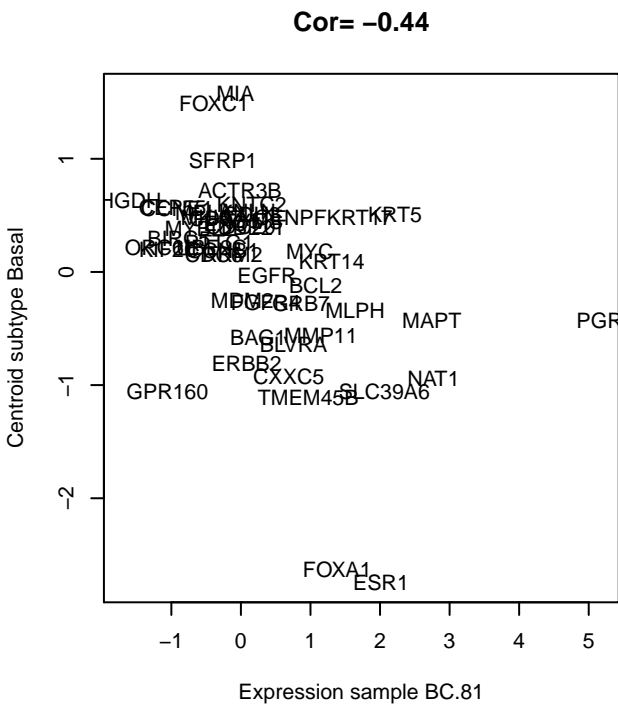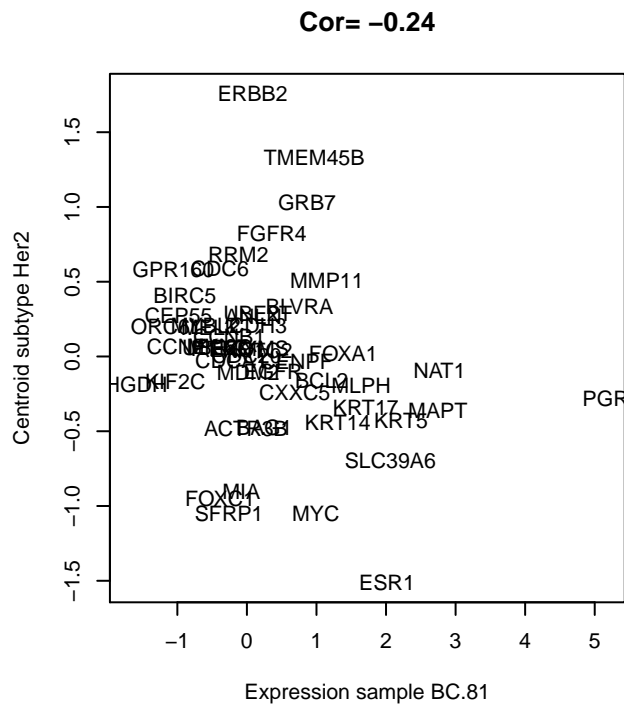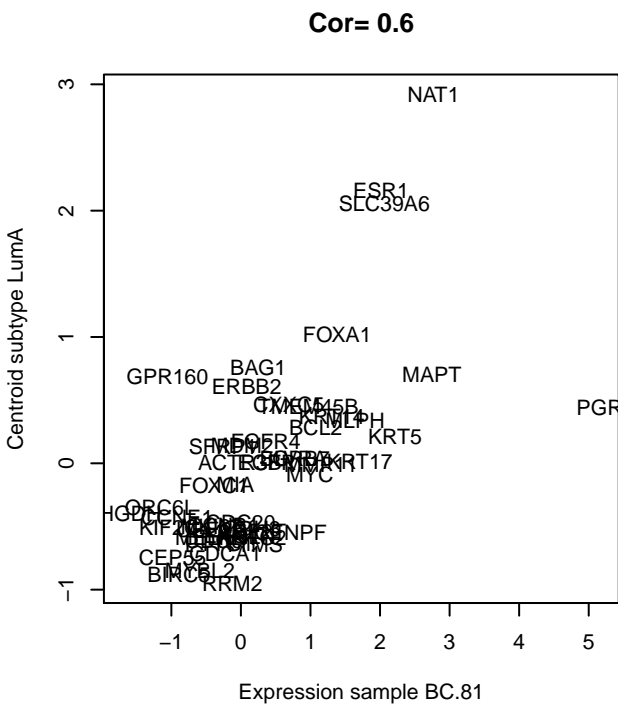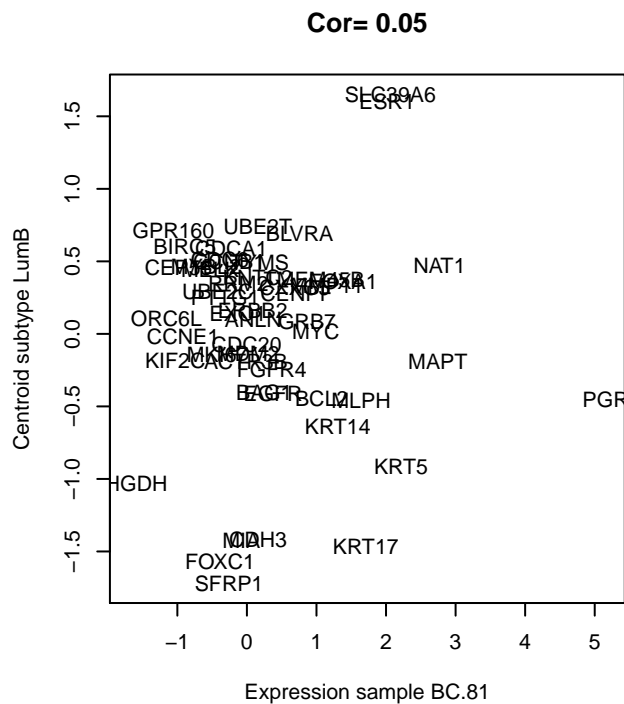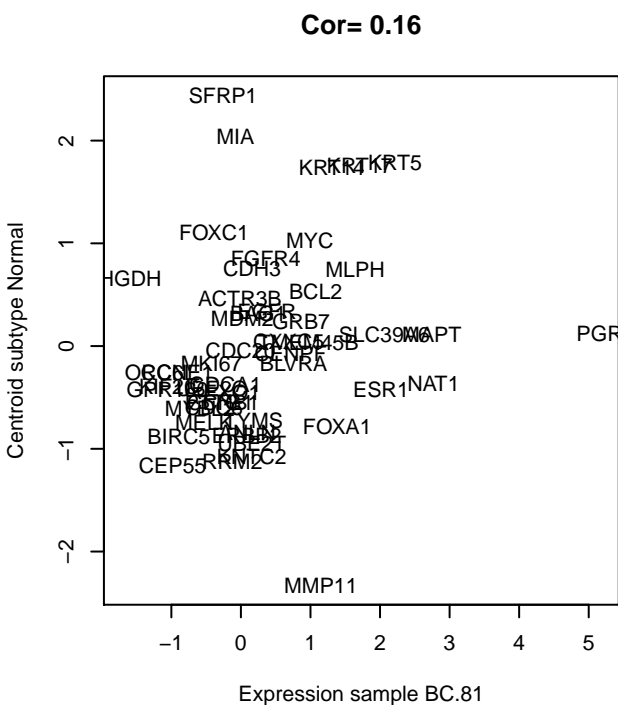

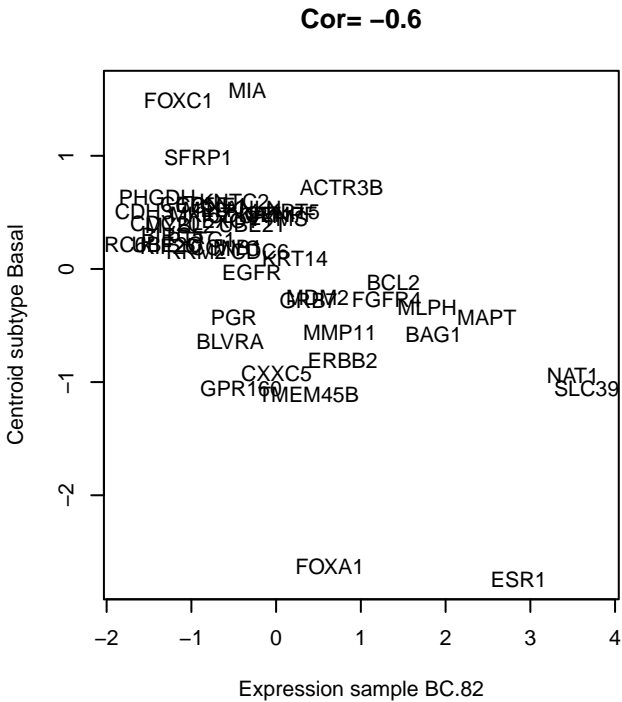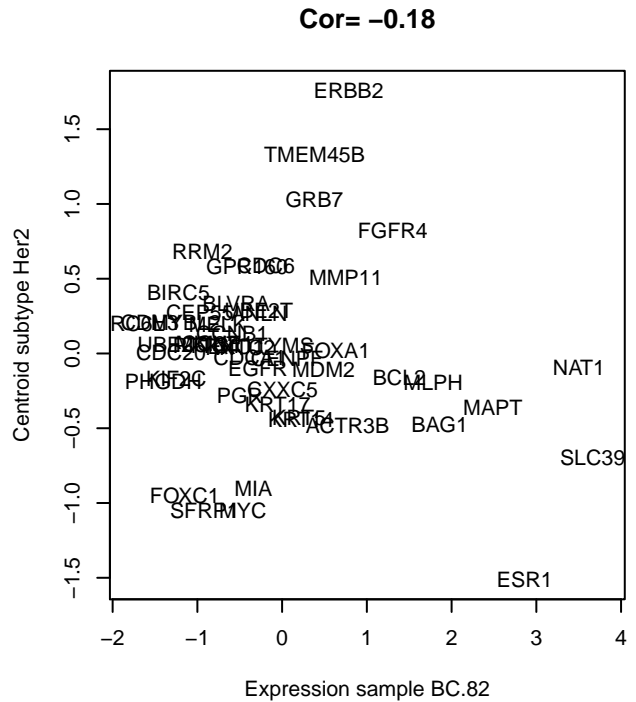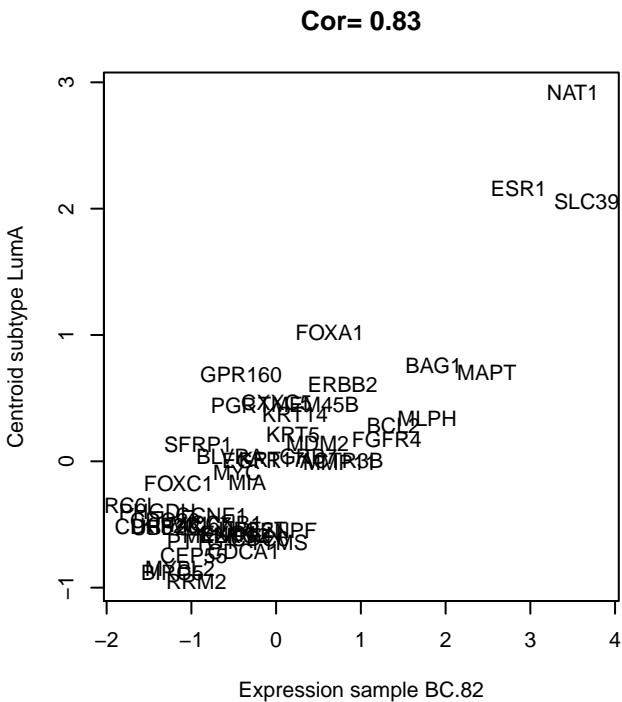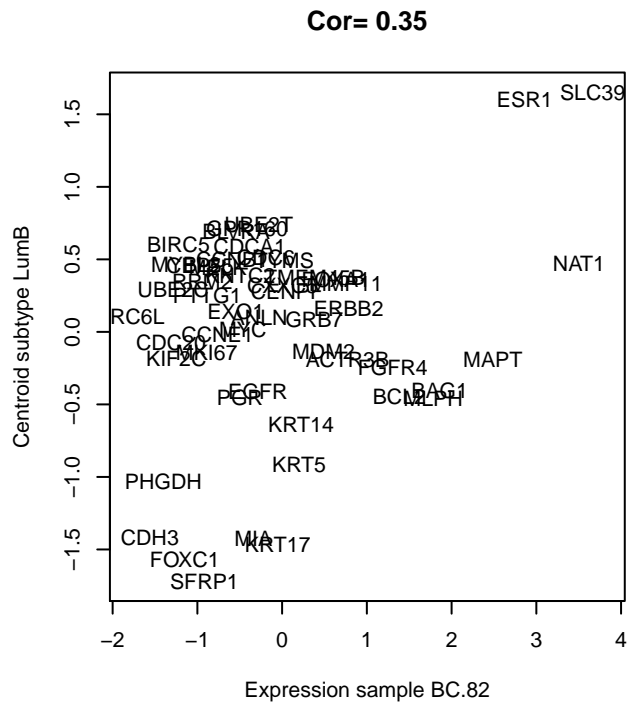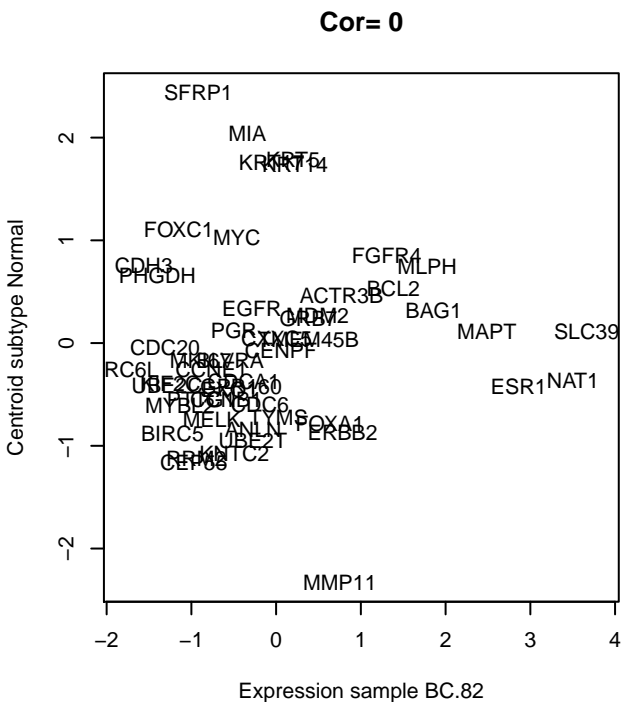

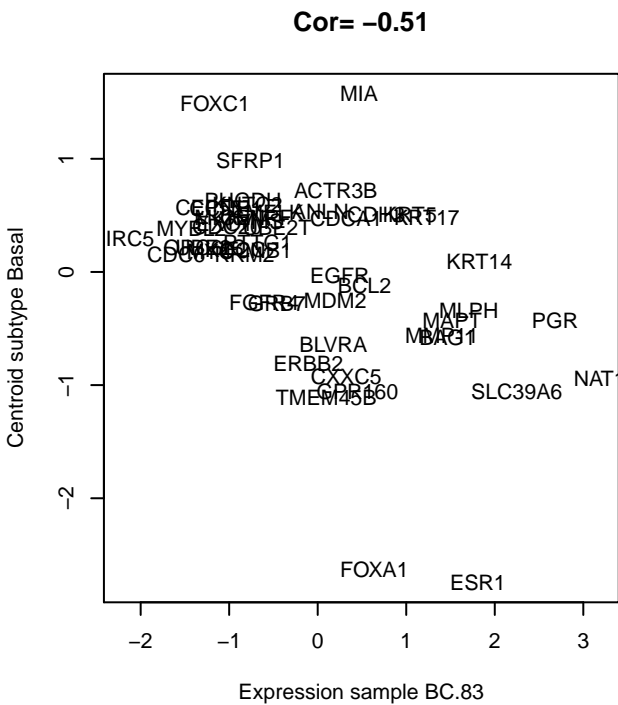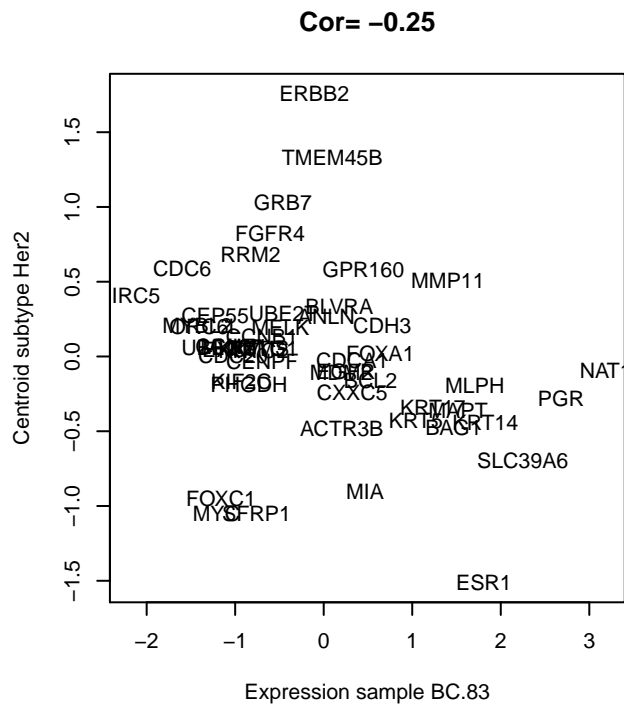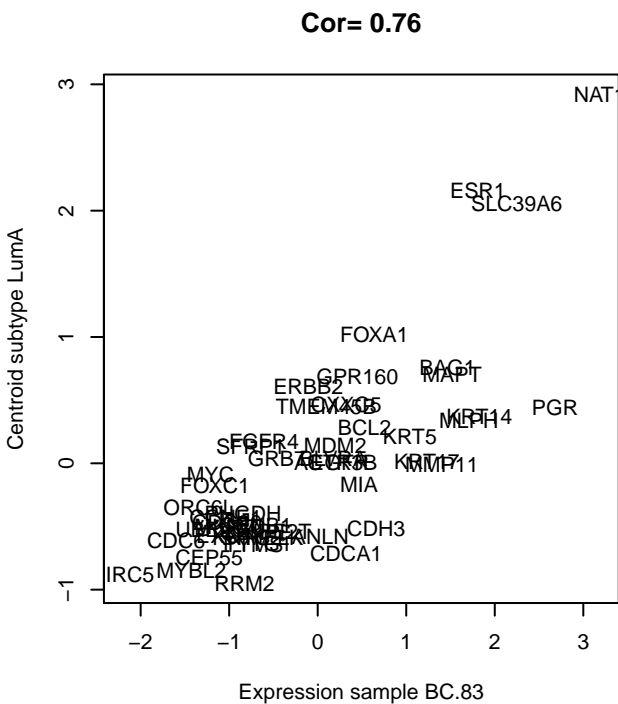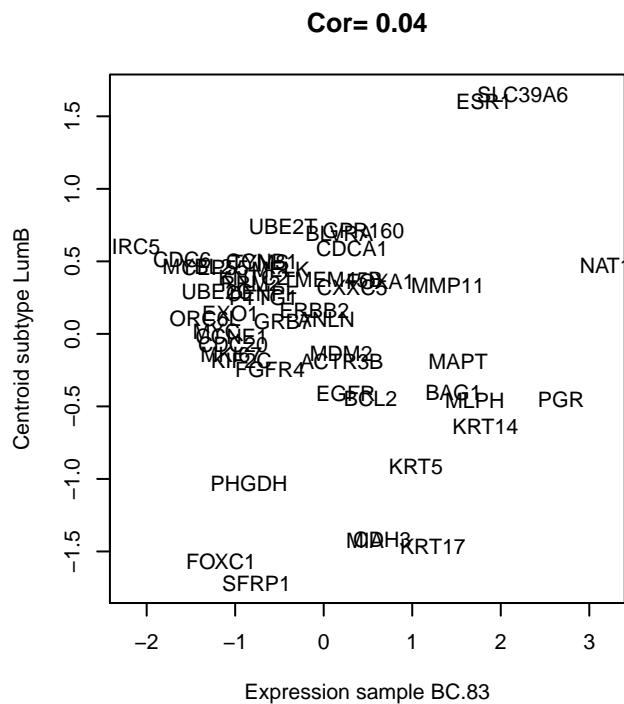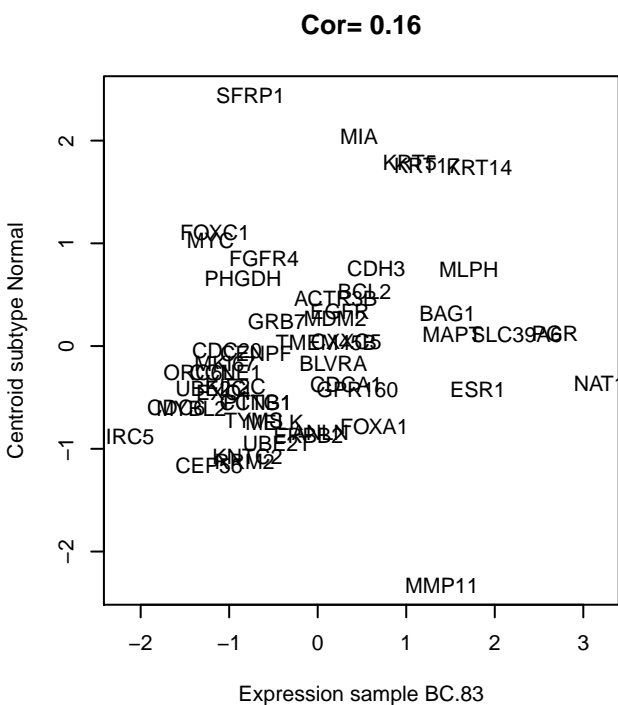

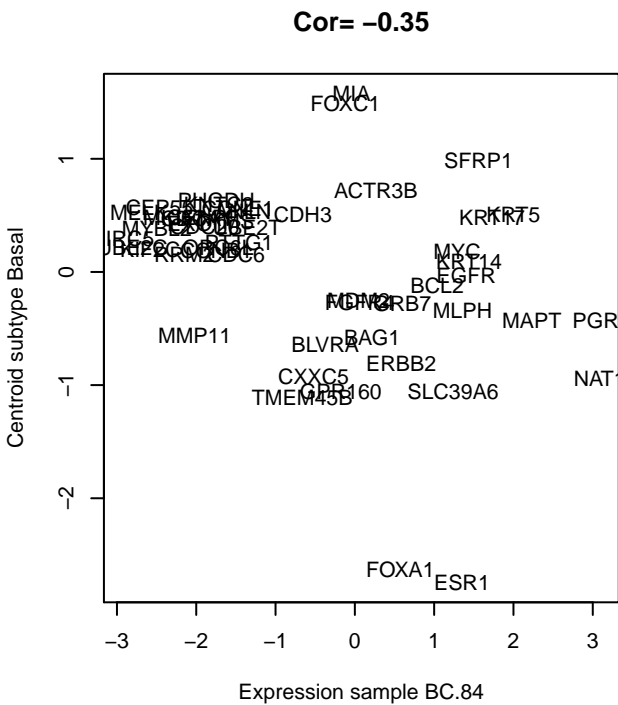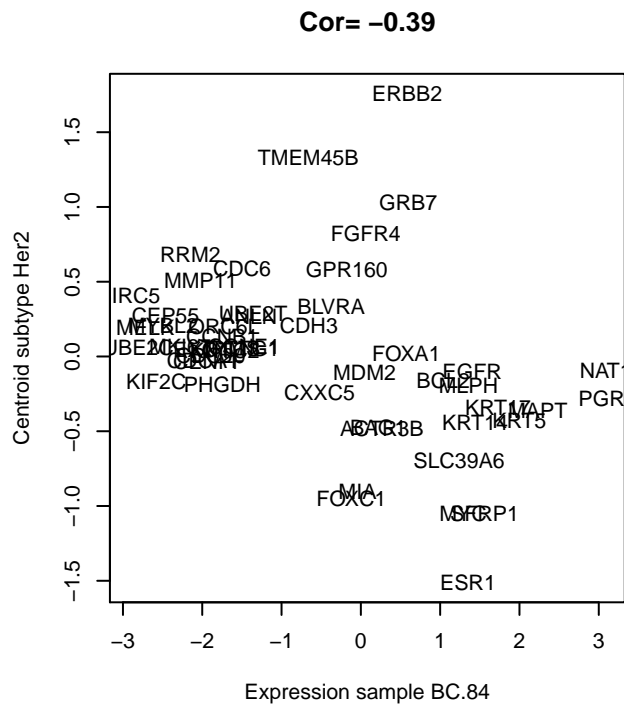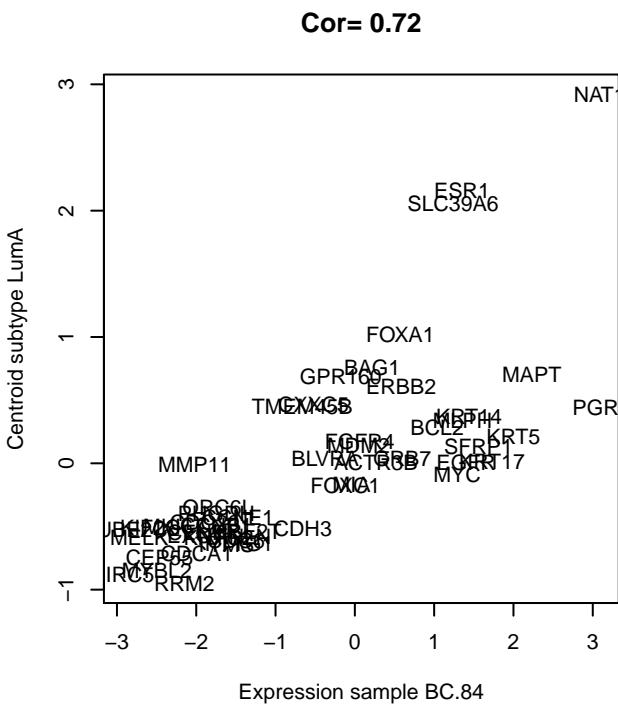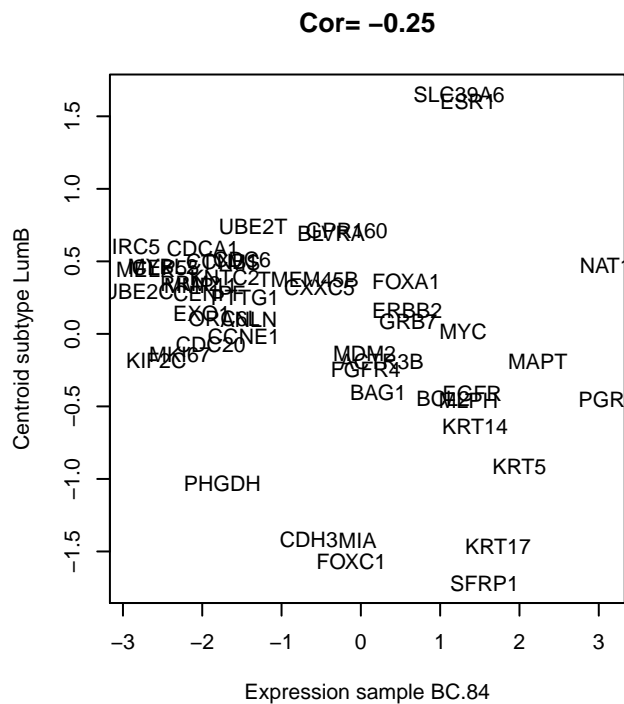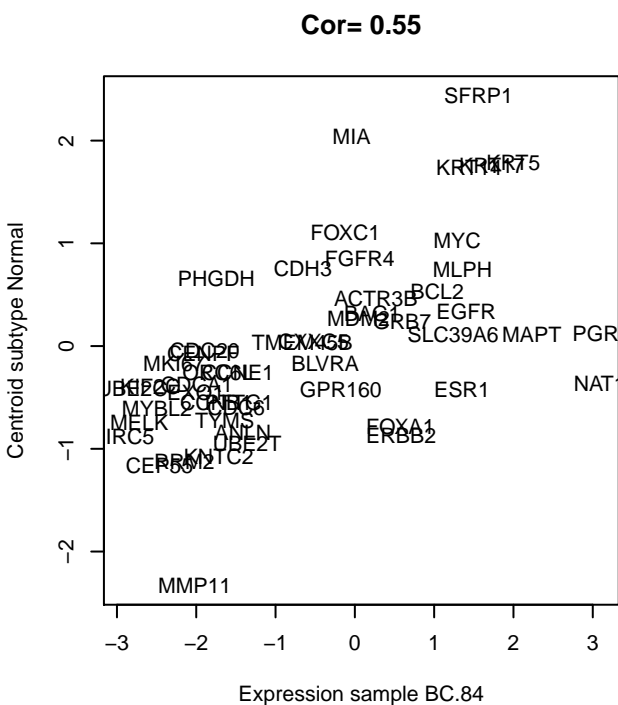

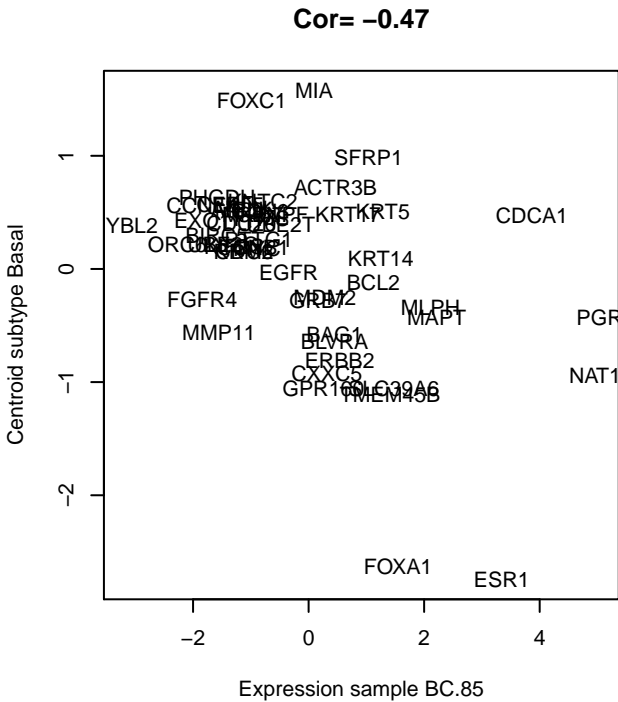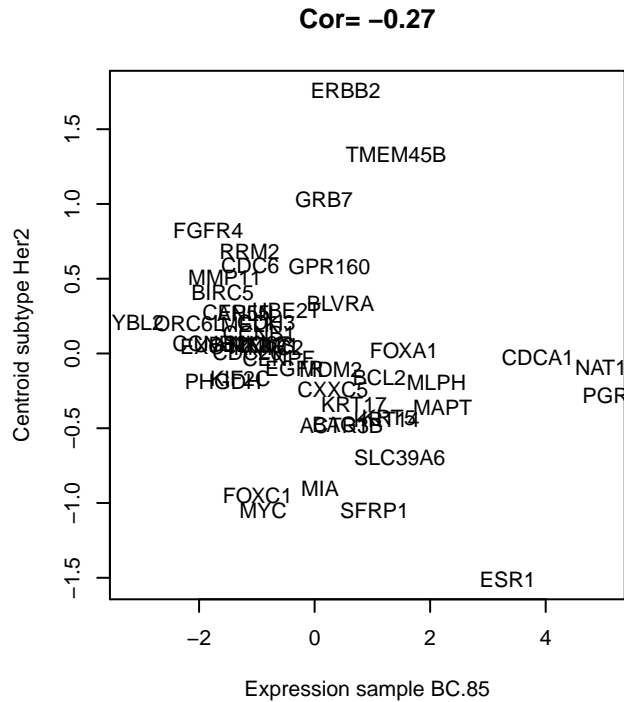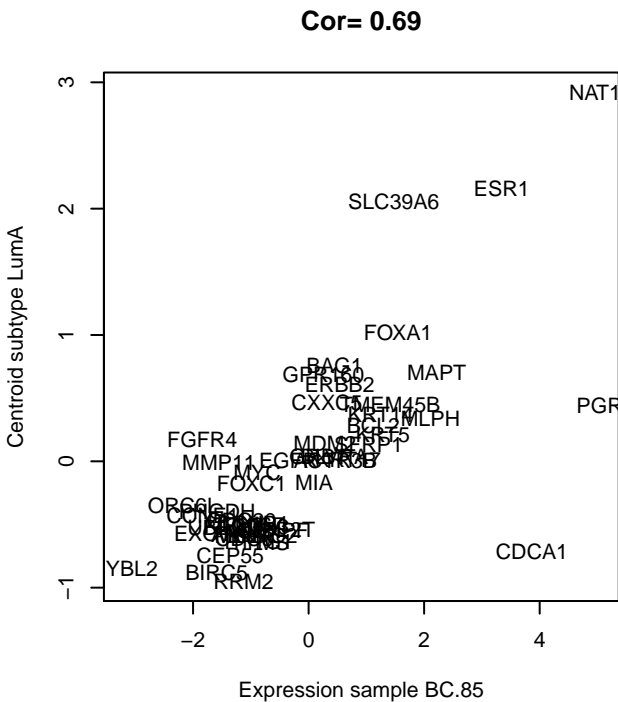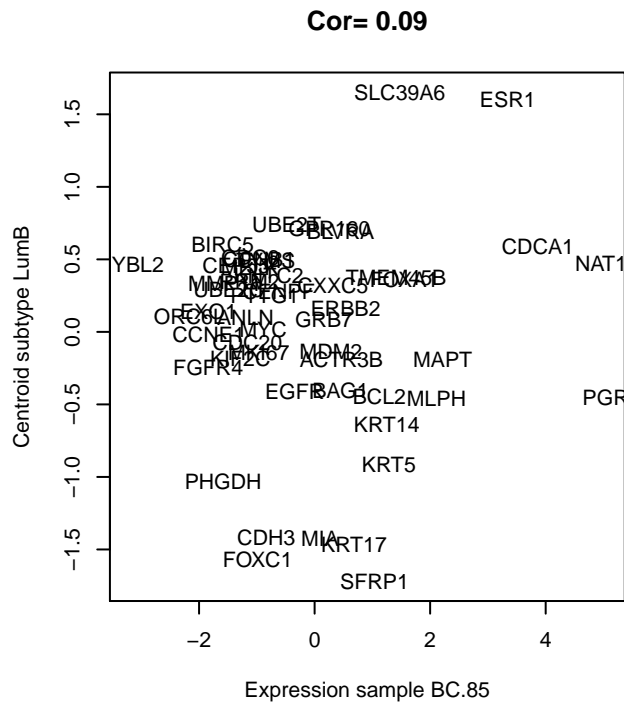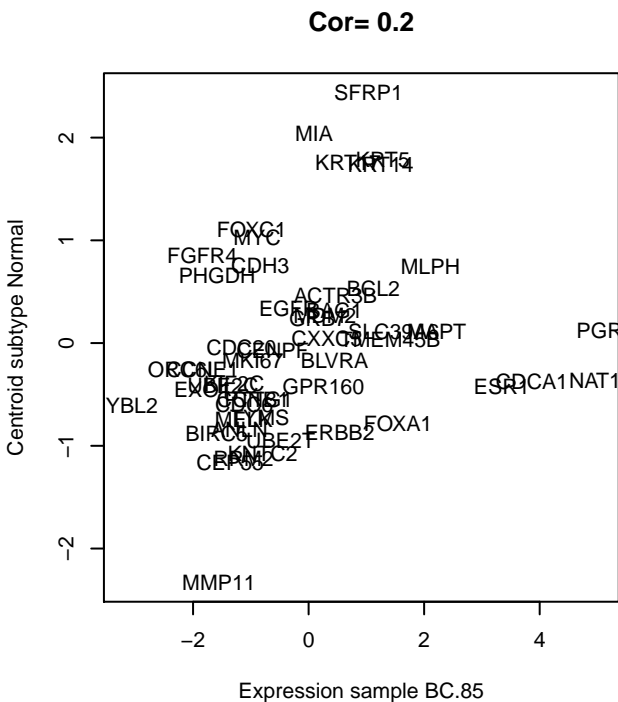



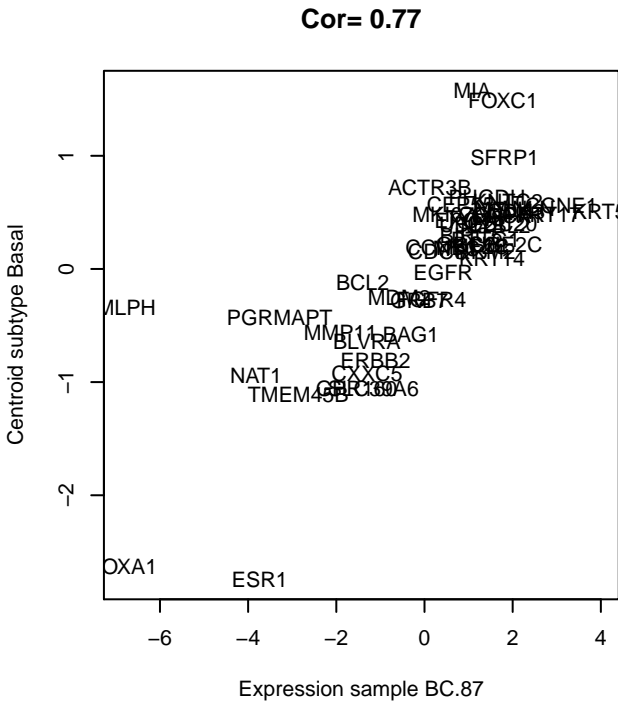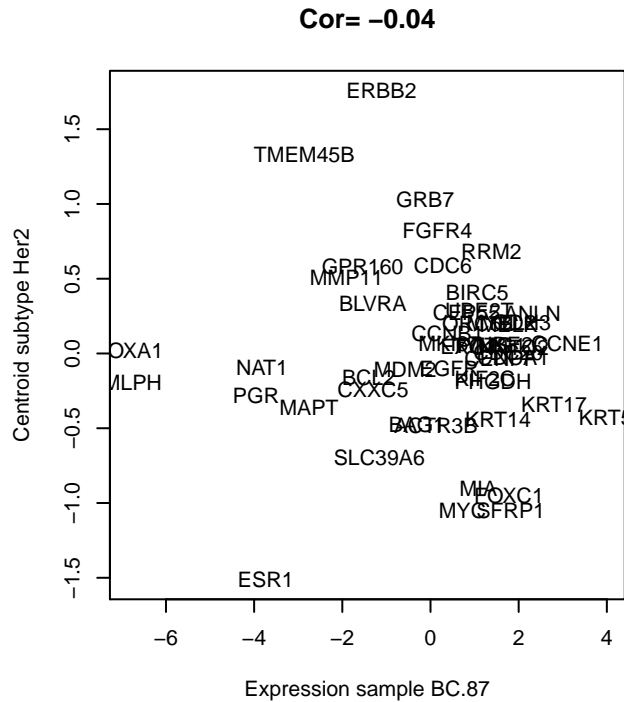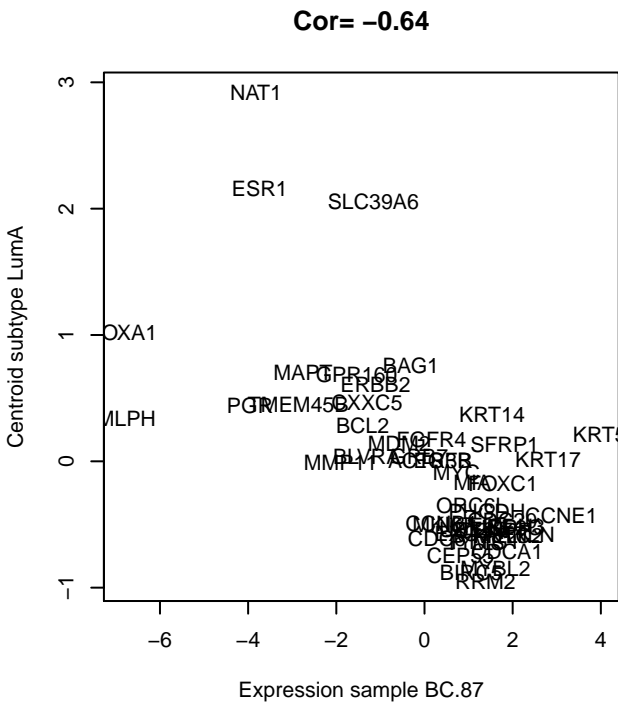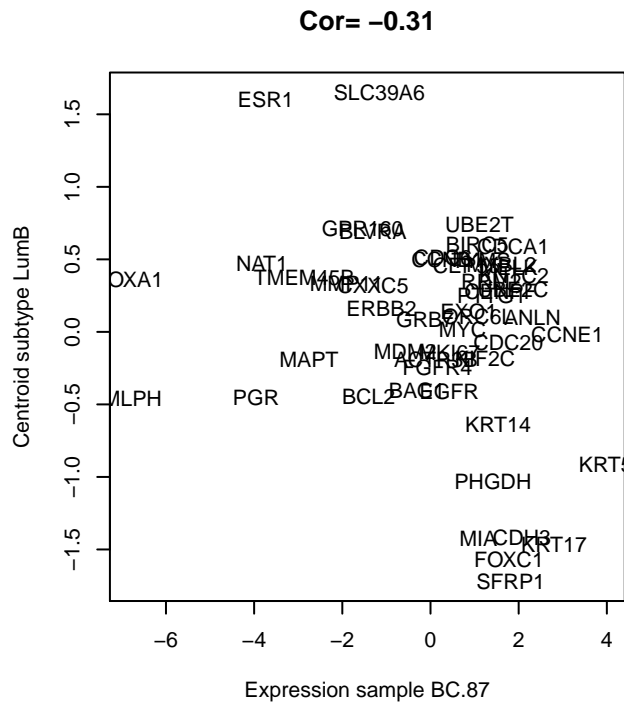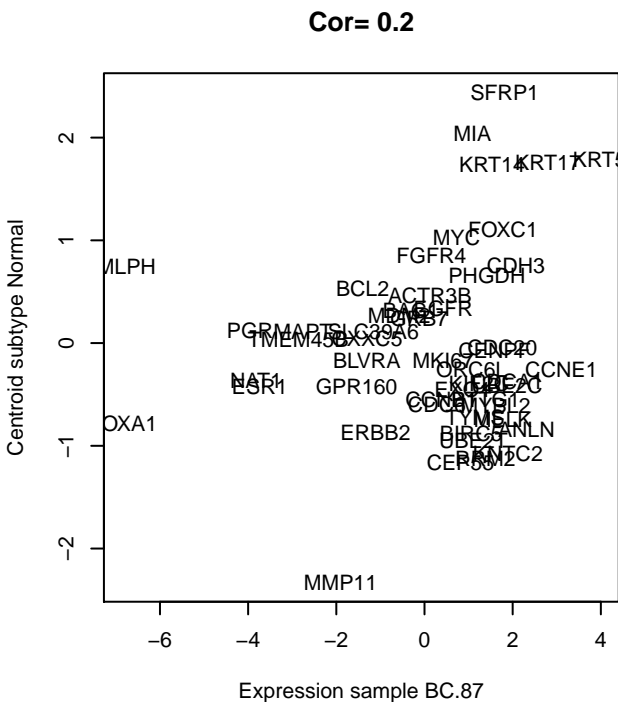

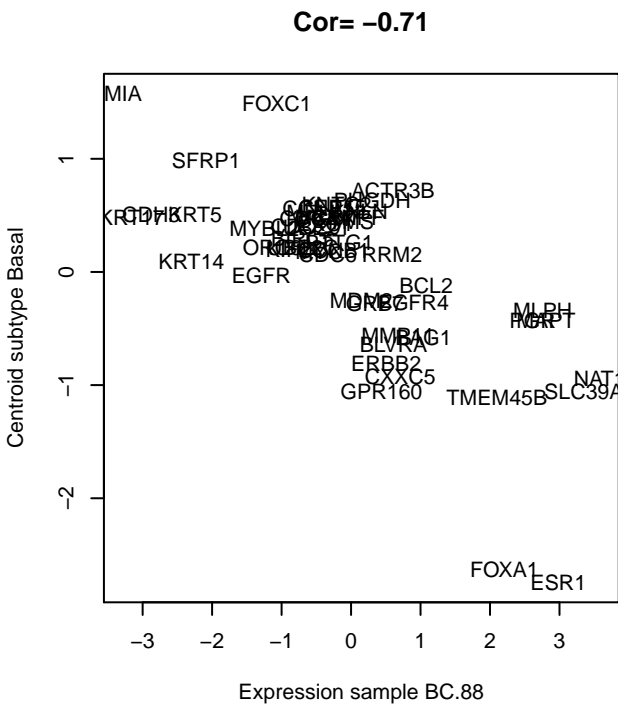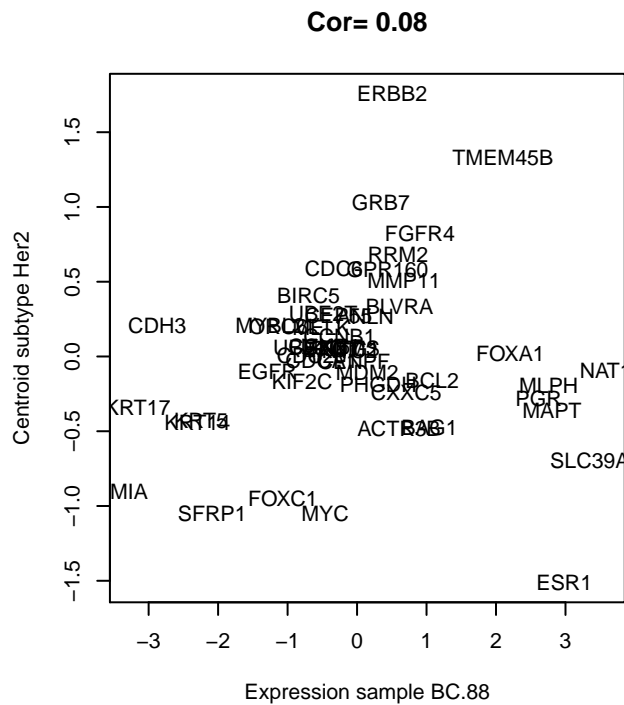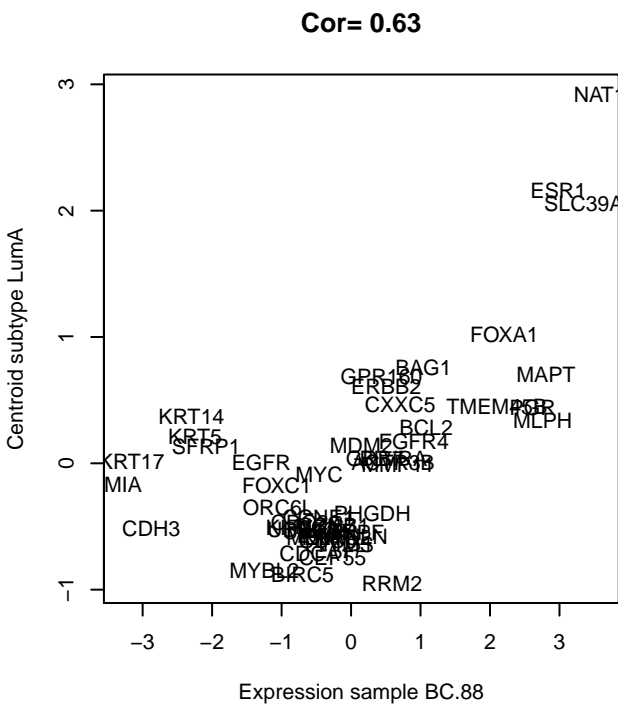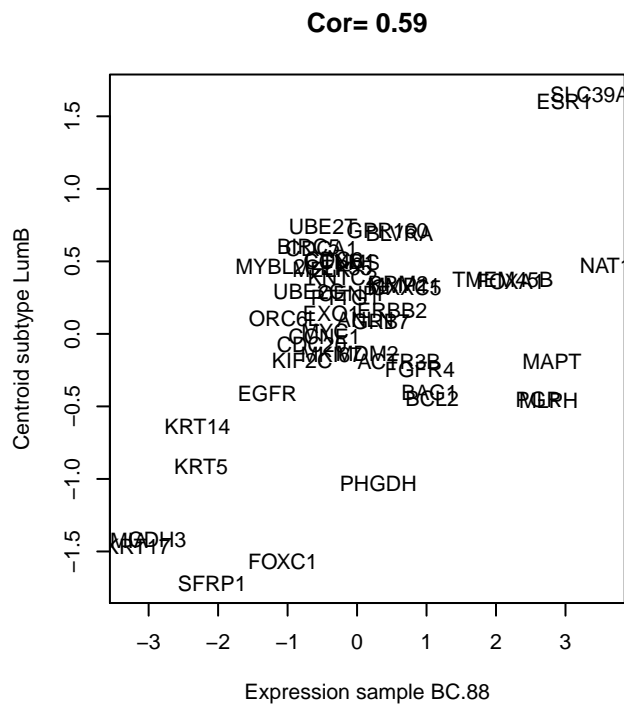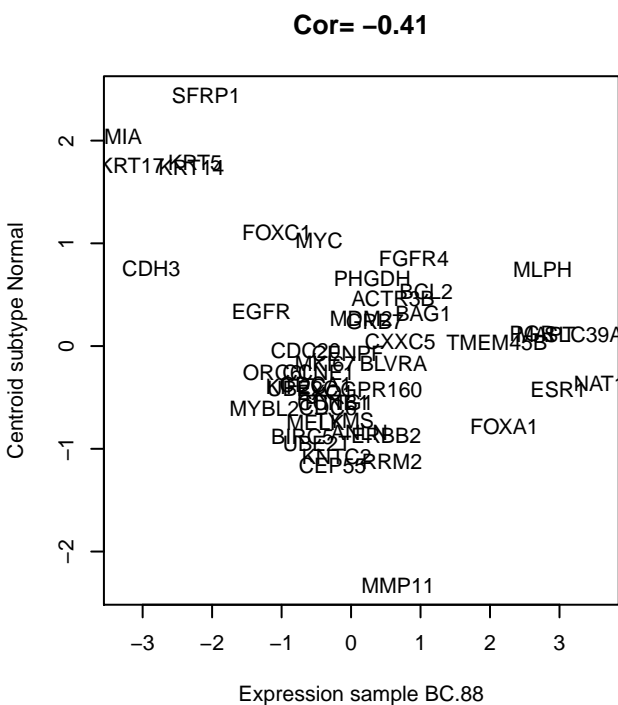

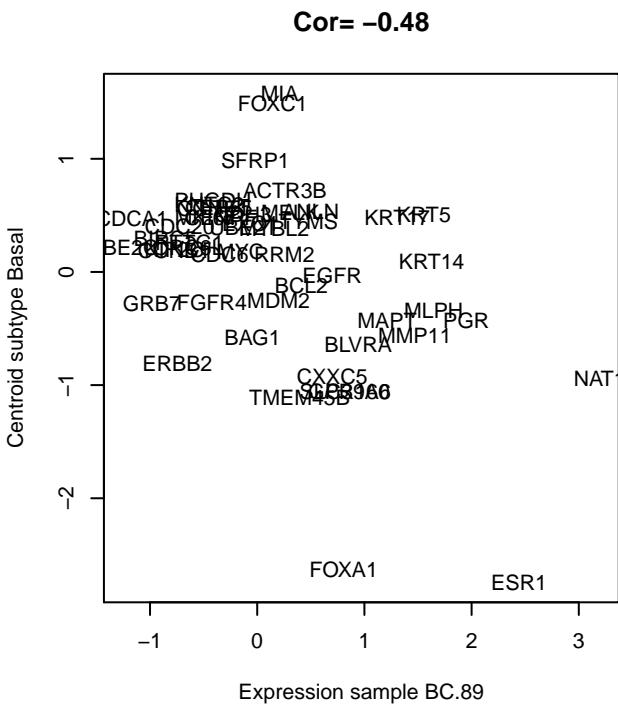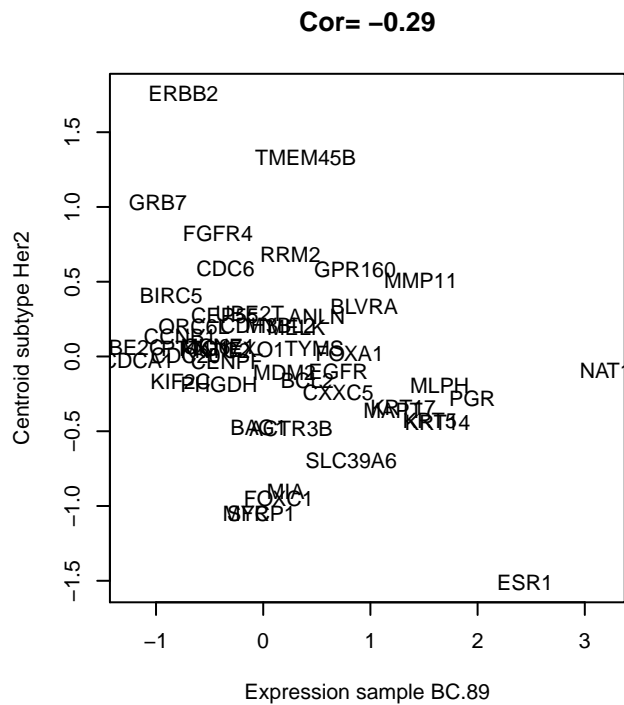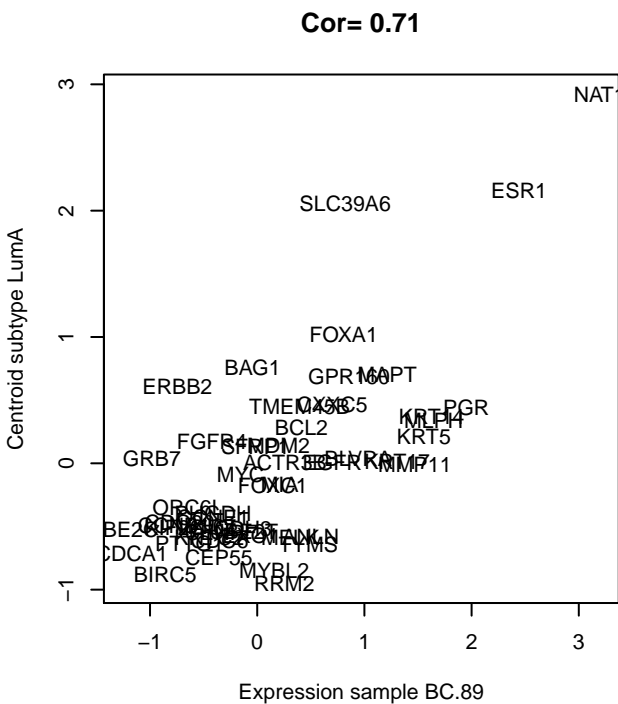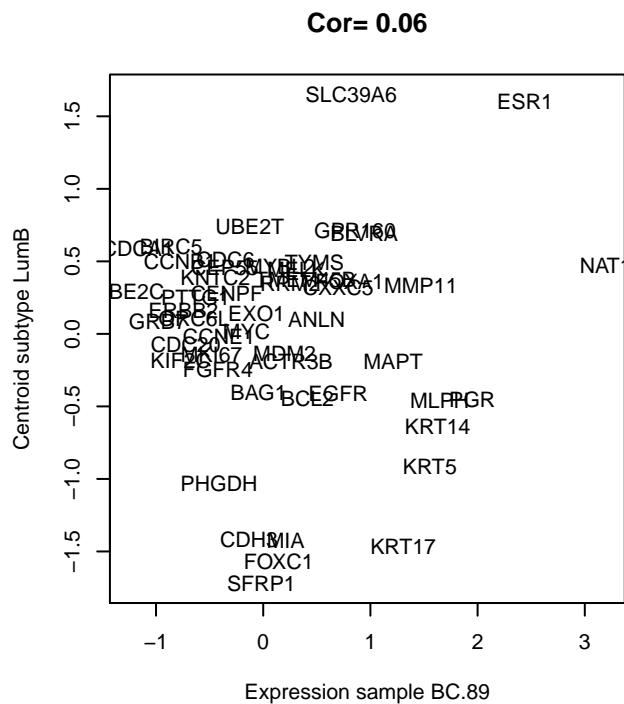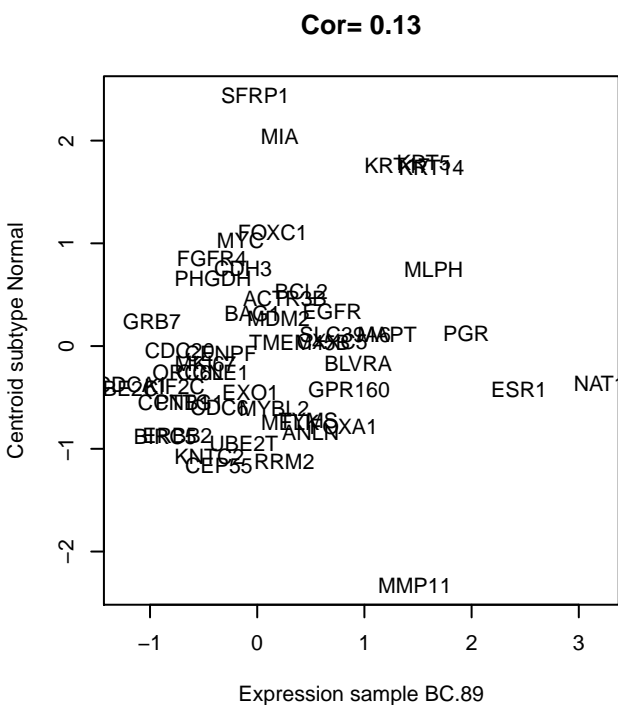

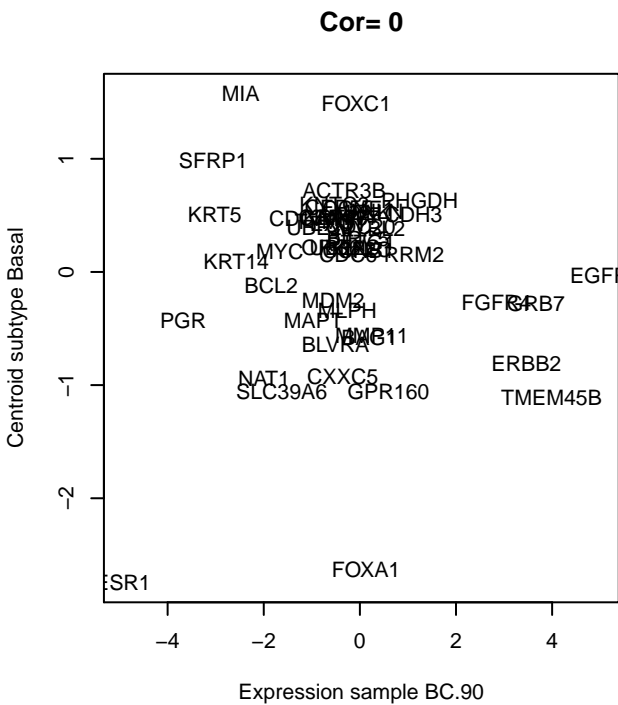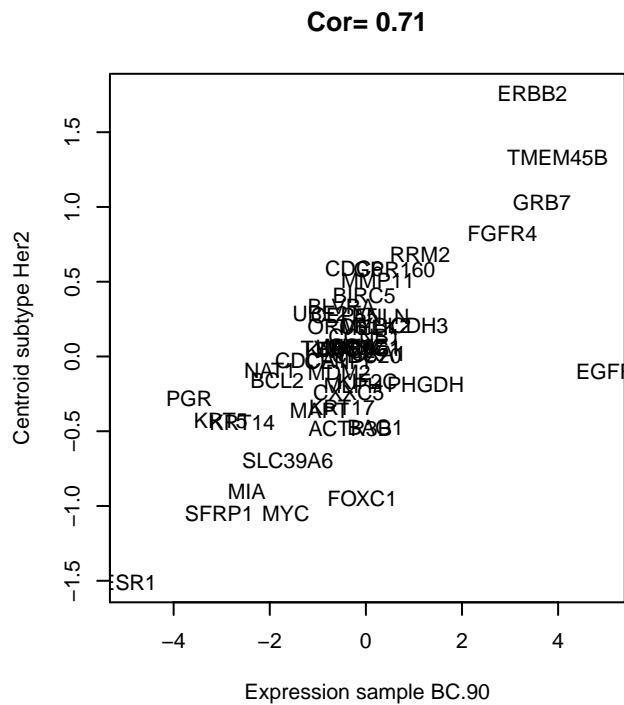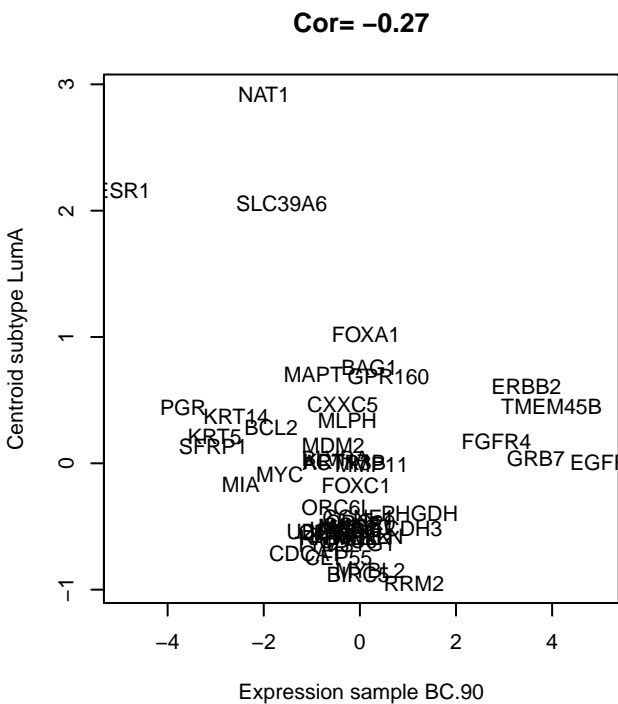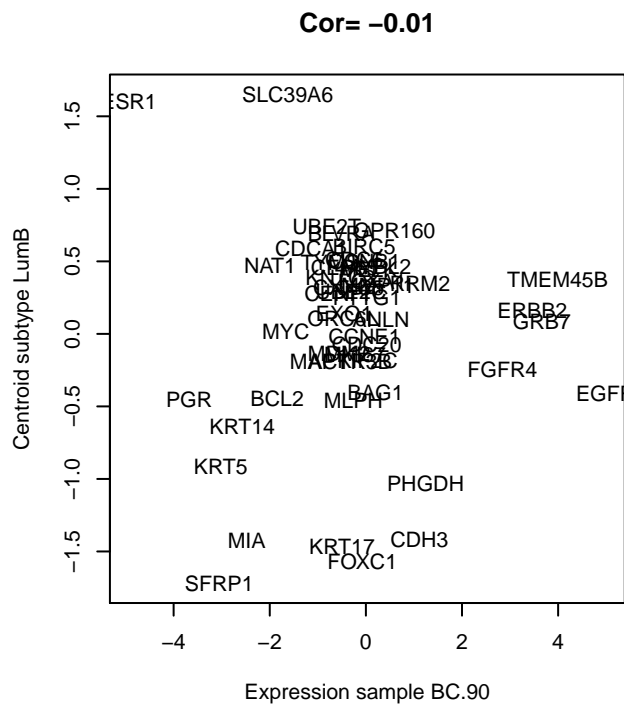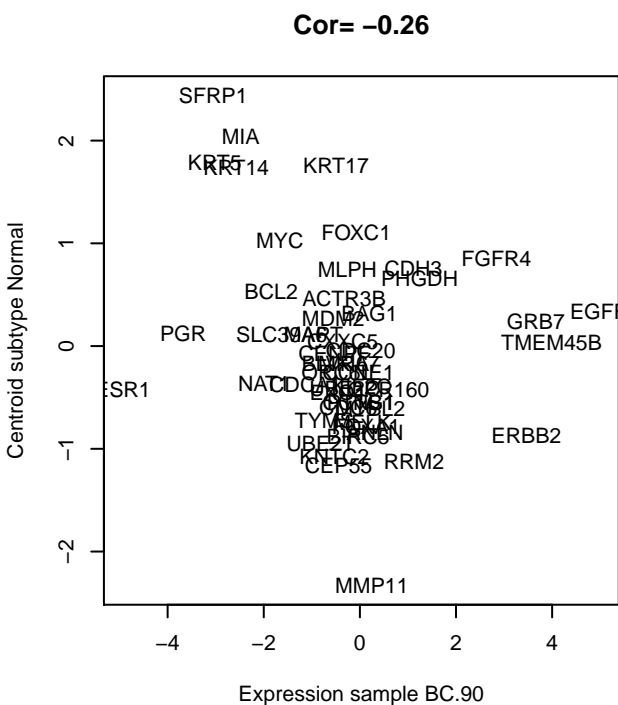

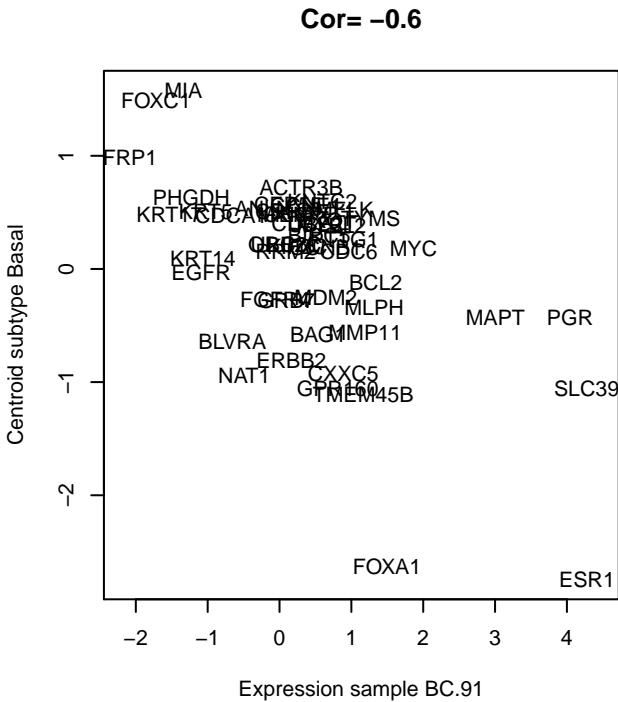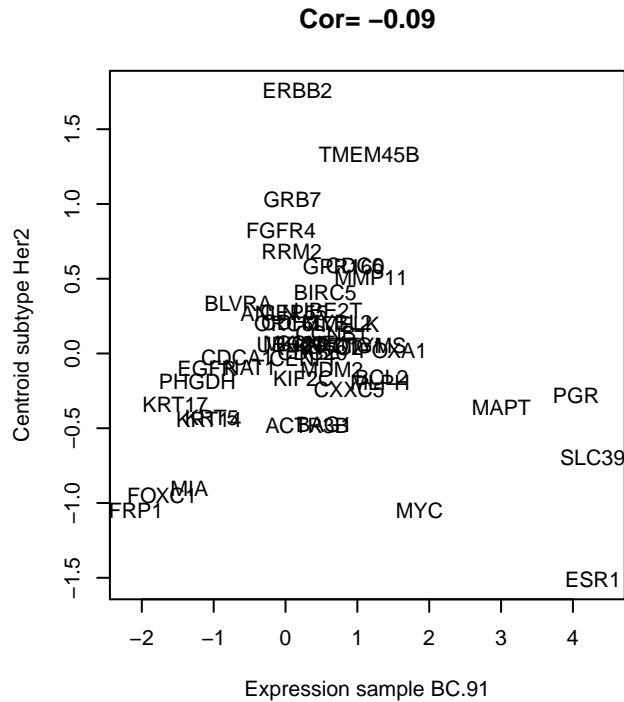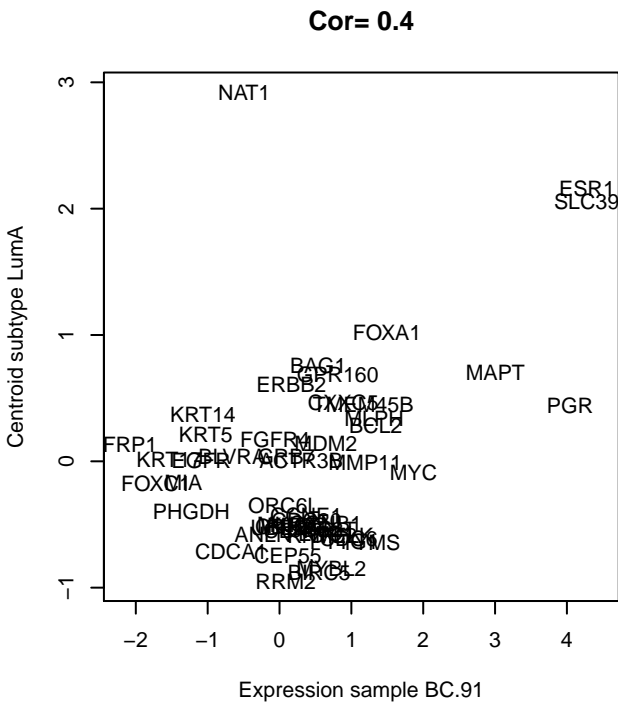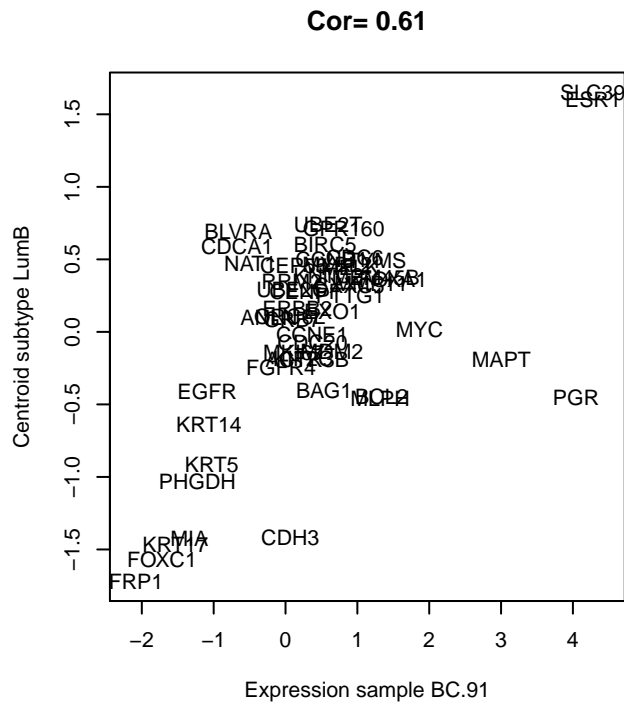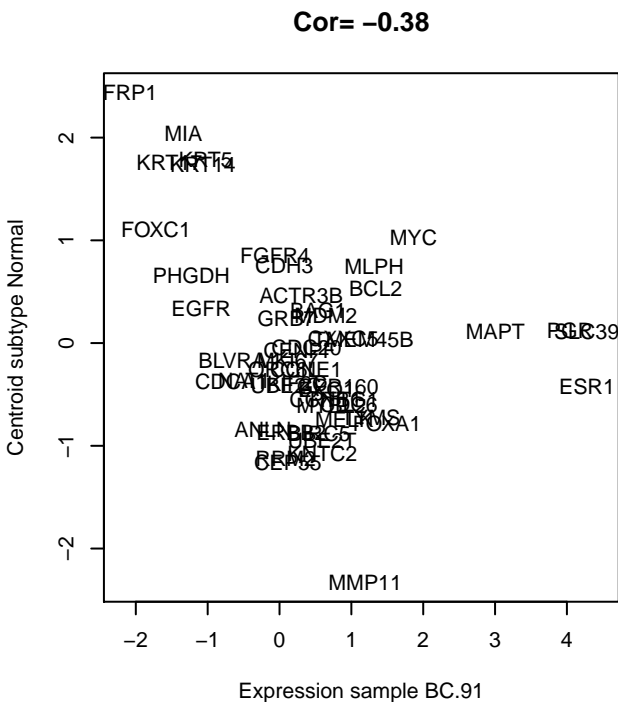

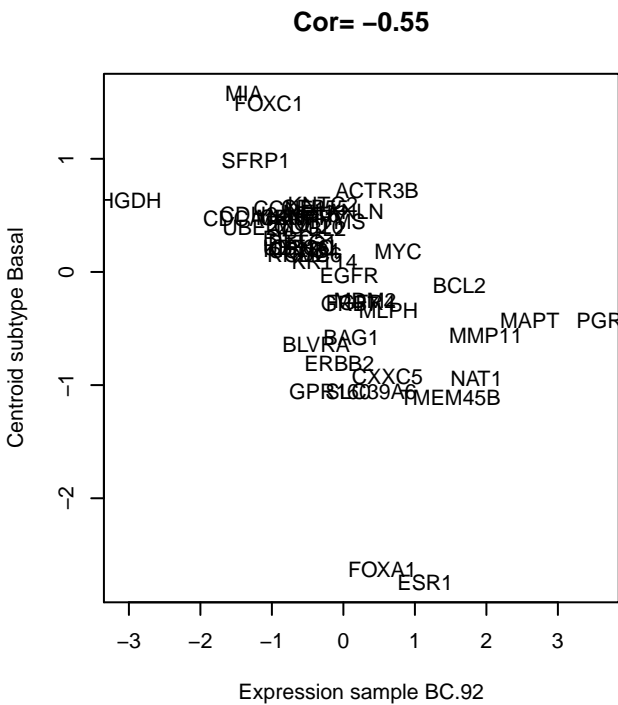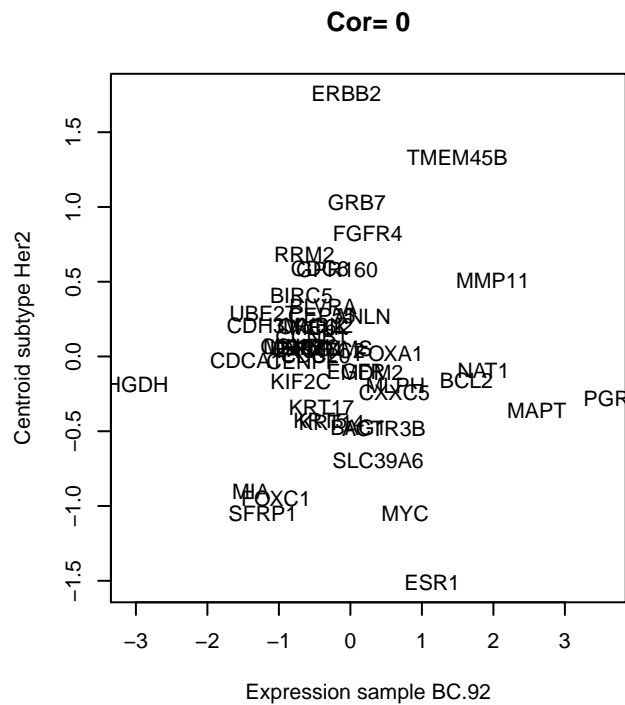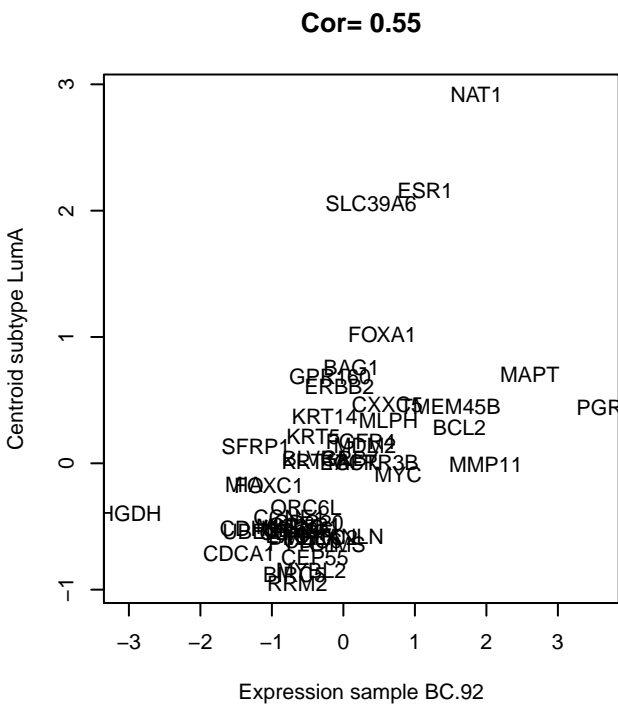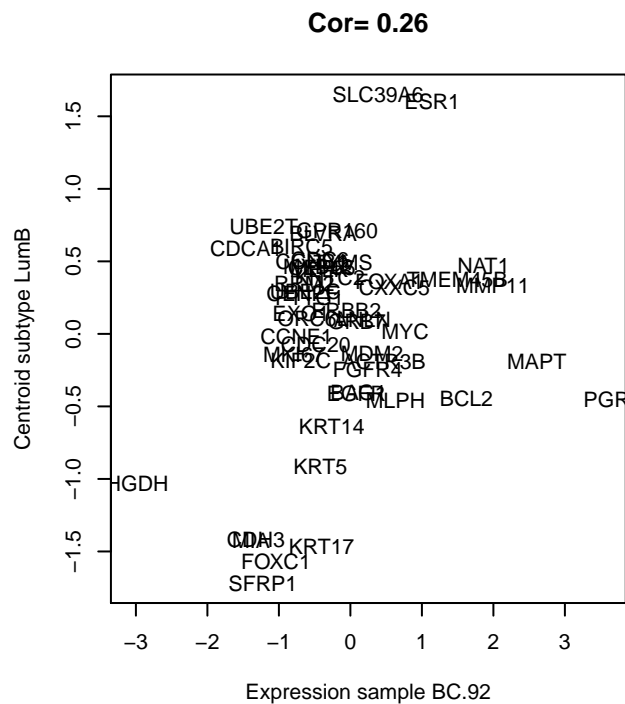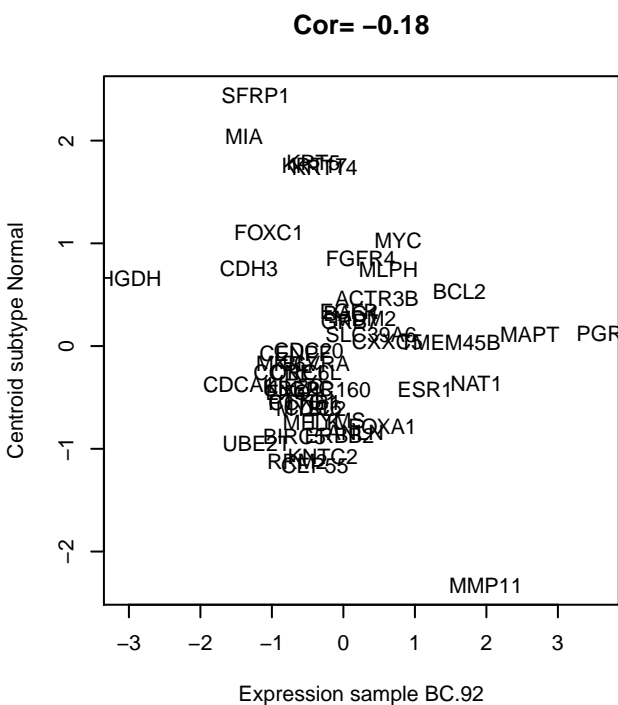

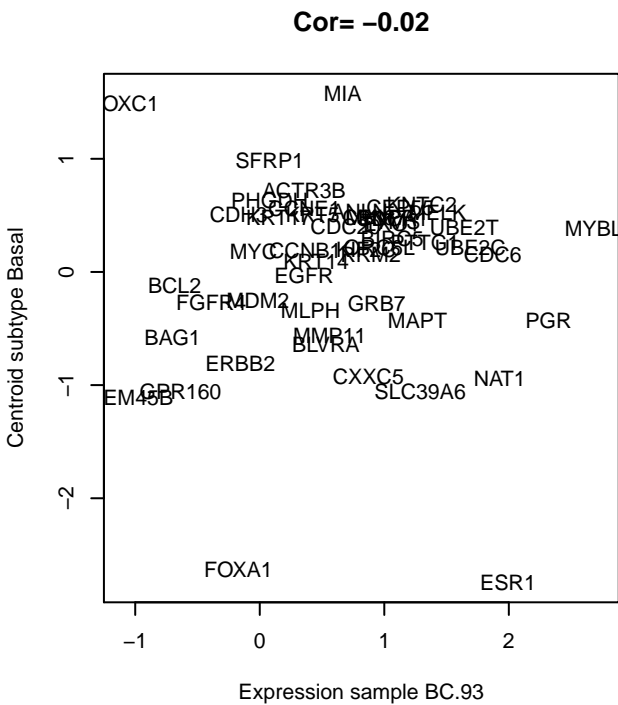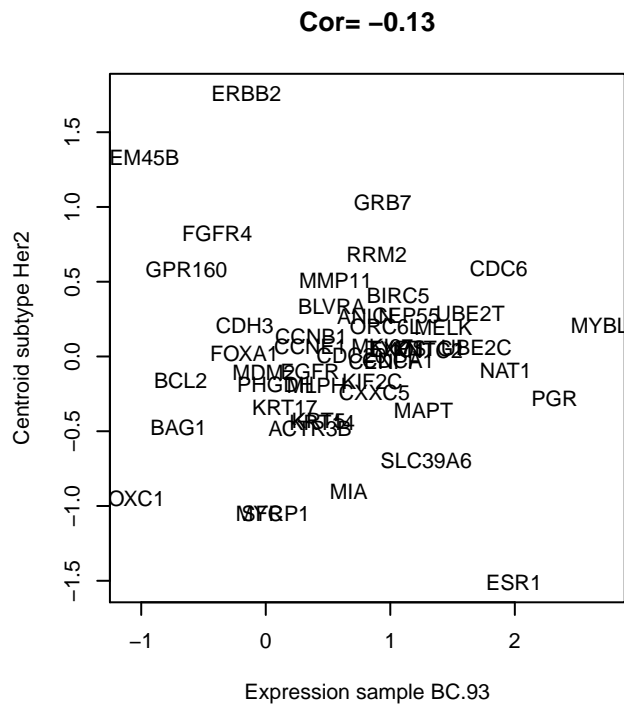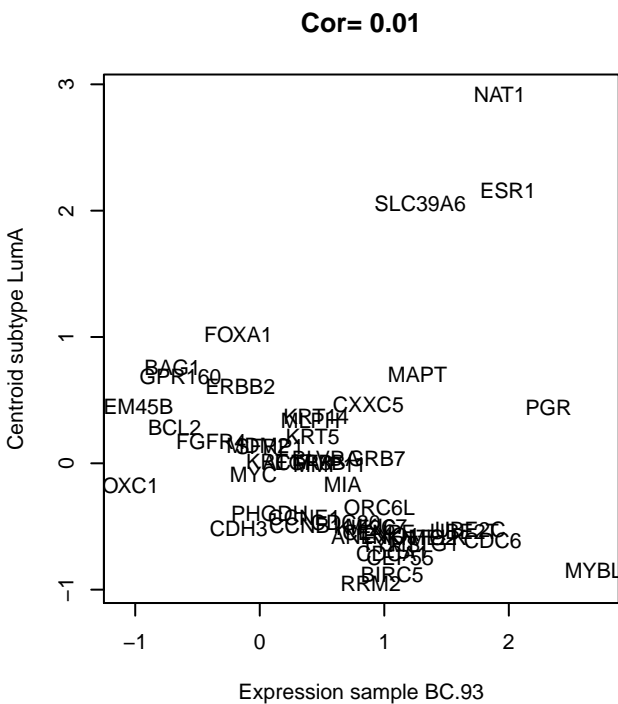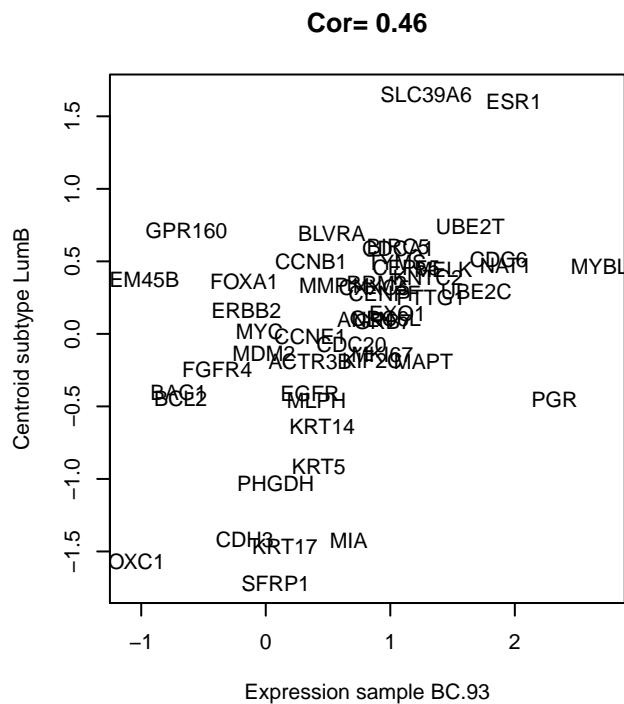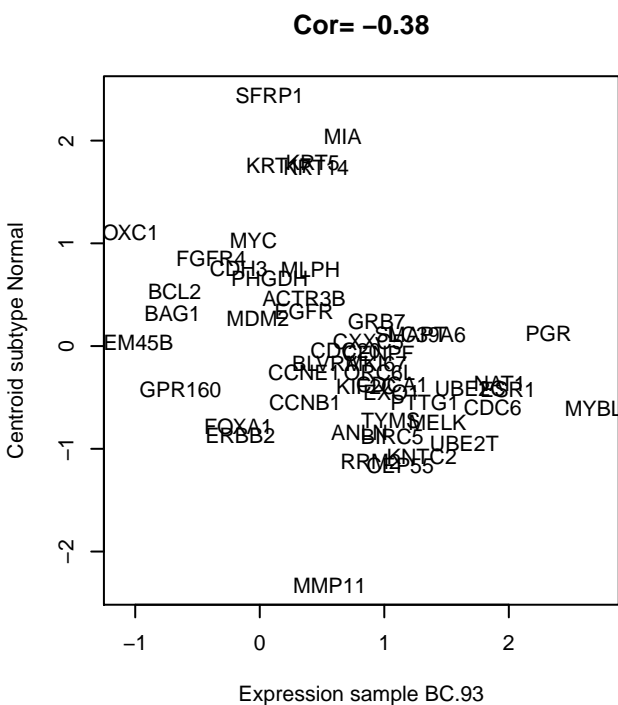

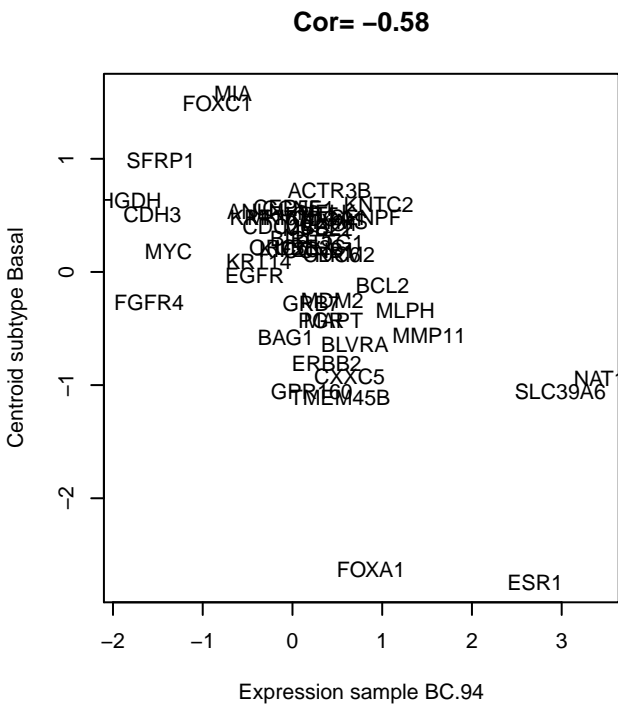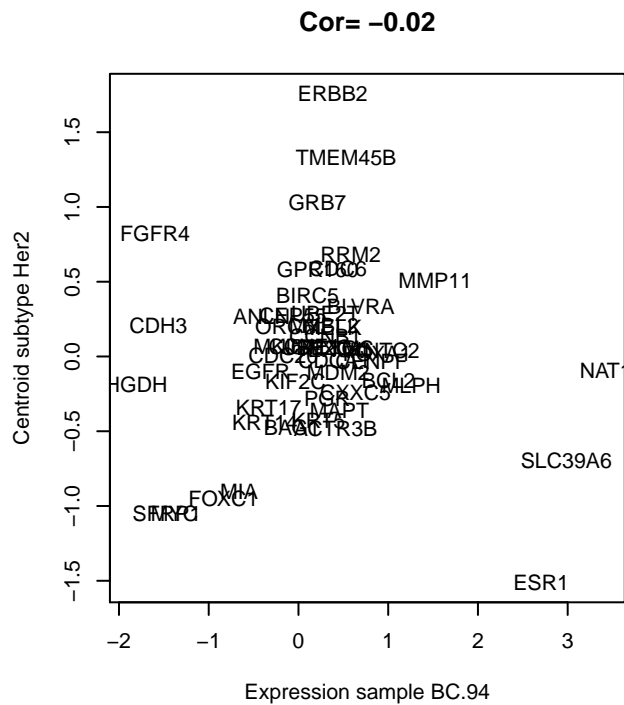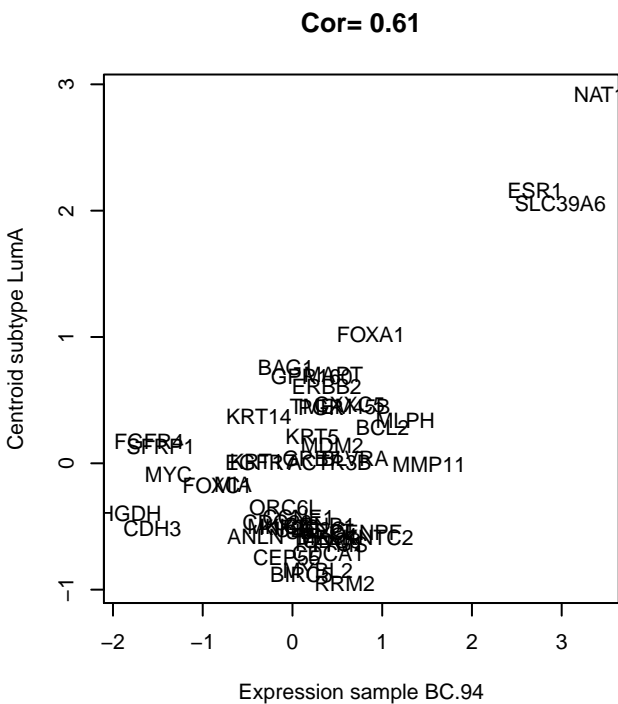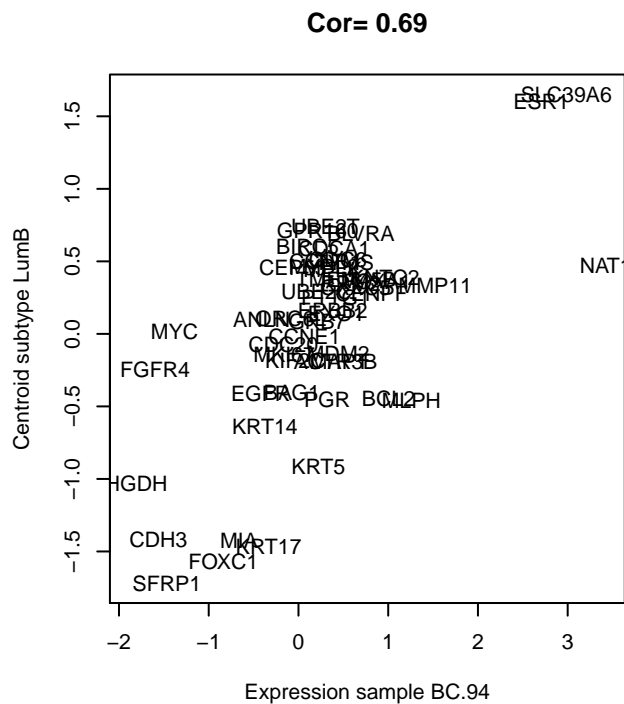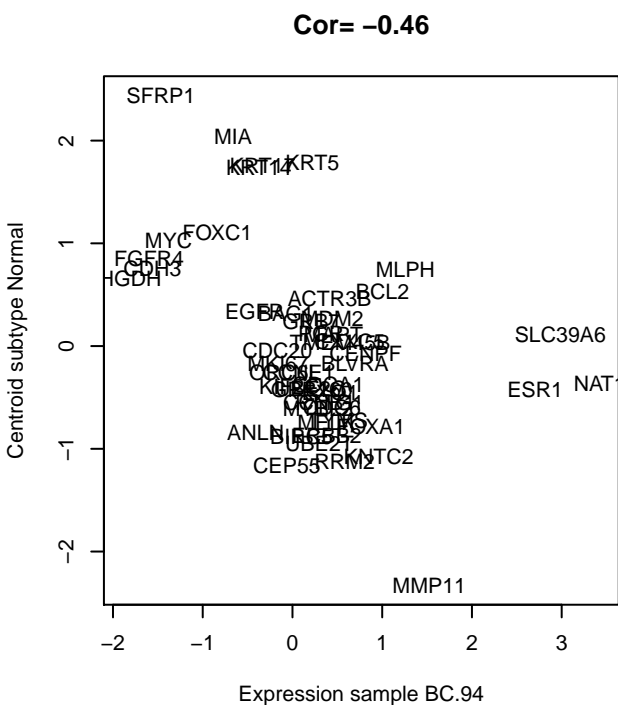

Supplement: Supplementary file 1 [file cancers-13-06118-s001.zip › SupplementaryCode/kodeTilSubmition/dataOutput/macro/ScatterPlot_expressionPerSample_EachCentroid.pdf]
